# How Pandemics Have Reshaped the Respiratory Virus Data Landscape in Europe: Scoping Review

**DOI:** 10.2196/92917

**Published:** 2026-07-17

**Authors:** Brice Mastrovito, Claire Nour Abou Chakra, Cédric Mahé, Marta C Nunes

**Affiliations:** 1 Centre of Excellence in Respiratory Pathogens (CERP), Hospices Civils de Lyon (HCL) and Centre International de Recherche en Infectiologie (CIRI), Équipe Santé Publique, Épidémiologie et Écologie Évolutive des Maladies Infectieuses (PHE3ID), Inserm U1111, CNRS UMR5308, ENS de Lyon, Université Claude Bernard Lyon 1 (UCBL Lyon 1), Bâtiment B – 7ème étage, 7 Rue Guillaume Paradin, Lyon, 69008, France, 33 478771032; 2 Sanofi Vaccines, Lyon, France

**Keywords:** public health surveillance, respiratory virus, influenza, COVID-19, SARS-CoV-2, respiratory syncytial virus, pandemics, Europe, European Health Data Space, EHDS, real world data, data integration, data linkage

## Abstract

**Background:**

Acute respiratory infections caused by influenza, respiratory syncytial virus (RSV), and SARS-CoV-2 remain a major public health challenge in Europe. Although surveillance systems for these pathogens are well established, the past 2 decades have seen a rapid diversification of data streams supporting surveillance and research. This expanding data landscape, combined with fragmentation across institutions, sectors, and countries, may limit timely evidence synthesis and effective public health decision-making.

**Objective:**

This scoping review aimed to identify and characterize data sources used for surveillance and research on influenza, RSV, and SARS-CoV-2 in Europe over the past 20 years, and to examine their evolution over time, their alignment with research objectives, and geographic variation in data availability and use.

**Methods:**

We conducted a scoping review using an objective-driven analytical framework. Empirical reports published between January 2005 and September 2025 were identified in MEDLINE, Web of Science, and Embase. Eligible reports focused on influenza, RSV, or SARS-CoV-2 and included data from 12 European countries. Clinical and interventional studies were excluded. Reports were classified according to 4 research objectives: epidemiological monitoring, evaluation of interventions, assessment of disease burden and health outcomes, and analyses of population adherence and trust toward public health measures. Data sources were grouped into 9 categories, including surveillance systems, electronic health records (EHRs), registries, claims, surveys, digital, environmental, integrated datasets, and others.

**Results:**

A total of 2564 empirical reports were included. Over time, respiratory virus research relied on an increasingly diverse set of data streams. While surveillance systems remained central, particularly for epidemiological monitoring, their relative dominance declined. From 2020 onward, there was a marked expansion in the use of EHRs, registries, claims data, digital sources, and linked or integrated datasets, alongside increased use of open-access data. Data source use varied by research objective: surveillance data predominated in monitoring and intervention evaluation; EHRs in studies of risk factors and treatment effectiveness; surveys in seroprevalence and public trust analyses; and claims data in assessments of economic burden. Substantial geographic disparities were observed. Northern European countries more frequently used linked and multisource datasets, whereas Southern Europe relied more often on open-access or single-source data.

**Conclusions:**

This scoping review provides a multipathogen, cross-country mapping of data sources for respiratory virus surveillance and research in Europe over 2 decades, applying an innovative objective-driven framework. Unlike prior reviews focused on single pathogens or data types, it offers a consolidated, comparative perspective based on 2564 reports to inform public health decision-making. The COVID-19 pandemic accelerated innovation in data generation and access, but progress remained largely centered on SARS-CoV-2, while structural fragmentation continues to limit timely, integrated data across Europe. Strengthening preparedness will require interoperable infrastructures, federated analysis platforms, sustainable funding for surveillance innovations, and cross-sectoral data sharing.

## Introduction

### Rationale

Acute respiratory infections caused by seasonal influenza, respiratory syncytial virus (RSV), and SARS-CoV-2 remain a significant public health burden worldwide, accounting annually for substantial morbidity, mortality, and stress on health care systems [1]. Their impact is more significant in infants [2], older adults [3], and individuals with underlying health conditions [4], and is well characterized through longstanding surveillance systems and widespread diagnostic testing [5,6], while effective vaccines have reduced associated morbidity and mortality [7]. Beyond these pathogens, other respiratory viruses such as rhinoviruses, human metapneumovirus, and adenoviruses contribute to the burden of respiratory infections, but the limited routine testing for these viruses and the absence of treatments and vaccines make their burden estimates unequal [8,9].

Influenza, RSV, and SARS-CoV-2 share key epidemiological characteristics, including droplet and aerosol transmission, distinct seasonal patterns, and the potential to trigger rapidly evolving epidemics [10,11]. In the winter of 2022, the simultaneous circulation of the 3 viruses, following the lifting of COVID-19–related restrictions, notably led to a “triple-demic” scenario, resulting in increased hospitalizations and considerable pressure on health care infrastructures [12]. More recently, the emergence of H5N1 avian influenza outbreaks in over 200 dairy cattle herds during the 2024‐2025 season in the United States has heightened concerns regarding future pandemics, highlighting the critical need for enhanced preparedness and surveillance mechanisms [13].

Monitoring the spread and impact of respiratory viruses increasingly relies on multiple, complementary data sources, each providing distinct insights into transmission dynamics, disease severity, and population-level patterns in incidence and spread [14]. Across Europe, numerous initiatives and data sources exist, leveraging a variety of information ranging from hospital data to primary care records, syndromic surveillance systems, laboratory testing, and emerging sources such as digital or environmental data [15]. However, despite the availability of multiple sources, heterogeneity and dispersion of data, together with fragmented institutional and governance arrangements for data production, stewardship, and access, frequently impede the timely and efficient synthesis of information necessary to guide public health decision-making [16].

Several reviews have examined specific dimensions of this landscape. Hammond et al [17] reviewed influenza surveillance systems using both traditional and alternative data sources; Clark et al [18] documented changes to public health surveillance methods during the COVID-19 pandemic; and Liu and Panagiotakos [16] assessed the role of real-world data in health research. Mollers et al [19] surveyed current practices for RSV surveillance across Europe, while Sweileh [20] mapped the research landscape on emerging respiratory infections worldwide. However, existing reviews have typically focused on single pathogens, specific data types, or particular surveillance mechanisms in isolation. There remains a gap in the literature for an integrated, cross-pathogen perspective on how the data landscape underpinning respiratory virus surveillance and research has evolved across Europe over the past 2 decades, through 2 major pandemics.

### Objectives

Given the growing complexity of the generation and utilization of data across Europe, a comprehensive description of available sources is essential to better understand the breadth of surveillance- and research-related data to support stronger decision-making. This scoping review aims to identify and characterize the data sources used to support the surveillance and research of influenza, RSV, and SARS-CoV-2 over the past 2 decades across 12 European countries. By providing a consolidated overview of existing practices and capacities, the review seeks to inform future policy, support strategic planning, and guide the development of sustainable, integrated, and resilient data infrastructures for respiratory virus surveillance and research in Europe.

## Methods

### Protocol and Registration

This scoping review was reported in accordance with the PRISMA-ScR (Preferred Reporting Items for Systematic Reviews and Meta-Analyses extension for Scoping Reviews). A review protocol defining the review objectives, eligibility criteria, search strategy, and analytical framework was developed and agreed upon by the review team prior to conducting the review. The protocol was not registered in a public registry. A completed PRISMA-ScR checklist is provided in Checklist 1.

### Eligibility Criteria

We included reports published between January 2005 and September 2025, a period selected to capture the evolution of data sources in response to 2 major public health crises, notably the 2009 H1N1 influenza pandemic and the COVID-19 pandemic. The selection of countries aimed to reflect a range of health care system contexts and levels of maturity in respiratory virus surveillance and data systems across Europe. Accordingly, countries with the highest publication output in each major European region were retained, while ensuring adequate geographic representation through the inclusion of at least 2 countries per region. The final sample included 12 countries: Denmark, England, Finland, and Sweden (Northern Europe); Poland and Romania (Eastern Europe); Italy and Spain (Southern Europe); France, Belgium, Germany, and the Netherlands (Western Europe). Eligible reports met the following criteria: (1) empirical reports presenting original quantitative or qualitative data, (2) reporting on influenza, RSV, or SARS-CoV-2 as the primary pathogen(s) of interest, (3) including data from at least one of the 12 selected European countries, and (4) including a minimum of 50 participants, when applicable, to maximize population-level relevance and exclude case reports or small case series. For multicountry reports, data for the 12 selected countries were retained if separately identifiable within the report. We excluded nonempirical works (eg, literature reviews, systematic reviews, meta-analyses, commentaries, editorials, conference abstracts, and letters), interventional studies (randomized controlled trials), and preclinical or laboratory-only reports. No language restriction was applied.

### Information Sources

The literature search was conducted in 3 bibliographic databases: MEDLINE (via PubMed), Web of Science, and Embase. Each database was searched from its inception to the date of the last search. The initial search was run on February 14, 2025, and updated on September 3, 2025. No additional information sources were used.

### Search

The full electronic search strategy for all 3 databases, including all search terms and the limits applied, is provided in Multimedia Appendix 1 (Table S1). The search combined title-field terms structured around 3 conceptual blocks: (1) study focus and outcome terms, (2) target pathogens (influenza, SARS-CoV-2, and RSV), and (3) the 12 target countries, together with exclusion terms removing nonempirical publication types and unrelated topics. The search strategy was reported in accordance with the PRISMA-S (Preferred Reporting Items for Systematic Reviews and Meta-Analyses literature search extension) guidelines for reporting literature searches [21]. A completed PRISMA-S checklist documenting the reporting of the literature search is provided in Checklist 2.

### Selection of Sources of Evidence

Following the search, all records were imported into Systematic Review Accelerator, a reference management and screening software, and duplicates were removed prior to screening [22]. Two reviewers (BM and CNAC) independently screened the titles and abstracts of all identified reports to assess their relevance. The full texts of potentially relevant articles were then reviewed by BM, with verification by CNAC, based on the predefined inclusion criteria. Disagreements were resolved through discussion between the 2 reviewers, with MCN serving as an arbiter when consensus could not be reached.

### Data Charting Process

Data charting was performed by 1 reviewer (BM) and verified by a second reviewer (CNAC). Internal consistency checks were conducted throughout the data extraction process. Each report was classified based on its primary stated research objective. The primary objective was identified through a three-step procedure: (1) the principal aim explicitly stated at the end of the introduction, (2) the focus of the principal analysis, and (3) the framing of the conclusions. These 3 elements were examined jointly. In case of discordance, the aim stated as principal in the introduction prevailed, as it reflects the authors’ own framing of the study’s purpose and is least susceptible to being shaped by unanticipated findings. When a report addressed multiple objectives, the dominant one, as determined by the report’s stated aim and main analysis, was retained according to the same procedure.

### Data Items

For each included report, we charted the primary research objective and subobjective, the type(s) of data source used to address that objective, and the accessibility of each data source. Reports were categorized according to four main research objectives, each comprising predefined subobjectives: (1) epidemiological monitoring (incidence, seroprevalence, and the identification of viral variants and subtypes), (2) evaluation of intervention effectiveness and impact (immunization, public health and social measures, and therapeutic strategies), (3) assessment of disease burden and health outcomes (morbidity, economic burden, and risk factors), and (4) analyses of population adherence and trust toward public health measures (coverage and public trust). The definition of these 4 objectives and their subobjectives was guided by a preliminary review of the literature and iteratively refined during the protocol development phase to capture the full breadth of research purposes observed in respiratory virus studies. Further details on the classification of reports according to their objectives and subobjectives are provided in Multimedia Appendix 1 (Tables S2 and S3). Data sources were classified into 9 categories adapted from the classification proposed by Makady et al [23]: electronic health records (EHRs), registries, claims databases, surveillance data, surveys, environmental data, digital data (eg, mobility, social media), integrated datasets (explicit linkage of 2 or more sources within the same environment), and other (open-access sociodemographic datasets, scientific literature, etc). In addition, data sources were classified according to their accessibility as open-source or non–open-source. The detailed definitions of each category are provided in Multimedia Appendix 1 (Table S4).

### Critical Appraisal of Individual Sources of Evidence

In line with established scoping review methodology, no formal critical appraisal or risk-of-bias assessment of the included sources of evidence was performed [24]. Consistent with the descriptive mapping objective of this review, the aim was to document the data sources used for respiratory virus surveillance and research rather than to evaluate the methodological quality of individual reports or the performance of individual data sources.

### Synthesis of Results

Charted data were synthesized descriptively. Quantitative analysis was only descriptive; no statistical associations or hypothesis testing were conducted. We visualized temporal trends, distributions of data source types, and cross-country comparisons using stacked bar charts and flow diagrams. Data analysis was performed using R (version 4.4.3) [25].

## Results

### Selection of Sources of Evidence

**Figure 1.**
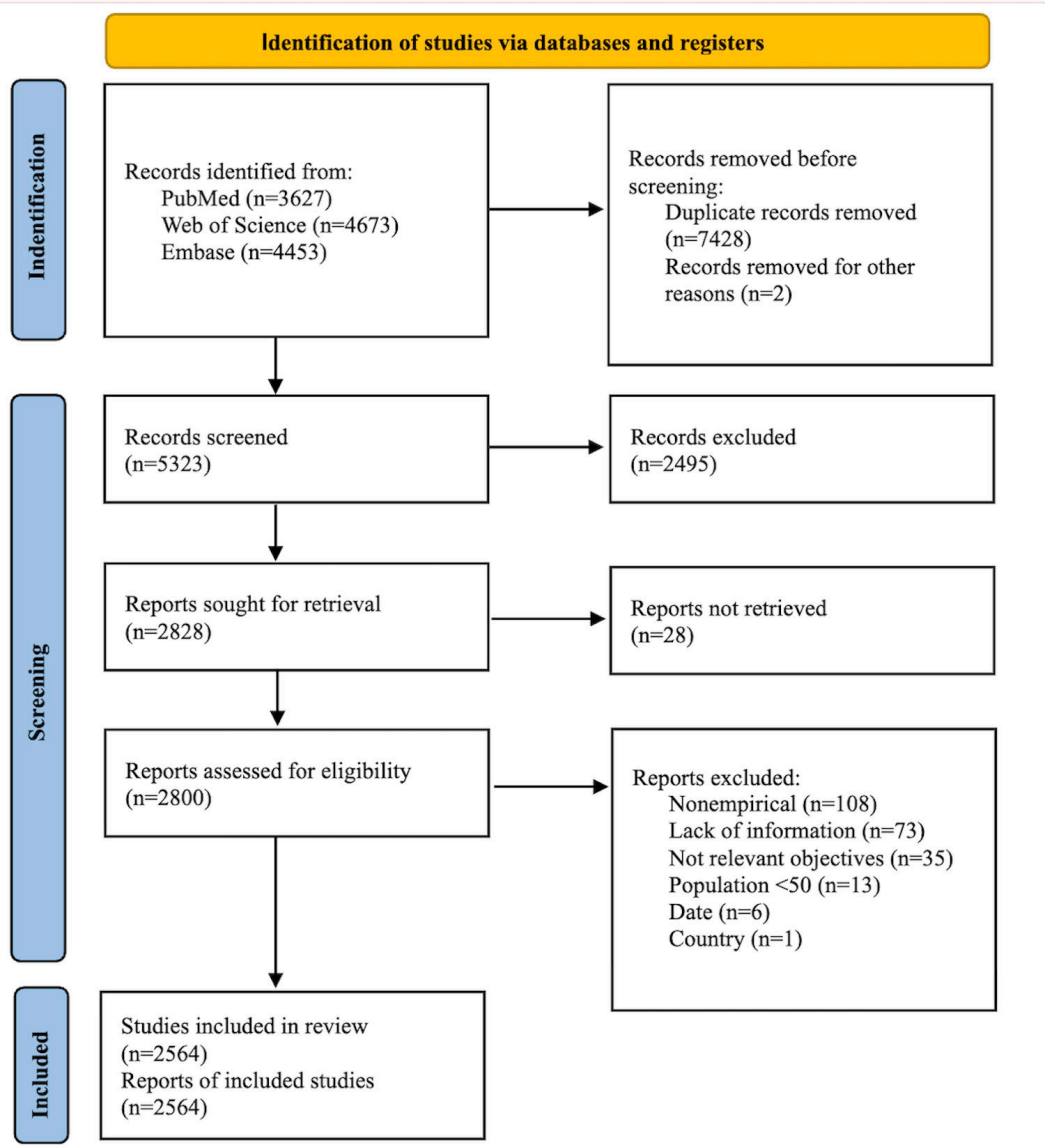
PRISMA (Preferred Reporting Items for Systematic Reviews and Meta-Analyses) flow diagram of the reports’ selection process for a scoping review of data sources used for influenza, respiratory syncytial virus, and SARS-CoV-2 surveillance and research across 12 European countries, 2005‐2025.

The literature search identified 12,753 records from MEDLINE, Web of Science, and Embase (Figure 1). After removing 7428 duplicates and excluding 2 additional retracted records, 5323 records underwent a screening of their title and abstract, and 2800 articles were reviewed for full-text assessment (n=28 reports could not be retrieved). Following this step, 236 reports were excluded. In total, 2564 reports met the inclusion criteria and form the basis of this review.

### Characteristics of Sources of Evidence

**Figure 2.**
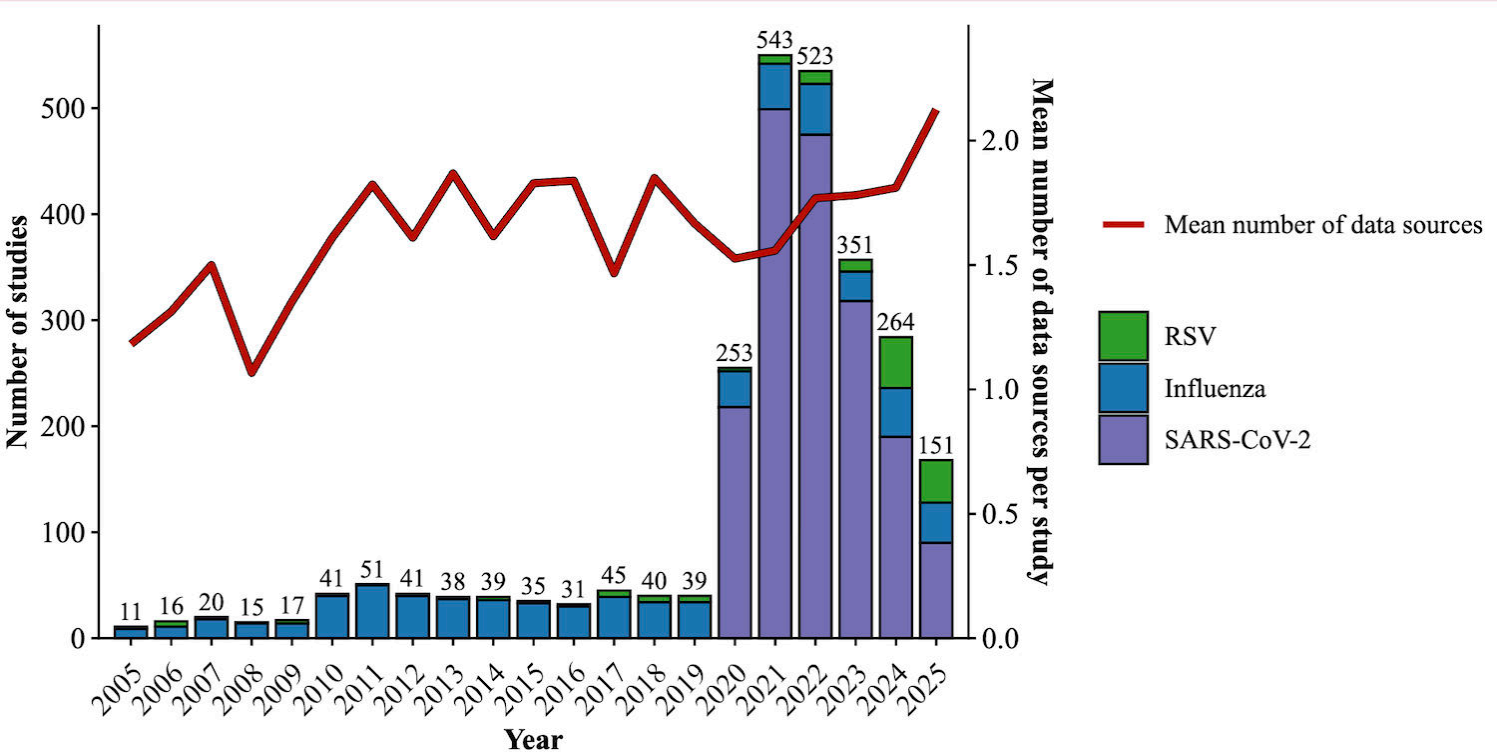
Temporal distribution of reports on influenza, respiratory syncytial virus (RSV), and SARS-CoV-2 surveillance and research across 12 European countries, 2005‐2025.

From 2005 to 2009, the number of reports remained low and relatively stable (Figure 2). The countries with the highest numbers of reports were Italy, Spain, and England (Figure S1 in Multimedia Appendix 1). The 2009 H1N1 influenza pandemic marked a turning point, with 17 reports published in 2009, followed by 41 in 2010 (+141%). Over the following decade (2010‐2019), annual report counts remained consistently higher than in the pre-2009 period, averaging 40 (range 31‐51) reports per year.

A second, much larger increase was observed with the onset of the COVID-19 pandemic: reports rose from 39 in 2019 to 253 in 2020 (+549%) and peaked at 543 in 2021. Report counts decreased progressively after 2021 but remained well above pre-2020 levels.

With respect to virus-specific focus, reports published before 2020 focused predominantly on influenza, while RSV-related reports were a minority. From 2020 onward, SARS-CoV-2 accounted for nearly 80% of all included reports each year. At the beginning of 2024, the number of RSV-focused reports increased, reaching levels higher than those for influenza over the 2024‐2025 period, with 88 RSV reports compared to 84 influenza reports (Figure 2).

The mean number of sources per report showed an overall increasing trend over time. This increase was more pronounced during 2 distinct periods: between 2009 and 2012, and from 2020 onward. In 2025, this value exceeded 2 sources per report for the first time (Figure 2).

### Synthesis of Results

#### Temporal Evolution of Data Source Types

**Figure 3.**
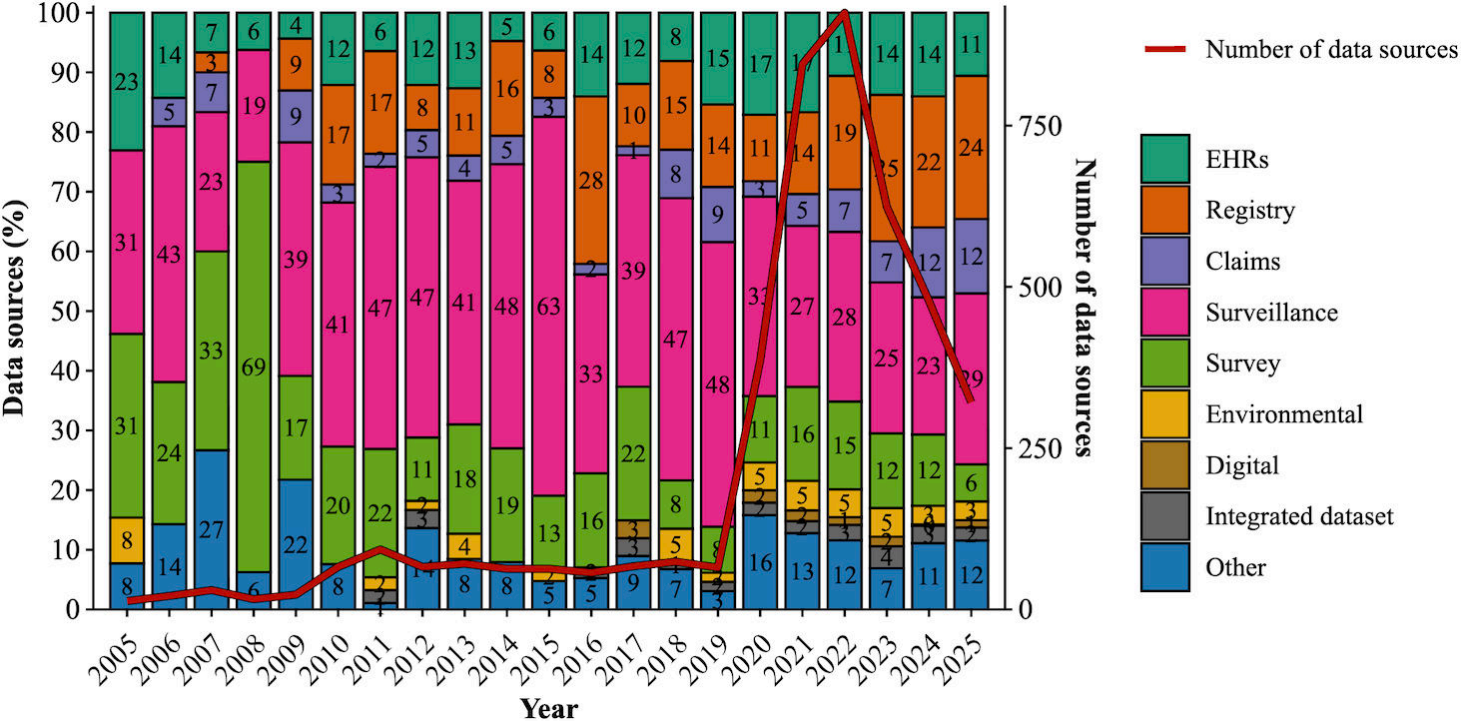
Temporal proportional distribution of data sources for influenza, respiratory syncytial virus, and SARS-CoV-2 surveillance and research across 12 European countries, 2005‐2025. EHRs: electronic health records.

Surveillance data, survey data, and EHRs were used throughout all years and accounted for the largest proportions of data sources overall (Figure 3 and Figure S2 in Multimedia Appendix 1). Surveillance data represented 31% of all data sources during 2005‐2009, increased substantially to 46% in 2010‐2019, then declined to 27% in 2020‐2025, while remaining the most frequently used type throughout. Survey data contributed 33% of data sources in 2005‐2009, decreasing to 16% in 2010‐2019 and further to 13% in 2020‐2025. EHRs remained relatively stable at around 10% in 2005‐2009 and 2010‐2019, increasing to 14% in 2020‐2025.

Registry and claims data were not present in all years but showed growing contributions when cumulative proportions were considered. Registry data accounted for 3% of data sources in 2005‐2009, increasing to 14% in 2010‐2019, and 19% in 2020‐2025. Claims data represented 5% of data sources in 2005‐2009, remained relatively stable at 4% in 2010‐2019, and rose to 7% in 2020‐2025.

Environmental data, integrated datasets, and digital data contributed to smaller proportions overall and showed later or more limited presence. Environmental data accounted for 1% in 2005‐2009, 2% in 2010‐2019, and increased to 4% in 2020‐2025. Integrated datasets were absent before 2010, rising to 1% in 2010‐2019 and 3% thereafter. Digital data were first recorded in 2010‐2019 (0.3%), increasing modestly to 1% in 2020‐2025.

The proportional contribution of open-access data sources also expanded. These accounted for 16% of all data sources in 2005‐2019, increasing to 25% in 2020‐2025. While proportions peaked during the early pandemic, they remained above pre-2020 levels thereafter (Figures S3 and S4 in Multimedia Appendix 1).

The proportion of reports using linked data sources rose from 8% during 2005‐2019 to 18% from 2020 onward (Figures S5 and S6 in Multimedia Appendix 1).

Additional data source distributions by virus and country are available in Multimedia Appendix 1 (Figures S7 and S8).

These temporal patterns showed a structured pre-COVID-19 vs post-COVID-19 pandemic shift in data source utilization. Before 2020, the data landscape was dominated by surveillance systems (46% of sources during 2010‐2019) and surveys (16%), with limited contributions from digital, environmental, or integrated sources. From 2020 onward, while surveillance data remained the most frequently used type (27%), its relative dominance declined substantially, and the landscape diversified: registries increased from 14% to 19%, EHRs from 10% to 14%, claims data from 4% to 7%, and environmental data from 2% to 4%. The use of open-access data sources expanded from 16% before 2020 to 25% afterward, and the proportion of reports using linked data sources more than doubled, from 8% to 18%. This diversification was accompanied by an increase in the mean number of data sources per report.

#### Distribution of Data Sources Across Report Objectives

**Figure 4.**
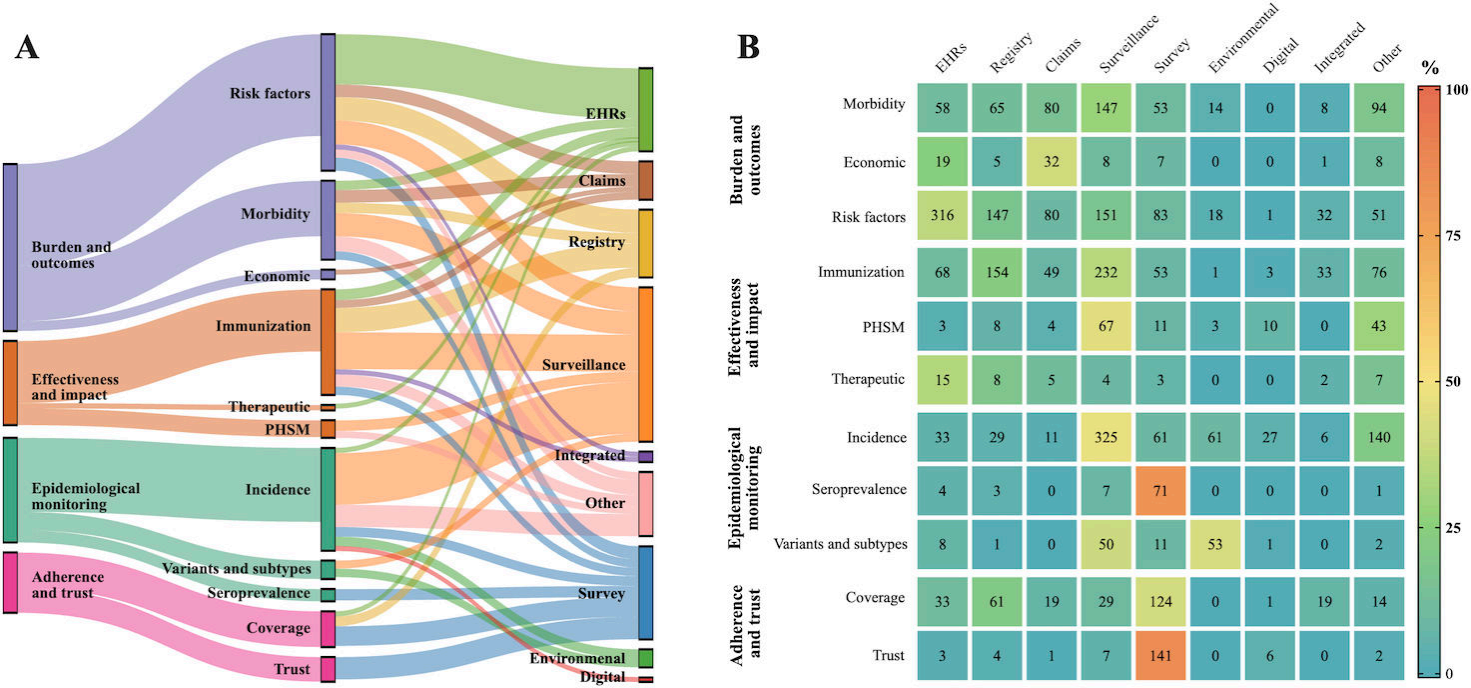
Distribution of data source types across research subobjectives on influenza, respiratory syncytial virus, and SARS-CoV-2 surveillance and research across 12 European countries, 2005‐2025. Panel A: Sankey diagram showing flows from primary research objectives (left) to subobjectives (center) to data source categories (right). Flows from primary objectives to subobjectives indicate the number of reports; flows from subobjectives to data sources indicate the total number of data sources. For readability, only flows ≥30 are shown; when none reach this level for a given node, only the largest available flow is displayed. Panel B: Evidence gap map presenting the number of data sources used for each combination of research subobjective (rows) and data source category (columns). Color intensity reflects the frequency of use. EHRs: electronic health records; PHSM: public health and social measures.

For epidemiological monitoring, incidence analyses relied mainly on surveillance data, with the use of additional data sources (Figure 4). Seroprevalence reports were mostly based on surveys, while analyses of variants and subtypes drew mainly on surveillance and environmental data.

Within the objective of evaluating effectiveness and impact, immunization reports used surveillance data and registries as primary sources, with moderate contributions from EHRs, surveys, and claims data. Public health and social measures analyses were also centered on surveillance data, while therapeutic effectiveness reports depended mainly on EHRs (Figure 4).

For the assessment of disease burden and health outcomes, reports on morbidity drew on a wide range of data sources, with an important role for surveillance systems followed by EHRs, registries, claims data, and surveys. Economic burden analyses relied mostly on claims data, while risk-factor analyses were mainly based on EHRs (Figure 4).

Reports focused on adherence and trust toward public health measures relied mostly on survey data, with limited use of other sources.

#### National Differences in Data Utilization

**Figure 5.**
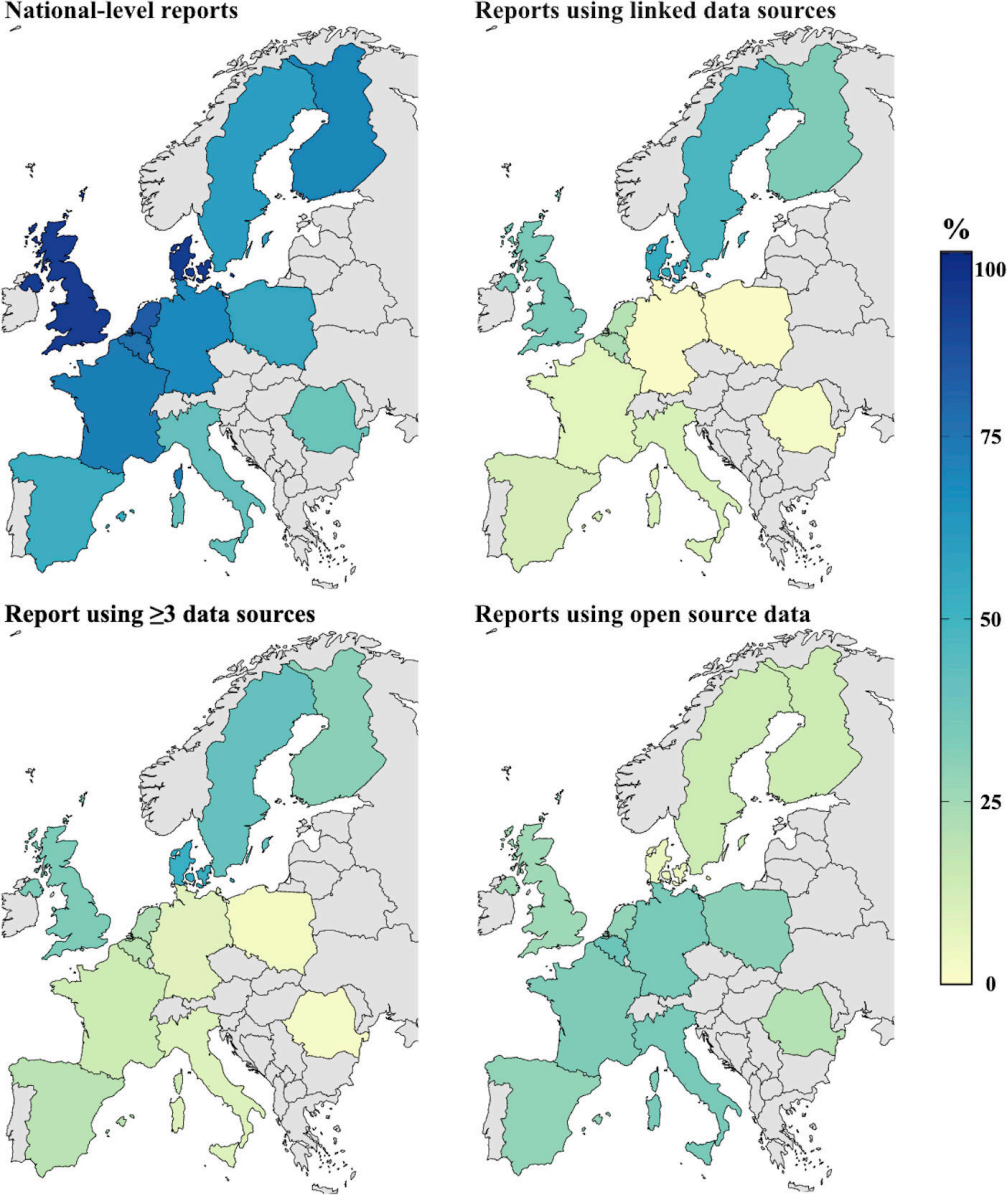
Cross-country variations in report level and data utilization for influenza, respiratory syncytial virus, and SARS-CoV-2 surveillance and research across 12 European countries, 2005‐2025.

Reports conducted at a supraregional level were most common in northern countries, mainly in England and Denmark (94% each), followed by the Netherlands (84%), Belgium (76%), France (73%), Finland (70%), Germany (69%), Sweden (59%), and Poland (56%), while lower proportions were observed in Spain (53%), Italy (41%), and Romania (39%) (Figure 5).

A similar gradient was evident for the use of linked data sources, with the highest proportions observed in Denmark (54%), Sweden (48%), England (35%), and Finland (33%). Intermediate levels were seen in Belgium (23%) and the Netherlands (20%), and lower levels were recorded in Spain (12%), Italy (11%), and France (8%). Minimal use of linkage was recorded in Romania (2%) and in both Poland and Germany (1% each).

The same geographical pattern was observed for the use of ≥3 data sources per report. Multisource reports were most common in Denmark (52%), Sweden (41%), England (34%), and Finland (30%). Intermediate proportions were found in the Netherlands (22%), Belgium and Spain (19% each), and France (14%), while proportions were below 10% in Italy (9%), Germany (9%), Poland (3%), and Romania (2%).

The use of open-access sources showed an opposite pattern, being more frequent in Southern Europe: Belgium (38%), Italy and Germany (35% each), France (33%), Poland (30%), the Netherlands (29%), and Spain (28%). England reported 26%, and Romania 20%, whereas lower proportions were seen in the Nordic countries, with Sweden and Finland at 14% and Denmark at 5%.

## Discussion

### Summary of Evidence

This scoping review characterized the data sources underpinning surveillance and research on influenza, RSV, and SARS-CoV-2 across 12 European countries over the past 2 decades. Four principal findings emerged from the analysis of 2564 reports. First, the COVID-19 pandemic was associated with a marked diversification in data source usage, with growing contributions from EHRs, registries, digital and environmental data, alongside traditional surveillance systems. Second, data sources showed clear functional alignment with research objectives, yet cross-sectoral integration remained rare. Third, pronounced geographic disparities revealed a gradient in data integration capacity, with Northern European countries more frequently using linked and multisource datasets compared with Southern and Eastern Europe. Fourth, many crisis-driven innovations showed limited sustainability beyond the acute pandemic period. By examining temporal trends, thematic uses, and cross-country differences, we aimed to clarify not only which data are being generated and used but also what this reveals about the broader capacity of European systems to detect, monitor, and study respiratory viruses.

### A Landscape Shaped by Public Health Crises

The temporal trajectory of respiratory virus research in Europe appears to align with major public health crises over the past 20 years. Between 2005 and 2009, activity remained modest and stable, which may indicate limited incentives or opportunities for structural change in data practices. The 2009 H1N1 influenza pandemic was associated with the first noticeable increase in report volume. However, this expansion did not translate into substantial diversification of data sources: surveillance data continued to dominate, while environmental and digital data appeared only sporadically. The 2009 H1N1 influenza pandemic can be interpreted as an early test of existing data infrastructures, but it did not substantially alter their overall architecture or breadth in most countries examined.

The COVID-19 pandemic was associated with far more substantial transformation. Report numbers increased more than fivefold between 2019 and 2020, peaking in 2021, representing the highest volume observed and aligning with a significant shift in data needs and practices. From early 2020, governments and research institutions responded by generating, releasing, and harmonizing data at extraordinary speed [18]. National dashboards emerged as major public health tools, providing daily updates on the number of tests, cases, hospitalizations, and vaccination coverage [26]. This rapid transition from closed repositories to open-access, publicly accessible platforms was associated with a broader reconfiguration of data governance [27]. During this period, the proportional use of open-access datasets increased, reaching around one-quarter of all sources, which may reflect evolving practices related to transparency and accountability [28,29].

At the same time, data types that had previously been marginal or absent were increasingly integrated into research. Digital mobility data from mobile devices were used to inform real-time modeling of transmission dynamics [30]. Moreover, social media platforms were leveraged for sentiment analysis, aiming to capture attitudes toward health measures and levels of trust in institutions [31]. Large-scale behavioral and serological surveys were deployed across multiple countries [32,33]. Environmental datasets, including meteorological and air-quality indicators, gained prominence in exploring potential ecological factors associated with SARS-CoV-2 circulation [34,35]. Wastewater surveillance was implemented at scale for the first time and, in several settings, showed potential for early signal capacity and complementing clinical testing systems [36].

Despite this rapid diversification, most of these emerging sources remained concentrated on SARS-CoV-2, suggesting that innovations introduced during crises may remain pathogen-specific and may not necessarily translate into broader, system-wide strengthening [37].

Finally, many innovations catalyzed by the COVID-19 pandemic showed signs of limited continuity as the pandemic waned: several national dashboards reduced their update frequency or were decommissioned [38]; wastewater programs were scaled back in multiple countries [39]; and genomic sequencing programs contracted as funding streams receded [40]. A recent EU-wide mapping survey confirmed that funding remains the primary challenge for sustaining wastewater surveillance systems, with more than 70% of participating countries lacking a legal framework governing their operation [41]. These observations suggest that crisis-driven advances, although effective under acute pressure, may face sustainability challenges in the absence of dedicated long-term funding mechanisms and institutional anchoring.

### Lack of Cross-Sectoral Data Integration

The distribution of data sources across research objectives suggests functional alignment. Reports addressing individual-level outcomes such as risk factors or effectiveness predominantly relied on data sources providing detailed patient-level information, such as EHRs, claims, and disease registries, that allow for longitudinal follow-up or linkage across care episodes [42-44].

Conversely, reports exploring population-level questions such as incidence trends, real-time monitoring, and transmission dynamics more commonly use aggregated surveillance data, large-scale surveys, digital data streams, or publicly accessible statistics [45-48]. These sources typically provide rapid situational awareness, broader population coverage, and frequent updates, aligning naturally with objectives requiring real-time or near-real-time signals. The observed alignment between research questions and data types is consistent with methodological principles documented in the literature [49,50].

However, each of these commonly used data source types carries inherent limitations that may influence the scope and quality of evidence derived from them. Sentinel surveillance systems, while designed for timeliness and consistency, may lack representativeness if participating sites are not population-based and are generally unable to capture mild or asymptomatic infections [17]. EHRs, although rich in clinical detail, are subject to well-documented biases, including informed-presence bias, where sicker patients are overrepresented, labeling bias related to coding practices, and variability in data quality across institutions and countries [51]. Claims data offer broad population coverage and longitudinal tracking but are constrained by the use of administrative coding systems that may not accurately reflect clinical reality and typically lack detailed clinical variables such as laboratory results or symptom severity [52]. Survey data, while valuable for capturing behaviors and attitudes, are susceptible to recall bias, social desirability bias, and low response rates, particularly during crisis periods [53].

Integration across data domains remained rare. Only a small proportion of reports combined clinical, behavioral, environmental, and digital information [54,55]. This finding is notable given the growing recognition that respiratory virus dynamics may be influenced by multilevel interactions: clinical vulnerability intersects with behavioral responses, environmental conditions, mobility patterns, and sociodemographic characteristics [56,57]. The persistence of predominantly single-source designs, even in the postpandemic period, suggests that structural and institutional barriers may continue to limit integration in several European research settings [58].

These patterns may reflect broader forms of data fragmentation in some European countries. Clinical data are typically managed by hospitals, regional health authorities, or national insurance systems [59-61]; behavioral and mobility data are held by private technology companies or statistical agencies [62]; and environmental indicators are generally maintained by meteorological or environmental institutions [63]. Each of these domains operates under distinct ethical, legal, and operational frameworks, which can complicate cross-sector data integration [64]. In practice, research teams often rely on the sources to which they already have access, which may limit the assembly of more comprehensive, multidimensional datasets. This institutional fragmentation may pose challenges in transitioning from pathogen-specific surveillance to more integrated forms of respiratory health intelligence.

Strategically, these findings point to the potential value of interoperable, cross-domain infrastructures capable of supporting multisource analyses as a routine standard rather than an exception.

### Geographical Inequalities in Data Capacity

Pronounced geographical disparities in data integration and source utilization suggest that Europe may operate as a set of distinct data environments rather than a fully coherent surveillance ecosystem [65]. Countries such as Denmark, Sweden, Finland, and England appear to have relatively strong capacity for national-level analyses, frequent use of linked datasets, and regular mobilization of multiple data sources. In contrast, Italy, Spain, Poland, and Romania exhibited more limited integration and linkage, and a greater reliance on single-source approaches. The pattern reverses for open-access data, which appears to be used more often in southern European countries than in the Nordic context. This pattern warrants cautious interpretation: greater use of open datasets in Italy and Spain does not necessarily indicate that these countries produce more open data [66,67]. One possible explanation could be that researchers compensate for the limited accessibility or integration of internal infrastructures by relying more on publicly available sources. Conversely, in countries with stronger internal infrastructures, standardized registries and linked datasets may already meet many analytical requirements, reducing reliance on open-access sources. This suggests that the use of open data reflects strategies of access rather than necessarily indicating the underlying abundance or maturity of data systems.

This geographical gradient appears to reflect factors beyond purely technical differences. It may also be influenced by broader political and institutional patterns shaping data production in Europe. Many northern European countries have long invested in integrated digital infrastructures, unique personal identifiers, and harmonized national registries [68]. These investments have contributed to environments where data appear to flow more easily across institutions, allowing researchers to draw on clinical, administrative, and laboratory information within a more unified framework.

The situation differs in countries with more decentralized health governance. Italy and Spain illustrate how regional autonomy may support high-quality datasets at the local level while making it more difficult to assemble national datasets. Variation in regional standards, institutional fragmentation, and the absence of coordinating mechanisms may restrict the development of integrated national evidence [69,70]. Consequently, even when rich data exist locally, they may not translate into unified national-level analyses [71,72].

Centralization, however, does not automatically guarantee effective coordination. France and Germany, despite strong national institutions, appear to face persistent challenges associated with divided responsibilities between agencies, complex administrative procedures, and rigid regulatory frameworks [73,74]. Multiple national databases exist in both systems, but many are often governed by different authorities and may operate on incompatible standards [75,76]. In Germany, data governance is shared across federal and corporatist institutions, including statutory health insurance funds and *Länder* authorities, which may contribute to limited interoperability and slow linkage processes despite high data availability [77].

The disparities identified across countries suggest that stronger European-level infrastructures may be beneficial to reduce fragmentation and support more coordinated surveillance. The European Health Data Space (EHDS), officially published in the Official Journal of the European Union in March 2025, represents a landmark regulatory development aimed at facilitating both primary and secondary use of electronic health data across member states [78,79]. Alongside EHDS, initiatives such as ELIXIR (European Life-science Infrastructure for Biological Information), EHDEN (European Health Data and Evidence Network), and the EU-WISH (EU-Wastewater Integrated Surveillance for Public Health) Joint Action for wastewater surveillance aim to strengthen specific aspects of health data infrastructure [41,79,80]. However, a recent analysis of the EHDS implementation landscape has highlighted persistent challenges related to governance complexity, varying institutional readiness across countries, and the need for sustained investment in national health data access bodies [81]. Progress remains largely project driven, and many initiatives lack the long-term institutional anchoring necessary for structural impact. Coordination across sectors and countries is limited, and synthesizing these efforts into a coherent, long-term strategy will be essential to build a truly integrated European data ecosystem.

### Leveraging AI for Data Integration Without Architectural Coherence

The diversification of data sources documented in this review suggests substantial changes in the architecture of respiratory virus surveillance. The COVID-19 pandemic was associated with the adoption of new data types contributing to a data landscape that appears more complex and voluminous than in earlier periods.

Such environments may benefit from analytical approaches that integrate diverse signals varying in structure, frequency, quality, and spatial resolution. AI has emerged as a potentially useful tool for managing this complexity at scale. Recent reports have highlighted the potential of machine learning models to integrate both traditional and nontraditional data for epidemic forecasting and response [82,83]. A distinctive feature of this emerging landscape is the growing reliance on aggregated data that are routinely released in open access. Many of the new data streams mobilized during and after the pandemic, whether published by national authorities, statistical agencies, or international platforms, are aggregated and routinely updated. Their lack of individual-level information generally subjects them to fewer legal and ethical restrictions, which may facilitate cross-border circulation. This capacity to harness standardized and publicly available data may enable AI approaches to support more comparable cross-country analyses and integrate heterogeneous signals within a shared analytical framework. The Franco-German AIOLOS program illustrates recent efforts to develop sustainable platforms using AI to combine diverse data streams for timely outbreak detection [84].

Fully leveraging AI in public health will depend on sustained investment in data that are transparent and accessible, well curated, and capable of capturing the complexity of respiratory virus transmission.

### Limitations

This review offers a 20-year perspective covering 2 major pandemics and their impact on respiratory virus data systems by including 12 European countries with diverse health care architectures, regulatory frameworks, and digital capacities. The breadth of the review enables documentation of the transition from the pre-COVID-19 pandemic surveillance models reliant on institutional datasets to the more complex and heterogeneous data landscape that followed. Through systematic mapping of the data sources used in over 2500 reports, the review generates a comprehensive overview of temporal trends and cross-country variations. Finally, this review used an objective-driven analytical framework. Unlike previous reviews that primarily classify reports by data source, our approach centers on the research and public health objectives underlying the use of data in respiratory virus research, offering a more practice-oriented and policy-relevant perspective [85,86].

Nonetheless, some limitations must be acknowledged. First, each report was assigned to a single primary objective, even when the underlying study addressed several research questions. For instance, vaccine coverage studies that also included effectiveness analyses, or burden-of-disease studies that informed intervention recommendations, were categorized solely according to their principal stated objective. Secondary objectives were therefore not captured in the synthesis. This choice was made deliberately to ensure a parsimonious and mutually exclusive categorization, allowing clear cross-report comparison and avoiding the double counting that a multilabel scheme would have introduced. As a consequence, our results should be interpreted as reflecting the dominant research orientation of each report rather than the full breadth of questions it may have addressed. Second, the level of detail reported on data sources varied substantially across reports. Some reports provided extensive descriptions of the datasets used, including information on coverage, linkage mechanisms, and update frequency, whereas others offered only minimal information. Third, identifying data linkage proved challenging. Many reports indicated whether linkage occurred but did not specify which sources were linked or how. Inconsistent terminology (eg, “integration,” “linkage,” “combination”) added to the ambiguity. Fourth, in line with established scoping review methodology, no formal quality assessment or risk-of-bias appraisal of included reports was performed [24,87]. This means that the review documents which data sources are being used for respiratory virus research but does not assess how well individual sources perform or how reliably they measure the outcomes of interest. Finally, like all literature-based reviews, our analysis is constrained by what is publicly reported, and data generated primarily for surveillance purposes are not systematically published. These gaps reflect broader issues in transparency and documentation across Europe and reinforce the need for more consistent reporting standards when using complex health data infrastructures.

### Conclusions

This scoping review provides a multipathogen, cross-country mapping of data sources used for respiratory virus surveillance and research in Europe over 2 decades, using an innovative objective-driven analytical framework. Unlike prior reviews focused on single pathogens, individual data types, or specific surveillance systems, this review offers a consolidated and comparative perspective that transcends single viruses, data categories, research objectives, and national settings. By documenting temporal trends, thematic patterns, and geographic disparities in data source utilization across 2564 reports, it generates an evidence base that can directly inform strategic decision-making in public health. The findings show that the COVID-19 pandemic was associated with major innovation in data generation, access, and analytical methods, but also revealed that much of this progress remained centered on SARS-CoV-2 and lacked long-term coordination, with structural barriers continuing to limit the production of timely, integrated, and decision-relevant data across Europe. To address these challenges, several concrete actions are needed: investment in unique patient identifiers and harmonized coding standards to facilitate cross-source data linkage; development of federated analysis platforms that enable cross-border analyses while respecting national data governance requirements; establishment of sustainable funding mechanisms for surveillance innovations, such as wastewater surveillance and genomic sequencing, beyond crisis-driven budgets; and institutional reforms to facilitate cross-sectoral data-sharing agreements between health, environmental, and social data custodians. The EHDS regulatory framework provides a foundation for standardizing data access procedures across EU member states, but its success will depend on strong national engagement, cross-sector collaboration, and a shared recognition that health data ultimately derive from individuals and should be used to protect and improve their health.

## Supplementary material

10.2196/92917 Multimedia Appendix 1 Supplementary tables and figures.

10.2196/92917 Checklist 1 PRISMA-ScR checklist.

10.2196/92917 Checklist 2 PRISMA-S checklist.

## Abbreviations

EHDEN: European Health Data and Evidence Network
EHDS: European Health Data Space
EHRs: electronic health records
ELIXIR: European Life-science Infrastructure for Biological Information
EU-WISH: EU-Wastewater Integrated Surveillance for Public Health
PRISMA-S: Preferred Reporting Items for Systematic Reviews and Meta-Analyses literature search extension
PRISMA-ScR: Preferred Reporting Items for Systematic Reviews and Meta-Analyses extension for Scoping Reviews
RSV: respiratory syncytial virus
