# Supplementary material for: How Pandemics Have Reshaped the Respiratory Virus Data Landscape in Europe: Scoping Review
Source: J Med Internet Res. 2026 Jul 17;28:e92917. doi: 10.2196/92917 (PMC13378907; doi:10.2196/92917)
Supplement: Multimedia Appendix 1 [file jmir-v28-e92917-s001.pdf]

How pandemics have reshaped respiratory virus data landscape in Europe: Scoping review

Multimedia Appendix 1

Table of contents

Tables – Multimedia Appendix 1 2

Table S1. Search query. 2

Table S2. Operational definitions used to categorize the research objectives of included studies, with typical indicators, data, and study designs. 3

Table S3. Operational rules for assigning reports across overlapping objectives. 5

Table S4. Definitions and examples of data source categories. 6

Figures – Multimedia Appendix 1 7

Figure S1. Temporal count distribution of reports on influenza, RSV, and SARS-CoV-2 surveillance and research across 12 European countries, 2005–2025. 7

Figure S2. Temporal count distribution of data source types used on influenza, RSV, and SARS-CoV-2 surveillance and research across 12 European countries, 2005–2025 7

Figure S3. Number of open-access data sources used per year on influenza, RSV, and SARS-CoV-2 surveillance and research across 12 European countries, 2005–2025 8

Figure S4. Proportion of open-access data sources used per year on influenza, RSV, and SARS-CoV-2 surveillance and research across 12 European countries, 2005–2025 8

Figure S5. Number of reports with linkage between at least two data sources per year on influenza, RSV, and SARS-CoV-2 surveillance and research across 12 European countries, 2005–2025. 9

Figure S6. Proportion of reports with linkage between at least two data sources per year on influenza, RSV, and SARS-CoV-2 surveillance and research across 12 European countries, 2005–2025. 9

Figure S7. Proportion of data source types used on influenza, RSV, and SARS-CoV-2 surveillance and research, by virus, across 12 European countries, 2005–2025. 10

Figure S8. Proportion of data source types used on influenza, RSV, and SARS-CoV-2 surveillance and research, by country, across 12 European countries, 2005–2025. 10

References 11

Tables – Multimedia Appendix 1

Table S1. Search query.

**Date of last search: September 3, 2025**
**PubMed**: (Surveillance[ti] OR Track*[ti] OR Monitor*[ti] OR Detection[ti] OR Dynamic[ti] OR Distribution[ti] OR Spread[ti] OR Prevalence[ti] OR Incidence[ti] OR Impact*[ti] OR Effect*[ti] OR Burden[ti] OR Mortality[ti] OR Morbidity[ti] OR Hospital*[ti] OR Cost*[ti] OR Economic[ti] OR "Risk Factors"[ti] OR High-risk[ti] OR Characteristics[ti] OR "Clinical Outcomes"[ti] OR Coverage[ti] OR Uptake[ti] OR Determinants[ti] OR Acceptance[ti] OR Adherence[ti] OR Hesitancy[ti] OR Sentiment[ti]) AND (COVID-19[ti] OR COVID[ti] OR SARS-CoV-2[ti] OR Influenza[ti] OR Flu[ti] OR "Respiratory Syncytial Virus"[ti] OR RSV[ti]) AND (France[ti] OR England[ti] OR Germany[ti] OR Sweden[ti] OR Netherlands[ti] OR Belgium[ti] OR Spain[ti] OR Italy[ti] OR Poland[ti] OR Denmark[ti] OR Finland[ti] OR Romania[ti]) NOT ("COVID-19 Pandemic"[ti] OR "COVID-19 epidemic"[ti] OR "Mental Health"[ti] OR Psych*[ti] OR Depression[ti] OR Anxiety[ti] OR Stress[ti]) NOT (Comment[ti] OR Comment[pt] OR Letter[ti] OR Letter[pt] OR Review[ti] OR Review[pt] OR Trial[ti] OR "Clinical Trial"[pt] OR Correction[tiab] OR Erratum[ti] OR "Published Erratum"[pt])
**WoS**: (TI=Surveillance OR TI=Track* OR TI=Monitor* OR TI=Detection OR TI=Dynamic OR TI=Distribution OR TI=Spread OR TI=Prevalence OR TI=Incidence OR TI=Impact* OR TI=Effect* OR TI=Burden OR TI=Mortality OR TI=Morbidity OR TI=Hospital* OR TI=Cost* OR TI=Economic OR TI="Risk Factors" OR TI=High-risk OR TI=Characteristics OR TI="Clinical Outcomes" OR TI=Coverage OR TI=Uptake OR TI=Determinants OR TI=Acceptance OR TI=Adherence OR TI=Hesitancy OR TI=Sentiment) AND (TI=COVID-19 OR TI=COVID OR TI=SARS-CoV-2 OR TI=Influenza OR TI=Flu OR TI="Respiratory Syncytial Virus" OR TI=RSV) AND (TI=France OR TI=England OR TI=Germany OR TI=Sweden OR TI=Netherlands OR TI=Belgium OR TI=Spain OR TI=Italy OR TI=Poland OR TI=Denmark OR TI=Finland OR TI=Romania) NOT (TI=("COVID-19 Pandemic") OR TI=("COVID-19 epidemic") OR TI="Mental Health" OR TI=Psych* OR TI=Depression OR TI=Anxiety OR TI=Stress) NOT (TI=Comment OR ALL=Comment OR TI=Letter OR ALL=Letter OR TI=Review OR ALL=Review OR TI=Trial OR ALL="Clinical Trial" OR (TI=Correction OR AB=Correction) OR TI=Erratum OR ALL="Published Erratum")
**Embase**: (Surveillance:ti OR Track*:ti OR Monitor*:ti OR Detection:ti OR Dynamic:ti OR Distribution:ti OR Spread:ti OR Prevalence:ti OR Incidence:ti OR Impact*:ti OR Effect*:ti OR Burden:ti OR Mortality:ti OR Morbidity:ti OR Hospital*:ti OR Cost*:ti OR Economic:ti OR 'Risk Factors':ti OR High-risk:ti OR Characteristics:ti OR 'Clinical Outcomes':ti OR Coverage:ti OR Uptake:ti OR Determinants:ti OR Acceptance:ti OR Adherence:ti OR Hesitancy:ti OR Sentiment:ti) AND (COVID-19:ti OR COVID:ti OR SARS-CoV-2:ti OR Influenza:ti OR Flu:ti OR 'Respiratory Syncytial Virus':ti OR RSV:ti) AND (France:ti OR England:ti OR Germany:ti OR Sweden:ti OR Netherlands:ti OR Belgium:ti OR Spain:ti OR Italy:ti OR Poland:ti OR Denmark:ti OR Finland:ti OR Romania:ti) NOT ('COVID-19 Pandemic':ti OR 'COVID-19 epidemic':ti OR 'Mental Health':ti OR Psych*:ti OR Depression:ti OR Anxiety:ti OR Stress:ti) NOT (Comment:ti OR Letter:ti OR Review:ti OR Trial:ti OR Correction:ti,ab OR Erratum:ti)

Table S2. Operational definitions used to categorize the research objectives of included studies, with typical indicators, data, and study designs.

Research objective	Sub-objective	Operational definition	Typical indicators, data and study designs	n
**Epidemiological monitoring** (n = 659)				
**Epidemiological monitoring**	**Incidence**	Describe the occurrence or circulation of respiratory viruses in a population (how many cases, where and when), without primarily estimating the effect of an intervention or the determinants of an outcome.	Case counts, incidence/positivity rates, attack rates, syndromic and laboratory surveillance, time trends and geographic spread.	466
	**Variants & subtypes**	Identify, characterize or track circulating viral types, subtypes, lineages or genetic variants.	Genomic / genetic sequencing, variant or subtype typing, lineage surveillance, mutation profiling.	116
	**Seroprevalence**	Estimate the proportion of a population with detectable antibodies as a marker of cumulative exposure or population immunity.	Cross-sectional or repeated serosurveys; seroprevalence; antibody titers at population level.	77
**Effectiveness & impact** (n = 526)				
**Effectiveness & impact**	**Immunization**	Estimate the effectiveness, cost-effectiveness, or impact of immunization.	Vaccine effectiveness estimates, test-negative or cohort designs, impact modeling of immunization, cost-effectiveness of vaccination.	387
	**PHSM**	Estimate the effect or impact of non-pharmaceutical, public-health or social measures on viral transmission or outcomes.	Evaluations of distancing, masking, school/workplace closures, mobility restrictions; interrupted time-series, modeling of counterfactual scenarios.	101
	**Therapeutic**	Estimate the effectiveness, cost-effectiveness, or impact of pharmacological treatments.	Comparative effectiveness of antivirals / treatment regimens; treatment cost-effectiveness analyses.	34
**Burden & outcomes** (n = 1029)				
**Burden & outcomes**	**Risk factors**	Identify determinants, predictors or characteristics associated with infection or with a clinical outcome.	Associations between exposures/patient characteristics and infection or outcomes; adjusted odds/hazard/risk ratios; predictor identification.	641
	**Morbidity**	Describe the burden of disease in a population at a given point or period in terms of number of cases,	Point or period prevalence of cases, hospitalization rates, ICU admission rates,	327

Research objective	Sub-objective	Operational definition	Typical indicators, data and study designs	n
		hospitalizations, and deaths, without tracking temporal trends over time or estimating the effect of an intervention or determinant.	case-fatality and mortality rates; cross-sectional studies.	
	**Economic**	Quantify the healthcare resource use or economic cost attributable to disease	Healthcare resource utilization, direct/indirect costs, length of stay, cost-of-illness studies	38
**Population adherence and trust toward public health measures** (n = 350)				
**Population adherence & trust**	**Coverage**	Measure the uptake of a public-health measure, chiefly vaccination, as an indicator of population adherence.	Vaccination coverage, uptake treated as a behavioral outcome not as an evaluated intervention.	195
	**Public trust**	Assess attitudes, beliefs, knowledge, hesitancy, willingness or confidence toward vaccines or public-health measures.	Surveys of knowledge/attitudes/beliefs, hesitancy and acceptance, intention to vaccinate, confidence in measures.	125

Within each objective, sub-objective counts may not sum to the objective total because some reports were assigned to more than one sub-objective.

Table S3. Operational rules for assigning reports across overlapping objectives.

Overlapping categories	Discriminating questions	Resolution
**Incidence VS Morbidity**	Does the report monitor the occurrence of infections over time (surveillance, trends, temporal tracking), or does it describe disease burden at a given point or period without temporal follow-up?	Temporal surveillance → Incidence<br>Point-in-time or period burden without trend analysis → Morbidity
**Incidence VS Immunization**	Is the goal to describe viral circulation, or to estimate the protective effect of vaccination?	Descriptive circulation → Incidence<br>Effect estimate → Immunization
**Incidence VS PHSM**	Is the change in cases merely described over time, or is the analysis designed to estimate the effect of a non-pharmaceutical measure?	Descriptive trend → Incidence<br>Effect estimation → PHSM
**Seroprevalence VS Coverage**	Does the report estimate the proportion with antibodies as a population marker, or document vaccine uptake as an adherence indicator?	Antibody prevalence → Seroprevalence<br>Recorded uptake → Coverage
**Coverage VS Immunization**	Is vaccination treated as a behavioral outcome (who is vaccinated and why), or as the intervention whose effect/impact is evaluated?	Uptake as outcome → Coverage<br>Vaccination as an intervention → Immunization
**Economic VS Effectiveness and impact**	Does the analysis estimate the cost or resource use attributable to disease, or the cost-effectiveness of a specific intervention?	Cost of illness → Economic burden<br>Cost-effectiveness of an intervention → Therapeutic/Immunization
**Risk factors VS Therapeutic**	Are clinical outcomes described in a general/untreated population, or compared between treatment groups to assess a therapeutic effect?	Outcomes described → Risk factors<br>Treatment-effect comparison → Therapeutic

Table S4. Definitions and examples of data source categories.

Data source	Definition	Examples
**Electronic health records**	Electronic version of a patient's medical information and history.	—
**Registry**	A structured collection of standardized data about individuals, conditions, or events, maintained for monitoring, research, or quality improvement.	Vaccination registries; birth/mortality registries; disease-specific registries (e.g., cancer)
**Claims database**	An administrative dataset built from billing records submitted by healthcare providers to payers, capturing services delivered, their costs, and patterns of healthcare use. Can also be used to estimate service demand and hospital resource needs.	Hospital stays claims; emergency department claims; pharmacy billing claims
**Surveillance data**	Information generated by identifiable surveillance systems that monitor respiratory viruses through structured, often institutionalized processes designed to track infections over time and across locations.	Sentinel GP networks; laboratory-confirmed virus reporting; hospital-based surveillance
**Survey**	Data collected through structured investigations designed to measure characteristics, behaviors, or health status in a population, via questionnaires, interviews, or field-based biological sampling.	Questionnaires; interviews; serological surveys
**Environmental data**	Data originating from the physical, chemical, or biological environment that can inform the detection, transmission, or risk factors of health events.	Wastewater surveillance; climate/weather variables; air-pollution measurements; environmental surface sampling
**Digital data**	Information generated by digital platforms or technologies that capture human behavior, interactions, or activities relevant for monitoring population trends or health-related patterns.	Smartphone mobility data; social-media activity; mobile apps or wearables
**Integrated dataset**	A data environment where multiple sources are brought together and linked within the same system, allowing cross-source connections, harmonization, and combined analyses.	Platform linking EHRs, laboratory results, and surveillance data via shared identifiers
**Other**	Data sources that do not fit the categories above, often including open-access socio-demographic or contextual information used to complement health analyses.	Population statistics; publicly available socioeconomic indicators

Open data: data sources accessible online or published online/in the literature, without specific access rights or a formal request. Data reusability and licensing conditions were not taken into account.

Figures – Multimedia Appendix 1

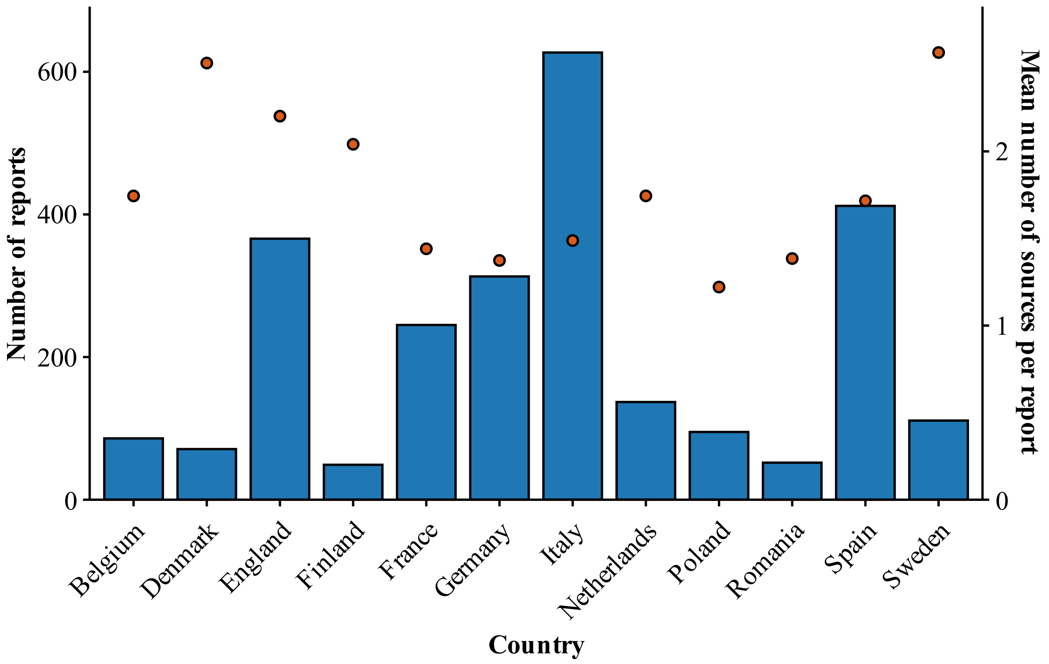

Figure S1. Temporal count distribution of reports on influenza, RSV, and SARS-CoV-2 surveillance and research across 12 European countries, 2005–2025.
Bars represent the total number of reports by country (left axis), while dots indicate the mean number of sources per report (right axis).

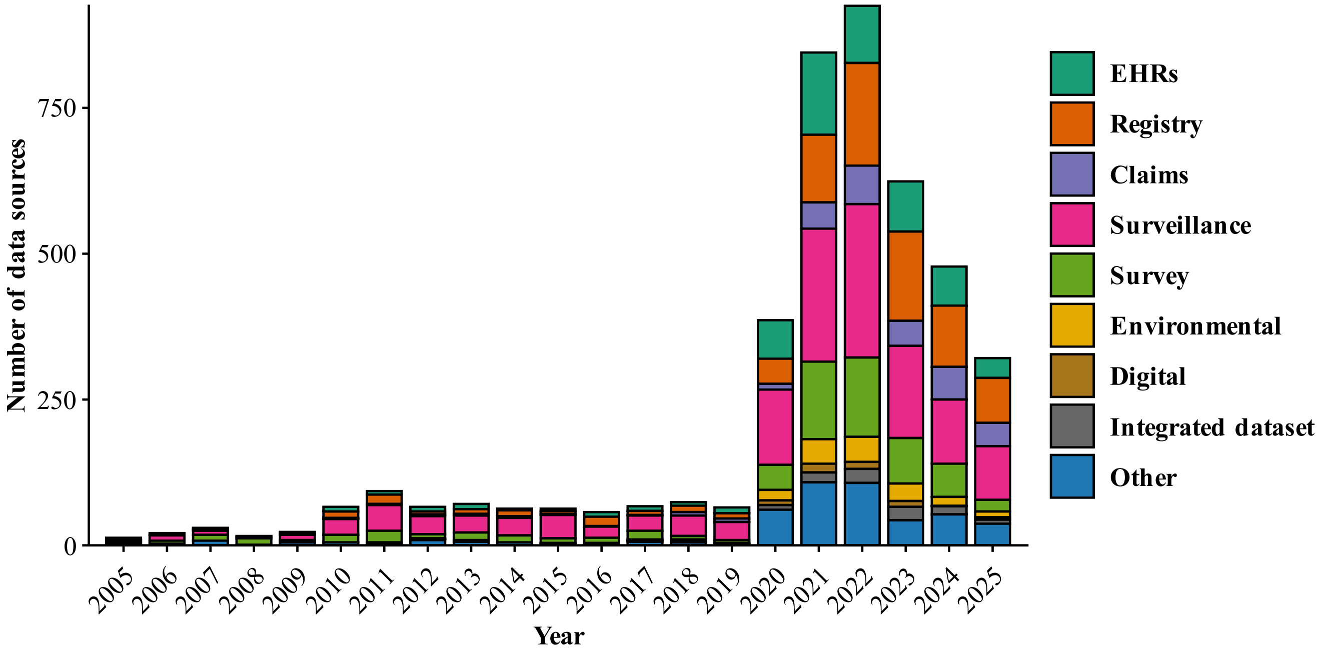

Figure S2. Temporal count distribution of data source types used on influenza, RSV, and SARS-CoV-2 surveillance and research across 12 European countries, 2005–2025.

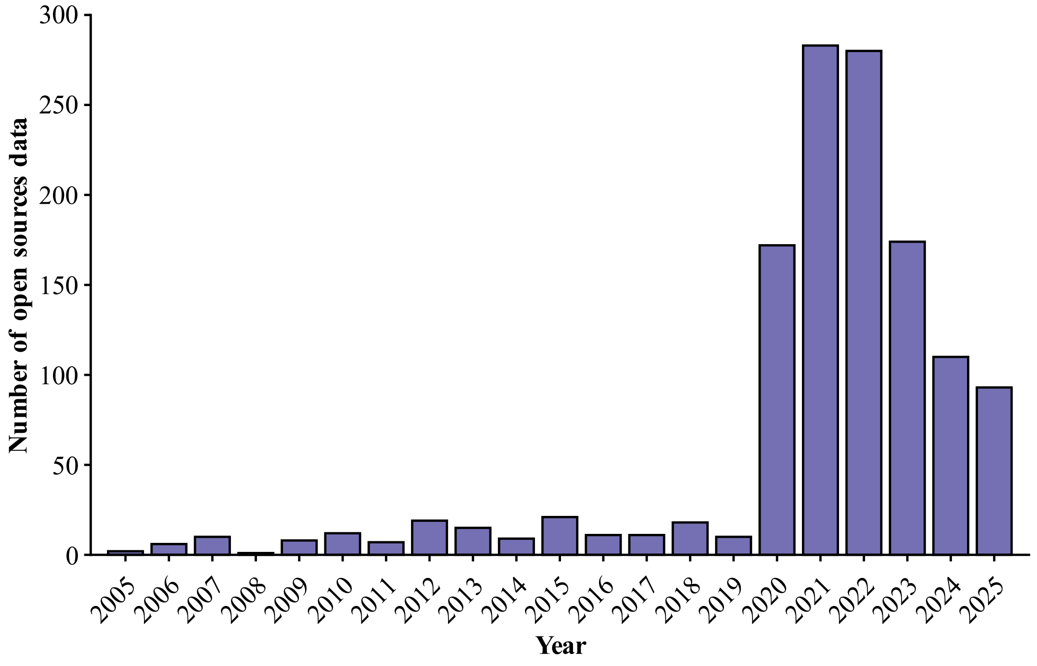

Figure S3. Number of open-access data sources used per year on influenza, RSV, and SARS-CoV-2 surveillance and research across 12 European countries, 2005–2025.

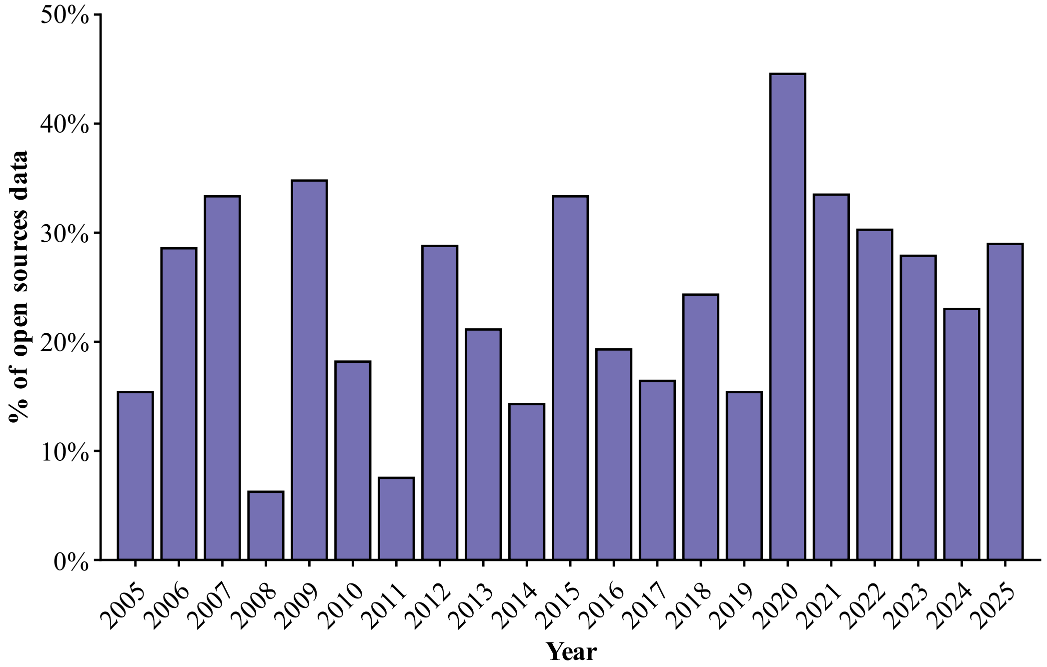

Figure S4. Proportion of open-access data sources used per year on influenza, RSV, and SARS-CoV-2 surveillance and research across 12 European countries, 2005–2025.

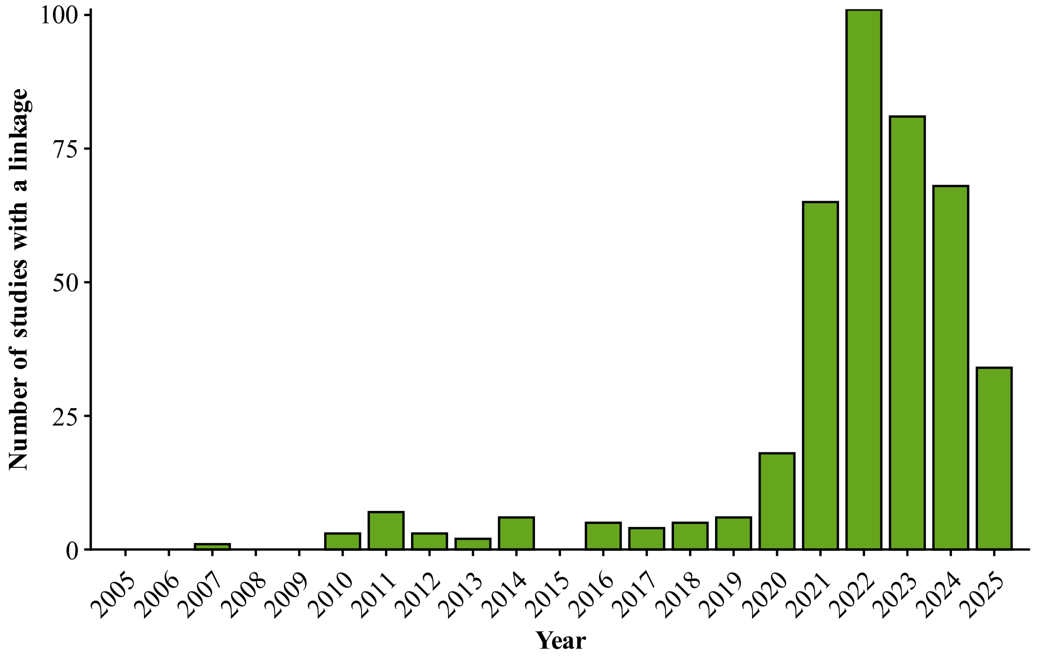

Figure S5. Number of reports with linkage between at least two data sources per year on influenza, RSV, and SARS-CoV-2 surveillance and research across 12 European countries, 2005–2025.

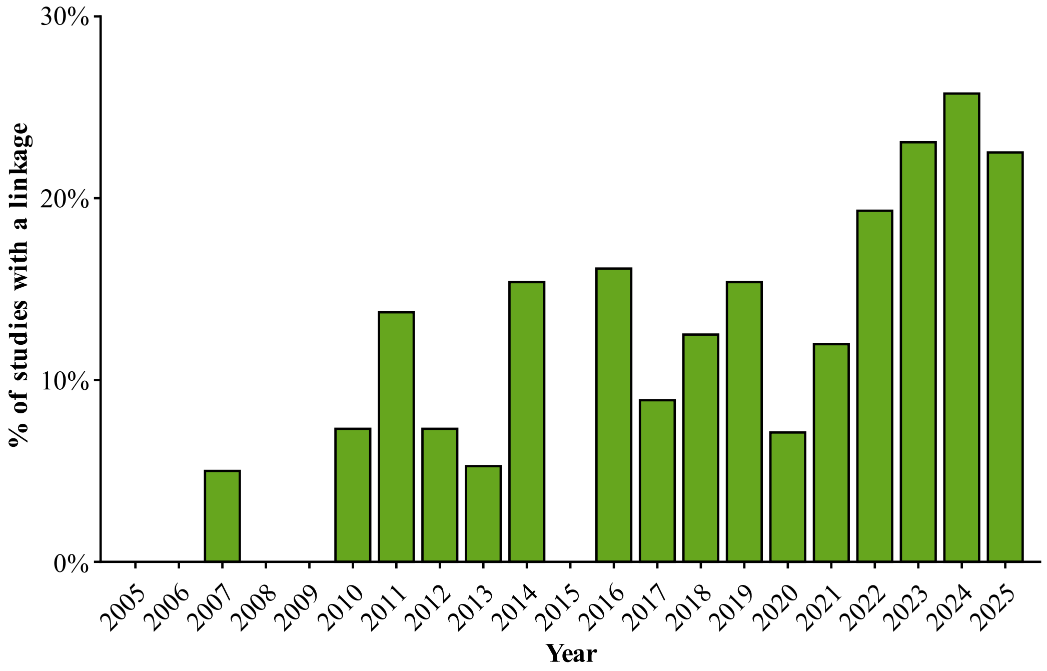

Figure S6. Proportion of reports with linkage between at least two data sources per year on influenza, RSV, and SARS-CoV-2 surveillance and research across 12 European countries, 2005–2025.

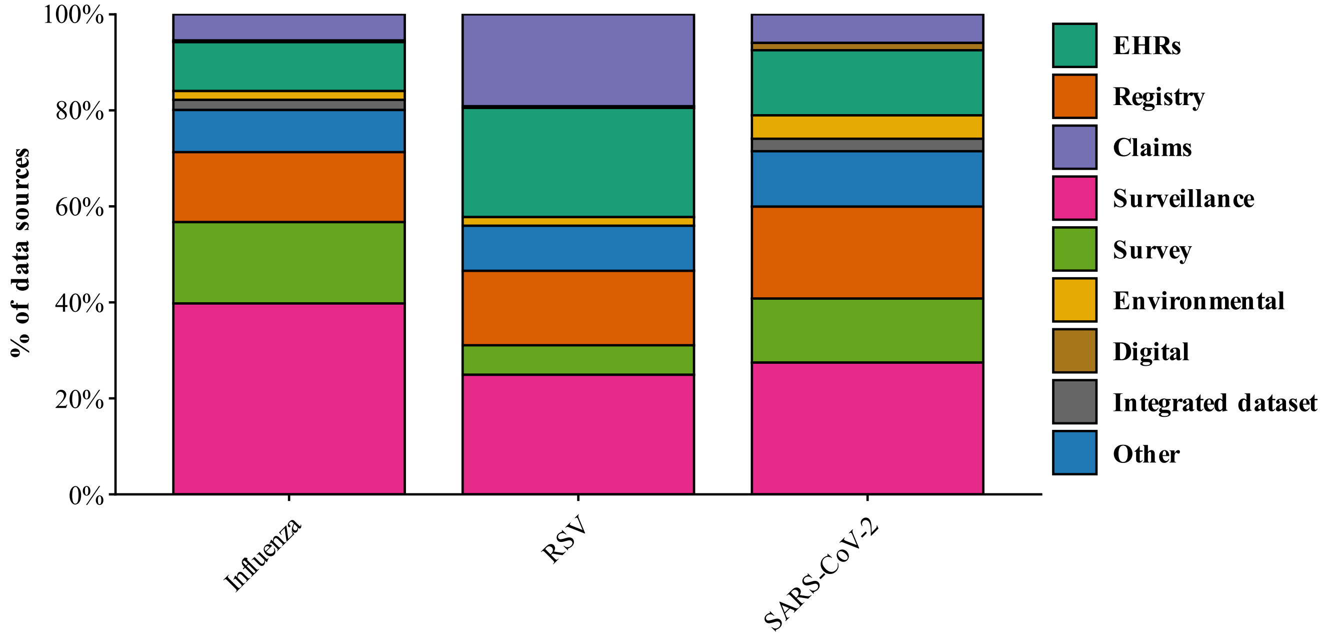

Figure S7. Proportion of data source types used on influenza, RSV, and SARS-CoV-2 surveillance and research, by virus, across 12 European countries, 2005–2025.

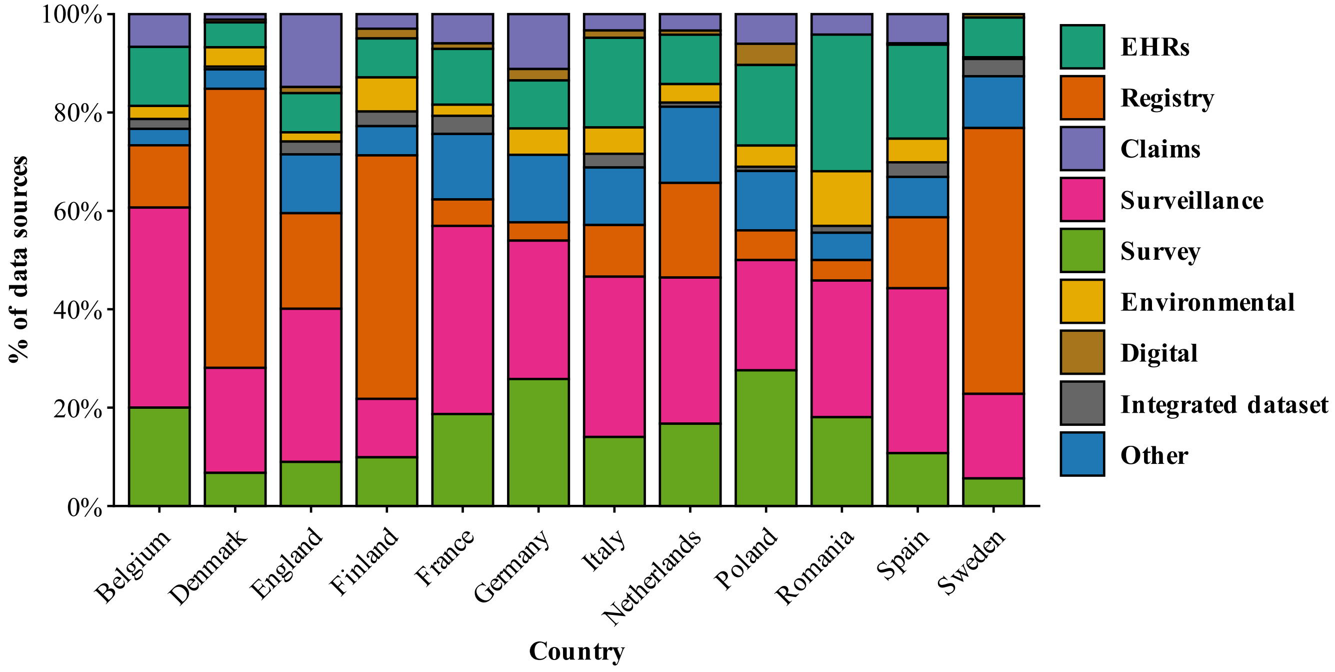

Figure S8. Proportion of data source types used on influenza, RSV, and SARS-CoV-2 surveillance and research, by country, across 12 European countries, 2005–2025.

References

Aabakke, A. J. M., Krebs, L., Petersen, T. G., Kjeldsen, F. S., Corn, G., Wøjdemann, K., Ibsen, M. H., Jonsdottir, F., Rønneberg, E., Andersen, C. S., Sundtoft, I., Clausen, T., Milbak, J., Burmester, L., Lindved, B., Thorsen-Meyer, A., Khalil, M. R., Henriksen, B., Jønsson, L., … Bliddal, M. (2021). SARS-CoV-2 infection in pregnancy in Denmark-characteristics and outcomes after confirmed infection in pregnancy : A nationwide, prospective, population-based cohort study. Acta Obstetricia Et Gynecologica Scandinavica, 100(11). https://doi.org/10.1111/aogs.14252

Abad, L., Antona, D., Roudier, C., Auvigne, V., Bastard, J., Blondel, C., Durand, J., Fayad, M., Forgeot, C., Figoni, J., Mailles, A., Moisan, F., Spaccaferri, G., Che, D., Levy-Bruhl, D., & Hulin, M. (2024). Keeping schools open during the emergence of the COVID-19 alpha variant : Impact on the circulation of SARS-CoV-2 among children during the 2020-2021 school year : Epidemiology of COVID-19 in children and adolescents during the 2020-2021 school year in France. Archives De Pediatrie: Organe Officiel De La Societe Francaise De Pediatrie, 31(8). https://doi.org/10.1016/j.arcped.2024.07.005

Aballéa, S., De Juanes, J. R., Barbieri, M., Martin, M., Chancellor, J., Oyagüez, I., Verwee, B., & Largeron, N. (2007). The cost effectiveness of influenza vaccination for adults aged 50 to 64 years : A model-based analysis for Spain. Vaccine, 25(39-40). https://doi.org/10.1016/j.vaccine.2007.07.033

Abbott, T. E. F., Fowler, A. J., Dobbs, T. D., Gibson, J., Shahid, T., Dias, P., Akbari, A., Whitaker, I. S., & Pearse, R. M. (2021). Mortality after surgery with SARS-CoV-2 infection in England : A population-wide epidemiological study. British Journal of Anaesthesia, 127(2). https://doi.org/10.1016/j.bja.2021.05.018

Abdul Aziz, N., Kirsebom, F. C. M., Allen, A., & Andrews, N. (2025). Effectiveness of Spring 2024 (Xbb.1.5) and Autumn 2024 (Jn.1) Covid-19 Vaccination Against Hospitalisation in England. SSRN. https://doi.org/10.2139/ssrn.5351383

Abenavoli, L., Cinaglia, P., Procopio, A. C., Serra, R., Aquila, I., Zanza, C., Longhitano, Y., Artico, M., Larussa, T., Boccuto, L., Ricci, P., & Luzza, F. (2021). SARS-CoV-2 Spread Dynamics in Italy : The Calabria Experience. Reviews on Recent Clinical Trials, 16(3). https://doi.org/10.2174/1574887116666210401124945

Abou Chakra, C. N., Blanquart, F., Vieillefond, V., Enouf, V., Visseaux, B., Haim-Boukobza, S., Josset, L., Rameix-Welti, M.-A., Lina, B., Nunes, M. C., RELAB Study group, & Bal, A. (2025). Vaccine effectiveness dynamics against influenza and SARS-CoV-2 in community-tested patients in France 2023-2024. Emerging Microbes & Infections, 14(1), 2466699. https://doi.org/10.1080/22221751.2025.2466699

Aboukorin, S., Han, H., & Mahran, M. (2023). Pandemic resilience planning: NPI measures and Covid-19 impacts in UK, Germany, and Italy. CITIES, 143. https://doi.org/10.1016/j.cities.2023.104621

Aboura, S. (2023). The role of climate on Covid-19 spread in France. International Journal of Environmental Health Research, 33(8). https://doi.org/10.1080/09603123.2022.2055747

Acosta, L., Soldevila, N., Torner, N., Martínez, A., Ayneto, X., Rius, C., Jané, M., Domínguez, A., & The Influenza Surveillance Network Of Catalonia Pidirac, null. (2021). Influenza Vaccine Effectiveness in Preventing Severe Outcomes in Patients Hospitalized with Laboratory-Confirmed Influenza during the 2017-2018 Season. A Retrospective Cohort Study in Catalonia (Spain). Viruses, 13(8). https://doi.org/10.3390/v13081465

Adamczuk, J., Kamiński, K. A., Sołomacha, S., Kazberuk, M., Chlabicz, M., Czupryna, P., Dunaj-Małyszko, J., Citko, A., Sowa, P., Dubatówka, M., Łapińska, M., Kiszkiel, Ł., Szczerbiński, Ł., Laskowski, P. P., Alimowski, M., & Moniuszko-Malinowska, A. (2025). Determinants and dynamics of the seroprevalence of anti-SARS-CoV-2 antibodies in Poland. Advances in Medical Sciences, 70(1), 219–228. https://doi.org/10.1016/j.advms.2025.03.002

Adlhoch, C., Wadl, M., Behnke, M., Diaz, L., Clausmeyer, J., & Eckmanns, T. (2012). Pandemic influenza A(H1)pdm09 in hospitals and intensive care units—Results from a new hospital surveillance, Germany 2009/2010. INFLUENZA AND OTHER RESPIRATORY VIRUSES, 6(6), e162–e168. https://doi.org/10.1111/j.1750-2659.2012.00404.x

Adriaenssens, N., Scholtes, B., Bruyndonckx, R., Van Ngoc, P., Verbakel, J. Y., De Sutter, A., Heytens, S., Van den Bruel, A., Desombere, I., Van Damme, P., Goossens, H., Buret, L., Duysburgh, E., & Coenen, S. (2022). The prevalence, incidence and longevity of antibodies against SARS-CoV-2 among primary healthcare providers in Belgium : A prospective cohort study with 12 months of follow-up. medRxiv, (Adriaenssens N.; Coenen S., samuel.coenen@uantwerpen.be) Centre for General Practice, Department of Family Medicine&Population Health (FAMPOP), University of Antwerp, Antwerp, Belgium. https://doi.org/10.1101/2022.06.17.22276478

Affanni, P., Colucci, M. E., Bracchi, M. T., Capobianco, E., Zoni, R., Caruso, L., Castrucci, M. R., Puzelli, S., Cantarelli, A., & Veronesi, L. (2019). Virological Surveillance of Influenza in the eight epidemic seasons after the 2009 pandemic in Emilia-Romagna (Northern Italy). Acta Bio-Medica: Atenei Parmensis, 90(9), 35–44. https://doi.org/10.23750/abm.v90i9-S.8722

Aghemo, A., Piovani, D., Parigi, T. L., Brunetta, E., Pugliese, N., Vespa, E., Omodei, P. D., Preatoni, P., Lleo, A., Repici, A., Voza, A., Cecconi, M., Malesci, A., Bonovas, S., Danese, S., & Humanitas COVID-19 Task Force. (2020). COVID-19 Digestive System Involvement and Clinical Outcomes in a Large Academic Hospital in Milan, Italy. Clinical Gastroenterology and Hepatology: The Official Clinical Practice Journal of the American Gastroenterological Association, 18(10). https://doi.org/10.1016/j.cgh.2020.05.011

Agrawal, S., Orschler, L., & Lackner, S. (2021). Long-term monitoring of SARS-CoV-2 RNA in wastewater of the Frankfurt metropolitan area in Southern Germany. Scientific Reports, 11(1). https://doi.org/10.1038/s41598-021-84914-2

Agrawal, S., Orschler, L., Tavazzi, S., Greither, R., Gawlik, B. M., & Lackner, S. (2022). Genome Sequencing of Wastewater Confirms the Arrival of the SARS-CoV-2 Omicron Variant at Frankfurt Airport but Limited Spread in the City of Frankfurt, Germany, in November 2021. Microbiology Resource Announcements, 11(2). https://doi.org/10.1128/MRA.01229-21

Agrawal, S., Orschler, L., Zachmann, K., & Lackner, S. (2023). Comprehensive mutation profiling from wastewater in southern Germany extends evidence of circulating SARS-CoV-2 diversity beyond mutations characteristic for Omicron. FEMS MICROBES, 4. https://doi.org/10.1093/femsmc/xtad006

Aguilar, I., Reyes, M., Martinez-Baz, I., Guevara, M., Albeniz, E., Belza, M., & Castilla, J. (2012). Use of the vaccination register to evaluate influenza vaccine coverage in seniors in the 2010/11 influenza season, Navarre, Spain. Euro Surveillance: Bulletin Europeen Sur Les Maladies Transmissibles = European Communicable Disease Bulletin, 17(17). https://doi.org/10.2807/ese.17.17.20154-en

Ahout, I. M. L., Philipsen, R. L. A., Las, M., Baysan, M., Brus, F., Rahamat-Langendoen, J. C., Roeleveld, N., Fraaij, P. L., Osterhaus, A. D. M. E., Ferwerda, G., de Groot, R., & Dutch H1N1 Research Group. (2018). Nationwide Study on the Course of Influenza A (H1N1) Infections in Hospitalized Children in the Netherlands During the Pandemic 2009-2010. The Pediatric Infectious Disease Journal, 37(12), e283–e291. https://doi.org/10.1097/INF.0000000000002177

Aiano, F., Ireland, G., Powell, A., Campbell, C. N. J., Judd, A., Davies, B., Saib, A., Mangtani, P., Nguipdop-Djomo, P., SIS Study Group, & Ladhani, S. N. (2023). Factors associated with COVID-19 vaccine uptake in adolescents : A national cross-sectional study, August 2021-January 2022, England. BMJ Open, 13(9). https://doi.org/10.1136/bmjopen-2023-071707

Aiano, F., Jones, S. E. I., Amin-Chowdhury, Z., Flood, J., Okike, I., Brent, A., Brent, B., Beckmann, J., Garstang, J., Ahmad, S., Baawuah, F., Ramsay, M. E., & Ladhani, S. N. (2021). Feasibility and acceptability of SARS-CoV-2 testing and surveillance in primary school children in England : Prospective, cross-sectional study. PloS One, 16(8). https://doi.org/10.1371/journal.pone.0255517

Aiano, F., McOwat, K., Obi, C., Powell, A. A., Flood, J., Bhardwaj, S., Stoker, K., Haskins, D., Wong, B., Bertran, M., Zavala, M., Bosowski, J., Jones, S. E. I., Amin-Chowdhury, Z., Coughlan, L., Sinnathamby, M., Zaidi, A., Merrick, R., Zhao, H., … Saliba, V. (2022). A cross-sectional national investigation of COVID-19 outbreaks in nurseries during rapid spread of the Alpha (B.1.1.7) variant of SARS-CoV-2 in England. BMC Public Health, 22(1). https://doi.org/10.1186/s12889-022-14228-z

Aiano, F., Mensah, A. A., McOwat, K., Obi, C., Vusirikala, A., Powell, A. A., Flood, J., Bosowski, J., Letley, L., Jones, S., Amin-Chowdhury, Z., Lacy, J., Hayden, I., Ismail, S. A., Ramsay, M. E., Ladhani, S. N., & Saliba, V.

(2021). COVID-19 outbreaks following full reopening of primary and secondary schools in England : Cross-sectional national surveillance, November 2020. The Lancet Regional Health. Europe, 6. https://doi.org/10.1016/j.lanepe.2021.100120

Ainslie, K. E. C., Backer, J. A., de Boer, P. T., van Hoek, A. J., Klinkenberg, D., Korthals Altes, H., Leung, K. Y., de Melker, H., Miura, F., & Wallinga, J. (2022). A scenario modelling analysis to anticipate the impact of COVID-19 vaccination in adolescents and children on disease outcomes in the Netherlands, summer 2021. Euro Surveillance: Bulletin Europeen Sur Les Maladies Transmissibles = European Communicable Disease Bulletin, 27(44). https://doi.org/10.2807/1560-7917.ES.2022.27.44.2101090

Airoldi, C., Patrucco, F., Milano, F., Alessi, D., Sarro, A., Rossi, M. A., Cena, T., Borrè, S., & Faggiano, F. (2021). High Seroprevalence of SARS-CoV-2 among Healthcare Workers in a North Italy Hospital. International Journal of Environmental Research and Public Health, 18(7). https://doi.org/10.3390/ijerph18073343

Alaa, A., Qian, Z., Rashbass, J., Benger, J., & van der Schaar, M. (2020). Retrospective cohort study of admission timing and mortality following COVID-19 infection in England. BMJ Open, 10(11). https://doi.org/10.1136/bmjopen-2020-042712

Alaniz, A. J., Carvajal, M. A., Carvajal, J. G., & Vergara, P. M. (2023). Effects of air pollution and weather on the initial COVID-19 outbreaks in United States, Italy, Spain, and China : A comparative study. Risk Analysis: An Official Publication of the Society for Risk Analysis, 43(1). https://doi.org/10.1111/risa.14080

Albani, V., Welsh, C. E., Brown, H., Matthews, F. E., & Bambra, C. (2022). Explaining the deprivation gap in COVID-19 mortality rates : A decomposition analysis of geographical inequalities in England. Social Science & Medicine (1982), 311. https://doi.org/10.1016/j.socscimed.2022.115319

Alchikh, M., Conrad, T. O. F., Obermeier, P. E., Ma, X., Schweiger, B., Opota, O., & Rath, B. A. (2024). Disease Burden and Inpatient Management of Children with Acute Respiratory Viral Infections during the Pre-COVID Era in Germany : A Cost-of-Illness Study. Viruses, 16(4). https://doi.org/10.3390/v16040507

Aldea, N. (2022). Mortality impact of the Covid-19 epidemic on immigrant populations in Spain. SSM - Population Health, 20. https://doi.org/10.1016/j.ssmph.2022.101291

Aldridge, R. W., Hayward, A. C., Field, N., Warren-Gash, C., Smith, C., Pebody, R., Fleming, D., McCracken, S., & Decipher my Data project and schools. (2016). Are School Absences Correlated with Influenza Surveillance Data in England ? Results from Decipher My Data-A Research Project Conducted through Scientific Engagement with Schools. PloS One, 11(3). https://doi.org/10.1371/journal.pone.0146964

Aldridge, R. W., Lewer, D., Katikireddi, S. V., Mathur, R., Pathak, N., Burns, R., Fragaszy, E. B., Johnson, A. M., Devakumar, D., Abubakar, I., & Hayward, A. (2020). Black, Asian and Minority Ethnic groups in England are at increased risk of death from COVID-19 : Indirect standardisation of NHS mortality data. Wellcome Open Research, 5. https://doi.org/10.12688/wellcomeopenres.15922.2

Alessi, D., Borré, S., Barale, A., Isabella, A., Milano, F., Rossi, M. A., Silano, V., Piu, N., Cena, T., Faggiano, F., & Gruppo di Lavoro Fondazione Valsesia. (2020). [Seroprevalence of anti-SARS-CoV-2 IgG/IgM antibodies in Borgosesia (Piedmont Region, Northern Italy) population : A surveillance strategy in post-lockdown period?]. Epidemiologia E Prevenzione, 44(5-6 Suppl 2). https://doi.org/10.19191/EP20.5-6.S2.119

Aleta, A., & Moreno, Y. (2020). Evaluation of the potential incidence of COVID-19 and effectiveness of containment measures in Spain : A data-driven approach. BMC Medicine, 18(1). https://doi.org/10.1186/s12916-020-01619-5

Aleta, A., Blas-Laína, J. L., Tirado Anglés, G., & Moreno, Y. (2023). Unraveling the COVID-19 hospitalization dynamics in Spain using Bayesian inference. BMC Medical Research Methodology, 23(1). https://doi.org/10.1186/s12874-023-01842-7

Aleva, F. E., van Mourik, L., Broeders, M. E. a. C., Paling, A. J., & de Jager, C. P. C. (2020). COVID-19 in critically ill patients in North Brabant, the Netherlands : Patient characteristics and outcomes. Journal of Critical Care, 60. https://doi.org/10.1016/j.jcrc.2020.08.001

Aliberti, S. M., Schiavo, L., Boccia, G., Santoro, E., Franci, G., Ruggiero, A., De Caro, F., & Capunzo, M. (2022). Gender and AB0 Blood Type Differences in a Unicentric Group of University Professors in Southern Italy Who Received the Vaxzevria COVID-19 Vaccine : A Cross-Sectional Survey of Vaccine Side Effects, Attitudes, and Hesitation. Vaccines, 10(3). https://doi.org/10.3390/vaccines10030373

Alicino, C., Iudici, R., Barberis, I., Paganino, C., Cacciani, R., Zacconi, M., Battistini, A., Bellina, D., Di Bella, A., Talamini, A., Sticchi, L., Morando, A., Ansaldi, F., & Durando, P. (2015). Influenza vaccination among healthcare workers in Italy The experience of a large tertiary acute-care teaching hospital. HUMAN VACCINES & IMMUNOTHERAPEUTICS, 11(1). https://doi.org/10.4161/hv.34362

Al-Jwadi, R. F., Mills, E. H. A., Torp-Pedersen, C., Andersen, M. P., & Jørgensen, I. M. (2023). Consequences of COVID-19-related lockdowns and reopenings on emergency hospitalizations in pediatric patients in Denmark during 2020-2021. European Journal of Pediatrics, 182(1). https://doi.org/10.1007/s00431-022-04682-7

Alleman, T. W., Vergeynst, J., De Visscher, L., Rollier, M., Torfs, E., Belgian Collaborative Group on COVID-19 Hospital Surveillance, Nopens, I., & Baetens, J. M. (2021). Assessing the effects of non-pharmaceutical interventions on SARS-CoV-2 transmission in Belgium by means of an extended SEIQRD model and public mobility data. Epidemics, 37. https://doi.org/10.1016/j.epidem.2021.100505

Allen, H., Hassell, K., Rawlinson, C., Pullen, O., Campbell, C., Jödicke, A. M., Català, M., Prats-Uribe, A., Dabrera, G., Prieto-Alhambra, D., & Campos-Matos, I. (2025). Development of the ECHOES national dataset: A resource for monitoring post-acute and long-term COVID-19 health outcomes in England. Frontiers in Public Health, 13, 1513508. https://doi.org/10.3389/fpubh.2025.1513508

Allen, H., Hassell, K., Rawlinson, C., Pullen, O., Campbell, C., Jödicke, A. M., Català, M., Uribe, A. P., Dabrera, G., Prieto-Alhambra, D., & Campos-Matos, I. (2024). Evaluation of post-acute COVID-19 health outcomes (ECHOES) in England : The development of national surveillance system for long-term health outcomes following COVID-19. medRxiv, (Allen H., hester.allen@seh.ox.ac.uk; Hassell K.; Rawlinson C.; Pullen O.; Campbell C.; Dabrera G.; Campos-Matos I.) Immunisation and Vaccine Preventable Diseases Division, UK Health Security Agency, 61 Colindale Avenue, London, United Kingdom. https://doi.org/10.1101/2024.10.18.24315744

Allen, H., Tessier, E., Turner, C., Anderson, C., Blomquist, P., Simons, D., Løchen, A., Jarvis, C. I., Groves, N., Capelastegui, F., Flannagan, J., Zaidi, A., Chen, C., Rawlinson, C., Hughes, G. J., Chudasama, D., Nash, S., Thelwall, S., Lopez-Bernal, J., … Lamagni, T. (2022). Comparative transmission of SARS-CoV-2 Omicron (B.1.1.529) and Delta (B.1.617.2) variants and the impact of vaccination : National cohort study, England. medRxiv, (Allen H.; Tessier E., Elise.Tessier@phe.gov.uk; Capelastegui F.; Flannagan J.; Zaidi A.; Chudasama D.; Nash S.; Thelwall S.; Dabrera G.; Kall M.; Lamagni T.) UK Health Security Agency, Wellington House, 133-155 Waterloo Road, London, United Kingdom. https://doi.org/10.1101/2022.02.15.22271001

Aloi, A., Alonso, B., Benavente, J., Cordera, R., Echániz, E., González, F., Ladisa, C., Lezama-Romanelli, R., López-Parra, A., Mazzei, V., Perrucci, L., Prieto-Quintana, D., Rodríguez, A., & Sañudo, R. (2020). Effects of the COVID-19 Lockdown on Urban Mobility : Empirical Evidence from the City of Santander (Spain). SUSTAINABILITY, 12(9). https://doi.org/10.3390/su12093870

Aloisi, V., Gatto, A., Accarino, G., Donato, F., & Aloisio, G. (2022). The effect of known and unknown confounders on the relationship between air pollution and Covid-19 mortality in Italy : A sensitivity analysis of an ecological study based on the E-value. Environmental Research, 207. https://doi.org/10.1016/j.envres.2021.112131

Alonso Bilbao, J. L., de Arriba Fernández, A., Espiñeira Francés, A., Cabeza Mora, A., Gutiérrez Pérez, A., & Díaz Barreiros, M. A. (2023). [Epidemiological study on the impact of influenza vaccination on the clinical course of patients with COVID-19 and co-infection by both viruses in Gran Canaria, Spain]. Revista Espanola De Quimioterapia: Publicacion Oficial De La Sociedad Espanola De Quimioterapia, 36(2). https://doi.org/10.37201/req/102.2022

Alteri, C., Cento, V., Piralla, A., Costabile, V., Tallarita, M., Colagrossi, L., Renica, S., Giardina, F., Novazzi, F., Gaiarsa, S., Matarazzo, E., Antonello, M., Vismara, C., Fumagalli, R., Epis, O. M., Puoti, M., Perno, C. F., & Baldanti, F. (2021). Genomic epidemiology of SARS-CoV-2 reveals multiple lineages and early spread of

SARS-CoV-2 infections in Lombardy, Italy. Nature Communications, 12(1). https://doi.org/10.1038/s41467-020-20688-x

Altes, H. K., de Kassteele, J. V., Wisse, B., Xiridou, M., van Hoek, A. J., & Wallinga, J. (2025). Work absenteeism across economic activity sectors and its association with COVID-19-like illness prevalence in the Netherlands, 2020-2023. medRxiv. https://doi.org/10.1101/2025.05.26.25328340

Altunkaya, J., Piernas, C., Pouwels, K. B., Jebb, S. A., Clarke, P., Astbury, N. M., & Leal, J. (2024). Associations between BMI and hospital resource use in patients hospitalised for COVID-19 in England : A community-based cohort study. The Lancet. Diabetes & Endocrinology, 12(7). https://doi.org/10.1016/S2213-8587(24)00129-3

Álvarez Aldean, J., Rivero Calle, I., Rodríguez Fernández, R., Aceituno Mata, S., Bellmunt, A., Prades, M., Law, A. W., López-Ibáñez de Aldecoa, A., Méndez, C., García Somoza, M. L., Soto, J., & Lozano, V. (2024). Cost-effectiveness Analysis of Maternal Immunization with RSVpreF Vaccine for the Prevention of Respiratory Syncytial Virus Among Infants in Spain. Infectious Diseases and Therapy, 13(6). https://doi.org/10.1007/s40121-024-00975-6

Alvarez, F. P., Chevalier, P., Borms, M., Bricout, H., Marques, C., Soininen, A., Sainio, T., Petit, C., & de Courville, C. (2023). Cost-effectiveness of influenza vaccination with a high dose quadrivalent vaccine of the elderly population in Belgium, Finland, and Portugal. Journal of Medical Economics, 26(1). Embase. https://doi.org/10.1080/13696998.2023.2194193

Álvarez-Arroyo, L., Carrera-Hueso, F. J., El-Qutob, D., Robustillo-Villarino, M., Girona-Sanz, A. M., Pin-Godos, M. T., Sánchez-Monzó, P., Martínez-Gonzálbez, R., Cepeda-Madrigal, S., & Martínez-Martínez, F. (2021). Descriptive study of a cohort of COVID-19 hospitalized patients in Spain. Gaceta Medica De Mexico, 157(1). https://doi.org/10.24875/GMM.M21000525

Álvarez-Del Río, B., Sánchez-de Prada, L., Álvaro-Meca, A., Martín-Fernández, M., Álvarez, F. J., Tamayo, E., & Gutiérrez-Abejón, E. (2023). Prevalence and cost of hospitalized patients with asymptomatic COVID-19 in 2020 in Spain. Frontiers in Public Health, 11. https://doi.org/10.3389/fpubh.2023.1229561

Álvarez-Del Río, B., Sánchez-de Prada, L., Arroyo-Hernantes, I., Álvarez, F. J., Tamayo, E., & Gutiérrez-Abejón, E. (2025). Nationwide analysis of COVID-19 complications, outcomes, and costs of childbirth in Spain. Frontiers in Medicine, 12, 1548245. https://doi.org/10.3389/fmed.2025.1548245

Álvarez-Esteban, P. C., Del Barrio, E., Rueda, O. M., & Rueda, C. (2021). Predicting COVID-19 progression from diagnosis to recovery or death linking primary care and hospital records in Castilla y León (Spain). PloS One, 16(9). https://doi.org/10.1371/journal.pone.0257613

Alvarez-Fischer, D., Borsche, M., Balck, A., Föh, B., Hoischen, A., Hotak, F., Reinhardt, J., Elsner, S., Peters, E., Rieck, A., Martin, E., Kuensting, I., Ehlers, M., Mischnik, A., Taube, S., Käding, N., Rupp, J., Katalinic, A., & Klein, C. (2025). Spreading of SARS-CoV-2 among adult asylum seekers in refugee community shelters in Lübeck, Germany between 2020 and 2022: A mixed-cohort observational and repeated cross-sectional study. BMC PUBLIC HEALTH, 25(1). https://doi.org/10.1186/s12889-025-22120-9

Amer, F., Lan, F.-Y., Gil-Conesa, M., Sidossis, A., Bruque, D., Iliaki, E., Buley, J., Nathan, N., Bruno-Murtha, L. A., Carlos, S., Kales, S. N., & Fernandez-Montero, A. (2025). Evolving SARS-CoV-2 severity among hospital and university affiliates in Spain and Greater Boston. Enfermedades Infecciosas Y Microbiologia Clinica (English Ed.), 43(1). https://doi.org/10.1016/j.eimce.2023.12.004

Ameri, P., Inciardi, R. M., Di Pasquale, M., Agostoni, P., Bellasi, A., Camporotondo, R., Canale, C., Carubelli, V., Carugo, S., Catagnano, F., Danzi, G., Dalla Vecchia, L., Giovinazzo, S., Gnecchi, M., Guazzi, M., Iorio, A., La Rovere, M. T., Leonardi, S., Maccagni, G., … Metra, M. (2021). Pulmonary embolism in patients with COVID-19 : Characteristics and outcomes in the Cardio-COVID Italy multicenter study. Clinical Research in Cardiology: Official Journal of the German Cardiac Society, 110(7). https://doi.org/10.1007/s00392-020-01766-y

Amirthalingam, G., Bernal, J. L., Andrews, N. J., Whitaker, H., Gower, C., Stowe, J., Tessier, E., Subbarao, S., Ireland, G., Baawuah, F., Linley, E., Warrener, L., O’Brien, M., Whillock, C., Moss, P., Ladhani, S. N., Brown,

K. E., & Ramsay, M. E. (2021). Serological responses and vaccine effectiveness for extended COVID-19 vaccine schedules in England. Nature Communications, 12(1). https://doi.org/10.1038/s41467-021-27410-5

Amodio, E., Battisti, M., Maida, C. M., Zarcone, M., Casuccio, A., & Vitale, F. (2021). Socio-Demographic Factors Involved in a Low-Incidence Phase of SARS-CoV-2 Spread in Sicily, Italy. Healthcare (Basel, Switzerland), 9(7). https://doi.org/10.3390/healthcare9070867

Amore, S., Puppo, E., Melara, J., Terracciano, E., Gentili, S., & Liotta, G. (2021). Impact of COVID-19 on older adults and role of long-term care facilities during early stages of epidemic in Italy. Scientific Reports, 11(1). https://doi.org/10.1038/s41598-021-91992-9

Amour, S., Bénet, T., Regis, C., Robert, O., Fontana, L., Lina, B., Pozzetto, B., Berthelot, P., & Vanhems, P. (2022). Effect of influenza vaccination among healthcare workers on hospital-acquired influenza in short-stay hospitalized patients : A multicenter pilot study in France. Infection Control and Hospital Epidemiology, 43(12). https://doi.org/10.1017/ice.2022.68

Andersen, A. L., Gribsholt, S. B., Pedersen, L., Thomsen, R. W., Benfield, T. L., Søgaard, O., Nielsen, S. L., Omland, L. H., Lindegaard, B., Richelsen, B., Bodilsen, J., & Bruun, J. M. (2023). The impact of age and obesity on outcomes among patients hospitalized with COVID-19 in Denmark : A nationwide cohort study. Obesity Science & Practice, 9(4). https://doi.org/10.1002/osp4.659

Andersen, P. A., Rasmussen, K. M. B., Channir, H. I., von Buchwald, C., Cayé-Thomasen, P., Klokker, M., Knudsen, J. D., Kirkby, N. S., Aanaes, K., & Jensen, R. G. (2021). The impact and prevalence of SARS-CoV-2 in patients with head and neck cancer and acute upper airway infection in a tertiary otorhinolaryngology referral center in Denmark. European Archives of Oto-Rhino-Laryngology: Official Journal of the European Federation of Oto-Rhino-Laryngological Societies (EUFOS): Affiliated with the German Society for Oto-Rhino-Laryngology - Head and Neck Surgery, 278(9). https://doi.org/10.1007/s00405-020-06514-6

Andersen, Z. J., Zhang, J., Lim, Y.-H., So, R., Jørgensen, J. T., Mortensen, L. H., Napolitano, G. M., Cole-Hunter, T., Loft, S., Bhatt, S., Hoek, G., Brunekreef, B., Westendorp, R., Ketzel, M., Brandt, J., Lange, T., & Kølsen-Fisher, T. (2023). Long-Term Exposure to AIR Pollution and COVID-19 Mortality and Morbidity in DENmark : Who Is Most Susceptible ? (AIRCODEN). Research Report (Health Effects Institute), 2023(214). http://www.ncbi.nlm.nih.gov/pubmed/38286761

Andeweg, S., de Gier, B., Vennema, H., van Walle, I., van Maarseveen, N., Kusters, N., de Melker, H., Hahne, S., van den Hof, S., Eggink, D., & Knol, M. (2023). Higher risk of SARS-CoV-2 Omicron BA.4/5 infection than of BA.2 infection after previous BA.1 infection, the Netherlands, 2 May to 24 July 2022. EUROSURVEILLANCE, 28(7). https://doi.org/10.2807/1560-7917.ES.2023.28.7.2200724

Andreadis, S., Antzoulatos, G., Mavropoulos, T., Giannakeris, P., Tzionis, G., Pantelidis, N., Ioannidis, K., Karakostas, A., Gialampoukidis, I., Vrochidis, S., & Kompatsiaris, I. (2021). A social media analytics platform visualising the spread of COVID-19 in Italy via exploitation of automatically geotagged tweets. Online Social Networks and Media, 23. https://doi.org/10.1016/j.osnem.2021.100134

Andrés, C., Del Cuerpo, M., Rabella, N., Piñana, M., Iglesias-Cabezas, M. J., González-Sánchez, A., Esperalba, J., Rando, A., Martín, M. C., Fuentes, F., Rubio, S., Saubi, N., Pumarola, T., & Antón, A. (2023). Detection of reassortant influenza B strains from 2004 to 2015 seasons in Barcelona (Catalonia, Spain) by whole genome sequencing. Virus Research, 330. https://doi.org/10.1016/j.virusres.2023.199089

Andrés, C., Peremiquel-Trillas, P., Gimferrer, L., Piñana, M., Codina, M. G., Rodrigo-Pendás, J. Á., Campins-Martí, M., Carmen Martín, M., Fuentes, F., Rubio, S., Pumarola, T., & Antón, A. (2019). Molecular influenza surveillance at a tertiary university hospital during four consecutive seasons (2012-2016) in Catalonia, Spain. Vaccine, 37(18). https://doi.org/10.1016/j.vaccine.2019.03.046

Andrews, C. D., Prestige, E., Parker, E. P. K., Walker, V., Palmer, T., Schaffer, A. L., Green, A. C. A., Curtis, H. J., Walker, A., Smith, R. M., Wood, C., Bates, C., Mehrkar, A., MacKenna, B., Bacon, S. C. J., Goldacre, B., Hernán, M. A., Sterne, J. A. C., & Hulme, W. J. (2024). Comparative safety and effectiveness of Pfizer BA.4-5 versus Sanofi during the spring 2023 COVID-19 booster vaccination programme in England: A matched cohort study in OpenSAFELY-TPP. medRxiv. https://doi.org/10.1101/2024.03.15.24304277

Andrews, N., Osuntoki, I., Stowe, J., Kirsebom, F. C. M., Allen, A., & Lopez Bernal, J. (2024). The impact of COVID-19 vaccine spring boosters on COVID-19 hospital admissions in England 2022/23. The Journal of Infection, 89(3). https://doi.org/10.1016/j.jinf.2024.106221

Andrews, N., Stowe, J., Kirsebom, F., Gower, C., Ramsay, M., & Lopez, J. B. (2021). Effectiveness of BNT162b2 (Comirnaty, Pfizer-BioNTech) COVID-19 booster vaccine against COVID-19 related symptoms in England : Test negative case-control study. medRxiv, (Andrews N.; Stowe J.; Kirsebom F.; Gower C.; Ramsay M.; Lopez J.B., jamie.lopezbernal2@phe.gov.uk) UK Health Security Agency, London, United Kingdom. https://doi.org/10.1101/2021.11.15.21266341

Andrews, N., Stowe, J., Kirsebom, F., Toffa, S., Sachdeva, R., Gower, C., Ramsay, M., & Lopez Bernal, J. (2022). Effectiveness of COVID-19 booster vaccines against COVID-19-related symptoms, hospitalization and death in England. Nature Medicine, 28(4). https://doi.org/10.1038/s41591-022-01699-1

Ang, H. J., Menegale, F., Preziosi, G., Pariani, E., Migliari, M., Pellegrinelli, L., Sechi, G. M., Buoro, S., Merler, S., Cereda, D., Tirani, M., Poletti, P., & Dorigatti, I. (2023). Reconstructing the impact of COVID-19 on the immunity gap and transmission of respiratory syncytial virus in Lombardy, Italy. EBioMedicine, 95. https://doi.org/10.1016/j.ebiom.2023.104745

Antón, A., Marcos, M. A., Codoñer, F. M., de Molina, P., Martínez, A., Cardeñosa, N., Godoy, P., Torner, N., Martínez, M. J., Ramón, S., Tudó, G., Isanta, R., Gonzalo, V., de Anta, M. T. J., & Pumarola, T. (2011). Influenza C virus surveillance during the first influenza A (H1N1) 2009 pandemic wave in Catalonia, Spain. Diagnostic Microbiology and Infectious Disease, 69(4), 419–427. https://doi.org/10.1016/j.diagmicrobio.2010.11.006

Antón, A., Marcos, M. A., Torner, N., Isanta, R., Camps, M., Martínez, A., Domínguez, A., Jané, M., Jiménez de Anta, M. T., & Pumarola, T. (2016). Virological surveillance of influenza and other respiratory viruses during six consecutive seasons from 2006 to 2012 in Catalonia, Spain. Clinical Microbiology and Infection: The Official Publication of the European Society of Clinical Microbiology and Infectious Diseases, 22(6). https://doi.org/10.1016/j.cmi.2016.02.007

Antonelli, E., Piccolomini, E. L., & Zama, F. (2022). Switched forced SEIRDV compartmental models to monitor COVID-19 spread and immunization in Italy. Infectious Disease Modelling, 7(1). https://doi.org/10.1016/j.idm.2021.11.001

Antonini, C., Calandrini, S., & Bianconi, F. (2021). A Modeling Study on Vaccination and Spread of SARS-CoV-2 Variants in Italy. Vaccines, 9(8). https://doi.org/10.3390/vaccines9080915

Antonini, C., Calandrini, S., & Bianconi, F. (2022). Robustness analysis for quantitative assessment of vaccination effects and SARS-CoV-2 lineages in Italy. BMC Infectious Diseases, 22(1). https://doi.org/10.1186/s12879-022-07395-2

Antonini, C., Calandrini, S., Stracci, F., Dario, C., & Bianconi, F. (2020). Mathematical Modeling and Robustness Analysis to Unravel COVID-19 Transmission Dynamics : The Italy Case †. BIOLOGY-BASEL, 9(11). https://doi.org/10.3390/biology9110394

Antwi-Berko, D., Bakuri, A. Z., Otabil, K. B., & Kwarteng, A. (2022). Determinants and Variations of COVID-19 Vaccine Uptake and Responses Among Minority Ethnic Groups in Amsterdam, the Netherlands. Frontiers in Public Health, 10. https://doi.org/10.3389/fpubh.2022.761987

Apolone, G., Montomoli, E., Manenti, A., Boeri, M., Sabia, F., Hyseni, I., Mazzini, L., Martinuzzi, D., Cantone, L., Milanese, G., Sestini, S., Suatoni, P., Marchianò, A., Bollati, V., Sozzi, G., & Pastorino, U. (2021). Unexpected detection of SARS-CoV-2 antibodies in the prepandemic period in Italy. Tumori, 107(5). https://doi.org/10.1177/0300891620974755

Aradhya, S., Brandén, M., Drefahl, S., Obućina, O., Andersson, G., Rostila, M., Mussino, E., & Juárez, S. P. (2021). Intermarriage and COVID-19 mortality among immigrants. A population-based cohort study from Sweden. BMJ Open, 11(9). https://doi.org/10.1136/bmjopen-2021-048952

Ares-Gómez, S., Mallah, N., Santiago-Pérez, M.-I., Pardo-Seco, J., Pérez-Martínez, O., Otero-Barrós, M.-T., Suárez-Gaiche, N., Kramer, R., Jin, J., Platero-Alonso, L., Alvárez-Gil, R.-M., Ces-Ozores, O.-M., Nartallo-

Penas, V., Mirás-Carballal, S., Piñeiro-Sotelo, M., Malvar-Pintos, A., González-Pérez, J.-M., Rodríguez-Tenreiro-Sánchez, C., Rivero-Calle, I., … NIRSE-GAL study group. (2024). Effectiveness and impact of universal prophylaxis with nirsevimab in infants against hospitalisation for respiratory syncytial virus in Galicia, Spain : Initial results of a population-based longitudinal study. The Lancet. Infectious Diseases, 24(8). https://doi.org/10.1016/S1473-3099(24)00215-9

Arghittu, A., Dettori, M., Masia, M. D., Azara, A., Dempsey, E., & Castiglia, P. (2018). Social deprivation indexes and anti-influenza vaccination coverage in the elderly in Sardinia, Italy, with a focus on the Sassari municipality. Journal of Preventive Medicine and Hygiene, 59(4 Suppl 2). https://doi.org/10.15167/2421-4248/jpmh2018.59.4s2.1077

Arienti, C., Brambilla, L., Campagnini, S., Fanciullacci, C., Giunco, F., Mannini, A., Patrini, M., Tartarone, F., & Carrozza, M. C. (2021). Mortality and characteristics of older people dying with COVID-19 in Lombardy nursing homes, Italy : An observational cohort study. Journal of Research in Medical Sciences: The Official Journal of Isfahan University of Medical Sciences, 26. https://doi.org/10.4103/jrms.JRMS_1012_20

Ariës, M. J. H., van den Bergh, J. P., Beudel, M., Boersma, W., Dormans, T., Douma, R. A., Eerens, A., Elbers, P. W. G., Fleuren, L. M., Gritters van den Oever, N. C., de Haan, L., van der Horst, I. J. C. C., Hu, S., Hubers, D., Janssen, M. L. F., de Kruif, M., Kubben, P. L., van Kuijk, S. M. J., Noordzij, P. G., … Wyers, C. E. (2021). [Clinical course of COVID-19 in the Netherlands : An overview of 2607 patients in hospital during the first wave]. Nederlands Tijdschrift Voor Geneeskunde, 165. http://www.ncbi.nlm.nih.gov/pubmed/33651497

Arlotto, S., Garès, A., Giraud-Gatineau, A., Lagier, J. C., Jimeno, M.-T., Peretti-Watel, P., Million, M., Parola, P., Brouqui, P., Raoult, D., & Gentile, S. (2021). Life-years lost by COVID-19 patients in public hospitals of Marseille (APHM-South-Eastern France) : A limited death toll : A retrospective analysis. BMJ Open, 11(12). https://doi.org/10.1136/bmjopen-2021-049475

Arlotto, S., Legueult, K., Blin, A., Cortaredona, S., Giraud-Gatineau, A., Bailly, L., Jimeno, M.-T., Delorme, L., Brouqui, P., Lagier, J.-C., Million, M., Dellamonica, J., Colson, P., Carles, M., Raoult, D., Pradier, C., & Gentile, S. (2022). Who Were Hospitalized Deceased Patients from COVID-19 During the First Year of Pandemic? Retrospective Analysis of 1104 Deceased Patients in South of France. Journal of Epidemiology and Global Health, 12(2), 196–205. https://doi.org/10.1007/s44197-022-00039-3

Armocida, B., Zamagni, G., Magni, E., Monasta, L., Comar, M., Zanotta, N., Cason, C., Argentini, G., Urriza, M., Cassone, A., Vascotto, F., Buzzetti, R., Barbi, E., Del Pin, M., Pani, P., Knowles, A., Carletti, C., Concina, F., Milinco, M., & Ronfani, L. (2022). Clinical, anamnestic, and sociodemographic predictors of positive SARS-CoV-2 testing in children : A cross sectional study in a tertiary hospital in Italy. PloS One, 17(1). https://doi.org/10.1371/journal.pone.0262923

Armstrong, J., Rudkin, J. K., Allen, N., Crook, D. W., Wilson, D. J., Wyllie, D. H., & O'Connell, A. M. (2020). Dynamic linkage of COVID-19 test results between Public Health England's Second Generation Surveillance System and UK Biobank. Microbial Genomics, 6(7). https://doi.org/10.1099/mgen.0.000397

Arnold, K. F., Gilthorpe, M. S., Alwan, N. A., Heppenstall, A. J., Tomova, G. D., McKee, M., & Tennant, P. W. G. (2022). Estimating the effects of lockdown timing on COVID-19 cases and deaths in England : A counterfactual modelling study. PloS One, 17(4). https://doi.org/10.1371/journal.pone.0263432

Arteaga Duarte, C. H., Peters, M. L., de Goeij, M. H. M., Spijkerman, R., & Postma, M. J. (2025). Cost-effectiveness of nirmatrelvir/ritonavir in COVID-19 patient groups at high risk for progression to severe COVID-19 in the Netherlands. Cost Effectiveness and Resource Allocation: C/E, 23(1), 5. https://doi.org/10.1186/s12962-025-00604-0

Aschele, C., Negru, M. E., Pastorino, A., Cavanna, L., Zagonel, V., Barone-Adesi, F., & Blasi, L. (2021). Incidence of SARS-CoV-2 Infection Among Patients Undergoing Active Antitumor Treatment in Italy. JAMA Oncology, 7(2). https://doi.org/10.1001/jamaoncol.2020.6778

Assche, S. B.-V., Ferraccioli, F., Riccetti, N., Gomez-Ramirez, J., Ghio, D., & Stilianakis, N. I. (2024). Urban-rural disparities in COVID-19 hospitalisations and mortality : A population-based study on national surveillance data from Germany and Italy. PloS One, 19(5). https://doi.org/10.1371/journal.pone.0301325

Asta, F., Michelozzi, P., De Sario, M., Santelli, E., Bauleo, L., Cozzi, I., Vairo, F., Davoli, M., & Porta, D. (2022). [Impact of the COVID-19 epidemic on total and cause-specific mortality in Rome (Italy) in 2020]. Epidemiologia E Prevenzione, 46(1-2). https://doi.org/10.19191/EP22.1-2.A003.003

Astray-Mochales, J., López de Andres, A., Hernandez-Barrera, V., Rodríguez-Rieiro, C., Carrasco Garrido, P., Esteban-Vasallo, M. D., Domínguez-Berjón, M. F., Jimenez-Trujillo, I., & Jiménez-García, R. (2016). Influenza vaccination coverages among high risk subjects and health care workers in Spain. Results of two consecutive National Health Surveys (2011-2014). Vaccine, 34(41). https://doi.org/10.1016/j.vaccine.2016.08.065

Atchison, C. J., Davies, B., Cooper, E., Lound, A., Whitaker, M., Hampshire, A., Azor, A., Donnelly, C. A., Chadeau-Hyam, M., Cooke, G. S., Ward, H., & Elliott, P. (2023). Long-term health impacts of COVID-19 among 242,712 adults in England. Nature Communications, 14(1). https://doi.org/10.1038/s41467-023-41879-2

Atchison, C. J., Whitaker, M., Donnelly, C. A., Chadeau-Hyam, M., Riley, S., Darzi, A., Ashby, D., Barclay, W., Cooke, G. S., Elliott, P., & Ward, H. (2023). Characteristics and predictors of persistent symptoms post-COVID-19 in children and young people : A large community cross-sectional study in England. Archives of Disease in Childhood, 108(7). https://doi.org/10.1136/archdischild-2022-325152

Aturinde, A., & Mansourian, A. (2022). Space-Time Surveillance of COVID-19 Seasonal Clusters : A Case of Sweden. ISPRS INTERNATIONAL JOURNAL OF GEO-INFORMATION, 11(5). https://doi.org/10.3390/ijgi11050307

Auranen, K., Shubin, M., Erra, E., Isosomppi, S., Kontto, J., Leino, T., & Lukkarinen, T. (2023). Efficacy and effectiveness of case isolation and quarantine during a growing phase of the COVID-19 epidemic in Finland. Scientific Reports, 13(1). https://doi.org/10.1038/s41598-022-27227-2

Auvigne, V., Vaux, S., Strat, Y. L., Schaeffer, J., Fournier, L., Tamandjou, C., Montagnat, C., Coignard, B., Levy-Bruhl, D., & Parent du Châtelet, I. (2022). Severe hospital events following symptomatic infection with Sars-CoV-2 Omicron and Delta variants in France, December 2021-January 2022 : A retrospective, population-based, matched cohort study. EClinicalMedicine, 48. https://doi.org/10.1016/j.eclinm.2022.101455

Averin, A., Huebbe, B., Atwood, M., Bayer, L. J., Lade, C., von Eiff, C., & Sato, R. (2025). Cost-effectiveness of bivalent respiratory syncytial virus prefusion F vaccine for prevention of respiratory syncytial virus among older adults in Germany. Expert Review of Vaccines, 24(1). https://doi.org/10.1080/14760584.2024.2436183

Aweimer, A., Petschulat, L., Jettkant, B., Köditz, R., Finkeldei, J., Dietrich, J. W., Breuer, T., Draese, C., Frey, U. H., Rahmel, T., Adamzik, M., Buchwald, D., Useini, D., Brechmann, T., Hosbach, I., Bünger, J., Ewers, A., El-Battrawy, I., & Mügge, A. (2023). Mortality rates of severe COVID-19-related respiratory failure with and without extracorporeal membrane oxygenation in the Middle Ruhr Region of Germany. Scientific Reports, 13(1). https://doi.org/10.1038/s41598-023-31944-7

Aziz, N. A., Andrews, N., Rawlinson, C., Buckley, A., & Allen, A. (2024). Effectiveness of COVID-19 vaccines against SARS-CoV-2 infection and symptomatic infection in the England Winter Coronavirus (COVID-19) Infection Study cohort. medRxiv, (Aziz N.A., iwanibintiabdulaziz@ukhsa.gov.uk; Andrews N.; Rawlinson C.; Allen A.) Immunisations and Vaccine-Preventable Diseases Division, UK Health Security Agency, United Kingdom. https://doi.org/10.1101/2024.12.30.24319550

Azzolina, D., Lorenzoni, G., Silvestri, L., Prosepe, I., Berchialla, P., & Gregori, D. (2022). Regional Differences in Mortality Rates During the COVID-19 Epidemic in Italy. Disaster Medicine and Public Health Preparedness, 16(4). https://doi.org/10.1017/dmp.2020.486

Azzolina, D., Magnani, C., Gallo, E., Ferrante, D., & Gregori, D. (2020). [Gender and age factors affecting the mortality during the COVID-19 epidemic in Italy]. Epidemiologia E Prevenzione, 44(5-6 Suppl 2). https://doi.org/10.19191/EP20.5-6.S2.125

Azzolini, E., Lupo Pasinetti, B., Voza, A., Desai, A., Bartoletti, M., Aliberti, S., & Greco, M. (2025). COVID-19 Vaccination Still Makes Sense: Insights on Pneumonia Risk and Hospitalization from a Large-Scale Study at an Academic Tertiary Center in Italy. Microorganisms, 13(8), 1744. https://doi.org/10.3390/microorganisms13081744

Babac, M. B., & Mornar, V. (2020). Resetting the Initial Conditions for Calculating Epidemic Spread : COVID-19 Outbreak in Italy. IEEE Access: Practical Innovations, Open Solutions, 8. https://doi.org/10.1109/ACCESS.2020.3015923

Bach-Mortensen, A. M., & Degli Esposti, M. (2021). Is area deprivation associated with greater impacts of COVID-19 in care homes across England ? A preliminary analysis of COVID-19 outbreaks and deaths. Journal of Epidemiology and Community Health, 75(7). https://doi.org/10.1136/jech-2020-215039

Bachour, Y., Wynberg, E., Coyer, L., Buster, M., Schreijer, A., van Duijnhoven, Y. T. H. P., van Dam, A. P., Prins, M., & Leenstra, T. (2023). COVID-19 burden differed by city districts and ethnicities during the pre-vaccination era in Amsterdam, the Netherlands. Frontiers in Public Health, 11. https://doi.org/10.3389/fpubh.2023.1166193

Backer, J. A., Wallinga, J., Meijer, A., Donker, G. A., van der Hoek, W., & van Boven, M. (2019). The impact of influenza vaccination on infection, hospitalisation and mortality in the Netherlands between 2003 and 2015. Epidemics, 26. https://doi.org/10.1016/j.epidem.2018.10.001

Baffour Awuah, G., Tanaka, L. F., Eberl, M., Donnachie, E., Schauberger, G., Lehner, C. T., Himmler, S., Sundmacher, L., & Klug, S. J. (2024). Analysis of health claims data on vaccination coverage in older adults in Bavaria, Germany : Influenza, pneumococcus and herpes zoster. Vaccine, 42(26). https://doi.org/10.1016/j.vaccine.2024.126354

Bagarella, G., Maistrello, M., Minoja, M., Leoni, O., Bortolan, F., Cereda, D., & Corrao, G. (2022). Early Detection of SARS-CoV-2 Epidemic Waves : Lessons from the Syndromic Surveillance in Lombardy, Italy. International Journal of Environmental Research and Public Health, 19(19). https://doi.org/10.3390/ijerph191912375

Bager, P., Svalgaard, I. B., Lomholt, F. K., Emborg, H.-D., Christiansen, L. E., Soborg, B., Hviid, A., & Vestergaard, L. S. (2024). The post-pandemic hospital and mortality burden of COVID-19 compared with influenza: A national cohort study in Denmark, May 2022 to June 2024. medRxiv. https://doi.org/10.1101/2024.09.26.24314428

Bager, P., Svalgaard, I. B., Lomholt, F. K., Emborg, H.-D., Christiansen, L. E., Soborg, B., Hviid, A., & Vestergaard, L. S. (2025). The hospital and mortality burden of COVID-19 compared with influenza in Denmark : A national observational cohort study, 2022-24. The Lancet. Infectious Diseases. https://doi.org/10.1016/S1473-3099(24)00806-5

Bager, P., Wohlfahrt, J., Bhatt, S., Stegger, M., Legarth, R., Møller, C. H., Skov, R. L., Valentiner-Branth, P., Voldstedlund, M., Fischer, T. K., Simonsen, L., Kirkby, N. S., Thomsen, M. K., Spiess, K., Marving, E., Larsen, N. B., Lillebaek, T., Ullum, H., Mølbak, K., … Omicron-Delta study group. (2022). Risk of hospitalisation associated with infection with SARS-CoV-2 omicron variant versus delta variant in Denmark : An observational cohort study. The Lancet. Infectious Diseases, 22(7). https://doi.org/10.1016/S1473-3099(22)00154-2

Bager, P., Wohlfahrt, J., Fonager, J., Rasmussen, M., Albertsen, M., Michaelsen, T. Y., Møller, C. H., Ethelberg, S., Legarth, R., Button, M. S. F., Gubbels, S., Voldstedlund, M., Mølbak, K., Skov, R. L., Fomsgaard, A., Krause, T. G., & Danish Covid-19 Genome Consortium. (2021). Risk of hospitalisation associated with infection with SARS-CoV-2 lineage B.1.1.7 in Denmark : An observational cohort study. The Lancet. Infectious Diseases, 21(11). https://doi.org/10.1016/S1473-3099(21)00290-5

Baglivo, F., De Angelis, L., Magrì, M., De Nicola, I., De Vita, E., Lopalco, P. L., Rizzo, C., & Fedele, A. (2024). The impact of COVID-19 vaccination campaign on pediatric vaccine uptake based on parents' attitudes towards mandatory and recommended vaccination in Southern Italy. Vaccine, 42(16). https://doi.org/10.1016/j.vaccine.2024.04.072

Baguelin, M., Camacho, A., Flasche, S., & Edmunds, W. J. (2015). Extending the elderly- and risk-group programme of vaccination against seasonal influenza in England and Wales : A cost-effectiveness study. BMC Medicine, 13. https://doi.org/10.1186/s12916-015-0452-y

Baguelin, M., Hoschler, K., Stanford, E., Waight, P., Hardelid, P., Andrews, N., & Miller, E. (2011). Age-specific incidence of A/H1N1 2009 influenza infection in England from sequential antibody prevalence data using likelihood-based estimation. PloS One, 6(2). https://doi.org/10.1371/journal.pone.0017074

Baguelin, M., Jit, M., Miller, E., & Edmunds, W. J. (2012). Health and economic impact of the seasonal influenza vaccination programme in England. Vaccine, 30(23). https://doi.org/10.1016/j.vaccine.2012.03.019

Baguelin, M., Van Hoek, A., Jit, M., Flasche, S., White, P., & Edmunds, W. (2010). Vaccination against pandemic influenza A/H1N1v in England: A real-time economic evaluation. VACCINE, 28(12), 2370–2384. https://doi.org/10.1016/j.vaccine.2010.01.002

Bai, T., Zhu, X., Zhou, X., Grathwohl, D., Yang, P., Zha, Y., Jin, Y., Chong, H., Yu, Q., Isberner, N., Wang, D., Zhang, L., Kortüm, K. M., Song, J., Rasche, L., Einsele, H., Ning, K., & Hou, X. (2021). Reliable and Interpretable Mortality Prediction With Strong Foresight in COVID-19 Patients : An International Study From China and Germany. Frontiers in Artificial Intelligence, 4. https://doi.org/10.3389/frai.2021.672050

Bajaj, S., Chen, S., Creswell, R., Naidoo, R., Tsui, J. L.-H., Kolade, O., Nicholson, G., Lehmann, B., Hay, J. A., Kraemer, M. U. G., Aguas, R., Donnelly, C. A., Fowler, T., Hopkins, S., Cantrell, L., Dahal, P., White, L. J., Stepniewska, K., Voysey, M., … EY-Oxford Health Analytics Consortium. (2024). COVID-19 testing and reporting behaviours in England across different sociodemographic groups : A population-based study using testing data and data from community prevalence surveillance surveys. The Lancet. Digital Health, 6(11). https://doi.org/10.1016/S2589-7500(24)00169-9

Bajos, N., Counil, E., Franck, J., Jusot, F., Pailhé, A., Spire, A., Martin, C., Lydie, N., Slama, R., Meyer, L., Warszawski, J., & EpiCoV Study Grp. (2021). Social inequalities and dynamics of the early COVID-19 epidemic : A prospective cohort study in France. BMJ OPEN, 11(11). https://doi.org/10.1136/bmjopen-2021-052888

Balboni, E., Filippini, T., Rothman, K. J., Costanzini, S., Bellino, S., Pezzotti, P., Brusaferro, S., Ferrari, F., Orsini, N., Teggi, S., & Vinceti, M. (2023). The influence of meteorological factors on COVID-19 spread in Italy during the first and second wave. Environmental Research, 228. https://doi.org/10.1016/j.envres.2023.115796

Baldo, V., & Bellone, M. (2022). Budget Impact Analysis of the Adjuvanted Quadrivalent Influenza Vaccine in the Elderly in Italy. FARMECONOMIA-HEALTH ECONOMICS AND THERAPEUTIC PATHWAYS, 23(1). https://doi.org/10.7175/fe.v23i1.1538

Baldovin, T., Amoruso, I., Fonzo, M., Buja, A., Baldo, V., Cocchio, S., & Bertoncello, C. (2021). SARS-CoV-2 RNA detection and persistence in wastewater samples : An experimental network for COVID-19 environmental surveillance in Padua, Veneto Region (NE Italy). The Science of the Total Environment, 760. https://doi.org/10.1016/j.scitotenv.2020.143329

Ballardini, E., Manfrini, M., Fattori, S., Pellacani, E., Ćosić, B., Gargano, G., & Berardi, A. (2025). Hospitalizations for bronchiolitis among infants before and after the SARS-CoV-2 pandemic: An area-based study of the Emilia-Romagna Region, Italy. Italian Journal of Pediatrics, 51(1), 34. https://doi.org/10.1186/s13052-025-01871-6

Ballotari, P., Guarda, L., Giacomazzi, E., Ceruti, A., Gatti, L., & Ricci, P. (2020). [Excess mortality risk in nursing care homes before and during the COVID-19 outbreak in Mantua and Cremona provinces (Lombardy Region, Northern Italy)]. Epidemiologia E Prevenzione, 44(5-6 Suppl 2). https://doi.org/10.19191/EP20.5-6.S2.128

Balzanelli, M., Distratis, P., Catucci, O., Amatulli, F., Cefalo, A., Lazzaro, R., Aityan, K. S., Dalagni, G., Nico, A., De Michele, A., Mazza, E., Tampoia, M., D'Errico, P., Pricolo, G., Prudenzano, A., D'Ettorre, E., Di Stasi, C., Morrone, L. F. P., Nguyen, K. C. D., … Gargiulo Isacco, C. (2021). Clinical and diagnostic findings in COVID-19 patients : An original research from SG Moscati Hospital in Taranto Italy. Journal of Biological Regulators and Homeostatic Agents, 35(1). https://doi.org/10.23812/20-605-A

Bañuelos Gimeno, J., Blanco, A., Díaz, J., Linares, C., López, J. A., Navas, M. A., Sánchez-Martínez, G., Luna, Y., Hervella, B., Belda, F., & Culqui, D. R. (2023). Air pollution and meteorological variables' effects on

COVID-19 first and second waves in Spain. International Journal of Environmental Science and Technology: IJEST, 20(3). https://doi.org/10.1007/s13762-022-04190-z

Barandalla, I., Alvarez, C., Barreiro, P., de Mendoza, C., González-Crespo, R., & Soriano, V. (2021). Impact of scaling up SARS-CoV-2 vaccination on COVID-19 hospitalizations in Spain. International Journal of Infectious Diseases: IJID: Official Publication of the International Society for Infectious Diseases, 112. https://doi.org/10.1016/j.ijid.2021.09.022

Barazzetti, A., Milesi, S., & Negri, A. (2024). Exploring Factors Influencing COVID-19 Vaccine Hesitancy and Refusal : A Study in Italy during the Vaccine Rollout. International Journal of Environmental Research and Public Health, 21(3). https://doi.org/10.3390/ijerph21030331

Barbadoro, P., Recanatini, C., Ponzio, E., Illuminati, D., D'Errico, M. M., & Prospero, E. (2016). Barriers to influenza vaccine uptake in obese people in Italy : Changes 2005-2013. European Journal of Internal Medicine, 34. https://doi.org/10.1016/j.ejim.2016.04.015

Barbarossa, M. V., Fuhrmann, J., Meinke, J. H., Krieg, S., Varma, H. V., Castelletti, N., & Lippert, T. (2020). Modeling the spread of COVID-19 in Germany : Early assessment and possible scenarios. PloS One, 15(9). https://doi.org/10.1371/journal.pone.0238559

Barbas Del Buey, J. F., Íñigo Martínez, J., Gutiérrez Rodríguez, M. Á., Alonso García, M., Sánchez-Gómez, A., Lasheras Carbajo, M. D., Jiménez Bueno, S., Esteban Vasallo, M. D., López Zambrano, M. A., Calvo Rey, C., Sanchez Luna, M., Molina Olivas, M., & Arce Arnáez, M. A. (2024). The effectiveness of nirsevimab in reducing the burden of disease due to respiratory syncytial virus (RSV) infection over time in the Madrid region (Spain) : A prospective population-based cohort study. Frontiers in Public Health, 12. https://doi.org/10.3389/fpubh.2024.1441786

Barbati, F., Moriondo, M., Pisano, L., Calistri, E., Lodi, L., Ricci, S., Giovannini, M., Canessa, C., Indolfi, G., & Azzari, C. (2020). Epidemiology of Respiratory Syncytial Virus-Related Hospitalization Over a 5-Year Period in Italy : Evaluation of Seasonality and Age Distribution Before Vaccine Introduction. Vaccines, 8(1). https://doi.org/10.3390/vaccines8010015

Barbeito, I., Precioso, D., Sierra, M. J., Vegas-Azcárate, S., Fernández Balbuena, S., Vitoriano, B., Goméz-Ullate, D., Cao, R., Monge, S., & Study Group for Non-Pharmaceutical Interventions in Spain. (2023). Effectiveness of non-pharmaceutical interventions in nine fields of activity to decrease SARS-CoV-2 transmission (Spain, September 2020-May 2021). Frontiers in Public Health, 11. https://doi.org/10.3389/fpubh.2023.1061331

Barberán, J., Ramos, M., Villanueva, J., Villares, P., Villareal, M., Vivas, M., Orche, S., Tejera-Gonzalez, M., Menéndez, J. M., Hinojosa, L. T., Almirall, C., Antolin, L., Martinez, Lady, Mendoza, S., Pelaez, A., Segarra-Cañamares, M., Guerrero, J. E., Pelaez, J., & Cardinal-Fernández, P. (2025). Epidemiology of the COVID-19 pneumonia in a group of hospitals from Madrid-Spain during the full period of the State of Alarm HM cohort. Revista Espanola De Quimioterapia: Publicacion Oficial De La Sociedad Espanola De Quimioterapia, 38(2), 97–107. https://doi.org/10.37201/req/110.2024

Barbieri, E., Porcu, G., Donà, D., Cavagnis, S., Cantarutti, L., Scamarcia, A., McGovern, I., Haag, M., Giaquinto, C., & Cantarutti, A. (2023). Epidemiology and Burden of Influenza in Children 0-14 Years Over Ten Consecutive Seasons in Italy. The Pediatric Infectious Disease Journal, 42(12). https://doi.org/10.1097/INF.0000000000004090

Barbieri, E., Trinh, N. T. H., Di Chiara, C., Corrao, G., Boracchini, R., Rosa, E., Liberati, C., Donà, D., Lupattelli, A., Giaquinto, C., & Cantarutti, A. (2025). Impact of Prior SARS-CoV-2 Infection on COVID-19 Vaccine Effectiveness in Children and Adolescents in Norway and Italy. Vaccines, 13(7), 698. https://doi.org/10.3390/vaccines13070698

Barbu, M. G., Thompson, R. J., Thompson, D. C., Cretoiu, D., & Suciu, N. (2020). The Impact of SARS-CoV-2 on the Most Common Comorbidities-A Retrospective Study on 814 COVID-19 Deaths in Romania. Frontiers in Medicine, 7, 567199. https://doi.org/10.3389/fmed.2020.567199

Barnekow, T., Peters, C., Dulon, M., & Nienhaus, A. (2024). Impact of pre-existing conditions on the severity of post-COVID syndrome among workers in healthcare and social services in Germany. Journal of Occupational Medicine and Toxicology (London, England), 19(1). https://doi.org/10.1186/s12995-024-00431-8

Baronio, R., Savaré, L., Ruggiero, J., Crotti, B., Mazza, A., Marseglia, G. L., Dodi, I., Cavalli, C., & Schumacher, R. F. (2021). Impact of Ethnicity on COVID-19 Related Hospitalizations in Children During the First Pandemic Wave in Northern Italy. Frontiers in Pediatrics, 9, 625398. https://doi.org/10.3389/fped.2021.625398

Barron, E., Bakhai, C., Kar, P., Weaver, A., Bradley, D., Ismail, H., Knighton, P., Holman, N., Khunti, K., Sattar, N., Wareham, N. J., Young, B., & Valabhji, J. (2020). Associations of type 1 and type 2 diabetes with COVID-19-related mortality in England : A whole-population study. The Lancet. Diabetes & Endocrinology, 8(10). https://doi.org/10.1016/S2213-8587(20)30272-2

Bartel, A., Grau, J. H., Bitzegeio, J., Werber, D., Linzner, N., Schumacher, V., Garske, S., Liere, K., Hackenbeck, T., Rupp, S. I., Sagebiel, D., Böckelmann, U., & Meixner, M. (2024). Timely Monitoring of SARS-CoV-2 RNA Fragments in Wastewater Shows the Emergence of JN.1 (BA.2.86.1.1, Clade 23I) in Berlin, Germany. Viruses, 16(1). https://doi.org/10.3390/v16010102

Bartig, S., Brücker, H., Butschalowsky, H., Danne, C., Gösswald, A., Gossner, L., Grabka, M., Haller, S., Hess, D., Hey, I., Hoebel, J., Jordan, S., Kubisch, U., Niehues, W., Poethko-Mueller, C., Priem, M., Rother, N., Schaade, L., Rosario, A., … Zinn, S. (2023). Corona Monitoring Nationwide (RKI-SOEP-2) : Seroepidemiological Study on the Spread of SARS-CoV-2 Across Germany. JAHRBUCHER FUR NATIONALOKONOMIE UND STATISTIK, 243(3-4). https://doi.org/10.1515/jbnst-2022-0047

Bartolini, L., Bonvicini, L., Ottone, M., Vicentini, M., Bisaccia, E., Riboldi, B., & Giorgi Rossi, P. (2024). [Differences between Italians and immigrants in COVID-19 vaccination coverage in the Reggio Emilia resident population (Emilia-Romagna Region, Northern Italy)]. Epidemiologia E Prevenzione, 48(4-5). https://doi.org/10.19191/EP24.4-5.S1.120

Bartolomeo, N., Giotta, M., Tafuri, S., & Trerotoli, P. (2022). Impact of Socioeconomic Deprivation on the Local Spread of COVID-19 Cases Mediated by the Effect of Seasons and Restrictive Public Health Measures : A Retrospective Observational Study in Apulia Region, Italy. International Journal of Environmental Research and Public Health, 19(18). https://doi.org/10.3390/ijerph191811410

Basellini, U., & Camarda, C. G. (2022). Explaining regional differences in mortality during the first wave of Covid-19 in Italy. Population Studies, 76(1). https://doi.org/10.1080/00324728.2021.1984551

Basellini, U., Alburez-Gutierrez, D., Del Fava, E., Perrotta, D., Bonetti, M., Camarda, C. G., & Zagheni, E. (2021). Linking excess mortality to mobility data during the first wave of COVID-19 in England and Wales. SSM - Population Health, 14. https://doi.org/10.1016/j.ssmph.2021.100799

Bassetti, M., Parisini, A., Calzi, A., Pallavicini, F. M. B., Cassola, G., Artioli, S., Anselmo, M., Pagano, G., Rezza, G., Viscoli, C., & Ligurian H1N1 Collaborative Group. (2011). Risk factors for severe complications of the novel influenza A (H1N1) : Analysis of patients hospitalized in Italy. Clinical Microbiology and Infection: The Official Publication of the European Society of Clinical Microbiology and Infectious Diseases, 17(2). https://doi.org/10.1111/j.1469-0691.2010.03275.x

Bassi, F., Arbia, G., & Falorsi, P. D. (2021). Observed and estimated prevalence of Covid-19 in Italy : How to estimate the total cases from medical swabs data. The Science of the Total Environment, 764. https://doi.org/10.1016/j.scitotenv.2020.142799

Basso, P., Negro, C., Cegolon, L., & Larese Filon, F. (2022). Risk of Vaccine Breakthrough SARS-CoV-2 Infection and Associated Factors in Healthcare Workers of Trieste Teaching Hospitals (North-Eastern Italy). Viruses, 14(2). https://doi.org/10.3390/v14020336

Bastard, J., Taisne, B., Figoni, J., Mailles, A., Durand, J., Fayad, M., Josset, L., Maisa, A., van der Werf, S., Parent du Châtelet, I., & Bernard-Stoecklin, S. (2022). Impact of the Omicron variant on SARS-CoV-2 reinfections in France, March 2021 to February 2022. Euro Surveillance: Bulletin Europeen Sur Les Maladies

Transmissibles = European Communicable Disease Bulletin, 27(13). https://doi.org/10.2807/1560-7917.ES.2022.27.13.2200247

Bastola, K., Nohynek, H., Lilja, E., Castaneda, A. E., Austero, S., Kuusio, H., & Skogberg, N. (2023). Incidence of SARS-CoV-2 Infection and Factors Associated With Complete COVID-19 Vaccine Uptake Among Migrant Origin Persons in Finland. International Journal of Public Health, 68. https://doi.org/10.3389/ijph.2023.1605547

Batista, A., & da Silva, S. (2022). An Epidemiological Compartmental Model With Automated Parameter Estimation and Forecasting of the Spread of COVID-19 With Analysis of Data From Germany and Brazil. FRONTIERS IN APPLIED MATHEMATICS AND STATISTICS, 8. https://doi.org/10.3389/fams.2022.645614

Bäuerl, C., Randazzo, W., Sánchez, G., Selma-Royo, M., García Verdevio, E., Martínez, L., Parra-Llorca, A., Lerin, C., Fumadó, V., Crovetto, F., Crispi, F., Pérez-Cano, F. J., Rodríguez, G., Ruiz-Redondo, G., Campoy, C., Martínez-Costa, C., Collado, M. C., & MilkCORONA study team. (2022). SARS-CoV-2 RNA and antibody detection in breast milk from a prospective multicentre study in Spain. Archives of Disease in Childhood. Fetal and Neonatal Edition, 107(2). https://doi.org/10.1136/archdischild-2021-322463

Bauernfeind, S., Hitzenbichler, F., Huppertz, G., Zeman, F., Koller, M., Schmidt, B., Plentz, A., Bauswein, M., Mohr, A., & Salzberger, B. (2021). Brief report : Attitudes towards Covid-19 vaccination among hospital employees in a tertiary care university hospital in Germany in December 2020. Infection, 49(6). https://doi.org/10.1007/s15010-021-01622-9

Baum, U., Kulathinal, S., & Auranen, K. (2021). Spotlight influenza : Estimation of influenza vaccine effectiveness in elderly people with assessment of residual confounding by negative control outcomes, Finland, 2012/13 to 2019/20. Euro Surveillance: Bulletin Europeen Sur Les Maladies Transmissibles = European Communicable Disease Bulletin, 26(36). https://doi.org/10.2807/1560-7917.ES.2021.26.36.2100054

Baum, U., Poukka, E., Leino, T., Kilpi, T., Nohynek, H., & Palmu, A. A. (2022). High vaccine effectiveness against severe COVID-19 in the elderly in Finland before and after the emergence of Omicron. BMC Infectious Diseases, 22(1). https://doi.org/10.1186/s12879-022-07814-4

Beaney, T., Clarke, J., Alboksmaty, A., Flott, K., Fowler, A., Benger, J. R., Aylin, P., Elkin, S., Neves, A. L., & Darzi, A. (2021). Population level impact of a pulse oximetry remote monitoring programme on mortality and healthcare utilisation in the people with covid-19 in England : A national analysis using a stepped wedge design. medRxiv, (Beaney T., thomas.beaney@imperial.ac.uk; Clarke J.; Alboksmaty A.; Flott K.; Aylin P.; Neves A.L.; Darzi A.) Patient Safety Translational Research Centre, Institute of Global Health Innovation, Imperial College London, London, United Kingdom. https://doi.org/10.1101/2021.11.29.21266847

Beaney, T., Neves, A. L., Alboksmaty, A., Ashrafian, H., Flott, K., Fowler, A., Benger, J. R., Aylin, P., Elkin, S., Darzi, A., & Clarke, J. (2022). Trends and associated factors for Covid-19 hospitalisation and fatality risk in 2.3 million adults in England. Nature Communications, 13(1). https://doi.org/10.1038/s41467-022-29880-7

Beca-Martínez, M. T., Ayala, A., Falcón-Romero, M., Rodríguez-Blázquez, C., Benito-Llanes, A., Forjaz, M. J., & Romay-Barja, M. (2024). Characteristics of adults who reported not having had COVID-19 in Spain after the first two years of the pandemic and associated factors. Journal of Infection and Public Health, 17(3), 435–442. https://doi.org/10.1016/j.jiph.2023.12.024

Beca-Martínez, M. T., Romay-Barja, M., Ayala, A., Falcon-Romero, M., Rodríguez-Blázquez, C., Benito, A., & Forjaz, M. J. (2022). Trends in COVID-19 Vaccine Acceptance in Spain, September 2020–May 2021. American Journal of Public Health, 112(11). https://doi.org/10.2105/AJPH.2022.307039

Beccia, F., Aulino, G., Amantea, C., Lontano, A., Altamura, G., Marziali, E., Rossi, M. F., Pascucci, D., Santoro, P. E., Oliva, A., Capelli, G., Federico, B., Damiani, G., & Laurenti, P. (2022). Medical Residents' Behaviours toward Compulsory COVID-19 Vaccination in a Tertiary Hospital in Italy. International Journal of Environmental Research and Public Health, 19(23). https://doi.org/10.3390/ijerph192315985

Beccia, F., Lontano, A., Rossi, M. F., Marziali, E., Pascucci, D., Raponi, M., Santoro, P. E., Moscato, U., & Laurenti, P. (2023). Three-year COVID-19 and flu vaccinations among medical residents in a tertiary hospital in Italy : The threat of acceptance decline in seasonal campaigns. Human Vaccines & Immunotherapeutics, 19(2). https://doi.org/10.1080/21645515.2023.2252708

Bednarska, K., Hallmann-Szelińska, E., Kondratiuk, K., Rabczenko, D., & Brydak, L. B. (2016). Molecular Characteristics of Influenza Virus Type B Lineages Circulating in Poland. Advances in Experimental Medicine and Biology, 910. https://doi.org/10.1007/5584_2015_200

Bednarska, K., Nowak, M., Kondratiuk, K., Hallmann-Szelinska, E., Brydak, L., Pokorski, & M. (2015). Incidence of Circulating Antibodies Against Hemagglutinin of Influenza Viruses in the Epidemic Season 2013/2014 in Poland. PULMONARY INFECTION, 857.

Bejan, I., Popescu, C. P., & Ruta, S. M. (2024). Insights into the Risk Factors and Outcomes of Post-COVID-19 Syndrome-Results from a Retrospective, Cross-Sectional Study in Romania. Life (Basel, Switzerland), 14(11), 1519. https://doi.org/10.3390/life14111519

Bekker, R., Uit Het Broek, M., & Koole, G. (2023). Modeling COVID-19 hospital admissions and occupancy in the Netherlands. European Journal of Operational Research, 304(1). https://doi.org/10.1016/j.ejor.2021.12.044

Bella, A., Gesualdo, F., Orsi, A., Arcuri, C., Chironna, M., Loconsole, D., Napoli, C., Orsi, G. B., Manini, I., Montomoli, E., Alfonsi, V., Castrucci, M. R., & Rizzo, C. (2019). Effectiveness of the trivalent MF59 adjuvated influenza vaccine in preventing hospitalization due to influenza B and A(H1N1)pdm09 viruses in the elderly in Italy, 2017—2018 season. Expert Review of Vaccines, 18(6). https://doi.org/10.1080/14760584.2019.1627206

Bellino, S., Bella, A., Puzelli, S., Di Martino, A., Facchini, M., Punzo, O., Pezzotti, P., Castrucci, M. R., & The InfluNet Study Group, null. (2019). Moderate influenza vaccine effectiveness against A(H1N1)pdm09 virus, and low effectiveness against A(H3N2) subtype, 2018/19 season in Italy. Expert Review of Vaccines, 18(11). https://doi.org/10.1080/14760584.2019.1688151

Bellino, S., Piovesan, C., Bella, A., Rizzo, C., Pezzotti, P., & Ramigni, M. (2020). Determinants of vaccination uptake, and influenza vaccine effectiveness in preventing deaths and hospital admissions in the elderly population; Treviso, Italy, 2014/2015-2016/2017 seasons. Human Vaccines & Immunotherapeutics, 16(2). https://doi.org/10.1080/21645515.2019.1661754

Bellino, S., Punzo, O., Rota, M. C., Del Manso, M., Urdiales, A. M., Andrianou, X., Fabiani, M., Boros, S., Vescio, F., Riccardo, F., Bella, A., Filia, A., Rezza, G., Villani, A., Pezzotti, P., & COVID-19 WORKING GROUP. (2020). COVID-19 Disease Severity Risk Factors for Pediatric Patients in Italy. Pediatrics, 146(4). https://doi.org/10.1542/peds.2020-009399

Benavides, E., Gavin, M., Garcia, R., Garcia, S., Pinto, M., Gimenez, R., & Grande, A. (2022). COVID-19 dynamics in Madrid (Spain) : A new convolutional model to find out the missing information during the first three waves. PLOS ONE, 17(12). https://doi.org/10.1371/journal.pone.0279080

Bender, K., Waßer, F., Keller, Y., Pankotsch, U., Held, H.-C., Weidemann, R. R., Kleber, C., Höser, C., & Stehr, S. N. (2023). Burden of hospital admissions and resulting patient interhospital transports during the 2020/2021 SARS-CoV-2 pandemic in Saxony, Germany. Scientific Reports, 13(1), 8407. https://doi.org/10.1038/s41598-023-35406-y

Benedetti, G., Krause, T. G., Schneider, U. V., Lisby, J. G., Voldstedlund, M., Bang, D., Trebbien, R., & Emborg, H.-D. (2021). Spotlight influenza : Influenza surveillance before and after the introduction of point-of-care testing in Denmark, season 2014/15 to 2018/19. Euro Surveillance: Bulletin Europeen Sur Les Maladies Transmissibles = European Communicable Disease Bulletin, 26(37). https://doi.org/10.2807/1560-7917.ES.2021.26.37.2000724

Bennet, R., Hamrin, J., Wirgart, B. Z., Östlund, M. R., Örtqvist, Å., & Eriksson, M. (2016). Influenza epidemiology among hospitalized children in Stockholm, Sweden 1998-2014. Vaccine, 34(28). https://doi.org/10.1016/j.vaccine.2016.04.082

Benny, D., Giacobini, M., Catalano, A., Costa, G., Gnavi, R., & Ricceri, F. (2024). A Multimorbidity Analysis of Hospitalized Patients With COVID-19 in Northwest Italy : Longitudinal Study Using Evolutionary Machine Learning and Health Administrative Data. JMIR Public Health and Surveillance, 10. https://doi.org/10.2196/52353

Benussi, A., Pilotto, A., Premi, E., Libri, I., Giunta, M., Agosti, C., Alberici, A., Baldelli, E., Benini, M., Bonacina, S., Brambilla, L., Caratozzolo, S., Cortinovis, M., Costa, A., Cotti Piccinelli, S., Cottini, E., Cristillo,

V., Delrio, I., Filosto, M., … Padovani, A. (2020). Clinical characteristics and outcomes of inpatients with neurologic disease and COVID-19 in Brescia, Lombardy, Italy. Neurology, 95(7). https://doi.org/10.1212/WNL.0000000000009848

Béraud, G., Mosnier, A., Guérin, O., Cugnardey, N., Gillet, S., Haond, J., Simon, S., Berkovitch, Q., Gamblin, P., Lesage, H., & Loubet, P. (2025). Cost-Effectiveness Analysis of Expanding Influenza Vaccination to Adults Aged 50 and Over in France. Infectious Diseases and Therapy, 14(7), 1513–1527. https://doi.org/10.1007/s40121-025-01168-5

Berenguer, J., Ryan, P., Rodríguez-Baño, J., Jarrín, I., Carratalà, J., Pachón, J., Yllescas, M., Arriba, J. R., COVID-19@Spain Study Group, Fundación SEIMC-GESIDA, Hospital General Universitario Gregorio Marañón, Hospital Universitario La Paz, Hospital Infanta Leonor, Complejo Hospitalario Virgen de la Salud, Hospital Universitario Rafael Méndez, Hospital Universitario de Cruces, Hospital de Melilla, Hospital San Eloy de Barakaldo, Hospital Universitario Central de Asturias, … Centro Nacional de Epidemiología. (2020). Characteristics and predictors of death among 4035 consecutively hospitalized patients with COVID-19 in Spain. Clinical Microbiology and Infection: The Official Publication of the European Society of Clinical Microbiology and Infectious Diseases, 26(11). https://doi.org/10.1016/j.cmi.2020.07.024

Bergman, J., Ballin, M., Nordström, A., & Nordström, P. (2021). Risk factors for COVID-19 diagnosis, hospitalization, and subsequent all-cause mortality in Sweden : A nationwide study. European Journal of Epidemiology, 36(3). https://doi.org/10.1007/s10654-021-00732-w

Bergqvist, R., Ahlqvist, V. H., Lundberg, M., Hergens, M.-P., Sundström, J., Bell, M., & Magnusson, C. (2021). HMG-CoA reductase inhibitors and COVID-19 mortality in Stockholm, Sweden : A registry-based cohort study. PLoS Medicine, 18(10). https://doi.org/10.1371/journal.pmed.1003820

Bergström, F., Günther, F., & Britton, T. (2024). A counterfactual analysis quantifying the COVID-19 vaccination impact in Sweden. medRxiv, (Bergström F.; Günther F., fanny.bergstrom@math.su.se; Britton T.) Stockholm University, Stockholm, Sweden. https://doi.org/10.1101/2024.08.22.24312361

Bermejo-Martin, J. F., García-Mateo, N., Motos, A., Resino, S., Tamayo, L., Ryan Murua, P., Bustamante-Munguira, E., Gallego Curto, E., Úbeda-Iglesias, A., de la Torre, M. D. C., Estella, Á., Campos-Fernández, S., Martínez Varela, I., Pérez-García, F., Socias, L., López Messa, J., Vidal-Cortés, P., Sagredo Meneses, V., González-Rivera, M., … CIBERES-UCI-COVID Group. (2023). Effect of viral storm in patients admitted to intensive care units with severe COVID-19 in Spain : A multicentre, prospective, cohort study. The Lancet. Microbe, 4(6). https://doi.org/10.1016/S2666-5247(23)00041-1

Bermingham, C., Morgan, J., Ayoubkhani, D., Glickman, M., Islam, N., Sheikh, A., Sterne, J., Walker, A. S., & Nafilyan, V. (2023). Estimating the Effectiveness of First Dose of COVID-19 Vaccine Against Mortality in England : A Quasi-Experimental Study. American Journal of Epidemiology, 192(2). https://doi.org/10.1093/aje/kwac157

Bermingham, C., Nafilyan, V., Andrews, N., & Gethings, O. (2023). Estimating the effectiveness of COVID-19 vaccination against COVID-19 hospitalisation and death : A cohort study based on the 2021 Census, England. medRxiv, (Bermingham C.; Nafilyan V.; Gethings O., owen.gethings@ons.gov.uk) Health Modelling Hub, Data Analysis for Social Care and Health, Health Analysis and Pandemic Insights, Office for National Statistics, Newport, United Kingdom. https://doi.org/10.1101/2023.06.06.23290982

Bernadou, A., Bouges, S., Catroux, M., Rigaux, J. C., Laland, C., Levêque, N., Noury, U., Larrieu, S., Acef, S., Habold, D., Cazenave-Roblot, F., & Filleul, L. (2021). High impact of COVID-19 outbreak in a nursing home in the Nouvelle-Aquitaine region, France, March to April 2020. BMC Infectious Diseases, 21(1). https://doi.org/10.1186/s12879-021-05890-6

Bernadou, A., Sommen, C., Pivette, M., & Hubert, B. (2023). Estimating the burden of influenza-attributable severe acute respiratory infections on the hospital system in Metropolitan France, 2012-2018. BMC Infectious Diseases, 23(1). https://doi.org/10.1186/s12879-023-08078-2

Bernal, J. L., Sinnathamby, M. A., Elgohari, S., Zhao, H., Obi, C., Coughlan, L., Lampos, V., Simmons, R., Tessier, E., Campbell, H., McDonald, S., Ellis, J., Hughes, H., Smith, G., Joy, M., Tripathy, M., Byford, R., Ferreira, F., de Lusignan, S., … Ramsay, M. (2021). The impact of social and physical distancing measures on

COVID-19 activity in England : Findings from a multi-tiered surveillance system. Euro Surveillance: Bulletin Europeen Sur Les Maladies Transmissibles = European Communicable Disease Bulletin, 26(11). https://doi.org/10.2807/1560-7917.ES.2021.26.11.2001062

Bertaglia, G., & Pareschi, L. (2021). Hyperbolic compartmental models for epidemic spread on networks with uncertain data : Application to the emergence of COVID-19 in Italy. MATHEMATICAL MODELS & METHODS IN APPLIED SCIENCES, 31(12). https://doi.org/10.1142/S0218202521500548

Bertaglia, G., Boscheri, W., Dimarco, G., & Pareschi, L. (2021). Spatial spread of COVID-19 outbreak in Italy using multiscale kinetic transport equations with uncertainty. Mathematical Biosciences and Engineering: MBE, 18(5). https://doi.org/10.3934/mbe.2021350

Bertino, V., Credendino, O., Sorrentino, L., Alinei, P., Avino, D., Bencivenga, M., Coppola, C., Del Prete, M., Di Muro, T., Evangelista, C., Giannattasio, P., Iannuzzi, M., Lus, G., Meo, R., Stellato, D., Iacobellis, F., Romano, L., De Angelis, V., Perrotta, M., & Borrelli, S. (2021). [Acute severe respiratory distress in chronic haemodialytic patients affected by SARS-CoV-2 pneumonia : Prevalence and associated factors. A single-centre experience from Cardarelli Hospital in Naples (Italy)]. Giornale Italiano Di Nefrologia: Organo Ufficiale Della Societa Italiana Di Nefrologia, 38(6). http://www.ncbi.nlm.nih.gov/pubmed/34919797

Bertoldo, G., Pesce, A., Pepe, A., Pelullo, C. P., Di Giuseppe, G., & Collaborative Working Group. (2019). Seasonal influenza : Knowledge, attitude and vaccine uptake among adults with chronic conditions in Italy. PloS One, 14(5). https://doi.org/10.1371/journal.pone.0215978

Bertolotti, M., Betti, M., Giacchero, F., Grasso, C., Franceschetti, G., Carotenuto, M., Odone, A., Pacileo, G., Ferrante, D., & Maconi, A. (2022). Long-Term Survival among Patients Hospitalized for COVID-19 during the First Three Epidemic Waves : An Observational Study in a Northern Italy Hospital. International Journal of Environmental Research and Public Health, 19(22). https://doi.org/10.3390/ijerph192215298

Bertran, M., Amin-Chowdhury, Z., Davies, H. G., Allen, H., Clare, T., Davison, C., Sinnathamby, M., Seghezzo, G., Kall, M., Williams, H., Gent, N., Ramsay, M. E., Ladhani, S. N., & Oligbu, G. (2022). COVID-19 deaths in children and young people in England, March 2020 to December 2021 : An active prospective national surveillance study. PLoS Medicine, 19(11). https://doi.org/10.1371/journal.pmed.1004118

Bertrand, I., Challant, J., Jeulin, H., Hartard, C., Mathieu, L., Lopez, S., Scientific Interest Group Obépine, Schvoerer, E., Courtois, S., & Gantzer, C. (2021). Epidemiological surveillance of SARS-CoV-2 by genome quantification in wastewater applied to a city in the northeast of France : Comparison of ultrafiltration- and protein precipitation-based methods. International Journal of Hygiene and Environmental Health, 233. https://doi.org/10.1016/j.ijheh.2021.113692

Bertuzzi, A. F., Ciccarelli, M., Marrari, A., Gennaro, N., Dipasquale, A., Giordano, L., Cariboni, U., Quagliuolo, V. L., Alloisio, M., & Santoro, A. (2021). Impact of active cancer on COVID-19 survival : A matched-analysis on 557 consecutive patients at an Academic Hospital in Lombardy, Italy. British Journal of Cancer, 125(3). https://doi.org/10.1038/s41416-021-01396-9

Bertuzzi, A. F., Marrari, A., Gennaro, N., Cariboni, U., Ciccarelli, M., Giordano, L., Quagliuolo, V. L., & Santoro, A. (2020). Low Incidence of SARS-CoV-2 in Patients with Solid Tumours on Active Treatment : An Observational Study at a Tertiary Cancer Centre in Lombardy, Italy. Cancers, 12(9). https://doi.org/10.3390/cancers12092352

Bertuzzo, E., Mari, L., Pasetto, D., Miccoli, S., Casagrandi, R., Gatto, M., & Rinaldo, A. (2020). The geography of COVID-19 spread in Italy and implications for the relaxation of confinement measures. Nature Communications, 11(1). https://doi.org/10.1038/s41467-020-18050-2

Bhaskaran, K., Rentsch, C. T., Hickman, G., Hulme, W. J., Schultze, A., Curtis, H. J., Wing, K., Warren-Gash, C., Tomlinson, L., Bates, C. J., Mathur, R., MacKenna, B., Mahalingasivam, V., Wong, A., Walker, A. J., Morton, C. E., Grint, D., Mehrkar, A., Eggo, R. M., … Goldacre, B. (2022). Overall and cause-specific hospitalisation and death after COVID-19 hospitalisation in England : A cohort study using linked primary care, secondary care, and death registration data in the OpenSAFELY platform. PLoS Medicine, 19(1). https://doi.org/10.1371/journal.pmed.1003871

Bianchi, F. P., Stefanizzi, P., Cuscianna, E., Di Lorenzo, A., Migliore, G., Tafuri, S., & Germinario, C. A. (2023). Influenza vaccine coverage in 6months-64 years-old patients affected by chronic diseases : A retrospective cohort study in Italy. Human Vaccines & Immunotherapeutics, 19(1). https://doi.org/10.1080/21645515.2022.2162301

Bianchi, F. P., Stefanizzi, P., Migliore, G., Melpignano, L., Daleno, A., Vimercati, L., Marra, M., Working Group, C. R., & Tafuri, S. (2023). A COVID-19 nosocomial cluster in a university hospital in southern Italy : A social network analysis. Annali Di Igiene: Medicina Preventiva E Di Comunita, 35(1). https://doi.org/10.7416/ai.2022.2519

Bianco, A., Della Polla, G., Angelillo, S., Pelullo, C. P., Licata, F., & Angelillo, I. F. (2022). Parental COVID-19 vaccine hesitancy : A cross-sectional survey in Italy. Expert Review of Vaccines, 21(4). https://doi.org/10.1080/14760584.2022.2023013

Bianconi, V., Mannarino, M. R., Bronzo, P., Marini, E., & Pirro, M. (2020). Time-related changes in sex distribution of COVID-19 incidence proportion in Italy. Heliyon, 6(10). https://doi.org/10.1016/j.heliyon.2020.e05304

Bidoli, E., Toffolutti, F., Del Zotto, S., & Serraino, D. (2022). Risk factors for territorial spreading of SARS-CoV-2 in North-eastern Italy. Scientific Reports, 12(1). https://doi.org/10.1038/s41598-022-05368-8

Bieńkowski, C., Kowalska, J. D., Paciorek, M., Wasilewski, P., Uliczny, P., Garbacz-Łagożna, E., Pihowicz, A., Mrozińska, M., Dyda, T., Makowiecki, M., Puła, J., & Horban, A. (2022). The Clinical Course and Outcomes of Patients Hospitalized Due to COVID-19 during Three Pandemic Waves in Poland: A Single Center Observational Study. Journal of Clinical Medicine, 11(24), 7386. https://doi.org/10.3390/jcm11247386

Bieńkowski, C., Skrzat-Klapaczyńska, A., Firląg-Burkacka, E., Horban, A., & Kowalska, J. D. (2023). The Clinical Effectiveness and Safety of Vaccinations against COVID-19 in HIV-Positive Patients : Data from Observational Study in Poland. Vaccines, 11(3). https://doi.org/10.3390/vaccines11030514

Bilancia, M., Vitale, D., Manca, F., Perchinunno, P., & Santacroce, L. (2023). A dynamic causal modeling of the second outbreak of COVID-19 in Italy. Advances in Statistical Analysis: AStA: A Journal of the German Statistical Society. https://doi.org/10.1007/s10182-023-00469-9

Bingöl, K., Meulman, I., Wassing, K., & van der Meer, I. (2025). Determinants of COVID-19-related hospital and ICU admissions in the region Haaglanden, The Netherlands: A cross-sectional study. BMC PUBLIC HEALTH, 25(1). https://doi.org/10.1186/s12889-025-23364-1

Birlutiu, V., Neamtu, B., & Birlutiu, R.-M. (2024). Identification of Factors Associated with Mortality in the Elderly Population with SARS-CoV-2 Infection: Results from a Longitudinal Observational Study from Romania. Pharmaceuticals (Basel, Switzerland), 17(2), 202. https://doi.org/10.3390/ph17020202

Bizzarri, M., Di Traglia, M., Giuliani, A., Vestri, A., Fedeli, V., & Prestininzi, A. (2020). New statistical RI index allow to better track the dynamics of COVID-19 outbreak in Italy. Scientific Reports, 10(1). https://doi.org/10.1038/s41598-020-79039-x

Björk, J., Bonander, C., Moghaddassi, M., Rasmussen, M., Malmqvist, U., Inghammar, M., & Kahn, F. (2022). COVID-19 vaccine effectiveness against severe disease from the Omicron BA.1 and BA.2 subvariants – surveillance results from southern Sweden, December 2021 to March 2022. medRxiv, (Björk J., jonas.bjork@med.lu.se) Clinical Studies Sweden, Forum South, Skåne University Hospital, Lund, Sweden. https://doi.org/10.1101/2022.04.14.22273896

Björk, J., Bonander, C., Moghaddassi, M., Rasmussen, M., Malmqvist, U., Kahn, F., & Inghammar, M. (2022). Surveillance of COVID-19 vaccine effectiveness—A real-time case-control study in southern Sweden. Epidemiology and Infection, 150. https://doi.org/10.1017/S0950268822000425

Blackburn, R. M., Frampton, D., Smith, C. M., Fragaszy, E. B., Watson, S. J., Ferns, R. B., Binter, Š., Coen, P. G., Grant, P., Shallcross, L. J., Kozlakidis, Z., Pillay, D., Kellam, P., Hué, S., Nastouli, E., Hayward, A. C., & ICONIC group. (2019). Nosocomial transmission of influenza : A retrospective cross-sectional study using next generation sequencing at a hospital in England (2012-2014). Influenza and Other Respiratory Viruses, 13(6). https://doi.org/10.1111/irv.12679

Blanchi, S., Torreggiani, M., Chatrenet, A., Fois, A., Mazé, B., Njandjo, L., Bianco, G., Lepori, N., Pili, A., Michel, P.-A., Sileno, G., Arazzi, M., Esposito, V., Pani, A., Versino, E., Esposito, C., Fessi, H., Cabiddu, G., & Piccoli, G. B. (2021). COVID-19 Vaccine Hesitancy in Patients on Dialysis in Italy and France. Kidney International Reports, 6(11). https://doi.org/10.1016/j.ekir.2021.08.030

Blandi, L., & Signorelli, C. (2025). Influenza Vaccine Uptake and Associated Hospitalization Risk in Older Adults with or Without Dementia: Differences Between at Home-Living and Nursing Home Residents in Lombardy, Italy. Vaccines, 13(5), 489. https://doi.org/10.3390/vaccines13050489

Blank, P. R., Freiburghaus, A. U., Ruf, B. R., Schwenkglenks, M. M., & Szucs, T. D. (2008). Trends in influenza vaccination coverage rates in Germany over six seasons from 2001/02 to 2006/07. Medizinische Klinik (Munich, Germany: 1983), 103(11). https://doi.org/10.1007/s00063-008-1121-0

Blanquart, F., Vieillefond, V., Visseaux, B., Abou Chakra, C. N., Nunes, M. C., Jacques, A., Haim-Boukobza, S., Josset, L., Wehrle, V., Deleglise, G., Duret, T., Rameix-Welti, M. A., Lina, B., Enouf, V., RELAB study group, & Bal, A. (2025). Influenza vaccine effectiveness against detected infection in the community, France, October 2024 to February 2025. Euro Surveillance: Bulletin Europeen Sur Les Maladies Transmissibles = European Communicable Disease Bulletin, 30(7), 2500074. https://doi.org/10.2807/1560-7917.ES.2025.30.7.2500074

Bliek-Bueno, K., Mucherino, S., Poblador-Plou, B., González-Rubio, F., Aza-Pascual-Salcedo, M., Orlando, V., Clerencia-Sierra, M., Ioakeim-Skoufa, I., Coscioni, E., Carmona-Pírez, J., Perrella, A., Trama, U., Prados-Torres, A., Menditto, E., & Gimeno-Miguel, A. (2021). Baseline Drug Treatments as Indicators of Increased Risk of COVID-19 Mortality in Spain and Italy. International Journal of Environmental Research and Public Health, 18(22). https://doi.org/10.3390/ijerph182211786

Bloemen, M., Delang, L., Rector, A., Raymenants, J., Thibaut, J., Pussig, B., Fondu, L., Aertgeerts, B., Van Ranst, M., Van Geet, C., Arnout, J., & Wollants, E. (2022). Detection Of SARS-COV-2 Variants Of Concern In Wastewater Of Leuven, Belgium. medRxiv, (Bloemen M.; Rector A.; Van Ranst M.; Wollants E., elke.wollants@kuleuven.be) KU Leuven, Rega Institute, Department of Microbiology, Immunology and Transplantation, Laboratory of Clinical and Epidemiological Virology, Leuven, Belgium. https://doi.org/10.1101/2022.05.12.22274823

Bloise, F., & Tancioni, M. (2021). Predicting the spread of COVID-19 in Italy using machine learning : Do socio-economic factors matter? Structural Change and Economic Dynamics, 56. https://doi.org/10.1016/j.strueco.2021.01.001

Blomaard, L. C., van der Linden, C. M. J., van der Bol, J. M., Jansen, S. W. M., Polinder-Bos, H. A., Willems, H. C., Festen, J., Barten, D. G., Borgers, A. J., Bos, J. C., van den Bos, F., de Brouwer, E. J. M., van Deudekom, F. J. A., van Dijk, S. C., Emmelot-Vonk, M. H., Geels, R. E. S., van de Glind, E. M. M., de Groot, B., Hempenius, L., … Mooijaart, S. P. (2021). Frailty is associated with in-hospital mortality in older hospitalised COVID-19 patients in the Netherlands : The COVID-OLD study. Age and Ageing, 50(3). https://doi.org/10.1093/ageing/afab018

Blommaert, A., Bilcke, J., Vandendijck, Y., Hanquet, G., Hens, N., & Beutels, P. (2014). Cost-effectiveness of seasonal influenza vaccination in pregnant women, health care workers and persons with underlying illnesses in Belgium. Vaccine, 32(46). https://doi.org/10.1016/j.vaccine.2014.08.085

Blotière, P.-O., Maura, G., Raitanen, J., Pulkki, J., Forma, L., Johnell, K., Aaltonen, M., & Wastesson, J. W. (2024). Long-term care use, hospitalizations and mortality during COVID-19 in Finland and Sweden : A nationwide register-based study in 2020. Scandinavian Journal of Public Health, 52(3). https://doi.org/10.1177/14034948241235730

Blümel, B., Schweiger, B., Dehnert, M., Buda, S., Reuss, A., Czogiel, I., Kamtsiuris, P., Schlaud, M., Poethko-Müller, C., Thamm, M., & Haas, W. (2015). Age-related prevalence of cross-reactive antibodies against influenza A(H3N2) variant virus, Germany, 2003 to 2010. Euro Surveillance: Bulletin Europeen Sur Les Maladies Transmissibles = European Communicable Disease Bulletin, 20(32). http://www.ncbi.nlm.nih.gov/pubmed/26290488

Boateng, J., Oeser, C., Seghezzo, G., Harman, K., Dabrera, G., Webster, H., Hope, R., Thelwall, S., & Lamagni, T. (2024). Using the Emergency Care Data Set for the epidemiological surveillance of Children and Young People aged less than 18 years : A case study of COVID-19 in England 2020-2023. medRxiv, (Boateng J., jacob.boateng@ukhsa.gov.uk; Seghezzo G.; Harman K.; Dabrera G.; Webster H.) COVID-19 Vaccines and Epidemiology Division, UK Health Security Agency, United Kingdom. https://doi.org/10.1101/2024.07.26.24310711

Boccia, A., Tufano, R., Ferrucci, V., Sepe, L., Bianchi, M., Pascarella, S., Zollo, M., & Paolella, G. (2022). SARS-CoV-2 Pandemic Tracing in Italy Highlights Lineages with Mutational Burden in Growing Subsets. International Journal of Molecular Sciences, 23(8), 4155. https://doi.org/10.3390/ijms23084155

Bochenek, B., Jankowski, M., Gruszczynska, M., Jaczewski, A., Ziemianski, M., Pyrc, R., Wyszogrodzki, A., Nykiel, G., Kopaczka, D., Figurski, M., & Pinkas, J. (2022). Weather as a potential cause of regional differences in the dynamics of COVID-19 transmission in Poland : Implications for epidemic forecasting. POLISH ARCHIVES OF INTERNAL MEDICINE-POLSKIE ARCHIWUM MEDYCYNY WEWNETRZNEJ, 132(1). https://doi.org/10.20452/pamw.16110

Bock, D., Andersson, E., & Frisén, M. (2008). Statistical surveillance of epidemics : Peak detection of influenza in Sweden. Biometrical Journal. Biometrische Zeitschrift, 50(1). https://doi.org/10.1002/bimj.200610362

Bocquier, A., Fressard, L., Paraponaris, A., Davin, B., & Verger, P. (2017). Seasonal influenza vaccine uptake among people with disabilities : A nationwide population study of disparities by type of disability and socioeconomic status in France. Preventive Medicine, 101. https://doi.org/10.1016/j.ypmed.2017.05.014

Boddington, N. L., Elgohari, S., Ellis, J., Donati, M., Zambon, M., & Pebody, R. G. (2022). Rapid influenza molecular testing in secondary care and influenza surveillance in England : Any impact? Influenza and Other Respiratory Viruses, 16(5). https://doi.org/10.1111/irv.13001

Boddington, N. L., Mangtani, P., Zhao, H., Verlander, N. Q., Ellis, J., Andrews, N., & Pebody, R. G. (2022). Live-attenuated influenza vaccine effectiveness against hospitalization in children aged 2-6 years, the first three seasons of the childhood influenza vaccination program in England, 2013/14-2015/16. Influenza and Other Respiratory Viruses, 16(5). https://doi.org/10.1111/irv.12990

Boddington, N. L., Verlander, N. Q., & Pebody, R. G. (2017). Developing a system to estimate the severity of influenza infection in England : Findings from a hospital-based surveillance system between 2010/2011 and 2014/2015. Epidemiology and Infection, 145(7). https://doi.org/10.1017/S095026881700005X

Boddington, N. L., Warburton, F., Zhao, H., Andrews, N., Ellis, J., Donati, M., & Pebody, R. G. (2019). Influenza vaccine effectiveness against hospitalisation due to laboratory-confirmed influenza in children in England in the 2015-2016 influenza season—A test-negative case-control study. Epidemiology and Infection, 147. https://doi.org/10.1017/S0950268819000876

Bodea, R., Voidăzan, T. S., Ferencz, L. I., & Ábrám, Z. (2025). Comorbidities and Severe COVID-19 Outcomes: A Retrospective Analysis of Hospitalized Patients in Three Counties in Romania. Microorganisms, 13(4), 787. https://doi.org/10.3390/microorganisms13040787

Bödeker, B., Remschmidt, C., Müters, S., & Wichmann, O. (2015). [Influenza, tetanus, and pertussis vaccination coverage among adults in Germany]. Bundesgesundheitsblatt, Gesundheitsforschung, Gesundheitsschutz, 58(2). https://doi.org/10.1007/s00103-014-2097-y

Bödeker, B., Walter, D., Reiter, S., & Wichmann, O. (2014). Cross-sectional study on factors associated with influenza vaccine uptake and pertussis vaccination status among pregnant women in Germany. Vaccine, 32(33). https://doi.org/10.1016/j.vaccine.2014.06.007

Bodewes, R., de Mutsert, G., van der Klis, F. R. M., Ventresca, M., Wilks, S., Smith, D. J., Koopmans, M., Fouchier, R. a. M., Osterhaus, A. D. M. E., & Rimmelzwaan, G. F. (2011). Prevalence of antibodies against seasonal influenza A and B viruses in children in Netherlands. Clinical and Vaccine Immunology: CVI, 18(3). https://doi.org/10.1128/CVI.00396-10

Boëlle, P.-Y., Delory, T., Maynadier, X., Janssen, C., Piarroux, R., Pichenot, M., Lemaire, X., Baclet, N., Weyrich, P., Melliez, H., Meybeck, A., Lanoix, J.-P., & Robineau, O. (2020). Trajectories of Hospitalization in

COVID-19 Patients : An Observational Study in France. Journal of Clinical Medicine, 9(10). https://doi.org/10.3390/jcm9103148

Boeraș, I., Curtean-Bănăduc, A., Bănăduc, D., & Cioca, G. (2022). Anthropogenic Sewage Water Circuit as Vector for SARS-CoV-2 Viral ARN Transport and Public Health Assessment, Monitoring and Forecasting-Sibiu Metropolitan Area (Transylvania/Romania) Study Case. International Journal of Environmental Research and Public Health, 19(18), 11725. https://doi.org/10.3390/ijerph191811725

Boes, L., Boedeker, B., Schmich, P., Wetzstein, M., Wichmann, O., & Remschmidt, C. (2017). Factors associated with parental acceptance of seasonal influenza vaccination for their children—A telephone survey in the adult population in Germany. Vaccine, 35(30). https://doi.org/10.1016/j.vaccine.2017.05.015

Bogler, O., Raissi, A., Colacci, M., Beaman, A., Biering-Sørensen, T., Cressman, A., Detsky, A., Gosset, A., Lassen, M. H., Kandel, C., Khaykin, Y., Barbosa, D., Shaw, L. L., MacFadden, D. R., Pearson, A., Perkins, B., Rothman, K. J., Skaarup, K. G., Weagle, R., … Fralick, M. (2022). The association between diabetes and mortality among patients hospitalized with COVID-19 : Cohort Study of Hospitalized Adults in Ontario, Canada and Copenhagen, Denmark. medRxiv, (Bogler O.; Colacci M.; Cressman A.; Detsky A.; Gosset A.; Sholzberg M.; Hodzic-Santor B.; Zipursky J.; Quinn K.L.; Fralick M., mike.fralick@mail.utoronto.ca) Department of Medicine, University of Toronto, Temerty Faculty of Medicine, Toronto, ON, Canada. https://doi.org/10.1101/2022.07.04.22276207

Böhm, S., Woudenberg, T., Chen, D., Marosevic, D. V., Böhmer, M. M., Hansen, L., Wallinga, J., Sing, A., & Katz, K. (2021). Epidemiology and transmission characteristics of early COVID-19 cases, 20 January-19 March 2020, in Bavaria, Germany. Epidemiology and Infection, 149. https://doi.org/10.1017/S0950268821000510

Böhmer, M. M. (2014). [Seasonal and pandemic influenza vaccination: Coverage and attitude among private physicians in Germany]. Gesundheitswesen (Bundesverband der Ärzte des Öffentlichen Gesundheitsdienstes (Germany)), 76(1), 44–47. https://doi.org/10.1055/s-0033-1343431

Böhmer, M. M., Walter, D., Falkenhorst, G., Müters, S., Krause, G., & Wichmann, O. (2012). Barriers to pandemic influenza vaccination and uptake of seasonal influenza vaccine in the post-pandemic season in Germany. BMC Public Health, 12, 938. https://doi.org/10.1186/1471-2458-12-938

Böhmer, M. M., Walter, D., Krause, G., Müters, S., Gösswald, A., & Wichmann, O. (2011). Determinants of tetanus and seasonal influenza vaccine uptake in adults living in Germany. Human Vaccines, 7(12). https://doi.org/10.4161/hv.7.12.18130

Böhmer, M. M., Walter, D., Müters, S., Krause, G., & Wichmann, O. (2011). Seasonal influenza vaccine uptake in Germany 2007/2008 and 2008/2009 : Results from a national health update survey. Vaccine, 29(27). https://doi.org/10.1016/j.vaccine.2011.04.039

Boldea, O., Cornea-Madeira, A., & Madeira, J. (2023). Disentangling the effect of measures, variants, and vaccines on SARS-CoV-2 infections in England : A dynamic intensity model. ECONOMETRICS JOURNAL, 26(3). https://doi.org/10.1093/ectj/utad004

Bolla, C., Penpa, S., Sarchi, E., Schimmenti, A., Piceni, G., Bertolotti, M., Panzacchi, S., Mandrioli, D., Chichino, G., & Maconi, A. (2025). Impact of the main pharmacological option for COVID-19 disease progression in hospitalized patients in northern Italy: A single-center retrospective study. Minerva Respiratory Medicine, 64(1), 9–18. https://doi.org/10.23736/S2784-8477.24.02145-4

Bollen, N., Artesi, M., Durkin, K., Hong, S. L., Potter, B., Boujemla, B., Vanmechelen, B., Martí-Carreras, J., Wawina-Bokalanga, T., Meex, C., Bontems, S., Hayette, M.-P., André, E., Maes, P., Bours, V., Baele, G., & Dellicour, S. (2021). Exploiting genomic surveillance to map the spatio-temporal dispersal of SARS-CoV-2 spike mutations in Belgium across 2020. Scientific Reports, 11(1). https://doi.org/10.1038/s41598-021-97667-9

Bolotin, S., Pebody, R., White, P. J., McMenamin, J., Perera, L., Nguyen-Van-Tam, J. S., Barlow, T., & Watson, J. M. (2012). A new sentinel surveillance system for severe influenza in England shows a shift in age distribution of hospitalised cases in the post-pandemic period. PLoS ONE, 7(1). https://doi.org/10.1371/journal.pone.0030279

Bonaccorsi, G., Lorini, C., Porchia, B. R., Niccolai, G., Martino, G., Giannarelli, L., & Santomauro, F. (2013). [Influenza vaccination : Coverage and risk perception among students of the health professions at Florence University, Italy]. Annali Di Igiene: Medicina Preventiva E Di Comunita, 25(3). https://doi.org/10.7416/ai.2013.1920

Bonaccorsi, G., Lorini, C., Santomauro, F., Guarducci, S., Pellegrino, E., Puggelli, F., Balli, M., & Bonanni, P. (2013). Predictive factors associated with the acceptance of pandemic and seasonal influenza vaccination in health care workers and students in Tuscany, Central Italy. Human Vaccines & Immunotherapeutics, 9(12), 2603–2612. https://doi.org/10.4161/hv.26036

Bonaldi, C., Fouillet, A., Sommen, C., Lévy-Bruhl, D., & Paireau, J. (2023). Monitoring the reproductive number of COVID-19 in France : Comparative estimates from three datasets. PloS One, 18(10). https://doi.org/10.1371/journal.pone.0293585

Bonde, J. P. E., Sell, L., Flachs, E. M., Coggon, D., Albin, M., Oude Hengel, K. M., Kolstad, H., Mehlum, I. S., Schlünssen, V., Solovieva, S., Torén, K., Jakobsson, K., Nielsen, C., Nilsson, K., Rylander, L., Petersen, K. U., & Tøttenborg, S. S. (2023). Occupational risk of COVID-19 related hospital admission in Denmark 2020-2021 : A follow-up study. Scandinavian Journal of Work, Environment & Health, 49(1). https://doi.org/10.5271/sjweh.4063

Bondeson, L., Thulin, A., Ny, L., Levin, M., Svensson, J., Lindh, M., & Zhao, Z. (2021). Clinical outcomes in cancer patients with COVID-19 in Sweden. Acta Oncologica (Stockholm, Sweden), 60(12). https://doi.org/10.1080/0284186X.2021.1973679

Bone, A., Guthmann, J.-P., Nicolau, J., & Lévy-Bruhl, D. (2010). Population and risk group uptake of H1N1 influenza vaccine in mainland France 2009-2010 : Results of a national vaccination campaign. Vaccine, 28(51). https://doi.org/10.1016/j.vaccine.2010.09.096

Bonfrate, L., Guida, P., Errico, M., Righetti, G., Giannandrea, G., Lupoli, M., Celani, F., & Mastroianni, F. (2020). Effectiveness of three-month social distancing measuresto control the COVID-19 infection in Italy. Italian Journal of Medicine, 14(SUPPL 2). Embase. https://www.embase.com/search/results?subaction=viewrecord&id=L633719003&from=export

Bongiorno, S. (2024). COVERAGE AND EFFECTIVENESS OF NIRSEVIMAB IN A UNIVERSAL PREVENTION PROGRAMME AGAINST RSV: PRELIMINARY DATA FROM THE VALLE D'AOSTA REGION (ITALY). Medico e Bambino, 43(4). Embase. https://doi.org/10.53126/MEB43239

Bönisch, S., Wegscheider, K., Krause, L., Sehner, S., Wiegel, S., Zapf, A., Moser, S., & Becher, H. (2020). Effects of Coronavirus Disease (COVID-19) Related Contact Restrictions in Germany, March to May 2020, on the Mobility and Relation to Infection Patterns. Frontiers in Public Health, 8. https://doi.org/10.3389/fpubh.2020.568287

Bonmarin, I., Belchior, E., Bergounioux, J., Brun-Buisson, C., Mégarbane, B., Chappert, J. L., Hubert, B., Le Strat, Y., & Lévy-Bruhl, D. (2015). Intensive care unit surveillance of influenza infection in France: The 2009/10 pandemic and the three subsequent seasons. Euro Surveillance: Bulletin Europeen Sur Les Maladies Transmissibles = European Communicable Disease Bulletin, 20(46). https://doi.org/10.2807/1560-7917.ES.2015.20.46.30066

Bonmarin, I., Belchior, E., Le Strat, Y., & Levy-Bruhl, D. (2012). First estimates of influenza vaccine effectiveness among severe influenza cases, France, 2011/12. Euro Surveillance: Bulletin Europeen Sur Les Maladies Transmissibles = European Communicable Disease Bulletin, 17(18). https://doi.org/10.2807/ese.17.18.20163-en

Bonnet, G., Weizman, O., Trimaille, A., Pommier, T., Cellier, J., Geneste, L., Panagides, V., Marsou, W., Deney, A., Attou, S., Delmotte, T., Ribeyrolles, S., Chemaly, P., Karsenty, C., Giordano, G., Gautier, A., Chaumont, C., Guilleminot, P., Sagnard, A., … Critical COVID-19 France Investigators. (2021). Characteristics and outcomes of patients hospitalized for COVID-19 in France : The Critical COVID-19 France (CCF) study. Archives of Cardiovascular Diseases, 114(5). https://doi.org/10.1016/j.acvd.2021.01.003

Bonsignore, M., Hohenstein, S., Kodde, C., Leiner, J., Schwegmann, K., Bollmann, A., Möller, R., Kuhlen, R., & Nachtigall, I. (2022). Burden of hospital-acquired SARS-CoV-2 infections in Germany : Occurrence and outcomes of different variants. The Journal of Hospital Infection, 129. https://doi.org/10.1016/j.jhin.2022.08.004

Bontadi, D., Bergamo, L., Torri, P., Patanè, P., Bertoldi, A., & Lonardi, U. (2020). Effectiveness of the measures aimed at containing Sars-cov-2 virus spreading in work settings : A survey in companies based in the Veneto region of Italy. MEDICINA DEL LAVORO, 111(5). https://doi.org/10.23749/mdl.v111i5.10037

Booton, R. D., MacGregor, L., Vass, L., Looker, K. J., Hyams, C., Bright, P. D., Harding, I., Lazarus, R., Hamilton, F., Lawson, D., Danon, L., Pratt, A., Wood, R., Brooks-Pollock, E., & Turner, K. M. E. (2021). Estimating the COVID-19 epidemic trajectory and hospital capacity requirements in South West England : A mathematical modelling framework. BMJ Open, 11(1). https://doi.org/10.1136/bmjopen-2020-041536

Bordas, A., Soriano-Arandes, A., Subirana, M., Malagrida, R., Reyes-Urueña, J. M., Folch, C., Soler-Palacin, P., Gascón, M., Sunyer, J., Anton, A., Blanco, I., Fernández-Morales, J., Colom-Cadena, A., Sentís, A., Pumarola, T., Basora, J., Casabona, J., & Sentinel School Network Study Group of Catalonia. (2022). Study protocol for monitoring SARS-CoV-2 infection and its determinants in Catalonia (Spain) : An observational and participatory research approach in a Sentinel Network of Schools. BMJ Open, 12(1). https://doi.org/10.1136/bmjopen-2021-055649

Börjesson, M., & Enander, A. (2014). Perceptions and sociodemographic factors influencing vaccination uptake and precautionary behaviours in response to the A/H1N1 influenza in Sweden. Scandinavian Journal of Public Health, 42(2). https://doi.org/10.1177/1403494813510790

Bosch Castells, V., Mira-Iglesias, A., López-Labrador, F. X., Mengual-Chuliá, B., Carballido-Fernández, M., Tortajada-Girbés, M., Mollar-Maseres, J., Puig-Barberà, J., Díez-Domingo, J., & Chaves, S. S. (2024). Pediatric Respiratory Hospitalizations in the Pre-COVID-19 Era : The Contribution of Viral Pathogens and Comorbidities to Clinical Outcomes, Valencia, Spain. Viruses, 16(10). https://doi.org/10.3390/v16101519

Boselli, P. M., & Soriano, J. M. (2023). COVID-19 in Italy : Is the Mortality Analysis a Way to Estimate How the Epidemic Lasts? Biology, 12(4). https://doi.org/10.3390/biology12040584

Bosetti, P., Kiem, C. T., Yazdanpanah, Y., Fontanet, A., Lina, B., Colizza, V., & Cauchemez, S. (2021). Impact of mass testing during an epidemic rebound of SARS-CoV-2 : A modelling study using the example of France. Euro Surveillance: Bulletin Europeen Sur Les Maladies Transmissibles = European Communicable Disease Bulletin, 26(1). https://doi.org/10.2807/1560-7917.ES.2020.26.1.2001978

Bosetti, P., Tran Kiem, C., Andronico, A., Paireau, J., Levy-Bruhl, D., Alter, L., Fontanet, A., & Cauchemez, S. (2022). Impact of booster vaccination on the control of COVID-19 Delta wave in the context of waning immunity : Application to France in the winter 2021/22. Euro Surveillance: Bulletin Europeen Sur Les Maladies Transmissibles = European Communicable Disease Bulletin, 27(1). https://doi.org/10.2807/1560-7917.ES.2022.27.1.2101125

Bosworth, M. L., Ahmed, T., Larsen, T., Lorenzi, L., Morgan, J., Ali, R., Goldblatt, P., Islam, N., Khunti, K., Raleigh, V., Ayoubkhani, D., Bannister, N., Glickman, M., & Nafilyan, V. (2023). Ethnic differences in COVID-19 mortality in the second and third waves of the pandemic in England during the vaccine rollout: A retrospective, population-based cohort study. BMC Medicine, 21(1), 13. https://doi.org/10.1186/s12916-022-02704-7

Bosworth, M. L., Schofield, R., Ayoubkhani, D., Charlton, L., Nafilyan, V., Khunti, K., Zaccardi, F., Gillies, C., Akbari, A., Knight, M., Wood, R., Hardelid, P., Zuccolo, L., & Harrison, C. (2023). Vaccine effectiveness for prevention of covid-19 related hospital admission during pregnancy in England during the alpha and delta variant dominant periods of the SARS-CoV-2 pandemic: Population based cohort study. BMJ Medicine, 2(1), e000403. https://doi.org/10.1136/bmjmed-2022-000403

Botti, C., Maglione, A., Russo, A., Micillo, A., Scognamiglio, G., & Cantile, M. (2021). Detection of SARS-CoV-2 infection in a pediatric population from south Italy without symptoms of Coronavirus Disease 2019. International Journal of Clinical and Experimental Pathology, 14(12). http://www.ncbi.nlm.nih.gov/pubmed/35027996

Bottle, A., Faitna, P., & Aylin, P. P. (2022). Patient-level and hospital-level variation and related time trends in COVID-19 case fatality rates during the first pandemic wave in England: Multilevel modelling analysis of routine data. BMJ Quality & Safety, 31(3), 211–220. https://doi.org/10.1136/bmjqs-2021-012990

Bottle, A., Faitna, P., Brett, S., & Aylin, P. (2022). Factors associated with, and variations in, COVID-19 hospital death rates in England's first two waves : Observational study. BMJ Open, 12(6). https://doi.org/10.1136/bmjopen-2021-060251

Bouckaert, N., Lefèvre, M., Van den Heede, K., & Van de Voorde, C. (2023). RSV Burden and Its Impact on Pediatric Inpatient Bed Occupancy in Belgium : An Analysis of National Hospital Claims Data. The Pediatric Infectious Disease Journal, 42(10). https://doi.org/10.1097/INF.0000000000004038

Bourdin, S., Jeanne, L., Nadou, F., & Noiret, G. (2021). Does lockdown work ? A spatial analysis of the spread and concentration of Covid-19 in Italy. REGIONAL STUDIES, 55(7). https://doi.org/10.1080/00343404.2021.1887471

Bourreau, C., Baron, A., Schwarzinger, M., Alla, F., Cambon, L., Donzel Godinot, L., & CoVaMax Study Group, null. (2022). Determinants of COVID-19 Vaccination Intention among Health Care Workers in France : A Qualitative Study. Vaccines, 10(10). https://doi.org/10.3390/vaccines10101661

Bouwman, M., van Osch, F., Crijns, F., Trienekens, T., Mehagnoul-Schipper, J., van den Bergh, J. P., & de Vries, J. (2021). SARS-CoV-2 seroprevalence in healthcare workers of a teaching hospital in a highly endemic region in the Netherlands after the first wave : A cross-sectional study. BMJ Open, 11(10). https://doi.org/10.1136/bmjopen-2021-051573

Bradács, A., Lorenzovici, L., Bába, L.-I., Kaló, Z., Farkas-Ráduly, S., Precup, A. M., Somodi, K., Gheorghe, M., Calcan, A., Tar, G., Adam, O., Briciu, V. T., Florescu, S. A., Ianoși, E. S., Gârbovan, O., Siriopol, D. C., & Vokó, Z. (2025). Extended Analysis of the Hospitalization Cost and Economic Burden of COVID-19 in Romania. Healthcare (Basel, Switzerland), 13(9), 982. https://doi.org/10.3390/healthcare13090982

Braeye, T., Proesmans, K., Van Cauteren, D., Brondeel, R., Hens, N., Vermeiren, E., Hammami, N., Rosas, A., Taame, A., André, E., & Cuypers, L. (2024). Personal characteristics and transmission dynamics associated with SARS-CoV-2 semi-quantitative PCR test results : An observational study from Belgium, 2021-2022. Frontiers in Public Health, 12. https://doi.org/10.3389/fpubh.2024.1429021

Braeye, T., van Loenhout, J. A. F., Brondeel, R., Stouten, V., Hubin, P., Billuart, M., Chung, P. Y. J., Vandromme, M., Wyndham-Thomas, C., Blot, K., & Catteau, L. (2023). COVID-19 vaccine effectiveness against symptomatic infection and hospitalisation in Belgium, July 2021 to May 2022. Euro Surveillance: Bulletin Europeen Sur Les Maladies Transmissibles = European Communicable Disease Bulletin, 28(26). https://doi.org/10.2807/1560-7917.ES.2023.28.26.2200768

Braeye, T., van Loenhout, J., Brondeel, R., Stouten, V., Hubin, P., Billuart, M., Chung, J., Vandromme, M., Wyndham-Thomas, C., Blot, K., & Catteau, L. (2022). COVID-19 VACCINE EFFECTIVENESS AGAINST SYMPTOMATIC INFECTION AND HOSPITALIZATION IN BELGIUM, JULY 2021-APRIL 2022. medRxiv, (Braeye T., toon.braeye@sciensano.be; van Loenhout J.; Brondeel R.; Stouten V.; Hubin P.; Billuart M.; Chung J.; Vandromme M.; Wyndham-Thomas C.; Blot K.; Catteau L.) Department of Epidemiology and Public Health, Sciensano, Juliette Wytsmansstraat 14, Brussel, Belgium. https://doi.org/10.1101/2022.05.09.22274623

Brągoszewska, E., & Mainka, A. (2022). Impact of Different Air Pollutants (PM10, PM2.5, NO2, and Bacterial Aerosols) on COVID-19 Cases in Gliwice, Southern Poland. International Journal of Environmental Research and Public Health, 19(21). https://doi.org/10.3390/ijerph192114181

Bragstad, K., Emborg, H., Fischer, T. K., Voldstedlund, M., Gubbels, S., Andersen, B., Molbak, K., & Krause, T. (2013). Low vaccine effectiveness against influenza A(H3N2) virus among elderly people in Denmark in 2012/13—A rapid epidemiological and virological assessment. Euro Surveillance: Bulletin Europeen Sur Les Maladies Transmissibles = European Communicable Disease Bulletin, 18(6). http://www.ncbi.nlm.nih.gov/pubmed/23410258

Brainard, J., Lake, I. R., Morbey, R. A., Elliot, A. J., & Hunter, P. R. (2025). Did COVID-19 surveillance system sensitivity change after Omicron? A retrospective observational study in England. BMC Infectious Diseases, 25(1), 770. https://doi.org/10.1186/s12879-025-11120-0

Brainard, J., Rushton, S., Winters, T., & Hunter, P. R. (2022). Spatial Risk Factors for Pillar 1 COVID-19 Excess Cases and Mortality in Rural Eastern England, UK. Risk Analysis: An Official Publication of the Society for Risk Analysis, 42(7). https://doi.org/10.1111/risa.13835

Branda, F. (2022). Impact of the additional/booster dose of COVID-19 vaccine against severe disease during the epidemic phase characterized by the predominance of the Omicron variant in Italy, December 2021—May 2022. medRxiv, (Branda F., francesco.branda@unical.it) University of Calabria, Rende, Italy. https://doi.org/10.1101/2022.04.21.22273567

Brandt, A., Breucker, L., Keller, J., Corman, V. M., Bethke, N., & Seybold, J. (2023). Seropositivity and flight-associated risk factors for SARS-CoV-2 infection among asylum seekers arriving in Berlin, Germany—A cross-sectional study. Frontiers in Public Health, 11. https://doi.org/10.3389/fpubh.2023.1134546

Brandt, F., Simone, G., Loth, J., & Schilling, D. (2024). COVID-19-associated costs and mortality in Germany : An incidence-based analysis from a payer’s perspective. BMC Health Services Research, 24(1). https://doi.org/10.1186/s12913-024-10838-y

Brault, A., Pontais, I., Enouf, V., Debeuret, C., Bloch, E., Paireau, J., Rameix-Welti, M.-A., White, M., Baudemont, G., Lina, B., Parent du Châtelet, I., Casalegno, J.-S., Vaux, S., & Cauchemez, S. (2024). Effect of nirsevimab on hospitalisations for respiratory syncytial virus bronchiolitis in France, 2023-24 : A modelling study. The Lancet. Child & Adolescent Health, 8(10). https://doi.org/10.1016/S2352-4642(24)00143-3

Braun, P., Braun, J., & Woodcock, B. G. (2021). COVID-19 : Effect-modelling of vaccination in Germany with regard to the mutant strain B.1.1.7 and occupancy of ICU facilities. International Journal of Clinical Pharmacology and Therapeutics, 59(7). https://doi.org/10.5414/CP204064

Breen, R., & Ermisch, J. (2021). The distributional impact of Covid-19 : Geographic variation in mortality in England. DEMOGRAPHIC RESEARCH, 44. https://doi.org/10.4054/demres.2021.44.17

Breulmann, M., Kallies, R., Bernhard, K., Gasch, A., Müller, R. A., Harms, H., Chatzinotas, A., & van Afferden, M. (2023). A long-term passive sampling approach for wastewater-based monitoring of SARS-CoV-2 in Leipzig, Germany. The Science of the Total Environment, 887. https://doi.org/10.1016/j.scitotenv.2023.164143

Bricout, H., Levant, M.-C., Assi, N., Crépey, P., Descamps, A., Mari, K., Gaillat, J., Gavazzi, G., Grenier, B., Launay, O., Mosnier, A., Raguideau, F., Watier, L., Harris, R. C., & Chit, A. (2024). The relative effectiveness of a high-dose quadrivalent influenza vaccine versus standard-dose quadrivalent influenza vaccines in older adults in France : A retrospective cohort study during the 2021-2022 influenza season. Clinical Microbiology and Infection: The Official Publication of the European Society of Clinical Microbiology and Infectious Diseases, 30(12). https://doi.org/10.1016/j.cmi.2024.08.012

Bricout, H., Levant, M.-C., Crépey, P., Gavazzi, G., Gaillat, J., Dufournet, M., Assi, N., Grenier, B., Raguideau, F., Péretz, F., Salamand, C., Mosnier, A., Watier, L., Launay, O., & Loiaconol, M. M. (2024). Relative effectiveness of high-dose vs standard-dose influenza vaccines in preventing hospitalizations : A national retrospective cohort study in France, 2022/23 season. medRxiv, (Bricout H., helene.bricout@sanofi.com; Levant M.-C.) Medical Department, Sanofi Vaccines, Lyon, France. https://doi.org/10.1101/2024.10.11.24315085

Brinkmann, F., Diebner, H. H., Matenar, C., Schlegtendal, A., Eitner, L., Timmesfeld, N., Maier, C., & Lücke, T. (2022). Seroconversion rate and socio-economic and ethnic risk factors for SARS-CoV-2 infection in children in a population-based cohort, Germany, June 2020 to February 2021. Euro Surveillance: Bulletin Europeen Sur Les Maladies Transmissibles = European Communicable Disease Bulletin, 27(37). https://doi.org/10.2807/1560-7917.ES.2022.27.37.2101028

Briz-Redón, Á. (2021). The impact of modelling choices on modelling outcomes : A spatio-temporal study of the association between COVID-19 spread and environmental conditions in Catalonia (Spain). Stochastic Environmental Research and Risk Assessment: Research Journal, 35(8). https://doi.org/10.1007/s00477-020-01965-z

Briz-Redón, Á., & Serrano-Aroca, Á. (2020). A spatio-temporal analysis for exploring the effect of temperature on COVID-19 early evolution in Spain. The Science of the Total Environment, 728. https://doi.org/10.1016/j.scitotenv.2020.138811

Briz-Redón, Á., & Serrano-Aroca, Á. (2022). On the association between COVID-19 vaccination levels and incidence and lethality rates at a regional scale in Spain. Stochastic Environmental Research and Risk Assessment: Research Journal, 36(9). https://doi.org/10.1007/s00477-021-02166-y

Briz-Redón, Á., Belenguer-Sapiña, C., & Serrano-Aroca, Á. (2022). A city-level analysis of PM2.5 pollution, climate and COVID-19 early spread in Spain. Journal of Environmental Health Science & Engineering, 20(1). https://doi.org/10.1007/s40201-022-00786-2

Broms, R., Dahlström, C., Najar, J., & Nistotskaya, M. (2024). COVID-19 Mortality and the Structural Characteristics of Long-Term Care Facilities: Evidence from Sweden. PUBLIC PERFORMANCE & MANAGEMENT REVIEW, 47(2), 505–533. https://doi.org/10.1080/15309576.2023.2295282

Brousseau, N., Green, H. K., Andrews, N., Pryse, R., Baguelin, M., Sunderland, A., Ellis, J., & Pebody, R. (2015). Impact of influenza vaccination on respiratory illness rates in children attending private boarding schools in England, 2013-2014 : A cohort study. Epidemiology and Infection, 143(16). https://doi.org/10.1017/S0950268815000667

Brown, C. S., Clare, K., Chand, M., Andrews, J., Auckland, C., Beshir, S., Choudhry, S., Davies, K., Freeman, J., Gallini, A., Moores, R., Patel, T., Poznalska, G., Rodger, A., Roberts, S., Rooney, C., Wilcox, M., Warren, S., Ellis, J., … Hopkins, S. (2020). Snapshot PCR surveillance for SARS-CoV-2 in hospital staff in England. The Journal of Infection, 81(3). https://doi.org/10.1016/j.jinf.2020.06.069

Brown, L., Sutton, K. J., Browne, C., Bartelt-Hofer, J., Greiner, W., Petitjean, A., & Roiz, J. (2024). Cost of illness of the vaccine-preventable diseases influenza, herpes zoster and pneumococcal disease in France. European Journal of Public Health, 34(1). https://doi.org/10.1093/eurpub/ckad212

Brugnano, L., Iavernaro, F., & Zanzottera, P. (2021). A multiregional extension of the SIR model, with application to the COVID-19 spread in Italy. Mathematical Methods in the Applied Sciences, 44(6). https://doi.org/10.1002/mma.7039

Brumboiu, M. I., Iuga, E., Ivanciuc, A., Mutaffof, S., Tudosa, A. S., Gherasimovici, C., & Iaru, I. (2023). Effectiveness and Protection Duration of Anti-COVID-19 Vaccinations among Healthcare Personnel in Cluj-Napoca, Romania. Vaccines, 11(3), 521. https://doi.org/10.3390/vaccines11030521

Bruni, A., Longhini, F., Macheda, S., Biamonte, E., Pasqua, P., Neri, G., Guzzo, M. L., Garofalo, E., & Calabria COVID-ICU Network authors. (2022). Characteristics of unvaccinated and vaccinated critically ill COVID-19 patients in calabria region (Italy) : A retrospective study. Frontiers in Medicine, 9. https://doi.org/10.3389/fmed.2022.1042411

Brunner, F. S., Payne, A., Cairns, E., Airey, G., Gregory, R., Pickwell, N. D., Wilson, M., Carlile, M., Holmes, N., Hill, V., Child, H., Tomlinson, J., Ahmed, S., Denise, H., Rowe, W., Frazer, J., Aerle, R. van, Evens, N., Porter, J., … Paterson, S. (2023). Utility of wastewater genomic surveillance compared to clinical surveillance to track the spread of the SARS-CoV-2 Omicron variant across England. Water Research, 247. https://doi.org/10.1016/j.watres.2023.120804

Brunner, F. S., Payne, A., Cairns, E., Airey, G., Gregory, R., Pickwell, N. D., Wilson, M., Carlile, M., Holmes, N., Hill, V., Child, H., Tomlinson, J., Ahmed, S., Denise, H., Rowe, W., Frazer, J., van Aerle, R., Evens, N., Porter, J., … Paterson, S. (2023). Wastewater genomic surveillance tracks the spread of the SARS-CoV-2 Omicron variant across England. medRxiv, (Brunner F.S.; Cairns E.; Airey G.; Gregory R.; Paterson S., s.paterson@liverpool.ac.uk) Institute of Infection, Veterinary and Ecological Sciences, University of Liverpool, United Kingdom. https://doi.org/10.1101/2023.02.15.23285942

Bruyneel, A., Dauvergne, J. E., Dauby, N., Goffard, J.-C., Rea, A., & Racape, J. (2024). Social health gradient and risk factors among patients hospitalized for COVID-19 and pre-pandemic respiratory infections. A linked national individual case-control study in Belgium. Frontiers in Public Health, 12, 1426898. https://doi.org/10.3389/fpubh.2024.1426898

Brydak, L., Roiz, J., Faivre, P., & Reygrobellet, C. (2012). Implementing an influenza vaccination programme for adults aged ≥65 years in Poland : A cost-effectiveness analysis. Clinical Drug Investigation, 32(2). https://doi.org/10.2165/11594030-000000000-00000

Brzuszek, B., Zielińska, P., Prochoń, A., & Jurczak, W. (2021). COVID-19 vaccine hesitancy among lymphoma patients in Poland. Acta Haematologica Polonica, 52(3). Embase. https://doi.org/10.5603/AHP.2021.0041

Bubenek-Turconi, Ș.-I., Andrei, S., Văleanu, L., Ştefan, M.-G., Grigoraş, I., Copotoiu, S., Bodolea, C., Tomescu, D., Popescu, M., Filipescu, D., Moldovan, H., Rogobete, A.-F., Bălan, C., Moroşanu, B., Săndesc, D., Arafat, R., & COVATI-RO Collaborative. (2023). Clinical characteristics and factors associated with ICU mortality during the first year of the SARS-Cov-2 pandemic in Romania: A prospective, cohort, multicentre study of 9000 patients. European Journal of Anaesthesiology, 40(1), 4–12. https://doi.org/10.1097/EJA.0000000000001776

Buccoliero, G., Romanelli, C., Lonero, G., Loperfido, P., Chimienti, A., & Resta, F. (2010). [Epidemiologic and clinical parameters in hospitalized patients with novel Influenza A (H1N1) in Taranto province, Italy]. Le Infezioni in Medicina, 18(2). http://www.ncbi.nlm.nih.gov/pubmed/20610932

Buchholz, U., Brockmann, S., Duwe, S., Schweiger, B., an der Heiden, M., Reinhardt, B., & Buda, S. (2010). Household transmissibility and other characteristics of seasonal oseltamivir-resistant influenza A(H1N1) viruses, Germany, 2007-8. Euro Surveillance: Bulletin Europeen Sur Les Maladies Transmissibles = European Communicable Disease Bulletin, 15(6). http://www.ncbi.nlm.nih.gov/pubmed/20158979

Bucyibaruta, G., Blangiardo, M., & Konstantinoudis, G. (2022). Community-level characteristics of COVID-19 vaccine hesitancy in England : A nationwide cross-sectional study. European Journal of Epidemiology, 37(10). https://doi.org/10.1007/s10654-022-00905-1

Buda, S., Köpke, K., & Haas, W. (2010). Epidemiological characteristics of the influenza pandemic (H1N1) 2009 in Germany based on the mandatory notification of cases. BUNDESGESUNDHEITSBLATT-GESUNDHEITSFORSCHUNG-GESUNDHEITSSCHUTZ, 53(12), 1223–1230. https://doi.org/10.1007/s00103-010-1158-0

Buda, S., Tolksdorf, K., Schuler, E., Kuhlen, R., & Haas, W. (2017). Establishing an ICD-10 code based SARI-surveillance in Germany—Description of the system and first results from five recent influenza seasons. BMC Public Health, 17(1). https://doi.org/10.1186/s12889-017-4515-1

Budihoi, A. A., Nasui, B. A., Ciuciuc, N., Rosioara, A.-I., Uzarciuc-Coldea, O., Apan, A., Calinici, T., Pop, V., & Popa, M. (2025). COVID-19 Vaccination Acceptance Among the Diabetic Population in the Northwestern Region of Romania: Insights From an Autofill Survey. Cureus, 17(3), e81464. https://doi.org/10.7759/cureus.81464

Buja, A., Paganini, M., Cocchio, S., Scioni, M., Rebba, V., & Baldo, V. (2020). Demographic and socio-economic factors, and healthcare resource indicators associated with the rapid spread of COVID-19 in Northern Italy : An ecological study. PloS One, 15(12). https://doi.org/10.1371/journal.pone.0244535

Buonsenso, D., Ferro, V., Viozzi, F., Morello, R., Proli, F., Bersani, G., Lazzareschi, I., Santangelo, R., Sanguinetti, M., Fiori, B., Zampino, G., & Valentini, P. (2024). Changes in clinical, demographic, and outcome patterns of children hospitalized with non-SARS-CoV-2 viral low respiratory tract infections before and during the COVID pandemic in Rome, Italy. Pediatric Pulmonology, 59(2), 362–370. https://doi.org/10.1002/ppul.26755

Buonsenso, D., Pazukhina, E., Gentili, C., Vetrugno, L., Morello, R., Zona, M., De Matteis, A., D'Ilario, F., Lanni, R., Rongai, T., Del Balzo, P., Fonte, M. T., Valente, M., De Rose, C., Munblit, D., Sigfrid, L., & Valentini, P. (2022). The Prevalence, Characteristics and Risk Factors of Persistent Symptoms in Non-Hospitalized and Hospitalized Children with SARS-CoV-2 Infection Followed-Up for up to 12 Months : A Prospective, Cohort Study in Rome, Italy. Journal of Clinical Medicine, 11(22). https://doi.org/10.3390/jcm11226772

Burström, B., Hemström, Ö., Doheny, M., Agerholm, J., & Liljas, A. (2025). The aftermath of COVID-19: Mortality impact of the pandemic on older persons in Sweden and other Nordic countries, 2020-2023. Scandinavian Journal of Public Health, 53(5), 456–464. https://doi.org/10.1177/14034948241253339

Bustos Sierra, N., Bossuyt, N., Braeye, T., Leroy, M., Moyersoen, I., Peeters, I., Scohy, A., Van der Heyden, J., Van Oyen, H., & Renard, F. (2020). All-cause mortality supports the COVID-19 mortality in Belgium and comparison with major fatal events of the last century. Archives of Public Health = Archives Belges De Sante Publique, 78(1). https://doi.org/10.1186/s13690-020-00496-x

Butkiewicz, S., Zaczyński, A., Pańkowski, I., Tomaka, P., Rzońca, E., Ślęzak, D., Podgórski, M., Gałązkowski, R., Hiczkiewicz, J., & Rzońca, P. (2022). Retrospective Study to Identify Risk Factors for Severe Disease and Mortality Using the Modified Early Warning Score in 5127 Patients with COVID-19 Admitted to an Emergency Department in Poland Between March 2020 and April 2021. Medical Science Monitor: International Medical Journal of Experimental and Clinical Research, 28. https://doi.org/10.12659/MSM.938647

Byttebier, G., Belmans, L., Alexander, M., Saxberg, B. E. H., De Spiegeleer, B., De Spiegeleer, A., Devreker, N., Van Praet, J. T., Vanhove, K., Reybrouck, R., Wynendaele, E., & Fedson, D. S. (2021). Hospital mortality in COVID-19 patients in Belgium treated with statins, ACE inhibitors and/or ARBs. Human Vaccines & Immunotherapeutics, 17(9). https://doi.org/10.1080/21645515.2021.1920271

Cabrera-Álvarez, P., Hornsey, M. J., & Lobera, J. (2022). Determinants of self-reported adherence to COVID-19 regulations in Spain : Social norms, trust and risk perception. Health Promotion International, 37(6). https://doi.org/10.1093/heapro/daac138

Cacha, I., Díaz, J., Castrillo, M., & García, A. (2023). Forecasting COVID-19 spreading through an ensemble of classical and machine learning models : Spain's case study. SCIENTIFIC REPORTS, 13(1). https://doi.org/10.1038/s41598-023-33795-8

Cai, R., Gerlier, L., Eichner, M., Schwehm, M., Rajaram, S., Mould-Quevedo, J., & Lamotte, M. (2021). Cost-effectiveness of the cell-based quadrivalent versus the standard egg-based quadrivalent influenza vaccine in Germany. Journal of Medical Economics, 24(1). https://doi.org/10.1080/13696998.2021.1908000

Caini, S., Paget, J., Spreeuwenberg, P., Korevaar, J. C., Meijer, A., & Hooiveld, M. (2021). Impact of influenza vaccination in the Netherlands, 2007-2016 : Vaccinees consult their general practitioner for clinically diagnosed influenza, acute respiratory infections, and pneumonia more often than non-vaccinees. PloS One, 16(5). https://doi.org/10.1371/journal.pone.0249883

Calderón, A., Pozo, F., Calvo, C., García-García, M., González-Esguevillas, M., Molinero, M., & Casas, I. (2017). Genetic variability of respiratory syncytial virus A in hospitalized children in the last five consecutive winter seasons in Central Spain. Journal of Medical Virology, 89(5). https://doi.org/10.1002/jmv.24703

Calderón-Larrañaga, A., Vetrano, D. L., Rizzuto, D., Bellander, T., Fratiglioni, L., & Dekhtyar, S. (2020). High excess mortality in areas with young and socially vulnerable populations during the COVID-19 outbreak in Stockholm Region, Sweden. BMJ Global Health, 5(10). https://doi.org/10.1136/bmjgh-2020-003595

Callaghan, C. J., Curtis, R. M. K., Mumford, L., Whitaker, H., Pettigrew, G., Gardiner, D., Marson, L., Thorburn, D., White, S., Parmar, J., Ushiro-Lumb, I., Manas, D., Ravanan, R., & NHS Blood and Transplant Organ and Tissue Donation and Transplantation Clinical Team. (2023). Vaccine Effectiveness Against the SARS-CoV-2 B.1.1.529 Omicron Variant in Solid Organ and Islet Transplant Recipients in England : A National Retrospective Cohort Study. Transplantation, 107(5). https://doi.org/10.1097/TP.0000000000004535

Calò, F., Russo, A., Palamone, M., Maggi, P., Allegorico, E., Gentile, I., Sangiovanni, V., Russomando, A., Gentile, V., Calabria, G., Pisapia, R., Megna, A. S., Masullo, A., Iodice, V., Russo, G., Parrella, R., Dell'Aquila, G., Gambardella, M., Ponticiello, A., … Group, O. B. O. C. (2022). Pre-existing chronic kidney disease (CDK) was not associated with a severe clinical outcome of hospitalized COVID-19 : Results of a case-control study in Southern Italy. Le Infezioni in Medicina, 30(4). https://doi.org/10.53854/liim-3004-8

Camacho, J., Giménez, E., Albert, E., Zulaica, J., Álvarez-Rodríguez, B., Torres, I., Rusu, L., Burgos, J. S., Peiró, S., Vanaclocha, H., Limón, R., Alcaraz, M. J., Sánchez-Payá, J., Díez-Domingo, J., Comas, I., Gonzáles-Candelas, F., Geller, R., Navarro, D., Burgos, J. S., … Navarro, D. (2022). Cumulative incidence of SARS-CoV-2 infection in the general population of the Valencian Community (Spain) after the surge of the Omicron BA.1 variant. medRxiv, (Camacho J.; Giménez E.; Albert E.; Torres I.; Alcaraz M.J.; Navarro D., david.navarro@uv.es) Microbiology Service, Clinic University Hospital, INCLIVA Health Research Institute, Valencia, Spain. https://doi.org/10.1101/2022.07.19.22277747

Cámara, A. L., Jiménez-Jorge, S., Méndez, L. S., & De Mateo Ontañón, S. (2010). Surveillance of influenza pandemic (H1N1)2009 in Spain. Revista Espanola de Salud Publica, 84(5), 569–588.

Cambrea, S. C., Halichidis, S., Mihai, C. M., Carp, D. S., Stoicescu, R. M., & Arghir, O. C. (2013). ENVIRONMENTAL RISK FACTORS FOR INFLUENZA AH1N1 AMONG CHILDREN IN SOUTH-EAST ROMANIA. JOURNAL OF ENVIRONMENTAL PROTECTION AND ECOLOGY, 14(3), 1132–1139.

Campa, A., Quattrocchi, M., Guido, M., Gabutti, G., Germinario, C., De Donno, A., & Group, T. I. C. (2010). Ten-year (1999-2009) epidemiological and virological surveillance of influenza in South Italy (Apulia). Influenza Research and Treatment, 2010. https://doi.org/10.1155/2010/642492

Campbell, C. N. J., Mytton, O. T., McLean, E. M., Rutter, P. D., Pebody, R. G., Sachedina, N., White, P. J., Hawkins, C., Evans, B., Waight, P. A., Ellis, J., Bermingham, A., Donaldson, L. J., & Catchpole, M. (2011). Hospitalization in two waves of pandemic influenza A(H1N1) in England. Epidemiology and Infection, 139(10), 1560–1569. https://doi.org/10.1017/S0950268810002657

Campi, G., Mazziotti, M., Valletta, A., Ravagnan, G., Marcelli, A., Perali, A., & Bianconi, A. (2021). Metastable states in plateaus and multi-wave epidemic dynamics of Covid-19 spreading in Italy. SCIENTIFIC REPORTS, 11(1). https://doi.org/10.1038/s41598-021-91950-5

Campi, I., Gennari, L., Merlotti, D., Mingiano, C., Frosali, A., Giovanelli, L., Torlasco, C., Pengo, M. F., Heilbron, F., Soranna, D., Zambon, A., Di Stefano, M., Aresta, C., Bonomi, M., Cangiano, B., Favero, V., Fatti, L., Perego, G. B., Chiodini, I., … Persani, L. (2021). Vitamin D and COVID-19 severity and related mortality : A prospective study in Italy. BMC Infectious Diseases, 21(1). https://doi.org/10.1186/s12879-021-06281-7

Campman, S. L., Boyd, A., Coyer, L., Schinkel, J., Agyemang, C., Galenkamp, H., Koopman, A. D. M., Chilunga, F. P., Schim van der Loeff, M. F., van Houtum, L., Leenstra, T., Stronks, K., & Prins, M. (2024). SARS-CoV-2 vaccination uptake in six ethnic groups living in Amsterdam, the Netherlands : A registry-based study within the HELIUS cohort. Preventive Medicine, 178. https://doi.org/10.1016/j.ypmed.2023.107822

Campman, S. L., van Rossem, G., Boyd, A., Coyer, L., Schinkel, J., Agyemang, C., Galenkamp, H., Koopman, A. D. M., Leenstra, T., Schim van der Loeff, M., Moll van Charante, E. P., van den Born, B.-J. H., Lok, A., Verhoeff, A., Zwinderman, A. H., Jurriaans, S., Stronks, K., & Prins, M. (2023). Intent to vaccinate against SARS-CoV-2 and its determinants across six ethnic groups living in Amsterdam, the Netherlands : A cross-sectional analysis of the HELIUS study. Vaccine, 41(12). https://doi.org/10.1016/j.vaccine.2023.02.030

Candrea, E., Vargha, J. L., Todor, N., Coman, R. T., Baican, A., & Filip, A. (2022). COVID-19 vaccine acceptance among medical students in Romania. Journal of Physiology and Pharmacology: An Official Journal of the Polish Physiological Society, 73(2). https://doi.org/10.26402/jpp.2022.2.14

Canevelli, M., Palmieri, L., Raparelli, V., Lo Noce, C., Colaizzo, E., Tiple, D., Vaianella, L., Vanacore, N., Brusaferro, S., Onder, G., & Italian National Institute of Health COVID-19 Mortality Group*. (2020). Prevalence and clinical correlates of dementia among COVID-19-related deaths in Italy. Alzheimer's & Dementia (Amsterdam, Netherlands), 12(1). https://doi.org/10.1002/dad2.12114

Canevelli, M., Palmieri, L., Raparelli, V., Punzo, O., Donfrancesco, C., Lo Noce, C., Vanacore, N., Brusaferro, S., Onder, G., & Italian National Institute of Health COVID-19 Mortality Group. (2020). COVID-19 mortality among migrants living in Italy. Annali Dell'Istituto Superiore Di Sanita, 56(3). https://doi.org/10.4415/ANN_20_03_16

Canouï-Poitrine, F., Rachas, A., Thomas, M., Carcaillon-Bentata, L., Fontaine, R., Gavazzi, G., Laurent, M., & Robine, J.-M. (2021). Magnitude, change over time, demographic characteristics and geographic distribution of excess deaths among nursing home residents during the first wave of COVID-19 in France : A nationwide cohort study. Age and Ageing, 50(5). https://doi.org/10.1093/ageing/afab098

Cantarutti, A., Barbieri, E., Didonè, F., Scamarcia, A., Giaquinto, C., & Corrao, G. (2022). Influenza Vaccination Effectiveness in Paediatric « Healthy » Patients : A Population-Based Study in Italy. Vaccines, 10(4). https://doi.org/10.3390/vaccines10040582

Cantenys-Molina, S., Fernández-Cruz, E., Francos, P., Lopez Bernaldo de Quirós, J. C., Muñoz, P., & Gil-Herrera, J. (2021). Lymphocyte subsets early predict mortality in a large series of hospitalized COVID-19 patients in Spain. Clinical and Experimental Immunology, 203(3). https://doi.org/10.1111/cei.13547

Cantero Caballero, M., Touma Fernández, A., Granda Martín, M. J., Castuera Gil, A., Zegarra Salas, P., Cuenca Carvajal, C., Cano Ballesteros, J. C., Granado De La Orden, S., Ferrer Civeira, M., Catalán Alonso, P., Pérez Sanz, C., Aguaron De La Cruz, A., Merello Godino, C., Andueza Lillo, J., Rodríguez Pérez, P., & Audibert Mena, L. (2010). [Hospital care of patients with A/H1N1 influenza : Evaluation of the first 1000 reported cases in Spain]. Medicina Clinica, 135(1). https://doi.org/10.1016/j.medcli.2009.10.051

Cantero, M., Millán-Pérez, R., Muñez, E., Gómez-Lozano, N., Anel-Pedroche, J., Parra, L. M., Rodríguez, M. L., & Asensio, A. (2022). SARS-COV-2 seroprevalence among all healthcare workers in a tertiary hospital in Spain. Infectious Diseases (London, England), 54(1). https://doi.org/10.1080/23744235.2021.1963468

Capalbo, C., Bertamino, E., Zerbetto, A., Santino, I., Petrucca, A., Mancini, R., Bonfini, R., Alfonsi, V., Ferracuti, S., Marchetti, P., Simmaco, M., Orsi, G. B., & Napoli, C. (2020). No Evidence of SARS-CoV-2 Circulation in Rome (Italy) during the Pre-Pandemic Period: Results of a Retrospective Surveillance. International Journal of Environmental Research and Public Health, 17(22), 8461. https://doi.org/10.3390/ijerph17228461

Capodici, A., Salussolia, A., La Fauci, G., Di Valerio, Z., Montalti, M., Odone, A., Costantino, C., Larson, H. J., Leask, J., Lenzi, J., Manzoli, L., Gori, D., & On Behalf Of The Obvious Board, null. (2024). Influenza Vaccine Uptake in Italy-The 2022-2023 Seasonal Influenza Vaccination Campaign in Italy : An Update from the OBVIOUS Project. Vaccines, 12(3). https://doi.org/10.3390/vaccines12030297

Capone, A. (2020). Simultaneous circulation of COVID-19 and flu in Italy : Potential combined effects on the risk of death? International Journal of Infectious Diseases: IJID: Official Publication of the International Society for Infectious Diseases, 99. https://doi.org/10.1016/j.ijid.2020.07.077

Capozzi, L., Bianco, A., Del Sambro, L., Simone, D., Lippolis, A., Notarnicola, M., Pesole, G., Pace, L., Galante, D., & Parisi, A. (2021). Genomic Surveillance of Circulating SARS-CoV-2 in South East Italy : A One-Year Retrospective Genetic Study. Viruses, 13(5). https://doi.org/10.3390/v13050731

Capraru, I. D., Marian, C., Vulcanescu, D. D., Tanasescu, S., Dragomir, T. L., Marti, T. D., Boru, C., Avram, C. R., Susan, M., & Vlad, C. S. (2024). Understanding the Impact of COVID-19 on Roma Vulnerable Communities in Western Romania: Insights and Predictive Factors from a Retrospective Study. Viruses, 16(3), 435. https://doi.org/10.3390/v16030435

Caputo, V., Calvino, G., Strafella, C., Termine, A., Fabrizio, C., Trastulli, G., Ingrascì, A., Peconi, C., Bardini, S., Rossini, A., Salvia, A., Borsellino, G., Battistini, L., Caltagirone, C., Cascella, R., & Giardina, E. (2022). Tracking the Initial Diffusion of SARS-CoV-2 Omicron Variant in Italy by RT-PCR and Comparison with Alpha and Delta Variants Spreading. Diagnostics (Basel, Switzerland), 12(2). https://doi.org/10.3390/diagnostics12020467

Caramello, V., Maciotta, A., De Salve, A. V., Gobbi, V., Ruffino, T. M., Mazzetti, G., Ricagni, L., Ling, C., Arione, R., Boccuzzi, A., Costa, G., Sacerdote, C., & Ricceri, F. (2020). Clinical characteristics and management of COVID-19 patients accessing the emergency department in a hospital in Northern Italy in March and April 2020. Epidemiologia E Prevenzione, 44(5-6 Suppl 2). https://doi.org/10.19191/EP20.5-6.S2.120

Caranci, N., Di Girolamo, C., Bartolini, L., Fortuna, D., Berti, E., Sforza, S., Giorgi Rossi, P., & Moro, M. L. (2021). General and COVID-19-Related Mortality by Pre-Existing Chronic Conditions and Care Setting during 2020 in Emilia-Romagna Region, Italy. International Journal of Environmental Research and Public Health, 18(24). https://doi.org/10.3390/ijerph182413224

Cardeñosa, N., Rodés, A., Follia, N., Plasencia, E., Lafuente, S., Arias, C., Torra, R., Minguell, S., Ferrús, G., Barrabeig, I., & Godoy, P. (2011). Epidemiological analysis of severe hospitalized 2009 pandemic influenza A (H1N1) cases in Catalonia, Spain. Human Vaccines, 7, 226–229. https://doi.org/10.4161/hv.7.0.14609

Carletti, M., & Pancrazi, R. (2021). Geographic Negative Correlation of Estimated Incidence between First and Second Waves of Coronavirus Disease 2019 (COVID-19) in Italy. MATHEMATICS, 9(2). https://doi.org/10.3390/math9020133

Carnicer-Pont, D., Fu, M., Castellano, Y., Tigova, O., Driezen, P., Quah, A. C. K., Kaai, S. C., Soriano, J. B., Vardavas, C. I., Fong, G. T., & Fernández, E. (2024). Incidence and Determinants of COVID-19 Among People Who Smoke (2018-2021) : Findings From the ITC EUREST-PLUS Spain Surveys. Archivos De Bronconeumologia, 60(11). https://doi.org/10.1016/j.arbres.2024.05.037

Caro-Martínez, E., Abad-Collado, S., Escrivá-Cerrudo, B., García-Almarza, S., García-Ródenas, M. D. M., Gómez-Merino, E., Serrano-Mateo, M.-I., & Ramos-Rincón, J.-M. (2022). Nosocomial COVID-19 Infection in a Long-Term Hospital in Spain : Retrospective Observational Study. Medicina (Kaunas, Lithuania), 58(5). https://doi.org/10.3390/medicina58050566

Carpagnano, G. E., Buonamico, E., Migliore, G., Resta, E., Di Lecce, V., de Candia, M. L., Solfrizzi, V., Panza, F., & Resta, O. (2021). Bilevel and continuous positive airway pressure and factors linked to all-cause mortality in COVID-19 patients in an intermediate respiratory intensive care unit in Italy. Expert Review of Respiratory Medicine, 15(6). https://doi.org/10.1080/17476348.2021.1866546

Carracedo, Á. (2021). A genome-wide association study of COVID-19 related hospitalization in Spain reveals genetic disparities among sexes. medRxiv, (Carracedo Á., angel.carracedo@usc.es) University of Santiago de Compostela, Spain. https://doi.org/10.1101/2021.11.24.21266741

Carrat, F., de Lamballerie, X., Rahib, D., Blanché, H., Lapidus, N., Artaud, F., Kab, S., Renuy, A., Szabo de Edelenyi, F., Meyer, L., Lydié, N., Charles, M.-A., Ancel, P.-Y., Jusot, F., Rouquette, A., Priet, S., Saba Villarroel, P. M., Fourié, T., Lusivika-Nzinga, C., … for the SAPRIS and SAPRIS-SERO study groups. (2021). Antibody status and cumulative incidence of SARS-CoV-2 infection among adults in three regions of France following the first lockdown and associated risk factors : A multicohort study. International Journal of Epidemiology, 50(5). https://doi.org/10.1093/ije/dyab110

Carstens, G., Kozanli, E., Bulsink, K., McDonald, S., Elahi, M., de Bakker, J., Schipper, M., van Gageldonk-Lafeber, R., van den Hof, S., van Hoek, A. J., & Eggink, D. (2024). Co-infection dynamics of SARS-CoV-2 and respiratory viruses in the 2022/2023 respiratory season in the Netherlands. medRxiv, (Carstens G.; Kozanli E.; Bulsink K.; McDonald S.; Elahi M.; de Bakker J.; Schipper M.; van Gageldonk-Lafeber R.; den Hof S.; Hoek A.J., albert.jan.van.hoek@rivm.nl; Eggink D., dirk.eggink@rivm.nl) Dutch National Institute for Public Health and the Environment (RIVM), Centre for Infectious Disease Control (CIb), Bilthoven, Netherlands. https://doi.org/10.1101/2024.09.10.24313400

Carter, E. C., Hill, H., Solórzano, C., Kerruish, L., McLellan, L., Dodd, J., Smith, A. B., Joseph, A., Lewis, D., Fyles, F., Drysdale, S. B., Gonzalez-Dias, P., Duncan, G. S. J., Davies, K., Saunderson, P., Bangert, M., Kramer, R., Vassilouthis, N., Lesosky, M., … Collins, A. M. (2025). High Respiratory Syncytial Virus Burden in Children Under 3 Years of Age Across All Care Levels in England. medRxiv. https://doi.org/10.1101/2025.07.14.25331490

Carugno, M., Dentali, F., Mathieu, G., Fontanella, A., Mariani, J., Bordini, L., Milani, G. P., Consonni, D., Bonzini, M., Bollati, V., & Pesatori, A. C. (2018). PM10 exposure is associated with increased hospitalizations for respiratory syncytial virus bronchiolitis among infants in Lombardy, Italy. Environmental Research, 166. https://doi.org/10.1016/j.envres.2018.06.016

Carvalho, K., Vicente, J. P., Jakovljevic, M., & Teixeira, J. P. R. (2021). Analysis and Forecasting Incidence, Intensive Care Unit Admissions, and Projected Mortality Attributable to COVID-19 in Portugal, the UK, Germany, Italy, and France : Predictions for 4 Weeks Ahead. Bioengineering (Basel, Switzerland), 8(6). https://doi.org/10.3390/bioengineering8060084

Casabianca, A., Orlandi, C., Amagliani, G., Magnani, M., Brandi, G., & Schiavano, G. F. (2022). SARS-CoV-2 RNA Detection on Environmental Surfaces in a University Setting of Central Italy. International Journal of Environmental Research and Public Health, 19(9). https://doi.org/10.3390/ijerph19095560

Casado, I., García Cenoz, M., Egüés, N., Burgui, C., Martínez-Baz, I., & Castilla, J. (2023). [COVID-19 infections, hospitalizations, and mortality in Navarre (Spain) between February 2020 and September 2022]. Anales Del Sistema Sanitario De Navarra, 46(2). https://doi.org/10.23938/ASSN.1044

Casalegno, J., Ottmann, M., Bouscambert-Duchamp, M., Valette, M., Morfin, F., & Lina, B. (2010). Impact of the 2009 influenza A(H1N1) pandemic wave on the pattern of hibernal respiratory virus epidemics, France, 2009. EUROSURVEILLANCE, 15(6), 5–7.

Casalegno, J.-S., Ploin, D., Cantais, A., Masson, E., Bard, E., Valette, M., Fanget, R., Targe, S. C., Myar-Dury, A.-F., Doret-Dion, M., Massoud, M., Queromes, G., Vanhems, P., Claris, O., Butin, M., Pillet, S., Ader, F., Bin, S., Gaymard, A., … Gillet, Y. (2021). Characteristics of the delayed respiratory syncytial virus epidemic, 2020/2021, Rhône Loire, France. Euro Surveillance: Bulletin Europeen Sur Les Maladies Transmissibles = European Communicable Disease Bulletin, 26(29). https://doi.org/10.2807/1560-7917.ES.2021.26.29.2100630

Casas-Deza, D., Bernal-Monterde, V., Aranda-Alonso, A. N., Montil-Miguel, E., Julián-Gomara, A. B., Letona-Giménez, L., & Arbones-Mainar, J. M. (2021). Age-related mortality in 61,993 confirmed COVID-19 cases over three epidemic waves in Aragon, Spain. Implications for vaccination programmes. PloS One, 16(12). https://doi.org/10.1371/journal.pone.0261061

Casas-Rojo, J. M., Antón-Santos, J. M., Millán-Núñez-Cortés, J., Lumbreras-Bermejo, C., Ramos-Rincón, J. M., Roy-Vallejo, E., Artero-Mora, A., Arnalich-Fernández, F., García-Bruñén, J. M., Vargas-Núñez, J. A., Freire-Castro, S. J., Manzano-Espinosa, L., Perales-Fraile, I., Crestelo-Viéitez, A., Puchades-Gimeno, F., Rodilla-Sala, E., Solís-Marquínez, M. N., Bonet-Tur, D., Fidalgo-Moreno, M. P., … en nombre del Grupo SEMI-COVID-19 Network. (2020). [Clinical characteristics of patients hospitalized with COVID-19 in Spain : Results from the SEMI-COVID-19 Registry]. Revista Clinica Espanola, 220(8). https://doi.org/10.1016/j.rce.2020.07.003

Casas-Rojo, J.-M., Antón-Santos, J.-M., Millán-Núñez-Cortés, J., Gómez-Huelgas, R., Ramos-Rincón, J.-M., Rubio-Rivas, M., Corrales-González, M.-Á., Fernández-Madera-Martínez, M.-R., Beato-Pérez, J.-L., Arnalich-Fernández, F., Gállego-Lezaun, C., Pérez-Martínez, P., Molinos-Castro, S., Tung-Chen, Y., Madrazo, M., Méndez-Bailón, M., Monge-Monge, D., García-García, G.-M., García-Fenoll, R., … SEMI-COVID-19 Network. (2023). Mortality reduction in older COVID-19-patients hospitalized in Spain during the second pandemic wave from the SEMI-COVID-19 Registry. Scientific Reports, 13(1), 17731. https://doi.org/10.1038/s41598-023-42735-5

Cascetta, E., Henke, I., & Di Francesco, L. (2021). The Effects of Air Pollution, Sea Exposure and Altitude on COVID-19 Hospitalization Rates in Italy. International Journal of Environmental Research and Public Health, 18(2). https://doi.org/10.3390/ijerph18020452

Caselli, D., Loconsole, D., Dario, R., Chironna, M., & Aricò, M. (2021). Effectiveness of Preventive Measures in Keeping Low Prevalence of SARS-CoV-2 Infection in Health Care Workers in a Referral Children's Hospital in Southern Italy. Pediatric Reports, 13(1). https://doi.org/10.3390/pediatric13010017

Castaldi, S., Maffeo, M., Rivieccio, B. A., Zignani, M., Manzi, G., Nicolussi, F., Salini, S., Micheletti, A., Gaito, S., & Biganzoli, E. (2020). Monitoring emergency calls and social networks for COVID-19 surveillance. To learn for the future : The outbreak experience of the Lombardia region in Italy. Acta Bio-Medica: Atenei Parmensis, 91(9-S). https://doi.org/10.23750/abm.v91i9-S.10038

Castilla, J., Cia, F., Zubicoa, J., Reina, G., Martínez-Artola, V., & Ezpeleta, C. (2012). Influenza outbreaks in nursing homes with high vaccination coverage in Navarre, Spain, 2011/12. Euro Surveillance: Bulletin Europeen Sur Les Maladies Transmissibles = European Communicable Disease Bulletin, 17(14). http://www.ncbi.nlm.nih.gov/pubmed/22516002

Castilla, J., Etxeberria, J., Ardanaz, E., Floristán, Y., Escudero, R., & Guevara, M. (2010). Estimating the impact of the 2009 influenza A(H1N1) pandemic on mortality in the elderly in Navarre, Spain. EUROSURVEILLANCE, 15(5), 8–12.

Castilla, J., Martínez-Artola, V., Salcedo, E., Martínez-Baz, I., Cenoz, M. G., Guevara, M., Alvarez, N., Irisarri, F., Morán, J., Barricarte, A., & Network for Influenza Surveillance in Hospitals of Navarre. (2012). Vaccine effectiveness in preventing influenza hospitalizations in Navarre, Spain, 2010-2011 : Cohort and case-control study. Vaccine, 30(2). https://doi.org/10.1016/j.vaccine.2011.11.024

Castilla, J., Martínez-Baz, I., Godoy, P., Toledo, D., Astray, J., García, S., Mayoral, J. M., Martín, V., González-Candelas, F., Guevara, M., Diaz-Borrego, J., Torner, N., Baricot, M., Tamames, S., Domínguez, A., & CIBERESP Working Group for the Survey on Influenza Vaccination in Primary Healthcare Professionals. (2013). Trends in influenza vaccine coverage among primary healthcare workers in Spain, 2008-2011. Preventive Medicine, 57(3). https://doi.org/10.1016/j.ypmed.2013.05.021

Castilla, J., Martinez-Baz, I., Martinez-Artola, V., Fernandez-Alonso, M., Reina, G., Guevara, M., Garcia Cenoz, M., Elia, F., Alvarez, N., Barricarte, A., & Ezpeleta, C. (2013). Early estimates of influenza vaccine effectiveness in Navarre, Spain : 2012/13 mid-season analysis. Euro Surveillance: Bulletin Europeen Sur Les Maladies Transmissibles = European Communicable Disease Bulletin, 18(7). http://www.ncbi.nlm.nih.gov/pubmed/23449182

Castilla, J., Martínez-Baz, I., Martínez-Artola, V., Reina, G., Pozo, F., García Cenoz, M., Guevara, M., Morán, J., Irisarri, F., Arriazu, M., Albéniz, E., Ezpeleta, C., Barricarte, A., Primary Health Care Sentinel Network, & Network for Influenza Surveillance in Hospitals of Navarre. (2013). Decline in influenza vaccine effectiveness with time after vaccination, Navarre, Spain, season 2011/12. Euro Surveillance: Bulletin Europeen Sur Les Maladies Transmissibles = European Communicable Disease Bulletin, 18(5). https://doi.org/10.2807/ese.18.05.20388-en

Castilla, J., Martínez-Baz, I., Navascués, A., Casado, I., Aguinaga, A., Díaz-González, J., Delfrade, J., Guevara, M., Ezpeleta, C., Primary Health Care Sentinel Network of Navarre, & Network for Influenza Surveillance in Hospitals of Navarre. (2018). Comparison of influenza vaccine effectiveness in preventing outpatient and inpatient influenza cases in older adults, northern Spain, 2010/11 to 2015/16. Euro Surveillance: Bulletin Europeen Sur Les Maladies Transmissibles = European Communicable Disease Bulletin, 23(2). https://doi.org/10.2807/1560-7917.ES.2018.23.2.16-00780

Castilla, J., Martínez-Baz, I., Navascués, A., Fernandez-Alonso, M., Reina, G., Guevara, M., Chamorro, J., Ortega, M. T., Albéniz, E., Pozo, F., Ezpeleta, C., Primary Health Care Sentinel Network, & Network for Influenza Surveillance in Hospitals of Navarre. (2014). Vaccine effectiveness in preventing laboratory-confirmed influenza in Navarre, Spain : 2013/14 mid-season analysis. Euro Surveillance: Bulletin Europeen Sur Les Maladies Transmissibles = European Communicable Disease Bulletin, 19(6). https://doi.org/10.2807/1560-7917.es2014.19.6.20700

Castilla, J., Morán, J., Martínez-Artola, V., Fernández-Alonso, M., Guevara, M., Cenoz, M. G., Reina, G., Alvarez, N., Arriazu, M., Elía, F., Salcedo, E., Barricarte, A., & Primary Health Care Sentinel Network of Navarre. (2011). Effectiveness of the monovalent influenza A(H1N1)2009 vaccine in Navarre, Spain, 2009-2010 : Cohort and case-control study. Vaccine, 29(35). https://doi.org/10.1016/j.vaccine.2011.06.063

Castilla, J., Moran, J., Martinez-Artola, V., Reina, G., Martinez-Baz, I., Garcia Cenoz, M., Alvarez, N., Irisarri, F., Arriazu, M., Elia, F., & Salcedo, E. (2011). Effectiveness of trivalent seasonal and monovalent influenza A(H1N1)2009 vaccines in population with major chronic conditions of Navarre, Spain : 2010/11 mid-season analysis. Euro Surveillance: Bulletin Europeen Sur Les Maladies Transmissibles = European Communicable Disease Bulletin, 16(7). http://www.ncbi.nlm.nih.gov/pubmed/21345321

Castilla, J., Navascués, A., Casado, I., Díaz-González, J., Pérez-García, A., Fernandino, L., Martínez-Baz, I., Aguinaga, A., Pozo, F., Ezpeleta, C., & Primary Health Care Sentinel Network And The Network For Influenza Surveillance In Hospitals Of Navarre, null. (2017). Combined effectiveness of prior and current season influenza vaccination in northern Spain : 2016/17 mid-season analysis. Euro Surveillance: Bulletin Europeen Sur Les Maladies Transmissibles = European Communicable Disease Bulletin, 22(7). https://doi.org/10.2807/1560-7917.ES.2017.22.7.30465

Castilla, J., Navascués, A., Casado, I., Pérez-García, A., Aguinaga, A., Ezpeleta, G., Pozo, F., Ezpeleta, C., Martínez-Baz, I., Primary Health Care Sentinel Network, & Network for Influenza Surveillance in Hospitals of Navarre. (2018). Interim effectiveness of trivalent influenza vaccine in a season dominated by lineage mismatched influenza B, northern Spain, 2017/18. Euro Surveillance: Bulletin Europeen Sur Les Maladies Transmissibles = European Communicable Disease Bulletin, 23(7). https://doi.org/10.2807/1560-7917.ES.2018.23.7.18-00057

Castilla, J., Navascués, A., Fernández-Alonso, M., Reina, G., Albéniz, E., Pozo, F., Álvarez, N., Martínez-Baz, I., Guevara, M., García-Cenoz, M., Irisarri, F., Casado, I., Ezpeleta, C., & Primary Health Care Sentinel Network and Network for Influenza Surveillance in Hospitals of Navarra. (2016). Effects of previous episodes of influenza and vaccination in preventing laboratory-confirmed influenza in Navarre, Spain, 2013/14 season. Euro Surveillance: Bulletin Europeen Sur Les Maladies Transmissibles = European Communicable Disease Bulletin, 20(22). https://doi.org/10.2807/1560-7917.ES.2016.21.22.30243

Castilla, J., Portillo, M. E., Casado, I., Pozo, F., Navascués, A., Adelantado, M., Gómez Ibáñez, C., Ezpeleta, C., Martínez-Baz, I., & Primary Health Care Sentinel Network and Network for Influenza Surveillance in Hospitals of Navarre. (2020). Effectiveness of the current and prior influenza vaccinations in Northern Spain, 2018-2019. Vaccine, 38(8). https://doi.org/10.1016/j.vaccine.2020.01.028

Castillo, M. S., Khaoua, H., & Courtejoie, N. (2022). Vaccine effectiveness and duration of protection against symptomatic and severe Covid-19 during the first year of vaccination in France. medRxiv, (Castillo M.S., milena.suarez-castillo@insee.fr; Khaoua H.; Courtejoie N.) DREES, Statistics office of the French Ministry for Solidarity and Health, Paris, France. https://doi.org/10.1101/2022.02.17.22270791

Castriotta, L., Rosolen, V., Barbiero, F., Tomietto, M., de Dottori, M., Barbone, F., & Zamaro, G. (2020). [Impact of the COVID-19 epidemic in Friuli Venezia Giulia Region (Northern Italy) : Assessment of factors associated with the risk of death by competing risks analysis]. Epidemiologia E Prevenzione, 44(5-6 Suppl 2). https://doi.org/10.19191/EP20.5-6.S2.111

Català, M., Mercadé-Besora, N., Kolde, R., Trinh, N. T. H., Roel, E., Burn, E., Rathod-Mistry, T., Kostka, K., Man, W. Y., Delmestri, A., Nordeng, H. M. E., Uusküla, A., Duarte-Salles, T., Prieto-Alhambra, D., & Jödicke, A. M. (2024). The effectiveness of COVID-19 vaccines to prevent long COVID symptoms : Staggered cohort study of data from the UK, Spain, and Estonia. The Lancet. Respiratory Medicine, 12(3). https://doi.org/10.1016/S2213-2600(23)00414-9

Catelan, D., Biggeri, A., Russo, F., Gregori, D., Pitter, G., Da Re, F., Fletcher, T., & Canova, C. (2021). Exposure to Perfluoroalkyl Substances and Mortality for COVID-19 : A Spatial Ecological Analysis in the Veneto Region (Italy). International Journal of Environmental Research and Public Health, 18(5). https://doi.org/10.3390/ijerph18052734

Catelli, R., Pelosi, S., Comito, C., Pizzuti, C., & Esposito, M. (2023). Lexicon-based sentiment analysis to detect opinions and attitude towards COVID-19 vaccines on Twitter in Italy. Computers in Biology and Medicine, 158. https://doi.org/10.1016/j.compbiomed.2023.106876

Cattaruzza, E., Radillo, L., Ronchese, F., Negro, C., Rui, F., De Michieli, P., & Larese Filon, F. (2022). COVID-19 susceptibility and vaccination coverage for measles, rubella and mumps in students and healthcare workers in Trieste hospitals (NE Italy). Vaccine: X, 10. https://doi.org/10.1016/j.jvacx.2022.100147

Cavanna, L., Citterio, C., Di Nunzio, C., Biasini, C., Palladino, M. A., Ambroggi, M., Madaro, S., Bidin, L., Porzio, R., & Proietto, M. (2021). Prevalence of COVID-19 Infection in Asymptomatic Cancer Patients in a District With High Prevalence of SARS-CoV-2 in Italy. Cureus, 13(3). https://doi.org/10.7759/cureus.13774

Cavazza, M., Sartirana, M., Wang, Y., & Falk, M. (2023). Assessment of a SARS-CoV-2 population-wide rapid antigen testing in Italy : A modeling and economic analysis study. European Journal of Public Health, 33(5). https://doi.org/10.1093/eurpub/ckad125

Cavillot, L., van Loenhout, J. A. F., Devleesschauwer, B., Wyndham-Thomas, C., Van Oyen, H., Ghattas, J., Blot, K., Van den Borre, L., Billuart, M., Speybroeck, N., De Pauw, R., Stouten, V., Catteau, L., & Hubin, P. (2023). Sociodemographic and socioeconomic disparities in COVID-19 vaccine uptake in Belgium : A nationwide record linkage study. Journal of Epidemiology and Community Health, 78(3). https://doi.org/10.1136/jech-2023-220751

Cawley, C., Bergey, F., Mehl, A., Finckh, A., & Gilsdorf, A. (2021). Novel Methods in the Surveillance of Influenza-Like Illness in Germany Using Data From a Symptom Assessment App (Ada) : Observational Case Study. JMIR Public Health and Surveillance, 7(11). https://doi.org/10.2196/26523

Cazzaniga, M., Testa, S., Brambilla, M., Vergori, A., Viganoni, M., & Montini, G. (2022). Incidence and outcome of SARS-CoV-2 infection in a pediatric kidney transplant recipient cohort from a single center in Northern Italy. Pediatric Transplantation, 26(7). https://doi.org/10.1111/petr.14335

Cazzolla Gatti, R., Velichevskaya, A., Tateo, A., Amoroso, N., & Monaco, A. (2020). Machine learning reveals that prolonged exposure to air pollution is associated with SARS-CoV-2 mortality and infectivity in Italy. Environmental Pollution (Barking, Essex: 1987), 267. https://doi.org/10.1016/j.envpol.2020.115471

Ceccarelli, E., Dorrucci, M., Minelli, G., Jona Lasinio, G., Prati, S., Battaglini, M., Corsetti, G., Bella, A., Boros, S., Petrone, D., Riccardo, F., Maruotti, A., & Pezzotti, P. (2022). Assessing COVID-19-Related Excess Mortality Using Multiple Approaches-Italy, 2020-2021. International Journal of Environmental Research and Public Health, 19(24). https://doi.org/10.3390/ijerph192416998

Cecconi, M., Piovani, D., Brunetta, E., Aghemo, A., Greco, M., Ciccarelli, M., Angelini, C., Voza, A., Omodei, P., Vespa, E., Pugliese, N., Parigi, T. L., Folci, M., Danese, S., & Bonovas, S. (2020). Early Predictors of Clinical Deterioration in a Cohort of 239 Patients Hospitalized for Covid-19 Infection in Lombardy, Italy. Journal of Clinical Medicine, 9(5). https://doi.org/10.3390/jcm9051548

Cederström, A., Mkoma, G. F., Benfield, T., Agyemang, C., Nørredam, M., & Rostila, M. (2025). Long COVID and its risk factors in migrants : A nationwide register study from Sweden. BMC Medicine, 23(1). https://doi.org/10.1186/s12916-025-03900-x

Cegolon, L., Mauro, M., Sansone, D., Tassinari, A., Gobba, F. M., Modenese, A., Casolari, L., Liviero, F., Pavanello, S., Scapellato, M. L., Taus, F., Carta, A., Spiteri, G., Monaco, M. G. L., Porru, S., & Larese Filon, F. (2023). A Multi-Center Study Investigating Long COVID-19 in Healthcare Workers from North-Eastern Italy : Prevalence, Risk Factors and the Impact of Pre-Existing Humoral Immunity-ORCHESTRA Project. Vaccines, 11(12). https://doi.org/10.3390/vaccines11121769

Cegolon, L., Negro, C., Mastrangelo, G., Filon, F. L., & ORCHESTRA working group. (2022). Primary SARS-CoV-2 Infections, Re-infections and Vaccine Effectiveness during the Omicron Transmission Period in Healthcare Workers of Trieste and Gorizia (Northeast Italy), 1 December 2021-31 May 2022. Viruses, 14(12). https://doi.org/10.3390/v14122688

Cegolon, L., Ronchese, F., Ricci, F., Negro, C., & Larese-Filon, F. (2022). SARS-CoV-2 Infection in Health Care Workers of Trieste (North-Eastern Italy), 1 October 2020-7 February 2022 : Occupational Risk and the Impact of the Omicron Variant. Viruses, 14(8). https://doi.org/10.3390/v14081663

Cento, V., Alteri, C., Merli, M., Di Ruscio, F., Tartaglione, L., Rossotti, R., Travi, G., Vecchi, M., Raimondi, A., Nava, A., Colagrossi, L., Fumagalli, R., Ughi, N., Epis, O. M., Fanti, D., Beretta, A., Galbiati, F., Scaglione, F., Vismara, C., … Perno, C. F. (2020). Effectiveness of infection-containment measures on SARS-CoV-2 seroprevalence and circulation from May to July 2020, in Milan, Italy. PloS One, 15(11). https://doi.org/10.1371/journal.pone.0242765

Centrone, F., Loconsole, D., Marziani, A., Orlando, V. A., Delle Fontane, A., Minelli, M., & Chironna, M. (2024). Respiratory Syncytial Virus (RSV) Hospitalizations in the Elderly in a Tertiary Care Hospital in Southern Italy as a Useful Proxy for Targeting Vaccine Preventive Strategies. Infectious Disease Reports, 16(3). https://doi.org/10.3390/idr16030037

Cereda, G., Viscardi, C., & Baccini, M. (2022). Combining and comparing regional SARS-CoV-2 epidemic dynamics in Italy : Bayesian meta-analysis of compartmental models and global sensitivity analysis. FRONTIERS IN PUBLIC HEALTH, 10. https://doi.org/10.3389/fpubh.2022.919456

Cernigliaro, A., Allotta, A. V., & Scondotto, S. (2020). [Can diabetes and its related hypoglycemic drug treatment be considered risk factors for health outcomes in COVID-19 patients ? The results of a study in the population residing in Sicily Region (Southern Italy)]. Epidemiologia E Prevenzione, 44(5-6 Suppl 2). https://doi.org/10.19191/EP20.5-6.S2.132

Cesareo, M., Tagliabue, M., Lopes, M. E., & Moderato, P. (2023). Framing Effects on Willingness and Perceptions towards COVID-19 Vaccination among University Students in Italy : An Exploratory Study. Vaccines, 11(6). https://doi.org/10.3390/vaccines11061079

Cetin, I., Mandalari, M., Cesari, E., Borriello, C. R., Ercolanoni, M., & Preziosi, G. (2022). SARS-CoV-2 Vaccine Uptake during Pregnancy in Regione Lombardia, Italy : A Population-Based Study of 122,942 Pregnant Women. Vaccines, 10(8). https://doi.org/10.3390/vaccines10081369

Ceylan, Z. (2020). Estimation of COVID-19 prevalence in Italy, Spain, and France. The Science of the Total Environment, 729. https://doi.org/10.1016/j.scitotenv.2020.138817

Chadeau-Hyam, M., Eales, O., Bodinier, B., Wang, H., Haw, D., Whitaker, M., Elliott, J., Walters, C. E., Jonnerby, J., Atchison, C., Diggle, P. J., Page, A. J., Ashby, D., Barclay, W., Taylor, G., Cooke, G., Ward, H., Darzi, A., Donnelly, C. A., & Elliott, P. (2022). Breakthrough SARS-CoV-2 infections in double and triple vaccinated adults and single dose vaccine effectiveness among children in Autumn 2021 in England : REACT-1 study. EClinicalMedicine, 48. https://doi.org/10.1016/j.eclinm.2022.101419

Chadeau-Hyam, M., Eales, O., Bodinier, B., Wang, H., Haw, D., Whitaker, M., Walters, C. E., Atchison, C., Diggle, P. J., Page, A. J., Ashby, D., Barclay, W., Taylor, G., Cooke, G., Ward, H., Darzi, A., Donnelly, C. A., & Elliott, P. (2021). REACT-1 round 15 interim report : High and rising prevalence of SARS-CoV-2 infection in England from end of September 2021 followed by a fall in late October 2021. medRxiv, (Chadeau-Hyam M.; Eales O.; Bodinier B.; Wang H.; Haw D.; Whitaker M.; Walters C.E.; Atchison C.; Ashby D.; Ward H.; Donnelly C.A., c.donnelly@imperial.ac.uk; Elliott P., p.elliott@imperial.ac.uk) School of Public Health, Imperial College London, United Kingdom. https://doi.org/10.1101/2021.11.03.21265877

Chadeau-Hyam, M., Wang, H., Eales, O., Haw, D., Bodinier, B., Whitaker, M., Walters, C. E., Ainslie, K. E. C., Atchison, C., Fronterre, C., Diggle, P. J., Page, A. J., Trotter, A. J., Ashby, D., Barclay, W., Taylor, G., Cooke, G., Ward, H., Darzi, A., … COVID-19 Genomics UK consortium. (2022). SARS-CoV-2 infection and vaccine effectiveness in England (REACT-1) : A series of cross-sectional random community surveys. The Lancet. Respiratory Medicine, 10(4). https://doi.org/10.1016/S2213-2600(21)00542-7

Chadsuthi, S., & Modchang, C. (2021). Modelling the effectiveness of intervention strategies to control COVID-19 outbreaks and estimating healthcare demand in Germany. Public Health in Practice (Oxford, England), 2. https://doi.org/10.1016/j.puhip.2021.100121

Chaibakhsh, S., Pourhoseingholi, A., & Vahedi, M. (2020). Global Incidence and Mortality Rate of COVID-19; Special Focus on Iran, Italy and China. Archives of Iranian Medicine, 23(7). https://doi.org/10.34172/aim.2020.42

Chan, D. K. Y., Mclaws, M.-L., & Forsyth, D. R. (2021). COVID-19 in aged care homes : A comparison of effects initial government policies had in the UK (primarily focussing on England) and Australia during the first wave. International Journal for Quality in Health Care: Journal of the International Society for Quality in Health Care, 33(1). https://doi.org/10.1093/intqhc/mzab033

Charaudeau, S., Pakdaman, K., & Boëlle, P.-Y. (2014). Commuter mobility and the spread of infectious diseases : Application to influenza in France. PloS One, 9(1). https://doi.org/10.1371/journal.pone.0083002

Charkiewicz, R., Nikliński, J., Biecek, P., Kiśluk, J., Pancewicz, S., Moniuszko-Malinowska, A. M., Flisiak, R., Krętowski, A. J., Dzięcioł, J., Moniuszko, M., Gierczyński, R., Juszczyk, G., & Reszeć, J. (2021). The first SARS-CoV-2 genetic variants of concern (VOC) in Poland : The concept of a comprehensive approach to monitoring and surveillance of emerging variants. Advances in Medical Sciences, 66(2). https://doi.org/10.1016/j.advms.2021.03.005

Charmet, T., Schaeffer, L., Grant, R., Galmiche, S., Chény, O., Von Platen, C., Maurizot, A., Rogoff, A., Omar, F., David, C., Septfons, A., Cauchemez, S., Gaymard, A., Lina, B., Lefrancois, L. H., Enouf, V., van der Werf, S., Mailles, A., Levy-Bruhl, D., … Fontanet, A. (2021). Impact of original, B.1.1.7, and B.1.351/P.1 SARS-CoV-2 lineages on vaccine effectiveness of two doses of COVID-19 mRNA vaccines : Results from a nationwide case-control study in France. The Lancet Regional Health. Europe, 8. https://doi.org/10.1016/j.lanepe.2021.100171

Chauvel, C., Casalegno, J.-S., Visseaux, B., Vieillefond, V., Haim-Boukozba, S., Enouf, V., Chanard, E., Fabre, M., Rameix-Welti, M.-A., Oblette, A., Giannoli, J.-M., Paireau, J., Josset, L., Lina, B., Gaymard, A., Cauchemez, S., Morfin, F., Nunes, M. C., & Bal, A. (2025). Community and Hospital-Based Laboratory

Surveillance for Influenza, Respiratory Syncytial Virus, and SARS-CoV-2 During the 2023-2024 Season, Lyon, France. Journal of Medical Virology, 97(9), e70549. https://doi.org/10.1002/jmv.70549

Chauvel, C., Horvat, C., Javouhey, E., Gillet, Y., Hassenboehler, J., Chakra, C. N. A., Ragouilliaux, C., Plaisant, F., Ploin, D., Butin, M., Casalegno, J.-S., & Nunes, M. C. (2024). Changes in Respiratory Syncytial Virus-Associated Hospitalisations Epidemiology After Nirsevimab Introduction in Lyon, France. Influenza and Other Respiratory Viruses, 18(12). https://doi.org/10.1111/irv.70054

Chen, D. T.-H., Copland, E., Hirst, J. A., Mi, E., Dixon, S., Coupland, C., & Hippisley-Cox, J. (2024). Uptake, effectiveness and safety of COVID-19 vaccines in individuals at clinical risk due to immunosuppressive drug therapy or transplantation procedures : A population-based cohort study in England. BMC Medicine, 22(1). https://doi.org/10.1186/s12916-024-03457-1

Chen, H., Cao, Y., Feng, L., Zhao, Q., & Torres, J. R. V. (2023). Understanding the spatial heterogeneity of COVID-19 vaccination uptake in England. BMC Public Health, 23(1). https://doi.org/10.1186/s12889-023-15801-w

Chen, Q., Toorop, M. M. A., de Boer, M. G. J., Rosendaal, F. R., & Lijfering, W. M. (2020). Why crowding matters in the time of COVID-19 pandemic? - A lesson from the carnival effect on the 2017/2018 influenza epidemic in the Netherlands. BMC Public Health, 20(1), 1516. https://doi.org/10.1186/s12889-020-09612-6

Chen, S., Creswell, R., Hounsell, R., Cantrell, L., Bajaj, S., Dahal, P., Tsui Lok Hei, J., Kolade, O., Amswych, M., Naidoo, R., Fowler, T., Hopkins, S., Stepniewska, K., Voysey, M., White, L., Shretta, R., Lambert, B., & EY-Oxford Health Analytics Consortium. (2025). Mass testing for discovery and control of COVID-19 outbreaks in adult social care: An observational study and cost-effectiveness analysis of 14 805 care homes in England. BMJ Public Health, 3(1), e001376. https://doi.org/10.1136/bmjph-2024-001376

Chen, Y., Beattie, H., Simpson, A., Nicholls, G., Sandys, V., Keen, C., & Curran, A. D. (2023). A COVID-19 Outbreak in a Large Meat-Processing Plant in England : Transmission Risk Factors and Controls. International Journal of Environmental Research and Public Health, 20(19). https://doi.org/10.3390/ijerph20196806

Cheng, T., Han, B., & Liu, Y. (2023). Exploring public sentiment and vaccination uptake of COVID-19 vaccines in England : A spatiotemporal and sociodemographic analysis of Twitter data. Frontiers in Public Health, 11. https://doi.org/10.3389/fpubh.2023.1193750

Cherri, S., Lemmers, D. H. L., Noventa, S., Abu Hilal, M., & Zaniboni, A. (2020). Outcome of oncological patients admitted with COVID-19 : Experience of a hospital center in northern Italy. Therapeutic Advances in Medical Oncology, 12. https://doi.org/10.1177/1758835920962370

Cherrie, M., Rhodes, S., Wilkinson, J., Mueller, W., Nafilyan, V., Van Tongeren, M., & Pearce, N. (2022). Longitudinal changes in proportionate mortality due to COVID-19 by occupation in England and Wales. Scandinavian Journal of Work, Environment & Health, 48(8). https://doi.org/10.5271/sjweh.4048

Chiatti, C., Di Rosa, M., Barbadoro, P., Lamura, G., Di Stanislao, F., & Prospero, E. (2010). Socioeconomic determinants of influenza vaccination among older adults in Italy. Preventive Medicine, 51(3-4). Embase. https://doi.org/10.1016/j.ypmed.2010.06.008

Chiavenna, C., Presanis, A. M., Charlett, A., de Lusignan, S., Ladhani, S., Pebody, R. G., & De Angelis, D. (2019). Estimating age-stratified influenza-associated invasive pneumococcal disease in England : A time-series model based on population surveillance data. PLoS Medicine, 16(6). https://doi.org/10.1371/journal.pmed.1002829

Chico-Sánchez, P., Gras-Valentí, P., Algado-Sellés, N., Jiménez-Sepúlveda, N., Vanaclocha, H., Peiró, S., Burgos, J. S., Berenguer, A., Navarro, D., Sánchez-Payá, J., & Valencian vaccine research program (ProVaVac) study group. (2022). The effectiveness of mRNA vaccines to prevent SARS-CoV-2 infection and hospitalisation for COVID-19 according to the time elapsed since their administration in health professionals in the Valencian Autonomous Community (Spain). Preventive Medicine, 163. https://doi.org/10.1016/j.ypmed.2022.107237

Chilunga, F. P., Appelman, B., van Vugt, M., Kalverda, K., Smeele, P., van Es, J., Wiersinga, W. J., Rostila, M., Prins, M., Stronks, K., Norredam, M., & Agyemang, C. (2023). Differences in incidence, nature of symptoms, and duration of long COVID among hospitalised migrant and non-migrant patients in the Netherlands : A

retrospective cohort study. The Lancet Regional Health. Europe, 29. https://doi.org/10.1016/j.lanepe.2023.100630

Chirico, G., Ravasio, R., & Sbarigia, U. (2009). Cost-utility analysis of palivizumab in Italy : Results from a simulation model in the prophylaxis of respiratory syncytial virus infection (RSV) among high-risk preterm infants. Italian Journal of Pediatrics, 35(1). https://doi.org/10.1186/1824-7288-35-4

Chirila, S., Hangan, T., Gurgas, L., Costache, M. G., Vlad, M. A., Nitu, B.-F., Bittar, S. M., Craciun, A., Condur, L., & Bjørklund, G. (2024). Pharmacy-Based Influenza Vaccination: A Study of Patient Acceptance in Romania. Risk Management and Healthcare Policy, 17, 1005–1013. https://doi.org/10.2147/RMHP.S459369

Chirizzi, D., Conte, M., Feltracco, M., Dinoi, A., Gregoris, E., Barbaro, E., La Bella, G., Ciccarese, G., La Salandra, G., Gambaro, A., & Contini, D. (2021). SARS-CoV-2 concentrations and virus-laden aerosol size distributions in outdoor air in north and south of Italy. ENVIRONMENT INTERNATIONAL, 146. https://doi.org/10.1016/j.envint.2020.106255

Chiumello, D., Tavelli, A., Serio, L., De Benedittis, S., Pozzi, T., Maj, R., Velati, M., Brusatori, S., D'Albo, R., Zinnato, C., Marchetti, G., Camporota, L., Coppola, S., & D'Arminio Monforte, A. (2023). Differences in clinical characteristics and quantitative lung CT features between vaccinated and not vaccinated hospitalized COVID-19 patients in Italy. Annals of Intensive Care, 13(1). https://doi.org/10.1186/s13613-023-01103-2

Chivu, C.-D., Crăciun, M.-D., Pițigoi, D., Aramă, V., Luminos, M. L., Jugulete, G., Constantin, C., Apostolescu, C. G., & Streinu Cercel, A. (2024). The Dynamic Risk of COVID-19-Related Events in Vaccinated Healthcare Workers (HCWs) from a Tertiary Hospital in Bucharest, Romania: A Study Based on Active Surveillance Data. Vaccines, 12(2), 182. https://doi.org/10.3390/vaccines12020182

Cho, S. (2020). Quantifying the impact of nonpharmaceutical interventions during the COVID-19 outbreak : The case of Sweden. ECONOMETRICS JOURNAL, 23(3). https://doi.org/10.1093/ectj/utaa025

Christensen, D. M., Jørgensen, S. M. B., El-Chouli, M., Phelps, M., Schjerning, A.-M., Sehested, T. S. G., Gerds, T., Sindet-Pedersen, C., Biering-Sørensen, T., Torp-Pedersen, C., Schou, M., & Gislason, G. (2023). Seasonal influenza vaccine uptake among patients with cardiovascular disease in Denmark, 2017-2019. European Heart Journal. Quality of Care & Clinical Outcomes, 9(5). https://doi.org/10.1093/ehjqcco/qcac049

Chudasama, D. Y., Milbourn, H., Nsonwu, O., Senyah, F., Florence, I., Cook, B., Marchant, E., Blomquist, P. B., Flannagan, J., Dabrera, G., Lewis, J., & Lamagni, T. (2022). Penetration and impact of COVID-19 in long term care facilities in England : Population surveillance study. International Journal of Epidemiology, 50(6). https://doi.org/10.1093/ije/dyab176

Cianci, R., Franza, L., Pignataro, G., Massaro, M. G., Rio, P., Tota, A., Ocarino, F., Sacco Fernandez, M., Franceschi, F., Gasbarrini, A., Gambassi, G., & Candelli, M. (2023). Effect of COVID-19 Vaccination on the In-Hospital Prognosis of Patients Admitted during Delta and Omicron Waves in Italy. Vaccines, 11(2). https://doi.org/10.3390/vaccines11020373

Ciardullo, S., Zerbini, F., Perra, S., Muraca, E., Cannistraci, R., Lauriola, M., Grosso, P., Lattuada, G., Ippoliti, G., Mortara, A., Manzoni, G., & Perseghin, G. (2021). Impact of diabetes on COVID-19-related in-hospital mortality : A retrospective study from Northern Italy. Journal of Endocrinological Investigation, 44(4). https://doi.org/10.1007/s40618-020-01382-7

Ciceri, F., Castagna, A., Rovere-Querini, P., De Cobelli, F., Ruggeri, A., Galli, L., Conte, C., De Lorenzo, R., Poli, A., Ambrosio, A., Signorelli, C., Bossi, E., Fazio, M., Tresoldi, C., Colombo, S., Monti, G., Fominskiy, E., Franchini, S., Spessot, M., … Zangrillo, A. (2020). Early predictors of clinical outcomes of COVID-19 outbreak in Milan, Italy. CLINICAL IMMUNOLOGY, 217. https://doi.org/10.1016/j.clim.2020.108509

Cieplucha, H., Zalewska, M., Kujawa, K., & Szetela, B. (2022). Prevalence of Anti-SARS-CoV-2 Antibodies in HIV-Positive Patients in Wroclaw, Poland-Unexpected Difference between First and Second Wave. COVID, 2(12). https://doi.org/10.3390/covid2120125

Cilla, G., Sarasua, A., Montes, M., Arostegui, N., Vicente, D., Pérez-Yarza, E., & Pérez-Trallero, E. (2006). Risk factors for hospitalization due to respiratory syncytial virus infection among infants in the Basque Country, Spain. Epidemiology and Infection, 134(3). https://doi.org/10.1017/S0950268805005571

Cilloniz, C., Motos, A., Pericàs, J. M., Castañeda, T. G., Gabarrús, A., Ferrer, R., García-Gasulla, D., Peñuelas, O., de Gonzalo-Calvo, D., Fernandez-Barat, L., Barbé, F., Torres, A., & CIBERESUCICOVID Project (COV20/00110 ISCIII). (2023). Risk factors associated with mortality among elderly patients with COVID-19 : Data from 55 intensive care units in Spain. Pulmonology, 29(5). https://doi.org/10.1016/j.pulmoe.2023.01.007

Ciofi Degli Atti, M. L., Beccia, F., D'Amore, C., Ravà, L., Bernaschi, P., Russo, C., Villani, A., Perno, C. F., & Raponi, M. (2024). Impact of SARS-CoV-2 Pandemic on Emergency Hospitalizations for Acute Respiratory Infections: The Experience of a Paediatric Tertiary Care Hospital in Italy. Influenza and Other Respiratory Viruses, 18(6), e13335. https://doi.org/10.1111/irv.13335

Ciofi Degli Atti, M., Rizzo, C., D'Amore, C., Ravà, L., Reale, A., Barbieri, M. A., Bernaschi, P., Russo, C., Villani, A., Perno, C. F., & Raponi, M. (2023). Acute respiratory infection emergency access in a tertiary care children hospital in Italy, prior and after the SARS-CoV-2 emergence. Influenza and Other Respiratory Viruses, 17(3). https://doi.org/10.1111/irv.13102

Cipelli, R., Falato, S., Lusito, E., Maifredi, G., Montedoro, M., Valpondi, P., Zucchi, A., Azzi, M. V., Zanetta, L., Gualano, M. R., Xoxi, E., Marchisio, P. G., & Castaldi, S. (2024). The Hospital Burden of Flu in Italy : A retrospective study on administrative data from season 2014-2015 to 2018-2019. BMC Infectious Diseases, 24(1). https://doi.org/10.1186/s12879-024-09446-2

Clifford, S., Waight, P., Hackman, J., Hué, S., Gower, C. M., Kirsebom, F. C. M., Skarnes, C., Letley, L., Bernal, J. L., Andrews, N., Flasche, S., & Miller, E. (2021). Effectiveness of BNT162b2 and ChAdOx1 against SARS-CoV-2 household transmission : A prospective cohort study in England. medRxiv, (Clifford S.; Hackman J.; Hué S.; Flasche S.) Centre for Mathematical Modelling of Infectious Diseases, London School of Hygiene&Tropical Medicine, London, United Kingdom. https://doi.org/10.1101/2021.11.24.21266401

Cobre, A. F., Böger, B., Vilhena, R. O., Fachi, M. M., dos Santos, J. M. M. F., & Tonin, F. S. (2022). A multivariate analysis of risk factors associated with death by Covid-19 in the USA, Italy, Spain, and Germany. Journal of Public Health (Germany), 30(5). Embase. https://doi.org/10.1007/s10389-020-01397-7

Cocco, P., & De Matteis, S. (2022). The determinants of the changing speed of spread of COVID-19 across Italy. Epidemiology and Infection, 150. https://doi.org/10.1017/S095026882200084X

Cocuz, M. E., Cocuz, I.-G., Rodina, L., Filip, R., & Filip, F. (2024). Clinical Outcomes and Characteristics of COVID-19 in Neonates: A Single-Center Study in Romania. Life (Basel, Switzerland), 14(12), 1650. https://doi.org/10.3390/life14121650

Cocuz, M.-E., Cocuz, I. G., Rodina, L., Tataranu, E., Caliman-Sturdza, O. A., & Filip, F. (2024). Treatment with Remdesivir of Children with SARS-CoV-2 Infection: Experience from a Clinical Hospital in Romania. Life (Basel, Switzerland), 14(3), 410. https://doi.org/10.3390/life14030410

Cohen, J. M., Mosnier, A., Valette, M., Bensoussan, J. L., Van Der Werf, S., & GROG-I. (2005). [General practice and surveillance : The example of influenza in France]. Medecine Et Maladies Infectieuses, 35(5). https://doi.org/10.1016/j.medmal.2005.02.012

Cohen, J. M., Silva, M. L., Caini, S., Ciblak, M., Mosnier, A., Daviaud, I., Matias, G., Badur, S., Valette, M., Enouf, V., Paget, J., Fleming, D. M., & IBGP study team. (2015). Striking Similarities in the Presentation and Duration of Illness of Influenza A and B in the Community : A Study Based on Sentinel Surveillance Networks in France and Turkey, 2010-2012. PloS One, 10(10). https://doi.org/10.1371/journal.pone.0139431

Coker, E. S., Molitor, J., Liverani, S., Martin, J., Maranzano, P., Pontarollo, N., & Vergalli, S. (2023). Bayesian profile regression to study the ecologic associations of correlated environmental exposures with excess mortality risk during the first year of the Covid-19 epidemic in lombardy, Italy. Environmental Research, 216(Pt 1). https://doi.org/10.1016/j.envres.2022.114484

Colamesta, V., Tamburrano, A., Barbara, A., Gentili, A., La Milia, D. I., Berloco, F., Cicchetti, A., Piacentini, D., Galluzzi, R., Mastrodonato, S. R., Cambieri, A., Ricciardi, W., & Laurenti, P. (2019). Cost-consequence analysis of influenza vaccination among the staff of a large teaching hospital in Rome, Italy : A pilot study. PloS One, 14(11). https://doi.org/10.1371/journal.pone.0225326

Collivignarelli, M. C., Abbà, A., Caccamo, F. M., Bertanza, G., Pedrazzani, R., Baldi, M., Ricciardi, P., & Carnevale Miino, M. (2021). Can particulate matter be identified as the primary cause of the rapid spread of CoViD-19 in some areas of Northern Italy? Environmental Science and Pollution Research International, 28(25). https://doi.org/10.1007/s11356-021-12735-x

Colombo, L., Witte, J., Gensorowsky, D., Batram, M., & Hadigal, S. (2024). Out of focus but still relevant ? Influenza-related resource utilization and vaccination coverage gaps in adults below 60 years of age with underlying conditions : An analysis of 2016-2024 real-world data in Germany. Journal of Medical Economics, 27(1). https://doi.org/10.1080/13696998.2024.2413284

Colson, P., Esteves-Vieira, V., Giraud-Gatineau, A., Zandotti, C., Filosa, V., Chaudet, H., Lagier, J., & Raoult, D. (2020). Temporal and age distributions of SARS-CoV-2 and other coronaviruses, southeastern France. INTERNATIONAL JOURNAL OF INFECTIOUS DISEASES, 101. https://doi.org/10.1016/j.ijid.2020.09.1417

Colson, P., Fantini, J., Yahi, N., Delerce, J., Levasseur, A., Fournier, P.-E., Lagier, J.-C., Raoult, D., & La Scola, B. (2022). Limited spread of a rare spike E484K-harboring SARS-CoV-2 in Marseille, France. Archives of Virology, 167(2). https://doi.org/10.1007/s00705-021-05331-4

Coma, E., Martinez-Marcos, M., Hermosilla, E., Mendioroz, J., Reñé, A., Fina, F., Perramon-Malavez, A., Prats, C., Cereza, G., Ciruela, P., Pineda, V., Antón, A., Ricós-Furió, G., Soriano-Arandes, A., & Cabezas, C. (2024). Effectiveness of nirsevimab immunoprophylaxis against respiratory syncytial virus-related outcomes in hospital and primary care settings : A retrospective cohort study in infants in Catalonia (Spain). Archives of Disease in Childhood, 109(9). https://doi.org/10.1136/archdischild-2024-327153

Comelli, A., Consonni, D., Lombardi, A., Viero, G., Oggioni, M., Bono, P., Uceda Renteria, S. C., Ceriotti, F., Mangioni, D., Muscatello, A., Piatti, A., Pesatori, A. C., Castaldi, S., Riboldi, L., Bandera, A., & Gori, A. (2021). Nasopharyngeal Testing among Healthcare Workers (HCWs) of a Large University Hospital in Milan, Italy during Two Epidemic Waves of COVID-19. International Journal of Environmental Research and Public Health, 18(16). https://doi.org/10.3390/ijerph18168748

Comelli, A., Focà, E., Sansone, E., Tomasi, C., Albini, E., Quiros-Roldan, E., Tomasoni, L. R., Sala, E., Bonfanti, C., Caccuri, F., Caruso, A., De Palma, G., & Castelli, F. (2021). Serological Response to SARS-CoV-2 in Health Care Workers Employed in a Large Tertiary Hospital in Lombardy, Northern Italy. Microorganisms, 9(3). https://doi.org/10.3390/microorganisms9030488

Consonni, D., Bono, P., Oggioni, M., Renteria, S. U., Piatti, A., Castaldi, S., Muscatello, A., Carugno, M., Riboldi, L., Ceriotti, F., Bandera, A., Gori, A., Pesatori, A. C., & Lombardi, A. (2021). Effectiveness of COVID-19 vaccine in health care workers, Milan, Italy. Occupational and Environmental Medicine, 78(SUPPL 1). Embase. https://doi.org/10.1136/OEM-2021-EPI.228

Consonni, D., Bordini, L., Nava, C., Todaro, A., Lunghi, G., Lombardi, A., Magioni, D., De Palo, F., Guerrieri, L., Gatti, M., Serra, D., Polonioli, M., Pratò, S., Muscatello, A., Bandera, A., Auxilia, F., & Castaldi, S. (2020). COVID-19 : What happened to the healthcare workers of a research and teaching hospital in Milan, Italy? Acta Bio-Medica: Atenei Parmensis, 91(3). https://doi.org/10.23750/abm.v91i3.10361

Consonni, D., Lombardi, A., Mangioni, D., Bono, P., Oggioni, M., Uceda Renteria, S., Valzano, A., Bordini, L., Nava, C. D., Tiwana, N., Gentiloni Silverj, F., Castaldi, S., Rognoni, M., Cavalieri D'Oro, L., Carugno, M., Luisetti, G., Riboldi, L., Ceriotti, F., Bandera, A., … Pesatori, A. C. (2022). Immunogenicity and effectiveness of BNT162b2 COVID-19 vaccine in a cohort of healthcare workers in Milan (Lombardy Region, Northern Italy). Epidemiologia E Prevenzione, 46(4). https://doi.org/10.19191/EP22.4.A513.065

Conti, S., Ferrara, P., Fornari, C., Harari, S., Madotto, F., Silenzi, A., Zucchi, A., Manzoli, L., & Mantovani, L. G. (2020). Estimates of the initial impact of the COVID-19 epidemic on overall mortality : Evidence from Italy. ERJ Open Research, 6(2). https://doi.org/10.1183/23120541.00179-2020

Conti, S., Ferrara, P., Mazzaglia, G., D'Orso, M. I., Ciampichini, R., Fornari, C., Madotto, F., Magoni, M., Sampietro, G., Silenzi, A., Sileo, C. V., Zucchi, A., Cesana, G., Manzoli, L., & Mantovani, L. G. (2020). Magnitude and time-course of excess mortality during COVID-19 outbreak : Population-based empirical evidence from highly impacted provinces in northern Italy. ERJ Open Research, 6(3). https://doi.org/10.1183/23120541.00458-2020

Contoli, B., Possenti, V., Minardi, V., Binkin, N. J., Ramigni, M., Carrozzi, G., & Masocco, M. (2021). What Is the Willingness to Receive Vaccination Against COVID-19 Among the Elderly in Italy ? Data From the PASSI d'Argento Surveillance System. Frontiers in Public Health, 9. https://doi.org/10.3389/fpubh.2021.736976

Contoli, B., Tosti, M. E., Asta, F., Minardi, V., Marchetti, G., Casigliani, V., Scarso, S., Declich, S., & Masocco, M. (2024). Exploring COVID-19 Vaccination Willingness in Italy : A Focus on Resident Foreigners and Italians Using Data from PASSI and PASSI d'Argento Surveillance Systems. Vaccines, 12(2). https://doi.org/10.3390/vaccines12020124

Cooper, B. S., Evans, S., Jafari, Y., Pham, T. M., Mo, Y., Lim, C., Pritchard, M. G., Pople, D., Hall, V., Stimson, J., Eyre, D. W., Read, J. M., Donnelly, C. A., Horby, P., Watson, C., Funk, S., Robotham, J. V., & Knight, G. M. (2023). The burden and dynamics of hospital-acquired SARS-CoV-2 in England. Nature, 623(7985). https://doi.org/10.1038/s41586-023-06634-z

Cordtz, R., Kristensen, S., Dalgaard, L. P. H., Westermann, R., Duch, K., Lindhardsen, J., Torp-Pedersen, C., & Dreyer, L. (2021). Incidence of COVID-19 Hospitalisation in Patients with Systemic Lupus Erythematosus : A Nationwide Cohort Study from Denmark. Journal of Clinical Medicine, 10(17). https://doi.org/10.3390/jcm10173842

Cordtz, R., Lindhardsen, J., Soussi, B. G., Vela, J., Uhrenholt, L., Westermann, R., Kristensen, S., Nielsen, H., Torp-Pedersen, C., & Dreyer, L. (2021). Incidence and severeness of COVID-19 hospitalization in patients with inflammatory rheumatic disease : A nationwide cohort study from Denmark. Rheumatology (Oxford, England), 60(SI). https://doi.org/10.1093/rheumatology/keaa897

Corea, F., Folcarelli, L., Napoli, A., Del Giudice, G. M., & Angelillo, I. F. (2022). The Impact of COVID-19 Vaccination in Changing the Adherence to Preventive Measures : Evidence from Italy. Vaccines, 10(5). https://doi.org/10.3390/vaccines10050777

Cornforth, F., Webber, L., Kerr, G., Dinsdale, H., Majeed, A., & Greengross, P. (2023). Impact of COVID-19 vaccination on COVID-19 hospital admissions in England during 2021 : An observational study. Journal of the Royal Society of Medicine, 116(5). https://doi.org/10.1177/01410768231157017

Corradini, C., Matheson, J., & Vanino, E. (2024). Neighbourhood labour structure, lockdown policies, and the uneven spread of COVID-19 : Within-city evidence from England. ECONOMICA, 91(363). https://doi.org/10.1111/ecca.12522

Corrao, G., Franchi, M., Cereda, D., Bortolan, F., Leoni, O., Vignati, E., Pavesi, G., & Gori, A. (2022). Increased risk of myocarditis and pericarditis and reduced likelihood of severe clinical outcomes associated with COVID-19 vaccination : A cohort study in Lombardy, Italy. BMC Infectious Diseases, 22(1). https://doi.org/10.1186/s12879-022-07823-3

Corrao, G., Franchi, M., Cereda, D., Bortolan, F., Zoli, A., Leoni, O., Borriello, C. R., Valle, G. P. D., Tirani, M., Pavesi, G., Barone, A., Ercolanoni, M., Jara, J., Galli, M., Bertolaso, G., & Mancia, G. (2022). Persistence of protection against SARS-CoV-2 clinical outcomes up to 9 months since vaccine completion : A retrospective observational analysis in Lombardy, Italy. The Lancet. Infectious Diseases, 22(5). https://doi.org/10.1016/S1473-3099(21)00813-6

Cosentino, F., Moscatt, V., Marino, A., Pampaloni, A., Scuderi, D., Ceccarelli, M., Benanti, F., Gussio, M., Larocca, L., Boscia, V., Vinci, G., Zagami, A., Onorante, A., Lupo, G., Torrisi, S., Grasso, S., Bruno, R., Iacobello, C., Bonfante, S., … Cacopardo, B. (2022). Clinical characteristics and predictors of death among hospitalized patients infected with SARS-CoV-2 in Sicily, Italy : A retrospective observational study. Biomedical Reports, 16(5). https://doi.org/10.3892/br.2022.1517

Costantino, A., Morlacchi, L., Donato, M. F., Gramegna, A., Farina, E., Dibenedetto, C., Campise, M., Redaelli, M., Perego, M., Alfieri, C., Blasi, F., Lampertico, P., & Favi, E. (2022). Hesitancy toward the Full COVID-19 Vaccination among Kidney, Liver and Lung Transplant Recipients in Italy. Vaccines, 10(11). https://doi.org/10.3390/vaccines10111899

Costantino, C., Graziano, G., Bonaccorso, N., Conforto, A., Cimino, L., Sciortino, M., Scarpitta, F., Giuffrè, C., Mannino, S., Bilardo, M., Ledda, C., Vitale, F., Restivo, V., & Mazzucco, W. (2022). Knowledge, Attitudes,

Perceptions and Vaccination Acceptance/Hesitancy among the Community Pharmacists of Palermo's Province, Italy : From Influenza to COVID-19. Vaccines, 10(3). https://doi.org/10.3390/vaccines10030475

Costantino, C., Mazzucco, W., Conforto, A., Cimino, L., Pieri, A., Rusignolo, S., Bonaccorso, N., Bravatà, F., Pipitone, L., Sciortino, M., Tocco, M., Zarcone, E., Graziano, G., Tramuto, F., Maida, C. M., Casuccio, A., & Vitale, F. (2024). Real-life experience on COVID-19 and seasonal influenza vaccines co-administration in the vaccination hub of the University Hospital of Palermo, Italy. Human Vaccines & Immunotherapeutics, 20(1). https://doi.org/10.1080/21645515.2024.2327229

Costantino, C., Restivo, V., Amodio, E., Colomba, G. M. E., Vitale, F., & Tramuto, F. (2019). A mid-term estimate of 2018/2019 vaccine effectiveness to prevent laboratory confirmed A(H1N1)pdm09 and A(H3N2) influenza cases in Sicily (Italy). Vaccine, 37(39). https://doi.org/10.1016/j.vaccine.2019.08.014

Coste, J., Delpierre, C., Richard, J.-B., Alleaume, C., Gallay, A., Tebeka, S., Lemogne, C., Robineau, O., Steichen, O., & Makovski, T. T. (2024). Prevalence of long COVID in the general adult population according to different definitions and sociodemographic and infection characteristics. A nationwide random sampling survey in France in autumn 2022. Clinical Microbiology and Infection: The Official Publication of the European Society of Clinical Microbiology and Infectious Diseases, 30(7). https://doi.org/10.1016/j.cmi.2024.03.020

Coudeville, L., Jollivet, O., Mahé, C., Chaves, S., & Gomez, G. B. (2021). Potential impact of introducing vaccines against COVID-19 under supply and uptake constraints in France : A modelling study. PloS One, 16(4). https://doi.org/10.1371/journal.pone.0250797

Courjon, J., Contenti, J., Demonchy, E., Levraut, J., Barbry, P., Rios, G., Dellamonica, J., Chirio, D., Bonnefoy, C., Giordanengo, V., & Carles, M. (2021). COVID-19 patients age, comorbidity profiles and clinical presentation related to the SARS-CoV-2 UK-variant spread in the Southeast of France. Scientific Reports, 11(1). https://doi.org/10.1038/s41598-021-95067-7

COVID-19 RISk and Treatments (CORIST) Collaboration. (2020). RAAS inhibitors are not associated with mortality in COVID-19 patients : Findings from an observational multicenter study in Italy and a meta-analysis of 19 studies. Vascular Pharmacology, 135. https://doi.org/10.1016/j.vph.2020.106805

Coyer, L., Boyd, A., Schinkel, J., Agyemang, C., Galenkamp, H., Koopman, A. D. M., Leenstra, T., Moll van Charante, E. P., van den Born, B.-J. H., Lok, A., Verhoeff, A., Zwinderman, A. H., Jurriaans, S., van Vught, L. A., Stronks, K., & Prins, M. (2022). SARS-CoV-2 antibody prevalence and correlates of six ethnic groups living in Amsterdam, the Netherlands : A population-based cross-sectional study, June-October 2020. BMJ Open, 12(1). https://doi.org/10.1136/bmjopen-2021-052752

Coyer, L., Wynberg, E., Buster, M., Wijffels, C., Prins, M., Schreijer, A., van Duijnhoven, Y. T. H. P., van Dam, A. P., van der Lubben, M., & Leenstra, T. (2021). Hospitalisation rates differed by city district and ethnicity during the first wave of COVID-19 in Amsterdam, The Netherlands. BMC Public Health, 21(1). https://doi.org/10.1186/s12889-021-11782-w

Cozza, V., Alfonsi, V., Rota, M. C., Paolini, V., & Ciofi degli Atti, M. L. (2015). Promotion of influenza vaccination among health care workers : Findings from a tertiary care children's hospital in Italy. BMC Public Health, 15. https://doi.org/10.1186/s12889-015-2067-9

Crépey, P., & Barthélemy, M. (2007). Detecting robust patterns in the spread of epidemics : A case study of influenza in the United States and France. American Journal of Epidemiology, 166(11). https://doi.org/10.1093/aje/kwm266

Crépey, P., Redondo, E., Díez-Domingo, J., Ortiz de Lejarazu, R., Martinón-Torres, F., Gil de Miguel, Á., López-Belmonte, J. L., Alvarez, F. P., Bricout, H., & Solozabal, M. (2020). From trivalent to quadrivalent influenza vaccines : Public health and economic burden for different immunization strategies in Spain. PloS One, 15(5). https://doi.org/10.1371/journal.pone.0233526

Crispino, F., Brinch, D., Carrozza, L., & Cappello, M. (2021). Vaccination against SARS-CoV-2 in IBD: Acceptance and concerns in a cohort from Southern Italy. United European Gastroenterology Journal, 9(SUPPL 8). Embase. https://doi.org/10.1002/ueg2.12144

Crispo, A., Di Gennaro, P., Coluccia, S., Gandini, S., Montagnese, C., Porciello, G., Nocerino, F., Grimaldi, M., Tafuri, M., Luongo, A., Rotondo, E., Amore, A., Labonia, F., Meola, S., Marone, S., Pierro, G., Menegozzo, S., Miscio, L., Perri, F., … Celentano, E. (2022). A SARS-CoV-2 Infection High-Uptake Program on Healthcare Workers and Cancer Patients of the National Cancer Institute of Naples, Italy. Healthcare (Basel, Switzerland), 10(2). https://doi.org/10.3390/healthcare10020205

Cromer, D., van Hoek, A. J., Jit, M., Edmunds, W. J., Fleming, D., & Miller, E. (2014). The burden of influenza in England by age and clinical risk group : A statistical analysis to inform vaccine policy. The Journal of Infection, 68(4). https://doi.org/10.1016/j.jinf.2013.11.013

Cromer, D., van Hoek, A. J., Newall, A. T., Pollard, A. J., & Jit, M. (2017). Burden of paediatric respiratory syncytial virus disease and potential effect of different immunisation strategies : A modelling and cost-effectiveness analysis for England. The Lancet. Public Health, 2(8). https://doi.org/10.1016/S2468-2667(17)30103-2

Crott, R., Pouplier, I., Roch, I., Chen, Y.-C., & Closon, M.-C. (2014). Pneumonia and influenza, and respiratory and circulatory hospital admissions in Belgium : A retrospective database study. Archives of Public Health = Archives Belges De Sante Publique, 72(1). https://doi.org/10.1186/2049-3258-72-33

Croxford, S., Emanuel, E., Ibitoye, A., Njoroge, J., Edmundson, C., Bardsley, M., Heinsbroek, E., Hope, V., & Phipps, E. (2021). Preliminary indications of the burden of COVID-19 among people who inject drugs in England and Northern Ireland and the impact on access to health and harm reduction services. Public Health, 192. https://doi.org/10.1016/j.puhe.2021.01.004

Culqui Lévano, D. R., Díaz, J., Blanco, A., Lopez, J. A., Navas, M. A., Sánchez-Martínez, G., Luna, M. Y., Hervella, B., Belda, F., & Linares, C. (2022). Mortality due to COVID-19 in Spain and its association with environmental factors and determinants of health. Environmental Sciences Europe, 34(1). https://doi.org/10.1186/s12302-022-00617-z

Curzio, O., Cori, L., Bianchi, F., Prinelli, F., Galli, M., Giacomelli, A., Imiotti, M. C., Jesuthasan, N., Recchia, V., & Adorni, F. (2023). COVID-19 Vaccine Hesitancy among Unvaccinated Adults : A Cross-Sectional Exploratory Analysis of Vaccination Intentions in Italy Related to Fear of Infection. Vaccines, 11(12). https://doi.org/10.3390/vaccines11121790

Cutrera, R., Ciofi Degli Atti, M. L., Dotta, A., D'Amore, C., Ravà, L., Perno, C. F., & Villani, A. (2024). Epidemiology of respiratory syncytial virus in a large pediatric hospital in Central Italy and development of a forecasting model to predict the seasonal peak. Italian Journal of Pediatrics, 50(1). https://doi.org/10.1186/s13052-024-01624-x

Cutrera, R., d'Angela, D., Orso, M., Guadagni, L., Vittucci, A. C., Bertoldi, I., Polistena, B., Spandonaro, F., Carrieri, C., Montuori, E. A., Iantomasi, R., & Orfeo, L. (2024). Trends in hospitalizations of children with respiratory syncytial virus aged less than 1 year in Italy, from 2015 to 2019. Italian Journal of Pediatrics, 50(1). https://doi.org/10.1186/s13052-024-01688-9

Cutrini, E., & Salvati, L. (2021). Unraveling spatial patterns of COVID-19 in Italy : Global forces and local economic drivers. Regional Science Policy & Practice, 13(Suppl 1). https://doi.org/10.1111/rsp3.12465

Czwojdzińska, M., Terpińska, M., Kuźniarski, A., Płaczkowska, S., & Piwowar, A. (2021). Exposure to PM2.5 and PM10 and COVID-19 infection rates and mortality : A one-year observational study in Poland. Biomedical Journal, 44(6 Suppl 1). https://doi.org/10.1016/j.bj.2021.11.006

D'accolti, M., Soffritti, I., Passaro, A., Zuliani, G., Antonioli, P., Mazzacane, S., Manfredini, R., & Caselli, E. (2020). SARS-CoV-2 RNA contamination on surfaces of a COVID-19 ward in a hospital of Northern Italy : What risk of transmission? European Review for Medical and Pharmacological Sciences, 24(17). https://doi.org/10.26355/eurrev_202009_22872

D'Ambrosi, F., Iurlaro, E., Tassis, B., Di Maso, M., Erra, R., Cetera, G. E., Cesano, N., Di Martino, D., Ossola, M. W., & Ferrazzi, E. M. (2021). Sociodemographic characteristics of pregnant women tested positive for COVID-19 admitted to a referral center in Northern Italy during lockdown period. The Journal of Obstetrics and Gynaecology Research, 47(5). https://doi.org/10.1111/jog.14729

D’Ambrosio, F., Lanza, T. E., Messina, R., Villani, L., Pezzullo, A. M., Ricciardi, W., Rosano, A., & Cadeddu, C. (2022). Influenza vaccination coverage in pediatric population in Italy : An analysis of recent trends. Italian Journal of Pediatrics, 48(1). https://doi.org/10.1186/s13052-022-01271-0

d’Andrea, V., Trentini, F., Marziano, V., Zardini, A., Manica, M., Guzzetta, G., Ajelli, M., Petrone, D., Del Manso, M., Sacco, C., Andrianou, X., Bella, A., Riccardo, F., Pezzotti, P., Poletti, P., & Merler, S. (2024). Spatial spread of COVID-19 during the early pandemic phase in Italy. BMC Infectious Diseases, 24(1), 450. https://doi.org/10.1186/s12879-024-09343-8

D’Angelo, N., Abbruzzo, A., & Adelfio, G. (2021). Spatio-Temporal Spread Pattern of COVID-19 in Italy. MATHEMATICS, 9(19). https://doi.org/10.3390/math9192454

D’Archivio, M., Cataldo, C., Del Manso, M., Petrone, D., Sacco, C., Vescio, M. F., Spuri, M., Rota, M. C., Bressi, M., Fabiani, M., Boros, S., Urdiales, A. M., Riccardo, F., Bella, A., Masella, R., Pezzotti, P., & Busani, L. (2022). Characteristics of COVID-19 cases in Italy from a sex/gender perspective. Annali Dell’Istituto Superiore Di Sanita, 58(4). https://doi.org/10.4415/ANN_22_04_02

D’Arminio Monforte, A., Tavelli, A., Bai, F., Tomasoni, D., Falcinella, C., Castoldi, R., Barbanotti, D., Mulè, G., Allegrini, M., Suardi, E., Tesoro, D., Tagliaferri, G., Mondatore, D., Augello, M., Cona, A., Beringheli, T., Gemignani, N., Sala, M., Varisco, B., … Marchetti, G. (2021). Declining Mortality Rate of Hospitalised Patients in the Second Wave of the COVID-19 Epidemics in Italy : Risk Factors and the Age-Specific Patterns. Life (Basel, Switzerland), 11(9). https://doi.org/10.3390/life11090979

d’Humières, C., Patrier, J., Lortat-Jacob, B., Tran-Dinh, A., Chemali, L., Maataoui, N., Rondinaud, E., Ruppé, E., Burdet, C., Ruckly, S., Montravers, P., Timsit, J.-F., & Armand-Lefevre, L. (2021). Two original observations concerning bacterial infections in COVID-19 patients hospitalized in intensive care units during the first wave of the epidemic in France. PloS One, 16(4). https://doi.org/10.1371/journal.pone.0250728

D’Onofrio, A., Manfredi, P., & Iannelli, M. (2021). Dynamics of partially mitigated multi-phasic epidemics at low susceptible depletion : Phases of COVID-19 control in Italy as case study. MATHEMATICAL BIOSCIENCES, 340. https://doi.org/10.1016/j.mbs.2021.108671

Dabrera, G., Allen, H., Zaidi, A., Flannagan, J., Twohig, K., Thelwall, S., Marchant, E., Aziz, N. A., Lamagni, T., Myers, R., Charlett, A., Capelastegui, F., Chudasama, D., Clare, T., Coukan, F., Sinnathamby, M., Ferguson, N., Hopkins, S., Chand, M., … COG-UK Consortium. (2022). Assessment of mortality and hospital admissions associated with confirmed infection with SARS-CoV-2 Alpha variant : A matched cohort and time-to-event analysis, England, October to December 2020. Euro Surveillance: Bulletin Europeen Sur Les Maladies Transmissibles = European Communicable Disease Bulletin, 27(20). https://doi.org/10.2807/1560-7917.ES.2022.27.20.2100377

Dabrera, G., Zhao, H., Andrews, N., Begum, F., Green, H., Ellis, J., Elias, K., Donati, M., Zambon, M., & Pebody, R. (2014). Effectiveness of seasonal influenza vaccination during pregnancy in preventing influenza infection in infants, England, 2013/14. Euro Surveillance: Bulletin Europeen Sur Les Maladies Transmissibles = European Communicable Disease Bulletin, 19(45). https://doi.org/10.2807/1560-7917.es2014.19.45.20959

Dähne, T., Bauer, W., Essig, A., Schaaf, B., Spinner, C., Pletz, M., Rohde, G., Rupp, J., Witzenrath, M., Panning, M., & CAPNETZ Study Grp. (2021). The impact of the SARS-CoV-2 pandemic on the prevalence of respiratory tract pathogens in patients with community-acquired pneumonia in Germany. EMERGING MICROBES & INFECTIONS, 10(1), 1515–1518. https://doi.org/10.1080/22221751.2021.1957402

Dal Negro, R. W., Turco, P., & Povero, M. (2018). Cost of influenza and influenza-like syndromes (I-LSs) in Italy : Results of a cross-sectional telephone survey on a representative sample of general population. Respiratory Medicine, 141. https://doi.org/10.1016/j.rmed.2018.07.001

Damm, O., Eichner, M., Rose, M. A., Knuf, M., Wutzler, P., Liese, J. G., Krüger, H., & Greiner, W. (2015). Public health impact and cost-effectiveness of intranasal live attenuated influenza vaccination of children in Germany. The European Journal of Health Economics: HEPAC: Health Economics in Prevention and Care, 16(5). https://doi.org/10.1007/s10198-014-0586-4

Damm, O., Krefft, A., Ahlers, J., Kramer, R., Witte, J., Batram, M., Schelling, J., & Greiner, W. (2023). Prevalence of chronic conditions and influenza vaccination coverage rates in Germany : Results of a health insurance claims data analysis. Influenza and Other Respiratory Viruses, 17(1). https://doi.org/10.1111/irv.13054

Dananché, C., Elias, C., Guibert, N., Gardes, S., Barreto, C., Denis, M.-A., Fascia, P., Gerbier, S., Grisi, B., Khanafer, N., Massardier-Pilonchéry, A., Munier-Marion, É., Pasquet, C., Fassier, J.-B., & Vanhems, P. (2022). COVID-19 clusters in a teaching hospital during the second wave of the SARS-CoV-2 pandemic in France: A descriptive study and lessons learned for waves to come. American Journal of Infection Control, 50(9), 1060–1063. https://doi.org/10.1016/j.ajic.2022.06.018

Danon, L., Brooks-Pollock, E., Bailey, M., & Keeling, M. (2021). A spatial model of COVID-19 transmission in England and Wales : Early spread, peak timing and the impact of seasonality. Philosophical Transactions of the Royal Society of London. Series B, Biological Sciences, 376(1829). https://doi.org/10.1098/rstb.2020.0272

Dao, T. L., Hoang, V. T., Nguyen, N. N., Delerce, J., Chaudet, H., Levasseur, A., Lagier, J. C., Raoult, D., Colson, P., & Gautret, P. (2021). Clinical outcomes in COVID-19 patients infected with different SARS-CoV-2 variants in Marseille, France. Clinical Microbiology and Infection: The Official Publication of the European Society of Clinical Microbiology and Infectious Diseases, 27(10). https://doi.org/10.1016/j.cmi.2021.05.029

Daperno, M., Guiotto, C., Casonato, I., Pagana, G., Micalizzi, S., Azzolina, M. C. R., Norbiato, C., Cosseddu, D., Rocca, R., & COVID-Serology in HCP Group. (2021). Risk factors of SARS-CoV-2 seroprevalence among hospital employees in Italy : A single-centre study. Internal Medicine Journal, 51(7). https://doi.org/10.1111/imj.15201

Darvishian, M., Dijkstra, F., van Doorn, E., Bijlsma, M. J., Donker, G. A., de Lange, M. M. A., Cadenau, L. M., Hak, E., & Meijer, A. (2017). Influenza Vaccine Effectiveness in the Netherlands from 2003/2004 through 2013/2014 : The Importance of Circulating Influenza Virus Types and Subtypes. PloS One, 12(1). https://doi.org/10.1371/journal.pone.0169528

Dasch, S., Wachinger, J., Bärnighausen, T., Chen, S., & McMahon, S. (2023). Deliberation, context, emotion and trust—Understanding the dynamics of adults' COVID-19 vaccination decisions in Germany. BMC PUBLIC HEALTH, 23(1). https://doi.org/10.1186/s12889-022-14587-7

David, G.-G., Rafael, H.-H., Ayelén, R.-B., Inmaculada, L.-G., Amparo, L., Marina, P., María, G.-V., Rebeca, R., & Diana, G.-B. (2022). Perimeter confinements of basic health zones and COVID-19 incidence in Madrid, Spain. BMC Public Health, 22(1). https://doi.org/10.1186/s12889-022-12626-x

Davidson, J., Banerjee, A., Mathur, R., Ramsay, M., Smeeth, L., Walker, J., McDonald, H., & Warren-Gash, C. (2021). Ethnic differences in the incidence of clinically diagnosed influenza : An England population-based cohort study 2008-2018. Wellcome Open Research, 6. https://doi.org/10.12688/wellcomeopenres.16620.3

Davies, M., & Roy, A. (2024). Comparing the rate of inpatient admissions of prison residents with COVID-19 to the general population in England in 2020/2021 using Hospital Episode Statistics data. BMJ Public Health, 2(1), e000515. https://doi.org/10.1136/bmjph-2023-000515

Davies, N. G., Abbott, S., Barnard, R. C., Jarvis, C. I., Kucharski, A. J., Munday, J. D., Pearson, C. A. B., Russell, T. W., Tully, D. C., Washburne, A. D., Wenseleers, T., Gimma, A., Waites, W., Wong, K. L. M., van Zandvoort, K., Silverman, J. D., CMMID COVID-19 Working Group, COVID-19 Genomics UK (COG-UK) Consortium, Diaz-Ordaz, K., … Edmunds, W. J. (2021). Estimated transmissibility and impact of SARS-CoV-2 lineage B.1.1.7 in England. Science (New York, N.Y.), 372(6538). https://doi.org/10.1126/science.abg3055

Davies, N. G., Barnard, R. C., Jarvis, C. I., Russell, T. W., Semple, M. G., Jit, M., Edmunds, W. J., Centre for Mathematical Modelling of Infectious Diseases COVID-19 Working Group, & ISARIC4C investigators. (2021). Association of tiered restrictions and a second lockdown with COVID-19 deaths and hospital admissions in England : A modelling study. The Lancet. Infectious Diseases, 21(4). https://doi.org/10.1016/S1473-3099(20)30984-1

De Angelis, E., Renzetti, S., Volta, M., Donato, F., Calza, S., Placidi, D., Lucchini, R. G., & Rota, M. (2021). COVID-19 incidence and mortality in Lombardy, Italy : An ecological study on the role of air pollution,

meteorological factors, demographic and socioeconomic variables. Environmental Research, 195. https://doi.org/10.1016/j.envres.2021.110777

De Angelis, M., Durastanti, C., Giovannoni, M., & Moretti, L. (2022). Spatio-temporal distribution pattern of COVID-19 in the Northern Italy during the first-wave scenario : The role of the highway network. Transportation Research Interdisciplinary Perspectives, 15. https://doi.org/10.1016/j.trip.2022.100646

De Carlo, A., Lo Caputo, S., Paolillo, C., Rosa, A. M., D'Orsi, U., De Palma, M., Reveglia, P., Lacedonia, D., Cinnella, G., Foschino, M. P., Margaglione, M., Mirabella, L., Santantonio, T. A., Corso, G., & Dattoli, V. (2020). SARS-COV-2 Serological Profile in Healthcare Professionals of a Southern Italy Hospital. International Journal of Environmental Research and Public Health, 17(24). https://doi.org/10.3390/ijerph17249324

De Cos, O., Castillo, V., & Cantarero, D. (2023). The Role of Functional Urban Areas in the Spread of COVID-19 Omicron (Northern Spain). Journal of Urban Health: Bulletin of the New York Academy of Medicine, 100(2). https://doi.org/10.1007/s11524-023-00720-3

De Cos, O., Castillo-Salcines, V. N., & Cantarero-Prieto, D. (2022). A geographical information system model to define COVID-19 problem areas with an analysis in the socio-economic context at the regional scale in the North of Spain. Geospatial Health, 17(s1). https://doi.org/10.4081/gh.2022.1067

DE Donno, A., Idolo, A., Quattrocchi, M., Zizza, A., Gabutti, G., Romano, A., Grima, P., Donatelli, I., & Guido, M. (2014). Surveillance of human influenza A(H3N2) virus from 1999 to 2009 in southern Italy. Epidemiology and Infection, 142(5). https://doi.org/10.1017/S095026881300201X

De Geyter, D., Vancutsem, E., Van Laere, S., Piérard, D., Lacor, P., Weets, I., & Allard, S. (2021). SARS-COV-2 seroprevalence among employees of a University Hospital in Belgium during the 2020 COVID-19 outbreak (COVEMUZ-study). Epidemiology and Infection, 149. https://doi.org/10.1017/S0950268821001540

De Jacobis, I. T., Vona, R., Cittadini, C., Marchesi, A., Cursi, L., Gambardella, L., Villani, A., & Straface, E. (2021). Clinical characteristics of children infected with SARS-CoV-2 in Italy. Italian Journal of Pediatrics, 47(1). https://doi.org/10.1186/s13052-021-01045-0

De Luca, L., D'Errigo, P., Rosato, S., Badoni, G., Giordani, B., Mureddu, G. F., Tavilla, A., Seccareccia, F., & Baglio, G. (2023). Impact of COVID-19 Diagnosis on Mortality in Patients with Ischemic Stroke Admitted during the 2020 Pandemic in Italy. Journal of Clinical Medicine, 12(14), 4560. https://doi.org/10.3390/jcm12144560

De Luca, L., Rosato, S., D'Errigo, P., Giordani, B., Mureddu, G. F., Badoni, G., Seccareccia, F., & Baglio, G. (2022). Impact of COVID-19 Diagnosis on Mortality in Patients with ST-Elevation Myocardial Infarction Hospitalized during the National Outbreak in Italy. Journal of Clinical Medicine, 11(24). https://doi.org/10.3390/jcm11247350

De Luca, L., Rosato, S., D'Errigo, P., Giordani, B., Mureddu, G. F., Badoni, G., Seccareccia, F., & Baglio, G. (2023). Covid-19 diagnosis and mortality in patients with non-ST-elevation myocardial infarction admitted in Italy during the national outbreak. International Journal of Cardiology, 370. https://doi.org/10.1016/j.ijcard.2022.11.008

De Marco, C., Veneziano, C., Massacci, A., Pallocca, M., Marascio, N., Quirino, A., Barreca, G., Giancotti, A., Gallo, L., Lamberti, A., Quaresima, B., Santamaria, G., Biamonte, F., Scicchitano, S., Trecarichi, E., Russo, A., Torella, D., Quattrone, A., Torti, C., … Viglietto, G. (2022). Dynamics of Viral Infection and Evolution of SARS-CoV-2 Variants in the Calabria Area of Southern Italy. FRONTIERS IN MICROBIOLOGY, 13. https://doi.org/10.3389/fmicb.2022.934993

De Paepe, A., Vlieghe, E., Brusselaers, N., Soentjens, P., Theunissen, C., Brosius, I., Grouwels, J., Van Petersen, L., van Tiggelen, H., Verbrugghe, W., Jorens, P. G., Lapperre, T., Peeters, K., Vermeulen, G., & van Ierssel, S. H. (2024). COVID-19 in three waves in a tertiary referral hospital in Belgium : A comparison of patient characteristics, management, and outcome. Virology Journal, 21(1). https://doi.org/10.1186/s12985-024-02360-8

De Rosa, F. G., Palazzo, A., Rosso, T., Shbaklo, N., Mussa, M., Boglione, L., Borgogno, E., Rossati, A., Mornese Pinna, S., Scabini, S., Chichino, G., Borrè, S., Del Bono, V., Garavelli, P. L., Barillà, D., Cattel, F., Di Perri, G., Ciccone, G., Lupia, T., & Corcione, S. (2021). Risk Factors for Mortality in COVID-19 Hospitalized

Patients in Piedmont, Italy : Results from the Multicenter, Regional, CORACLE Registry. Journal of Clinical Medicine, 10(9). https://doi.org/10.3390/jcm10091951

De Salazar, P. M., Gómez-Barroso, D., Pampaka, D., Gil, J. M., Peñalver, B., Fernández-Escobar, C., Lipsitch, M., Larrauri, A., Goldstein, E., & Hernán, M. A. (2020). Lockdown measures and relative changes in the age-specific incidence of SARS-CoV-2 in Spain. Epidemiology and Infection, 148. https://doi.org/10.1017/S0950268820002551

De Vito, A., Geremia, N., Fiore, V., Princic, E., Babudieri, S., & Madeddu, G. (2020). Clinical features, laboratory findings and predictors of death in hospitalized patients with COVID-19 in Sardinia, Italy. European Review for Medical and Pharmacological Sciences, 24(14). https://doi.org/10.26355/eurrev_202007_22291

de Boer, P. T., Nagy, L., Dolk, F. C. K., Wilschut, J. C., Pitman, R., & Postma, M. J. (2021). Cost-Effectiveness of Pediatric Influenza Vaccination in The Netherlands. Value in Health: The Journal of the International Society for Pharmacoeconomics and Outcomes Research, 24(1). https://doi.org/10.1016/j.jval.2020.10.011

de Courville, C., Bricout, H., Alvarez, F. P., Clouting, J., Patel, S., Mohamed, H., Giblin, S., & Coles, B. (2024). Secondary healthcare resource utilization and related costs associated with influenza-related hospital admissions in adult patients, England 2016—2020. Expert Review of Pharmacoeconomics & Outcomes Research. https://doi.org/10.1080/14737167.2024.2427307

de Fougerolles, T. R., Baïssas, T., Perquier, G., Vitoux, O., Crépey, P., Bartelt-Hofer, J., Bricout, H., & Petitjean, A. (2024). Public health and economic benefits of seasonal influenza vaccination in risk groups in France, Italy, Spain and the UK: state of play and perspectives. BMC Public Health, 24(1). https://doi.org/10.1186/s12889-024-18694-5

de Gier, B., Andeweg, S., Backer, J. A., RIVM COVID-19 surveillance and epidemiology team, Hahné, S. J., van den Hof, S., de Melker, H. E., Knol, M. J., & RIVM COVID-19 surveillance and epidemiology team (in addition to the named authors). (2021). Vaccine effectiveness against SARS-CoV-2 transmission to household contacts during dominance of Delta variant (B.1.617.2), the Netherlands, August to September 2021. Euro Surveillance: Bulletin Europeen Sur Les Maladies Transmissibles = European Communicable Disease Bulletin, 26(44). https://doi.org/10.2807/1560-7917.ES.2021.26.44.2100977

de Gier, B., Andeweg, S., Joosten, R., Ter Schegget, R., Smorenburg, N., van de Kassteele, J., RIVM COVID-19 surveillance and epidemiology team 1, Hahné, S. J., van den Hof, S., de Melker, H. E., Knol, M. J., & Members of the RIVM COVID-19 surveillance and epidemiology team. (2021). Vaccine effectiveness against SARS-CoV-2 transmission and infections among household and other close contacts of confirmed cases, the Netherlands, February to May 2021. Euro Surveillance: Bulletin Europeen Sur Les Maladies Transmissibles = European Communicable Disease Bulletin, 26(31). https://doi.org/10.2807/1560-7917.ES.2021.26.31.2100640

de Gier, B., van Asten, L., Boere, T. M., van Roon, A., van Roekel, C., Pijpers, J., van Werkhoven, C. H. H., van den Ende, C., Hahné, S. J. M., de Melker, H. E., Knol, M. J., & van den Hof, S. (2023). Effect of COVID-19 vaccination on mortality by COVID-19 and on mortality by other causes, the Netherlands, January 2021-January 2022. Vaccine, 41(31). https://doi.org/10.1016/j.vaccine.2023.06.005

de Meijere, G., Valdano, E., Castellano, C., Debin, M., Kengne-Kuetche, C., Turbelin, C., Noël, H., Weitz, J., Paolotti, D., Hermans, L., Hens, N., & Colizza, V. (2023). Attitudes towards booster, testing and isolation, and their impact on COVID-19 response in winter 2022/2023 in France, Belgium, and Italy. medRxiv, (de Meijere G.) Gran Sasso Science Institute (GSSI), L'Aquila, Italy. https://doi.org/10.1101/2022.12.30.22283726

de Miguel-Diez, J., Lopez-de-Andres, A., Jimenez-Garcia, R., de Miguel-Yanes, J. M., Hernández-Barrera, V., Carabantes-Alarcon, D., Zamorano-Leon, J. J., Lopez-Herranz, M., & Omaña-Palanco, R. (2022). Sex Differences in COVID-19 Hospitalization and Hospital Mortality among Patients with COPD in Spain : A Retrospective Cohort Study. Viruses, 14(6). https://doi.org/10.3390/v14061238

de Miguel-Yanes, J. M., Jimenez-Garcia, R., de Miguel-Diez, J., Hernández-Barrera, V., Carabantes-Alarcon, D., Zamorano-Leon, J. J., Omaña-Palanco, R., & Lopez-de-Andres, A. (2022). Impact of Type 2 Diabetes Mellitus on the Incidence and Outcomes of COVID-19 Needing Hospital Admission According to Sex : Retrospective Cohort Study Using Hospital Discharge Data in Spain, Year 2020. Journal of Clinical Medicine, 11(9). https://doi.org/10.3390/jcm11092654

de Nooijer, A. H., Kooistra, E. J., Grondman, I., Janssen, N. A. F., Joosten, L. A. B., van de Veerdonk, F. L., Kox, M., Pickkers, P., Netea, M. G., & RCI-COVID-19 study group. (2023). Adipocytokine plasma concentrations reflect influence of inflammation but not body mass index (BMI) on clinical outcomes of COVID-19 patients : A prospective observational study from the Netherlands. Clinical Obesity, 13(2). https://doi.org/10.1111/cob.12568

de San Segundo Reyes, M., Granizo Martínez, J. J., Veiga Crespo, M. C., Sanchís Ruiz, A., Camacho Muñoz, I., & Sánchez-Uriz, M. Á. (2023). [Factors associated with the duration of SARS-CoV-2 infection in healthcare professionals at a second-level public hospital in the Community of Madrid (Spain) during the sixth wave.]. Revista Espanola De Salud Publica, 97. http://www.ncbi.nlm.nih.gov/pubmed/36794790

Deák, G., Prangate, R., Croitoru, C., Matei, M., & Boboc, M. (2024). The first detection of SARS-CoV-2 RNA in the wastewater of Bucharest, Romania. Scientific Reports, 14(1), 21730. https://doi.org/10.1038/s41598-024-72854-6

Debin, M., Turbelin, C., Blanchon, T., Bonmarin, I., Falchi, A., Hanslik, T., Levy-Bruhl, D., Poletto, C., & Colizza, V. (2013). Evaluating the feasibility and participants' representativeness of an online nationwide surveillance system for influenza in France. PloS One, 8(9). https://doi.org/10.1371/journal.pone.0073675

Debski, M., Tsampasian, V., Haney, S., Blakely, K., Weston, S., Ntatsaki, E., Lim, M., Madden, S., Perperoglou, A., & Vassiliou, V. S. (2022). Post-COVID-19 syndrome risk factors and further use of health services in East England. PLOS Global Public Health, 2(11). https://doi.org/10.1371/journal.pgph.0001188

Decoster, A., Minten, T., & Spinnewijn, J. (2021). The Income Gradient in Mortality during the Covid-19 Crisis : Evidence from Belgium. Journal of Economic Inequality, 19(3). https://doi.org/10.1007/s10888-021-09505-7

Del Cura-Bilbao, A., López-Mendoza, H., Chaure-Pardos, A., Vergara-Ugarriza, A., & Guimbao-Bescós, J. (2022). Effectiveness of 3 COVID-19 Vaccines in Preventing SARS-CoV-2 Infections, January-May 2021, Aragon, Spain. Emerging Infectious Diseases, 28(3). https://doi.org/10.3201/eid2803.212027

Del Riccio, M., Boccalini, S., Rigon, L., Biamonte, M. A., Albora, G., Giorgetti, D., Bonanni, P., & Bechini, A. (2021). Factors Influencing SARS-CoV-2 Vaccine Acceptance and Hesitancy in a Population-Based Sample in Italy. Vaccines, 9(6). https://doi.org/10.3390/vaccines9060633

del Rosal, T., Baquero-Artigao, F., Calvo, C., Mellado, M. J., Molina, J. C., Santos, M. del M., Cilleruelo, M. J., Bueno, M., Storch de Gracia, P., Terol, C., Roa, M. Á., Piñeiro, R., García López-Hortelano, M., García-García, M. L., Rodríguez, S., Penín, M., Zarauza, A., Alvarado, F., de Blas, A., … Cabezas, M. E. (2011). Pandemic H1N1 influenza-associated hospitalizations in children in Madrid, Spain. Influenza and Other Respiratory Viruses, 5(6), e544-551. https://doi.org/10.1111/j.1750-2659.2011.00272.x

Delabre, R. M., Lapidus, N., Salez, N., Mansiaux, Y., de Lamballerie, X., & Carrat, F. (2015). Risk factors of pandemic influenza A/H1N1 in a prospective household cohort in the general population: Results from the CoPanFlu-France cohort. Influenza and Other Respiratory Viruses, 9(1), 43–50. https://doi.org/10.1111/irv.12294

Delestrain, C., Danis, K., Hau, I., Behillil, S., Billard, M.-N., Krajten, L., Cohen, R., Bont, L., & Epaud, R. (2021). Impact of COVID-19 social distancing on viral infection in France : A delayed outbreak of RSV. Pediatric Pulmonology, 56(12). https://doi.org/10.1002/ppul.25644

Delgado-Rodríguez, M., Castilla, J., Godoy, P., Martín, V., Soldevila, N., Alonso, J., Astray, J., Baricot, M., Cantón, R., Castro, A., Gónzález-Candelas, F., Mayoral, J. M., Quintana, J. M., Pumarola, T., Tamames, S., Sáez, M., Domínguez, A., & CIBERESP Cases and Controls in Pandemic Influenza Working Group. (2012). Prognosis of hospitalized patients with 2009 H1N1 influenza in Spain : Influence of neuraminidase inhibitors. The Journal of Antimicrobial Chemotherapy, 67(7). https://doi.org/10.1093/jac/dks098

Delgado-Rodríguez, M., Castilla, J., Godoy, P., Martín, V., Soldevila, N., Alonso, J., Astray, J., Baricot, M., Galán, J. C., Castro, A., Gónzález-Candelas, F., Mayoral, J. M., Quintana, J. M., Pumarola, T., Tamames, S., Sáez, M., Domínguez, A., & CIBERESP Cases and Controls in Pandemic Influenza Working Group, S. (2013). Different prognosis in hospitalized patients with influenza one season after the pandemic H1N1 influenza of

2009-2010 in Spain. Influenza and Other Respiratory Viruses, 7(6), 1336–1342. https://doi.org/10.1111/irv.12119

Delgado-Sánchez, S., Serrano-Ortiz, Á., Ruiz-Montero, R., Lorusso, N., Rumbao-Aguirre, J. M., & Salcedo-Leal, I. (2022). Impact of the first superspreading outbreak of COVID-19 related to a nightlife establishment in Andalusia, Spain. Journal of Healthcare Quality Research, 37(4). https://doi.org/10.1016/j.jhqr.2021.12.006

Delgado-Sanz, C., Mazagatos-Ateca, C., Oliva, J., Gherasim, A., & Larrauri, A. (2020). Illness Severity in Hospitalized Influenza Patients by Virus Type and Subtype, Spain, 2010-2017. Emerging Infectious Diseases, 26(2). https://doi.org/10.3201/eid2602.181732

Dellicour, S., Linard, C., Van Goethem, N., Da Re, D., Artois, J., Bihin, J., Schaus, P., Massonnet, F., Van Oyen, H., Vanwambeke, S. O., Speybroeck, N., & Gilbert, M. (2021). Investigating the drivers of the spatio-temporal heterogeneity in COVID-19 hospital incidence-Belgium as a study case. International Journal of Health Geographics, 20(1). https://doi.org/10.1186/s12942-021-00281-1

Demey, M., Bruyneel, A., Chatzis, O., Christiaens, C., Cossey, V., De Crombrugghe, G., De Lille, L., Goetghebuer, T., Gueulette, E., Hainaut, M., Heijmans, C., Hubinont, H., Lé, P.-Q., Lecomte, L., Mattijs, I., Mignon, C., Mondovits, B., Rodesch, M., Rooze, S., … Tilmanne, A. (2024). SARS-CoV-2 Infection in Children Less Than Forty Days Hospitalized in Belgium Between 2020 and 2022. The Pediatric Infectious Disease Journal, 43(9). https://doi.org/10.1097/INF.0000000000004400

Demont, C., Petrica, N., Bardoulat, I., Duret, S., Watier, L., Chosidow, A., Lorrot, M., Kieffer, A., & Lemaitre, M. (2021). Economic and disease burden of RSV-associated hospitalizations in young children in France, from 2010 through 2018. BMC Infectious Diseases, 21(1). https://doi.org/10.1186/s12879-021-06399-8

Dennis, J. M., Mateen, B. A., Sonabend, R., Thomas, N. J., Patel, K. A., Hattersley, A. T., Denaxas, S., McGovern, A. P., & Vollmer, S. J. (2021). Type 2 Diabetes and COVID-19-Related Mortality in the Critical Care Setting : A National Cohort Study in England, March-July 2020. Diabetes Care, 44(1). https://doi.org/10.2337/dc20-1444

Dentone, C., Portunato, F., Vena, A., Dettori, S., Mora, S., Ansaldi, F., & Bassetti, M. (2021). A comparative analysis of the first and second COVID-19 wave in Italy : Evaluation of mortality in the Infectious Disease Unit of Genoa University Hospital. The New Microbiologica, 44(4). http://www.ncbi.nlm.nih.gov/pubmed/35007331

Dentone, C., Vena, A., Loconte, M., Grillo, F., Brunetti, I., Barisione, E., Tedone, E., Mora, S., Di Biagio, A., Orsi, A., De Maria, A., Nicolini, L., Ball, L., Giacobbe, D. R., Magnasco, L., Delfino, E., Mastracci, L., Mangerini, R., Taramasso, L., … Bassetti, M. (2021). Bronchoalveolar lavage fluid characteristics and outcomes of invasively mechanically ventilated patients with COVID-19 pneumonia in Genoa, Italy. BMC Infectious Diseases, 21(1). https://doi.org/10.1186/s12879-021-06015-9

Denu, M. K. I., Montrond, A., Piltch-Loeb, R., Bonetti, M., Toffolutti, V., Testa, M. A., & Savoia, E. (2022). Freedom of Choice to Vaccinate and COVID-19 Vaccine Hesitancy in Italy. Vaccines, 10(11). https://doi.org/10.3390/vaccines10111973

Derqui, N., Nealon, J., Mira-Iglesias, A., Díez-Domingo, J., Mahé, C., & Chaves, S. S. (2022). Predictors of influenza severity among hospitalized adults with laboratory confirmed influenza : Analysis of nine influenza seasons from the Valencia region, Spain. Influenza and Other Respiratory Viruses, 16(5). https://doi.org/10.1111/irv.12985

Descamps, A., Launay, O., Bonnet, C., & Blondel, B. (2020). Seasonal influenza vaccine uptake and vaccine refusal among pregnant women in France : Results from a national survey. Human Vaccines & Immunotherapeutics, 16(5). https://doi.org/10.1080/21645515.2019.1688035

Descamps, A., Lenzi, N., Galtier, F., Lainé, F., Lesieur, Z., Vanhems, P., Amour, S., L'Honneur, A.-S., Fidouh, N., Foulongne, V., Lagathu, G., Duval, X., Merle, C., Lina, B., Carrat, F., Launay, O., Loubet, P., & FLUVAC study group. (2022). In-hospital and midterm post-discharge complications of adults hospitalised with respiratory syncytial virus infection in France, 2017-2019 : An observational study. The European Respiratory Journal, 59(3). https://doi.org/10.1183/13993003.00651-2021

Destordeur, L., Delhoulle, V., Papadopoulos, I., Fombellida, K., El Moussaoui, M., & Darcis, G. (2024). Factors contributing to pneumococcal, COVID-19 and influenza vaccine uptake among people living with HIV in Belgium: A retrospective study. JOURNAL OF THE INTERNATIONAL AIDS SOCIETY, 27, 212–213.

Devleesschauwer, B., Willem, L., Jurčević, J., Smith, P., Scohy, A., Wyper, G. M. A., Pires, S. M., Van Goethem, N., Beutels, P., Franco, N., Abrams, S., Van Cauteren, D., Speybroeck, N., Hens, N., & De Pauw, R. (2023). The direct disease burden of COVID-19 in Belgium in 2020 and 2021. BMC Public Health, 23(1). https://doi.org/10.1186/s12889-023-16572-0

Devroey, D., Semaille, P., Vansintejan, J., Vandevoorde, J., & Van De Vijver, E. (2011). Online monitoring of flu in Belgium. Influenza and Other Respiratory Viruses, 5(5). https://doi.org/10.1111/j.1750-2659.2011.00239.x

Dhada, M., & Montes, J. L. (2023). Demographic Influence on the Effectiveness of England's SARS-CoV-2 Policies. medRxiv, (Dhada M., mhd37@cam.ac.uk) Institute for Manufacturing, Department of Engineering, University of Cambridge, Cambridge, United Kingdom. https://doi.org/10.1101/2023.04.25.23288871

Di Carlo, P., Romano, A., Salsa, L., Gueli, A., Poma, A., Fucà, F., Dones, P., Collura, M., Pampinella, D., Motisi, D., & Corsello, G. (2009). Epidemiological assessment of Respiratory Syncytial Virus infection in hospitalized infants, during the season 2005-2006 in Palermo, Italy. Italian Journal of Pediatrics, 35(1). https://doi.org/10.1186/1824-7288-35-11

Di Cosimo, S., Tagliaferri, B., Generali, D., Giudici, F., Agustoni, F., Bernardo, A., Borgonovo, K., Farina, G., Luchena, G., Luciani, A., Nolè, F., Palmeri, L., Pietrantonio, F., Poggi, G., Zucali, P. A., Balletti, E., Catania, G., Bernocchi, O., D'Antonio, F., … Danova, M. (2021). Baseline Characteristics and Outcomes of Cancer Patients Infected with SARS-CoV-2 in the Lombardy Region, Italy (AIOM-L CORONA) : A Multicenter, Observational, Ambispective, Cohort Study. Cancers, 13(6). https://doi.org/10.3390/cancers13061324

Di Domenico, L., Pullano, G., Sabbatini, C. E., Boëlle, P.-Y., & Colizza, V. (2020). Impact of lockdown on COVID-19 epidemic in Île-de-France and possible exit strategies. BMC Medicine, 18(1). https://doi.org/10.1186/s12916-020-01698-4

Di Domenico, L., Sabbatini, C. E., Pullano, G., Lévy-Bruhl, D., & Colizza, V. (2021). Impact of January 2021 curfew measures on SARS-CoV-2 B.1.1.7 circulation in France. Euro Surveillance: Bulletin Europeen Sur Les Maladies Transmissibles = European Communicable Disease Bulletin, 26(15). https://doi.org/10.2807/1560-7917.ES.2021.26.15.2100272

Di Fabio, S., & La Torre, G. (2023). Analysis of the Economic Burden of COVID-19 on the Workers of a Teaching Hospital in the Centre of Italy : Changes in Productivity Loss and Healthcare Costs Pre and Post Vaccination Campaign. Vaccines, 11(12). https://doi.org/10.3390/vaccines11121791

Di Giallonardo, F., Puglia, I., Curini, V., Cammà, C., Mangone, I., Calistri, P., Cobbin, J. C. A., Holmes, E. C., & Lorusso, A. (2021). Emergence and Spread of SARS-CoV-2 Lineages B.1.1.7 and P.1 in Italy. Viruses, 13(5). https://doi.org/10.3390/v13050794

Di Giamberardino, P., Iacoviello, D., Papa, F., & Sinisgalli, C. (2021). A data-driven model of the COVID-19 spread among interconnected populations : Epidemiological and mobility aspects following the lockdown in Italy. Nonlinear Dynamics, 106(2). https://doi.org/10.1007/s11071-021-06840-2

Di Girolamo, C., Bartolini, L., Caranci, N., & Moro, M. L. (2020). Socioeconomic inequalities in overall and COVID-19 mortality during the first outbreak peak in Emilia-Romagna Region (Northern Italy). Epidemiologia E Prevenzione, 44(5-6 Suppl 2). https://doi.org/10.19191/EP20.5-6.S2.129

Di Girolamo, P. (2020). Assessment of the potential role of atmospheric particulate pollution and airborne transmission in intensifying the first wave pandemic impact of SARS-CoV-2/COVID-19 in Northern Italy. Bulletin of Atmospheric Science and Technology, 1(3), 515–550. https://doi.org/10.1007/s42865-020-00024-3

Di Giuseppe, G., Paduano, G., Vaienna, S., Maisto, G., Pelullo, C. P., & Pavia, M. (2022). Surveying Parents' Awareness and Adherence to Influenza Vaccination Recommendations in Children in Southern Italy. Vaccines, 10(8). https://doi.org/10.3390/vaccines10081298

Di Giuseppe, G., Pelullo, C. P., Lanzano, R., Lombardi, C., Nese, G., & Pavia, M. (2022). COVID-19 Vaccination Uptake and Related Determinants in Detained Subjects in Italy. Vaccines, 10(5). https://doi.org/10.3390/vaccines10050673

Di Lecce, V., Carpagnano, G. E., Pierucci, P., Quaranta, V. N., Barratta, F., Zito, A., Buonamico, E., & Resta, O. (2020). Baseline characteristics and outcomes of COVID-19 patients admitted to a Respiratory Intensive Care Unit (RICU) in Southern Italy. Multidisciplinary Respiratory Medicine, 15(1). https://doi.org/10.4081/mrm.2020.704

Di Lorenzo, A., Mangone, I., Colangeli, P., Cioci, D., Curini, V., Vincifori, G., Mercante, M. T., Di Pasquale, A., & Iannetti, S. (2023). One health system supporting surveillance during COVID-19 epidemic in Abruzzo region, southern Italy. One Health (Amsterdam, Netherlands), 16. https://doi.org/10.1016/j.onehlt.2022.100471

Di Lorenzo, G., Buonerba, L., Ingenito, C., Crocetto, F., Buonerba, C., Libroia, A., Sciarra, A., Ragone, G., Sanseverino, R., Iaccarino, S., Napodano, G., Imbimbo, C., Leo, E., Kozlakidis, Z., & De Placido, S. (2020). Clinical Characteristics of Metastatic Prostate Cancer Patients Infected with COVID-19 in South Italy. Oncology, 98(10). https://doi.org/10.1159/000509434

Di Maggio, E., Petrone, D., Del Manso, M., Riccardo, F., Bella, A., Brusaferro, S., & Pezzotti, P. (2025). Was severe SARS-CoV-2 substantially spreading in Northern Italy before its first detection in February 2020? An evaluation of pneumonia-associated hospitalization trends from September 2014 to February 2020. European Journal of Public Health, ckaf137. https://doi.org/10.1093/eurpub/ckaf137

Di Martino, D., Chiaffarino, F., Patanè, L., Prefumo, F., Vergani, P., Ornaghi, S., Savasi, V., Spinillo, A., Cromi, A., D'Ambrosi, F., Tassis, B., Iurlaro, E., Parazzini, F., & Ferrazzi, E. (2021). Assessing risk factors for severe forms of COVID-19 in a pregnant population : A clinical series from Lombardy, Italy. INTERNATIONAL JOURNAL OF GYNECOLOGY & OBSTETRICS, 152(2). https://doi.org/10.1002/ijgo.13435

Di Pasquale, A., Radomski, N., Mangone, I., Calistri, P., Lorusso, A., & Cammà, C. (2021). SARS-CoV-2 surveillance in Italy through phylogenomic inferences based on Hamming distances derived from pan-SNPs, -MNPs and -InDels. BMC Genomics, 22(1). https://doi.org/10.1186/s12864-021-08112-0

Di Pietra, G., Munegato, D., Poletto, C., Conciatori, V., Di Sopra, S., Franchin, E., Castagliuolo, I., Salata, C., & Del Vecchio, C. (2025). Surveillance of influenza viruses circulating from 2017/2018 to 2023/2024 seasons in Veneto Region, North-East Italy. Virology Journal, 22(1), 114. https://doi.org/10.1186/s12985-025-02723-9

Di Pietro, G. M., Ronzoni, L., Meschia, L. M., Tagliabue, C., Lombardi, A., Pinzani, R., Bosis, S., Marchisio, P. G., & Valenti, L. (2023). SARS-CoV-2 infection in children : A 24 months experience with focus on risk factors in a pediatric tertiary care hospital in Milan, Italy. Frontiers in Pediatrics, 11. https://doi.org/10.3389/fped.2023.1082083

Di Stefano, M., Sarno, M., Faleo, G., Farhan Mohamed, A. M., Lipsi, M. R., De Nittis, R., Bruno, S. R., De Feo, L., Granato, T., Corso, G., Arena, F., Centra, M., Lo Caputo, S., Margaglione, M., Santantonio, T. A., & Fiore, J. R. (2021). Low Prevalence of Antibodies to SARS-CoV-2 and Undetectable Viral Load in Seropositive Blood Donors from South-Eastern Italy. Acta Haematologica, 144(5). https://doi.org/10.1159/000515258

Diaconu, I. E., Irimie-Baluta, R. E., Vâta, A., Avadanei, A. N., Vasilescu, A. M., Ignat, B. E., & Luca, M. C. (2024). COVID-19 NEUROLOGICAL MANIFESTATIONS - THE EXPERIENCE OF A TERTIARY HOSPITAL FROM NORTHEASTERN ROMANIA. MEDICAL-SURGICAL JOURNAL-REVISTA MEDICO-CHIRURGICALA, 128(1), 21–33. https://doi.org/10.22551/MSJ.2024.01.03

Diao, Y., Kodera, S., Anzai, D., Gomez-Tames, J., Rashed, E. A., & Hirata, A. (2021). Influence of population density, temperature, and absolute humidity on spread and decay durations of COVID-19 : A comparative study of scenarios in China, England, Germany, and Japan. One Health (Amsterdam, Netherlands), 12. https://doi.org/10.1016/j.onehlt.2020.100203

Díaz-Simón, R., Lalueza, A., Lora-Tamayo, J., Rubio-Rivas, M., Mendo, C. L., Martínez, M. L. T., Méndez, C. A., Pesqueira Fontán, P. M., Cruz, A. F., Cabrera, J. L. R., Rodríguez, B. C., Rubio, A. E., de Ávila, V. S. R., García, G. M. G., Osorio, L. C., González-Fernández, M., Noya, A. G., Wittel, M. B., Fernandez, F. A., … SEMI-COVID-19 Network. (2021). Clinical Characteristics and Risk Factors of Respiratory Failure in a Cohort

of Young Patients Requiring Hospital Admission with SARS-CoV2 Infection in Spain : Results of the Multicenter SEMI-COVID-19 Registry. Journal of General Internal Medicine, 36(10). https://doi.org/10.1007/s11606-021-07066-z

Dickow, J., Gunawardene, M. A., Willems, S., Feldhege, J., Wohlmuth, P., Bachmann, M., Bergmann, M. W., Gesierich, W., Nowak, L., Pape, U.-F., Schreiber, R., Wirtz, S., Twerenbold, R., Sheikhzadeh, S., & Gessler, N. (2023). Higher in-hospital mortality in SARS-CoV-2 omicron variant infection compared to influenza infection-Insights from the CORONA Germany study. PloS One, 18(9). https://doi.org/10.1371/journal.pone.0292017

Dickson, M. M., Espa, G., Giuliani, D., Santi, F., & Savadori, L. (2020). Assessing the effect of containment measures on the spatio-temporal dynamic of COVID-19 in Italy. Nonlinear Dynamics, 101(3). https://doi.org/10.1007/s11071-020-05853-7

Diebner, H. H. (2021). Phase shift between age-specific COVID-19 incidence curves points to a potential epidemic driver function of kids and juveniles in Germany. medRxiv, (Diebner H.H., hans.diebner@rub.de) Dept. of Medical Informatics, Biometry and Epidemiology, Ruhr-Universität Bochum, Bochum, Germany. https://doi.org/10.1101/2021.11.29.21267004

Díez Domingo, J., Ridao López, M., Ubeda Sansano, I., & Ballester Sanz, A. (2006). [Incidence and cost of hospitalizations for bronchiolitis and respiratory syncytial virus infections in the autonomous community of Valencia in Spain (2001 and 2002)]. Anales De Pediatria (Barcelona, Spain: 2003), 65(4). https://doi.org/10.1157/13093515

Digregorio, M., Van Ngoc, P., Delogne, S., Meyers, E., Deschepper, E., Dardenne, N., Duysburgh, E., De Rop, L., De Burghgraeve, T., Coen, A., De Clercq, N., De Sutter, A., Verbakel, J. Y., Cools, P., Heytens, S., Buret, L., & Scholtes, B. (2024). Vaccine hesitancy for the COVID-19 vaccine booster dose among nursing home staff fully vaccinated with the primary vaccination course in Belgium. Vaccine: X, 16. https://doi.org/10.1016/j.jvacx.2024.100453

Dijkstra, F., Donker, G. A., Wilbrink, B., Van Gageldonk-Lafeber, A. B., & Van Der Sande, M. a. B. (2009). Long time trends in influenza-like illness and associated determinants in The Netherlands. Epidemiology and Infection, 137(4). https://doi.org/10.1017/S095026880800126X

Dimeglio, C., Miedougé, M., Loubes, J.-M., Mansuy, J.-M., & Izopet, J. (2021a). Estimating the impact of public health strategies on the spread of SARS-CoV-2 : Epidemiological modelling for Toulouse, France. Reviews in Medical Virology, 31(5). https://doi.org/10.1002/rmv.2224

Dings, C., Götz, K. M., Och, K., Sihinevich, I., Werthner, Q., Smola, S., Bliem, M., Mahfoud, F., Volk, T., Kreuer, S., Rissland, J., Selzer, D., & Lehr, T. (2022). Model-Based Analysis of SARS-CoV-2 Infections, Hospitalization and Outcome in Germany, the Federal States and Districts. Viruses, 14(10). https://doi.org/10.3390/v14102114

Dings, C., Selzer, D., Bragazzi, N. L., Möhler, E., Wenning, M., Gehrke, T., Richter, U., Nonnenmacher, A., Brinkmann, F., Rothoeft, T., Zemlin, M., Lücke, T., & Lehr, T. (2024). Effect of vaccinations and school restrictions on the spread of COVID-19 in different age groups in Germany. Infectious Disease Modelling, 9(4). https://doi.org/10.1016/j.idm.2024.07.004

Dini, G., Montecucco, A., Rahmani, A., Barletta, C., Pellegrini, L., Debarbieri, N., Orsi, A., Caligiuri, P., Varesano, S., Manca, A., Vargiu, M. P., Di Carlo, P., Massa, E., Icardi, G., & Durando, P. (2021). Clinical and epidemiological characteristics of COVID-19 during the early phase of the SARS-CoV-2 pandemic: A cross-sectional study among medical school physicians and residents employed in a regional reference teaching hospital in Northern Italy. International Journal of Occupational Medicine and Environmental Health, 34(2), 189–201. https://doi.org/10.13075/ijomeh.1896.01759

Dios-Guerra, C., Carmona-Torres, J. M., López-Soto, P. J., Morales-Cané, I., & Rodríguez-Borrego, M. A. (2017). Prevalence and factors associated with influenza vaccination of persons over 65 years old in Spain (2009-2014). Vaccine, 35(51). https://doi.org/10.1016/j.vaccine.2017.10.086

Djuric, O., Larosa, E., Cassinadri, M., Cilloni, S., Bisaccia, E., Pepe, D., Vicentini, M., Venturelli, F., Bonvicini, L., Giorgi Rossi, P., Pezzotti, P., Mateo Urdiales, A., Bedeschi, E., & Reggio Emilia Covid-19 Working Group.

(2022). Surveillance, contact tracing and characteristics of SARS-CoV-2 transmission in educational settings in Northern Italy, September 2020 to April 2021. PloS One, 17(10). https://doi.org/10.1371/journal.pone.0275667

Djuric, O., Mancuso, P., Zannini, A., Nicolaci, A., Massari, M., Zerbini, A., Belloni, L., Collini, G., Sampaolesi, F., Celotti, A., Boni, I., Rossi, P., & Reggio Emilia COVID-19 Working Grp. (2021). Are Individuals with Substance Use Disorders at Higher Risk of SARS-CoV-2 Infection ? Population-Based Registry Study in Northern Italy. EUROPEAN ADDICTION RESEARCH, 27(4). https://doi.org/10.1159/000515101

Djuric, O., Ottone, M., Vicentini, M., Venturelli, F., Pezzarossi, A., Manicardi, V., Greci, M., Giorgi Rossi, P., & Reggio Emilia Covid-19 Working Group. (2022). Diabetes and COVID-19 testing, positivity, and mortality : A population-wide study in Northern Italy. Diabetes Research and Clinical Practice, 191. https://doi.org/10.1016/j.diabres.2022.110051

Dobi, A., Sandenon Seteyen, A.-L., Lalarizo Rakoto, M., Lebeau, G., Vagner, D., Frumence, É., Giry, C., Septembre-Malaterre, A., Raffray, L., & Gasque, P. (2020). Serological Surveillance of COVID-19 Hospitalized Patients in Réunion Island (France) Revealed that Specific Immunoglobulin G Are Rapidly Vanishing in Severe Cases. Journal of Clinical Medicine, 9(12). https://doi.org/10.3390/jcm9123847

Doblhammer, G., Kreft, D., & Reinke, C. (2021). Regional Characteristics of the Second Wave of SARS-CoV-2 Infections and COVID-19 Deaths in Germany. International Journal of Environmental Research and Public Health, 18(20). https://doi.org/10.3390/ijerph182010663

Doblhammer, G., Reinke, C., & Kreft, D. (2022). Social disparities in the first wave of COVID-19 incidence rates in Germany : A county-scale explainable machine learning approach. BMJ Open, 12(2). https://doi.org/10.1136/bmjopen-2021-049852

Doenhardt, M., Gano, C., Sorg, A.-L., Diffloth, N., Tenenbaum, T., von Kries, R., Berner, R., & Armann, J. P. (2022). Burden of Pediatric SARS-CoV-2 Hospitalizations during the Omicron Wave in Germany. Viruses, 14(10). https://doi.org/10.3390/v14102102

Doenhardt, M., Hufnagel, M., Diffloth, N., Hübner, J., Mauer, R., Schneider, D. T., Simon, A., Tenenbaum, T., Trotter, A., Armann, J., Berner, R., & DGPI COVID-19 working group. (2024). Epidemiology of 7375 children and adolescents hospitalized with COVID-19 in Germany, reported via a prospective, nationwide surveillance study in 2020-2022. Scientific Reports, 14(1). https://doi.org/10.1038/s41598-023-49210-1

Doerre, A., & Doblhammer, G. (2022). The influence of gender on COVID-19 infections and mortality in Germany: Insights from age- and gender-specific modeling of contact rates, infections, and deaths in the early phase of the pandemic. PloS One, 17(5), e0268119. https://doi.org/10.1371/journal.pone.0268119

Doglietto, F., Vezzoli, M., Gheza, F., Lussardi, G. L., Domenicucci, M., Vecchiarelli, L., Zanin, L., Saraceno, G., Signorini, L., Panciani, P. P., Castelli, F., Maroldi, R., Rasulo, F. A., Benvenuti, M. R., Portolani, N., Bonardelli, S., Milano, G., Casiraghi, A., Calza, S., & Fontanella, M. M. (2020). Factors Associated With Surgical Mortality and Complications Among Patients With and Without Coronavirus Disease 2019 (COVID-19) in Italy. JAMA Surgery, 155(8). https://doi.org/10.1001/jamasurg.2020.2713

Dolby, T., Finning, K., Baker, A., Fowler-Dowd, L., Khunti, K., Razieh, C., Yates, T., & Nafilyan, V. (2022). Monitoring sociodemographic inequality in COVID-19 vaccination uptake in England : A national linked data study. Journal of Epidemiology and Community Health, 76(7). https://doi.org/10.1136/jech-2021-218415

Dolk, C., Eichner, M., Welte, R., Anastassopoulou, A., Van Bellinghen, L.-A., Poulsen Nautrup, B., Van Vlaenderen, I., Schmidt-Ott, R., Schwehm, M., & Postma, M. (2016). Cost-Utility of Quadrivalent Versus Trivalent Influenza Vaccine in Germany, Using an Individual-Based Dynamic Transmission Model. PharmacoEconomics, 34(12). https://doi.org/10.1007/s40273-016-0443-7

Domènech-Montoliu, S., Puig-Barberà, J., Badenes-Marques, G., Gil-Fortuño, M., Orrico-Sánchez, A., Pac-Sa, M. R., Perez-Olaso, O., Sala-Trull, D., Sánchez-Urbano, M., & Arnedo-Pena, A. (2023). Long COVID Prevalence and the Impact of the Third SARS-CoV-2 Vaccine Dose : A Cross-Sectional Analysis from the Third Follow-Up of the Borriana Cohort, Valencia, Spain (2020-2022). Vaccines, 11(10). https://doi.org/10.3390/vaccines11101590

Domínguez, À., Soldevila, N., Toledo, D., Godoy, P., Castilla, J., Force, L., Morales, M., Mayoral, J. M., Egurrola, M., Tamames, S., Martín, V., Astray, J., & Working Group of the Project PI12/02079. (2016). Factors Associated with Influenza Vaccination of Hospitalized Elderly Patients in Spain. PloS One, 11(1). https://doi.org/10.1371/journal.pone.0147931

Domínguez, A., Soldevila, N., Toledo, D., Godoy, P., Espejo, E., Fernandez, M. A., Mayoral, J. M., Castilla, J., Egurrola, M., Tamames, S., Astray, J., Morales-Suárez-Varela, M., & The Working Group Of The Project Pi/. (2017). The effectiveness of influenza vaccination in preventing hospitalisations of elderly individuals in two influenza seasons : A multicentre case-control study, Spain, 2013/14 and 2014/15. Euro Surveillance: Bulletin Europeen Sur Les Maladies Transmissibles = European Communicable Disease Bulletin, 22(34). https://doi.org/10.2807/1560-7917.ES.2017.22.34.30602

Domnich, A., Icardi, G., Panatto, D., Scarpaleggia, M., Trombetta, C.-S., Ogliastro, M., Stefanelli, F., Bruzzone, B., & Orsi, A. (2024). Influenza epidemiology and vaccine effectiveness during the 2023/2024 season in Italy : A test-negative case-control study. International Journal of Infectious Diseases: IJID: Official Publication of the International Society for Infectious Diseases, 147. https://doi.org/10.1016/j.ijid.2024.107202

Domnicu, A., Mogoi, M., Manea, A., Boia, E. R., & Boia, M. (2022). Clinical Factors Associated with COVID-19 Severity in Chronic Hospitalized Infants and Toddlers: Data from a Center in the West Part of Romania. Healthcare (Basel, Switzerland), 10(5), 808. https://doi.org/10.3390/healthcare10050808

Donaldson, L. J., Rutter, P. D., Ellis, B. M., Greaves, F. E. C., Mytton, O. T., Pebody, R. G., & Yardley, I. E. (2010). Mortality from pandemic A/H1N1 2009 influenza in England: Public health surveillance study. BMJ (Online), 340(7737), 82. https://doi.org/10.1136/bmj.b5213

Donati, S., Corsi, E., Maraschini, A., Salvatore, M. A., & ItOSS-COVID-19 Working Group. (2022). SARS-CoV-2 infection among hospitalised pregnant women and impact of different viral strains on COVID-19 severity in Italy : A national prospective population-based cohort study. BJOG: An International Journal of Obstetrics and Gynaecology, 129(2). https://doi.org/10.1111/1471-0528.16980

Dong, M., Zhang, X., Yang, K., Liu, R., & Chen, P. (2021). Forecasting the COVID-19 transmission in Italy based on the minimum spanning tree of dynamic region network. PeerJ, 9. https://doi.org/10.7717/peerj.11603

Dong, Y., He, Z., Liu, T., Huang, J., Zhang, C. J. P., Akinwunmi, B., & Ming, W.-K. (2022). Acceptance of and Preference for COVID-19 Vaccination in India, the United Kingdom, Germany, Italy, and Spain : An International Cross-Sectional Study. Vaccines, 10(6). https://doi.org/10.3390/vaccines10060832

Donida, B. M., Pirola, F. S., Opizzi, R., & Assembergs, P. (2024). First COVID-19 wave in the province of Bergamo, Italy : Epidemiological and clinical characteristics, outcome and management of the first hospitalized patients. BMC Infectious Diseases, 24(1). https://doi.org/10.1186/s12879-024-09034-4

Donker, T., Van Boven, M., Van Ballegooijen, W. M., Van'T Klooster, T. M., Wielders, C. C., & Wallinga, J. (2011). Nowcasting pandemic influenza A/H1N1 2009 hospitalizations in the Netherlands. European Journal of Epidemiology, 26(3), 195–201. https://doi.org/10.1007/s10654-011-9566-5

Donnachie, E., Hapfelmeier, A., Linde, K., Tauscher, M., Gerlach, R., Greißel, A., & Schneider, A. (2022). Incidence of Post-Covid Syndrome and Associated Symptoms in Outpatient Care in Bavaria, Germany. medRxiv, (Donnachie E.; Tauscher M.) Bavarian Association of Statutory Health Insurance Physicians, Kassenärztliche Vereinigung Bayerns, Munich, Germany. https://doi.org/10.1101/2022.05.29.22275262

Donsimoni, J. R., Glawion, R., Plachter, B., & Wälde, K. (2020). [Projecting the Spread of COVID-19 for Germany]. Wirtschaftsdienst (Hamburg, Germany: 1949), 100(4). https://doi.org/10.1007/s10273-020-2631-5

Donzelli, A., Alessandria, M., & Orlando, L. (2021). Comparison of hospitalizations and deaths from COVID-19 2021 versus 2020 in Italy : Surprises and implications. F1000Research, 10. https://doi.org/10.12688/f1000research.73132.1

Donzelli, G., Biggeri, A., Tobias, A., Nottmeyer, L. N., & Sera, F. (2022). Role of meteorological factors on SARS-CoV-2 infection incidence in Italy and Spain before the vaccination campaign. A multi-city time series study. Environmental Research, 211. https://doi.org/10.1016/j.envres.2022.113134

Dörr, S., Joachim, R., Chatzitomaris, A., & Lobmann, R. (2023). [Risk factors for outcome and mortality in hospitalized geriatric patients with SARS-CoV-2 infection : Data from a hospital of maximum care during in the period of the second corona wave 2020/2021 in Germany]. Zeitschrift Fur Gerontologie Und Geriatrie, 56(2). https://doi.org/10.1007/s00391-023-02161-8

Dorrucci, M., Minelli, G., Boros, S., Manno, V., Prati, S., Battaglini, M., Corsetti, G., Andrianou, X., Riccardo, F., Fabiani, M., Vescio, M. F., Spuri, M., Mateo-Urdiales, A., Del Manso, M., Pezzotti, P., Bella, A., Italian Integrated Surveillance COVID-19 Group, & Italian Integrated Surveillance COVID-19 group. (2022). A population-based cohort approach to assess excess mortality due to the spread of COVID-19 in Italy, January-May 2020. Annali Dell'Istituto Superiore Di Sanita, 58(1). https://doi.org/10.4415/ANN_22_01_04

Doyle, A., Bonmarin, I., Lévy-Bruhl, D., Le Strat, Y., & Desenclos, J.-C. (2006). Influenza pandemic preparedness in France: Modelling the impact of interventions. Journal of Epidemiology and Community Health, 60(5), 399–404. https://doi.org/10.1136/jech.2005.034082

Drăgănescu, A. C., Miron, V. D., Streinu-Cercel, A., Florea, D., Vlaicu, O., Bilaşco, A., Oţelea, D., Luminos, M. L., Piţigoi, D., Streinu-Cercel, A., & Săndulescu, O. (2021). Circulation of influenza A viruses among patients hospitalized for severe acute respiratory infection in a tertiary care hospital in Romania in the 2018/19 season: Results from an observational descriptive epidemiological study. Medicine, 100(52), e28460. https://doi.org/10.1097/MD.0000000000028460

Drăgănescu, A., Săndulescu, O., Florea, D., Vlaicu, O., Streinu-Cercel, A., Oţelea, D., Aramă, V., Luminos, M. L., Streinu-Cercel, A., Niţescu, M., Ivanciuc, A., Bacruban, R., & Piţigoi, D. (2018). The influenza season 2016/17 in Bucharest, Romania—Surveillance data and clinical characteristics of patients with influenza-like illness admitted to a tertiary infectious diseases hospital. The Brazilian Journal of Infectious Diseases: An Official Publication of the Brazilian Society of Infectious Diseases, 22(5), 377–386. https://doi.org/10.1016/j.bjid.2018.10.275

Drăgănescu, A., Săndulescu, O., Florea, D., Vlaicu, O., Streinu-Cercel, A., Oțelea, D., Luminos, M. L., Aramă, V., Abrudan, S., Streinu-Cercel, A., & Pițigoi, D. (2019). The 2017-2018 influenza season in Bucharest, Romania: Epidemiology and characteristics of hospital admissions for influenza-like illness. BMC Infectious Diseases, 19(1), 967. https://doi.org/10.1186/s12879-019-4613-z

Dragano, N., Dortmann, O., Timm, J., Mohrmann, M., Wehner, R., Rupprecht, C. J., Scheider, M., Mayatepek, E., & Wahrendorf, M. (2022). Association of Household Deprivation, Comorbidities, and COVID-19 Hospitalization in Children in Germany, January 2020 to July 2021. JAMA Network Open, 5(10). https://doi.org/10.1001/jamanetworkopen.2022.34319

Drago, G., Pérez-Sádaba, F. J., Aceituno, S., Gari, C., & López-Belmonte, J. L. (2023). Healthcare resource use and associated costs in a cohort of hospitalized COVID-19 patients in Spain: A retrospective analysis from the first to the third pandemic wave. EPICOV study. PloS One, 18(1), e0280940. https://doi.org/10.1371/journal.pone.0280940

Drefahl, S., Wallace, M., Mussino, E., Aradhya, S., Kolk, M., Brandén, M., Malmberg, B., & Andersson, G. (2020). A population-based cohort study of socio-demographic risk factors for COVID-19 deaths in Sweden. Nature Communications, 11(1). https://doi.org/10.1038/s41467-020-18926-3

Drobnik, J., Susło, R., Pobrotyn, P., Fabich, E., Magiera, V., Diakowska, D., & Uchmanowicz, I. (2021). COVID-19 among Healthcare Workers in the University Clinical Hospital in Wroclaw, Poland. International Journal of Environmental Research and Public Health, 18(11). https://doi.org/10.3390/ijerph18115600

Dropkin, G. (2022). Variation in COVID-19 booster uptake in England : An ecological study. PloS One, 17(6). https://doi.org/10.1371/journal.pone.0270624

Drysdale, M., Hautekiet, T., Singh, M., Hautekiet, J., Ludikhuyze, L., Patel, V., Gibbons, D. C., De Roeck, D., Colpaert, K., Lloyd, E. J., & Van Braeckel, E. (2023). Characteristics and outcomes of patients treated with sotrovimab to prevent progression to severe COVID-19 in Belgium. medRxiv, (Drysdale M., myriam.g.drysdale@gsk.com; Singh M.; Patel V.; Gibbons D.C.; Lloyd E.J.) GSK, Brentford, United Kingdom. https://doi.org/10.1101/2023.12.14.23298578

Duarte-Salles, T., Vizcaya, D., Pistillo, A., Casajust, P., Sena, A. G., Lai, L. Y. H., Prats-Uribe, A., Ahmed, W.-U.-R., Alshammari, T. M., Alghoul, H., Alser, O., Burn, E., You, S. C., Areia, C., Blacketer, C., DuVall, S., Falconer, T., Fernandez-Bertolin, S., Fortin, S., … Prieto-Alhambra, D. (2020). Baseline characteristics, management, and outcomes of 55,270 children and adolescents diagnosed with COVID-19 and 1,952,693 with influenza in France, Germany, Spain, South Korea and the United States : An international network cohort study. medRxiv: The Preprint Server for Health Sciences. https://doi.org/10.1101/2020.10.29.20222083

Duchemin, L., Veber, P., & Boussau, B. (2022). Bayesian investigation of SARS-CoV-2-related mortality in France. PEER COMMUNITY JOURNAL, 2. https://doi.org/10.24072/pcjournal.84

Dumke, R., Geissler, M., Skupin, A., Helm, B., Mayer, R., Schubert, S., Oertel, R., Renner, B., & Dalpke, A. H. (2022). Simultaneous Detection of SARS-CoV-2 and Influenza Virus in Wastewater of Two Cities in Southeastern Germany, January to May 2022. International Journal of Environmental Research and Public Health, 19(20). https://doi.org/10.3390/ijerph192013374

Durando, P., Alicino, C., Alberti, M., Sticchi, L., Turello, V., Marensi, L., Caiazzo, A. L., Panico, M. G., Giugliano, F., Parlato, A., Peluso, F., Sgricia, S., Icardi, G., & Italian Intradermal Influenza Vaccine Working Group. (2012). Acceptance and safety of the intradermal influenza vaccine among the elderly in Italy : An on-field national study. Advances in Therapy, 29(4). https://doi.org/10.1007/s12325-012-0012-1

Dziadzko, M., Belhassen, M., Van Ganse, E., Heritier, F., Berard, M., Marant-Micallef, C., & Aubrun, F. (2024). Health Care Resource Use and Total Mortality After HospitalAdmission for Severe COVID-19 Infections During the InitialPandemic Wave in France:Descriptive Study. JMIR PUBLIC HEALTH AND SURVEILLANCE, 10. https://doi.org/10.2196/56398

Dziedzic, A., Issa, J., Hussain, S., Tanasiewicz, M., Wojtyczka, R., Kubina, R., Konwinska, M. D., & Riad, A. (2022). COVID-19 vaccine booster hesitancy (VBH) of healthcare professionals and students in Poland : Cross-sectional survey-based study. Frontiers in Public Health, 10. https://doi.org/10.3389/fpubh.2022.938067

Eales, O., de Oliveira Martins, L., Page, A. J., Wang, H., Bodinier, B., Tang, D., Haw, D., Jonnerby, J., Atchison, C., Ashby, D., Barclay, W., Taylor, G., Cooke, G., Ward, H., Darzi, A., Riley, S., Elliott, P., Donnelly, C. A., & Chadeau-Hyam, M. (2022a). Dynamics of competing SARS-CoV-2 variants during the Omicron epidemic in England. Nature Communications, 13(1). Embase. https://doi.org/10.1038/s41467-022-32096-4

Eales, O., Haw, D., Wang, H., Atchison, C., Ashby, D., Cooke, G. S., Barclay, W., Ward, H., Darzi, A., Donnelly, C. A., Chadeau-Hyam, M., Elliott, P., & Riley, S. (2023). Dynamics of SARS-CoV-2 infection hospitalisation and infection fatality ratios over 23 months in England. PLoS Biology, 21(5). https://doi.org/10.1371/journal.pbio.3002118

Eales, O., Page, A., Martins, L., Wang, H., Bodinier, B., Haw, D., Jonnerby, J., Atchison, C., Ashby, D., Barclay, W., Taylor, G., Cooke, G., Ward, H., Darzi, A., Riley, S., Chadeau-Hyam, M., Donnelly, C., Elliott, P., & COVID-19 Genomics UK COG-UK Consor. (2022). SARS-CoV-2 lineage dynamics in England from September to November 2021 : High diversity of Delta sub-lineages and increased transmissibility of AY.4.2. BMC INFECTIOUS DISEASES, 22(1). https://doi.org/10.1186/s12879-022-07628-4

Eales, O., Wang, H., Haw, D., Ainslie, K. E. C., Walters, C. E., Atchison, C., Cooke, G., Barclay, W., Ward, H., Darzi, A., Ashby, D., Donnelly, C. A., Elliott, P., & Riley, S. (2022). Trends in SARS-CoV-2 infection prevalence during England’s roadmap out of lockdown, January to July 2021. PLoS Computational Biology, 18(11). https://doi.org/10.1371/journal.pcbi.1010724

Eberhardt, J., Al-Qerem, W., & Ling, J. (2024). Comparing COVID-19 booster vaccine acceptance in the United Kingdom, Germany, Austria, and Jordan : The role of protection motivation theory, conspiracy beliefs, social media use and religiosity. Vaccine, 42(26). https://doi.org/10.1016/j.vaccine.2024.126474

Eberle, U., Heinzinger, S., Konrad, R., Wimmer, C., Liebl, B., Katz, K., Ackermann, N., Sing, A., & Bavarian SARS-CoV-Public Health Laboratory and Epidemiology Team. (2021). Virological COVID-19 surveillance in Bavaria, Germany suggests no SARS-CoV-2 spread prior to the first German case in January 2020. Infection, 49(5). https://doi.org/10.1007/s15010-021-01611-y

Ebranati, E., Pariani, E., Piralla, A., Gozalo-Margüello, M., Veo, C., Bubba, L., Amendola, A., Ciccozzi, M., Galli, M., Zanetti, A., Baldanti, F., & Zehender, G. (2015). Reconstruction of the Evolutionary Dynamics of A(H3N2) Influenza Viruses Circulating in Italy from 2004 to 2012. PLOS ONE, 10(9). https://doi.org/10.1371/journal.pone.0137099

Edelstein, M., Obi, C., Chand, M., Hopkins, S., Brown, K., & Ramsay, M. (2021). SARS-CoV-2 infection in London, England : Changes to community point prevalence around lockdown time, March-May 2020. Journal of Epidemiology and Community Health, 75(2). https://doi.org/10.1136/jech-2020-214730

Edyko, P., Zdunek, M., Nowicka, M., & Kurnatowska, I. (2025). Prevalence and Risk Factors for Acute Kidney Injury in COVID-19-Hospitalized Patients in Poland Across Three Pandemic Periods. Journal of Clinical Medicine, 14(4), 1384. https://doi.org/10.3390/jcm14041384

Egeskov-Cavling, A. M., Hansen, C. L., Johannesen, C. K., Lindegaard Madsen, B., Bhatt, S., Viboud, C., & Fischer, T. K. (2025). Mortality and Hospitalizations Associated with Respiratory Syncytial Virus, Influenza, and COVID-19, Denmark. SSRN. https://doi.org/10.2139/ssrn.5199147

Egeskov-Cavling, A. M., Hansen, C. L., Johannesen, C. K., Lindegaard, B., Bhatt, S., Viboud, C., & Fischer, T. K. (2025). Excess mortality and hospitalisations associated with respiratory syncytial virus, influenza, and COVID-19 among adults in Denmark (2015-2024): A modelling study. The Lancet Regional Health. Europe, 55, 101396. https://doi.org/10.1016/j.lanepe.2025.101396

Eggeling, R., König, F., Koeppel, L., Böhler, L.-I., Böhm, M., Schmeißer, N., Pfeifer, N., Kaiser, R., & Clinical Virology Network. (2025). Long-term impact of the SARS-CoV-2 pandemic on respiratory viruses in Germany. BMC Public Health, 25(1), 2654. https://doi.org/10.1186/s12889-025-23983-8

Ehlken, B., Anastassopoulou, A., Hain, J., Schröder, C., & Wahle, K. (2015). Cost for physician-diagnosed influenza and influenza-like illnesses on primary care level in Germany—Results of a database analysis from May 2010 to April 2012. BMC Public Health, 15. https://doi.org/10.1186/s12889-015-1885-0

Eichner, M., Schwehm, M., Hain, J., Uphoff, H., Salzberger, B., Knuf, M., & Schmidt-Ott, R. (2014). 4Flu—An individual based simulation tool to study the effects of quadrivalent vaccination on seasonal influenza in Germany. BMC Infectious Diseases, 14. https://doi.org/10.1186/1471-2334-14-365

Einhauser, S., Peterhoff, D., Beileke, S., Günther, F., Niller, H.-H., Steininger, P., Knöll, A., Korn, K., Berr, M., Schütz, A., Wiegrebe, S., Stark, K. J., Gessner, A., Burkhardt, R., Kabesch, M., Schedl, H., Küchenhoff, H., Pfahlberg, A. B., Heid, I. M., … Wagner, R. (2022). Time Trend in SARS-CoV-2 Seropositivity, Surveillance Detection- and Infection Fatality Ratio until Spring 2021 in the Tirschenreuth County-Results from a Population-Based Longitudinal Study in Germany. Viruses, 14(6). https://doi.org/10.3390/v14061168

Eiros-Bouza, J. M., & Pérez-Rubio, A. (2015). [Burden of influenza virus type B and mismatch with the flu vaccine in Spain]. Revista Espanola De Quimioterapia: Publicacion Oficial De La Sociedad Espanola De Quimioterapia, 28(1). http://www.ncbi.nlm.nih.gov/pubmed/25690144

Ejnar Hansen, M., & David Pickering, S. (2024). The role of religion and COVID-19 vaccine uptake in England. Vaccine, 42(13). https://doi.org/10.1016/j.vaccine.2024.04.006

El-Battrawy, I., Nuñez-Gil, I. J., Abumayyaleh, M., Estrada, V., Manuel Becerra-Muñoz, V., Uribarri, A., Fernández-Rozas, I., Feltes, G., Arroyo-Espliguero, R., Trabattoni, D., López-País, J., Pepe, M., Romero, R., Castro-Mejía, A. F., Cerrato, E., Capel Astrua, T., D'Ascenzo, F., Fabregat-Andres, O., Signes-Costa, J., … Akin, I. (2021). COVID-19 and the impact of arterial hypertension-An analysis of the international HOPE COVID-19 Registry (Italy-Spain-Germany). European Journal of Clinical Investigation, 51(11). https://doi.org/10.1111/eci.13582

Elenis, E., Kallner, H. K., Karalexi, M. A., Hägg, D., Linder, M., Fall, K., Papadopoulos, F. C., & Skalkidou, A. (2024). Estrogen-modulating treatment among mid-life women and COVID-19 morbidity and mortality : A multiregister nationwide matched cohort study in Sweden. BMC Medicine, 22(1). https://doi.org/10.1186/s12916-024-03297-z

Elias, C., Fournier, A., Vasiliu, A., Beix, N., Demillac, R., Tillaut, H., Guillois, Y., Eyebe, S., Mollo, B., & Crépey, P. (2017). Seasonal influenza vaccination coverage and its determinants among nursing homes personnel in western France. BMC Public Health, 17(1). https://doi.org/10.1186/s12889-017-4556-5

Elliot, A. J., & Fleming, D. M. (2006). Surveillance of influenza-like illness in England and Wales during 1966-2006. Euro Surveillance: Bulletin Europeen Sur Les Maladies Transmissibles = European Communicable Disease Bulletin, 11(10). http://www.ncbi.nlm.nih.gov/pubmed/17130657

Elliot, A. J., Powers, C., Thornton, A., Obi, C., Hill, C., Simms, I., Waight, P., Maguire, H., Foord, D., Povey, E., Wreghitt, T., Goddard, N., Ellis, J., Bermingham, A., Sebastianpillai, P., Lackenby, A., Zambon, M., Brown, D., Smith, G. E., & Gill, O. N. (2009). Monitoring the emergence of community transmission of influenza A/H1N1 2009 in England : A cross sectional opportunistic survey of self sampled telephone callers to NHS Direct. BMJ (Clinical Research Ed.), 339. https://doi.org/10.1136/bmj.b3403

Elliott, P., Eales, O., Bodinier, B., Tang, D., Wang, H., Jonnerby, J., Haw, D., Elliott, J., Whitaker, M., Walters, C. E., Atchison, C., Diggle, P. J., Page, A. J., Trotter, A. J., Ashby, D., Barclay, W., Taylor, G., Ward, H., Darzi, A., … Donnelly, C. A. (2022). Dynamics of a national Omicron SARS-CoV-2 epidemic during January 2022 in England. Nature Communications, 13(1). Embase. https://doi.org/10.1038/s41467-022-32121-6

Elliott, P., Whitaker, M., Tang, D., Eales, O., Steyn, N., Bodinier, B., Wang, H., Elliott, J., Atchison, C., Ashby, D., Barclay, W., Taylor, G., Darzi, A., Cooke, G. S., Ward, H., Donnelly, C. A., Riley, S., & Chadeau-Hyam, M. (2023). Design and Implementation of a National SARS-CoV-2 Monitoring Program in England : REACT-1 Study. American Journal of Public Health, 113(5). https://doi.org/10.2105/AJPH.2023.307230

Elson, R., Davies, T. M., Lake, I. R., Vivancos, R., Blomquist, P. B., Charlett, A., & Dabrera, G. (2021). The spatio-temporal distribution of COVID-19 infection in England between January and June 2020. Epidemiology and Infection, 149. https://doi.org/10.1017/S0950268821000534

Emborg, H. D., Krause, T. G., Nielsen, L., Thomsen, M. K., Christiansen, C. B., Skov, M. N., Nielsen, X. C., Weinreich, L. S., Fischer, T. K., Rønn, J., & Trebbien, R. (2016). Influenza vaccine effectiveness in adults 65 years and older, Denmark, 2015/16—A rapid epidemiological and virological assessment. Euro Surveillance: Bulletin Europeen Sur Les Maladies Transmissibles = European Communicable Disease Bulletin, 21(14). https://doi.org/10.2807/1560-7917.ES.2016.21.14.30189

Emborg, H.-D., Krause, T. G., Hviid, A., Simonsen, J., & Mølbak, K. (2011). Effectiveness of vaccine against pandemic influenza A/H1N1 among people with underlying chronic diseases: Cohort study, Denmark, 2009-10. BMJ (Clinical Research Ed.), 344, d7901. https://doi.org/10.1136/bmj.d7901

Emborg, H.-D., Valentiner-Branth, P., Trebbien, R., Bolt Botnen, A., Grove Krause, T., & Søborg, B. (2025). Enhanced influenza vaccines impact effectiveness in individuals aged 65 years and older, Denmark, 2024/25 influenza season up to 4 March 2025. Euro Surveillance: Bulletin Europeen Sur Les Maladies Transmissibles = European Communicable Disease Bulletin, 30(12), 2500174. https://doi.org/10.2807/1560-7917.ES.2025.30.12.2500174

Emborg, H.-D., Vestergaard, L. S., Botnen, A. B., Nielsen, J., Krause, T. G., & Trebbien, R. (2022). A late sharp increase in influenza detections and low interim vaccine effectiveness against the circulating A(H3N2) strain, Denmark, 2021/22 influenza season up to 25 March 2022. Euro Surveillance: Bulletin Europeen Sur Les Maladies Transmissibles = European Communicable Disease Bulletin, 27(15). https://doi.org/10.2807/1560-7917.ES.2022.27.15.2200278

Emmett, H. E., Hall, J., Webster, H. H., Izzard, A., Singanayagam, A., Zambon, M., & Dabrera, G. (2025). Post-Mortem Community Surveillance of COVID-19: Implementation and Evaluation of a Pilot System in the Funeral Sector in England, UK, January 2021 to February 2022. Influenza and Other Respiratory Viruses, 19(6), e70116. https://doi.org/10.1111/irv.70116

Enciu, B. G., Draganescu, A. C., Pitiigoi, D., Sandulescu, O., Craciun, M. D., Bilasco, A., Streinu-Cercel, A., Streinu-Cercel, A., Florea, D., Miron, V. D., & Arama, V. (2022). Comparative Analysis of Clinical and Epidemiological Characteristics in Patients with SARI Confirmed as Influenza or COVID-19 Admitted in a Tertiary Care Hospital in Bucharest, Romania. PROCESSES, 10(2). https://doi.org/10.3390/pr10020327

Enciu, B. G., Pitigoi, D., Zaharia, A., Popescu, R., Sirbu, A., Sandulescu, O., Streinu-cercel, A., Draganescu, A. C., Streinu-cercel, A., Pistol, A., Rafila, A., & Arama, V. (2023). THE INFLUENZA VACCINATION UPTAKE IN ROMANIA DURING THE 2022-2023 SEASON. FARMACIA, 71(6), 1289–1294. https://doi.org/10.31925/farmacia.2023.6.20

Englund, H., Campe, H., & Hautmann, W. (2013). Effectiveness of trivalent and monovalent influenza vaccines against laboratory-confirmed influenza infection in persons with medically attended influenza-like illness in Bavaria, Germany, 2010/2011 season. Epidemiology and Infection, 141(9). https://doi.org/10.1017/S0950268812002282

Enserink, R., Meijer, A., Dijkstra, F., Van Benthem, B., Van Der Steen, J. T., Haenen, A., Van Delden, H., Cools, H., Van Der Sande, M., & Veldman-Ariesen, M.-J. (2011). Absence of influenza A(H1N1) during seasonal and pandemic seasons in a sentinel nursing home surveillance network in the Netherlands. Journal of the American Geriatrics Society, 59(12), 2301–2305. https://doi.org/10.1111/j.1532-5415.2011.03715.x

Epelboin, S., Labrosse, J., De Mouzon, J., Fauque, P., Gervoise-Boyer, M., Levy, R., Sermondade, N., Hesters, L., Bergère, M., Devienne, C., Jonveaux, P., Ghosn, J., & Pessione, F. (2021). Obstetrical outcomes and maternal morbidities associated with COVID-19 in pregnant women in France : A national retrospective cohort study. PLOS MEDICINE, 18(11). https://doi.org/10.1371/journal.pmed.1003857

Erazo, D., Vincenti-Gonzalez, M. F., van Loenhout, J. A. F., Hubin, P., Vandromme, M., Maes, P., Taquet, M., Van Weyenbergh, J., Catteau, L., & Dellicour, S. (2022). Investigating COVID-19 Vaccine Impact on the Risk of Hospitalisation through the Analysis of National Surveillance Data Collected in Belgium. Viruses, 14(6). https://doi.org/10.3390/v14061315

Erber, J., Kappler, V., Haller, B., Mijočević, H., Galhoz, A., Prazeres da Costa, C., Gebhardt, F., Graf, N., Hoffmann, D., Thaler, M., Lorenz, E., Roggendorf, H., Kohlmayer, F., Henkel, A., Menden, M. P., Ruland, J., Spinner, C. D., Protzer, U., Knolle, P., … SeCoMRI Study Group2. (2022). Infection Control Measures and Prevalence of SARS-CoV-2 IgG among 4,554 University Hospital Employees, Munich, Germany. Emerging Infectious Diseases, 28(3). https://doi.org/10.3201/eid2803.204436

Erdwiens, A., Hackmann, C., Wedde, M., Biere, B., Reiche, J., Preuß, U., Tolksdorf, K., Buda, S., & Dürrwald, R. (2025). Interim Estimates of 2024-2025 Seasonal Influenza Vaccine Effectiveness in Germany-Data From Primary Care and Hospital Sentinel Surveillance. Influenza and Other Respiratory Viruses, 19(5), e70115. https://doi.org/10.1111/irv.70115

Eriksen, A. R. R., Fogh, K., Hasselbalch, R. B., Bundgaard, H., Nielsen, S. D., Jørgensen, C. S., Scharff, B. F. S. S., Erikstrup, C., Sækmose, S. G., Holm, D. K., Aagaard, B., Kristensen, J. H., Bødker, C. A., Norsk, J. B., Nielsen, P. B., Østergaard, L., Ellermann-Eriksen, S., Andersen, B., Nielsen, H., … Iversen, K. (2022). SARS-CoV-2 antibody prevalence among homeless people and shelter workers in Denmark : A nationwide cross-sectional study. BMC Public Health, 22(1). https://doi.org/10.1186/s12889-022-13642-7

Escolano-Utrilla, S., Roca-Medina, A., & Barrado-Timón, D. (2024). Spatial disparities in incidence of COVID-19 in relation to economic and socio-demographic factors in the Autonomous Community of Madrid, Spain. DOCUMENTS D ANALISI GEOGRAFICA, 70(3). https://doi.org/10.5565/rev/dag.904

Escribano Ceruelo, E., Espinel Ruíz, M. A., Ortega López-Peláez, M., Fernández Garoz, B., Asensio Antón, J., & Jiménez García, R. (2022). Seroprevalence of antibodies against SARS-CoV-2 among health care workers in a pediatric monographic hospital in Madrid (Spain). Enfermedades Infecciosas Y Microbiologia Clinica (English Ed.), 40(6). https://doi.org/10.1016/j.eimce.2022.03.009

Escribano, P., Pérez-Granda, M. J., Alonso, R., Catalán, P., Alcalá, L., Serra-Rexarch, J. A., Osuna, L., Fernández, A., Conti, A. P., Castellanos, A., Guinea, J., Muñoz, P., & Bouza, E. (2022). High incidence of COVID-19 at nursing homes in Madrid, Spain, despite preventive measures. Revista Espanola De Quimioterapia: Publicacion Oficial De La Sociedad Espanola De Quimioterapia, 35(3). https://doi.org/10.37201/req/008.2022

Espenhain, L., Tribler, S., Særke Jørgensen, C., Holm Hansen, C., Wolff Sönksen, U., & Ethelberg, S. (2021). Prevalence of SARS-CoV-2 antibodies in Denmark : Nationwide, population-based seroepidemiological study. European Journal of Epidemiology, 36(7). https://doi.org/10.1007/s10654-021-00796-8

Espinosa-Gongora, C., Berg, C., Rehn, M., Varg, J. E., Dillner, L., Latorre-Margalef, N., Székely, A. J., Andersson, E., & Movert, E. (2023). Early detection of the emerging SARS-CoV-2 BA.2.86 lineage through integrated genomic surveillance of wastewater and COVID-19 cases in Sweden, weeks 31 to 38 2023. Euro Surveillance: Bulletin Europeen Sur Les Maladies Transmissibles = European Communicable Disease Bulletin, 28(46). https://doi.org/10.2807/1560-7917.ES.2023.28.46.2300595

Esposito, S., Bosis, S., Pelucchi, C., Tremolati, E., Sabatini, C., Semino, M., Marchisio, P., della Croce, F., & Principi, N. (2008). Influenza vaccination among healthcare workers in a multidisciplinary University hospital in Italy. BMC Public Health, 8. https://doi.org/10.1186/1471-2458-8-422

Esposito, S., Molteni, C. G., Daleno, C., Valzano, A., Fossali, E., Da Dalt, L., Cecinati, V., Bruzzese, E., Giacchino, R., Giaquinto, C., Galeone, C., Lackenby, A., & Principi, N. (2010). Clinical importance and impact on the households of oseltamivir-resistant seasonal A/H1N1 influenza virus in healthy children in Italy. Virology Journal, 7. https://doi.org/10.1186/1743-422X-7-202

Esposito, S., Piralla, A., Zampiero, A., Bianchini, S., Di Pietro, G., Scala, A., Pinzani, R., Fossali, E., Baldanti, F., & Principi, N. (2015). Characteristics and Their Clinical Relevance of Respiratory Syncytial Virus Types and Genotypes Circulating in Northern Italy in Five Consecutive Winter Seasons. PloS One, 10(6). https://doi.org/10.1371/journal.pone.0129369

Estadilla, C. D. S., Mar, J., Ibarrondo, O., Stollenwerk, N., & Aguiar, M. (2024). Impact of High Covid-19 Vaccination Rate in an Aging Population : Estimating Averted Hospitalizations and Deaths in the Basque Country, Spain Using Counterfactual Modeling. Journal of Epidemiology and Global Health, 14(3). https://doi.org/10.1007/s44197-024-00286-6

Esteban-Vasallo, M. D., Domínguez-Berjón, M. F., Aerny-Perreten, N., Astray-Mochales, J., Martín-Martínez, F., & Gènova-Maleras, R. (2012). Pandemic influenza A (H1N1) 2009 in Madrid, Spain: Incidence and characteristics in immigrant and native population. European Journal of Public Health, 22(6), 792–796. https://doi.org/10.1093/eurpub/ckr171

Fabiani, M., Di Napoli, A., Riccardo, F., Gargiulo, L., Declich, S., & Petrelli, A. (2017). [Differences in influenza vaccination coverage among subgroups of adult immigrants residing in Italy at risk for complications (2012-2013)]. Epidemiologia E Prevenzione, 41(3-4 (Suppl 1)). https://doi.org/10.19191/EP17.3-4S1.P050.065

Fabiani, M., Mateo-Urdiales, A., Sacco, C., Fotakis, E. A., Battilomo, S., Petrone, D., Del Manso, M., Bella, A., Riccardo, F., Stefanelli, P., Palamara, A. T., Pezzotti, P., & Italian Integrated Surveillance of COVID-19 study group and of the Italian COVID-19 Vaccines Registry group. (2024). Effectiveness against severe COVID-19 of a seasonal booster dose of bivalent (original/Omicron BA.4-5) mRNA vaccines in persons aged ≥60 years : Estimates over calendar time and by time since administration during prevalent circulation of different Omicron subvariants, Italy, 2022-2023. Vaccine, 42(23). https://doi.org/10.1016/j.vaccine.2024.05.074

Fabiani, M., Mateo-Urdiales, A., Sacco, C., Rota, M. C., Fotakis, E. A., Petrone, D., Del Manso, M., Siddu, A., Stefanelli, P., Bella, A., Riccardo, F., Rezza, G., Palamara, A. T., Brusaferro, S., Pezzotti, P., & Italian Integrated Surveillance of COVID-19 study group and of the Italian COVID-19 Vaccines Registry group. (2023). Relative effectiveness of bivalent Original/Omicron BA.4-5 mRNA vaccine in preventing severe COVID-19 in persons 60 years and above during SARS-CoV-2 Omicron XBB.1.5 and other XBB sublineages circulation, Italy, April to June 2023. Euro Surveillance: Bulletin Europeen Sur Les Maladies Transmissibles = European Communicable Disease Bulletin, 28(32). https://doi.org/10.2807/1560-7917.ES.2023.28.32.2300397

Fabiani, M., Mateo-Urdiales, A., Sacco, C., Rota, M. C., Petrone, D., Bressi, M., Del Manso, M., Siddu, A., Proietti, V., Battilomo, S., Menniti-Ippolito, F., Popoli, P., Bella, A., Riccardo, F., Palamara, A. T., Rezza, G., Brusaferro, S., Pezzotti, P., & Italian Integrated Surveillance of COVID-19 study group, of the I. C.-19 V. R. group. (2023). Relative effectiveness of a 2nd booster dose of COVID-19 mRNA vaccine up to four months post administration in individuals aged 80 years or more in Italy : A retrospective matched cohort study. Vaccine, 41(1). https://doi.org/10.1016/j.vaccine.2022.11.013

Fabiani, M., Puopolo, M., Filia, A., Sacco, C., Mateo-Urdiales, A., Spila Alegiani, S., Del Manso, M., D'Ancona, F., Vescio, F., Bressi, M., Petrone, D., Spuri, M., Rota, M. C., Massari, M., Da Cas, R., Morciano, C., Stefanelli, P., Bella, A., Tallon, M., … Pezzotti, P. (2022). Effectiveness of an mRNA vaccine booster dose against SARS-CoV-2 infection and severe COVID-19 in persons aged ≥60 years and other high-risk groups

during predominant circulation of the delta variant in Italy, 19 July to 12 December 2021. Expert Review of Vaccines, 21(7). https://doi.org/10.1080/14760584.2022.2064280

Fabiani, M., Puopolo, M., Morciano, C., Spuri, M., Spila Alegiani, S., Filia, A., D'Ancona, F., Del Manso, M., Riccardo, F., Tallon, M., Proietti, V., Sacco, C., Massari, M., Da Cas, R., Mateo-Urdiales, A., Siddu, A., Battilomo, S., Bella, A., Palamara, A. T., … Italian Integrated Surveillance of covid-19 study group and Italian covid-19 Vaccines Registry group. (2022). Effectiveness of mRNA vaccines and waning of protection against SARS-CoV-2 infection and severe covid-19 during predominant circulation of the delta variant in Italy : Retrospective cohort study. BMJ (Clinical Research Ed.), 376. https://doi.org/10.1136/bmj-2021-069052

Fabiani, M., Ramigni, M., Gobbetto, V., Mateo-Urdiales, A., Pezzotti, P., & Piovesan, C. (2021). Effectiveness of the Comirnaty (BNT162b2, BioNTech/Pfizer) vaccine in preventing SARS-CoV-2 infection among healthcare workers, Treviso province, Veneto region, Italy, 27 December 2020 to 24 March 2021. Euro Surveillance: Bulletin Europeen Sur Les Maladies Transmissibles = European Communicable Disease Bulletin, 26(17). https://doi.org/10.2807/1560-7917.ES.2021.26.17.2100420

Fabiani, M., Volpe, E., Faraone, M., Bella, A., Pezzotti, P., & Chini, F. (2020). Effectiveness of influenza vaccine in reducing influenza-associated hospitalizations and deaths among the elderly population; Lazio region, Italy, season 2016-2017. Expert Review of Vaccines, 19(5). https://doi.org/10.1080/14760584.2020.1750380

Fabiani, M., Volpe, E., Faraone, M., Bella, A., Rizzo, C., Marchetti, S., Pezzotti, P., & Chini, F. (2019). Influenza vaccine uptake in the elderly population : Individual and general practitioner's determinants in Central Italy, Lazio region, 2016-2017 season. Vaccine, 37(36). https://doi.org/10.1016/j.vaccine.2019.07.054

Facci, M., & Previti, A. (2022). Characteristics and risk factors for in-hospital mortality in 243 elderly patients with COVID-19 in Santorso, Italy : A retrospective study. Advances in Gerontology = Uspekhi Gerontologii, 35(6). http://www.ncbi.nlm.nih.gov/pubmed/36905586

Falco, A., Piscitelli, P., Vito, D., Pacella, F., Franco, C., Pulimeno, M., Ambrosino, P., Arias, J., & Miani, A. (2023). COVID-19 epidemic spread and green areas Italy and Spain between 2020 and 2021 : An observational multi-country retrospective study. Environmental Research, 216(Pt 1). https://doi.org/10.1016/j.envres.2022.114089

Falcon, M., Rodríguez-Blázquez, C., Romay-Barja, M., Ayala, A., Burgos, A., De Tena-Dávila, M. J., & Forjaz, M. J. (2023). COVID-19 vaccine hesitancy in Spain and associated factors. Frontiers in Public Health, 11. https://doi.org/10.3389/fpubh.2023.1129079

Falcone, M., Suardi, L. R., Tiseo, G., Galfo, V., Occhineri, S., Verdenelli, S., Ceccarelli, G., Poli, M., Merli, M., Bavaro, D., Carretta, A., Nunnari, G., Venanzi Rullo, E., Trecarichi, E. M., Papalini, C., Franco, A., Del Vecchio, R. F., Bianco, V., Punzi, R., … Menichetti, F. (2022). Superinfections caused by carbapenem-resistant Enterobacterales in hospitalized patients with COVID-19 : A multicentre observational study from Italy (CREVID Study). JAC-Antimicrobial Resistance, 4(3). https://doi.org/10.1093/jacamr/dlac064

Fanelli, D., & Piazza, F. (2020). Analysis and forecast of COVID-19 spreading in China, Italy and France. CHAOS SOLITONS & FRACTALS, 134. https://doi.org/10.1016/j.chaos.2020.109761

Fano, V., Coviello, E., Consonni, D., Agresta, A., Orsini, N., Crielesi, A., Miglietta, A. S., Pasqua, C., Vairo, F., Vivaldi, F., De Angelis, G., Colaiocco, G., & Fabiani, M. (2022). COVID-19 vaccines coverage and effectiveness against SARS-CoV-2 infection among residents in the largest Health Authority of Lazio region (Italy) : A population-based cohort study. Expert Review of Vaccines, 21(8). https://doi.org/10.1080/14760584.2022.2080057

Fano, V., Crielesi, A., Coviello, E., Fabiani, M., Salvatore Miglietta, A., Colaiocco, G., Moretti, I., Pasqua, C., Vivaldi, F., De Angelis, G., & Cerimele, M. (2022). Effectiveness of the Comirnaty and the Vaxzevria vaccines in preventing SARS-CoV-2 infection among residents in Lazio region (Italy). Vaccine, 40(18). https://doi.org/10.1016/j.vaccine.2022.02.063

Faranda, D., Alberti, T., Arutkin, M., Lembo, V., & Lucarini, V. (2021). Interrupting vaccination policies can greatly spread SARS-CoV-2 and enhance mortality from COVID-19 disease : The AstraZeneca case for France and Italy. Chaos (Woodbury, N.Y.), 31(4). https://doi.org/10.1063/5.0050887

Fattore, G., Pongiglione, B., & Vezzosi, L. (2024). Excess hospitalizations and in-hospital mortality associated with seasonal influenza in Italy : A 11-year retrospective study. BMC Infectious Diseases, 24(1). https://doi.org/10.1186/s12879-024-09071-z

Faucheux, L., Bassolli de Oliveira Alves, L., Chevret, S., & Rocha, V. (2023). Comparison of characteristics and laboratory tests of COVID-19 hematological patients from France and Brazil during the pre-vaccination period : Identification of prognostic profiles for survival. Hematology, Transfusion and Cell Therapy, 45(3). https://doi.org/10.1016/j.htct.2022.05.003

Faviez, C., Foulquié, P., Chen, X., Mebarki, A., Quennelle, S., Texier, N., Katsahian, S., Schuck, S., & Burgun, A. (2021). Fuzzy Matching for Symptom Detection in Tweets: Application to Covid-19 During the First Wave of the Pandemic in France. Studies in Health Technology and Informatics, 281, 896–900. https://doi.org/10.3233/SHTI210308

Fdez-Arróyabe, P., Marti-Ezpeleta, A., Roye, D., & Zarrabeitia, A. S. (2021). Effects of circulation weather types on influenza hospital admissions in Spain. International Journal of Biometeorology, 65(8). https://doi.org/10.1007/s00484-021-02107-y

Fedrizzi, L., Carugno, M., Consonni, D., Lombardi, A., Bandera, A., Bono, P., Ceriotti, F., Gori, A., & Pesatori, A. C. (2023). Air pollution exposure, SARS-CoV-2 infection, and immune response in a cohort of healthcare workers of a large university hospital in Milan, Italy. Environmental Research, 236(Pt 1). https://doi.org/10.1016/j.envres.2023.116755

Felten, R., Scherlinger, M., Guffroy, A., Poindron, V., Meyer, A., Giannini, M., Korganow, A.-S., Sordet, C., Chatelus, E., Javier, R.-M., Meyer, A., Pijnenburg, L., Kleinmann, J.-F., Gottenberg, J.-E., Sibilia, J., Martin, T., & Arnaud, L. (2021). Incidence and predictors of COVID-19 and flares in patients with rare autoimmune diseases : A systematic survey and serological study at a national reference center in France. Arthritis Research & Therapy, 23(1). https://doi.org/10.1186/s13075-021-02565-0

Feng, Z. (2023). Spatiotemporal pattern of COVID-19 mortality and its relationship with socioeconomic and environmental factors in England. Spatial and Spatio-Temporal Epidemiology, 45. https://doi.org/10.1016/j.sste.2023.100579

Feoli, A., Iannella, A., & Benedetto, E. (2020). Spreading of COVID-19 in Italy as the spreading of a wave packet. EUROPEAN PHYSICAL JOURNAL PLUS, 135(8). https://doi.org/10.1140/epjp/s13360-020-00663-7

Fernández-Cano, M. I., Arreciado Marañón, A., Reyes-Lacalle, A., Feijoo-Cid, M., Manresa-Domínguez, J. M., Montero-Pons, L., Cabedo-Ferreiro, R. M., Toran-Monserrat, P., & Falguera-Puig, G. (2022). Influenza and Pertussis Maternal Vaccination Coverage and Influencing Factors in Spain : A Study Based on Primary Care Records Registry. International Journal of Environmental Research and Public Health, 19(7). https://doi.org/10.3390/ijerph19074391

Fernández-Cooke, E., Grasa, C., Domínguez-Rodríguez, S., Tascón, A., Sánchez-Manubens, J., Anton, J., Mercader, B., Villalobos, E., Camacho, M., Gómez, M., Benavent, M., Giralt, G., Bustillo, M., Naranjo, A., Rocandio, B., Rodríguez-González, M., Cuadros, E., Santos, J., Moreno, D., … KAWA-RACE Study Grp. (2021). Prevalence and Clinical Characteristics of SARS-CoV-2 Confirmed and Negative Kawasaki Disease Patients During the Pandemic in Spain. FRONTIERS IN PEDIATRICS, 8. https://doi.org/10.3389/fped.2020.617039

Fernández-Villa, T., Molina, A. J., Torner, N., Castilla, J., Astray, J., García-Gutiérrez, S., Mayoral, J. M., Tamames, S., Domínguez, Á., Martín, V., & CIBERESP Working Group for the Survey on Influenza Vaccination in Primary Health Care Workers. (2017). Factors associated with acceptance of pandemic flu vaccine by healthcare professionals in Spain, 2009-2010. Research in Nursing & Health, 40(5), 435–443. https://doi.org/10.1002/nur.21815

Ferrando, C., Mellado-Artigas, R., Gea, A., Arruti, E., Aldecoa, C., Bordell, A., Adalia, R., Zattera, L., Ramasco, F., Monedero, P., Maseda, E., Martínez, A., Tamayo, G., Mercadal, J., Muñoz, G., Jacas, A., Ángeles, G., Castro, P., Hernández-Tejero, M., … de la Red de UCI Española para COVID-19. (2020). Patient characteristics, clinical course and factors associated to ICU mortality in critically ill patients infected with SARS-CoV-2 in

Spain : A prospective, cohort, multicentre study. Revista Espanola De Anestesiologia Y Reanimacion, 67(8). https://doi.org/10.1016/j.redar.2020.07.003

Ferrante, P. (2021). The first year of COVID-19 in Italy : Incidence, lethality, and health policies. Journal of Public Health Research, 11(1). https://doi.org/10.4081/jphr.2021.2201

Ferrante, P. (2022). The first 2 years of COVID-19 in Italy : Incidence, lethality, and health policies. Frontiers in Public Health, 10. https://doi.org/10.3389/fpubh.2022.986743

Ferroni, E., Gennaro, N., Barbiellini Amidei, C., Avossa, F., Maifredi, G., Spadea, T., Cacciani, L., Silvestri, C., Bartolini, L., Petrelli, A., Di Napoli, A., Zorzi, M., & Gruppo di lavoro INMP Covid19 e immigrati. (2022). [Impact of COVID-19 on the immigrant population in the Veneto Region (Northern Italy), by geographical area of origin]. Epidemiologia E Prevenzione, 46(4). https://doi.org/10.19191/EP22.4S1.059

Ferroni, E., Giorgi Rossi, P., Spila Alegiani, S., Trifirò, G., Pitter, G., Leoni, O., Cereda, D., Marino, M., Pellizzari, M., Fabiani, M., Riccardo, F., Sultana, J., Massari, M., & ITA-COVID Working Group. (2020). Survival of Hospitalized COVID-19 Patients in Northern Italy : A Population-Based Cohort Study by the ITA-COVID-19 Network. Clinical Epidemiology, 12. https://doi.org/10.2147/CLEP.S271763

Fiasca, F., Minelli, M., Maio, D., Minelli, M., Vergallo, I., Necozione, S., & Mattei, A. (2020). Associations between COVID-19 Incidence Rates and the Exposure to PM2.5 and NO2 : A Nationwide Observational Study in Italy. International Journal of Environmental Research and Public Health, 17(24). https://doi.org/10.3390/ijerph17249318

Figueira Gonçalves, J. M., Hernández Pérez, J. M., Acosta Sorensen, M., Wangüemert Pérez, A. L., Martín Ruiz de la Rosa, E., Trujillo Castilla, J. L., Díaz Pérez, D., & Ramallo-Fariña, Y. (2020). Biomarkers of acute respiratory distress syndrome in adults hospitalised for severe SARS-CoV-2 infection in Tenerife Island, Spain. BMC Research Notes, 13(1). https://doi.org/10.1186/s13104-020-05402-w

Figueras-Aloy, J., Carbonell-Estrany, X., Quero-Jiménez, J., Fernández-Colomer, B., Guzmán-Cabañas, J., Echaniz-Urcelay, I., Doménech-Martínez, E., & IRIS Study Group. (2008). FLIP-2 Study : Risk factors linked to respiratory syncytial virus infection requiring hospitalization in premature infants born in Spain at a gestational age of 32 to 35 weeks. The Pediatric Infectious Disease Journal, 27(9). https://doi.org/10.1097/INF.0b013e3181710990

Figueras-Aloy, J., Carbonell-Estrany, X., Quero-Jiménez, J., Fernández-Colomer, B., Guzmán-Cabañas, J., Echaniz-Urcelay, I., Doménech-Martínez, E., Ripoll, L. M., Monasterolo, R. C., Zaballos, M. F. B., Demestre, X., Bejarano, M. J. H., Calvo, J. L. F., Krauel, X., Millán, L., Gonźalez, J. Q., Perales, A. B., Narbona, E., Echevarría, I., … Aloy, J. F. (2010). Effectiveness of palivizumab to prevent hospitalization for respiratory syncytial virus infection in preterm infants 321 to 35° weeks' gestation in Spain. Acta Pediatrica Espanola, 68(1). Embase. https://www.embase.com/search/results?subaction=viewrecord&id=L358349156&from=export

Filippini, T., Rothman, K. J., Goffi, A., Ferrari, F., Maffeis, G., Orsini, N., & Vinceti, M. (2020). Satellite-detected tropospheric nitrogen dioxide and spread of SARS-CoV-2 infection in Northern Italy. The Science of the Total Environment, 739. https://doi.org/10.1016/j.scitotenv.2020.140278

Filippo, T., Oriana, C., Elena, V., Simone, G., Aleksandra, T., Elena, A., & Alessia, M. (2023). A repeated cross-sectional analysis on the economic impact of SARS-CoV-2 pandemic at the hospital level in Italy. Scientific Reports, 13(1), 12386. https://doi.org/10.1038/s41598-023-39592-7

Fink, N., Rueckel, J., Kaestle, S., Schwarze, V., Gresser, E., Hoppe, B., Rudolph, J., Goller, S., Kunz, W. G., Ricke, J., & Sabel, B. O. (2021). Evaluation of patients with respiratory infections during the first pandemic wave in Germany: Characteristics of COVID-19 versus non-COVID-19 patients. BMC Infectious Diseases, 21(1), 167. https://doi.org/10.1186/s12879-021-05829-x

Finkenzeller, T., Lenhart, S., Reinwald, M., Lüth, S., Dendl, L. M., Paetzel, C., Szczypien, N., Klawonn, F., Von Meyer, A., & Schreyer, A. G. (2021). Risk to Radiology Staff for Occupational COVID-19 Infection in a High-Risk and a Low-Risk Region in Germany : Lessons from the « First Wave ». RoFo: Fortschritte Auf Dem Gebiete Der Rontgenstrahlen Und Der Nuklearmedizin, 193(5). https://doi.org/10.1055/a-1393-6668

Fiorino, S., Carusi, A., Zappi, A., Tateo, F., Peruzzo, L., Zanardi, M., Savelli, F., Di Marzio, G., Cesaretti, S., Dazzani, F., Francesconi, R., Leandri, P., Tortorici, G., Vicari, S., Melucci, D., & Lari, F. (2023). Characteristics, comorbidities and laboratory measures associated with disease severity and poor prognosis in young and elderly patients with COVID-19 admitted to medical wards in Emilia-Romagna region, Italy : A multicentre retrospective study. ITALIAN JOURNAL OF MEDICINE, 17(1). https://doi.org/10.4081/itjm.2023.1608

Firenze, A., Amodio, E., Anastasi, A., Morici, M., Marsala, L., Torregrossa, M., & Romano, N. (2010). Vaccination against the 2009 pandemic influenza A (H1N1) among health-care workers in the major teaching hospital of Sicily (Italy). EUROPEAN JOURNAL OF PUBLIC HEALTH, 20, 207–207.

Fischer, N., Moreels, S., Dauby, N., Reynders, M., Petit, E., Gérard, M., Lacor, P., Daelemans, S., Lissoir, B., Holemans, X., Magerman, K., Jouck, D., Bourgeois, M., Delaere, B., Quoilin, S., Van Gucht, S., Thomas, I., Bossuyt, N., & Barbezange, C. (2023). Influenza versus other respiratory viruses—Assessing severity among hospitalised children, Belgium, 2011 to 2020. Euro Surveillance: Bulletin Europeen Sur Les Maladies Transmissibles = European Communicable Disease Bulletin, 28(29). https://doi.org/10.2807/1560-7917.ES.2023.28.29.2300056

Fléchelles, O., Brissaud, O., Fowler, R., Ducruet, T., Jouvet, P., & The Pediatric Canadian Critical Care Trials Group H N Collaborative And Groupe Francophone de Réa... (2016). Pandemic influenza 2009: Impact of vaccination coverage on critical illness in children, a Canada and France observational study. World Journal of Clinical Pediatrics, 5(4), 374–382. https://doi.org/10.5409/wjcp.v5.i4.374

Flechsler, J., Angermeier, H., Lacroix, S., Schmidt, S., Weber, J., Kasten, D., Paravinja, N., Eberle, U., Heinzinger, S., Konrad, R., Ackermann, N., & Sing, A. (2025). Molecular surveillance of acute respiratory infections (ARIs) in Bavaria, Germany: The Bavarian Influenza + Corona Sentinel (BIS + C). Gesundheitswesen, Supplement, 87, S147–S148. https://doi.org/10.1055/s-0045-1802200

Flisiak, R., Rzymski, P., Zarębska-Michaluk, D., Rogalska, M., Rorat, M., Czupryna, P., Lorenc, B., Ciechanowski, P., Kozielewicz, D., Piekarska, A., Pokorska-Śpiewak, M., Sikorska, K., Tudrujek, M., Bolewska, B., Angielski, G., Kowalska, J., Podlasin, R., Mazur, W., Oczko-Grzesik, B., … Grabowski, H. (2021). Demographic and Clinical Overview of Hospitalized COVID-19 Patients during the First 17 Months of the Pandemic in Poland. Journal of Clinical Medicine, 11(1), 117. https://doi.org/10.3390/jcm11010117

Floridia, M., Giuliano, M., Monaco, M., Palmieri, L., Lo Noce, C., Palamara, A. T., Pantosti, A., Brusaferro, S., Onder, G., Agazio, E., Barbariol, P., Bella, A., Benelli, E., Bertinato, L., Bocci, M., Boros, S., Bressi, M., Calcagnini, G., Canevelli, M., … Weimer, L. E. (2022). Microbiologically confirmed infections and antibiotic-resistance in a national surveillance study of hospitalised patients who died with COVID-19, Italy 2020–2021. Antimicrobial Resistance and Infection Control, 11(1). Embase. https://doi.org/10.1186/s13756-022-01113-y

Flückiger, M., & Ludwig, M. (2023). Spatial networks and the spread of COVID-19 : Results and policy implications from Germany. Jahrbuch Fur Regionalwissenschaftt = Review of Regional Research, 43(1). https://doi.org/10.1007/s10037-023-00185-6

Fogh, K., Eriksen, A. R. R., Larsen, T. G., Hasselbalch, R. B., Bundgaard, H., Scharff, B. F. S. S., Nielsen, S. D., Jørgensen, C. S., Erikstrup, C., Østergaard, L., Ellermann-Eriksen, S., Andersen, B., Nielsen, H., Johansen, I. S., Wiese, L., Hindhede, L., Mikkelsen, S., Sækmose, S. G., Aagaard, B., … Iversen, K. (2023). A Cross-Sectional Study of SARS-CoV-2 Antibodies and Risk Factors for Seropositivity in Staff in Day Care Facilities and Preschools in Denmark. Microbiology Spectrum, 11(1). https://doi.org/10.1128/spectrum.04174-22

Fogh, K., Graakjær Larsen, T., Martel, C. J.-M., Trier Møller, F., Skafte Vestergaard, L., Trebbien, R., Vangsted, A.-M., & Grove Krause, T. (2023). Surveillance of SARS-CoV-2 infection based on self-administered swabs, Denmark, May to July 2022 : Evaluation of a pilot study. Euro Surveillance: Bulletin Europeen Sur Les Maladies Transmissibles = European Communicable Disease Bulletin, 28(38). https://doi.org/10.2807/1560-7917.ES.2023.28.38.2200907

Fogh, K., Strange, J. E., Scharff, B. F. S. S., Eriksen, A. R. R., Hasselbalch, R. B., Bundgaard, H., Nielsen, S. D., Jørgensen, C. S., Erikstrup, C., Norsk, J., Nielsen, P. B., Kristensen, J. H., Østergaard, L., Ellermann-Eriksen, S., Andersen, B., Nielsen, H., Johansen, I. S., Wiese, L., Simonsen, L., … Iversen, K. (2021). Testing Denmark : A

Danish Nationwide Surveillance Study of COVID-19. Microbiology Spectrum, 9(3). https://doi.org/10.1128/Spectrum.01330-21

Fome, A. D., Rodiah, I., Bock, W., Lange, B., & Klar, A. (2024). The Interplay of Influenza and Covid-19 in Germany, January 2020—December 2022 : A Study of Competitive Disease Dynamics with Quarantine Measures and Partial Cross-Immunity. SSRN, (Fome A.D., fome@mathematik.uni-kl.de; Klar A., klar@mathematik.uni-de) Mathematics Department, Rheinland-Pfälzische Technische Universität Kaiserslautern-Landau, Kaiserslautern, Germany. https://doi.org/10.2139/ssrn.5019051

Fong, W. L. E., Nguyen, V. G., Beale, S., Byrne, T. E., Geismar, C., Fragaszy, E., Kovar, J., Navaratnam, A. M. D., Yavlinsky, A., Abubakar, I., Hayward, A. C., & Aldridge, R. W. (2023). Tracking COVID-19 in England and Wales : Insights from Virus Watch—A prospective community cohort study. medRxiv, (Fong W.L.E., erica.fong.16@ucl.ac.uk; Nguyen V.G.; Beale S.; Byrne T.E.; Fragaszy E.; Kovar J.; Navaratnam A.M.D.; Yavlinsky A.; Aldridge R.W.) Institute of Health Informatics, University College London, London, United Kingdom. https://doi.org/10.1101/2023.12.19.23299951

Fonseca, M. J., Hagenaars, S., Bangert, M., Flach, C., & Hudson, R. (2023). Respiratory Syncytial Virus (RSV) Hospital Admission Rates and Patients' Characteristics Before the Age of Two in England, 2015-2019. medRxiv, (Fonseca M.J.) IQVIA, Lisbon, Portugal. https://doi.org/10.1101/2023.04.04.23288132

Fonseca, M. J., Hagenaars, S., Bangert, M., Flach, C., & Hudson, R. D. A. (2024). Respiratory Syncytial Virus Hospital Admission Rates and Patients' Characteristics Before the Age of 2 Years in England, 2015-2019. The Pediatric Infectious Disease Journal, 43(9). https://doi.org/10.1097/INF.0000000000004467

Fonseca-Rodríguez, O., Gustafsson, P. E., San Sebastián, M., & Connolly, A.-M. F. (2021). Spatial clustering and contextual factors associated with hospitalisation and deaths due to COVID-19 in Sweden : A geospatial nationwide ecological study. BMJ Global Health, 6(7). https://doi.org/10.1136/bmjgh-2021-006247

Fontán-Vela, M., Gullón, P., Bilal, U., & Franco, M. (2023). Social and ideological determinants of COVID-19 vaccination status in Spain. Public Health, 219. https://doi.org/10.1016/j.puhe.2023.04.007

Forsberg, G., Taxbro, K., Elander, L., Hanberger, H., Berg, S., Idh, J., Berkius, J., Ekman, A., Hammarskjöld, F., Niward, K., & Balkhed, Å. Ö. (2024). Risk factors for ventilator-associated lower respiratory tract infection in COVID-19, a retrospective multicenter cohort study in Sweden. Acta Anaesthesiologica Scandinavica, 68(2). https://doi.org/10.1111/aas.14338

Fortunato, F., Iannelli, G., Cozza, A., Del Prete, M., Pollidoro, F. V., Cocciardi, S., DI Trani, M., Martinelli, D., & Prato, R. (2018). Local deprivation status and seasonal influenza vaccination coverage in adults ≥ 65 years residing in the Foggia municipality, Italy, 2009-2016. Journal of Preventive Medicine and Hygiene, 59(4 Suppl 2). https://doi.org/10.15167/2421-4248/jpmh2018.59.4s2.1167

Fortunato, F., Lillini, R., Martinelli, D., Iannelli, G., Ascatigno, L., Casanova, G., Lopalco, P. L., & Prato, R. (2023). Association of socio-economic deprivation with COVID-19 incidence and fatality during the first wave of the pandemic in Italy: Lessons learned from a local register-based study. International Journal of Health Geographics, 22(1), 10. https://doi.org/10.1186/s12942-023-00332-9

Fortunato, F., Martinelli, D., Ascatigno, L., Campanozzi, A., Cristiano, G., Iannelli, G., Lauriola, M., Maffei, G., Piano, G., Stea, R., Trerotoli, P., Lopalco, P. L., & Prato, R. (2025). Cost of universal immunization with Nirsevimab vs. Standard of practice for infants in their first RSV season. Foggia District, Italy, 2023-2024. Vaccine, 62, 127550. https://doi.org/10.1016/j.vaccine.2025.127550

Fotakis, E. A., Picasso, E., Sacco, C., Petrone, D., Del Manso, M., Bella, A., Riccardo, F., Odone, A., Cannone, A., Tallon, M., De Angelis, L., Sciurti, A., Cescutti, D., Pezzotti, P., Fabiani, M., Mateo-Urdiales, A., & Italian Integrated Surveillance of COVID-19 study group and of the Italian COVID-19 Vaccines Registry group. (2024). Impact of the 2023/24 autumn-winter COVID-19 seasonal booster campaign in preventing severe COVID-19 cases in Italy (October 2023-March 2024). Vaccine, 42(26). https://doi.org/10.1016/j.vaccine.2024.126375

Fouillet, A., Pontais, I., & Caserio-Schönemann, C. (2020). Excess all-cause mortality during the first wave of the COVID-19 epidemic in France, March to May 2020. Euro Surveillance: Bulletin Europeen Sur Les Maladies

Transmissibles = European Communicable Disease Bulletin, 25(34). https://doi.org/10.2807/1560-7917.ES.2020.25.34.2001485

Fourgeaud, J., Toubiana, J., Chappuy, H., Delacourt, C., Moulin, F., Parize, P., Scemla, A., Abid, H., Leruez-Ville, M., & Frange, P. (2021). Impact of public health measures on the post-COVID-19 respiratory syncytial virus epidemics in France. European Journal of Clinical Microbiology & Infectious Diseases: Official Publication of the European Society of Clinical Microbiology, 40(11). https://doi.org/10.1007/s10096-021-04323-1

Fragaszy, E. B., Warren-Gash, C., White, P. J., Zambon, M., Edmunds, W. J., Nguyen-Van-Tam, J. S., Hayward, A. C., & Flu Watch Group. (2018). Effects of seasonal and pandemic influenza on health-related quality of life, work and school absence in England: Results from the Flu Watch cohort study. Influenza and Other Respiratory Viruses, 12(1), 171–182. https://doi.org/10.1111/irv.12506

Franceschi, J., Pareschi, L., Bellodi, E., Gavanelli, M., & Bresadola, M. (2023). Modeling opinion polarization on social media : Application to Covid-19 vaccination hesitancy in Italy. PloS One, 18(10). https://doi.org/10.1371/journal.pone.0291993

Francetic, I., & Munford, L. (2021). Corona and coffee on your commute : A spatial analysis of COVID-19 mortality and commuting flows in England in 2020. European Journal of Public Health, 31(4). https://doi.org/10.1093/eurpub/ckab072

Friesema, I. H. M., Koppeschaar, C. E., Donker, G. A., Dijkstra, F., van Noort, S. P., Smallenburg, R., van der Hoek, W., & van der Sande, M. a. B. (2009). Internet-based monitoring of influenza-like illness in the general population : Experience of five influenza seasons in The Netherlands. Vaccine, 27(45). https://doi.org/10.1016/j.vaccine.2009.05.042

Friis, N. U., Martin-Bertelsen, T., Pedersen, R. K., Nielsen, J., Krause, T. G., Andreasen, V., & Vestergaard, L. S. (2023). COVID-19 mortality attenuated during widespread Omicron transmission, Denmark, 2020 to 2022. Euro Surveillance: Bulletin Europeen Sur Les Maladies Transmissibles = European Communicable Disease Bulletin, 28(3). https://doi.org/10.2807/1560-7917.ES.2023.28.3.2200547

Fritsch, A., Schweiger, B., & Biere, B. (2019). Influenza C virus in pre-school children with respiratory infections : Retrospective analysis of data from the national influenza surveillance system in Germany, 2012 to 2014. Euro Surveillance: Bulletin Europeen Sur Les Maladies Transmissibles = European Communicable Disease Bulletin, 24(10). https://doi.org/10.2807/1560-7917.ES.2019.24.10.1800174

Fritz, C., & Kauermann, G. (2022). On the interplay of regional mobility, social connectedness and the spread of COVID-19 in Germany. Journal of the Royal Statistical Society. Series A, (Statistics in Society), 185(1). https://doi.org/10.1111/rssa.12753

Fröhlich, G. M., Jeschke, E., Eichler, U., Thiele, H., Alhariri, L., Reinthaler, M., Kastrati, A., Leistner, D. M., Skurk, C., Landmesser, U., & Günster, C. (2021). Impact of oral anticoagulation on clinical outcomes of COVID-19 : A nationwide cohort study of hospitalized patients in Germany. Clinical Research in Cardiology: Official Journal of the German Cardiac Society, 110(7). https://doi.org/10.1007/s00392-020-01783-x

Fuentes-Alonso, M., Jimenez-Garcia, R., Lopez-de-Andres, A., Zamorano-Leon, J. J., Carabantes-Alarcon, D., Jimenez-Trujillo, I., Sanz-Rojo, S., & de Miguel-Diez, J. (2022). Time Trends (2012-2020), Sex Differences and Predictors for Influenza Vaccination Uptake among Individuals with Chronic Obstructive Pulmonary Disease in Spain. Journal of Clinical Medicine, 11(5). https://doi.org/10.3390/jcm11051423

Führer, A., Pacolli, L., Yilmaz-Aslan, Y., & Brzoska, P. (2022). COVID-19 Vaccine Acceptance and Its Determinants among Migrants in Germany-Results of a Cross-Sectional Study. Vaccines, 10(8). https://doi.org/10.3390/vaccines10081350

Fuhrman, C., Bonmarin, I., Paty, A., Duport, N., Chiron, E., Lucas, E., Bitar, D., Mailles, A., Herida, M., Vaux, S., & Lévy-Bruhl, D. (2010). Severe hospitalised 2009 pandemic influenza A(H1N1) cases in France, 1 July-15 November 2009. EUROSURVEILLANCE, 15(2), 17–21.

Fusco, F. M., Pisaturo, M., Iodice, V., Bellopede, R., Tambaro, O., Parrella, G., Di Flumeri, G., Viglietti, R., Pisapia, R., Carleo, M. A., Boccardi, M., Atripaldi, L., Chignoli, B., Maturo, N., Rescigno, C., Esposito, V.,

Dell'Aversano, R., Sangiovanni, V., & Punzi, R. (2020). COVID-19 among healthcare workers in a specialist infectious diseases setting in Naples, Southern Italy : Results of a cross-sectional surveillance study. The Journal of Hospital Infection, 105(4). https://doi.org/10.1016/j.jhin.2020.06.021

Gabellone, V., Nuccetelli, F., Gabrielli, E., Ascatigno, L., Lopalco, P. L., & Prato, R. (2025). The World Café as a Tool for Evaluating the Level of Acceptance of SARS-CoV-2 Screening in School Settings, Puglia Region, Italy, 2023. Health Expectations: An International Journal of Public Participation in Health Care and Health Policy, 28(1). https://doi.org/10.1111/hex.70137

Gabet, A., Grave, C., Chatignoux, E., Tuppin, P., Béjot, Y., & Olié, V. (2021). Characteristics, Management, and Case-Fatality of Patients Hospitalized for Stroke with a Diagnosis of COVID-19 in France. Neuroepidemiology, 55(4). https://doi.org/10.1159/000516670

Galante, M., Garin, O., Sicuri, E., Cots, F., García-Altés, A., Ferrer, M., Dominguez, À., & Alonso, J. (2012). Health services utilization, work absenteeism and costs of pandemic influenza A (H1N1) 2009 in Spain: A multicenter-longitudinal study. PloS One, 7(2), e31696. https://doi.org/10.1371/journal.pone.0031696

Galiero, R., Loffredo, G., Simeon, V., Caturano, A., Vetrano, E., Medicamento, G., Alfano, M., Beccia, D., Brin, C., Colantuoni, S., Di Salvo, J., Epifani, R., Nevola, R., Marfella, R., Sardu, C., Coppola, C., Scarano, F., Maggi, P., Calabrese, C., … COVOCA Study Group. (2024). Impact of liver fibrosis on COVID-19 in-hospital mortality in Southern Italy. PloS One, 19(5). https://doi.org/10.1371/journal.pone.0296495

Galiero, R., Simeon, V., Loffredo, G., Caturano, A., Rinaldi, L., Vetrano, E., Medicamento, G., Alfano, M., Beccia, D., Brin, C., Colantuoni, S., Di Salvo, J., Epifani, R., Nevola, R., Marfella, R., Sardu, C., Coppola, C., Scarano, F., Maggi, P., … On Behalf Of Covoca Study Group, null. (2022). Association between Renal Function at Admission and COVID-19 in-Hospital Mortality in Southern Italy : Findings from the Prospective Multicenter Italian COVOCA Study. Journal of Clinical Medicine, 11(20). https://doi.org/10.3390/jcm11206121

Gallè, F., Sabella, E. A., Roma, P., Da Molin, G., Diella, G., Montagna, M. T., Ferracuti, S., Liguori, G., Orsi, G. B., & Napoli, C. (2021). Acceptance of COVID-19 Vaccination in the Elderly : A Cross-Sectional Study in Southern Italy. Vaccines, 9(11). https://doi.org/10.3390/vaccines9111222

Gallè, F., Sabella, E. A., Roma, P., De Giglio, O., Caggiano, G., Tafuri, S., Da Molin, G., Ferracuti, S., Montagna, M. T., Liguori, G., Orsi, G. B., & Napoli, C. (2021). Knowledge and Acceptance of COVID-19 Vaccination among Undergraduate Students from Central and Southern Italy. Vaccines, 9(6). https://doi.org/10.3390/vaccines9060638

Gallego-García, P., Hong, S. L., Bollen, N., Dellicour, S., Baele, G., Suchard, M. A., Lemey, P., & Posada, D. (2025). International importance and spread of SARS-CoV-2 variants Alpha, Delta, and Omicron BA.1 into Spain. Communications Medicine, 5(1), 209. https://doi.org/10.1038/s43856-025-00912-9

Galli, M. G., Djuric, O., Besutti, G., Ottone, M., Amidei, L., Bitton, L., Bonilauri, C., Boracchia, L., Campanale, S., Curcio, V., Lucchesi, D. M. F., Mulas, C. S., Santi, F., Ferrari, A. M., Giorgi Rossi, P., Luppi, F., & Reggio Emilia COVID-19 Working Group. (2022). Clinical and imaging characteristics of patients with COVID-19 predicting hospital readmission after emergency department discharge : A single-centre cohort study in Italy. BMJ Open, 12(4). https://doi.org/10.1136/bmjopen-2021-052665

Gallo, E., Prosepe, I., Lorenzoni, G., Acar, A. Ş., Lanera, C., Berchialla, P., Azzolina, D., & Gregori, D. (2021). Excess of all-cause mortality is only partially explained by COVID-19 in Veneto (Italy) during spring outbreak. BMC Public Health, 21(1). https://doi.org/10.1186/s12889-021-10832-7

Gallus, S., Bosetti, C., Gorini, G., Stival, C., Boffi, R., Lugo, A., Carreras, G., Veronese, C., Santucci, C., Pacifici, R., Tinghino, B., Zagà, V., Russo, P., Cattaruzza, M. S., & COSMO-IT Investigators. (2023). The Association of Tobacco Smoking, Second-hand Smoke, and Novel Tobacco Products With COVID-19 Severity and Mortality in Italy : Results From the COSMO-IT Study. Journal of Epidemiology, 33(7). https://doi.org/10.2188/jea.JE20220321

Gámbaro, F., Behillil, S., Baidaliuk, A., Donati, F., Albert, M., Alexandru, A., Vanpeene, M., Bizard, M., Brisebarre, A., Barbet, M., Derrar, F., van der Werf, S., Enouf, V., & Simon-Loriere, E. (2020). Introductions and early spread of SARS-CoV-2 in France, 24 January to 23 March 2020. Euro Surveillance: Bulletin Europeen

Sur Les Maladies Transmissibles = European Communicable Disease Bulletin, 25(26). https://doi.org/10.2807/1560-7917.ES.2020.25.26.2001200

Ganczak, M., Kalinowski, P., Twardowski, P., Osicka, D. A., Pasek, O., Duda-Duma, Ł., Vervoort, J. P. M., Edelstein, M., & Kowalska, M. (2024). « Why would we? » A qualitative study on COVID-19 vaccination decision making among Ukrainian economic female migrants in Poland. Frontiers in Public Health, 12. https://doi.org/10.3389/fpubh.2024.1380627

Gandjour, A. (2022). The clinical and economic value of a successful shutdown during the SARS-CoV-2 pandemic in Germany. The Quarterly Review of Economics and Finance: Journal of the Midwest Economics Association, 84, 502–509. https://doi.org/10.1016/j.qref.2020.10.007

Ganem, F., Alonso, L., Colom-Cadena, A., Bordas, A., Folch, C., Soriano-Arandes, A., & Casabona, J. (2023). Syndromic surveillance as a predictive tool for health-related school absences in COVID-19 Sentinel Schools in Catalonia, Spain. medRxiv, (Ganem F.; Alonso L.; Colom-Cadena A.; Bordas A.; Folch C., cfolch@iconcologia.net; Casabona J.) Centre d'Estudis Epidemiològics Sobre les ITS i Sida de Catalunya (CEEISCAT), Badalona, Spain. https://doi.org/10.1101/2023.03.24.23287681

Ganem, F., Folch, C., Colom-Cadena, A., Bordas, A., Alonso, L., Soriano-Arandes, A., Casabona, J., & Sentinel School Network Study Group of Catalonia. (2023). Determinants of COVID-19 vaccine hesitancy among students and parents in Sentinel Schools Network of Catalonia, Spain. PloS One, 18(3). https://doi.org/10.1371/journal.pone.0282871

Ganser, I., Buckeridge, D. L., Heffernan, J., Prague, M., & Thiébaut, R. (2024). Estimating the population effectiveness of interventions against COVID-19 in France : A modelling study. Epidemics, 46. https://doi.org/10.1016/j.epidem.2024.100744

García de Guadiana-Romualdo, L., Morell-García, D., Morales-Indiano, C., Bauça, J. M., Alcaide Martín, M. J., Esparza Del Valle, C., Gutiérrez Revilla, J. I., Urrechaga, E., Álamo, J. M., Hernando Holgado, A. M., Lorenzo-Lozano, M.-C., Sánchez Fdez-Pacheco, S., de la Hera Cagigal, P., Juncos Tobarra, M. A., Vílchez, J. A., Vírseda Chamorro, I., Gutiérrez Garcia, I., Pastor Murcia, Y., Sahuquillo Frías, L., … Galán Ortega, A. (2021). Characteristics and laboratory findings on admission to the emergency department among 2873 hospitalized patients with COVID-19 : The impact of adjusted laboratory tests in multicenter studies. A multicenter study in Spain (BIOCOVID-Spain study). Scandinavian Journal of Clinical and Laboratory Investigation, 81(3). https://doi.org/10.1080/00365513.2021.1881997

García Gómez, M., Gherasim, A. M., Roldán Romero, J. M., Zimmermann Verdejo, M., Monge Corella, S., Sierra Moros, M. J., & Montoya Martínez, L. M. (2022). [The impact of COVID-19 on sick leave of the persons working in nursing homes in Spain. Usefulness of sick leave for the surveillance of the pandemic evolution.]. Revista Espanola De Salud Publica, 96, e202204038.

Garcia, E., Sanchez-Rodriguez, D., Levy, S., Claessens, M., Van Hauwermeiren, C., Taliha, M., Benoit, F., & Surquin, M. (2022). [Factors associated with intrahospital mortality in older patients with COVID-19 in Belgium : The COVID-AgeBru study]. Revue Medicale De Liege, 77(3). http://www.ncbi.nlm.nih.gov/pubmed/35258862

Garcia-Agundez, A., Ojo, O., Hernández-Roig, H. A., Baquero, C., Frey, D., Georgiou, C., Goessens, M., Lillo, R. E., Menezes, R., Nicolaou, N., Ortega, A., Stavrakis, E., & Fernandez Anta, A. (2021). Estimating the COVID-19 Prevalence in Spain With Indirect Reporting via Open Surveys. Frontiers in Public Health, 9. https://doi.org/10.3389/fpubh.2021.658544

García-García, D., Herranz-Hernández, R., Rojas-Benedicto, A., León-Gómez, I., Larrauri, A., Peñuelas, M., Guerrero-Vadillo, M., Ramis, R., & Gómez-Barroso, D. (2022). Assessing the effect of non-pharmaceutical interventions on COVID-19 transmission in Spain, 30 August 2020 to 31 January 2021. Euro Surveillance: Bulletin Europeen Sur Les Maladies Transmissibles = European Communicable Disease Bulletin, 27(19). https://doi.org/10.2807/1560-7917.ES.2022.27.19.2100869

García-Peña, C., Molina, J., & Sinoga, J. (2023). Learning About the Incidence and Lethality of COVID-19 in Vulnerable Neighborhoods: The Case of Malaga (Spain). INTERNATIONAL REGIONAL SCIENCE REVIEW, 46(5), 701–725. https://doi.org/10.1177/01600176221145879

Garcia-Vidal, C., Cózar-Llistó, A., Meira, F., Dueñas, G., Puerta-Alcalde, P., Cilloniz, C., Garcia-Pouton, N., Chumbita, M., Cardozo, C., Hernández, M., Rico, V., Bodro, M., Morata, L., Castro, P., Almuedo-Riera, A., García, F., Mensa, J., Antonio Martínez, J., Sanjuan, G., … COVID-19-researcher group. (2021). Trends in mortality of hospitalised COVID-19 patients : A single centre observational cohort study from Spain. The Lancet Regional Health. Europe, 3. https://doi.org/10.1016/j.lanepe.2021.100041

Garenne, M., & Stiegler, N. (2024). Covid-19 demography in France and South Africa : A comparative study of morbidity and mortality in 2020-2022. PloS One, 19(2). https://doi.org/10.1371/journal.pone.0294870

Garjani, A., Patel, S., Bharkhada, D., Rashid, W., Coles, A., Law, G. R., & Evangelou, N. (2022). Impact of mass vaccination on SARS-CoV-2 infections among multiple sclerosis patients taking immunomodulatory disease-modifying therapies in England. Multiple Sclerosis and Related Disorders, 57. https://doi.org/10.1016/j.msard.2021.103458

Garlantezec, R., Tadié, E., Heslan, C., Gary-Bobo, P., Oumari, S., Saade, A., Sitruk, A., Tattevin, P., Thibault, V., Paris, C., & AntiCOV-HB* working group. (2022). SARS-CoV-2 seroprevalence and antibodies persistence among health care workers after the first COVID-19 wave in nine hospitals in Western France. Infectious Diseases Now, 52(8). https://doi.org/10.1016/j.idnow.2022.09.004

Garralda Fernandez, J., Molero Vilches, I., Bermejo Rodríguez, A., Cano Torres, I., Colino Romay, E. I., García Arata, I., Jaqueti Aroca, J., Lillo Rodríguez, R., López Lacomba, D., Mazón Cuadrado, L., Molina Esteban, L., Morales García, L. J., Moratilla Monzo, L., Nieto-Borrajo, E., Pacheco Delgado, M., Prieto Menchero, S., Sánchez Hernández, C., Sánchez Testillano, E., & García-Martínez, J. (2021). Impact of SARS-CoV-2 pandemic among health care workers in a secondary teaching hospital in Spain. PloS One, 16(1), e0245001. https://doi.org/10.1371/journal.pone.0245001

Garrido, J., Martínez-Rodríguez, D., Rodríguez-Serrano, F., Sferle, S., & Villanueva, R. (2021). Modeling COVID-19 with Uncertainty in Granada, Spain. Intra-Hospitalary Circuit and Expectations over the Next Months. MATHEMATICS, 9(10). https://doi.org/10.3390/math9101132

Garzaro, G., Clari, M., Ciocan, C., Grillo, E., Mansour, I., Godono, A., Borgna, L. G., Sciannameo, V., Costa, G., Raciti, I. M., Bert, F., Berchialla, P., Coggiola, M., & Pira, E. (2020). COVID-19 infection and diffusion among the healthcare workforce in a large university-hospital in northwest Italy. La Medicina Del Lavoro, 111(3). https://doi.org/10.23749/mdl.v111i3.9767

Gasparini, R., Bonanni, P., Amicizia, D., Bella, A., Donatelli, I., Cristina, M. L., Panatto, D., & Lai, P. L. (2013). Influenza epidemiology in Italy two years after the 2009-2010 pandemic: Need to improve vaccination coverage. Human Vaccines & Immunotherapeutics, 9(3), 561–567. https://doi.org/10.4161/hv.23235

Gasparini, R., Lucioni, C., Ansaldi, F., Durando, P., Sticchi, L., Icardi, G., Panatto, D., Martin, M., Chancellor, J., & Aballéa, S. (2007). Cost-effectiveness study of influenza vaccination for people aged 50-64 years in Italy. PharmacoEconomics - Italian Research Articles, 9(2). Embase. https://doi.org/10.1007/bf03320703

Gates, P., Noakes, K., Begum, F., Pebody, R., & Salisbury, D. (2009). Collection of routine national seasonal influenza vaccine coverage data from GP practices in England using a web-based collection system. Vaccine, 27(48). https://doi.org/10.1016/j.vaccine.2009.08.094

Gatto, A., Accarino, G., Aloisi, V., Immorlano, F., Donato, F., & Aloisio, G. (2021). Limits of Compartmental Models and New Opportunities for Machine Learning : A Case Study to Forecast the Second Wave of COVID-19 Hospitalizations in Lombardy, Italy. INFORMATICS-BASEL, 8(3). https://doi.org/10.3390/informatics8030057

Gatto, A., Aloisi, V., Accarino, G., Immorlano, F., Chiarelli, M., & Aloisio, G. (2022). An Artificial Neural Network-Based Approach for Predicting the COVID-19 Daily Effective Reproduction Number Rt in Italy. AI, 3(1). https://doi.org/10.3390/ai3010009

Gatto, M., Bertuzzo, E., Mari, L., Miccoli, S., Carraro, L., Casagrandi, R., & Rinaldo, A. (2020). Spread and dynamics of the COVID-19 epidemic in Italy : Effects of emergency containment measures. Proceedings of the National Academy of Sciences of the United States of America, 117(19). https://doi.org/10.1073/pnas.2004978117

Gaubert, G., Nauleau, S., Franke, F., Rebaudet, S., Mosnier, E., Landier, J., Chaud, P., Malfait, P., Vandentorren, S., Huart, M., Ramdani, A., Bendiane, M.-K., Danjou, F., & Gaudart, J. (2023). Deprivation effect on COVID-19 cases incidence and severity : A geo-epidemiological study in PACA region, France. medRxiv, (Gaubert G., guillaume.gaubert@hotmail.fr) Aix Marseille Univ, SESSTIM, ISSPAM, Marseille, France. https://doi.org/10.1101/2023.04.18.23288723

Gaudouen, H., Tattevin, P., Thibault, V., Ménard, G., Paris, C., & Saade, A. (2024). Determinants of influenza and COVID vaccine uptake in healthcare workers: A cross-sectional survey during the post-pandemic era in a network of academic hospitals in France. Vaccine, 42(26), 126272. https://doi.org/10.1016/j.vaccine.2024.126272

Gaughan, C. H., Razieh, C., Khunti, K., Banerjee, A., Chudasama, Y. V., Davies, M. J., Dolby, T., Gillies, C. L., Lawson, C., Mirkes, E. M., Morgan, J., Tingay, K., Zaccardi, F., Yates, T., & Nafilyan, V. (2023). COVID-19 vaccination uptake amongst ethnic minority communities in England : A linked study exploring the drivers of differential vaccination rates. Journal of Public Health (Oxford, England), 45(1). https://doi.org/10.1093/pubmed/fdab400

Gavazzi, G., Paccalin, M., Berkovitch, Q., Leleu, H., Moreau, R., Ciglia, E., Burlet, N., & Mould-Quevedo, J. (2024). Cost-effectiveness of cell-based influenza vaccine in France. Expert Review of Vaccines, 23(1). https://doi.org/10.1080/14760584.2024.2417854

Gavazzi, G., Wazieres, B., Lejeune, B., Rothan-Tondeur, M., & Observ Risque Infect Geriatrie. (2007). Influenza and pneumococcal vaccine coverages in geriatric health care settings in France. GERONTOLOGY, 53(6). https://doi.org/10.1159/000105166

Gea-Izquierdo, E., Gil-Prieto, R., Hernández-Barrera, V., & Gil-de-Miguel, Á. (2023). Respiratory syncytial virus-associated hospitalization in children aged <2 years in Spain from 2018 to 2021. Human Vaccines & Immunotherapeutics, 19(2). https://doi.org/10.1080/21645515.2023.2231818

Gefenaite, G., Pistol, A., Popescu, R., Popovici, O., Ciurea, D., Dolk, C., Jit, M., & Gross, D. (2018). Estimating burden of influenza-associated influenza-like illness and severe acute respiratory infection at public healthcare facilities in Romania during the 2011/12-2015/16 influenza seasons. Influenza and Other Respiratory Viruses, 12(1), 183–192. https://doi.org/10.1111/irv.12525

Gehrau, V., Fujarski, S., Lorenz, H., Schieb, C., & Blöbaum, B. (2021). The Impact of Health Information Exposure and Source Credibility on COVID-19 Vaccination Intention in Germany. International Journal of Environmental Research and Public Health, 18(9). https://doi.org/10.3390/ijerph18094678

Gémes, K., Talbäck, M., Modig, K., Ahlbom, A., Berglund, A., Feychting, M., & Matthews, A. A. (2020). Burden and prevalence of prognostic factors for severe COVID-19 in Sweden. European Journal of Epidemiology, 35(5). https://doi.org/10.1007/s10654-020-00646-z

Generaal, E., van Santen, D. K. D., Campman, S. L., Booij, M. J., Price, D., Buster, M., van Dijk, C., Boyd, A., Bruisten, S. M., van Dam, A. P., van der Lubben, M., van Duijnhoven, Y. T. H. P., & Prins, M. (2023). Low prevalence of current and past SARS-CoV-2 infections among visitors and staff members of homelessness services in Amsterdam at the end of the second wave of infections in the Netherlands. PloS One, 18(7). https://doi.org/10.1371/journal.pone.0288610

Genovese, C., Biondo, C., Rizzo, C., Cortese, R., La Spina, I., Tripodi, P., Romeo, B., La Fauci, V., Trimarchi, G., Prete, V., & Squeri, R. (2023). Knowledge, Propensity and Hesitancy among Pregnant Women in the Post-Pandemic Phase Regarding COVID-19 Vaccination: A Prevalence Survey in Southern Italy. WOMEN, 3(3), 374–384. https://doi.org/10.3390/women3030028

Gentile, I., Iorio, M., Zappulo, E., Scotto, R., Maraolo, A. E., Buonomo, A. R., Pinchera, B., Muto, G., Iervolino, C., Villari, R., Schiano Moriello, N., Scirocco, M. M., Triassi, M., Paternoster, M., Russo, V., Viceconte, G., & Federico Ii Covid-Team, null. (2022). COVID-19 Post-Exposure Evaluation (COPE) Study : Assessing the Role of Socio-Economic Factors in Household SARS-CoV-2 Transmission within Campania Region (Southern Italy). International Journal of Environmental Research and Public Health, 19(16). https://doi.org/10.3390/ijerph191610262

Gerardo, R.-M., Guiomar, H.-G., Ander, G.-S., Carlos Andrés, G.-C., & Coral, A.-C. (2024). Dynamics of endemic human coronavirus and SARS-CoV-2 in a hospital of Madrid, Spain. Retrospective study from June 2020 to July 2023. APMIS: Acta Pathologica, Microbiologica, et Immunologica Scandinavica, 132(9). https://doi.org/10.1111/apm.13446

Gerbaud, L., Guiguet-Auclair, C., Breysse, F., Odoul, J., Ouchchane, L., Peterschmitt, J., Dezfouli-Desfer, C., & Breton, V. (2020). Hospital and Population-Based Evidence for COVID-19 Early Circulation in the East of France. International Journal of Environmental Research and Public Health, 17(19). https://doi.org/10.3390/ijerph17197175

Gerlee, P., Karlsson, J., Fritzell, I., Brezicka, T., Spreco, A., Timpka, T., Jöud, A., & Lundh, T. (2021). Predicting regional COVID-19 hospital admissions in Sweden using mobility data. Scientific Reports, 11(1). https://doi.org/10.1038/s41598-021-03499-y

Gerlier, L., Lamotte, M., Dos Santos Mendes, S., Damm, O., Schwehm, M., & Eichner, M. (2016). Estimates of the Public Health Impact of a Pediatric Vaccination Program Using an Intranasal Tetravalent Live-Attenuated Influenza Vaccine in Belgium. Paediatric Drugs, 18(4). https://doi.org/10.1007/s40272-016-0180-6

Gerlier, L., Lamotte, M., Grenèche, S., Lenne, X., Carrat, F., Weil-Olivier, C., Damm, O., Schwehm, M., & Eichner, M. (2017). Assessment of Public Health and Economic Impact of Intranasal Live-Attenuated Influenza Vaccination of Children in France Using a Dynamic Transmission Model. Applied Health Economics and Health Policy, 15(2). https://doi.org/10.1007/s40258-016-0296-4

Gerna, G., Percivalle, E., Piralla, A., Rognoni, V., Marchi, A., & Baldanti, F. (2009). Surveillance of influenza virus B circulation in Northern Italy : Summer-fall 2008 isolation of three strains and phylogenetic analysis. The New Microbiologica, 32(4). http://www.ncbi.nlm.nih.gov/pubmed/20128448

Gervasi, F., Andreano, A., & Russo, A. G. (2021). Metabolic syndrome and risk of COVID-19-related hospitalization : A large, population-based cohort study carried out during the first European outbreak of SARS-CoV-2 infection in the Metropolitan area of Milan (Lombardy Region, Northern Italy). Epidemiologia E Prevenzione, 45(6). https://doi.org/10.19191/EP21.6.115

Gerver, S. M., Guy, R., Wilson, K., Thelwall, S., Nsonwu, O., Rooney, G., Brown, C. S., Muller-Pebody, B., Hope, R., & Hall, V. (2021). National surveillance of bacterial and fungal coinfection and secondary infection in COVID-19 patients in England : Lessons from the first wave. Clinical Microbiology and Infection: The Official Publication of the European Society of Clinical Microbiology and Infectious Diseases, 27(11). https://doi.org/10.1016/j.cmi.2021.05.040

Gessler, N., Gunawardene, M. A., Wohlmuth, P., Arnold, D., Behr, J., Gloeckner, C., Herrlinger, K., Hoelting, T., Pape, U.-F., Schreiber, R., Stang, A., Wesseler, C., Willems, S., Arms, C., & Herborn, C. U. (2021). Clinical outcome, risk assessment, and seasonal variation in hospitalized COVID-19 patients-Results from the CORONA Germany study. PloS One, 16(6). https://doi.org/10.1371/journal.pone.0252867

Ghebrehewet, S., Stewart, A. G., & MacPherson, P. (2024). A natural experiment during lockdown and on-going care-home COVID-19 outbreaks showed a single dose of vaccine reduced hospitalisation and deaths of care-home residents in North West England. Public Health, 233. https://doi.org/10.1016/j.puhe.2024.05.002

Gherasim, A., Martínez-Baz, I., Castilla, J., Pozo, F., Larrauri, A., Jimenez Jorge, S., De Mateo, S., Sanz, C. D., Domínguez, J. A. O., Savulescu, C., Casas, I., García-Cenoz, M., Arotzena, J. M. A., Martinez, R. S., Etxebarriarteun-Aranzabal, L., Gamarra, I. A., Carril, F. G., Berga, J. M. V., Durán, J. G., … Aceitero, J. M. R. (2017). Effect of previous and current vaccination against influenza A(H1N1)pdm09, A(H3N2), and B during the post-pandemic period 2010-2016 in Spain. PLoS ONE, 12(6).nbn https://doi.org/10.1371/journal.pone.0179160

Ghio, S., Baldi, E., Vicentini, A., Lenti, M. V., Di Sabatino, A., Di Matteo, A., Zuccaro, V., Piloni, D., Corsico, A., Gnecchi, M., Speciale, F., Sabena, A., Oltrona Visconti, L., Perlini, S., & San Matteo COVID Cardiac Injury Task Force. (2020). Cardiac involvement at presentation in patients hospitalized with COVID-19 and their outcome in a tertiary referral hospital in Northern Italy. Internal and Emergency Medicine, 15(8). https://doi.org/10.1007/s11739-020-02493-y

Ghosh, J., Saha, P., Kamrujjaman, M., & Ghosh, U. (2023). Transmission Dynamics of COVID-19 with Saturated Treatment : A Case Study of Spain. BRAZILIAN JOURNAL OF PHYSICS, 53(3). https://doi.org/10.1007/s13538-023-01267-z

Giacomelli, A., Ridolfo, A. L., Bonazzetti, C., Oreni, L., Conti, F., Pezzati, L., Siano, M., Bassoli, C., Casalini, G., Schiuma, M., Covizzi, A., Passerini, M., Piscaglia, M., Borgonovo, F., Galbiati, C., Colombo, R., Catena, E., Rizzardini, G., Milazzo, L., … Antinori, S. (2022). Mortality among Italians and immigrants with COVID-19 hospitalised in Milan, Italy : Data from the Luigi Sacco Hospital registry. BMC Infectious Diseases, 22(1). https://doi.org/10.1186/s12879-022-07051-9

Giacomelli, A., Ridolfo, A. L., Pezzati, L., Oreni, L., Carrozzo, G., Beltrami, M., Poloni, A., Caloni, B., Lazzarin, S., Colombo, M., Pozza, G., Pagano, S., Caronni, S., Fusetti, C., Gerbi, M., Petri, F., Borgonovo, F., D'Aloia, F., Negri, C., … Antinori, S. (2022). Mortality rates among COVID-19 patients hospitalised during the first three waves of the epidemic in Milan, Italy : A prospective observational study. PloS One, 17(4). https://doi.org/10.1371/journal.pone.0263548

Giamberardino, P. D., Iacoviello, D., Papa, F., & Sinisgalli, C. (2021). Dynamical evolution of COVID-19 in Italy with an evaluation of the size of the asymptomatic infective population. IEEE Journal of Biomedical and Health Informatics, 25(4). Embase. https://doi.org/10.1109/JBHI.2020.3009038

Gianola, S., Bargeri, S., Campanini, I., Corbetta, D., Gambazza, S., Innocenti, T., Meroni, R., Castellini, G., Turolla, A., & Scientific Committee of AIFI. (2021). The Spread of COVID-19 Among 15,000 Physical Therapists in Italy : A Cross-Sectional Study. Physical Therapy, 101(8). https://doi.org/10.1093/ptj/pzab123

Gianquintieri, L., Pagliosa, A., Bonora, R., & Caiani, E. (2025). A High-Granularity, Machine Learning Informed Spatial Predictive Model for Epidemic Monitoring: The Case of COVID-19 in Lombardy Region, Italy. APPLIED SCIENCES-BASEL, 15(15). https://doi.org/10.3390/app15158729

Giansante, C., Stivanello, E., Perlangeli, V., Ferretti, F., Marzaroli, P., Musti, M. A., Pizzi, L., Resi, D., Saraceni, S., & Pandolfi, P. (2021). COVID-19 vaccine effectiveness among the staff of the Bologna Health Trust, Italy, December 2020-April 2021. Acta Bio-Medica: Atenei Parmensis, 92(4). https://doi.org/10.23750/abm.v92i4.11896

Gibbs, A., Maripuu, M., Öhlund, L., Widerström, M., Nilsson, N., & Werneke, U. (2024). COVID-19-associated mortality in individuals with serious mental disorders in Sweden during the first two years of the pandemic- a population-based register study. BMC Psychiatry, 24(1), 189. https://doi.org/10.1186/s12888-024-05629-y

Gibertoni, D., Adja, K. Y. C., Golinelli, D., Reno, C., Regazzi, L., Lenzi, J., Sanmarchi, F., & Fantini, M. P. (2021). Patterns of COVID-19 related excess mortality in the municipalities of Northern Italy during the first wave of the pandemic. Health & Place, 67, 102508. https://doi.org/10.1016/j.healthplace.2021.102508

Giesen, C., Diez-Izquierdo, L., Saa-Requejo, C. M., Lopez-Carrillo, I., Lopez-Vilela, C. A., Seco-Martinez, A., Prieto, M. T. R., Malmierca, E., Garcia-Fernandez, C., & COVID Epidemiological Surveillance and Control Study Group. (2021). Epidemiological characteristics of the COVID-19 outbreak in a secondary hospital in Spain. American Journal of Infection Control, 49(2). https://doi.org/10.1016/j.ajic.2020.07.014

Giesen, C., Saa-Requejo, C., Lopez-Carrillo, I., Ortega-Torres, A., García-Fernández, C., & COVID-19 Vaccination Team. (2022). Risks of adverse outcomes in COVID-19 patients and vaccination status in a secondary hospital in Spain. Vacunas, 23. https://doi.org/10.1016/j.vacun.2022.05.004

Gil, A., Gil, R., Oyagüez, I., Carrasco, P., & Gonz Lez, A. (2006). Hospitalization by pneumonia and influenza in the 50-64 year old population in Spain (1999-2002). Human Vaccines, 2(4). https://doi.org/10.4161/hv.2.4.3104

Gil-de-Miguel, Á., Martinón-Torres, F., Díez-Domingo, J., de Lejarazu Leonardo, R. O., Pumarola, T., Carmo, M., Drago, G., López-Belmonte, J. L., Bricout, H., de Courville, C., & Margüello, E. R. (2022). Clinical and economic burden of physician-diagnosed influenza in adults during the 2017/2018 epidemic season in Spain. BMC Public Health, 22(1). https://doi.org/10.1186/s12889-022-14732-2

Gili, T., Benelli, G., Buscarini, E., Canetta, C., La Piana, G., Merli, G., Scartabellati, A., Viganò, G., Sfogliarini, R., Melilli, G., Assandri, R., Cazzato, D., Rossi, D. S., Usai, S., Caldarelli, G., Tramacere, I., Pellegata, G., &

Lauria, G. (2021). SARS-COV-2 comorbidity network and outcome in hospitalized patients in Crema, Italy. PloS One, 16(3). https://doi.org/10.1371/journal.pone.0248498

Gil-Jardiné, C., Chenais, G., Pradeau, C., Tentillier, E., Revel, P., Combes, X., Galinski, M., Tellier, E., & Lagarde, E. (2022). Surveillance of COVID-19 using a keyword search for symptoms in reports from emergency medical communication centers in Gironde, France : A 15 year retrospective cross-sectional study. Internal and Emergency Medicine, 17(2). https://doi.org/10.1007/s11739-021-02818-5

Gil-Prieto, R., Pérez, J. J., Drago, G., Kieffer, A., Roïz, J., Kazmierska, P., Sardesai, A., de Boisvilliers, S., López-Belmonte, J. L., Beuvelet, M., & Aldean, J. A. (2024). Modelling the potential clinical and economic impact of universal immunisation with nirsevimab versus standard of practice for protecting all neonates and infants in their first respiratory syncytial virus season in Spain. BMC Infectious Diseases, 24(1). https://doi.org/10.1186/s12879-024-09642-0

Gimeno-Miguel, A., Bliek-Bueno, K., Poblador-Plou, B., Carmona-Pírez, J., Poncel-Falcó, A., González-Rubio, F., Ioakeim-Skoufa, I., Pico-Soler, V., Aza-Pascual-Salcedo, M., Prados-Torres, A., Gimeno-Feliu, L. A., & PRECOVID Group. (2021). Chronic diseases associated with increased likelihood of hospitalization and mortality in 68,913 COVID-19 confirmed cases in Spain : A population-based cohort study. PloS One, 16(11). https://doi.org/10.1371/journal.pone.0259822

Gimferrer, L., Vila, J., Piñana, M., Andrés, C., Rodrigo-Pendás, J. A., Peremiquel-Trillas, P., Codina, M. G., C Martín, M. D., Esperalba, J., Fuentes, F., Rubio, S., Campins-Martí, M., Pumarola, T., & Antón, A. (2019). Virological surveillance of human respiratory syncytial virus A and B at a tertiary hospital in Catalonia (Spain) during five consecutive seasons (2013-2018). Future Microbiology, 14. https://doi.org/10.2217/fmb-2018-0261

Gioia, E., Colocci, A., Casareale, C., Marchetti, N., & Marincioni, F. (2022). The role of the socio-economic context in the spread of the first wave of COVID-19 in the Marche Region (central Italy). International Journal of Disaster Risk Reduction: IJDRR, 82. https://doi.org/10.1016/j.ijdrr.2022.103324

Giordani, B., Burgio, A., Grippo, F., Barone, A., Eugeni, E., & Baglio, G. (2024). The Use of ICD-9-CM Coding to Identify COVID-19 Diagnoses and Determine Risk Factors for 30-Day Death Rate in Hospitalized Patients in Italy : Retrospective Study. JMIR Public Health and Surveillance, 10. https://doi.org/10.2196/44062

Giorgi Rossi, P., Marino, M., Formisano, D., Venturelli, F., Vicentini, M., Grilli, R., & Reggio Emilia COVID-19 Working Group. (2020). Characteristics and outcomes of a cohort of COVID-19 patients in the Province of Reggio Emilia, Italy. PloS One, 15(8). https://doi.org/10.1371/journal.pone.0238281

Giovanetti, M., Cella, E., Benedetti, F., Magalis, B., Fonseca, V., Fabris, S., Campisi, G., Ciccozzi, A., Angeletti, S., Borsetti, A., Tambone, V., Sagnelli, C., Pascarella, S., Riva, A., Ceccarelli, G., Marcello, A., Azarian, T., Wilkinson, E., de Oliveira, T., … Ciccozzi, M. (2021). SARS-CoV-2 shifting transmission dynamics and hidden reservoirs potentially limit efficacy of public health interventions in Italy. COMMUNICATIONS BIOLOGY, 4(1). https://doi.org/10.1038/s42003-021-02025-0

Gisondi, P., Piaserico, S., Naldi, L., Dapavo, P., Conti, A., Malagoli, P., Marzano, A. V., Bardazzi, F., Gasperini, M., Cazzaniga, S., Costanzo, A., & collaborators in the studies of COVID-19 pandemic. (2021). Incidence rates of hospitalization and death from COVID-19 in patients with psoriasis receiving biological treatment : A Northern Italy experience. The Journal of Allergy and Clinical Immunology, 147(2). https://doi.org/10.1016/j.jaci.2020.10.032

Giuliani, D., Dickson, M. M., Espa, G., & Santi, F. (2020). Modelling and predicting the spatio-temporal spread of cOVID-19 in Italy. BMC Infectious Diseases, 20(1). https://doi.org/10.1186/s12879-020-05415-7

Gobbato, M., Clagnan, E., Burba, I., Rizzi, L., Grassetti, L., Del Zotto, S., Dal Maso, L., Serraino, D., & Tonutti, G. (2020). Clinical, demographical characteristics and hospitalisation of 3,010 patients with Covid-19 in Friuli Venezia Giulia Region (Northern Italy). A multivariate, population-based, statistical analysis. Epidemiologia E Prevenzione, 44(5-6 Suppl 2). https://doi.org/10.19191/EP20.5-6.S2.122

Gobbato, M., Clagnan, E., Toffolutti, F., Del Zotto, S., Burba, I., Tosolini, F., Polimeni, J., Serraino, D., & Taborelli, M. (2023). Vaccination against SARS-CoV-2 and risk of hospital admission and death among infected

cancer patients : A population-based study in northern Italy. Cancer Epidemiology, 82. https://doi.org/10.1016/j.canep.2022.102318

Godaert, L., Cebille, A., Proye, E., & Dramé, M. (2021). COVID-19 versus Seasonal Influenza : Comparison of Profiles of Older Adults Hospitalized in a Short-Term Geriatric Ward in France. The American Journal of Tropical Medicine and Hygiene, 106(1). https://doi.org/10.4269/ajtmh.21-0480

Godoy, P., Romero, A., Soldevila, N., Torner, N., Jané, M., Martínez, A., Caylà, J. A., Rius, C., Domínguez, A., & Working Group on Surveillance of Severe Influenza Hospitalized Cases in Catalonia. (2018). Influenza vaccine effectiveness in reducing severe outcomes over six influenza seasons, a case-case analysis, Spain, 2010/11 to 2015/16. Euro Surveillance: Bulletin Europeen Sur Les Maladies Transmissibles = European Communicable Disease Bulletin, 23(43). https://doi.org/10.2807/1560-7917.ES.2018.23.43.1700732

Goettler, D., Niekler, P., Liese, J. G., & Streng, A. (2022). Epidemiology and direct healthcare costs of Influenza-associated hospitalizations—Nationwide inpatient data (Germany 2010-2019). BMC Public Health, 22(1). https://doi.org/10.1186/s12889-022-12505-5

Goldstein, E. (2022). Non-COVID-19 mortality in France, April 2020—June 2022: Reduction compared to pre-pandemic mortality patterns, relative increase during the Omicron period, and the importance of detecting SARS-CoV-2 infections. medRxiv. https://doi.org/10.1101/2022.11.28.22282832

Goldstein, E. (2022a). Mortality associated with different influenza subtypes in France between 2015-2019. medRxiv, (Goldstein E., edmigo3@gmail.com) Massachusetts Eye and Ear Hospital, Harvard Medical School, Boston, MA, United States. https://doi.org/10.1101/2022.11.21.22282612

Goldstein, E. (2022b). SARS-CoV-2-associated mortality with a principal cause other than COVID-19 during the Omicron epidemic in France and detection and treatment of Omicron infections in associated complications. medRxiv, (Goldstein E., edmigo3@gmail.com) Massachusetts Eye and Ear, Harvard Medical School, Boston, MA, United States. https://doi.org/10.1101/2022.12.15.22283529

Goldstein, E., & Lipsitch, M. (2020). Temporal rise in the proportion of both younger adults and older adolescents among COVID-19 cases in Germany : Evidence of lesser adherence to social distancing practices? medRxiv: The Preprint Server for Health Sciences. https://doi.org/10.1101/2020.04.08.20058719

Gomes da Silva, P., Gonçalves, J., Torres Franco, A., Rodriguez, E., Diaz, I., Orduña Domingo, A., Garcinuño Pérez, S., March Roselló, G. A., Dueñas Gutiérrez, C. J., São José Nascimento, M., Sousa, S. I. V., Garcia Encina, P., & Mesquita, J. R. (2023). Environmental Dissemination of SARS-CoV-2 in a University Hospital during the COVID-19 5th Wave Delta Variant Peak in Castile-León, Spain. International Journal of Environmental Research and Public Health, 20(2). https://doi.org/10.3390/ijerph20021574

Gomes, D., Beyerlein, A., Katz, K., Hoelscher, G., Nennstiel, U., Liebl, B., Überla, K., & von Kries, R. (2021). Is the BNT162b2 COVID-19 vaccine effective in elderly populations ? Results from population data from Bavaria, Germany. PloS One, 16(11). https://doi.org/10.1371/journal.pone.0259370

Gómez-Antúnez, M., Muiño-Míguez, A., Bacete-Cebrián, M., Rubio-Rivas, M., Lebrón Ramos, J. M., de Cossío Tejido, S., Peris-García, J., López-Caleya, J. F., Casas-Rojo, J. M., Millán Núñez-Cortés, J., & en nombre del Grupo SEMI-COVID-19 Network. (2023). [Patients with COPD hospitalized due to COVID-19 in Spain : A comparison between the first and second wave]. Revista Clinica Espanola, 223(5). https://doi.org/10.1016/j.rce.2023.02.003

Gomez-Barroso, D., Martinez-Beneito, M. A., Flores, V., Amorós, R., Delgado, C., Botella, P., Zurriaga, O., Larrauri, A., Jiménez, S., Simón, F., León, I., Miralles, M., Maside, A. L., Perez, E., Romero, E. R., Cuenca, S. M., Vigil, M. D. P. A., Giménez, J., Gonzalez, L., … Pérez, D. C. (2014). Geographical spread of influenza incidence in Spain during the 2009 A(H1N1) pandemic wave and the two succeeding influenza seasons. Epidemiology and Infection, 142(12), 2629–2641. https://doi.org/10.1017/S0950268813003439

Gómez-García, R. M., De-Miguel-Díez, J., López-de-Andrés, A., Hernández-Barrera, V., Jimenez-Sierra, A., Cuadrado-Corrales, N., Zamorano-León, J. J., Carabantes-Alarcón, D., Bodas-Pinedo, A., & Jiménez-García, R. (2025). Prevalence of Respiratory Syncytial Virus Infection in Hospitalized COPD Patients in Spain Between 2018-2022. Diseases (Basel, Switzerland), 13(1). https://doi.org/10.3390/diseases13010023

Gomez-Garcia, R. M., Jiménez-Garcia, R., López-de-Andrés, A., Hernández-Barrera, V., Carabantes-Alarcon, D., Zamorano-León, J. J., Cuadrado-Corrales, N., Jiménez-Sierra, A., & De-Miguel-Diez, J. (2024). Burden of Respiratory Syncytial Virus Infection in Children and Older Patients Hospitalized with Asthma : A Seven-Year Longitudinal Population-Based Study in Spain. Viruses, 16(11). https://doi.org/10.3390/v16111749

Gómez-García, R. M., Jiménez-García, R., López-de-Andrés, A., Hernández-Barrera, V., Jimenez-Sierra, A., Cuadrado-Corrales, N., Zamorano-León, J. J., Carabantes-Alarcón, D., Bodas-Pinedo, A., & De-Miguel-Díez, J. (2025). The Role of Respiratory Syncytial Virus Infection in the Hospitalization and Mortality of Adults with Congestive Heart Failure in Spain, 2018-2022. Viruses, 17(4), 516. https://doi.org/10.3390/v17040516

González, O., & Santos-Requejo, L. (2024). The Impact of Knowledge and Trust on COVID-19 Vaccination Intention : Analysis of a Population Group with Low Incentives to Vaccinate in Spain. SAGE OPEN, 14(3). https://doi.org/10.1177/21582440241267044

González-Beltrán, D., Donat, M., Politi, J., Ronda, E., Barrio, G., Belza, M. J., & Regidor, E. (2024). Changes in all-cause and cause-specific mortality by occupational skill during COVID-19 epidemic in Spain. Journal of Epidemiology and Community Health, 78(11). https://doi.org/10.1136/jech-2024-222065

González-Candelas, F., Astray, J., Alonso, J., Castro, A., Cantón, R., Galán, J. C., Garin, O., Sáez, M., Soldevila, N., Baricot, M., Castilla, J., Godoy, P., Delgado-Rodríguez, M., Martín, V., Mayoral, J. M., Pumarola, T., Quintana, J. M., Tamames, S., Domínguez, A., & CIBERESP Cases and Controls in Pandemic Influenza Working Group. (2012). Sociodemographic factors and clinical conditions associated to hospitalization in influenza A (H1N1) 2009 virus infected patients in Spain, 2009-2010. PloS One, 7(3). https://doi.org/10.1371/journal.pone.0033139

Goodall, J. W., Reed, T. a. N., Ardissino, M., Bassett, P., Whittington, A. M., Cohen, D. L., & Vaid, N. (2020). Risk factors for severe disease in patients admitted with COVID-19 to a hospital in London, England : A retrospective cohort study. Epidemiology and Infection, 148. https://doi.org/10.1017/S0950268820002472

Gorgels, K. M. F., Dukers-Muijrers, N. H. T. M., Evers, Y. J., Hackert, V. H., Savelkoul, P. H. M., & Hoebe, C. J. P. A. (2024). Impact of a large-scale event on SARS-CoV-2 cases and hospitalizations in the Netherlands, carnival seasons 2022 and 2023. Public Health in Practice (Oxford, England), 8. https://doi.org/10.1016/j.puhip.2024.100523

Gorgels, K. M. F., Mujakovic, S., Stallenberg, E., Hackert, V. H., & Hoebe, C. J. P. A. (2024). Implementation and effectiveness of non-pharmaceutical interventions, including mask mandates and ventilation, on SARS-CoV-2 transmission (alpha variant) in primary schools in the Netherlands. PloS One, 19(6). https://doi.org/10.1371/journal.pone.0305195

Gosselin, A., Warszawski, J., Bajos, N., & EpiCov Study Grp. (2022). Higher risk, higher protection : COVID-19 risk among immigrants in France-results from the population-based EpiCov survey. EUROPEAN JOURNAL OF PUBLIC HEALTH, 32(4). https://doi.org/10.1093/eurpub/ckac046

Götz, T., & Heidrich, P. (2020). Early stage COVID-19 disease dynamics in Germany : Models and parameter identification. JOURNAL OF MATHEMATICS IN INDUSTRY, 10(1). https://doi.org/10.1186/s13362-020-00088-y

Græsbøll, K., Eriksen, R. S., Kirkeby, C., & Christiansen, L. E. (2024). Digital twin simulation modelling shows that mass testing and local lockdowns effectively controlled COVID-19 in Denmark. Communications Medicine, 4(1). https://doi.org/10.1038/s43856-024-00621-9

Gram, M. A., Moustsen-Helms, I. R., Valentiner-Branth, P., & Emborg, H.-D. (2023). Sociodemographic differences in Covid-19 vaccine uptake in Denmark : A nationwide register-based cohort study. BMC Public Health, 23(1). https://doi.org/10.1186/s12889-023-15301-x

Gram, M. A., Nielsen, J., Schelde, A. B., Nielsen, K. F., Moustsen-Helms, I. R., Sørensen, A. K. B., Valentiner-Branth, P., & Emborg, H.-D. (2021). Vaccine effectiveness against SARS-CoV-2 infection, hospitalization, and death when combining a first dose ChAdOx1 vaccine with a subsequent mRNA vaccine in Denmark : A nationwide population-based cohort study. PLoS Medicine, 18(12). https://doi.org/10.1371/journal.pmed.1003874

Grant, R., Charmet, T., Schaeffer, L., Galmiche, S., Madec, Y., Von Platen, C., Chény, O., Omar, F., David, C., Rogoff, A., Paireau, J., Cauchemez, S., Carrat, F., Septfons, A., Levy-Bruhl, D., Mailles, A., & Fontanet, A. (2022). Impact of SARS-CoV-2 Delta variant on incubation, transmission settings and vaccine effectiveness : Results from a nationwide case-control study in France. The Lancet Regional Health. Europe, 13. https://doi.org/10.1016/j.lanepe.2021.100278

Grasselli, G., Greco, M., Zanella, A., Albano, G., Antonelli, M., Bellani, G., Bonanomi, E., Cabrini, L., Carlesso, E., Castelli, G., Cattaneo, S., Cereda, D., Colombo, S., Coluccello, A., Crescini, G., Forastieri Molinari, A., Foti, G., Fumagalli, R., Iotti, G. A., … COVID-19 Lombardy ICU Network. (2020). Risk Factors Associated With Mortality Among Patients With COVID-19 in Intensive Care Units in Lombardy, Italy. JAMA Internal Medicine, 180(10). https://doi.org/10.1001/jamainternmed.2020.3539

Grasselli, G., Zangrillo, A., Zanella, A., Antonelli, M., Cabrini, L., Castelli, A., Cereda, D., Coluccello, A., Foti, G., Fumagalli, R., Iotti, G., Latronico, N., Lorini, L., Merler, S., Natalini, G., Piatti, A., Ranieri, M. V., Scandroglio, A. M., Storti, E., … COVID-19 Lombardy ICU Network. (2020). Baseline Characteristics and Outcomes of 1591 Patients Infected With SARS-CoV-2 Admitted to ICUs of the Lombardy Region, Italy. JAMA, 323(16). https://doi.org/10.1001/jama.2020.5394

Grassi, A., Andriolo, L., Golinelli, D., Tedesco, D., Rosa, S., Gramegna, P., Ciaffi, J., Meliconi, R., Landini, M. P., Filardo, G., Fantini, M. P., & Zaffagnini, S. (2021). Higher 90-Day Mortality after Surgery for Hip Fractures in Patients with COVID-19 : A Case-Control Study from a Single Center in Italy. International Journal of Environmental Research and Public Health, 18(10). https://doi.org/10.3390/ijerph18105205

Grave, M., Viguerie, A., Barros, G. F., Reali, A., & Coutinho, A. L. G. A. (2021). Assessing the Spatio-temporal Spread of COVID-19 via Compartmental Models with Diffusion in Italy, USA, and Brazil. Archives of Computational Methods in Engineering: State of the Art Reviews, 28(6). https://doi.org/10.1007/s11831-021-09627-1

Gray, P., Eriksson, T., Skoglund, L., Lagheden, C., Hellström, C., Pin, E., Suomenrinne-Nordvik, A., Pimenoff, V. N., Nilsson, P., Dillner, J., & Lehtinen, M. (2024). Seroepidemiological assessment of the spread of SARS-CoV-2 among 25 and 28 year-old adult women in Finland between March 2020-June 2022. PloS One, 19(7). https://doi.org/10.1371/journal.pone.0305285

Gray, W. K., Navaratnam, A. V., Day, J., Babu, P., Mackinnon, S., Adelaja, I., Bartlett-Pestell, S., Moulton, C., Mann, C., Batchelor, A., Swart, M., Snowden, C., Dyer, P., Jones, M., Allen, M., Hopper, A., Rayman, G., Kar, P., Wheeler, A., … Briggs, T. W. R. (2021). Variability in COVID-19 in-hospital mortality rates between national health service trusts and regions in England : A national observational study for the Getting It Right First Time Programme. EClinicalMedicine, 35. https://doi.org/10.1016/j.eclinm.2021.100859

Gray, W. K., Navaratnam, A. V., Day, J., Wendon, J., & Briggs, T. W. R. (2021). Changes in COVID-19 in-hospital mortality in hospitalised adults in England over the first seven months of the pandemic: An observational study using administrative data. The Lancet Regional Health. Europe, 5, 100104. https://doi.org/10.1016/j.lanepe.2021.100104

Gray, W. K., Navaratnam, A. V., Day, J., Wendon, J., & Briggs, T. W. R. (2022). COVID-19 hospital activity and in-hospital mortality during the first and second waves of the pandemic in England: An observational study. Thorax, 77(11), 1113–1120. https://doi.org/10.1136/thoraxjnl-2021-218025

Green, H. K., Andrews, N., Fleming, D., Zambon, M., & Pebody, R. (2013). Mortality attributable to influenza in England and Wales prior to, during and after the 2009 pandemic. PloS One, 8(12), e79360. https://doi.org/10.1371/journal.pone.0079360

Green, H. K., Andrews, N., Letley, L., Sunderland, A., White, J., & Pebody, R. (2015). Phased introduction of a universal childhood influenza vaccination programme in England : Population-level factors predicting variation in national uptake during the first year, 2013/14. Vaccine, 33(22). https://doi.org/10.1016/j.vaccine.2015.03.049

Green, H. K., Brousseau, N., Andrews, N., Selby, L., & Pebody, R. (2016). Illness absenteeism rates in primary and secondary schools in 2013-2014 in England : Was there any impact of vaccinating children of primary-school age against influenza? Epidemiology and Infection, 144(16). https://doi.org/10.1017/S0950268816001680

Green, H. K., Ellis, J., Galiano, M., Watson, J. M., & Pebody, R. G. (2013). Critical care surveillance: Insights into the impact of the 2010/11 influenza season relative to the 2009/10 pandemic season in England. Euro Surveillance: Bulletin Europeen Sur Les Maladies Transmissibles = European Communicable Disease Bulletin, 18(23), 20499. https://doi.org/10.2807/ese.18.23.20499-en

Green, H. K., Zhao, H., Boddington, N. L., Andrews, N., Durnall, H., Elliot, A. J., Smith, G., Gorton, R., Donati, M., Ellis, J., Zambon, M., & Pebody, R. (2015). Detection of varying influenza circulation within England in 2012/13 : Informing antiviral prescription and public health response. Journal of Public Health (Oxford, England), 37(2). https://doi.org/10.1093/pubmed/fdu046

Green, M. A., & Semple, M. G. (2023). Occupational inequalities in the prevalence of COVID-19 : A longitudinal observational study of England, August 2020 to January 2021. PloS One, 18(4). https://doi.org/10.1371/journal.pone.0283119

Gregori, D., Azzolina, D., Lanera, C., Prosepe, I., Destro, N., Lorenzoni, G., & Berchialla, P. (2020). A first estimation of the impact of public health actions against COVID-19 in Veneto (Italy). Journal of Epidemiology and Community Health, 74(10). https://doi.org/10.1136/jech-2020-214209

Greiner, T., Aigner, A., & Tinnemann, P. (2022). SARS-CoV-2 infection dynamics in the tourism season 2020 in North Frisia, Germany. FRONTIERS IN EPIDEMIOLOGY, 2. https://doi.org/10.3389/fepid.2022.1029807

Griffith, G. J., Davey Smith, G., Manley, D., Howe, L. D., & Owen, G. (2021). Interrogating structural inequalities in COVID-19 mortality in England and Wales. Journal of Epidemiology and Community Health, 75(12). https://doi.org/10.1136/jech-2021-216666

Griffith, G. J., Owen, G., Manley, D., Howe, L. D., & Davey Smith, G. (2022). Continuing inequalities in COVID-19 mortality in England and Wales, and the changing importance of regional, over local, deprivation. Health & Place, 76. https://doi.org/10.1016/j.healthplace.2022.102848

Groenheit, R., Bacchus, P., Galanis, I., Sondén, K., Bujila, I., Efimova, T., Garli, F., Lindsjö, O. K., Mansjö, M., Movert, E., Pettke, A., Rapp, M., Sperk, M., Söderholm, S., Asin, K. V., Zanetti, S., Karlberg, M. L., Bråve, A., Blom, K., & Klingström, J. (2023). High Prevalence of SARS-CoV-2 Omicron Infection Despite High Seroprevalence, Sweden, 2022. Emerging Infectious Diseases, 29(6). https://doi.org/10.3201/eid2906.221862

Groenheit, R., Beser, J., Kühlmann Berenzon, S., Galanis, I., van Straten, E., Duracz, J., Rapp, M., Hansson, D., Mansjö, M., Söderholm, S., Muradrasoli, S., Risberg, A., Ölund, R., Wiklund, A., Metzkes, K., Lundberg, M., Bacchus, P., Tegmark Wisell, K., & Bråve, A. (2022). Point prevalence of SARS-CoV-2 infection in Sweden at six time points during 2020. BMC Infectious Diseases, 22(1). https://doi.org/10.1186/s12879-022-07858-6

Grønkjær, C. S., Christensen, R. H. B., Kondziella, D., & Benros, M. E. (2023). Long-term neurological outcome after COVID-19 using all SARS-CoV-2 test results and hospitalisations in Denmark with 22-month follow-up. Nature Communications, 14(1). https://doi.org/10.1038/s41467-023-39973-6

Grune, J., Savelsberg, D., Kobus, M., Lindner, A. K., Herrmann, W. J., & Schuster, A. (2023). Determinants of COVID-19 vaccine acceptance and access among people experiencing homelessness in Germany : A qualitative interview study. Frontiers in Public Health, 11. https://doi.org/10.3389/fpubh.2023.1148029

Gu, X., Agrawal, U., Midgley, W., Bedston, S., Anand, S. N., Goudie, R., Byford, R., Joy, M., Jamie, G., Hoang, U., Ordóñez-Mena, J. M., Robertson, C., Hobbs, F. D. R., Akbari, A., Sheikh, A., & de Lusignan, S. (2024). COVID-19 and influenza vaccine uptake among pregnant women in national cohorts of England and Wales. NPJ Vaccines, 9(1). https://doi.org/10.1038/s41541-024-00934-9

Guarnieri, V., Moriondo, M., Giovannini, M., Lodi, L., Ricci, S., Pisano, L., Barbacci, P., Bini, C., Indolfi, G., Zanobini, A., & Azzari, C. (2021). Surveillance on Healthcare Workers During the First Wave of SARS-CoV-2 Pandemic in Italy: The Experience of a Tertiary Care Pediatric Hospital. Frontiers in Public Health, 9, 644702. https://doi.org/10.3389/fpubh.2021.644702

Gubbels, S., Perner, A., Valentiner-Branth, P., & Molbak, K. (2010). National surveillance of pandemic influenza A(H1N1) infection-related admissions to intensive care units during the 2009-10 winter peak in Denmark: Two complementary approaches. Euro Surveillance: Bulletin Europeen Sur Les Maladies

Transmissibles = European Communicable Disease Bulletin, 15(49), 19743. https://doi.org/10.2807/ese.15.49.19743-en

Gudde, A., Krogsgaard, L. W., Benedetti, G., Schierbech, S. K., Brokhattingen, N., Petrovic, K., Rasmussen, L. D., Franck, K. T., Ethelberg, S., Larsen, N. B., & Christiansen, L. E. (2025). Predicting hospital admissions due to COVID-19 in Denmark using wastewater-based surveillance. The Science of the Total Environment, 966. https://doi.org/10.1016/j.scitotenv.2025.178674

Guemas, E., Cassaing, S., Malavaud, S., Fillaux, J., Chauvin, P., Lelièvre, L., Ruiz, S., Riu, B., Berry, A., & Iriart, X. (2022). A Clustered Case Series of Mucorales Detection in Respiratory Samples from COVID-19 Patients in Intensive Care, France, August to September 2021. Journal of Fungi (Basel, Switzerland), 8(3). https://doi.org/10.3390/jof8030258

Guerra, I., Algaba, A., Jiménez, L., Mar Aller, M., Garza, D., Bonillo, D., Molina Esteban, L. M., & Bermejo, F. (2021). Incidence, Clinical Characteristics, and Evolution of SARS-CoV-2 Infection in Patients With Inflammatory Bowel Disease : A Single-Center Study in Madrid, Spain. Inflammatory Bowel Diseases, 27(1). https://doi.org/10.1093/ibd/izaa221

Guerra, O., Salcines, V., & Prieto, D. (2024). Geodemographic profiles of COVID-19 mortality inside/outside nursing homes. Spatial analysis from microdata in North Spain. APPLIED GEOGRAPHY, 162. https://doi.org/10.1016/j.apgeog.2023.103153

Guerriero, M., Bisoffi, Z., Poli, A., Micheletto, C., & Pomari, C. (2020). Prevalence of asymptomatic SARS-CoV-2-positive individuals in the general population of northern Italy and evaluation of a diagnostic serological ELISA test : A cross-sectional study protocol. BMJ Open, 10(10). https://doi.org/10.1136/bmjopen-2020-040036

Guerriero, M., Bisoffi, Z., Poli, A., Micheletto, C., Conti, A., & Pomari, C. (2021). Prevalence of SARS-CoV-2, Verona, Italy, April-May 2020. Emerging Infectious Diseases, 27(1). https://doi.org/10.3201/eid2701.202740

Guerrisi, C., Turbelin, C., Souty, C., Poletto, C., Blanchon, T., Hanslik, T., Bonmarin, I., Levy-Bruhl, D., & Colizza, V. (2018). The potential value of crowdsourced surveillance systems in supplementing sentinel influenza networks : The case of France. Euro Surveillance: Bulletin Europeen Sur Les Maladies Transmissibles = European Communicable Disease Bulletin, 23(25). https://doi.org/10.2807/1560-7917.ES.2018.23.25.1700337

Guibert, N., Trepat, K., Pozzetto, B., Josset, L., Fassier, J.-B., Allatif, O., Saker, K., Brengel-Pesce, K., Walzer, T., Vanhems, P., Trouillet-Assant, S., & Lyon-COVID study group. (2023). A third vaccine dose equalises the levels of effectiveness and immunogenicity of heterologous or homologous COVID-19 vaccine regimens, Lyon, France, December 2021 to March 2022. Euro Surveillance: Bulletin Europeen Sur Les Maladies Transmissibles = European Communicable Disease Bulletin, 28(15). https://doi.org/10.2807/1560-7917.ES.2023.28.15.2200746

Guirao, A. (2020). The Covid-19 outbreak in Spain. A simple dynamics model, some lessons, and a theoretical framework for control response. INFECTIOUS DISEASE MODELLING, 5. https://doi.org/10.1016/j.idm.2020.08.010

Guisado-Clavero, M., Herrero Gil, A., Pérez Álvarez, M., Castelo Jurado, M., Herrera Marinas, A., Aguilar Ruiz, V., Gefaell Iarrondo, I., Menéndez Orenga, M., & Ares-Blanco, S. (2021). Clinical characteristics of SARS-CoV-2 pneumonia diagnosed in a primary care practice in Madrid (Spain). BMC Family Practice, 22(1). https://doi.org/10.1186/s12875-021-01430-y

Gujski, M., Jankowski, M., Pinkas, J., Wierzba, W., Samel-Kowalik, P., Zaczyński, A., Jędrusik, P., Pańkowski, I., Juszczyk, G., Rakocy, K., & Raciborski, F. (2020). Prevalence of Current and Past SARS-CoV-2 Infections among Police Employees in Poland, June-July 2020. Journal of Clinical Medicine, 9(10). https://doi.org/10.3390/jcm9103245

Gujski, M., Jankowski, M., Rabczenko, D., Goryński, P., & Juszczyk, G. (2021). Characteristics and Clinical Outcomes of 116,539 Patients Hospitalized with COVID-19-Poland, March-December 2020. Viruses, 13(8). https://doi.org/10.3390/v13081458

Gul, S., Tuncay, K., Binici, B., & Aydin, B. (2020). Transmission dynamics of Covid-19 in Italy, Germany and Turkey considering social distancing, testing and quarantine. JOURNAL OF INFECTION IN DEVELOPING COUNTRIES, 14(7). https://doi.org/10.3855/jidc.12844

Gullón, P., Cuesta-Lozano, D., Cuevas-Castillo, C., Fontán-Vela, M., & Franco, M. (2022). Temporal trends in within-city inequities in COVID-19 incidence rate by area-level deprivation in Madrid, Spain. Health & Place, 76. https://doi.org/10.1016/j.healthplace.2022.102830

Gunawardene, M. A., Gessler, N., Wohlmuth, P., Heitmann, K., Anders, P., Jaquet, K., Herborn, C. U., Arnold, D., Bein, B., Bergmann, M. W., Herrlinger, K. R., Stang, A., Schreiber, R., Wesseler, C., & Willems, S. (2021). Prognostic Impact of Acute Cardiovascular Events in COVID-19 Hospitalized Patients-Results from the CORONA Germany Study. Journal of Clinical Medicine, 10(17). https://doi.org/10.3390/jcm10173982

Günster, C., Busse, R., Spoden, M., Rombey, T., Schillinger, G., Hoffmann, W., Weber-Carstens, S., Schuppert, A., & Karagiannidis, C. (2021). 6-month mortality and readmissions of hospitalized COVID-19 patients : A nationwide cohort study of 8,679 patients in Germany. PloS One, 16(8). https://doi.org/10.1371/journal.pone.0255427

Gustafsson, P. E., San Sebastian, M., Fonseca-Rodriguez, O., & Fors Connolly, A.-M. (2022). Inequitable impact of infection: Social gradients in severe COVID-19 outcomes among all confirmed SARS-CoV-2 cases during the first pandemic wave in Sweden. Journal of Epidemiology and Community Health, 76(3), 261–267. https://doi.org/10.1136/jech-2021-216778

Guthmann, J.-P., Fonteneau, L., Bonmarin, I., & Lévy-Bruhl, D. (2012). Influenza vaccination coverage one year after the A(H1N1) influenza pandemic, France, 2010-2011. Vaccine, 30(6), 995–997. https://doi.org/10.1016/j.vaccine.2011.12.011

Gutiérrez, M., Inguanzo, B., & Orbe, S. (2021). Distributional impact of COVID-19 : Regional inequalities in cases and deaths in Spain during the first wave. APPLIED ECONOMICS, 53(31). https://doi.org/10.1080/00036846.2021.1884838

Guzek, A., Rybicki, Z., Woźniak-Kosek, A., & Tomaszewski, D. (2022). The Clinical Manifestation of SARS-CoV-2 in Critically Ill Patients with Klebsiella pneumoniae NDM Hospitalized in the ICU of a Modular Hospital during the Third Wave of the Pandemic in Poland-An Observational Cohort Study. Diagnostics (Basel, Switzerland), 12(5), 1118. https://doi.org/10.3390/diagnostics12051118

Guzmán-Rincón, L. M., Hill, E. M., Dyson, L., Tildesley, M. J., & Keeling, M. J. (2022). Bayesian Estimation of real-time Epidemic Growth Rates using Gaussian Processes : Local dynamics of SARS-CoV-2 in England. medRxiv, (Guzmán-Rincón L.M., laura.guzman-rincon@warwick.ac.uk; Hill E.M.; Dyson L.; Tildesley M.J.; Keeling M.J.) The Zeeman Institute for Systems Biology&Infectious Disease Epidemiology Research, School of Life Sciences and Mathematics Institute, University of Warwick, Coventry, United Kingdom. https://doi.org/10.1101/2022.01.01.21268131

Guzzetta, G., Riccardo, F., Marziano, V., Poletti, P., Trentini, F., Bella, A., Andrianou, X., Del Manso, M., Fabiani, M., Bellino, S., Boros, S., Urdiales, A. M., Vescio, M. F., Piccioli, A., COVID-19 Working Group, 2, Brusaferro, S., Rezza, G., Pezzotti, P., Ajelli, M., & Merler, S. (2021). Impact of a Nationwide Lockdown on SARS-CoV-2 Transmissibility, Italy. Emerging Infectious Diseases, 27(1). https://doi.org/10.3201/eid2701.202114

Haanappel, C. P., Oude Munnink, B. B., Sikkema, R. S., Voor In 't Holt, A. F., de Jager, H., de Boever, R., Koene, H. H. H. T., Boter, M., Chestakova, I. V., van der Linden, A., Molenkamp, R., Osbak, K. K., Arcilla, M. S., Vos, M. C., Koopmans, M. P. G., & Severin, J. A. (2023). Combining epidemiological data and whole genome sequencing to understand SARS-CoV-2 transmission dynamics in a large tertiary care hospital during the first COVID-19 wave in The Netherlands focusing on healthcare workers. Antimicrobial Resistance and Infection Control, 12(1). https://doi.org/10.1186/s13756-023-01247-7

Haapanen, M., Renko, M., Artama, M., & Kuitunen, I. (2021). The impact of the lockdown and the re-opening of schools and day cares on the epidemiology of SARS-CoV-2 and other respiratory infections in children—A nationwide register study in Finland. EClinicalMedicine, 34. https://doi.org/10.1016/j.eclinm.2021.100807

Haars, J., Wallin, F., Elfving, K., Jonsson, A.-K., Ellström, P., Mölling, P., Lindh, J., Yin, H., Sundqvist, M., Kaden, R., Palanisamy, N., & Lennerstrand, J. (2025). Dynamics of SARS-CoV-2 variants and mutations in Central Sweden between 2023 and 2024 and their potential implications on monoclonal antibodies pemivibart and sipavibart as PrEP in the region. Infectious Diseases. https://doi.org/10.1080/23744235.2025.2509011

Haas, J., Braun, S., & Wutzler, P. (2016). Burden of influenza in Germany : A retrospective claims database analysis for the influenza season 2012/2013. The European Journal of Health Economics: HEPAC: Health Economics in Prevention and Care, 17(6). https://doi.org/10.1007/s10198-015-0708-7

Haase, N., Plovsing, R., Christensen, S., Poulsen, L. M., Brøchner, A. C., Rasmussen, B. S., Helleberg, M., Jensen, J. U. S., Andersen, L. P. K., Siegel, H., Ibsen, M., Jørgensen, V. L., Winding, R., Iversen, S., Pedersen, H. P., Madsen, J., Sølling, C., Garcia, R. S., Michelsen, J., … Perner, A. (2022). Changes over time in characteristics, resource use and outcomes among ICU patients with COVID-19-A nationwide, observational study in Denmark. Acta Anaesthesiologica Scandinavica, 66(8). https://doi.org/10.1111/aas.14113

Haase, N., Plovsing, R., Christensen, S., Poulsen, L. M., Brøchner, A. C., Rasmussen, B. S., Helleberg, M., Jensen, J. U. S., Andersen, L. P. K., Siegel, H., Ibsen, M., Jørgensen, V., Winding, R., Iversen, S., Pedersen, H. P., Madsen, J., Sølling, C., Garcia, R. S., Michelsen, J., … Perner, A. (2021). Characteristics, interventions, and longer term outcomes of COVID-19 ICU patients in Denmark-A nationwide, observational study. Acta Anaesthesiologica Scandinavica, 65(1). https://doi.org/10.1111/aas.13701

Hackenberg, A., Arman-Kalcek, G., Hiller, J., & Gabriel, G. (2013). Antibody prevalence to the 2009 pandemic influenza A (H1N1) virus in Germany: Geographically variable immunity in winter 2010/2011. Medical Microbiology and Immunology, 202(1), 87–94. https://doi.org/10.1007/s00430-012-0251-4

Häckl, D., Pignot, M., Dang, P. L., Lauenroth, V., Jah, F., & Wendtner, C.-M. (2024). [Clinical courses and costs for hospitalizations of potentially immunocompromised COVID-19 patients in Germany]. Deutsche Medizinische Wochenschrift (1946), 149(7). https://doi.org/10.1055/a-2239-0453

Hadigal, S., & Cook, J. (2022). Knowledge and perception regarding effectiveness in influenza vaccines among General Practitioners in Germany : A national survey. Vaccine: X, 12. https://doi.org/10.1016/j.jvacx.2022.100236

Haeberer, M., Bruyndonckx, R., Polkowska-Kramek, A., Torres, A., Liang, C., Nuttens, C., Casas, M., Lemme, F., Ewnetu, W. B., Tran, T. M. P., Atwell, J. E., Diez, C. M., Gessner, B. D., & Begier, E. (2024). Estimated Respiratory Syncytial Virus-Related Hospitalizations and Deaths Among Children and Adults in Spain, 2016-2019. Infectious Diseases and Therapy, 13(3). https://doi.org/10.1007/s40121-024-00920-7

Haeberer, M., López-Ibáñez de Aldecoa, A., Seabroke, S., Ramirez Agudelo, J. L., Mora, L., Sarabia, L., Meroc, E., Aponte-Torres, Z., Sato, R., & Law, A. W. (2025). Economic burden of children hospitalized with respiratory syncytial virus infection in Spain, 2016-2019. Vaccine, 43(Pt 2). https://doi.org/10.1016/j.vaccine.2024.126512

Haeberer, M., López-Ibáñez de Aldecoa, A., Seabroke, S., Ramirez Agudelo, J. L., Mora, L., Sarabia, L., Peerawaranun, P., Meroc, E., Aponte-Torres, Z., Law, A. W., & Sato, R. (2025). Hospitalization cost estimates of respiratory syncytial virus and influenza infections in adults in Spain, 2016-2019. Vaccine, 46. https://doi.org/10.1016/j.vaccine.2024.126683

Haeberer, M., Mengel, M., Fan, R., Toquero-Asensio, M., Martin-Toribio, A., Liu, Q., He, Y., Uppal, S., Rojo-Rello, S., Domínguez-Gil, M., Hernán-García, C., Fernández-Espinilla, V., Atwell, J. E., Sanz, J. C., Eiros, J. M., & Sanz-Muñoz, I. (2024). Respiratory Syncytial Virus Risk Profile in Hospitalized Infants and Comparison with Influenza and COVID-19 Controls in Valladolid, Spain, 2010-2022. Infectious Diseases and Therapy, 13(11). https://doi.org/10.1007/s40121-024-01058-2

Haeberer, M., Mengel, M., Fan, R., Toquero-Asensio, M., Martin-Toribio, A., Liu, Q., He, Y., Uppal, S., Rojo-Rello, S., Domínguez-Gil, M., Hernán-García, C., Fernández-Espinilla, V., Liang, C., Begier, E., Castrodeza Sanz, J., Eiros, J. M., & Sanz-Muñoz, I. (2024). RSV Risk Profile in Hospitalized Adults and Comparison with Influenza and COVID-19 Controls in Valladolid, Spain, 2010-2022. Infectious Diseases and Therapy, 13(9). https://doi.org/10.1007/s40121-024-01021-1

Haeusser, S., Möller, R., Smarsly, K., Al-Hakim, Y., Kreuzinger, N., Pinnekamp, J., Pletz, M., Kluemper, C., & Beier, S. (2023). SARS-CoV-2 Wastewater Monitoring in Thuringia, Germany : Analytical Aspects and Normalization of Results. WATER, 15(24). https://doi.org/10.3390/w15244290

Haga, L., Ruuhela, R., Auranen, K., Lakkala, K., Heikkilä, A., & Gregow, H. (2022). Impact of Selected Meteorological Factors on COVID-19 Incidence in Southern Finland during 2020-2021. International Journal of Environmental Research and Public Health, 19(20). https://doi.org/10.3390/ijerph192013398

Hagen, T. L., Nitschke, M. J., & Smit, J. (2025). Respiratory syncytial virus : Characteristics, complications and mortality in immunocompetent versus immunocompromised hospitalized adults in Northern Denmark. Diagnostic Microbiology and Infectious Disease, 111(2). https://doi.org/10.1016/j.diagmicrobio.2024.116605

Hagenbeck, C., Zöllkau, J., Helbig, M., Fehm, T., & Schaal, N. (2023). COVID-19 Vaccination during Pregnancy and Lactation : Attitudes and Uptakes before and after Official Recommendations in Germany. VACCINES, 11(3). https://doi.org/10.3390/vaccines11030627

Haim-Boukobza, S., Roquebert, B., Trombert-Paolantoni, S., Lecorche, E., Verdurme, L., Foulongne, V., Selinger, C., Michalakis, Y., Sofonea, M. T., & Alizon, S. (2021). Detecting Rapid Spread of SARS-CoV-2 Variants, France, January 26-February 16, 2021. Emerging Infectious Diseases, 27(5). https://doi.org/10.3201/eid2705.210397

Hak, E., Meijboom, M. J., & Buskens, E. (2006). Modelling the health-economic impact of the next influenza pandemic in The Netherlands. Vaccine, 24(44), 6756–6760. https://doi.org/10.1016/j.vaccine.2006.05.065

Halford, F., Yates, K., Clare, T., Lopez-Bernal, J., Kall, M., & Allen, H. (2024). Temporal changes to adult case fatality risk of COVID-19 after vaccination in England between May 2020 and February 2022 : A national surveillance study. Journal of the Royal Society of Medicine, 117(6). https://doi.org/10.1177/01410768231216332

Hall, J. A., Harris, R. J., Zaidi, A., Dabrera, G., & Dunbar, J. K. (2022). Risk of hospitalisation or death in households with a case of COVID-19 in England : An analysis using the HOSTED data set. Public Health, 211. https://doi.org/10.1016/j.puhe.2022.07.013

Hall, J. A., Harris, R. J., Zaidi, A., Woodhall, S. C., Dabrera, G., & Dunbar, J. K. (2021). HOSTED-England's Household Transmission Evaluation Dataset : Preliminary findings from a novel passive surveillance system of COVID-19. International Journal of Epidemiology, 50(3). https://doi.org/10.1093/ije/dyab057

Hall, V. J., Foulkes, S., Saei, A., Andrews, N., Oguti, B., Charlett, A., Wellington, E., Stowe, J., Gillson, N., Atti, A., Islam, J., Karagiannis, I., Munro, K., Khawam, J., Chand, M. A., Brown, C. S., Ramsay, M., Lopez-Bernal, J., Hopkins, S., & SIREN Study Group. (2021). COVID-19 vaccine coverage in health-care workers in England and effectiveness of BNT162b2 mRNA vaccine against infection (SIREN) : A prospective, multicentre, cohort study. Lancet (London, England), 397(10286). https://doi.org/10.1016/S0140-6736(21)00790-X

Hallauer, J. F., & Neuschaefer-Rube, N. (2005). Influenza vaccination of hospital staff in Germany : A five-year survey on vaccination coverage and policies : Identified deficits in influenza immunisation campaigns for hospital employees. Sozial- Und Praventivmedizin, 50(1). https://doi.org/10.1007/s00038-004-3103-1

Hämäläinen, A., Patovirta, R.-L., Vuorinen, S., Leppäaho-Lakka, J., Kilpinen, S., Sieberns, J., Ruotsalainen, E., Koivula, I., & Hämäläinen, S. (2024). COVID-19 vaccination among health care workers in Finland : Coverage, perceptions and attitudes. Scandinavian Journal of Public Health, 52(3). https://doi.org/10.1177/14034948231203779

Hämäläinen, A., Savinainen, E., Hämäläinen, S., Sivenius, K., Kauppinen, J., Koivula, I., & Patovirta, R.-L. (2022). Disease burden caused by respiratory syncytial virus compared with influenza among adults : A retrospective cohort study from Eastern Finland in 2017-2018. BMJ Open, 12(12). https://doi.org/10.1136/bmjopen-2022-060805

Hamm, S. R., Rezahosseini, O., Møller, D. L., Loft, J. A., Poulsen, J. R., Knudsen, J. D., Pedersen, M. S., Schønning, K., Harboe, Z. B., Rasmussen, A., Sørensen, S. S., & Nielsen, S. D. (2022). Incidence and severity of SARS-CoV-2 infections in liver and kidney transplant recipients in the post-vaccination era : Real-life data from Denmark. American Journal of Transplantation: Official Journal of the American Society of Transplantation and the American Society of Transplant Surgeons, 22(11). https://doi.org/10.1111/ajt.17141

Hammer, C. C., Cristea, V., Dub, T., & Sivelä, J. (2021). High but slightly declining COVID-19 vaccine acceptance and reasons for vaccine acceptance, Finland April to December 2020. Epidemiology and Infection, 149. https://doi.org/10.1017/S0950268821001114

Hammer, C. C., Lyytikäinen, O., Arifulla, D., Toura, S., & Nohynek, H. (2022). High influenza vaccination coverage among healthcare workers in acute care hospitals in Finland, seasons 2017/18, 2018/19 and 2019/20. Euro Surveillance: Bulletin Europeen Sur Les Maladies Transmissibles = European Communicable Disease Bulletin, 27(17). https://doi.org/10.2807/1560-7917.ES.2022.27.17.2100411

Han, A. X., Kozanli, E., Koopsen, J., Vennema, H., RIVM COVID-19 molecular epidemiology group, Hajji, K., Kroneman, A., van Walle, I., Klinkenberg, D., Wallinga, J., Russell, C. A., Eggink, D., & Reusken, C. (2022). Regional importation and asymmetric within-country spread of SARS-CoV-2 variants of concern in the Netherlands. eLife, 11. https://doi.org/10.7554/eLife.78770

Hâncean, M.-G., Perc, M., & Lerner, J. (2020). Early spread of COVID-19 in Romania: Imported cases from Italy and human-to-human transmission networks. Royal Society Open Science, 7(7), 200780. https://doi.org/10.1098/rsos.200780

Haneef, R., Fayad, M., Fouillet, A., Sommen, C., Bonaldi, C., Wyper, G. M. A., Pires, S. M., Devleesschauwer, B., Rachas, A., Constantinou, P., Levy-Bruhl, D., Beltzer, N., & Gallay, A. (2023). Direct impact of COVID-19 by estimating disability-adjusted life years at national level in France in 2020. PloS One, 18(1). https://doi.org/10.1371/journal.pone.0280990

Hanrath, A. T., Schim van der Loeff, I., Lendrem, D. W., Baker, K. F., Price, D. A., McDowall, P., McDowall, K., Cook, S., Towns, P., Schwab, U., Evans, A., Dixon, J., Collins, J., Burton-Fanning, S., Saunders, D., Harwood, J., Samuel, J., Schmid, M. L., Pareja-Cebrian, L., … Duncan, C. J. A. (2021). SARS-CoV-2 Testing of 11,884 Healthcare Workers at an Acute NHS Hospital Trust in England : A Retrospective Analysis. Frontiers in Medicine, 8. https://doi.org/10.3389/fmed.2021.636160

Hanslik, T., Boelle, P.-Y., & Flahault, A. (2010). Preliminary estimation of risk factors for admission to intensive care units and for death in patients infected with A(H1N1)2009 influenza virus, France, 2009-2010. PLoS Currents, 2. https://doi.org/10.1371/currents.rrn1150

Hao, Y., Yan, G., Ma, R., & Hasan, M. T. (2021). Linking dynamic patterns of COVID-19 spreads in Italy with regional characteristics : A two level longitudinal modelling approach. Mathematical Biosciences and Engineering: MBE, 18(3). https://doi.org/10.3934/mbe.2021131

Harcourt, S. E., Morbey, R. A., Smith, G. E., Loveridge, P., Green, H. K., Pebody, R., Rutter, J., Yeates, F. A., Stuttard, G., & Elliot, A. J. (2019). Developing influenza and respiratory syncytial virus activity thresholds for syndromic surveillance in England. Epidemiology and Infection, 147. https://doi.org/10.1017/S0950268819000542

Hardelid, P., Fleming, D. M., Andrews, N., Barley, M., Durnall, H., Mangtani, P., & Pebody, R. (2012). Effectiveness of trivalent and pandemic influenza vaccines in England and Wales 2008-2010: Results from a cohort study in general practice. Vaccine, 30(7), 1371–1378. https://doi.org/10.1016/j.vaccine.2011.12.038

Hardelid, P., Fleming, D., McMenamin, J., Andrews, N., Robertson, C., SebastianPillai, P., Ellis, J., Carman, W., Wreghitt, T., Watson, J., & Pebody, R. (2011). Effectiveness of pandemic and seasonal influenza vaccine in preventing pandemic influenza A(H1N1)2009 infection in England and Scotland 2009-2010. EUROSURVEILLANCE, 16(2), 12–18.

Hardelid, P., Kapetanstrataki, M., Norman, L., Fleming, S. J., Lister, P., Gilbert, R., & Parslow, R. C. (2018a). Characteristics and mortality risk of children with life-threatening influenza infection admitted to paediatric intensive care in England 2003-2015. Respiratory Medicine, 137. https://doi.org/10.1016/j.rmed.2018.02.012

Hardelid, P., Kapetanstrataki, M., Norman, L., Fleming, S. J., Lister, P., Gilbert, R., & Parslow, R. C. (2018b). Impact of the introduction of a universal childhood influenza vaccination programme on influenza-related admissions to paediatric intensive care units in England. BMJ Open Respiratory Research, 5(1). https://doi.org/10.1136/bmjresp-2018-000297

Hardelid, P., Pebody, R., & Andrews, N. (2013). Mortality caused by influenza and respiratory syncytial virus by age group in England and Wales 1999-2010. Influenza and Other Respiratory Viruses, 7(1). https://doi.org/10.1111/j.1750-2659.2012.00345.x

Hardelid, P., Rait, G., Gilbert, R., & Petersen, I. (2016). Factors associated with influenza vaccine uptake during a universal vaccination programme of preschool children in England and Wales : A cohort study. Journal of Epidemiology and Community Health, 70(11). https://doi.org/10.1136/jech-2015-207014

Harder, K. M., Andersen, P. H., Bæhr, I., Nielsen, L. P., Ethelberg, S., Glismann, S., & Molbak, K. (2011). Electronic real-time surveillance for influenza-like illness: Experience from the 2009 influenza A(H1N1) pandemic in Denmark. Euro Surveillance: Bulletin Europeen Sur Les Maladies Transmissibles = European Communicable Disease Bulletin, 16(3), 19767.

Harman, K., Nash, S. G., Webster, H. H., Groves, N., Hardstaff, J., Bridgen, J., Blomquist, P. B., Hope, R., Ashano, E., Myers, R., Rokadiya, S., Hopkins, S., Brown, C. S., Chand, M., Dabrera, G., & Thelwall, S. (2022). Comparison of the risk of hospitalisation among BA.1 and BA.2 COVID-19 cases treated with Sotrovimab in the community in England. medRxiv, (Harman K., katie.harman@ukhsa.gov.uk; Nash S.G.; Webster H.H.; Hope R.; Dabrera G.; Thelwall S.) UKHSA, COVID-19 National Epidemiology Cell, London, United Kingdom. https://doi.org/10.1101/2022.10.21.22281171

Harries, M., Jaeger, V. K., Rodiah, I., Hassenstein, M. J., Ortmann, J., Dreier, M., von Holt, I., Brinkmann, M., Dulovic, A., Gornyk, D., Hovardovska, O., Kuczewski, C., Kurosinski, M.-A., Schlotz, M., Schneiderhan-Marra, N., Strengert, M., Krause, G., Sester, M., Klein, F., … Lange, B. (2024). Bridging the gap—Estimation of 2022/2023 SARS-CoV-2 healthcare burden in Germany based on multidimensional data from a rapid epidemic panel. International Journal of Infectious Diseases: IJID: Official Publication of the International Society for Infectious Diseases, 139. https://doi.org/10.1016/j.ijid.2023.11.014

Harrison, C., Frain, S., Jalalinajafabadi, F., Williams, S. G., Keavney, B., & CVD-COVID-UK/COVID-IMPACT consortium. (2024). The impact of COVID-19 vaccination on patients with congenital heart disease in England : A case-control study. Heart (British Cardiac Society), 110(23). https://doi.org/10.1136/heartjnl-2024-324470

Harrison, D. A., Watkinson, P. J., Doidge, J. C., Shankar-Hari, M., Mouncey, P. R., Patone, M., Coupland, C. A. C., Hippisley-Cox, J., & Rowan, K. M. (2022). Impact of vaccination on COVID-19-associated admissions to critical care in England : A population cohort study of linked data. medRxiv, (Harrison D.A., david.harrison@icnarc.org; Doidge J.C.; Mouncey P.R.; Rowan K.M.) Intensive Care National Audit&Research Centre, London, United Kingdom. https://doi.org/10.1101/2022.10.03.22280649

Harrison, G., Newport, D., Robbins, T., Arvanitis, T. N., & Stein, A. (2020). Mortality statistics in England and Wales : The SARS-CoV-2 paradox. The Journal of International Medical Research, 48(6). https://doi.org/10.1177/0300060520931298

Harteloh, P., & van Mechelen, R. (2024). Mortality Rates of the Spanish Flu and Coronavirus Disease 2019 in the Netherlands : A Historical Comparison. The Journal of Infectious Diseases, 230(1). https://doi.org/10.1093/infdis/jiae071

Hartonen, T., Jermy, B., Sõnajalg, H., Vartiainen, P., Krebs, K., Vabalas, A., FinnGen, Estonian Biobank Research Team, Leino, T., Nohynek, H., Sivelä, J., Mägi, R., Daly, M., Ollila, H. M., Milani, L., Perola, M., Ripatti, S., & Ganna, A. (2023). Nationwide health, socio-economic and genetic predictors of COVID-19 vaccination status in Finland. Nature Human Behaviour, 7(7). https://doi.org/10.1038/s41562-023-01591-z

Harvala, H., Carnahan, A., Axelsson, S., & Brytting, M. (2018). Evaluation of the national laboratory-based surveillance system for respiratory syncytial virus in Sweden, 2015-2016. Journal of Clinical Virology: The Official Publication of the Pan American Society for Clinical Virology, 104. https://doi.org/10.1016/j.jcv.2018.04.004

Hasegawa, K., Jartti, T., Mansbach, J. M., Laham, F. R., Jewell, A. M., Espinola, J. A., Piedra, P. A., & Camargo, C. A. (2015). Respiratory syncytial virus genomic load and disease severity among children hospitalized with bronchiolitis : Multicenter cohort studies in the United States and Finland. The Journal of Infectious Diseases, 211(10). https://doi.org/10.1093/infdis/jiu658

Hashim, A. B., McKeever, T., Kelly, S. J., & Nguyen-Van-Tam, J. S. (2010). Evaluation of inter-pandemic influenza vaccine effectiveness during eight consecutive winter seasons in England and Wales in patients with cardiovascular risk factors. Journal of Infection and Public Health, 3(4), 159–165. https://doi.org/10.1016/j.jiph.2010.09.007

Hassan, S., Ferrari, B., Rossio, R., la Mura, V., Artoni, A., Gualtierotti, R., Martinelli, I., Nobili, A., Bandera, A., Gori, A., Blasi, F., Monzani, V., Costantino, G., Harari, S., Rosendaal, F. R., Peyvandi, F., & COVID-19 Network working group. (2022). The usefulness of D-dimer as a predictive marker for mortality in patients with COVID-19 hospitalized during the first wave in Italy. PloS One, 17(7). https://doi.org/10.1371/journal.pone.0264106

Hausmann, J., Dörre, A., Katz, K., & van de Berg, S. (2024). Lifting COVID-19-associated non-pharmaceutical interventions : Potential impact on notifications of infectious diseases transmitted from person to person in 2022 in Bavaria, Germany. Frontiers in Public Health, 12. https://doi.org/10.3389/fpubh.2024.1437485

Haverkate, M. R., de Kassteele, J. V., Hof, S. V. D., Sanders, J. G., Lambooij, M. S., de Bruin, M., de Melker, H. E., & Hahné, S. J. M. (2025). The effect of pre-booked appointments on COVID-19 vaccine uptake during the 2023 autumn campaign in the Netherlands: A regression discontinuity analysis. medRxiv. https://doi.org/10.1101/2025.07.10.25331000

Heald, A. H., Jenkins, D. A., Williams, R., Mudaliar, R. N., Khan, A., Syed, A., Sattar, N., Khunti, K., Naseem, A., Bowden-Davies, K. A., Gibson, J. M., Ollier, W., & CVD-COVID-UK/COVID-IMPACT Consortium. (2023). Sars-Cov-2 Infection in People with Type 1 Diabetes and Hospital Admission : An Analysis of Risk Factors for England. Diabetes Therapy: Research, Treatment and Education of Diabetes and Related Disorders, 14(12). https://doi.org/10.1007/s13300-023-01456-8

Hecker, K. J., Bethe, H., Engels, G., Knies, K., Krempl, C., Weissbrich, B., Liese, J., & Streng, A. (2025). Elucidating the Outpatient Burden of Lower Respiratory Tract Infections Due to Rsv and Other Viruses in Children Under 2 Years of Age: Clinical Presentation, Medical Treatment, and Family Impact (Germany, 11/2022-10/2024). SSRN. https://doi.org/10.2139/ssrn.5216280

Heese, H., Marquis, A., Diercke, M., Markus, I., Böhm, S., Metz, J., Katz, K., Wildner, M., & Liebl, B. (2022). Results of the enhanced COVID-19 surveillance during UEFA EURO 2020 in Germany. Epidemiology and Infection, 150. https://doi.org/10.1017/S0950268822000449

Heibati, B., Wang, W., Ryti, N. R. I., Dominici, F., Ducatman, A., Zhang, Z., & Jaakkola, J. J. K. (2020). Weather Conditions and COVID-19 Incidence in a Cold Climate : A Time-Series Study in Finland. Frontiers in Public Health, 8. https://doi.org/10.3389/fpubh.2020.605128

Heider, A., Wedde, M., Dürrwald, R., Wolff, T., & Schweiger, B. (2022). Molecular characterization and evolution dynamics of influenza B viruses circulating in Germany from season 1996/1997 to 2019/2020. VIRUS RESEARCH, 322. https://doi.org/10.1016/j.virusres.2022.198926

Heijnen, L., & Medema, G. (2011). Surveillance of influenza A and the pandemic influenza A (H1N1) 2009 in sewage and surface water in the Netherlands. Journal of Water and Health, 9(3), 434–442. https://doi.org/10.2166/wh.2011.019

Hein, A., Kehl, S., Häberle, L., Tiemann, C., Peuker, R., Mereutanu, D., Stumpfe, F. M., Faschingbauer, F., Meyer-Schlinkmann, K., Koch, M. C., Kainer, F., Dammer, U., Philipp, H., Kladt, C., Schrauder, M. G., Weingärtler, S., Hanf, V., Hartmann, A., Rübner, M., … Schneider, M. O. (2022). Prevalence of SARS-CoV-2 in Pregnant Women Assessed by RT-PCR in Franconia, Germany : First Results of the SCENARIO Study (SARS-CoV-2 prEvalence in pregNAncy and at biRth In FrancOnia). Geburtshilfe Und Frauenheilkunde, 82(2). https://doi.org/10.1055/a-1727-9672

Heinemeier, D., Schmid, P., Eitze, S., & Betsch, C. (2025). Influenza and pneumococcal vaccine hesitancy in the elderly population: Results from two representative surveys in Germany. BMC Public Health, 25(1), 1672. https://doi.org/10.1186/s12889-025-22441-9

Heins, M. J., Spreeuwenberg, P., Caini, S., Hooiveld, M., Meijer, A., & Paget, J. (2024). Measuring the impact of influenza vaccination in the Netherlands using retrospective observational primary care, hospitalisation and mortality data. Vaccine, 42(26). https://doi.org/10.1016/j.vaccine.2024.126244

Heireman, L., Abrams, S., Bruynseels, P., Cartuyvels, R., Cuypers, L., De Schouwer, P., Laffut, W., Lagrou, K., Hens, N., Ho, E., Padalko, E., Reynders, M., Vandamme, S., Van der Moeren, N., Verstrepen, W., Willems, P., & Naesens, R. (2021). Evaluation of the SARS-CoV-2 positivity ratio and upper respiratory tract viral load among asymptomatic individuals screened before hospitalization or surgery in Flanders, Belgium. PloS One, 16(11). https://doi.org/10.1371/journal.pone.0259908

Hélène, B., Marie-Cécile, L., Nada, A., Pascal, C., Alexandre, D., Karine, M., Jacques, G., Gaétan, G., Benjamin, G., Odile, L., Anne, M., Fanny, R., Laurence, W., Rebecca, H. C., & Ayman, C. (2023). The relative effectiveness of a high-dose quadrivalent influenza vaccine vs standard-dose quadrivalent influenza vaccines in older adults in France : A retrospective cohort study during the 2021-22 influenza season. medRxiv, (Hélène B., Helene.bricout@sanofi.com; Marie-Cécile L.; Karine M.; Ayman C.) Sanofi Vaccines, France. https://doi.org/10.1101/2023.06.15.23291345

Helmeke, C., Gräfe, L., Irmscher, H., Gottschalk, C., Karagiannis, L., & Oppermann, H. (2015). Effectiveness of the 2012/13 Trivalent Live and Inactivated Influenza Vaccines in Children and Adolescents in Saxony-Anhalt, Germany : A Test-Negative Case-Control Study. PLOS ONE, 10(4). https://doi.org/10.1371/journal.pone.0122910

Heppe-Montero, M., Gil-Prieto, R., Del Diego Salas, J., Hernández-Barrera, V., & Gil-de-Miguel, Á. (2022). Impact of Respiratory Syncytial Virus and Influenza Virus Infection in the Adult Population in Spain between 2012 and 2020. International Journal of Environmental Research and Public Health, 19(22). https://doi.org/10.3390/ijerph192214680

Heppe-Montero, M., Walter, S., Hernández-Barrera, V., Gil-Prieto, R., & Gil-de-Miguel, Á. (2022). Burden of respiratory syncytial virus-associated lower respiratory infections in children in Spain from 2012 to 2018. BMC Infectious Diseases, 22(1). https://doi.org/10.1186/s12879-022-07261-1

Hergens, M.-P., Baum, U., Brytting, M., Ikonen, N., Haveri, A., Wiman, Å., Nohynek, H., & Örtqvist, Å. (2017). Mid-season real-time estimates of seasonal influenza vaccine effectiveness in persons 65 years and older in register-based surveillance, Stockholm County, Sweden, and Finland, January 2017. Euro Surveillance: Bulletin Europeen Sur Les Maladies Transmissibles = European Communicable Disease Bulletin, 22(8). https://doi.org/10.2807/1560-7917.ES.2017.22.8.30469

Herkenrath, S. D., Boschung, K., Nacov, J. A., Heibges, A., Schroer, B., Treml, M., & Randerath, W. J. (2022). Initial Proportion and Dynamic of B.1.1.7 SARS-CoV-2 in a Large City in the West of Germany. Biomedicine Hub, 7(1). https://doi.org/10.1159/000519968

Hernández-García, I., Chaure-Pardos, A., Aibar-Remón, C., Garcia-Montero, J., Prieto, P., Mareca, R., Moliner, J., Barrasa, I., Félix, M., & Grp Trabajovacunas HCULB. (2019). Influenza vaccination coverages and related factors among splenectomy patients from a health sector in Zaragoza (Spain). REVISTA ESPANOLA DE SALUD PUBLICA, 93.

Hernández-Negrín, H., Bernal-López, M. R., López-Sampalo, A., Rubio-Rivas, M., Aguilar-García, J. A., Gómez-Uranga, A., Carnevali, M., Taboada-Martínez, M. L., Ramos-Rincón, J.-M., Gómez-Huelgas, R., list of members of the SEMI-COVID-19 Network, & Appendix A. List of members of the SEMI-COVID-19 Network. (2025). Cardiovascular profile of systemic lupus erythematosus patients hospitalized for COVID-19 in Spain : Analysis of the SEMI-COVID-19 Registry. Medicina Clinica. https://doi.org/10.1016/j.medcli.2024.11.022

Herrmann, B. L. (2020). The prevalence rate of anti-SARS-CoV-2-IgG is 1.2%—Screening in asymptomatic outpatients in Germany (Northrhine-Westfalia). MMW-Fortschritte der Medizin, 162(14). Embase. https://doi.org/10.1007/s15006-020-0750-y

Hetebrij, W. A., de Roda Husman, A. M., Nagelkerke, E., van der Beek, R. F. H. J., van Iersel, S. C. J. L., Breuning, T. G. V., Lodder, W. J., & van Boven, M. (2024). Inferring hospital admissions from SARS-CoV-2 virus loads in wastewater in The Netherlands, August 2020—February 2022. The Science of the Total Environment, 912. https://doi.org/10.1016/j.scitotenv.2023.168703

Hetemäki, I., Kääriäinen, S., Alho, P., Mikkola, J., Savolainen-Kopra, C., Ikonen, N., Nohynek, H., & Lyytikäinen, O. (2021). An outbreak caused by the SARS-CoV-2 Delta variant (B.1.617.2) in a secondary care hospital in Finland, May 2021. Euro Surveillance: Bulletin Europeen Sur Les Maladies Transmissibles = European Communicable Disease Bulletin, 26(30). https://doi.org/10.2807/1560-7917.ES.2021.26.30.2100636

Hetland, G., Fagerhol, M. K., Mirlashari, M. R., Nissen-Meyer, L. S. H., Croci, S., Lonati, P. A., Bonacini, M., Salvarani, C., Marvisi, C., Bodio, C., Muratore, F., Borghi, M. O., & Meroni, P. L. (2024). Elevated NET, Calprotectin, and Neopterin Levels Discriminate between Disease Activity in COVID-19, as Evidenced by Need for Hospitalization among Patients in Northern Italy. Biomedicines, 12(4). https://doi.org/10.3390/biomedicines12040766

Heudorf, U., & Kowall, B. (2025). Mortality in Frankfurt am Main, Germany, 2020-2023: Higher excess mortality during an influenza wave in 2022 than during all COVID-19 waves altogether. GMS Hygiene and Infection Control, 20, Doc04. https://doi.org/10.3205/dgkh000533

Heudorf, U., Domann, E., Förner, M., Kunz, S., Latasch, L., Trost, B., & Steul, K. (2023). Development of morbidity and mortality of SARS-CoV-2 in nursing homes for the elderly in Frankfurt am Main, Germany, 2020-2022 : What protective measures are still required? GMS Hygiene and Infection Control, 18. https://doi.org/10.3205/dgkh000431

Heudorf, U., Müller, M., Schmehl, C., Gasteyer, S., & Steul, K. (2020). COVID-19 in long-term care facilities in Frankfurt am Main, Germany : Incidence, case reports, and lessons learned. GMS Hygiene and Infection Control, 15. https://doi.org/10.3205/dgkh000361

Heyd, R., Eis-Hübinger, A. M., Berger, A., Bierbaum, S., Pietzonka, S., Wenzel, J. J., Huzly, D., Keppler, O. T., & Panning, M. (2017). Retrospective analysis of clinical and virological parameters of influenza cases at four university hospitals in Germany, 2015. Infection, 45(3). https://doi.org/10.1007/s15010-017-1008-1

Hiam, L., McKee, M., & Dorling, D. (2024). Influenza : Cause or excuse ? An analysis of flu's influence on worsening mortality trends in England and Wales, 2010-19. British Medical Bulletin, 149(1). https://doi.org/10.1093/bmb/ldad028

Hiam, L., Minton, J., Burns, R., McKee, M., & Aldridge, R. W. (2024). To what extent did mortality from COVID-19 in England and Wales differ for migrants compared to non-migrants in 2020 and 2021 ? A descriptive, observational study. European Journal of Public Health, 34(6). https://doi.org/10.1093/eurpub/ckae142

Hildebrandt, A., Dolega, K., Uflacker, L., Rudolf, H., & Gatermann, S. G. (2024). SARS-CoV-2 infections in patients, health care workers and hospital outbreaks during the first 3 waves of the pandemic: A retrospective analysis in a secondary care hospital network in Germany. BMC Infectious Diseases, 24(1), 859. https://doi.org/10.1186/s12879-024-09641-1

Hildebrandt, A., Hökelekli, O., Uflacker, L., Rudolf, H., & Gatermann, S. G. (2021). COVID-19 : Hotspot hospital?- seroprevalence of SARS-CoV-2 antibodies in hospital employees in a secondary care hospital network in Germany : Intermediate results of a prospective surveillance study. International Journal of Hygiene and Environmental Health, 235. https://doi.org/10.1016/j.ijheh.2021.113771

Hildebrandt, A., Hökelekli, O., Uflacker, L., Rudolf, H., Paulussen, M., & Gatermann, S. G. (2022). Seroprevalence of SARS-CoV-2 Antibodies in Employees of Three Hospitals of a Secondary Care Hospital Network in Germany and an Associated Fire Brigade : Results of a Repeated Cross-Sectional Surveillance Study Over 1 Year. International Journal of Environmental Research and Public Health, 19(4). https://doi.org/10.3390/ijerph19042402

Hill, E. M., Petrou, S., Forster, H., de Lusignan, S., Yonova, I., & Keeling, M. J. (2020). Optimising age coverage of seasonal influenza vaccination in England : A mathematical and health economic evaluation. PLoS Computational Biology, 16(10). https://doi.org/10.1371/journal.pcbi.1008278

Hinch, R., Panovska-Griffiths, J., Probert, W. J. M., Ferretti, L., Wymant, C., Di Lauro, F., Baya, N., Ghafari, M., Abeler-Dörner, L., COVID-19 Genomics UK (COG-UK) Consortium, & Fraser, C. (2022). Estimating SARS-CoV-2 variant fitness and the impact of interventions in England using statistical and geo-spatial agent-

based models. Philosophical Transactions. Series A, Mathematical, Physical, and Engineering Sciences, 380(2233). https://doi.org/10.1098/rsta.2021.0304

Hirst, J., Mi, E., Copland, E., Patone, M., Coupland, C., & Hippisley-Cox, J. (2023). Uptake of COVID-19 vaccination in people with blood cancer : Population-level cohort study of 12 million patients in England. European Journal of Cancer (Oxford, England: 1990), 183. https://doi.org/10.1016/j.ejca.2023.02.001

Ho, C.-C., Hung, S.-C., & Ho, W.-C. (2021). Effects of short- and long-term exposure to atmospheric pollution on COVID-19 risk and fatality : Analysis of the first epidemic wave in northern Italy. Environmental Research, 199. https://doi.org/10.1016/j.envres.2021.111293

Ho, J., Stange, C., Suhrborg, R., Wurzbacher, C., Drewes, J. E., & Tiehm, A. (2022). SARS-CoV-2 wastewater surveillance in Germany : Long-term RT-digital droplet PCR monitoring, suitability of primer/probe combinations and biomarker stability. Water Research, 210. https://doi.org/10.1016/j.watres.2021.117977

Hoang, V.-T., Colson, P., Levasseur, A., Delerce, J., Lagier, J.-C., Parola, P., Million, M., Fournier, P.-E., Raoult, D., & Gautret, P. (2021). Clinical outcomes in patients infected with different SARS-CoV-2 variants at one hospital during three phases of the COVID-19 epidemic in Marseille, France. Infection, Genetics and Evolution: Journal of Molecular Epidemiology and Evolutionary Genetics in Infectious Diseases, 95. https://doi.org/10.1016/j.meegid.2021.105092

Hobohm, L., Sagoschen, I., Barco, S., Schmidtmann, I., Espinola-Klein, C., Konstantinides, S., Münzel, T., & Keller, K. (2022). Trends and Risk Factors of In-Hospital Mortality of Patients with COVID-19 in Germany : Results of a Large Nationwide Inpatient Sample. Viruses, 14(2). https://doi.org/10.3390/v14020275

Hoch, M., Vogel, S., Kolberg, L., Dick, E., Fingerle, V., Eberle, U., Ackermann, N., Sing, A., Huebner, J., Rack-Hoch, A., Schober, T., & von Both, U. (2021). Weekly SARS-CoV-2 Sentinel Surveillance in Primary Schools, Kindergartens, and Nurseries, Germany, June–November 2020. Emerging Infectious Diseases, 27(8). https://doi.org/10.3201/eid2708.204859

Hodgson, D., Baguelin, M., van Leeuwen, E., Panovska-Griffiths, J., Ramsay, M., Pebody, R., & Atkins, K. E. (2017). Effect of mass paediatric influenza vaccination on existing influenza vaccination programmes in England and Wales : A modelling and cost-effectiveness analysis. The Lancet. Public Health, 2(2). https://doi.org/10.1016/S2468-2667(16)30044-5

Hoebel, J., Busch, M. A., Grabka, M. M., Zinn, S., Allen, J., Göfêwald, A., Wernitz, J., Goebel, J., Steinhauer, H. W., Siegers, R., Schroder, C., Kuttig, T., Butschalowsky, H., Schlaud, M., Rosario, A. S., Brix, J., Rysina, A., Glemser, A., Neuhauser, H., … Lampert, T. (2021). Seroepidemiological study on the spread of SARS-CoV-2 in Germany : Study protocol of the CORONA-MONITORING bundesweit' study (RKI-SOEP study). Journal of Health Monitoring, 6(Suppl 1). https://doi.org/10.25646/7853

Hoebel, J., Michalski, N., Diercke, M., Hamouda, O., Wahrendorf, M., Dragano, N., & Nowossadeck, E. (2021). Emerging socio-economic disparities in COVID-19-related deaths during the second pandemic wave in Germany. International Journal of Infectious Diseases: IJID: Official Publication of the International Society for Infectious Diseases, 113, 344–346. https://doi.org/10.1016/j.ijid.2021.10.037

Hoeve, C. E., de Gier, B., Huiberts, A. J., de Melker, H. E., Hahné, S. J. M., van den Hof, S., & Knol, M. J. (2023). Vaccine effectiveness against SARS-CoV-2 Delta and Omicron infection and infectiousness within households in the Netherlands between July 2021 and August 2022. medRxiv, (Hoeve C.E., christel.hoeve@rivm.nl; de Gier B.; Huiberts A.J.; Melker H.E.; Hahné S.J.M.; van den Hof S.; Knol M.J.) Centre for Infectious Disease Control, National Institute for Public Health and the Environment, Bilthoven, Netherlands. https://doi.org/10.1101/2023.01.10.23284386

Hoffmann, K., Paczkowska, A., Michalak, M., Jarząb, M., Bryl, W., Nowakowska, E., Kus, K., Ratajczak, P., Zaprutko, T., & Kopciuch, D. (2023). Impact of the SARS-CoV-2 Vaccination Program on Presenteeism and Absenteeism among Healthcare Workers in Poland. Vaccines, 12(1). https://doi.org/10.3390/vaccines12010023

Hoffmann, S., Schiebel, J., Hufert, F., Gremmels, H.-D., & Spallek, J. (2021). COVID-19 among Healthcare Workers : A Prospective Serological-Epidemiological Cohort Study in a Standard Care Hospital in Rural

Germany. International Journal of Environmental Research and Public Health, 18(20). https://doi.org/10.3390/ijerph182010999

Hohmuth, N., Khanyaree, I., Lang, A.-L., Duering, O., Konigorski, S., Višković, V., Heising, T., Egender, F., Remschmidt, C., & Leistner, R. (2022). Participatory disease surveillance for a mass gathering—A prospective cohort study on COVID-19, Germany 2021. BMC Public Health, 22(1). https://doi.org/10.1186/s12889-022-14505-x

Hokajärvi, A.-M., Rytkönen, A., Tiwari, A., Kauppinen, A., Oikarinen, S., Lehto, K.-M., Kankaanpää, A., Gunnar, T., Al-Hello, H., Blomqvist, S., Miettinen, I. T., Savolainen-Kopra, C., & Pitkänen, T. (2021). The detection and stability of the SARS-CoV-2 RNA biomarkers in wastewater influent in Helsinki, Finland. The Science of the Total Environment, 770. https://doi.org/10.1016/j.scitotenv.2021.145274

Holler, J. G., Eriksson, R., Jensen, T. Ø., van Wijhe, M., Fischer, T. K., Søgaard, O. S., Israelsen, S. B., Mohey, R., Fabricius, T., Jøhnk, F., Wiese, L., Johnsen, S., Søborg, C., Nielsen, H., Kirk, O., Madsen, B. L., & Harboe, Z. B. (2021). First wave of COVID-19 hospital admissions in Denmark : A Nationwide population-based cohort study. BMC Infectious Diseases, 21(1). https://doi.org/10.1186/s12879-020-05717-w

Holleyman, R. J., Khan, S. K., Charlett, A., Inman, D. S., Johansen, A., Brown, C., Barnard, S., Fox, S., Baker, P. N., Deehan, D., Burton, P., & Gregson, C. L. (2022). The impact of COVID-19 on mortality after hip fracture : A population cohort study from England. The Bone & Joint Journal, 104-B(10). https://doi.org/10.1302/0301-620X.104B10.BJJ-2022-0082.R1

Holm, M. V., Arrazola Martínez, M. P., Szucs, T. D., De Juanes, J. R., & García De Codes, A. (2007). Evolution of influenza vaccine coverage in Spain (2002-2006). Vacunas, 8(2). Embase. https://www.embase.com/search/results?subaction=viewrecord&id=L47501098&from=export

Holm, M. V., Blank, P. R., & Szucs, T. D. (2007). Trends in influenza vaccination coverage rates in Germany over five seasons from 2001 to 2006. BMC Infectious Diseases, 7, 144. https://doi.org/10.1186/1471-2334-7-144

Holm, M. V., Blank, P. R., & Szucs, T. D. (2007a). Developments in influenza vaccination coverage in England, Scotland and Wales covering five consecutive seasons from 2001 to 2006. Vaccine, 25(46). https://doi.org/10.1016/j.vaccine.2007.09.022

Holm, M. V., Blank, P. R., & Szucs, T. D. (2007b). Trends in influenza vaccination coverage rates in Germany over five seasons from 2001 to 2006. BMC Infectious Diseases, 7. https://doi.org/10.1186/1471-2334-7-144

Holm, M. V., Szucs, T. D., & Fara, G. M. (2007). Developments in influenza vaccination coverage in Italy over five seasons (2001-2006). Annali Di Igiene: Medicina Preventiva E Di Comunita, 19(5). http://www.ncbi.nlm.nih.gov/pubmed/18210771

Holman, N., Knighton, P., Kar, P., O'Keefe, J., Curley, M., Weaver, A., Barron, E., Bakhai, C., Khunti, K., Wareham, N. J., Sattar, N., Young, B., & Valabhji, J. (2020). Risk factors for COVID-19-related mortality in people with type 1 and type 2 diabetes in England : A population-based cohort study. The Lancet. Diabetes & Endocrinology, 8(10). https://doi.org/10.1016/S2213-8587(20)30271-0

Holmberg, V., Salmi, H., Kattainen, S., Ollgren, J., Kantele, A., Pynnönen, J., Järvinen, A., Forsblom, E., Silén, S., Kivivuori, S.-M., Meretoja, A., & Hästbacka, J. (2022). Association between first language and SARS-CoV-2 infection rates, hospitalization, intensive care admissions and death in Finland : A population-based observational cohort study. Clinical Microbiology and Infection: The Official Publication of the European Society of Clinical Microbiology and Infectious Diseases, 28(1). https://doi.org/10.1016/j.cmi.2021.08.022

Holstiege, J., Akmatov, M. K., Kohring, C., Dammertz, L., Ng, F., Czihal, T., von Stillfried, D., & Bätzing, J. (2021). Patients at high risk for a severe clinical course of COVID-19—Small-area data in support of vaccination and other population-based interventions in Germany. BMC Public Health, 21(1). https://doi.org/10.1186/s12889-021-11735-3

Holzmann-Littig, C., Braunisch, M. C., Kranke, P., Popp, M., Seeber, C., Fichtner, F., Littig, B., Carbajo-Lozoya, J., Allwang, C., Frank, T., Meerpohl, J. J., Haller, B., & Schmaderer, C. (2021). COVID-19 Vaccination Acceptance and Hesitancy among Healthcare Workers in Germany. Vaccines, 9(7). https://doi.org/10.3390/vaccines9070777

Homan, T., Mazzilli, S., Chieti, A., Musa, A., Roth, A., Fortunato, F., Bisceglia, L., Prato, R., Lopalco, P. L., & Martinelli, D. (2022). Covid-19 vaccination programme effectiveness against SARS-CoV-2 related infections, hospital admissions and deaths in the Apulia region of Italy : A one-year retrospective cohort study. Scientific Reports, 12(1). https://doi.org/10.1038/s41598-022-23235-4

Hönemann, M., Maier, M., Frille, A., Thiem, S., Bergs, S., Williams, T. C., Mas, V., Lübbert, C., & Pietsch, C. (2024). Respiratory Syncytial Virus in Adult Patients at a Tertiary Care Hospital in Germany : Clinical Features and Molecular Epidemiology of the Fusion Protein in the Severe Respiratory Season of 2022/2023. Viruses, 16(6). https://doi.org/10.3390/v16060943

Hönemann, M., Thiem, S., Bergs, S., Berthold, T., Propach, C., Siekmeyer, M., Frille, A., Wallborn, T., Maier, M., & Pietsch, C. (2023). In-Depth Analysis of the Re-Emergence of Respiratory Syncytial Virus at a Tertiary Care Hospital in Germany in the Summer of 2021 after the Alleviation of Non-Pharmaceutical Interventions Due to the SARS-CoV-2 Pandemic. Viruses, 15(4), 877. https://doi.org/10.3390/v15040877

Hopcroft, L. E., Curtis, H. J., Brown, A. D., Hulme, W. J., Andrews, C. D., Morton, C. E., Inglesby, P., Morley, J., Mehrkar, A., Bacon, S. C., Eggo, R. M., Mahalingasivam, V., Parker, E. P. K., Tomlinson, L. A., Bates, C., Cockburn, J., Parry, J., Hester, F., Harper, S., … MacKenna, B. (2023). First dose COVID-19 vaccine coverage amongst adolescents and children in England : An analysis of 3.21 million patients' primary care records in situ using OpenSAFELY. Wellcome Open Research, 8. https://doi.org/10.12688/wellcomeopenres.18735.2

Horne, E. M. F., Hulme, W. J., Parker, E. P. K., Keogh, R. H., Williamson, E. J., Walker, V. M., Palmer, T. M., Denholm, R., Knight, R., Curtis, H. J., Walker, A. J., Andrews, C. D., Mehrkar, A., Morley, J., MacKenna, B., Bacon, S. C. J., Goldacre, B., Hernán, M. A., Sterne, J. A. C., & and the OpenSAFELY collaborative. (2024). Effectiveness of mRNA COVID-19 Vaccines as First Booster Doses in England : An Observational Study in OpenSAFELY-TPP. Epidemiology (Cambridge, Mass.), 35(4). https://doi.org/10.1097/EDE.0000000000001747

Houhamdi, L., Gautret, P., Hoang, V. T., Fournier, P.-E., Colson, P., & Raoult, D. (2022). Characteristics of the first 1119 SARS-CoV-2 Omicron variant cases, in Marseille, France, November-December 2021. Journal of Medical Virology, 94(5). https://doi.org/10.1002/jmv.27613

Hourani, A., Abdelsalman, A., & Sürmeli, A. D. (2025). The Long-Term Impact of COVID-19 Non-Pharmaceutical Interventions on Notifiable Infectious Diseases in Poland: A Comprehensive Analysis from 2014-2022. medRxiv. https://doi.org/10.1101/2025.03.05.25323398

Houwaart, T., Belhaj, S., Tawalbeh, E., Nagels, D., Fröhlich, Y., Finzer, P., Ciruela, P., Sabrià, A., Herrero, M., Andrés, C., Antón, A., Benmoumene, A., Asskali, D., Haidar, H., von Dahlen, J., Nicolai, J., Stiller, M., Blum, J., Lange, C., … German COVID-19 OMICs Initiative (DeCOI). (2022). Integrated genomic surveillance enables tracing of person-to-person SARS-CoV-2 transmission chains during community transmission and reveals extensive onward transmission of travel-imported infections, Germany, June to July 2021. Euro Surveillance: Bulletin Europeen Sur Les Maladies Transmissibles = European Communicable Disease Bulletin, 27(43). https://doi.org/10.2807/1560-7917.ES.2022.27.43.2101089

Howkins, J., Packer, S., Walsh, E., Kumar, D., Edeghere, O., Hickman, M., & Oliver, I. (2024). Risk of transmission of SARS-CoV-2 on international flights, a retrospective cohort study using national surveillance data in England. BMC Infectious Diseases, 24(1). https://doi.org/10.1186/s12879-024-09052-2

Huang, G., & Brown, P. E. (2021). Population-weighted exposure to air pollution and COVID-19 incidence in Germany. Spatial Statistics, 41. https://doi.org/10.1016/j.spasta.2020.100480

Huang, J., & Qi, G. (2020). Effects of control measures on the dynamics of COVID-19 and double-peak behavior in Spain. Nonlinear Dynamics, 101(3). https://doi.org/10.1007/s11071-020-05901-2

Huber, M., & Langen, H. (2020). Timing matters : The impact of response measures on COVID-19-related hospitalization and death rates in Germany and Switzerland. Swiss Journal of Economics and Statistics, 156(1). https://doi.org/10.1186/s41937-020-00054-w

Hubin, P., van den Borre, L., Braeye, T., Cavillot, L., Billuart, M., Stouten, V., Nasiadka, L., Vermeiren, E., Evercooren, I., Devleesschauwer, B., Catteau, L., & van Loenhout, J. (2025). Disparities in COVID-19

vaccination in Belgium: The impact of demographic and socio-economic factors at area and individual levels. ARCHIVES OF PUBLIC HEALTH, 83.

Hubin, P., Van den Borre, L., Braeye, T., Cavillot, L., Billuart, M., Stouten, V., Nasiadka, L., Vermeiren, E., Van Evercooren, I., Devleesschauwer, B., Catteau, L., & van Loenhout, J. A. F. (2024). Area and individual level analyses of demographic and socio-economic disparities in COVID-19 vaccination uptake in Belgium. Vaccine: X, 18. https://doi.org/10.1016/j.jvacx.2024.100496

Huebbe, B., Mocek, A., Manz, K. C., Vivirito, A., Bayer, L. J., Norris, R., Schiffner-Rohe, J., von Eiff, C., & Lade, C. (2024). Economic burden of respiratory syncytial virus in adults in Germany—A health claims analysis between 2015 and 2018. Journal of Medical Economics, 27(1). https://doi.org/10.1080/13696998.2024.2389676

Hughes, D., Cheyne, C., Ashton, M., Coffey, E., Crozier, A., Semple, M., Buchan, I., & García-Fiñana, M. (2023). Association of SARS-CoV-2 viral load distributions with individual demographics and suspected variant type : Results from the Liverpool community testing pilot, England, 6 November 2020 to 8 September 2021. EUROSURVEILLANCE, 28(4). https://doi.org/10.2807/1560-7917.ES.2023.28.4.2200129

Huiberts, A. J., de Gier, B., Hoeve, C. E., de Melker, H. E., Hahné, S. J. M., den Hartog, G., Grobbee, D. E., van de Wijgert, J. H. H. M., van den Hof, S., & Knol, M. J. (2023). Vaccine effectiveness of primary and booster COVID-19 vaccinations against SARS-CoV-2 infection in the Netherlands from July 12, 2021 to June 6, 2022 : A prospective cohort study. International Journal of Infectious Diseases: IJID: Official Publication of the International Society for Infectious Diseases, 133. https://doi.org/10.1016/j.ijid.2023.04.401

Huiberts, A. J., de Gier, B., Hoeve, C. E., de Melker, H. E., Hahné, S. J., den Hartog, G., van de Wijgert, J. H., van den Hof, S., & Knol, M. J. (2023a). Effectiveness of bivalent mRNA booster vaccination against SARS-CoV-2 Omicron infection, the Netherlands, September to December 2022. Euro Surveillance: Bulletin Europeen Sur Les Maladies Transmissibles = European Communicable Disease Bulletin, 28(7). https://doi.org/10.2807/1560-7917.ES.2023.28.7.2300087

Huiberts, A. J., Hoeve, C. E., de Gier, B., Cremer, J., van der Veer, B., de Melker, H. E., van de Wijgert, J. H., van den Hof, S., Eggink, D., & Knol, M. J. (2024b). Effectiveness of Omicron XBB.1.5 vaccine against infection with SARS-CoV-2 Omicron XBB and JN.1 variants, prospective cohort study, the Netherlands, October 2023 to January 2024. Euro Surveillance: Bulletin Europeen Sur Les Maladies Transmissibles = European Communicable Disease Bulletin, 29(10). https://doi.org/10.2807/1560-7917.ES.2024.29.10.2400109

Huiberts, A. J., Hoeve, C. E., Kooijman, M. N., de Melker, H. E., Hahné, S. J., Grobbee, D. E., van Binnendijk, R., den Hartog, G., van de Wijgert, J. H., van den Hof, S., & Knol, M. J. (2024). Cohort profile : An observational population-based cohort study on COVID-19 vaccine effectiveness in the Netherlands—The VAccine Study COVID-19 (VASCO). BMJ Open, 14(10). https://doi.org/10.1136/bmjopen-2024-085388

Hulme, W. J., Horne, E. M. F., Parker, E. P. K., Keogh, R. H., Williamson, E. J., Walker, V., Palmer, T. M., Curtis, H. J., Walker, A. J., Andrews, C. D., Mehrkar, A., Morley, J., MacKenna, B., Bacon, S. C. J., Goldacre, B., Hernán, M. A., & Sterne, J. A. C. (2023). Comparative effectiveness of BNT162b2 versus mRNA-1273 covid-19 vaccine boosting in England : Matched cohort study in OpenSAFELY-TPP. BMJ (Clinical Research Ed.), 380. https://doi.org/10.1136/bmj-2022-072808

Hulme, W. J., Williamson, E. J., Green, A. C. A., Bhaskaran, K., McDonald, H. I., Rentsch, C. T., Schultze, A., Tazare, J., Curtis, H. J., Walker, A. J., Tomlinson, L. A., Palmer, T., Horne, E. M. F., MacKenna, B., Morton, C. E., Mehrkar, A., Morley, J., Fisher, L., Bacon, S. C. J., … Goldacre, B. (2022). Comparative effectiveness of ChAdOx1 versus BNT162b2 covid-19 vaccines in health and social care workers in England : Cohort study using OpenSAFELY. BMJ (Clinical Research Ed.), 378. https://doi.org/10.1136/bmj-2021-068946

Hulth, A., & Rydevik, G. (2011). Web query-based surveillance in Sweden during the influenza A(H1N1)2009 pandemic, April 2009 to February 2010. Euro Surveillance: Bulletin Europeen Sur Les Maladies Transmissibles = European Communicable Disease Bulletin, 16(18), 19856.

Huy, C., Kuhn, D., Schneider, S., & Zöllner, I. (2012). Seasonal waves of influenza and cause-specific mortality in Germany. CENTRAL EUROPEAN JOURNAL OF MEDICINE, 7(4). https://doi.org/10.2478/s11536-012-0017-9

Huyghe, E., Abrams, S., André, E., Anseeuw, K., Bernaert, E., Bruynseels, P., Cuypers, L., De Schouwer, P., Hilkens, P., Keyaerts, E., Laenen, L., Maes, J., Magerman, K., Van de Gaer, O., Verdonck, A., Verstrepen, W., Ombelet, S., & Naesens, R. (2025). Systematic Molecular Influenza A/B Screening Upon Hospital Admission in Belgium, January-April 2022 : Positivity Ratios and Viral Loads According to Symptomatology, Age, and Vaccination Status. Journal of Medical Virology, 97(1). https://doi.org/10.1002/jmv.70167

Hyafil, A., & Moriña, D. (2021). Analysis of the impact of lockdown on the reproduction number of the SARS-Cov-2 in Spain. Gaceta Sanitaria, 35(5). https://doi.org/10.1016/j.gaceta.2020.05.003

Iacoella, C., Ralli, M., Maggiolini, A., Arcangeli, A., & Ercoli, L. (2021). Acceptance of COVID-19 vaccine among persons experiencing homelessness in the City of Rome, Italy. European Review for Medical and Pharmacological Sciences, 25(7). https://doi.org/10.26355/eurrev_202104_25568

Iannitti, T., Capone, S., & Palmieri, B. (2011). A telephone interview to assess alkylglycerols' effectiveness in preventing influenza-like symptoms in Modena, Emilia Romagna, Italy, in the season 2009-2010. La Clinica Terapeutica, 162(4). http://www.ncbi.nlm.nih.gov/pubmed/21912812

Iftimie, S., López-Azcona, A. F., Lozano-Olmo, M. J., Naval-Ferrando, À., Domingo-Cortés, V., Castañé, H., Jiménez-Franco, A., Hernández-Aguilera, A., Guilarte, C., Riu, F., Camps, J., Joven, J., & Castro, A. (2023). Retrospective Analysis of Vaccination Status and Predominant Viral Variants in Patients Hospitalized with COVID-19 in Reus, Spain. Viruses, 15(4). https://doi.org/10.3390/v15040886

Iftimie, S., López-Azcona, A. F., Vicente-Miralles, M., Descarrega-Reina, R., Hernández-Aguilera, A., Riu, F., Simó, J. M., Garrido, P., Joven, J., Camps, J., & Castro, A. (2020). Risk factors associated with mortality in hospitalized patients with SARS-CoV-2 infection. A prospective, longitudinal, unicenter study in Reus, Spain. PloS One, 15(9). https://doi.org/10.1371/journal.pone.0234452

Ilardi, A., Chieffi, S., Iavarone, A., & Ilardi, C. R. (2021). SARS-CoV-2 in Italy : Population Density Correlates with Morbidity and Mortality. Japanese Journal of Infectious Diseases, 74(1). https://doi.org/10.7883/yoken.JJID.2020.200

Inciardi, R. M., Adamo, M., Lupi, L., Cani, D. S., Di Pasquale, M., Tomasoni, D., Italia, L., Zaccone, G., Tedino, C., Fabbricatore, D., Curnis, A., Faggiano, P., Gorga, E., Lombardi, C. M., Milesi, G., Vizzardi, E., Volpini, M., Nodari, S., Specchia, C., … Metra, M. (2020). Characteristics and outcomes of patients hospitalized for COVID-19 and cardiac disease in Northern Italy. European Heart Journal, 41(19). https://doi.org/10.1093/eurheartj/ehaa388

Influenza A(H1N1)v investigation teams, Levy-Bruhl, D., & Vaux, S. (2009). Modified surveillance of influenza A(H1N1)v virus infections in France. Euro Surveillance: Bulletin Europeen Sur Les Maladies Transmissibles = European Communicable Disease Bulletin, 14(29). https://doi.org/10.2807/ese.14.29.19276-en

Inglis, N. J., Bagnall, H., Janmohamed, K., Suleman, S., Awofisayo, A., De Souza, V., Smit, E., Pebody, R., Mohamed, H., Ibbotson, S., Smith, G. E., House, T., & Olowokure, B. (2014). Measuring the effect of influenza A(H1N1)pdm09 : The epidemiological experience in the West Midlands, England during the « containment » phase. Epidemiology and Infection, 142(2). https://doi.org/10.1017/S0950268813001234

Iosa, M., Paolucci, S., & Morone, G. (2020). Covid-19 : A Dynamic Analysis of Fatality Risk in Italy. Frontiers in Medicine, 7. https://doi.org/10.3389/fmed.2020.00185

Isitt, C., Sjöholm, D., Hergens, M.-P., Granath, F., & Nauclér, P. (2022). The early impact of vaccination against SARS-CoV-2 in Region Stockholm, Sweden. Vaccine, 40(20). https://doi.org/10.1016/j.vaccine.2022.03.061

Ismail, M., Warsame, A., & Wilhelmsson, M. (2022). An Exploratory Analysis of Housing and the Distribution of COVID-19 in Sweden. BUILDINGS, 12(1). https://doi.org/10.3390/buildings12010071

Isphording, I., Lipfert, M., & Pestel, N. (2021). Does re-opening schools contribute to the spread of SARS-CoV-2 ? Evidence from staggered summer breaks in Germany. JOURNAL OF PUBLIC ECONOMICS, 198. https://doi.org/10.1016/j.jpubeco.2021.104426

Izquierdo-Lara, R., Elsinga, G., Heijnen, L., Munnink, B. B. O., Schapendonk, C. M. E., Nieuwenhuijse, D., Kon, M., Lu, L., Aarestrup, F. M., Lycett, S., Medema, G., Koopmans, M. P. G., & de Graaf, M. (2021).

Monitoring SARS-CoV-2 Circulation and Diversity through Community Wastewater Sequencing, the Netherlands and Belgium. Emerging Infectious Diseases, 27(5). https://doi.org/10.3201/eid2705.204410

Jäckle, S., & Timmis, J. K. (2024). Esoteric beliefs and CAM impact SARS-CoV-2 immunization drivers, uptake and pediatric immunization views in Germany. NPJ Vaccines, 9(1). https://doi.org/10.1038/s41541-024-00928-7

Jacks, A., Ollgren, J., Ziegler, T., & Lyytikäinen, O. (2012). Influenza-associated hospitalisations in Finland from 1996 to 2010: Unexpected age-specific burden during the influenza A(H1N1)pdm09 pandemic from 2009 to 2010. EUROSURVEILLANCE, 17(38), 7–14.

Jacob, J., Biering-Sørensen, T., Holger Ehlers, L., Edwards, C. H., Mohn, K. G.-I., Nilsson, A., Hjelmgren, J., Ma, W., Sharma, Y., Ciglia, E., & Mould-Quevedo, J. (2023). Cost-Effectiveness of Vaccination of Older Adults with an MF59®-Adjuvanted Quadrivalent Influenza Vaccine Compared to Standard-Dose and High-Dose Vaccines in Denmark, Norway, and Sweden. Vaccines, 11(4). https://doi.org/10.3390/vaccines11040753

Jacob, L., Koyanagi, A., Smith, L., Haro, J. M., Rohe, A. M., & Kostev, K. (2021). Prevalence of and factors associated with COVID-19 diagnosis in symptomatic patients followed in general practices in Germany between March 2020 and March 2021. International Journal of Infectious Diseases: IJID: Official Publication of the International Society for Infectious Diseases, 111. https://doi.org/10.1016/j.ijid.2021.08.010

Jacob, L., Koyanagi, A., Smith, L., Tanislav, C., Konrad, M., van der Beck, S., & Kostev, K. (2021). Prevalence of, and factors associated with, long-term COVID-19 sick leave in working-age patients followed in general practices in Germany. International Journal of Infectious Diseases: IJID: Official Publication of the International Society for Infectious Diseases, 109. https://doi.org/10.1016/j.ijid.2021.06.063

Jaffer Broman, N., Nilsson, A. C., Lengquist, M., Frigyesi, A., Friberg, H., & Reepalu, A. (2025). High one-year mortality following intensive care among adults with influenza A(H1N1)pdm09, A(H3N2), or B in Southern Sweden: A retrospective observational study. Infectious Diseases (London, England), 1–12. https://doi.org/10.1080/23744235.2025.2535443

Jagielska, A. M., Jasik, M., & Nitsch-Osuch, A. (2021). Determinants and coverage of seasonal influenza vaccination among women of childbearing age in Poland. Ginekologia Polska, 92(1). https://doi.org/10.5603/GP.a2020.0138

Jain, V., Kerr, G., & Beaney, T. (2024). The impact of the 2022 spring COVID-19 booster vaccination programme on hospital occupancy in England : An interrupted time series analysis. PLOS Global Public Health, 4(3). https://doi.org/10.1371/journal.pgph.0002046

Janik, K., Nietupska, K., Iwanowicz-Palus, G., & Cybulski, M. (2022). Fear of COVID-19 and Vaccine Hesitancy among Pregnant Women in Poland : A Cross-Sectional Study. Vaccines, 10(10). https://doi.org/10.3390/vaccines10101700

Jank, M., Oechsle, A.-L., Armann, J., Behrends, U., Berner, R., Chao, C.-M., Diffloth, N., Doenhardt, M., Hansen, G., Hufnagel, M., Lander, F., Liese, J. G., Muntau, A. C., Niehues, T., von Both, U., Verjans, E., Weil, K., von Kries, R., & Schroten, H. (2023). Comparing SARS-CoV-2 variants among children and adolescents in Germany : Relative risk of COVID-19-related hospitalization, ICU admission and mortality. Infection, 51(5). https://doi.org/10.1007/s15010-023-01996-y

Janssen, C., Maillard, A., Bodelet, C., Claudel, A.-L., Gaillat, J., Delory, T., & On Behalf Of The Acv Alpin Study Group, null. (2021). Hesitancy towards COVID-19 Vaccination among Healthcare Workers : A Multi-Centric Survey in France. Vaccines, 9(6). https://doi.org/10.3390/vaccines9060547

Jarva, H., Lappalainen, M., Luomala, O., Jokela, P., Jääskeläinen, A. E., Jääskeläinen, A. J., Kallio-Kokko, H., Kekäläinen, E., Mannonen, L., Soini, H., Suuronen, S., Toivonen, A., Savolainen-Kopra, C., Loginov, R., & Kurkela, S. (2021). Laboratory-based surveillance of COVID-19 in the Greater Helsinki area, Finland, February-June 2020. International Journal of Infectious Diseases: IJID: Official Publication of the International Society for Infectious Diseases, 104. https://doi.org/10.1016/j.ijid.2020.12.038

Järvinen, A., Joutseno, J., & Gyldmark, M. (2007). Cost effectiveness of oseltamivir for the treatment of influenza in adults, adolescents and children in Finland. Journal of Medical Economics, 10(3). Embase. https://doi.org/10.3111/13696990701427747

Jaskolowska, J., Balcerzyk-Barzdo, E., Jozwik, A., Gaszynski, T., & Ratajczyk, P. (2023). Selected Predictors of COVID-19 Mortality in the Hospitalised Patient Population in a Single-Centre Study in Poland. Healthcare (Basel, Switzerland), 11(5). https://doi.org/10.3390/healthcare11050719

Jaya, I. G. N. M., Folmer, H., & Lundberg, J. (2022). A joint Bayesian spatiotemporal risk prediction model of COVID-19 incidence, IC admission, and death with application to Sweden. The Annals of Regional Science. https://doi.org/10.1007/s00168-022-01191-1

Jędrzejek, M. J., & Mastalerz-Migas, A. (2022). Influenza Vaccination Coverage, Motivators for, and Barriers to Influenza Vaccination among Healthcare Workers in Wroclaw, Poland. International Journal of Environmental Research and Public Health, 19(3). https://doi.org/10.3390/ijerph19031586

Jeffery, C., Cheyne, C. P., Buchan, I., Garcia-Finana, M., Green, M. A., Bonnett, L., Hughes, D. M., French, N., & Hungerford, D. (2025). Effect of Influenza Vaccination on Post-Admission Outcomes for Influenza Patients in England: A Population-Based Cohort Study. SSRN. https://doi.org/10.2139/ssrn.5339382

Jepsen, M. T., Trebbien, R., Emborg, H. D., Krause, T. G., Schønning, K., Voldstedlund, M., Nielsen, J., & Fischer, T. K. (2018). Incidence and seasonality of respiratory syncytial virus hospitalisations in young children in Denmark, 2010 to 2015. Euro Surveillance: Bulletin Europeen Sur Les Maladies Transmissibles = European Communicable Disease Bulletin, 23(3). https://doi.org/10.2807/1560-7917.ES.2018.23.3.17-00163

Jesús Pérez Martín, J., de la Cruz Gómez Moreno, M., Sánchez Manresa, S., del Pilar Ros Abellán, M., & Zornoza-Moreno, M. (2025). Respiratory syncytial virus immunization with nirsevimab: Acceptance and satisfaction assessment in infants and risk groups in the region of Murcia (Spain). Human Vaccines and Immunotherapeutics, 21(1). https://doi.org/10.1080/21645515.2025.2471700

Jimenez, A. J., Estevez-Reboredo, R. M., Santed, M. A., & Ramos, V. (2020). COVID-19 Symptom-Related Google Searches and Local COVID-19 Incidence in Spain : Correlational Study. Journal of Medical Internet Research, 22(12). https://doi.org/10.2196/23518

Jiménez, E., Fontán-Vela, M., Valencia, J., Fernandez-Jimenez, I., Álvaro-Alonso, E. A., Izquierdo-García, E., Lazaro Cebas, A., Gallego Ruiz-Elvira, E., Troya, J., Tebar-Martinez, A. J., Garcia-Marina, B., Peña-Lillo, G., Abad-Motos, A., Macaya, L., Ryan, P., Pérez-Butragueño, M., COVID@HUIL Working Group, & COVID@HUIL Working Group. (2020). Characteristics, complications and outcomes among 1549 patients hospitalised with COVID-19 in a secondary hospital in Madrid, Spain : A retrospective case series study. BMJ Open, 10(11). https://doi.org/10.1136/bmjopen-2020-042398

Jiménez-García, R., Esteban-Vasallo, M. D., Rodríguez-Rieiro, C., Hernandez-Barrera, V., Domínguez-Berjón, M. A. F., Carrasco Garrido, P., Lopez de Andres, A., Cameno Heras, M., Iniesta Fornies, D., & Astray-Mochales, J. (2014). Coverage and predictors of vaccination against 2012/13 seasonal influenza in Madrid, Spain : Analysis of population-based computerized immunization registries and clinical records. Human Vaccines & Immunotherapeutics, 10(2). https://doi.org/10.4161/hv.27152

Jiménez-García, R., Hernández-Barrera, V., Carrasco-Garrido, P., de Andres, A. L., Esteban y Peña, M. M., & de Miguel, A. G. (2008). Coverage and predictors of influenza vaccination among adults living in a large metropolitan area in Spain : A comparison between the immigrant and indigenous populations. Vaccine, 26(33). https://doi.org/10.1016/j.vaccine.2008.05.053

Jiménez-García, R., Hernández-Barrera, V., Carrasco-Garrido, P., de Andrés, A., Pérez, N., & de Miguel, A. (2008). Influenza vaccination coverages among children, adults, health care workers and immigrants in Spain : Related factors and trends, 2003-2006. JOURNAL OF INFECTION, 57(6). https://doi.org/10.1016/j.jinf.2008.10.005

Jiménez-García, R., Hernández-Barrera, V., de Andres, A. L., Jimenez-Trujillo, I., Esteban-Hernández, J., & Carrasco-Garrido, P. (2010). Gender influence in influenza vaccine uptake in Spain : Time trends analysis (1995-2006). Vaccine, 28(38). https://doi.org/10.1016/j.vaccine.2010.07.029

Jiménez-García, R., Hernández-Barrera, V., Rodríguez-Rieiro, C., Lopez de Andres, A., De Miguel-Diez, J., Jimenez-Trujillo, I., Gil de Miguel, A., & Carrasco-Garrido, P. (2013). Hospitalizations from pandemic Influenza [A(H1N1)pdm09] infections among type 1 and 2 diabetes patients in Spain. Influenza and Other Respiratory Viruses, 7(3), 439–447. https://doi.org/10.1111/j.1750-2659.2012.00419.x

Jiménez-García, R., Jimenez, I., Garrido, P. C., Hernández-Barrera, V., de Andres, A. L., del Barrio, J. L., & de Miguel, A. G. (2008). Coverage and predictors of influenza vaccination among adults with diabetes in Spain. Diabetes Research and Clinical Practice, 79(3). https://doi.org/10.1016/j.diabres.2007.10.013

Jiménez-García, R., Mayo-Montero, E., Hernández-Barrera, V., Sierra-Moros, M. J., Pachón del Amo, I., Carrasco-Garrido, P., Martinez-Hernandez, D., & de Miguel, A. G. (2005). Evolution of anti-influenza vaccination coverage in Spain from 1993 to 2001. Vaccine, 23(22). https://doi.org/10.1016/j.vaccine.2004.11.055

Jiménez-García, R., Rodríguez-Rieiro, C., Hernandez-Barrera, V., Carrasco Garrido, P., López de Andres, A., Esteban-Vasallo, M. D., Domínguez-Berjón, M. F., & Astray-Mochales, J. (2014). Negative trends from 2008/9 to 2011/12 seasons in influenza vaccination coverages among high risk subjects and health care workers in Spain. Vaccine, 32(3). https://doi.org/10.1016/j.vaccine.2013.11.040

Jiménez-García, R., Rodríguez-Rieiro, C., Hernández-Barrera, V., Lopez de Andres, A., Rivero Cuadrado, A., Rodriguez Laso, A., & Carrasco-Garrido, P. (2011). Effectiveness of age-based strategies to increase influenza vaccination coverage among high risk subjects in Madrid (Spain). Vaccine, 29(16). https://doi.org/10.1016/j.vaccine.2011.02.004

Jiménez-Jorge, S., de Mateo, S., Delgado-Sanz, C., Pozo, F., Casas, I., Garcia-Cenoz, M., Castilla, J., Pérez, E., Gallardo, V., Rodriguez, C., Vega, T., Quiñones, C., Martínez, E., Vanrell, J. M., Giménez, J., Castrillejo, D., Serrano, M. del C., Ramos, J. M., Larrauri, A., & Spanish Influenza Sentinel Surveillance System. (2013). Effectiveness of influenza vaccine against laboratory-confirmed influenza, in the late 2011-2012 season in Spain, among population targeted for vaccination. BMC Infectious Diseases, 13. https://doi.org/10.1186/1471-2334-13-441

Jimenez-Jorge, S., de Mateo, S., Delgado-Sanz, C., Pozo, F., Casas, I., Garcia-Cenoz, M., Castilla, J., Rodriguez, C., Vega, T., Quinones, C., Martinez, E., Vanrell, J. M., Gimenez, J., Castrillejo, D., Altzibar, J. M., Carril, F., Ramos, J. M., Serrano, M. C., Martinez, A., … Spanish Influenza Sentinel Surveillance System. (2015). Estimating influenza vaccine effectiveness in Spain using sentinel surveillance data. Euro Surveillance: Bulletin Europeen Sur Les Maladies Transmissibles = European Communicable Disease Bulletin, 20(28). https://doi.org/10.2807/1560-7917.es2015.20.28.21187

Jimenez-Jorge, S., de Mateo, S., Pozo, F., Casas, I., Garcia Cenoz, M., Castilla, J., Gallardo, V., Perez, E., Vega, T., Rodriguez, C., Quinones, C., Martinez, E., Gimenez, J., Vanrell, J., Castrillejo, D., Serrano, M., Ramos, J., & Larrauri, A. (2012). Early estimates of the effectiveness of the 2011/12 influenza vaccine in the population targeted for vaccination in Spain, 25 December 2011 to 19 February 2012. Euro Surveillance: Bulletin Europeen Sur Les Maladies Transmissibles = European Communicable Disease Bulletin, 17(12). http://www.ncbi.nlm.nih.gov/pubmed/22490308

Jiménez-Jorge, S., Mateo Ontañón, S. de, Savulescu, C., Delgado-Sanz, C., Pozo Sánchez, F., García-Cenoz, M., Castilla Catalán, J., Rodríguez Gay, C., Vega Alonso, T., Quiñones Rubio, C., Martínez Ochoa, E., Vanrell Berga, J. M., Giménez Durán, J., Castrillejo Pérez, D., Altzíbar Arotzena, J. M., González Carril, F., Ramos Aceitero, J. M., Serrano Martin, M. del C., Martínez i Mateo, A., … Larrauri Cámara, A. (2014). [cycEVA study : Case control study measuring influenza vaccine effectiveness in Spain, 2008-2013]. Revista Espanola De Salud Publica, 88(5). https://doi.org/10.4321/S1135-57272014000500005

Jimenez-Jorge, S., Pozo, F., de Mateo, S., Delgado-Sanz, C., Casas, I., Garcia-Cenoz, M., Castilla, J., Sancho, R., Etxebarriarteun-Aranzabal, L., Quinones, C., Martinez, E., Vega, T., Garcia, A., Gimenez, J., Vanrell, J. M., Castrillejo, D., Larrauri, A., & Spanish Influenza Sentinel Surveillance System (SISS). (2014). Influenza vaccine effectiveness in Spain 2013/14 : Subtype-specific early estimates using the cycEVA study. Euro Surveillance: Bulletin Europeen Sur Les Maladies Transmissibles = European Communicable Disease Bulletin, 19(9). https://doi.org/10.2807/1560-7917.es2014.19.9.20727

Jiménez-Jorge, S., Pozo, F., Larrauri, A., & cycEVA Study Team. (2015). Interim influenza vaccine effectiveness : A good proxy for final estimates in Spain in the seasons 2010-2014. Vaccine, 33(29). https://doi.org/10.1016/j.vaccine.2015.03.051

Jiménez-Jorge, S., Savulescu, C., Pozo, F., de Mateo, S., Casas, I., Ledesma, J., Larrauri, A., cycEVA Study Team, & Spanish Influenza Sentinel Surveillance System. (2012). Effectiveness of the 2010-11 seasonal trivalent influenza vaccine in Spain : cycEVA study. Vaccine, 30(24). https://doi.org/10.1016/j.vaccine.2012.03.048

Jiménez-Rodríguez, P., Muñoz-Fernández, G. A., Rodrigo-Chocano, J. C., Seoane-Sepúlveda, J. B., & Weber, A. (2022). A population structure-sensitive mathematical model assessing the effects of vaccination during the third surge of COVID-19 in Italy. Journal of Mathematical Analysis and Applications, 514(2). https://doi.org/10.1016/j.jmaa.2021.125975

Jiménez-Sepúlveda, N., Gras-Valentí, P., Chico-Sánchez, P., Castro-García, J. M., Ronda-Pérez, E., Vanaclocha, H., Peiró, S., Burgos, J. S., Ana Berenguer, null, Navarro, D., Sánchez-Payá, J., & Valencian vaccine research program ProVaVac study group. (2024). Effectiveness of mRNA booster doses in preventing infections and hospitalizations due to SARS-CoV-2 and its dominant variant over time in Valencian healthcare workers, Spain. Vaccine, 42(19). https://doi.org/10.1016/j.vaccine.2024.05.011

Jimenez-Trujillo, I., López-de Andrés, A., Hernández-Barrera, V., Carrasco-Garrido, P., Santos-Sancho, J. M., & Jiménez-García, R. (2013). Influenza vaccination coverage rates among diabetes sufferers, predictors of adherence and time trends from 2003 to 2010 in Spain. Human Vaccines & Immunotherapeutics, 9(6). https://doi.org/10.4161/hv.23926

Jimeno Ruiz, S., Peláez, A., Calle Gómez, Á., Villarreal García-Lomas, M., & Martínez, S. N. (2024). Impact of Respiratory Syncytial Virus (RSV) in Adults 60 Years and Older in Spain. Geriatrics (Basel, Switzerland), 9(6). https://doi.org/10.3390/geriatrics9060145

Jit, M., Cromer, D., Baguelin, M., Stowe, J., Andrews, N., & Miller, E. (2010). The cost-effectiveness of vaccinating pregnant women against seasonal influenza in England and Wales. Vaccine, 29(1). https://doi.org/10.1016/j.vaccine.2010.08.078

Jones, R. P., & Ponomarenko, A. (2023). COVID-19-Related Age Profiles for SARS-CoV-2 Variants in England and Wales and States of the USA (2020 to 2022) : Impact on All-Cause Mortality. Infectious Disease Reports, 15(5). https://doi.org/10.3390/idr15050058

Jones, S., Mason, N., Palser, T., Swift, S., Petrilli, C. M., & Horwitz, L. I. (2021). Trends in Risk-Adjusted 28-Day Mortality Rates for Patients Hospitalized with COVID-19 in England. Journal of Hospital Medicine, 16(5). https://doi.org/10.12788/jhm.3599

Jones, T., Adamali, H., Redaniel, M. T., de Vocht, F., Tilling, K., Kenward, C., Ben-Shlomo, Y., & Creavin, S. (2024). The impact of targeted local outreach clinics to improve COVID-19 vaccine uptake : Controlled interrupted time series in South West England. Archives of Public Health = Archives Belges De Sante Publique, 82(1). https://doi.org/10.1186/s13690-024-01341-1

Jonges, M., van der Lubben, I., Dijkstra, F., Verhoef, L., Koopmans, M., & Meijer, A. (2009). Dynamics of antiviral-resistant influenza viruses in the Netherlands, 2005-2008. ANTIVIRAL RESEARCH, 83(3). https://doi.org/10.1016/j.antiviral.2009.07.003

Joseph, C., Elgohari, S., Nichols, T., & Verlander, N. (2006). Influenza vaccine uptake in adults aged 50-64 years : Policy and practice in England 2003/2004. Vaccine, 24(11). https://doi.org/10.1016/j.vaccine.2005.10.024

Joseph, C., Goddard, N., & Gelb, D. (2005). Influenza vaccine uptake and distribution in England and Wales using data from the General Practice Research Database, 1989/90-2003/04. Journal of Public Health (Oxford, England), 27(4). https://doi.org/10.1093/pubmed/fdi054

Joshi, C., Ali, A., ÓConnor, T., Chen, L., & Jahanshahi, K. (2022). Understanding community level influences on the prevalence of SARS-CoV-2 infection in England. medRxiv, (Joshi C., chaitanya.joshi@ons.gov.uk; Ali A.; ÓConnor T.; Chen L., li.chen@ons.gov.uk; Jahanshahi K., kaveh.jahanshahi@ons.gov.uk) Office for National Statistics, Data Science Campus, United Kingdom. https://doi.org/10.1101/2022.04.14.22273759

Joshi, K., Scholz, S., Maschio, M., Kohli, M., Lee, A., Fust, K., Ultsch, B., Van de Velde, N., & Beck, E. (2023). Clinical impact and cost-effectiveness of the updated COVID-19 mRNA Autumn 2023 vaccines in Germany. medRxiv, (Joshi K., Keya.Joshi@modernatx.com; Van de Velde N.; Beck E.) Moderna Inc, 200 Technology Square, Cambridge, MA, United States. https://doi.org/10.1101/2023.10.09.23296505

Jourdes, A., Lafaurie, M., Martin-Blondel, G., Delobel, P., Faruch, M., Charpentier, S., Minville, V., Silva, S., Thalamas, C., Sommet, A., & Moulis, G. (2020). Clinical characteristics and outcome of hospitalized patients with SARS-CoV-2 infection at Toulouse University hospital (France). Results from the Covid-clinic-Toul cohort. La Revue De Medecine Interne, 41(11). https://doi.org/10.1016/j.revmed.2020.08.006

Joy, M., Hobbs, F. R., Bernal, J. L., Sherlock, J., Amirthalingam, G., McGagh, D., Akinyemi, O., Byford, R., Dabrera, G., Dorward, J., Ellis, J., Ferreira, F., Jones, N., Oke, J., Okusi, C., Nicholson, B. D., Ramsay, M., Sheppard, J. P., Sinnathamby, M., … de Lusignan, S. (2020). Excess mortality in the first COVID pandemic peak: Cross-sectional analyses of the impact of age, sex, ethnicity, household size, and long-term conditions in people of known SARS-CoV-2 status in England. The British Journal of General Practice: The Journal of the Royal College of General Practitioners, 70(701), e890–e898. https://doi.org/10.3399/bjgp20X713393

Juárez, S. P., Debiasi, E., Wallace, M., Drefahl, S., Mussino, E., Cederström, A., Rostila, M., & Aradhya, S. (2024). COVID-19 mortality among immigrants by duration of residence in Sweden : A population-based cohort study. Scandinavian Journal of Public Health, 52(3). https://doi.org/10.1177/14034948241244560

Juutinen, A., Sarvikivi, E., Laukkanen-Nevala, P., & Helve, O. (2021). Closing lower secondary schools had no impact on COVID-19 incidence in 13-15-year-olds in Finland. Epidemiology and Infection, 149. https://doi.org/10.1017/S0950268821002351

Juutinen, A., Sarvikivi, E., Laukkanen-Nevala, P., & Helve, O. (2023). Face mask recommendations in schools did not impact COVID-19 incidence among 10-12-year-olds in Finland—Joinpoint regression analysis. BMC Public Health, 23(1). https://doi.org/10.1186/s12889-023-15624-9

Kaddu-Mulindwa, D., Keuser, L., Lesan, V., Rissland, J., Smola, S., Werdecker, V., Stilgenbauer, S., Christofyllakis, K., Thurner, L., Bewarder, M., Lohr, B., Lutz, J., Lohse, S., & Rieke, A. (2022). IgG seroprevalence of COVID-19 among people living with HIV or at high risk of HIV in south-west Germany : A seroprevalence study. HIV Medicine, 23(5). https://doi.org/10.1111/hiv.13207

Kadiane-Oussou, N. J., Klopfenstein, T., Royer, P.-Y., Toko, L., Gendrin, V., & Zayet, S. (2020). COVID-19 : Comparative clinical features and outcome in 114 patients with or without pneumonia (Nord Franche-Comte Hospital, France). Microbes and Infection, 22(10). https://doi.org/10.1016/j.micinf.2020.10.002

Kaeuffer, C., Le Hyaric, C., Fabacher, T., Mootien, J., Dervieux, B., Ruch, Y., Hugerot, A., Zhu, Y.-J., Pointurier, V., Clere-Jehl, R., Greigert, V., Kassegne, L., Lefebvre, N., Gallais, F., Covid Alsace Study Group, Meyer, N., Hansmann, Y., Hinschberger, O., Danion, F., & COVID Alsace Study Group. (2020). Clinical characteristics and risk factors associated with severe COVID-19 : Prospective analysis of 1,045 hospitalised cases in North-Eastern France, March 2020. Euro Surveillance: Bulletin Europeen Sur Les Maladies Transmissibles = European Communicable Disease Bulletin, 25(48). https://doi.org/10.2807/1560-7917.ES.2020.25.48.2000895

Kafatos, G., Pebody, R., Andrews, N., Durnall, H., Barley, M., & Fleming, D. (2013). Effectiveness of seasonal influenza vaccine in preventing medically attended influenza infection in England and Wales during the 2010/2011 season : A primary care-based cohort study. Influenza and Other Respiratory Viruses, 7(6). https://doi.org/10.1111/irv.12163

Kahn, F., Bonander, C., Moghaddassi, M., Bennet, L., Malmqvist, U., Inghammar, M., & Björk, J. (2022). Protection against infection with the Omicron BA.5 subvariant among people with previous SARS-CoV-2 infection—Surveillance results from southern Sweden, June to August 2022. medRxiv, (Kahn F.; Inghammar M.) Department of Clinical Sciences Lund, Section for Infection Medicine, Lund University, Lund, Sweden. https://doi.org/10.1101/2022.11.08.22282069

Kahn, F., Bonander, C., Moghaddassi, M., Christiansen, C. B., Bennet, L., Malmqvist, U., Inghammar, M., & Björk, J. (2024). Previous SARS-CoV-2 infections and their impact on the protection from reinfection during the

Omicron BA.5 wave—A nested case-control study among vaccinated adults in Sweden. IJID Regions, 10. https://doi.org/10.1016/j.ijregi.2024.02.004

Kahn, F., Bonander, C., Moghaddassi, M., Rasmussen, M., Malmqvist, U., Inghammar, M., & Björk, J. (2022b). Risk of severe COVID-19 from the Delta and Omicron variants in relation to vaccination status, sex, age and comorbidities—Surveillance results from southern Sweden, July 2021 to January 2022. Euro Surveillance: Bulletin Europeen Sur Les Maladies Transmissibles = European Communicable Disease Bulletin, 27(9). https://doi.org/10.2807/1560-7917.ES.2022.27.9.2200121

Kałucka, S., & Grzegorczyk-Karolak, I. (2021). Barriers Associated with the Uptake Ratio of Seasonal Flu Vaccine and Ways to Improve Influenza Vaccination Coverage among Young Health Care Workers in Poland. Vaccines, 9(5). https://doi.org/10.3390/vaccines9050530

Kałucka, S., Kusideł, E., & Grzegorczyk-Karolak, I. (2022). A Retrospective Cross-Sectional Study on the Risk of Getting Sick with COVID-19, the Course of the Disease, and the Impact of the National Vaccination Program against SARS-CoV-2 on Vaccination among Health Professionals in Poland. International Journal of Environmental Research and Public Health, 19(12). https://doi.org/10.3390/ijerph19127231

Kananen, L., Hong, X., Annetorp, M., Mak, J. K. L., Jylhävä, J., Eriksdotter, M., Hägg, S., & Religa, D. (2023). Health progression for Covid-19 survivors hospitalized in geriatric clinics in Sweden. PloS One, 18(3). https://doi.org/10.1371/journal.pone.0283344

Kananen, L., Molnár, C., Ansker, F., Kozlowska, D. J., Hägg, S., Jylhävä, J., Religa, D., & Raaschou, P. (2023). Anticoagulant treatment and COVID-19 mortality among older adults living in nursing homes in Sweden. Health Science Reports, 6(11). https://doi.org/10.1002/hsr2.1692

Kanecki, K., Nitsch-Osuch, A., Goryński, P., Wojtyniak, B., Juszczyk, G., Magdalena, B., Kosińska, I., & Tyszko, P. (2021). Hospitalizations for COVID-19 in Poland : A study based on data from a national hospital register. Polish Archives of Internal Medicine, 131(6). https://doi.org/10.20452/pamw.15946

Kania, M., Koń, B., Kamiński, K., Hohendorff, J., Witek, P., Klupa, T., & Malecki, M. T. (2023). Diabetes as a risk factor of death in hospitalized COVID-19 patients—An analysis of a National Hospitalization Database from Poland, 2020. Frontiers in Endocrinology, 14. https://doi.org/10.3389/fendo.2023.1161637

Kania, M., Mazur, K., Terlecki, M., Matejko, B., Hohendorff, J., Chaykivska, Z., Fiema, M., Kopka, M., Kostrzycka, M., Wilk, M., Klupa, T., Witek, P., Katra, B., Klocek, M., Rajzer, M., & Malecki, M. T. (2023). Characteristics, Mortality, and Clinical Outcomes of Hospitalized Patients with COVID-19 and Diabetes : A Reference Single-Center Cohort Study from Poland. International Journal of Endocrinology, 2023. https://doi.org/10.1155/2023/8700302

Kant, R., Nguyen, P. T., Blomqvist, S., Erdin, M., Alburkat, H., Suvanto, M., Zakham, F., Salminen, V., Olander, V., Paloniemi, M., Huhti, L., Lehtinen, S., Luukinen, B., Jarva, H., Kallio-Kokko, H., Kurkela, S., Lappalainen, M., Liimatainen, H., Hannula, S., … Vapalahti, O. (2021). Incidence Trends for SARS-CoV-2 Alpha and Beta Variants, Finland, Spring 2021. Emerging Infectious Diseases, 27(12). https://doi.org/10.3201/eid2712.211631

Kantele, A., Lääveri, T., Kareinen, L., Pakkanen, S. H., Blomgren, K., Mero, S., Patjas, A., Virtanen, J., Uusitalo, R., Lappalainen, M., Järvinen, A., Kurkela, S., Jääskeläinen, A. J., Vapalahti, O., & Sironen, T. (2021). SARS-CoV-2 infections among healthcare workers at Helsinki University Hospital, Finland, spring 2020 : Serosurvey, symptoms and risk factors. Travel Medicine and Infectious Disease, 39. https://doi.org/10.1016/j.tmaid.2020.101949

Karadayi, Y., Aydin, M. N., & Ogrenci, A. S. (2020). Unsupervised Anomaly Detection in Multivariate Spatio-Temporal Data Using Deep Learning : Early Detection of COVID-19 Outbreak in Italy. IEEE Access: Practical Innovations, Open Solutions, 8. https://doi.org/10.1109/ACCESS.2020.3022366

Kardas, P., Zasowska, A., Dec, J., & Stachurska, M. (2011). Reasons for low influenza vaccination coverage : Cross-sectional survey in Poland. Croatian Medical Journal, 52(2). https://doi.org/10.3325/cmj.2011.52.126

Kasztelewicz, B., Janiszewska, K., Burzyńska, J., Szydłowska, E., Migdał, M., & Dzierżanowska-Fangrat, K. (2021). Prevalence of IgG antibodies against SARS-CoV-2 among healthcare workers in a tertiary pediatric hospital in Poland. PloS One, 16(4). https://doi.org/10.1371/journal.pone.0249550

Keeling, M. J., Hill, E. M., Petrou, S., Tran, P. B., Png, M. E., Staniszewska, S., Clark, C., Hassel, K., Stowe, J., & Andrews, N. (2024). Cost-effectiveness of routine COVID-19 adult vaccination programmes in England. medRxiv, (Keeling M.J., m.j.keeling@warwick.ac.uk) The Zeeman Institute for Systems Biology&Infectious Disease Epidemiology Research, School of Life Sciences, Mathematics Institute, University of Warwick, Coventry, United Kingdom. https://doi.org/10.1101/2024.11.08.24316972

Keeling, M. J., Moore, S., Penman, B., & Hill, E. M. (2022). The Impact of SARS-CoV-2 Vaccine Dose Separation and Dose Targeting on Hospital Admissions and Deaths from COVID-19 in England. medRxiv, (Keeling M.J., m.j.keeling@warwick.ac.uk; Penman B.; Hill E.M.) The Zeeman Institute for Systems Biology&Infectious Disease Epidemiology Research, University of Warwick, Coventry, United Kingdom. https://doi.org/10.1101/2022.08.22.22278973

Keeling, M. J., Tildesley, M. J., Atkins, B. D., Penman, B., Southall, E., Guyver-Fletcher, G., Holmes, A., McKimm, H., Gorsich, E. E., Hill, E. M., & Dyson, L. (2021). The impact of school reopening on the spread of COVID-19 in England. Philosophical Transactions of the Royal Society of London. Series B, Biological Sciences, 376(1829). https://doi.org/10.1098/rstb.2020.0261

Kejžar, N., & Lusa, L. (2020). Classification of weekly provincial overall age- and gender-specific mortality patterns during the COVID-19 epidemics in Italy. Epidemiologia E Prevenzione, 44(5-6 Suppl 2). https://doi.org/10.19191/EP20.5-6.S2.127

Keller, C., Chung, H., Jerrentrup, A., Feldmann, L., Rohde, C., Halwe, S., Wölfel, F., Günther, S., & Renz, H. (2022). Prevalence of SARS-CoV-2 antibodies in hospital employees, Central Germany. JOURNAL OF LABORATORY MEDICINE, 46(1). https://doi.org/10.1515/labmed-2021-0107

Kemp, F., Proverbio, D., Aalto, A., Mombaerts, L., d'Hérouël, A., Husch, A., Ley, C., Gonçalves, J., Skupin, A., & Magni, S. (2021). Modelling COVID-19 dynamics and potential for herd immunity by vaccination in Austria, Luxembourg and Sweden. JOURNAL OF THEORETICAL BIOLOGY, 530. https://doi.org/10.1016/j.jtbi.2021.110874

Kendall, M., Tsallis, D., Wymant, C., Di Francia, A., Balogun, Y., Didelot, X., Ferretti, L., & Fraser, C. (2023). Epidemiological impacts of the NHS COVID-19 app in England and Wales throughout its first year. Nature Communications, 14(1). https://doi.org/10.1038/s41467-023-36495-z

Kennedy, B., Fitipaldi, H., Hammar, U., Maziarz, M., Tsereteli, N., Oskolkov, N., Varotsis, G., Franks, C. A., Nguyen, D., Spiliopoulos, L., Adami, H.-O., Björk, J., Engblom, S., Fall, K., Grimby-Ekman, A., Litton, J.-E., Martinell, M., Oudin, A., Sjöström, T., … Fall, T. (2022). App-based COVID-19 syndromic surveillance and prediction of hospital admissions in COVID Symptom Study Sweden. Nature Communications, 13(1). https://doi.org/10.1038/s41467-022-29608-7

Keogh-Brown, M. R., Smith, R. D., Edmunds, J. W., & Beutels, P. (2010). The macroeconomic impact of pandemic influenza: Estimates from models of the United Kingdom, France, Belgium and The Netherlands. The European Journal of Health Economics: HEPAC: Health Economics in Prevention and Care, 11(6), 543–554. https://doi.org/10.1007/s10198-009-0210-1

Kern, A., Kuhlmann, P. H., Matl, S., Ege, M., Maison, N., Eckert, J., von Both, U., Behrends, U., Anger, M., Frühwald, M. C., Gerstlauer, M., Woelfle, J., Neubert, A., Melter, M., Liese, J., Goettler, D., Sing, A., Liebl, B., Hübner, J., … COVID Kids Bavaria Consortium. (2022). Surveillance of Acute SARS-CoV-2 Infections in Elementary Schools and Daycare Facilities in Bavaria, Germany (09/2020-03/2021). Frontiers in Pediatrics, 10. https://doi.org/10.3389/fped.2022.888498

Khazaei, Y., Küchenhoff, H., Hoffmann, S., Syliqi, D., & Rehms, R. (2023). Using a Bayesian hierarchical approach to study the association between non-pharmaceutical interventions and the spread of Covid-19 in Germany. Scientific Reports, 13(1). https://doi.org/10.1038/s41598-023-45950-2

Khedmati Morasae, E., Derbyshire, D. W., Amini, P., & Ebrahimi, T. (2024). Social determinants of spatial inequalities in COVID-19 outcomes across England : A multiscale geographically weighted regression analysis. SSM - Population Health, 25. https://doi.org/10.1016/j.ssmph.2024.101621

Kheifetz, Y., Kirsten, H., Schuppert, A., & Scholz, M. (2024). Modelling complete dynamics of SARS-CoV-2 pandemics of Germany and its federal states using multiple levels of data. medRxiv, (Kheifetz Y., yuri.kheifetz@imise.uni-leipzig.de; Kirsten H., holger.kirsten@imise.uni-leipzig.de; Schuppert A.; Scholz M., markus.scholz@imise.unileipzig.de) Institute for Medical Informatics, Statistics and Epidemiology, University of Leipzig, Haertelstrasse 16-18, Leipzig, Germany. https://doi.org/10.1101/2024.11.11.24317088

Khunti, K., Knighton, P., Zaccardi, F., Bakhai, C., Barron, E., Holman, N., Kar, P., Meace, C., Sattar, N., Sharp, S., Wareham, N. J., Weaver, A., Woch, E., Young, B., & Valabhji, J. (2021). Prescription of glucose-lowering therapies and risk of COVID-19 mortality in people with type 2 diabetes : A nationwide observational study in England. The Lancet. Diabetes & Endocrinology, 9(5). https://doi.org/10.1016/S2213-8587(21)00050-4

Kilcoyne, A., Jordan, E., Thomas, K., Pepper, A. N., Zhou, A., Chappell, D., Amarapala, M., Thériault, R.-K., & Thompson, M. (2022). Clinical and Economic Benefits of Lenzilumab Plus Standard of Care Compared with Standard of Care Alone for the Treatment of Hospitalized Patients with Coronavirus Disease 19 (COVID-19) from the Perspective of National Health Service England. ClinicoEconomics and Outcomes Research: CEOR, 14. https://doi.org/10.2147/CEOR.S360741

Killander Möller, I., Hedberg, P., Wagner, P., Lindahl, H., Nyström, S., Blixt, L., Eketorp Sylvan, S., Nilsdotter-Augustinsson, Å., Österborg, A., Fredrikson, M., Hansson, L., Kahn, F., Sparén, P., Gisslén, M., Nauclér, P., Bergman, P., Aleman, S., & Carlander, C. (2025). Risk of COVID-19 hospitalisation by HIV-status and SARS-CoV-2 vaccination status during pre- and post-Omicron era in a national register-based cohort study in Sweden. Infectious Diseases (London, England), 57(2). https://doi.org/10.1080/23744235.2024.2405582

Kindgen-Milles, D., Brandenburger, T., Braun, J., Cleff, C., Moussazadeh, K., Mrosewski, I., Timm, J., & Wetzchewald, D. (2021). Prevalence of SARS-COV-2 positivity in 516 German intensive care and emergency physicians studied by seroprevalence of antibodies National Covid Survey Germany (NAT-COV-SURV). PLOS ONE, 16(4). https://doi.org/10.1371/journal.pone.0248813

Kirsebom, F. C. M., Andrews, N., Mensah, A. A., Stowe, J., Ladhani, S., Ramsay, M., Lopez Bernal, J., & Campbell, H. J. (2024). Vaccine effectiveness against mild and severe covid-19 in pregnant individuals and their infants in England : Test negative case-control study. BMJ Medicine, 3(1). https://doi.org/10.1136/bmjmed-2023-000696

Kirsebom, F. C. M., Andrews, N., Sachdeva, R., Stowe, J., Ramsay, M., & Lopez Bernal, J. (2022). Effectiveness of ChAdOx1-S COVID-19 booster vaccination against the Omicron and Delta variants in England. Nature Communications, 13(1). https://doi.org/10.1038/s41467-022-35168-7

Kirsebom, F. C. M., Andrews, N., Stowe, J., Ramsay, M., & Lopez Bernal, J. (2023). Duration of protection of ancestral-strain monovalent vaccines and effectiveness of bivalent BA.1 boosters against COVID-19 hospitalisation in England : A test-negative case-control study. The Lancet. Infectious Diseases, 23(11). https://doi.org/10.1016/S1473-3099(23)00365-1

Kirsebom, F. C. M., Harman, K., Lunt, R. J., Andrews, N., Groves, N., Abdul Aziz, N., Hope, R., Stowe, J., Chand, M., Ramsay, M., Dabrera, G., Kall, M., & Bernal, J. L. (2023). Vaccine effectiveness against hospitalisation estimated using a test-negative case-control study design, and comparative odds of hospital admission and severe outcomes with COVID-19 sub-lineages BQ.1, CH.1.1. And XBB.1.5 in England. The Lancet Regional Health. Europe, 35. https://doi.org/10.1016/j.lanepe.2023.100755

Kirsebom, F. C. M., Stowe, J., Lopez Bernal, J., Allen, A., & Andrews, N. (2024). Effectiveness of autumn 2023 COVID-19 vaccination and residual protection of prior doses against hospitalisation in England, estimated using a test-negative case-control study. The Journal of Infection, 89(1). https://doi.org/10.1016/j.jinf.2024.106177

Kirwan, P. D., Charlett, A., Birrell, P., Elgohari, S., Hope, R., Mandal, S., De Angelis, D., & Presanis, A. M. (2022). Trends in COVID-19 hospital outcomes in England before and after vaccine introduction, a cohort study. Nature Communications, 13(1). https://doi.org/10.1038/s41467-022-32458-y

Kisiel, M. A., Lee, S., Janols, H., & Faramarzi, A. (2023). Absenteeism Costs Due to COVID-19 and Their Predictors in Non-Hospitalized Patients in Sweden : A Poisson Regression Analysis. International Journal of Environmental Research and Public Health, 20(22). https://doi.org/10.3390/ijerph20227052

Kjeldsen, J., Nielsen, J., Ellingsen, T., Knudsen, T., Nielsen, R. G., Larsen, M. D., Lund, K., & Nørgård, B. M. (2021). Outcome of COVID-19 in hospitalized patients with chronic inflammatory diseases. A population based national register study in Denmark. Journal of Autoimmunity, 120. https://doi.org/10.1016/j.jaut.2021.102632

Klaesson, J., Lobo, J., & Mellander, C. (2023). Social interactions and COVID-19 vaccine hesitancy : Evidence from a full population study in Sweden. PloS One, 18(11). https://doi.org/10.1371/journal.pone.0289309

Klak, A., Furmanczyk, K., Nowicka, P., Manczak, M., Baranska, A., Religioni, U., Siekierska, A., Ambroziak, M., & Chlopek, M. (2022). The Relationship between Searches for COVID-19 Vaccines and Dynamics of Vaccinated People in Poland : An Infodemiological Study. INTERNATIONAL JOURNAL OF ENVIRONMENTAL RESEARCH AND PUBLIC HEALTH, 19(20). https://doi.org/10.3390/ijerph192013275

Klee, B., Diexer, S., Sarajan, M. H., Glaser, N., Binder, M., Frese, T., Girndt, M., Sedding, D., Hoell, J. I., Moor, I., Gekle, M., Mikolajczyk, R., & Gottschick, C. (2023). Regional Differences in Uptake of Vaccination against COVID-19 and Influenza in Germany : Results from the DigiHero Cohort. Vaccines, 11(11). https://doi.org/10.3390/vaccines11111640

Kliim-Hansen, V., Johansson, K. S., Gasbjerg, L. S., Jimenez-Solem, E., Petersen, T. S., Nyeland, M. E., Winther-Jensen, M., Ankarfeldt, M. Z., Pedersen, M. G., Ellegaard, A.-M., Knop, F. K., & Christensen, M. B. (2024). The impact of type 2 diabetes and glycaemic control on mortality and clinical outcomes in hospitalized patients with COVID-19 in the capital region of Denmark. Diabetes, Obesity & Metabolism, 26(1). https://doi.org/10.1111/dom.15302

Kloka, J. A., Blum, L. V., Old, O., Zacharowski, K., & Friedrichson, B. (2022). Characteristics and mortality of 561,379 hospitalized COVID-19 patients in Germany until December 2021 based on real-life data. Scientific Reports, 12(1). https://doi.org/10.1038/s41598-022-15287-3

Knight, G. M., Pham, T. M., Stimson, J., Funk, S., Jafari, Y., Pople, D., Evans, S., Yin, M., Brown, C. S., Bhattacharya, A., Hope, R., Semple, M. G., ISARIC4C Investigators, CMMID COVID-19 Working Group, Read, J. M., Cooper, B. S., & Robotham, J. V. (2022). The contribution of hospital-acquired infections to the COVID-19 epidemic in England in the first half of 2020. BMC Infectious Diseases, 22(1). https://doi.org/10.1186/s12879-022-07490-4

Knock, E. S., Whittles, L. K., Lees, J. A., Perez-Guzman, P. N., Verity, R., FitzJohn, R. G., Gaythorpe, K. A. M., Imai, N., Hinsley, W., Okell, L. C., Rosello, A., Kantas, N., Walters, C. E., Bhatia, S., Watson, O. J., Whittaker, C., Cattarino, L., Boonyasiri, A., Djaafara, B. A., … Baguelin, M. (2021). Key epidemiological drivers and impact of interventions in the 2020 SARS-CoV-2 epidemic in England. Science Translational Medicine, 13(602). https://doi.org/10.1126/scitranslmed.abg4262

Koch, S., Hoffmann, C., Caseiro, A., Ledebur, M., Menk, M., & von Schneidemesser, E. (2022). Air quality in Germany as a contributing factor to morbidity from COVID-19. Environmental Research, 214(Pt 2). https://doi.org/10.1016/j.envres.2022.113896

Kodde, C., Hohenstein, S., Nachtigall, I., Cavalli, Y., Schuepbach, R., Graf, R., Bollmann, A., & Kuhlen, R. (2024). Comparison of SARS-CoV-2 related in-hospital mortality, ICU admission and mechanical ventilation of 1.4 million patients in Germany and Switzerland, 2019 to 2022. Infection. https://doi.org/10.1007/s15010-024-02412-9

Koetz, A., Nilsson, P., Lindén, M., van der Hoek, L., & Ripa, T. (2006). Detection of human coronavirus NL63, human metapneumovirus and respiratory syncytial virus in children with respiratory tract infections in south-west Sweden. Clinical Microbiology and Infection: The Official Publication of the European Society of Clinical Microbiology and Infectious Diseases, 12(11). https://doi.org/10.1111/j.1469-0691.2006.01506.x

Kohli, M. A., Maschio, M., Cartier, S., Mould-Quevedo, J., & Fricke, F.-U. (2022). The Cost-Effectiveness of Vaccination of Older Adults with an MF59-Adjuvanted Quadrivalent Influenza Vaccine Compared to Other Available Quadrivalent Vaccines in Germany. Vaccines, 10(9). https://doi.org/10.3390/vaccines10091386

Kolhe, N. V., Fluck, R. J., & Taal, M. W. (2024). Regional variation of COVID-19 admissions, acute kidney injury and mortality in England—A national observational study using administrative data. BMC Infectious Diseases, 24(1). https://doi.org/10.1186/s12879-024-09210-6

Kolhe, N. V., Fluck, R., & Taal, M. (2025). Effect of COVID-19 with or without acute kidney injury on inpatient mortality in England: A national observational study using administrative data. BMJ Open, 15(3), e095020. https://doi.org/10.1136/bmjopen-2024-095020

Kondratiuk, K., Hallmann, E., Szymański, K., Łuniewska, K., Poznańska, A., & Brydak, L. B. (2024). Prevalence of circulating antibodies against hemagglutinin of influenza viruses in epidemic season 2021/2022 in Poland. Acta Biochimica Polonica, 71. https://doi.org/10.3389/abp.2024.12289

Konermann, F. M., Gessler, N., Wohlmuth, P., Behr, J., Feldhege, J., Gloeckner, C., Gunawardene, M. A., Herrlinger, K. R., Hoelting, T., Pape, U.-F., Reinmuth, N., Stang, A., Sheikhzadeh, S., Arnold, D., & Wesseler, C. (2023). High In-Hospital Mortality in SARS-CoV-2-Infected Patients with Active Cancer Disease during Omicron Phase of the Pandemic: Insights from the CORONA Germany Study. Oncology Research and Treatment, 46(5), 201–210. https://doi.org/10.1159/000529788

Konstantinoudis, G., Padellini, T., Bennett, J., Davies, B., Ezzati, M., & Blangiardo, M. (2021). Long-term exposure to air-pollution and COVID-19 mortality in England : A hierarchical spatial analysis. Environment International, 146. https://doi.org/10.1016/j.envint.2020.106316

Kopciuch, D., Hoffmann, K., Kus, K., Koligat-Seitz, A., Ratajczak, P., Nowakowska, E., & Paczkowska, A. (2024). Analysis of Attitudes and Practices towards the Influenza Vaccine in High-Risk Adults in Poland. Vaccines, 12(3). https://doi.org/10.3390/vaccines12030341

Koppe, U., Schilling, J., Stecher, M., Rüthrich, M. M., Marquis, A., Diercke, M., Haselberger, M., Koll, C. E. M., Niebank, M., Ruehe, B., Borgmann, S., Grabenhenrich, L., Hellwig, K., Pilgram, L., Spinner, C. D., Paerisch, T., & LEOSS study group. (2023). Disease severity in hospitalized COVID-19 patients : Comparing routine surveillance with cohort data from the LEOSS study in 2020 in Germany. BMC Infectious Diseases, 23(1). https://doi.org/10.1186/s12879-023-08035-z

Körner, R. W., & Weber, L. T. (2021). Prevalence of COVID-19 Among Children and Adolescents While Easing Lockdown Restrictions in Cologne, North Rhine-Westphalia, Germany. Klinische Padiatrie, 233(3). https://doi.org/10.1055/a-1341-9530

Korth, J., Wilde, B., Dolff, S., Anastasiou, O. E., Krawczyk, A., Jahn, M., Cordes, S., Ross, B., Esser, S., Lindemann, M., Kribben, A., Dittmer, U., Witzke, O., & Herrmann, A. (2020). SARS-CoV-2-specific antibody detection in healthcare workers in Germany with direct contact to COVID-19 patients. Journal of Clinical Virology: The Official Publication of the Pan American Society for Clinical Virology, 128. https://doi.org/10.1016/j.jcv.2020.104437

Kos, M., Hatalska-Żerebiec, R., Jurczyk-Stachyra, M., Paciura, K., Janeczko, J., Podsiadły, R., & Sobolewski, P. (2025). Analysis of selected factors influencing mortality of patients with COVID-19 treated in a community hospital in a rural region of Poland. Rural and Remote Health, 25(1). https://doi.org/10.22605/RRH8939

Koschollek, C., Kajikhina, K., El Bcheraoui, C., Wieler, L. H., Michalski, N., & Hövener, C. (2023). SARS-CoV-2 infections in migrant populations in Germany : Results from the COVID-19 snapshot monitoring survey. Public Health, 219. https://doi.org/10.1016/j.puhe.2023.03.015

Kostev, K., Gessler, N., Wohlmuth, P., Arnold, D., Bein, B., Bohlken, J., Herrlinger, K., Jacob, L., Koyanagi, A., Nowak, L., Smith, L., Wesseler, C., Sheikhzadeh, S., & Wollmer, M. A. (2023). Is Dementia Associated with COVID-19 Mortality ? A Multicenter Retrospective Cohort Study Conducted in 50 Hospitals in Germany. Journal of Alzheimer's Disease: JAD, 91(2). https://doi.org/10.3233/JAD-220918

Kostev, K., Smith, L., Koyanagi, A., & Jacob, L. (2022). Prevalence of and Factors Associated With Post-Coronavirus Disease 2019 (COVID-19) Condition in the 12 Months After the Diagnosis of COVID-19 in Adults Followed in General Practices in Germany. Open Forum Infectious Diseases, 9(7). https://doi.org/10.1093/ofid/ofac333

Kotronia, E., Rosinska, M., Stepien, M., Czerwinski, M., & Sadkowska-Todys, M. (2023). Willingness to vaccinate among adults, and factors associated with vaccine acceptance of COVID-19 vaccines in a nationwide study in Poland between March 2021 and April 2022. Frontiers in Public Health, 11. https://doi.org/10.3389/fpubh.2023.1235585

Kowall, B., Standl, F., Oesterling, F., Brune, B., Brinkmann, M., Dudda, M., Pflaumer, P., Jöckel, K.-H., & Stang, A. (2021). Excess mortality due to Covid-19 ? A comparison of total mortality in 2020 with total mortality in 2016 to 2019 in Germany, Sweden and Spain. PloS One, 16(8). https://doi.org/10.1371/journal.pone.0255540

Kowalska, J. D., Lara, M., Hlebowicz, M., Mularska, E., Jabłonowska, E., Siwak, E., Wandałowicz, A., Witak-Jędra, M., Olczak, A., Bociąga-Jasik, M., Suchacz, M., Stempkowska-Rejek, J., Wasilewski, P., & Parczewski, M. (2023). Non-HIV-related comorbidities and uncontrolled HIV replication are independent factors increasing the odds of hospitalization due to COVID-19 among HIV-positive patients in Poland. Infection, 51(2). https://doi.org/10.1007/s15010-022-01887-8

Kowalska, M., & Niewiadomska, E. (2022). Spatial Variability of COVID-19 Hospitalization in the Silesian Region, Poland. International Journal of Environmental Research and Public Health, 19(15). https://doi.org/10.3390/ijerph19159007

Kowalska, M., Barański, K., Brożek, G., Kaleta-Pilarska, A., & Zejda, J. E. (2021). COVID-19-related risk of in-hospital death in Silesia, Poland. Polish Archives of Internal Medicine, 131(4). https://doi.org/10.20452/pamw.15893

Kowalska, M., de Winter, P., Godderis, L., Boone, A., & Szemik, S. (2023). Hospital medical care and the COVID-19 mortality in METEOR partner countries (the Netherlands, Belgium, Italy, And Poland). International Journal of Occupational Medicine and Environmental Health, 36(3). https://doi.org/10.13075/ijomeh.1896.02187

Kramer, R., Duclos, A., VRS study group in Lyon, Lina, B., & Casalegno, J.-S. (2018). Cost and burden of RSV related hospitalisation from 2012 to 2017 in the first year of life in Lyon, France. Vaccine, 36(45). https://doi.org/10.1016/j.vaccine.2018.09.029

Krauer, F., Guenther, F., Treskova-Schwarzbach, M., Schoenfeld, V., Koltai, M., Jit, M., Hodgson, D., Schneider, U., Wichmann, O., Harder, T., Sandmann, F., & Flasche, S. (2024). Effectiveness and efficiency of immunisation strategies to prevent RSV among infants and older adults in Germany: A modelling study. BMC MEDICINE, 22(1). https://doi.org/10.1186/s12916-024-03687-3

Krogsgaard, L. W., Benedetti, G., Gudde, A., Richter, S. R., Rasmussen, L. D., Midgley, S. E., Qvesel, A. G., Nauta, M., Bahrenscheer, N. S., von Kappelgaard, L., McManus, O., Hansen, N. C., Pedersen, J. B., Haimes, D., Gamst, J., Nørgaard, L. S., Jørgensen, A. C. U., Ejegod, D. M., Møller, S. S., … Ethelberg, S. (2024). Results from the SARS-CoV-2 wastewater-based surveillance system in Denmark, July 2021 to June 2022. Water Research, 252. https://doi.org/10.1016/j.watres.2024.121223

Kromer, C., Wellmann, P., Kromer, D., Patt, S., Mohr, J., Wilsmann-Theis, D., & Mössner, R. (2024). Impact of COVID-19 on Influenza and Pneumococcal Vaccination of Psoriatic Patients in Germany : Results from Vac-Pso. Vaccines, 12(6). https://doi.org/10.3390/vaccines12060614

Kroneman, M. W., & van Essen, G. A. (2007a). Stagnating influenza vaccine coverage rates among high-risk groups in Poland and Sweden in 2003/4 and 2004/5. Euro Surveillance: Bulletin Europeen Sur Les Maladies Transmissibles = European Communicable Disease Bulletin, 12(4). https://doi.org/10.2807/esm.12.04.00696-en

Kroneman, M. W., & van Essen, G. A. (2007b). Variations in influenza vaccination coverage among the high-risk population in Sweden in 2003/4 and 2004/5 : A population survey. BMC Public Health, 7. https://doi.org/10.1186/1471-2458-7-113

Krutikov, M., Palmer, T., Tut, G., Fuller, C., Azmi, B., Giddings, R., Shrotri, M., Kaur, N., Sylla, P., Lancaster, T., Irwin-Singer, A., Hayward, A., Moss, P., Copas, A., & Shallcross, L. (2022). Prevalence and duration of detectable SARS-CoV-2 nucleocapsid antibodies in staff and residents of long-term care facilities over the first year of the pandemic (VIVALDI study): Prospective cohort study in England. The Lancet. Healthy Longevity, 3(1), e13–e21. https://doi.org/10.1016/S2666-7568(21)00282-8

Kuchar, E., Ludwikowska, K., Marciniak, D., Szenborn, L., & Nitsch-Osuch, A. (2017). Public Perception of the Risks Associated with Infectious Diseases in Poland : Ebola and Influenza and Their Impact on the Attitude to Vaccination. Advances in Experimental Medicine and Biology, 980. https://doi.org/10.1007/5584_2016_207

Kuchar, E., Nitsch-Osuch, A., Karpinska, T., Kurpas, D., Zycinska, K., Wardyn, K., & Szenborn, L. (2013). Pandemic influenza in the 2009/2010 season in central Poland: The surveillance study of laboratory confirmed cases. Respiratory Physiology & Neurobiology, 187(1), 94–98. https://doi.org/10.1016/j.resp.2013.02.013

Kuciel, N., Mazurek, J., Hap, K., Marciniak, D., Biernat, K., & Sutkowska, E. (2022). COVID-19 Vaccine Acceptance in Pregnant and Lactating Women and Mothers of Young Children in Poland. International Journal of Women's Health, 14. https://doi.org/10.2147/IJWH.S348652

Kuczborska, K., & Książyk, J. (2021). Prevalence and Course of SARS-CoV-2 Infection among Immunocompromised Children Hospitalised in the Tertiary Referral Hospital in Poland. Journal of Clinical Medicine, 10(19). https://doi.org/10.3390/jcm10194556

Kuhdari, P., Brosio, F., Malaventura, C., Stefanati, A., Orsi, A., Icardi, G., & Gabutti, G. (2018). Human respiratory syncytial virus and hospitalization in young children in Italy. Italian Journal of Pediatrics, 44(1). https://doi.org/10.1186/s13052-018-0492-y

Kühn, M. J., Abele, D., Mitra, T., Koslow, W., Abedi, M., Rack, K., Siggel, M., Khailaie, S., Klitz, M., Binder, S., Spataro, L., Gilg, J., Kleinert, J., Häberle, M., Plötzke, L., Spinner, C. D., Stecher, M., Zhu, X. X., Basermann, A., & Meyer-Hermann, M. (2021). Assessment of effective mitigation and prediction of the spread of SARS-CoV-2 in Germany using demographic information and spatial resolution. Mathematical Biosciences, 339. https://doi.org/10.1016/j.mbs.2021.108648

Kwon, J., Mensah, J., Milne, R., Rayner, C., Lawrence, R. R., De Kock, J., Sivan, M., & Petrou, S. (2025). Health economic outcomes and national economic impacts associated with Long COVID in England and Scotland. European Journal of Health Economics. https://doi.org/10.1007/s10198-025-01788-1

La Rosa, G., Brandtner, D., Bonanno Ferraro, G., Veneri, C., Mancini, P., Iaconelli, M., Lucentini, L., Del Giudice, C., Orlandi, L., SARI network, & Suffredini, E. (2023). Wastewater surveillance of SARS-CoV-2 variants in October-November 2022 in Italy : Detection of XBB.1, BA.2.75 and rapid spread of the BQ.1 lineage. The Science of the Total Environment, 873. https://doi.org/10.1016/j.scitotenv.2023.162339

La Rosa, G., Iaconelli, M., Mancini, P., Bonanno Ferraro, G., Veneri, C., Bonadonna, L., Lucentini, L., & Suffredini, E. (2020). First detection of SARS-CoV-2 in untreated wastewaters in Italy. The Science of the Total Environment, 736. https://doi.org/10.1016/j.scitotenv.2020.139652

La Rosa, G., Iaconelli, M., Veneri, C., Mancini, P., Bonanno Ferraro, G., Brandtner, D., Lucentini, L., Bonadonna, L., Rossi, M., Grigioni, M., SARI network, & Suffredini, E. (2022). The rapid spread of SARS-COV-2 Omicron variant in Italy reflected early through wastewater surveillance. The Science of the Total Environment, 837. https://doi.org/10.1016/j.scitotenv.2022.155767

La Rosa, G., Mancini, P., Bonanno Ferraro, G., Veneri, C., Iaconelli, M., Bonadonna, L., Lucentini, L., & Suffredini, E. (2021). SARS-CoV-2 has been circulating in northern Italy since December 2019 : Evidence from environmental monitoring. The Science of the Total Environment, 750. https://doi.org/10.1016/j.scitotenv.2020.141711

La Torre, G., Iarocci, G., Cadeddu, C., & Boccia, A. (2010). Influence of sociodemographic inequalities and chronic conditions on influenza vaccination coverage in Italy : Results from a survey in the general population. Public Health, 124(12). https://doi.org/10.1016/j.puhe.2010.06.006

Laarman, C., Hahné, S. J., de Melker, H. E., & Knol, M. J. (2024). SARS-CoV-2 risk factors among symptomatic vaccinated adults attending community testing locations in the Netherlands from June 2021 till February 2022. PloS One, 19(12). https://doi.org/10.1371/journal.pone.0311229

Labuschagne, L. J. E., Smorenburg, N., van de Kassteele, J., Bom, B., de Weerdt, A. C., de Melker, H. E., & Hahné, S. J. M. (2023). Neighbourhood sociodemographic factors and COVID-19 vaccine uptake in the Netherlands : An ecological analysis. BMC Public Health, 23(1). https://doi.org/10.1186/s12889-023-16600-z

Lacy, J., Mensah, A., Simmons, R., Andrews, N., Siddiqui, M. R., Bukasa, A., O'Boyle, S., Campbell, H., & Brown, K. (2022). Protective effect of a first SARS-CoV-2 infection from reinfection : A matched retrospective cohort study using PCR testing data in England. Epidemiology and Infection, 150. https://doi.org/10.1017/S0950268822000966

Lade, C., Bayer, L., Huebbe, B., Riedel, J., Melnik, S., Brestrich, G., von Eiff, C., & Tenenbaum, T. (2024). Clinical and economic inpatient burden of respiratory syncytial virus (RSV) infections in children < 2 years of age in Germany, 2014-2019 : A retrospective health claims analysis. medRxiv, (Lade C., caroline.lade@pfizer.com; Bayer L.; Brestrich G.; von Eiff C.) Pfizer Pharma GmbH, Berlin, Germany. https://doi.org/10.1101/2024.02.12.24302675

Ladhani, S. N., Baawuah, F., Beckmann, J., Okike, I. O., Ahmad, S., Garstang, J., Brent, A. J., Brent, B., Walker, J., Andrews, N., Ireland, G., Aiano, F., Amin-Chowdhury, Z., Letley, L., Flood, J., Jones, S. E. I., Borrow, R., Linley, E., Zambon, M., … Ramsay, M. E. (2021). SARS-CoV-2 infection and transmission in primary schools in England in June-December, 2020 (sKIDs) : An active, prospective surveillance study. The Lancet. Child & Adolescent Health, 5(6). https://doi.org/10.1016/S2352-4642(21)00061-4

Ladhani, S. N., Ireland, G., Baawuah, F., Beckmann, J., Okike, I. O., Ahmad, S., Garstang, J., Brent, A. J., Brent, B., Aiano, F., Amin-Chowdhury, Z., Kall, M., Borrow, R., Linley, E., Zambon, M., Poh, J., Warrener, L., Lackenby, A., Ellis, J., … Ramsay, M. E. (2021a). Emergence of SARS-CoV-2 Alpha (B.1.1.7) variant, infection rates, antibody seroconversion and seroprevalence rates in secondary school students and staff : Active prospective surveillance, December 2020 to March 2021, England. The Journal of Infection, 83(5). https://doi.org/10.1016/j.jinf.2021.08.019

Ladhani, S. N., Ireland, G., Baawuah, F., Beckmann, J., Okike, I. O., Ahmad, S., Garstang, J., Brent, A. J., Brent, B., Aiano, F., Amin-Chowdhury, Z., Kall, M., Borrow, R., Linley, E., Zambon, M., Poh, J., Warrener, L., Lackenby, A., Ellis, J., … Ramsay, M. E. (2022). Emergence of the delta variant and risk of SARS-CoV-2 infection in secondary school students and staff : Prospective surveillance in 18 schools, England. EClinicalMedicine, 45. https://doi.org/10.1016/j.eclinm.2022.101319

Ladhani, S. N., Jeffery-Smith, A., Patel, M., Janarthanan, R., Fok, J., Crawley-Boevey, E., Vusirikala, A., Fernandez Ruiz De Olano, E., Perez, M. S., Tang, S., Dun-Campbell, K., Evans, E. W.-, Bell, A., Patel, B., Amin-Chowdhury, Z., Aiano, F., Paranthaman, K., Ma, T., Saavedra-Campos, M., … Zambon, M. (2020). High prevalence of SARS-CoV-2 antibodies in care homes affected by COVID-19 : Prospective cohort study, England. EClinicalMedicine, 28. https://doi.org/10.1016/j.eclinm.2020.100597

Lafaurie, M., Martin-Blondel, G., Delobel, P., Charpentier, S., Sommet, A., & Moulis, G. (2021). Outcome of patients hospitalized for COVID-19 and exposure to angiotensin-converting enzyme inhibitors and angiotensin-receptor blockers in France : Results of the ACE-CoV study. Fundamental & Clinical Pharmacology, 35(1). https://doi.org/10.1111/fcp.12613

Laffont-Lozes, P., Laureillard, D., Loubet, P., Stephan, R., Chiaruzzi, M., Clemmer, E., Martin, A., Roger, C., Muller, L., Claret, P.-G., Goulabchand, R., Roux, C., Lavigne, J.-P., Sotto, A., & Larcher, R. (2023). Effect of Tocilizumab on Mortality in Patients with SARS-CoV-2 Pneumonia Caused by Delta or Omicron Variants : A Propensity-Matched Analysis in Nimes University Hospital, France. Antibiotics (Basel, Switzerland), 12(1). https://doi.org/10.3390/antibiotics12010088

Lagier, J.-C., Million, M., Cortaredona, S., Delorme, L., Colson, P., Fournier, P.-E., Brouqui, P., Raoult, D., Parola, P., & IHU Task Force. (2022). Outcomes of 2111 COVID-19 Hospitalized Patients Treated with Hydroxychloroquine/Azithromycin and Other Regimens in Marseille, France, 2020 : A Monocentric Retrospective Analysis. Therapeutics and Clinical Risk Management, 18. https://doi.org/10.2147/TCRM.S364022

Lai, P. L., Panatto, D., Ansaldi, F., Canepa, P., Amicizia, D., Patria, A. G., & Gasparini, R. (2011). Burden of the 1999-2008 seasonal influenza epidemics in Italy: Comparison with the H1N1v (A/California/07/09) pandemic. Human Vaccines, 7, 217–225. https://doi.org/10.4161/hv.7.0.14607

Lajot, A., Cornelissen, L., Van Cauteren, D., Meurisse, M., Brondeel, R., & Dupont-Gillain, C. (2023). Comparing the incidence of SARS-CoV-2 across age groups considering sampling biases—Use of testing data

of autumn 2021 in Belgium. Archives of Public Health = Archives Belges De Sante Publique, 81(1). https://doi.org/10.1186/s13690-023-01072-9

Laliotis, I., & Minos, D. (2022). Religion, social interactions, and COVID-19 incidence in Western Germany. European Economic Review, 141. https://doi.org/10.1016/j.euroecorev.2021.103992

Lambooij, M. S., Pijpers, J., van de Kassteele, J., Fransen, M. P., Hahné, S. J., Hof, N., Kroese, F. M., de Melker, H., van Dijk, M., Uiters, E., & de Bruin, M. (2024). Mobile vaccination units to increase COVID-19 vaccination uptake in areas with lower coverage : A within-neighbourhood analysis using national registration data, the Netherlands, September-December 2021. Euro Surveillance: Bulletin Europeen Sur Les Maladies Transmissibles = European Communicable Disease Bulletin, 29(34). https://doi.org/10.2807/1560-7917.ES.2024.29.34.2300503

Lampl, B. M. J., Edenharter, B., Leitzmann, M. F., & Salzberger, B. (2023). COVID-19-related deaths : A 2-year inter-wave comparison of mortality data from Germany. Infection, 51(4). https://doi.org/10.1007/s15010-023-01982-4

Landmesser, J. (2021). The use of the dynamic time warping (DTW) method to describe the COVID-19 dynamics in Poland. OECONOMIA COPERNICANA, 12(3). https://doi.org/10.24136/oc.2021.018

Lang, A.-L., Hohmuth, N., Višković, V., Konigorski, S., Scholz, S., Balzer, F., Remschmidt, C., & Leistner, R. (2024). COVID-19 Vaccine Effectiveness and Digital Pandemic Surveillance in Germany (eCOV Study): Web Application-Based Prospective Observational Cohort Study. Journal of Medical Internet Research, 26, e47070. https://doi.org/10.2196/47070

Lang, J. C., Kura, K., Garba, S. M., Elbasha, E. H., & Chen, Y.-H. (2024). Comparison of a static cohort model and dynamic transmission model for respiratory syncytial virus intervention programs for infants in England and Wales. Vaccine, 42(8). Embase. https://doi.org/10.1016/j.vaccine.2024.02.004

Langedijk, A. C., van den Dungen, F., Harteveld, L., van den Boer, J., Smit, L., Averin, A., Quinn, E., Atwood, M., Law, A., Mendes, D., & van Houten, M. (2025). Cost-effectiveness of immunization strategies to protect infants against respiratory syncytial virus in the Netherlands. Human Vaccines & Immunotherapeutics, 21(1), 2521912. https://doi.org/10.1080/21645515.2025.2521912

Länsivaara, A., Lehto, K., Hyder, R., Luomala, O., Lipponen, A., Hokajärvi, A., Heikinheimo, A., Pitkänen, T., & Oikarinen, S. (2024). Wastewater-Based Surveillance of Respiratory Syncytial Virus Epidemic at the National Level in Finland. ACS ES&T WATER, 4(6). https://doi.org/10.1021/acsestwater.3c00752

Lapeña, S., Robles, M. B., Castañón, L., Martínez, J. P., Reguero, S., Alonso, M. P., & Fernández, I. (2005). Climatic factors and lower respiratory tract infection due to respiratory syncytial virus in hospitalised infants in northern Spain. European Journal of Epidemiology, 20(3). https://doi.org/10.1007/s10654-004-4539-6

Larrauri Cámara, A., Jiménez-Jorge, S., Mateo Ontañón, S. de, Pozo Sánchez, F., Ledesma Moreno, J., Casas Flecha, I., & Spanish Influenza Surveillance System (SISS). (2012). Epidemiology of the 2009 influenza pandemic in Spain. The Spanish Influenza Surveillance System. Enfermedades Infecciosas Y Microbiologia Clinica, 30, 2–9. https://doi.org/10.1016/S0213-005X(12)70098-8

Larrauri, A., Savulescu, C., Jiménez-Jorge, S., Pérez-Breña, P., Pozo, F., Casas, I., Ledesma, J., de Mateo, S., & Spanish Influenza Surveillance System (SISS). (2011). Influenza pandemic (H1N1) 2009 activity during summer 2009. Effectiveness of the 2008-9 trivalent vaccine against pandemic influenza in Spain. Gaceta Sanitaria, 25(1), 23–28. https://doi.org/10.1016/j.gaceta.2010.06.010

Larsen, C. S., Westergaard, C. L., Stærke, N. B., Arnet, U., Liu, G., Kantsø, L. R., & Kjellberg, J. (2025). Clinical outcomes among COVID-19 patients initiated on molnupiravir in Denmark—A national registry study. Antiviral Therapy, 30(1). https://doi.org/10.1177/13596535241313244

Larsen, L., Bistrup, C., Sørensen, S. S., Boesby, L., Nguyen, M. T. T., & Johansen, I. S. (2021). The coverage of influenza and pneumococcal vaccination among kidney transplant recipients and waiting list patients : A cross-sectional survey in Denmark. Transplant Infectious Disease: An Official Journal of the Transplantation Society, 23(3). https://doi.org/10.1111/tid.13537

Larsen, L., Nguyen, M. T. T., & Johansen, I. S. (2021). The coverage of influenza and pneumococcal vaccinations among people living with HIV in Denmark : A single-center cross-sectional survey. Human Vaccines & Immunotherapeutics, 17(8). https://doi.org/10.1080/21645515.2021.1894895

Larsen, T., Bosworth, M. L., Ayoubkhani, D., Schofield, R., Ali, R., Khunti, K., Walker, A. S., Glickman, M., & Nafilyan, V. (2022). Disparities in SARS-CoV-2 case rates by ethnicity, religion, measures of socio-economic position, English proficiency, and self-reported disability : Cohort study of 39 million people in England during the Alpha and Delta waves. medRxiv, (Larsen T., tim.larsen@ons.gov.uk; Bosworth M.L.; Ayoubkhani D.; Schofield R.; Glickman M.; Nafilyan V.) Office for National Statistics, Health Analysis and Life Events, Newport, United Kingdom. https://doi.org/10.1101/2022.03.02.22271762

Larson, A., Musse, I., Mitrovich, R., Shanmugam, P., Vohra, D., Lansdale, A. J., Bencina, G., Le Fevre, T., & Eiden, A. L. (2025). The effect of pharmacists as vaccinators on uptake of influenza vaccination in Denmark: An interrupted time series analysis. Research in Social & Administrative Pharmacy: RSAP, S1551-7411(25)00367-5. https://doi.org/10.1016/j.sapharm.2025.06.100

Larsson, A. C., Palstam, A., & Persson, H. C. (2021). Physical Function, Cognitive Function, and Daily Activities in Patients Hospitalized Due to COVID-19 : A Descriptive Cross-Sectional Study in Sweden. International Journal of Environmental Research and Public Health, 18(21). https://doi.org/10.3390/ijerph182111600

Larsson, E., Brattström, O., Agvald-Öhman, C., Grip, J., Campoccia Jalde, F., Strålin, K., Nauclér, P., Oldner, A., Konrad, D., Persson, B. P., Eriksson, L. I., Mårtensson, J., & Karolinska Intensive Care COVID-19 Study Group. (2021). Characteristics and outcomes of patients with COVID-19 admitted to ICU in a tertiary hospital in Stockholm, Sweden. Acta Anaesthesiologica Scandinavica, 65(1). https://doi.org/10.1111/aas.13694

Lastrucci, V., Lorini, C., Stacchini, L., Stancanelli, E., Guida, A., Radi, A., Morittu, C., Zimmitti, S., Alderotti, G., Del Riccio, M., Bechini, A., Boccalini, S., Covid-Population Research Group, null, & Bonaccorsi, G. (2022). Determinants of Actual COVID-19 Vaccine Uptake in a Cohort of Essential Workers : An Area-Based Longitudinal Study in the Province of Prato, Italy. International Journal of Environmental Research and Public Health, 19(20). https://doi.org/10.3390/ijerph192013216

Lastrucci, V., Pacifici, M., Alderotti, G., Puglia, M., Berti, E., Barbati, F., Lodi, L., Boscia, S., Nieddu, F., Indolfi, G., Peroni, D., Martini, M., Azzari, C., Voller, F., Moriondo, M., & Ricci, S. (2025). The impact of nirsevimab prophylaxis on RSV hospitalizations: A real-world cost-benefit analysis in Tuscany, Italy. Frontiers in Public Health, 13, 1604331. https://doi.org/10.3389/fpubh.2025.1604331

Latorre-Millán, M., Rodríguez Del Águila, M. M., Clusa, L., Mazagatos, C., Larrauri, A., Fernández, M. A., Rezusta, A., & Milagro, A. M. (2024). Severity Patterns in COVID-19 Hospitalised Patients in Spain : I-MOVE-COVID-19 Study. Viruses, 16(11). https://doi.org/10.3390/v16111705

Lattanzio, S. (2022). The Impact of Schools on the Transmission of Sars-Cov-2 : Evidence from Italy. medRxiv, (Lattanzio S., salvatore.lattanzio@bancaditalia.it) Bank of Italy, Italy. https://doi.org/10.1101/2022.07.18.22276940

Lau, K., Hauck, K., & Miraldo, M. (2019). Excess influenza hospital admissions and costs due to the 2009 H1N1 pandemic in England. Health Economics, 28(2), 175–188. https://doi.org/10.1002/hec.3834

Laza, R., Lazureanu, V. E., Musta, V. F., Nicolescu, N. D., Vernic, C., Tudor, A., Critu, R., Vilceanu, L., Paczeyka, R., Profir, V. B., Marinescu, A. R., Mocanu, A., Cut, T. G., Lighezan, D. F., & Baditoiu, L. M. (2022). COVID-19 Independent Risk Factors for Unfavorable Disease Progression: A Cross-Sectional Study from Romania. INTERNATIONAL JOURNAL OF GENERAL MEDICINE, 15, 2025–2036. https://doi.org/10.2147/IJGM.S350920

Laza, R., Musta, V. F., Nicolescu, N. D., Marinescu, A. R., Mocanu, A., Vilceanu, L., Paczeyka, R., Cut, T. G., & Lazureanu, V. E. (2021). Cutaneous Manifestations in SARS-CoV-2 Infection-A Series of Cases from the Largest Infectious Diseases Hospital in Western Romania. Healthcare (Basel, Switzerland), 9(7), 800. https://doi.org/10.3390/healthcare9070800

Lazar Neto, F., Mercadé-Besora, N., Raventós, B., Pérez-Crespo, L., Castro Junior, G., Ranzani, O. T., & Duarte-Salles, T. (2024). Effectiveness of COVID-19 vaccines against severe COVID-19 among patients with cancer in Catalonia, Spain. Nature Communications, 15(1). https://doi.org/10.1038/s41467-024-49285-y

Lázaro y de Mercado, P., Figueras Aloy, J., Doménech Martínez, E., Echániz Urcelay, I., Closa Monasterolo, R., Wood Wood, M. A., & Fitch Warner, K. (2006). [The efficiency (cost-effectiveness) of palivizumab as prophylaxis against respiratory syncytial virus infection in premature infants with a gestational age of 32-35 weeks in Spain]. Anales De Pediatria (Barcelona, Spain: 2003), 65(4). https://doi.org/10.1157/13092505

Lázaro Y De Mercado, P., Figueras, J., Domenech, E., Closa, R., Echániz, I., Wood, M. Á., & Fitch, K. (2007). Cost-effectiveness of palivizumab in preventing respiratory syncytial virus in premature infants and children with chronic lung disease in Spain. Pharmacoeconomics - Spanish Research Articles, 4(2). Embase. https://www.embase.com/search/results?subaction=viewrecord&id=L47262309&from=export

Lazuka, A., Arnal, C., Soyeux, E., Sampson, M., Lepeuple, A.-S., Deleuze, Y., Pouradier Duteil, S., & Lacroix, S. (2021). COVID-19 wastewater based epidemiology : Long-term monitoring of 10 WWTP in France reveals the importance of the sampling context. Water Science and Technology: A Journal of the International Association on Water Pollution Research, 84(8). https://doi.org/10.2166/wst.2021.418

Lazzeri, M., Duga, S., Azzolini, E., Fasulo, V., Buffi, N., Saita, A., Lughezzani, G., Paraboschi, E. M., Hurle, R., Nobili, A., Cecconi, M., Guazzoni, G., Casale, P., Asselta, R., & Humanitas COVID-19 Task Force, T. H. G. C.-19 T. F. (2022). Impact of chronic exposure to 5-alpha reductase inhibitors on the risk of hospitalization for COVID-19 : A case-control study in male population from two COVID-19 regional centers of Lombardy, Italy. Minerva Urology and Nephrology, 74(1). https://doi.org/10.23736/S2724-6051.20.04081-3

Lazzerini, M., Sforzi, I., Trapani, S., Biban, P., Silvagni, D., Villa, G., Tibaldi, J., Bertacca, L., Felici, E., Perricone, G., Parrino, R., Gioè, C., Lega, S., Conte, M., Marchetti, F., Magista, A., Berlese, P., Martelossi, S., Vaienti, F., … COVID-19 Italian Pediatric Study Network. (2021). Characteristics and risk factors for SARS-CoV-2 in children tested in the early phase of the pandemic: A cross-sectional study, Italy, 23 February to 24 May 2020. Euro Surveillance: Bulletin Europeen Sur Les Maladies Transmissibles = European Communicable Disease Bulletin, 26(14), 2001248. https://doi.org/10.2807/1560-7917.ES.2021.26.14.2001248

Le Bourg, E. (2021). Covid-19 : Were curfews in France associated with hospitalisations? Epidemiologic Methods, 10(s1). Embase. https://doi.org/10.1515/em-2021-0011

Le Vu, S., Jones, G., Anna, F., Rose, T., Richard, J.-B., Bernard-Stoecklin, S., Goyard, S., Demeret, C., Helynck, O., Escriou, N., Gransagne, M., Petres, S., Robin, C., Monnet, V., Perrin de Facci, L., Ungeheuer, M.-N., Léon, L., Guillois, Y., Filleul, L., … Noel, H. (2021). Prevalence of SARS-CoV-2 antibodies in France : Results from nationwide serological surveillance. Nature Communications, 12(1). https://doi.org/10.1038/s41467-021-23233-6

Leache, L., Librero, J., Gutiérrez-Valencia, M., de la Fuente, M., Lafita, J., & Gorricho, J. (2023). Impact of demographic, social and economic determinants in COVID-19 related outcomes in the middle-aged population of Navarre (Spain). JOURNAL OF PUBLIC HEALTH-HEIDELBERG. https://doi.org/10.1007/s10389-023-02124-8

Lecce, M., Biganzoli, G., Agnello, L., Belisario, I., Cicconi, G., D'Amico, M., De Giorgi, F., Ferilli, A., Ferraguzzi, G., Guzzardi, F., Lanzillotti, D., Lattanzio, R., Marrocu, C., Noto, M. E., Piccinelli, S., Sabatelli, N., Santisteban, S., Sharma, S., Tognaccini, L., & Castaldi, S. (2022). COVID-19 and Influenza Vaccination Campaign in a Research and University Hospital in Milan, Italy. International Journal of Environmental Research and Public Health, 19(11). https://doi.org/10.3390/ijerph19116500

Ledberg, A. (2021). Mortality of the COVID-19 Outbreak in Sweden in Relation to Previous Severe Disease Outbreaks. Frontiers in Public Health, 9. https://doi.org/10.3389/fpubh.2021.579948

Leducq, V., Jary, A., Bridier-Nahmias, A., Daniel, L., Zafilaza, K., Damond, F., Goldstein, V., Duval, A., Blanquart, F., Calvez, V., Descamps, D., Marcelin, A., & Visseaux, B. (2022). Nosocomial transmission clusters and lineage diversity characterized by SARS-CoV-2 genomes from two large hospitals in Paris, France, in 2020. SCIENTIFIC REPORTS, 12(1). https://doi.org/10.1038/s41598-022-05085-2

Lee, A., Davido, B., Beck, E., Demont, C., Joshi, K., Kohli, M., Maschio, M., Uhart, M., & El Mouaddin, N. (2024). Substantial reduction in the clinical and economic burden of disease following variant-adapted mRNA COVID-19 vaccines in immunocompromised patients in France. Human Vaccines & Immunotherapeutics, 20(1). https://doi.org/10.1080/21645515.2024.2423474

Lee, M. J., Snell, L. B., Douthwaite, S. T., Fidler, S., Fitzgerald, N., Goodwin, L., Hamzah, L., Kulasegaram, R., Lawrence, S., Lwanga, J., Marchant, R., Orkin, C., Palfreeman, A., Parthasarathi, P., Pareek, M., Ring, K., Sharaf, H., Shekarchi-Khanghahi, E., Simons, R., … Smith, C. (2022). Clinical outcomes of patients with and without HIV hospitalized with COVID-19 in England during the early stages of the pandemic: A matched retrospective multi-centre analysis (RECEDE-C19 study). HIV Medicine, 23(2), 121–133. https://doi.org/10.1111/hiv.13174

Leeman, D. S., Ma, T. S.-G., Pathiraja, M. M., Taylor, J. A., Adnan, T. Z., Baltas, I., Ioannou, A., Iyengar, S. R. S., Mearkle, R. A., Stockdale, T. J., Van Den Abbeele, K., & Balasegaram, S. (2022). Severe acute respiratory coronavirus virus 2 (SARS-CoV-2) nosocomial transmission dynamics, a retrospective cohort study of two healthcare-associated coronavirus disease 2019 (COVID-19) clusters in a district hospital in England during March and April 2020. Infection Control and Hospital Epidemiology, 43(11). https://doi.org/10.1017/ice.2021.483

Leeman, D., Flannagan, J., Chudasama, D., Dack, K., Anderson, C., Dabrera, G., & Lamagni, T. (2022). Effect of Returning University Students on COVID-19 Infections in England, 2020. Emerging Infectious Diseases, 28(7). https://doi.org/10.3201/eid2807.212332

Lefrancq, N., Paireau, J., Hozé, N., Courtejoie, N., Yazdanpanah, Y., Bouadma, L., Boëlle, P.-Y., Chereau, F., Salje, H., & Cauchemez, S. (2021). Evolution of outcomes for patients hospitalised during the first 9 months of the SARS-CoV-2 pandemic in France: A retrospective national surveillance data analysis. The Lancet Regional Health. Europe, 5, 100087. https://doi.org/10.1016/j.lanepe.2021.100087

Lehmann, M., Peeters, S., Streuter, M., Nawrocki, M., Kösters, K., & Kröger, K. (2023). [COVID 19—Hospital Admission in the First and Second Wave in Germany]. Deutsche Medizinische Wochenschrift (1946), 148(4). https://doi.org/10.1055/a-1951-0629

Lemaitre, M., Carrat, F., Rey, G., Miller, M., Simonsen, L., & Viboud, C. (2012). Mortality burden of the 2009 A/H1N1 influenza pandemic in France: Comparison to seasonal influenza and the A/H3N2 pandemic. PloS One, 7(9), e45051. https://doi.org/10.1371/journal.pone.0045051

Lemaitre, M., Fouad, F., Carrat, F., Crépey, P., Gaillat, J., Gavazzi, G., Launay, O., Mosnier, A., Levant, M.-C., & Uhart, M. (2022). Estimating the burden of influenza-related and associated hospitalizations and deaths in France : An eight-season data study, 2010-2018. Influenza and Other Respiratory Viruses, 16(4). https://doi.org/10.1111/irv.12962

Lemhöfer, C., Sturm, C., Loudovici-Krug, D., Best, N., & Gutenbrunner, C. (2021). The impact of Post-COVID-Syndrome on functioning—Results from a community survey in patients after mild and moderate SARS-CoV-2-infections in Germany. Journal of Occupational Medicine and Toxicology (London, England), 16(1). https://doi.org/10.1186/s12995-021-00337-9

Lendorf, M. E., Boisen, M. K., Kristensen, P. L., Løkkegaard, E. C. L., Krog, S. M., Brandi, L., Brinth, L. S., Nolsöe, R. L. M., Ryrsø, C., Eiken, P., Bestle, M. H., Jørgensen, I. M., Pedersen-Bjergaard, U., Lindegaard, B., Christensen, T. B., & Fischer, T. K. (2020). Characteristics and early outcomes of patients hospitalised for COVID-19 in North Zealand, Denmark. Danish Medical Journal, 67(9). http://www.ncbi.nlm.nih.gov/pubmed/32800073

Lenglet, A. D., Hernando, V., Rodrigo, P., Larrauri, A., Donado, J. D. M., & de Mateo, S. (2007). Impact of flu on hospital admissions during 4 flu seasons in Spain, 2000-2004. BMC Public Health, 7. https://doi.org/10.1186/1471-2458-7-197

Lenti, M. V., Borrelli de Andreis, F., Pellegrino, I., Klersy, C., Merli, S., Miceli, E., Aronico, N., Mengoli, C., Di Stefano, M., Cococcia, S., Santacroce, G., Soriano, S., Melazzini, F., Delliponti, M., Baldanti, F., Triarico, A., Corazza, G. R., Pinzani, M., Di Sabatino, A., & Internal Medicine Covid-19 Team. (2020). Impact of COVID-19

on liver function : Results from an internal medicine unit in Northern Italy. Internal and Emergency Medicine, 15(8). https://doi.org/10.1007/s11739-020-02425-w

León Gómez, I., Flores Segovia, V. M., Jiménez Jorge, S., Larrauri Cámara, A., Palmera Suárez, R., & Simón Soria, F. (2010). [Excess mortality in Spain during transmission of pandemic influenza in 2009]. Revista Espanola De Salud Publica, 84(5), 589–596. https://doi.org/10.1590/s1135-57272010000500010

León-Gómez, I., Delgado-Sanz, C., Jiménez-Jorge, S., Flores, V., Simón, F., Gómez-Barroso, D., Larrauri, A., & de Mateo Ontañón, S. (2015). [Excess mortality associated with influenza in Spain in winter 2012]. Gaceta Sanitaria, 29(4). https://doi.org/10.1016/j.gaceta.2015.01.011

León-Gómez, I., Mazagatos, C., Delgado-Sanz, C., Frías, L., Vega-Piris, L., Rojas-Benedicto, A., & Larrauri, A. (2021). The Impact of COVID-19 on Mortality in Spain : Monitoring Excess Mortality (MoMo) and the Surveillance of Confirmed COVID-19 Deaths. Viruses, 13(12). https://doi.org/10.3390/v13122423

Leszko, K., Sulik, A., & Żelazowska-Rutkowska, B. (2023). Seroprevalance of anti-SARS-CoV-2 antibodies in the IgG class in hospitalized children in the north-eastern part of Poland in 2021. Przeglad Epidemiologiczny, 77(2). https://doi.org/10.32394/pe.77.15

Leval, A., Hergens, M. P., Persson, K., & Örtqvist, Å. (2016). Real-time real-world analysis of seasonal influenza vaccine effectiveness : Method development and assessment of a population-based cohort in Stockholm County, Sweden, seasons 2011/12 to 2014/15. Euro Surveillance: Bulletin Europeen Sur Les Maladies Transmissibles = European Communicable Disease Bulletin, 21(43). https://doi.org/10.2807/1560-7917.ES.2016.21.43.30381

Levi, M., Cipriani, F., & Balzi, D. (2022). All-cause mortality during the influenza season 2019-2020 : Comparison with previous influenza seasons in the territory of the local health authority « Central Tuscany », Italy. Annali Di Igiene: Medicina Preventiva E Di Comunita, 34(2). https://doi.org/10.7416/ai.2021.2465

Levi, M., Cipriani, F., Romeo, G., & Balzi, D. (2021). Analysis of the excess mortality and factors associated with deaths from COVID-19 versus other causes in Central Tuscany (Italy) in 2020. Epidemiologia E Prevenzione, 45(6). https://doi.org/10.19191/EP21.6.123

Liang, C., Begier, E., Hagel, S., Ankert, J., Wang, L., Schwarz, C., Bayer, L. J., von Eiff, C., Liu, Q., Southern, J., Vietri, J., Uppal, S., Gessner, B. D., Theilacker, C., & Pletz, M. (2024). Estimated Incidence of Respiratory Syncytial Virus (RSV)-related Hospitalizations for Acute Respiratory Infections (ARIs), including Community Acquired Pneumonia (CAP), in Adults in Germany. medRxiv, (Liang C.; Wang L.) Pfizer Inc, New York, United States. https://doi.org/10.1101/2024.06.09.24308507

Liang, C., Polkowska-Kramek, A., Lade, C., Bayer, L. J., Bruyndonckx, R., Huebbe, B., Ewnetu, W. B., Peerawaranun, P., Casas, M., Mai Phuong Tran, T., Brestrich, G., von Eiff, C., Gessner, B. D., Begier, E., & Rohde, G. (2024). Estimated incidence rate of specific cardiovascular and respiratory hospitalizations attributable to Respiratory Syncytial Virus among adults in Germany between 2015 and 2019. medRxiv, (Liang C., caihua.liang@pfizer.com; Gessner B.D.) Pfizer Inc, New York, United States. https://doi.org/10.1101/2024.07.19.24310503

Liang, C., Polkowska-Kramek, A., Lade, C., Bayer, L. J., Bruyndonckx, R., Huebbe, B., Ewnetu, W. B., Peerawaranun, P., Casas, M., Tran, T. M. P., Brestrich, G., von Eiff, C., Gessner, B. D., Begier, E., & Rohde, G. (2025). Estimated Incidence Rate of Specific Types of Cardiovascular and Respiratory Hospitalizations Attributable to Respiratory Syncytial Virus Among Adults in Germany Between 2015 and 2019. Influenza and Other Respiratory Viruses, 19(5), e70097. https://doi.org/10.1111/irv.70097

Liard, R., Souty, C., Guerrisi, C., Colizza, V., Hanslik, T., Kengne Kuetche, C., Turbelin, C., Blanchon, T., & Debin, M. (2022). Seasonal influenza vaccination in pharmacy in France : Description and determinants of the vaccinated at-risk population using this service, 1 year after implementation. The International Journal of Pharmacy Practice, 30(3). https://doi.org/10.1093/ijpp/riac007

Lin, T., Zhao, Z., Yang, Z., Li, B., Wei, C., Li, F., Jiang, Y., Liu, D., Yang, Z., Sha, F., & Tang, J. (2022). Hospital Strain and COVID-19 Fatality—England, April 2020-March 2022. China CDC Weekly, 4(52). https://doi.org/10.46234/ccdcw2022.236

Lina, B., Holm, M. V., & Szucs, T. D. (2008). [Evolution of influenza vaccination coverage in France from 2001 to 2006]. Medecine Et Maladies Infectieuses, 38(3). https://doi.org/10.1016/j.medmal.2007.11.014

Linares, C., Belda, F., López-Bueno, J. A., Luna, M. Y., Sánchez-Martínez, G., Hervella, B., Culqui, D., & Díaz, J. (2021). Short-term associations of air pollution and meteorological variables on the incidence and severity of COVID-19 in Madrid (Spain) : A time series study. Environmental Sciences Europe, 33(1). https://doi.org/10.1186/s12302-021-00548-1

Linares, C., Culqui, D., Belda, F., López-Bueno, J. A., Luna, Y., Sánchez-Martínez, G., Hervella, B., & Díaz, J. (2021). Impact of environmental factors and Sahara dust intrusions on incidence and severity of COVID-19 disease in Spain. Effect in the first and second pandemic waves. Environmental Science and Pollution Research International, 28(37), 51948–51960. https://doi.org/10.1007/s11356-021-14228-3

Linder, R., Peltner, J., Astvatsatourov, A., Gomm, W., & Haenisch, B. (2025). COVID-19 in the years 2020 to 2022 in Germany : Effects of comorbidities and co-medications based on a large-scale database analysis. BMC Public Health, 25(1). https://doi.org/10.1186/s12889-024-21110-7

Lindner-Pawłowicz, K., Mydlikowska-Śmigórska, A., Łampika, K., & Sobieszczańska, M. (2021). COVID-19 Vaccination Acceptance among Healthcare Workers and General Population at the Very Beginning of the National Vaccination Program in Poland : A Cross-Sectional, Exploratory Study. Vaccines, 10(1). https://doi.org/10.3390/vaccines10010066

Liotti, F. M., Marchetti, S., D'Onghia, S., Sanguinetti, M., Santangelo, R., & Posteraro, B. (2025). Detection of Respiratory Viruses Other Than SARS-CoV-2 in a Large Hospital Laboratory in Rome, Italy, During the Seasons 2016-2017 to 2022-2023. Influenza and Other Respiratory Viruses, 19(2), e70079. https://doi.org/10.1111/irv.70079

Lippiello, E., Petrillo, G., Baccari, S., & de Arcangelis, L. (2023). Estimating generation time of SARS-CoV-2 variants in Italy from the daily incidence rate. Scientific Reports, 13(1). https://doi.org/10.1038/s41598-023-38327-y

Lippold, D., Kergassner, A., Burkhardt, C., Kergassner, M., Loos, J., Nistler, S., Steinmann, P., Budday, D., & Budday, S. (2022). Spatiotemporal modeling of first and second wave outbreak dynamics of COVID-19 in Germany. BIOMECHANICS AND MODELING IN MECHANOBIOLOGY, 21(1). https://doi.org/10.1007/s10237-021-01520-x

Lipponen, A., Kolehmainen, A., Oikarinen, S., Hokajärvi, A.-M., Lehto, K.-M., Heikinheimo, A., Halkilahti, J., Juutinen, A., Luomala, O., Smura, T., Liitsola, K., Blomqvist, S., Savolainen-Kopra, C., Pitkänen, T., & WastPan Study Group. (2024). Detection of SARS-COV-2 variants and their proportions in wastewater samples using next-generation sequencing in Finland. Scientific Reports, 14(1). https://doi.org/10.1038/s41598-024-58113-8

Liu, P.-Y., He, S., Rong, L.-B., & Tang, S.-Y. (2020). The effect of control measures on COVID-19 transmission in Italy : Comparison with Guangdong province in China. Infectious Diseases of Poverty, 9(1). https://doi.org/10.1186/s40249-020-00730-2

Liu, Y., Tang, J., & Lam, T. (2021). Transmission dynamics of the COVID-19 epidemic in England. INTERNATIONAL JOURNAL OF INFECTIOUS DISEASES, 104. https://doi.org/10.1016/j.ijid.2020.12.055

Liviero, F., Volpin, A., Furlan, P., Cocchio, S., Baldo, V., Pavanello, S., Moretto, A., Gobba, F., Modenese, A., Mauro, M., Larese Filon, F., Carta, A., Monaco, M. G. L., Spiteri, G., Porru, S., & Scapellato, M. L. (2025). Risk of SARS-CoV-2 Reinfections Among Healthcare Workers of Four Large University Hospitals in Northern Italy: Results of an Online Survey Within the ORCHESTRA Project. Vaccines, 13(8), 815. https://doi.org/10.3390/vaccines13080815

Ljung, R., Feychting, M., Burström, B., & Möller, J. (2022). Differences by region of birth in SARS-CoV-2 vaccine coverage and positive SARS-CoV-2 test among 400 000 healthcare workers and the general population in Sweden. Vaccine, 40(21). https://doi.org/10.1016/j.vaccine.2022.04.014

Llorca, J., Gómez-Acebo, I., Alonso-Molero, J., Delgado-Rodríguez, M., & Dierssen-Sotos, T. (2025). Direct and indirect burden of COVID-19 on mortality in Spain (2020 to 2022). BMC Public Health, 25(1), 1885. https://doi.org/10.1186/s12889-025-23077-5

Lo Presti, A., Di Martino, A., Ambrosio, L., De Sabato, L., Knijn, A., Vaccari, G., Di Bartolo, I., Morabito, S., Terregino, C., Fusaro, A., Monne, I., Giussani, E., Tramuto, F., Maida, C. M., Mazzucco, W., Costantino, C., Rueca, M., Giombini, E., Gruber, C. E. M., … On Behalf Of The Italian Genomic Laboratory Network, null. (2023). Tracking the Selective Pressure Profile and Gene Flow of SARS-CoV-2 Delta Variant in Italy from April to October 2021 and Frequencies of Key Mutations from Three Representative Italian Regions. Microorganisms, 11(11). https://doi.org/10.3390/microorganisms11112644

Lo Presti, A., Di Martino, A., Faggioni, G., Giordani, F., Fillo, S., Anselmo, A., Fain, V. V., Fortunato, A., Petralito, G., Molinari, F., Palomba, S., De Santis, R., Fiore, S., Fabiani, C., Di Mario, G., Facchini, M., Calzoletti, L., Lista, F., Rezza, G., & Stefanelli, P. (2022). Analysis of Genomic Characteristics of SARS-CoV-2 in Italy, 29 January to 27 March 2020. Viruses, 14(3). https://doi.org/10.3390/v14030472

Lobiuc, A., Dimian, M., Gheorghita, R., Sturdza, O. A. C., & Covasa, M. (2021). Introduction and Characteristics of SARS-CoV-2 in North-East of Romania During the First COVID-19 Outbreak. Frontiers in Microbiology, 12, 654417. https://doi.org/10.3389/fmicb.2021.654417

Loché Fernández-Ahúja, J. M., & Fernández Martínez, J. L. (2021). Effects of climate variables on the COVID-19 outbreak in Spain. International Journal of Hygiene and Environmental Health, 234. https://doi.org/10.1016/j.ijheh.2021.113723

Loconsole, D., Centrone, F., Morcavallo, C., Campanella, S., Accogli, M., Sallustio, A., Peccarisi, D., Stufano, A., Lovreglio, P., & Chironna, M. (2021). Changing Features of COVID-19 : Characteristics of Infections with the SARS-CoV-2 Delta (B.1.617.2) and Alpha (B.1.1.7) Variants in Southern Italy. Vaccines, 9(11). https://doi.org/10.3390/vaccines9111354

Loconsole, D., Centrone, F., Morcavallo, C., Campanella, S., Sallustio, A., Accogli, M., Fortunato, F., Parisi, A., & Chironna, M. (2021). Rapid Spread of the SARS-CoV-2 Variant of Concern 202012/01 in Southern Italy (December 2020-March 2021). International Journal of Environmental Research and Public Health, 18(9). https://doi.org/10.3390/ijerph18094766

Loconsole, D., Centrone, F., Sallustio, A., Accogli, M., Casulli, D., Sacco, D., Zagaria, R., Morcavallo, C., & Chironna, M. (2022). Characteristics of the First 284 Patients Infected with the SARS-CoV-2 Omicron BA.2 Subvariant at a Single Center in the Apulia Region of Italy, January-March 2022. Vaccines, 10(5). https://doi.org/10.3390/vaccines10050674

Loconsole, D., De Robertis, A. L., Morea, A., Casulli, D., Mallamaci, R., Baldacci, S., Centrone, F., Bruno, V., Quarto, M., Accogli, M., & Chironna, M. (2019). High Public-Health Impact in an Influenza-B-Mismatch Season in Southern Italy, 2017-2018. BioMed Research International, 2019. https://doi.org/10.1155/2019/4643260

Lodigiani, C., Iapichino, G., Carenzo, L., Cecconi, M., Ferrazzi, P., Sebastian, T., Kucher, N., Studt, J.-D., Sacco, C., Bertuzzi, A., Sandri, M. T., Barco, S., & Humanitas COVID-19 Task Force. (2020). Venous and arterial thromboembolic complications in COVID-19 patients admitted to an academic hospital in Milan, Italy. Thrombosis Research, 191. https://doi.org/10.1016/j.thromres.2020.04.024

Loenenbach, A., Lehfeld, A.-S., Puetz, P., Biere, B., Abunijela, S., Buda, S., Diercke, M., Dürrwald, R., Greiner, T., Haas, W., Helmrich, M., Prahm, K., Schumacher, J., Wedde, M., & Buchholz, U. (2024). Participatory, Virologic, and Wastewater Surveillance Data to Assess Underestimation of COVID-19 Incidence, Germany, 2020-2024. Emerging Infectious Diseases, 30(9). https://doi.org/10.3201/eid3009.240640

Loghin, I. I., Mihai, I. F., Roşu, M. F., Diaconu, I. E., Vâţă, A., Popa, R., & Luca, M. C. (2022). Characteristics and Trends of COVID-19 Infection in a Tertiary Hospital in Romania: A Retrospective Study. Journal of Personalized Medicine, 12(11), 1928. https://doi.org/10.3390/jpm12111928

Lohiniva, A.-L., Pensola, A., Hyökki, S., Sivelä, J., Härmä, V., & Tammi, T. (2023). Identifying factors influencing COVID-19 vaccine uptake in Finland—A qualitative study using social media data. Frontiers in Public Health, 11. https://doi.org/10.3389/fpubh.2023.1138800

Loiacono, M. M., Mahmud, S. M., Chit, A., van Aalst, R., Kwong, J. C., Mitsakakis, N., Skinner, L., Thommes, E., Bricout, H., & Grootendorst, P. (2020). Patient and practice level factors associated with seasonal influenza vaccine uptake among at-risk adults in England, 2011 to 2016 : An age-stratified retrospective cohort study. Vaccine: X, 4. https://doi.org/10.1016/j.jvacx.2020.100054

Lois, M., Polo, D., Pérez del Molino, M. L., Coira, A., Aguilera, A., & Romalde, J. L. (2025). Monitoring the Emergence of SARS-CoV-2 VOCs in Wastewater and Clinical Samples—A One-Year Study in Santiago de Compostela (Spain). Viruses, 17(4). https://doi.org/10.3390/v17040489

Lombardi, A., Consonni, D., Carugno, M., Bozzi, G., Mangioni, D., Muscatello, A., Castelli, V., Palomba, E., Cantù, A. P., Ceriotti, F., Tiso, B., Pesatori, A. C., Riboldi, L., Bandera, A., Lunghi, G., & Gori, A. (2020). Characteristics of 1573 healthcare workers who underwent nasopharyngeal swab testing for SARS-CoV-2 in Milan, Lombardy, Italy. Clinical Microbiology and Infection: The Official Publication of the European Society of Clinical Microbiology and Infectious Diseases, 26(10). https://doi.org/10.1016/j.cmi.2020.06.013

Lombardi, A., Mangioni, D., Consonni, D., Cariani, L., Bono, P., Cantù, A. P., Tiso, B., Carugno, M., Muscatello, A., Lunghi, G., Pesatori, A. C., Riboldi, L., Ceriotti, F., Bandera, A., & Gori, A. (2021). Seroprevalence of anti-SARS-CoV-2 IgG among healthcare workers of a large university hospital in Milan, Lombardy, Italy : A cross-sectional study. BMJ Open, 11(2). https://doi.org/10.1136/bmjopen-2020-047216

Lombardi, A., Villa, S., Colaneri, M., Scaglione, G., Bai, F., Varisco, B., Bono, V., Vena, A., Dentone, C., Russo, C., Tettamanti, M., Renisi, G., Viero, G., Azzarà, C., Mantero, M., Peyvandi, F., Bassetti, M., Marchetti, G., Muscatello, A., … “COVID 19 NETWORK”. (2024). Clinical characteristics and outcomes of vaccinated patients hospitalised with SARS-CoV-2 breakthrough infection : Multi-IPV, a multicentre study in Northern Italy. Journal of Infection and Public Health, 17(3). https://doi.org/10.1016/j.jiph.2023.12.026

Longchamps, C., Ducarroz, S., Crouzet, L., Vignier, N., Pourtau, L., Allaire, C., Colleville, A. C., El Aarbaoui, T., Melchior, M., & ECHO study group. (2021). COVID-19 vaccine hesitancy among persons living in homeless shelters in France. Vaccine, 39(25). https://doi.org/10.1016/j.vaccine.2021.05.012

Lopez Bernal, J., Andrews, N., Gower, C., Robertson, C., Stowe, J., Tessier, E., Simmons, R., Cottrell, S., Roberts, R., O’Doherty, M., Brown, K., Cameron, C., Stockton, D., McMenamin, J., & Ramsay, M. (2021). Effectiveness of the Pfizer-BioNTech and Oxford-AstraZeneca vaccines on covid-19 related symptoms, hospital admissions, and mortality in older adults in England : Test negative case-control study. BMJ (Clinical Research Ed.), 373. https://doi.org/10.1136/bmj.n1088

López, M. G., Chiner-Oms, Á., García de Viedma, D., Ruiz-Rodriguez, P., Bracho, M. A., Cancino-Muñoz, I., D’Auria, G., de Marco, G., García-González, N., Goig, G. A., Gómez-Navarro, I., Jiménez-Serrano, S., Martinez-Priego, L., Ruiz-Hueso, P., Ruiz-Roldán, L., Torres-Puente, M., Alberola, J., Albert, E., Aranzamendi Zaldumbide, M., … Comas, I. (2021). The first wave of the COVID-19 epidemic in Spain was associated with early introductions and fast spread of a dominating genetic variant. Nature Genetics, 53(10). https://doi.org/10.1038/s41588-021-00936-6

López-Cuadrado, T., de Mateo, S., Jiménez-Jorge, S., Savulescu, C., & Larrauri, A. (2012). Influenza-related mortality in Spain, 1999-2005. Gaceta Sanitaria, 26(4). https://doi.org/10.1016/j.gaceta.2011.09.033

López-de-Andrés, A., Carrasco-Garrido, P., Hernández-Barrera, V., de Miguel, A., & Jiménez-García, R. (2008). Coverages and factors associated with influenza vaccination among subjects with chronic respiratory diseases in Spain. EUROPEAN JOURNAL OF PUBLIC HEALTH, 18(2). https://doi.org/10.1093/eurpub/ckm093

López-García, E., Fernández-Arribas, S., Pérez-Rubio, A., Eiros-Bouza, J. M., Castrodeza-Sanz, J. J., & Grupo de Vigilancia de Castilla y León. (2010). [Influenza (H1N1) 2009 in Castilla y Leon, Spain : Hospitalized case studies and match with the protocols of action developed]. Revista Espanola De Salud Publica, 84(5). https://doi.org/10.1590/s1135-57272010000500018

López-Herrero, R., Sánchez-de Prada, L., Tamayo-Velasco, A., Lorenzo-López, M., Gómez-Pesquera, E., Sánchez-Quirós, B., de la Varga-Martínez, O., Gómez-Sánchez, E., Resino, S., Tamayo, E., & Álvaro-Meca, A. (2023). Epidemiology of bacterial co-infections and risk factors in COVID-19-hospitalized patients in Spain : A nationwide study. European Journal of Public Health, 33(4). https://doi.org/10.1093/eurpub/ckad060

López-Lacort, M., Muñoz-Quiles, C., Mira-Iglesias, A., López-Labrador, F. X., Mengual-Chuliá, B., Fernández-García, C., Carballido-Fernández, M., Pineda-Caplliure, A., Mollar-Maseres, J., Shalabi Benavent, M., Sanz-Herrero, F., Zornoza-Moreno, M., Pérez-Martín, J. J., Alfayate-Miguelez, S., Pérez Crespo, R., Bastida Sánchez, E., Menasalvas-Ruiz, A. I., Téllez-González, M. C., Esquiva Soto, S., … Orrico-Sánchez, A. (2024). Early estimates of nirsevimab immunoprophylaxis effectiveness against hospital admission for respiratory syncytial virus lower respiratory tract infections in infants, Spain, October 2023 to January 2024. Euro Surveillance: Bulletin Europeen Sur Les Maladies Transmissibles = European Communicable Disease Bulletin, 29(6). https://doi.org/10.2807/1560-7917.ES.2024.29.6.2400046

López-Sánchez, I., Perramon, A., Soriano-Arandes, A., Prats, C., Duarte-Salles, T., Raventós, B., & Roel, E. (2023). Socioeconomic inequalities in COVID-19 infection and vaccine uptake among children and adolescents in Catalonia, Spain. medRxiv, (López-Sánchez I.; Duarte-Salles T., tduarte@idiapjgol.info; Raventós B.; Roel E.) Fundació Institut Universitari per a la Recerca a l'Atenció Primària de Salut Jordi Gol i Gurina (IDIAPJGol), Barcelona, Spain. https://doi.org/10.1101/2023.10.17.23297134

López-Zambrano, M. A., Pita, C. C., Escribano, M. F., Galán Meléndez, I. M., Cebrián, M. G., Arroyo, J. J. G., Huerta, C., Cuadrado, L. M., Ruiperez, C. M., Núñez, C., Zapata, A. P., de la Pinta, M. L. R., Uriz, M. A. S., Conejo, I. S.-A., Gomila, C. M., Carbajo, M. D. L., & Gómez, A. S. (2023). Factors associated to influenza vaccination among hospital's healthcare workers in the Autonomous Community of Madrid, Spain 2021-2022. Vaccine, 41(45). https://doi.org/10.1016/j.vaccine.2023.09.047

Loreggia, A., Passarelli, A., & Pini, M. S. (2022). The Influence of Environmental Factors on the Spread of COVID-19 in Italy. Procedia Computer Science, 207. https://doi.org/10.1016/j.procs.2022.09.112

Lorenzon, A., Palandri, L., Uguzzoni, F., Cristofor, C. D., Lozza, F., Rizzi, C., Poluzzi, R., Bertoli, P., Zerzer, F., & Righi, E. (2024). An Ecological Study Relating the SARS-CoV-2 Epidemiology with Health-Related, Socio-Demographic, and Geographical Characteristics in South Tyrol (Italy). International Journal of Environmental Research and Public Health, 21(12). https://doi.org/10.3390/ijerph21121604

Lorettu, L., Mastrangelo, G., Stepien, J., Grabowski, J., Meloni, R., Piu, D., Michalski, T., Waszak, P. M., Bellizzi, S., & Cegolon, L. (2021). Attitudes and Perceptions of Health Protection Measures Against the Spread of COVID-19 in Italy and Poland. Frontiers in Psychology, 12. https://doi.org/10.3389/fpsyg.2021.805790

Lorini, C., Lastrucci, V., Zanella, B., Gori, E., Chiesi, F., Bechini, A., Boccalini, S., Del Riccio, M., Moscadelli, A., Puggelli, F., Berti, R., Bonanni, P., & Bonaccorsi, G. (2022). Predictors of Influenza Vaccination Uptake and the Role of Health Literacy among Health and Social Care Volunteers in the Province of Prato (Italy). International Journal of Environmental Research and Public Health, 19(11). https://doi.org/10.3390/ijerph19116688

Loubet, P., Fernandes, J., de Pouvourville, G., Sosnowiez, K., Elong, A., Guilmet, C., Omichessan, H., Bureau, I., Fagnani, F., Emery, C., & Abou Chakra, C. N. (2024). Respiratory syncytial virus-related hospital stays in adults in France from 2012 to 2021 : A national hospital database study. Journal of Clinical Virology: The Official Publication of the Pan American Society for Clinical Virology, 171. https://doi.org/10.1016/j.jcv.2023.105635

Loubet, P., Guitton, S., Rolland, S., Lefrancois, L. H., Nguyen, L. B. L., Vanhems, P., Laine, F., Galtier, F., Duval, X., Lina, B., Valette, M., Lagathu, G., Foulongne, V., Houhou-Fidhou, N., L'Honneur, A. S., Carrat, F., Meyer, L., Durier, C., & Launay, O. (2025). Characteristics of Human Metapneumovirus Infection Compared to Respiratory Syncytial Virus and Influenza Infections in Adults Hospitalized for Influenza-Like Illness in France, 2012-2022. The Journal of Infectious Diseases, 232, S93–S100. https://doi.org/10.1093/infdis/jiaf082

Loubet, P., Lenzi, N., Valette, M., Foulongne, V., Krivine, A., Houhou, N., Lagathu, G., Rogez, S., Alain, S., Duval, X., Galtier, F., Postil, D., Tattevin, P., Vanhems, P., Carrat, F., Lina, B., Launay, O., & FLUVAC Study Group. (2017). Clinical characteristics and outcome of respiratory syncytial virus infection among adults hospitalized with influenza-like illness in France. Clinical Microbiology and Infection: The Official Publication

of the European Society of Clinical Microbiology and Infectious Diseases, 23(4). https://doi.org/10.1016/j.cmi.2016.11.014

Loubet, P., Samih-Lenzi, N., Galtier, F., Vanhems, P., Loulergue, P., Duval, X., Jouneau, S., Postil, D., Rogez, S., Valette, M., Merle, C., Régis, C., Costa, Y., Lesieur, Z., Tattevin, P., Lina, B., Carrat, F., Launay, O., & FLUVAC Study Group. (2016). Factors associated with poor outcomes among adults hospitalized for influenza in France : A three-year prospective multicenter study. Journal of Clinical Virology: The Official Publication of the Pan American Society for Clinical Virology, 79. https://doi.org/10.1016/j.jcv.2016.04.005

Loubet, P., Samih-Lenzi, N., Valette, M., Duval, X., Canoui, E., Galtier, F., Postil, D., Jouneau, S., Vanhems, P., Carrat, F., Lina, B., & Launay, O. (2016). Respiratory syncytial virus infection in adults hospitalized with influenza-like illness in France. Open Forum Infectious Diseases, 3((Loubet P.) Inserm, CIC 1417, Paris, France). Embase. https://doi.org/10.1093/ofid/ofw172.511

Loubet, P., Verger, P., Abitbol, V., Peyrin-Biroulet, L., & Launay, O. (2018). Pneumococcal and influenza vaccine uptake in adults with inflammatory bowel disease in France : Results from a web-based study. Digestive and Liver Disease: Official Journal of the Italian Society of Gastroenterology and the Italian Association for the Study of the Liver, 50(6). https://doi.org/10.1016/j.dld.2017.12.027

Lozano, M., Iftimi, A., Briz-Redon, A., Peiró, J., Manyes, L., Otero, M., Ballester, M., de Las Marinas, M. D., Catalá, J. C., de Andrés, J., & Romero, C. (2022). Clinical characteristics of COVID-19 hospitalized patients associated with mortality : A cohort study in Spain. Infectious Medicine, 1(2). https://doi.org/10.1016/j.imj.2022.04.002

Lu, M., Zheng, X., Jia, W., & Tian, C. (2023). Analysis and prediction of improved SEIR transmission dynamics model : Taking the second outbreak of COVID-19 in Italy as an example. FRONTIERS IN PUBLIC HEALTH, 11. https://doi.org/10.3389/fpubh.2023.1223039

Luca, G. D., Kerckhove, K. V., Coletti, P., Poletto, C., Bossuyt, N., Hens, N., & Colizza, V. (2018). The impact of regular school closure on seasonal influenza epidemics : A data-driven spatial transmission model for Belgium. BMC Infectious Diseases, 18(1). https://doi.org/10.1186/s12879-017-2934-3

Lucerón, C. O., Sánchez, A. L., de la Fuente Sánchez, M., & Galindo Moreno, E. (2022). Influenza vaccine hesitancy among healthcare workers in Spain : Results of a survey. Vacunas, 23(3). Embase. https://doi.org/10.1016/j.vacun.2022.01.005

Ludwig, M., Jacob, J., Basedow, F., Andersohn, F., & Walker, J. (2021). Clinical outcomes and characteristics of patients hospitalized for Influenza or COVID-19 in Germany. International Journal of Infectious Diseases: IJID: Official Publication of the International Society for Infectious Diseases, 103. https://doi.org/10.1016/j.ijid.2020.11.204

Lundberg-Morris, L., Leach, S., Xu, Y., Martikainen, J., Santosa, A., Gisslén, M., Li, H., Nyberg, F., & Bygdell, M. (2023). Covid-19 vaccine effectiveness against post-covid-19 condition among 589 722 individuals in Sweden : Population based cohort study. BMJ (Clinical Research Ed.), 383. https://doi.org/10.1136/bmj-2023-076990

Lundkvist, Å., Hanson, S., & Olsen, B. (2020). Pronounced difference in Covid-19 antibody prevalence indicates cluster transmission in Stockholm, Sweden. Infection Ecology & Epidemiology, 10(1). https://doi.org/10.1080/20008686.2020.1806505

Lunelli, A., Rizzo, C., Puzelli, S., Bella, A., Montomoli, E., Rota, M. C., Donatelli, I., & Pugliese, A. (2013). Understanding the dynamics of seasonal influenza in Italy : Incidence, transmissibility and population susceptibility in a 9-year period. Influenza and Other Respiratory Viruses, 7(3). https://doi.org/10.1111/j.1750-2659.2012.00388.x

Łuniewska, K., Szymański, K., Hallmann-Szelińska, E., Kowalczyk, D., Sałamatin, R., Masny, A., & Brydak, L. B. (2019). Evaluation of the 2017/18 Influenza Epidemic Season in Poland Based on the SENTINEL Surveillance System. Advances in Experimental Medicine and Biology, 1222. https://doi.org/10.1007/5584_2019_424

Luong Ngyen, L. B., Bauer, R., Lesieur, Z., Galtier, F., Duval, X., Vanhems, P., Lainé, F., Tattevin, P., Durier, C., Launay, O., & FLUVAC Study group. (2022). Vaccine effectiveness against COVID-19 hospitalization in adults in France : A test negative case control study. Infectious Diseases Now, 52(1). https://doi.org/10.1016/j.idnow.2021.12.002

Lupi, S. M., Todaro, C., Camassa, D., Rizzo, S., Storelli, S., & Rodriguez Y Baena, R. (2022). Excess Mortality among Physicians and Dentists during COVID-19 in Italy : A Cross-Sectional Study Related to a High-Risk Territory. Healthcare (Basel, Switzerland), 10(9). https://doi.org/10.3390/healthcare10091684

Lupi, S., Stefanati, A., Califano, A., DE Togni, A., Cosenza, G., & Gabutti, G. (2021). Trends in influenza coverage rates in five consecutive immunisation seasons in the Local Health Unit of Ferrara (North Italy). Journal of Preventive Medicine and Hygiene, 62(3). https://doi.org/10.15167/2421-4248/jpmh2021.62.3.1657

Lyytikainen, O., Kuusi, M., Snellman, M., Virtanen, M., Eskola, J., Ronkko, E., Ikonen, N., Julkunen, I., Ziegler, T., & Ruutu, P. (2011). Surveillance of influenza in Finland during the 2009 pandemic, 10 May 2009 to 8 March 2010. Euro Surveillance: Bulletin Europeen Sur Les Maladies Transmissibles = European Communicable Disease Bulletin, 16(27), 19908.

Ma, T., Englund, H., Bjelkmar, P., Wallensten, A., & Hulth, A. (2015). Syndromic surveillance of influenza activity in Sweden : An evaluation of three tools. Epidemiology and Infection, 143(11). https://doi.org/10.1017/S0950268814003240

Maas, A. F. G., Wyers, C., Dielis, A., Barten, D. G., van Kampen, V. E. M., van der Krieken, T. E., de Kruif, M., Simsek, S., Spaetgens, B., van Haaps, T., Appelman, B., Gritters, N. C., Doornbos, S., Moeniralam, H. S., Noordzij, P. G., Reidinga, A., Douma, R. A., Nossent, E. J., Beudel, M., … van Osch, F. H. M. (2024). The Incidence of Pulmonary Embolism in Hospitalized Non-ICU Patients with COVID-19 during the First Wave : A Multicenter Retrospective Cohort Study in the Netherlands. Journal of Vascular Research, 61(3). https://doi.org/10.1159/000538312

Maccari, L. (2023). On the Performance and Effectiveness of Digital Contact Tracing in the Second Wave of COVID-19 in Italy. IEEE TRANSACTIONS ON COMPUTATIONAL SOCIAL SYSTEMS, 10(1), 108–119. https://doi.org/10.1109/TCSS.2022.3143369

Machado, A., Mazagatos, C., Dijkstra, F., Kislaya, I., Gherasim, A., McDonald, S. A., Kissling, E., Valenciano, M., Meijer, A., Hooiveld, M., Nunes, B., & Larrauri, A. (2019). Impact of influenza vaccination programmes among the elderly population on primary care, Portugal, Spain and the Netherlands : 2015/16 to 2017/18 influenza seasons. Euro Surveillance: Bulletin Europeen Sur Les Maladies Transmissibles = European Communicable Disease Bulletin, 24(45). https://doi.org/10.2807/1560-7917.ES.2019.24.45.1900268

Machała, M. K., Zycińska, K., & Brydak, L. B. (2006). Virological and epidemiological surveillance of influenza SENTINEL in Poland—Function in the first two influenza epidemic seasons 2004/2005 and 2005/2006. Family Medicine and Primary Care Review, 8(3). Embase. https://www.embase.com/search/results?subaction=viewrecord&id=L44850106&from=export

Mack, D., Gärtner, B. C., Rössler, A., Kimpel, J., Donde, K., Harzer, O., Krutsch, W., von Laer, D., & Meyer, T. (2021). Prevalence of SARS-CoV-2 IgG antibodies in a large prospective cohort study of elite football players in Germany (May-June 2020) : Implications for a testing protocol in asymptomatic individuals and estimation of the rate of undetected cases. Clinical Microbiology and Infection: The Official Publication of the European Society of Clinical Microbiology and Infectious Diseases, 27(3). https://doi.org/10.1016/j.cmi.2020.11.033

Maechler, F., Gertler, M., Hermes, J., van Loon, W., Schwab, F., Piening, B., Rojansky, S., Hommes, F., Kausch, F., Lindner, A. K., Burock, S., Rössig, H., Hartmann, C., Kirchberger, V., Thieme, A., Behnke, M., Gastmeier, P., Mockenhaupt, F. P., & Seybold, J. (2020). Epidemiological and clinical characteristics of SARS-CoV-2 infections at a testing site in Berlin, Germany, March and April 2020-a cross-sectional study. Clinical Microbiology and Infection: The Official Publication of the European Society of Clinical Microbiology and Infectious Diseases, 26(12). https://doi.org/10.1016/j.cmi.2020.08.017

Maertens, K., Braeckman, T., Top, G., Van Damme, P., & Leuridan, E. (2016). Maternal pertussis and influenza immunization coverage and attitude of health care workers towards these recommendations in Flanders, Belgium. Vaccine, 34(47). https://doi.org/10.1016/j.vaccine.2016.09.055

Maestre-Muñiz, M. M., Arias, Á., Arias-González, L., Angulo-Lara, B., & Lucendo, A. J. (2021). Prognostic Factors at Admission for In-Hospital Mortality from COVID-19 Infection in an Older Rural Population in Central Spain. Journal of Clinical Medicine, 10(2). https://doi.org/10.3390/jcm10020318

Mageau, A., Simard, J. F., Svenungsson, E., & Arkema, E. V. (2024). Anti-SARS-CoV-2 mRNA vaccination among patients living with SLE in Sweden : Coverage and clinical effectiveness. Lupus, 33(11). https://doi.org/10.1177/09612033241273052

Magee, L. A., Molteni, E., Bowyer, V., Bone, J. N., Boulding, H., Khalil, A., Mistry, H. D., Poston, L., Silverio, S. A., Wolfe, I., Duncan, E. L., von Dadelszen, P., & RESILIENT Study Group. (2023). National surveillance data analysis of COVID-19 vaccine uptake in England by women of reproductive age. Nature Communications, 14(1). https://doi.org/10.1038/s41467-023-36125-8

Magnani, C., Azzolina, D., Gallo, E., Ferrante, D., & Gregori, D. (2020). How Large Was the Mortality Increase Directly and Indirectly Caused by the COVID-19 Epidemic ? An Analysis on All-Causes Mortality Data in Italy. International Journal of Environmental Research and Public Health, 17(10). https://doi.org/10.3390/ijerph17103452

Magro, P., Degli Antoni, M., Formenti, B., Viola, F., Castelli, F., Amadasi, S., & Quiros-Roldan, E. (2023). Characteristics of the population with mild COVID-19 symptoms eligible for early treatment attended in a single center in Northern Italy. Journal of Infection and Public Health, 16(1). https://doi.org/10.1016/j.jiph.2022.11.027

Maida, C. M., Mazzucco, W., Priano, W., Palermo, R., Graziano, G., Costantino, C., Russo, A., Andolina, G., Restivo, I., Giangreco, V., Iaia, F. R., Santino, A., Li Muli, R., Guzzetta, V., Vitale, F., & Tramuto, F. (2024). Detection of influenza virus in urban wastewater during the season 2022/2023 in Sicily, Italy. Frontiers in Public Health, 12. https://doi.org/10.3389/fpubh.2024.1383536

Maier, B. F., Rose, A. H., Burdinski, A., Klamser, P., Neuhauser, H., Wichmann, O., Schaade, L., Wieler, L. H., & Brockmann, D. (2022). Estimating the distribution of COVID-19-susceptible, -recovered, and -vaccinated individuals in Germany up to April 2022. medRxiv, (Maier B.F., benjaminfrankmaier@gmail.com; Rose A.H.; Burdinski A.; Klamser P.; Schaade L.; Wieler L.H.; Brockmann D.) Robert Koch Institute, Nordufer 20, Berlin, Germany. https://doi.org/10.1101/2022.04.19.22274030

Maifredi, G., Izzo, I., Gasparotti, C., Sileo, C. V., Hiv-CoV Group, null, Castelli, F., & Quiros-Roldan, E. (2022). SARS-CoV-2 Infection and Vaccination Coverage among Fragile Populations in a Local Health Area of Northern Italy. Life (Basel, Switzerland), 12(7). https://doi.org/10.3390/life12071009

Maldonado, M., Ossorio, M., del Peso, G., Santos-Alonso, C., Álvarez, L., Sánchez-Villanueva, R., Rivas, B., Vega, C., Selgas, R., & Bajo, M. A. (2021). COVID-19 incidence and outcomes in a home dialysis unit in Madrid (Spain) at the height of the pandemic. Nefrologia, 41(3), 329–336. https://doi.org/10.1016/j.nefroe.2020.09.009

Mallah, N., Pardo-Seco, J., Ares-Gómez, S., López-Pérez, L.-R., González-Pérez, J.-M., Rosón, B., Otero-Barrós, M.-T., Durán-Parrondo, C., Nartallo-Penas, V., Mirás-Carballal, S., Rodríguez-Tenreiro-Sánchez, C., Rivero-Calle, I., Gómez-Carballa, A., Salas, A., & Martinón-Torres, F. (2023). COVID-19 vaccine effectiveness in children by age groups. A population-based study in Galicia, Spain. Pediatric Allergy and Immunology: Official Publication of the European Society of Pediatric Allergy and Immunology, 34(10). https://doi.org/10.1111/pai.14037

Mallah, N., Pardo-Seco, J., López-Pérez, L.-R., González-Pérez, J.-M., Rosón, B., Otero-Barrós, M.-T., Durán-Parrondo, C., Nartallo-Penas, V., Mirás-Carballal, S., Rodríguez-Tenreiro, C., Rivero-Calle, I., Gómez-Carballa, A., Salas, A., & Martinón-Torres, F. (2022). Effectiveness of COVID-19 vaccine booster in the general population and in subjects with comorbidities. A population-based study in Spain. Environmental Research, 215(Pt 2). https://doi.org/10.1016/j.envres.2022.114252

Manansala, R., Bilcke, J., Willem, L., Hens, N., & Beutels, P. (2025). Optimizing influenza vaccine allocation by age using cost-effectiveness analysis: A comparison of 6720 vaccination program scenarios in children and adults in Belgium. Epidemics, 51, 100826. https://doi.org/10.1016/j.epidem.2025.100826

Mancinelli, L., Onori, M., Concato, C., Sorge, R., Chiavelli, S., Coltella, L., Raucci, U., Reale, A., Menichella, D., & Russo, C. (2016). Clinical features of children hospitalized with influenza A and B infections during the 2012-2013 influenza season in Italy. BMC Infectious Diseases, 16. https://doi.org/10.1186/s12879-015-1333-x

Mancini, P., Brandtner, D., Veneri, C., Bonanno Ferraro, G., Iaconelli, M., Puzelli, S., Facchini, M., Di Mario, G., Stefanelli, P., Lucentini, L., Muratore, A., SARI network, Suffredini, E., & La Rosa, G. (2024). Evaluation of Trends in Influenza A and B Viruses in Wastewater and Human Surveillance Data : Insights from the 2022-2023 Season in Italy. Food and Environmental Virology, 17(1). https://doi.org/10.1007/s12560-024-09622-2

Manciulli, T., Spinicci, M., Rossetti, B., Antonello, R. M., Lagi, F., Barbiero, A., Chechi, F., Formica, G., Francalanci, E., Alesi, M., Gaggioli, S., Modi, G., Modica, S., Paggi, R., Costa, C., Morea, A., Paglicci, L., Rancan, I., Amadori, F., … Bartoloni, A. (2023). Safety and Efficacy of Outpatient Treatments for COVID-19 : Real-Life Data from a Regionwide Cohort of High-Risk Patients in Tuscany, Italy (the FEDERATE Cohort). Viruses, 15(2). https://doi.org/10.3390/v15020438

Mangas-Moro, A., Zamarrón, E., Carpio, C., Álvarez-Sala, R., Arribas-López, J. R., Prados, C., & Seasonal Flu Working Group of La Paz University Hospital. (2024). Influenza vaccination mitigates severe complications in hospitalized patients : A ten-year observational study, Spain, 2009-2019. American Journal of Infection Control, 52(5). https://doi.org/10.1016/j.ajic.2023.11.009

Mangone, L., Gioia, F., Mancuso, P., Bisceglia, I., Ottone, M., Vicentini, M., Pinto, C., & Giorgi Rossi, P. (2021). Cumulative COVID-19 incidence, mortality and prognosis in cancer survivors : A population-based study in Reggio Emilia, Northern Italy. International Journal of Cancer, 149(4). https://doi.org/10.1002/ijc.33601

Manica, M., Guzzetta, G., Riccardo, F., Valenti, A., Poletti, P., Marziano, V., Trentini, F., Andrianou, X., Mateo-Urdiales, A., Del Manso, M., Fabiani, M., Vescio, M. F., Spuri, M., Petrone, D., Bella, A., Iavicoli, S., Ajelli, M., Brusaferro, S., Pezzotti, P., & Merler, S. (2021). Impact of tiered restrictions on human activities and the epidemiology of the second wave of COVID-19 in Italy. Nature Communications, 12(1). https://doi.org/10.1038/s41467-021-24832-z

Manley, H., Bayley, T., Danelian, G., Burton, L., Finnie, T., Charlett, A., Watkins, N. A., Birrel, P., De Angelis, D., Keeling, M., Funk, S., Medley, G., Pellis, L., Baguelin, M., Ackland, G. J., Hutchinson, J., Riley, S., & Panovska-Griffiths, J. (2023). Combining models to generate consensus medium-term projections of hospital admissions, occupancy and deaths relating to COVID-19 in England. medRxiv, (Manley H.; Bayley T.; Danelian G.; Burton L.; Finnie T.; Charlett A.; Watkins N.A.; Birrel P.; De Angelis D.; Hutchinson J.; Riley S.; Panovska-Griffiths J., jasmina.panvoska-griffths@queens.ox.ac.uk) UK Health Security Agency, United Kingdom. https://doi.org/10.1101/2023.11.06.23298026

Mann, A. G., Mangtani, P., Russell, C. A., & Whittaker, J. C. (2013). The impact of targeting all elderly persons in England and Wales for yearly influenza vaccination : Excess mortality due to pneumonia or influenza and time trend study. BMJ Open, 3(8). https://doi.org/10.1136/bmjopen-2013-002743

Mannarà, G., Martinelli, M., Giubbi, C., Rizza, M., Giordano, E., Perdoni, F., Bruno, E., Morella, A., Azzellino, A., Turolla, A., Pedrini, R., Malpei, F., La Rosa, G., Suffredini, E., Cereda, D., Ammoni, E., Villa, S., Pregnolato, F., Lavitrano, M., … Cocuzza, C. E. (2025). Wastewater Surveillance for SARS-CoV-2 in Northern Italy: An Evaluation of Three Different Gene Targets. Microorganisms, 13(2), 236. https://doi.org/10.3390/microorganisms13020236

Manolescu, L. S. C., Zaharia, C. N., Dumitrescu, A. I., Prasacu, I., Radu, M. C., Boeru, A. C., Boidache, L., Nita, I., Necsulescu, A., Medar, C., Cristache, C. M., & Chivu, R. D. (2022). COVID-19 Parental Vaccine Hesitancy in Romania: Nationwide Cross-Sectional Study. Vaccines, 10(4), 493. https://doi.org/10.3390/vaccines10040493

Manz, K. M., Schwettmann, L., Mansmann, U., & Maier, W. (2022). Area Deprivation and COVID-19 Incidence and Mortality in Bavaria, Germany : A Bayesian Geographical Analysis. Frontiers in Public Health, 10. https://doi.org/10.3389/fpubh.2022.927658

Manzanares, A., Pardo-Seco, J., Rivero-Calle, I., Dacosta-Urbieta, A., Mallah, N., Santiago-Pérez, M.-I., Pérez-Martínez, O., Otero-Barrós, M.-T., Suárez-Gaiche, N., Kramer, R., Jin, J., Platero-Alonso, L., Alvárez-Gil, R.-M., Ces-Ozores, O.-M., Nartallo-Penas, V., Mirás-Carballal, S., Piñeiro-Sotelo, M., González-Pérez, J.-M.,

Rodríguez-Tenreiro-Sánchez, C., … Martinón-Torres, F. (2025). Respiratory syncytial virus-related lower respiratory tract infection hospitalizations in infants receiving nirsevimab in Galicia (Spain): The NIRSE-GAL study. European Journal of Pediatrics, 184(5), 321. https://doi.org/10.1007/s00431-025-06151-3

Manzoli, L., Villari, P., Granchelli, C., Savino, A., Carunchio, C., Alessandrini, M., Palumbo, F., De Vito, C., Schioppa, F., Di Stanislao, F., & Boccia, A. (2009). Influenza vaccine effectiveness for the elderly : A cohort study involving general practitioners from Abruzzo, Italy. Journal of Preventive Medicine and Hygiene, 50(2). http://www.ncbi.nlm.nih.gov/pubmed/20099441

Maraia, Z., Mazzoni, T., Turtora, M. P., Tempera, A., Spinosi, M., Vagnoni, A., & Mazzoni, I. (2023). Epidemiological Impact on Use of Antibiotics in Patients Hospitalized for COVID-19 : A Retrospective Cohort Study in Italy. Antibiotics (Basel, Switzerland), 12(5). https://doi.org/10.3390/antibiotics12050912

Marangi, M., Boughattas, S., Valzano, F., La Bella, G., De Nittis, R., Margaglione, M., & Arena, F. (2024). Prevalence of Blastocystis sp. And other gastrointestinal pathogens among diarrheic COVID-19 patients in Italy. New Microbes and New Infections, 58. https://doi.org/10.1016/j.nmni.2024.101228

Marano, G., Boracchi, P., Luconi, E., Pariani, E., Pellegrinelli, L., Galli, C., Gandolfi, C. E., Magoni, M., Piro, A., Scarcella, C., Castaldi, S., & Biganzoli, E. M. (2021). Evaluation of influenza vaccination efficacy in reducing influenza-related complications and excess mortality in Northern Italy (2014-2017). Expert Review of Vaccines, 20(1). https://doi.org/10.1080/14760584.2021.1874927

Marbán-Castro, E., Nedic, I., Ferrari, M., Crespo-Mirasol, E., Ferrer, L., Noya, B., Marin, A., Fumadó, V., López, M., Menéndez, C., Martínez Bueno, C., Llupià, A., Goncé, A., & Bardají, A. (2022). Perceptions of COVID-19 Maternal Vaccination among Pregnant Women and Healthcare Workers and Factors That Influence Vaccine Acceptance : A Cross-Sectional Study in Barcelona, Spain. Vaccines, 10(11). https://doi.org/10.3390/vaccines10111930

Marbus, S. D., Schweitzer, V. A., Groeneveld, G. H., Oosterheert, J. J., Schneeberger, P. M., van der Hoek, W., van Dissel, J. T., van Gageldonk-Lafeber, A. B., & Mangen, M.-J. (2020). Incidence and costs of hospitalized adult influenza patients in The Netherlands : A retrospective observational study. The European Journal of Health Economics: HEPAC: Health Economics in Prevention and Care, 21(5). https://doi.org/10.1007/s10198-020-01172-1

Marc, M. S., Rosca, D., Bratosin, F., Fira-Mladinescu, O., Oancea, C., Pescaru, C. C., Velescu, D., Wellmann, N., Motofelea, A. C., Ciuca, I. M., Saracin, K., & Manolescu, D. (2023). The Effect of Comorbidities and Complications on COVID-19 Mortality: A Detailed Retrospective Study in Western Romania. Journal of Personalized Medicine, 13(11), 1552. https://doi.org/10.3390/jpm13111552

Mărcău, F. C., Gheorghițoiu, R., & Bărbăcioru, I. C. (2022). Survey upon the Reasons of COVID-19 Vaccination Acceptance in Romania. Vaccines, 10(10), 1679. https://doi.org/10.3390/vaccines10101679

Mărcău, F. C., Peptan, C., Nedelcuță, R. M., Băleanu, V. D., Băleanu, A. R., & Niculescu, B. (2022). Parental COVID-19 Vaccine Hesitancy for Children in Romania: National Survey. Vaccines, 10(4), 547. https://doi.org/10.3390/vaccines10040547

Marcellusi, A., Bini, C., Muzii, B., Soudani, S., Kieffer, A., Beuvelet, M., Bozzola, E., Midulla, F., Baraldi, E., Bonanni, P., Boccalini, S., & Orfeo, L. (2025). Economic and clinical burden associated with respiratory syncytial virus and impact of universal immunization with nirsevimab in Italy. Global & Regional Health Technology Assessment, 12. https://doi.org/10.33393/grhta.2025.3182

Marchese, V., Formenti, B., Cola, G., Gregori, N., Albini, E., De Palma, G., Possenti, I., Scala, M., Castelli, F., & Matteelli, A. (2021). Importance of mitigation measures for hospital transmission of SARS-CoV-2 at the onset of the epidemic : The experience of Brescia, Northern Italy. Infection, 49(6). https://doi.org/10.1007/s15010-021-01692-9

Marchi, S., Bruttini, M., Milano, G., Manini, I., Chironna, M., Pariani, E., Manenti, A., Kistner, O., Montomoli, E., Temperton, N., & Trombetta, C. M. (2024). Prevalence of Influenza B/Yamagata Viruses From Season 2012/2013 to 2021/2022 in Italy as an Indication of a Potential Lineage Extinction. Influenza and Other Respiratory Viruses, 18(9). https://doi.org/10.1111/irv.13359

Marco-Franco, J., Pita-Barros, P., González-de-Julián, S., Sabat, I., & Vivas-Consuelo, D. (2021). Simplified Mathematical Modelling of Uncertainty : Cost-Effectiveness of COVID-19 Vaccines in Spain. MATHEMATICS, 9(5). https://doi.org/10.3390/math9050566

Mardiko, A. A., Heinemann, S., Bludau, A., Kaba, H. E. J., Leha, A., von Maltzahn, N., Mutters, N. T., Leistner, R., Mattner, F., & Scheithauer, S. (2022). COVID-19 vaccination strategy for hospital staff in Germany : A cross-sectional study in March-April 2021. The Journal of Hospital Infection, 126. https://doi.org/10.1016/j.jhin.2022.05.012

Mare, C., Belbe, S., & Petrovici, N. (2024). Exploring the spatial clustering and spillover effects of COVID-19 vaccination uptake in Romania: An analysis at municipality level. ASTA-ADVANCES IN STATISTICAL ANALYSIS. https://doi.org/10.1007/s10182-024-00520-3

Mårild, K., Fredlund, H., & Ludvigsson, J. F. (2010). Increased risk of hospital admission for influenza in patients with celiac disease : A nationwide cohort study in Sweden. The American Journal of Gastroenterology, 105(11). https://doi.org/10.1038/ajg.2010.352

Marin, R., Runvik, H., Medvedev, A., & Engblom, S. (2023). Bayesian monitoring of COVID-19 in Sweden. Epidemics, 45. https://doi.org/10.1016/j.epidem.2023.100715

Mariné Barjoan, E., Prouvost-Keller, B., Chaarana, A., Festraëts, J., Geloen, C., Legueult, K., & Pradier, C. (2024). Spatial distribution of SARS-CoV-2 incidence, social inequality, housing conditions, and density in South-Eastern France : Keys for future epidemics. Frontiers in Public Health, 12. https://doi.org/10.3389/fpubh.2024.1422112

Marinescu, A. R., Laza, R., Musta, V. F., Cut, T. G., Dumache, R., Tudor, A., Porosnicu, M., Lazureanu, V. E., & Licker, M. (2021). Clostridium Difficile and COVID-19: General Data, Ribotype, Clinical Form, Treatment-Our Experience from the Largest Infectious Diseases Hospital in Western Romania. Medicina (Kaunas, Lithuania), 57(10), 1099. https://doi.org/10.3390/medicina57101099

Marino, C., Angelici, L., Pistolesi, V., Morabito, S., Di Napoli, A., Calandrini, E., Cascini, S., Bargagli, A. M., Petrosillo, N., Agabiti, N., Davoli, M., & On Behalf Of The Regional Registry Dialysis And Transplant Lazio Region, null. (2021). SARS-CoV-2 Infection in Patients on Dialysis : Incidence and Outcomes in the Lazio Region, Italy. Journal of Clinical Medicine, 10(24). https://doi.org/10.3390/jcm10245818

Mariotti, F., Sponchiado, F., Lagi, F., Moroni, C., Paggi, R., Kiros, S. T., Miele, V., Bartoloni, A., Mencarini, J., & The Cocora Working Group, null. (2023). Latent Tuberculosis Infection and COVID-19 : Analysis of a Cohort of Patients from Careggi University Hospital (Florence, Italy). Infectious Disease Reports, 15(6). https://doi.org/10.3390/idr15060068

Marone, E. M., Bonalumi, G., Curci, R., Arzini, A., Chierico, S., Marazzi, G., Diaco, D. A., Rossini, R., Boschini, S., & Rinaldi, L. F. (2020). Characteristics of Venous Thromboembolism in COVID-19 Patients : A Multicenter Experience from Northern Italy. Annals of Vascular Surgery, 68. https://doi.org/10.1016/j.avsg.2020.07.007

Marquès, M., Rovira, J., Nadal, M., & Domingo, J. L. (2021). Effects of air pollution on the potential transmission and mortality of COVID-19 : A preliminary case-study in Tarragona Province (Catalonia, Spain). Environmental Research, 192. https://doi.org/10.1016/j.envres.2020.110315

Marquet, R. L., Bartelds, A. I. M., van Noort, S. P., Koppeschaar, C. E., Paget, J., Schellevis, F. G., & van der Zee, J. (2006). Internet-based monitoring of influenza-like illness (ILI) in the general population of the Netherlands during the 2003-2004 influenza season. BMC Public Health, 6. https://doi.org/10.1186/1471-2458-6-242

Martella, M., Peano, A., Politano, G., Onorati, R., & Gianino, M. M. (2023). Paediatric hospitalizations over three waves of COVID-19 (February 2020 to May 2021) in Italy : Determinants and rates. PeerJ, 11. https://doi.org/10.7717/peerj.15492

Mårtensson, J., Engerström, L., Walther, S., Grip, J., Berggren, R. K., & Larsson, E. (2020). COVID-19 critical illness in Sweden : Characteristics and outcomes at a national population level. Critical Care and Resuscitation: Journal of the Australasian Academy of Critical Care Medicine, 22(4). https://doi.org/10.51893/2020.4.OA3

Martínez de Victoria Carazo, J., Fernández Reyes, D., de Salazar González, A., Montero Alonso, M. Á., Fernández Morales, P., García García, F., García García, F., Yuste Ossorio, E., Hernández Quero, J., & Guirao Arrabal, E. (2024). Effects of COVID-19-Associated Pulmonary Aspergillosis (CAPA) on the prognosis of severe COVID-19 : Clinical characteristics and risk factors in a second-level hospital from Southern Spain. Enfermedades Infecciosas y Microbiologia Clinica, (Martínez de Victoria Carazo J., jmdvc94@gmail.com; Fernández Reyes D.; Hernández Quero J.; Guirao Arrabal E.) Infectious Diseases Service, Hospital Universitario Clínico San Cecilio, Granada, Spain. Embase. https://doi.org/10.1016/j.eimc.2024.08.007

Martínez, A., Gómez-Barroso, D., Delgado-Sanz, C., Monge, S., Cascajo, A., Marinescu, M., Larrauri, A., Carretero, J., & Singh, D. (2024). Real COVID-19 incidence rate estimate in Spain. REVISTA ESPANOLA DE COMUNICACION EN SALUD. https://doi.org/10.20318/recs.2024.7970

Martínez-Baz, I., Casado, I., Miqueleiz, A., Navascués, A., Pozo, F., Trobajo-Sanmartín, C., Albéniz, E., Elía, F., Burgui, C., Fernández-Huerta, M., Ezpeleta, C., & Castilla, J. (2022). Effectiveness of influenza vaccination in preventing influenza in primary care, Navarre, Spain, 2021/22. Euro Surveillance: Bulletin Europeen Sur Les Maladies Transmissibles = European Communicable Disease Bulletin, 27(26). https://doi.org/10.2807/1560-7917.ES.2022.27.26.2200488

Martínez-Baz, I., Fernández-Huerta, M., Navascués, A., Pozo, F., Trobajo-Sanmartín, C., Casado, I., Echeverria, A., Ezpeleta, C., & Castilla, J. (2023). Influenza Vaccine Effectiveness in Preventing Laboratory-Confirmed Influenza Cases and Hospitalizations in Navarre, Spain, 2022-2023. Vaccines, 11(9). https://doi.org/10.3390/vaccines11091478

Martínez-Baz, I., Martínez-Artola, V., Reina, G., Guevara, M., Cenoz, M. G., Morán, J., Irisarri, F., Arriazu, M., Albeniz, E., Castilla, J., & Primary Health Care Sentinel Network of Navarre. (2013). Effectiveness of the trivalent influenza vaccine in Navarre, Spain, 2010-2011 : A population-based test-negative case-control study. BMC Public Health, 13. https://doi.org/10.1186/1471-2458-13-191

Martínez-Baz, I., Miqueleiz, A., Casado, I., Navascués, A., Trobajo-Sanmartín, C., Burgui, C., Guevara, M., Ezpeleta, C., Castilla, J., & Working Group for the Study of COVID-19 in Navarra. (2021). Effectiveness of COVID-19 vaccines in preventing SARS-CoV-2 infection and hospitalisation, Navarre, Spain, January to April 2021. Euro Surveillance: Bulletin Europeen Sur Les Maladies Transmissibles = European Communicable Disease Bulletin, 26(21). https://doi.org/10.2807/1560-7917.ES.2021.26.21.2100438

Martínez-Baz, I., Navascués, A., Trobajo-Sanmartín, C., Pozo, F., Fernández-Huerta, M., Olazabal-Arruiz, M., Argente-Colas, L., Ezpeleta, G., Echeverria, A., Casado, I., Ezpeleta, C., & Castilla, J. (2025). Effectiveness of influenza vaccination in preventing confirmed influenza cases and hospitalizations in Northern Spain, 2023/24 season : A population-based test-negative case-control study. International Journal of Infectious Diseases: IJID: Official Publication of the International Society for Infectious Diseases, 151. https://doi.org/10.1016/j.ijid.2024.107364

Martínez-Baz, I., Trobajo-Sanmartín, C., Miqueleiz, A., Casado, I., Navascués, A., Burgui, C., Ezpeleta, C., Castilla, J., Guevara, M., Working Group for the Study of COVID-19 in Navarra, & Members of the Working Group for the Study of COVID-19 in Navarra. (2023). Risk reduction of hospitalisation and severe disease in vaccinated COVID-19 cases during the SARS-CoV-2 variant Omicron BA.1-predominant period, Navarre, Spain, January to March 2022. Euro Surveillance: Bulletin Europeen Sur Les Maladies Transmissibles = European Communicable Disease Bulletin, 28(5). https://doi.org/10.2807/1560-7917.ES.2023.28.5.2200337

Martínez-Baz, I., Trobajo-Sanmartín, C., Miqueleiz, A., Egüés, N., García Cenoz, M., Casado, I., Navascués, A., Fernández-Huerta, M., Echeverría, A., Guevara, M., Ezpeleta, C., & Castilla, J. (2024). Hospitalisations and Deaths Averted by COVID-19 Vaccination in Navarre, Spain, 2021-2022. Vaccines, 12(1). https://doi.org/10.3390/vaccines12010058

Martínez-Baz, I., Trobajo-Sanmartín, C., Miqueleiz, A., Guevara, M., Fernández-Huerta, M., Burgui, C., Casado, I., Portillo, M. E., Navascués, A., Ezpeleta, C., Castilla, J., Working Group for the Study of COVID-19 in Navarre, & Investigators, other members of the W. G. for the S. of C.-19 in N. (2021). Product-specific COVID-19 vaccine effectiveness against secondary infection in close contacts, Navarre, Spain, April to August 2021. Euro Surveillance: Bulletin Europeen Sur Les Maladies Transmissibles = European Communicable Disease Bulletin, 26(39). https://doi.org/10.2807/1560-7917.ES.2021.26.39.2100894

Martínez-Cortés, M., León-Dominguez, C. M., Fernandez-Pinero, J., Rodriguez, M., Almonacid, M., Ferrari, M. J., Romero, R., Antona, A., Rivas, M. D., de La Fuente, M., Pérez-Gómez, B., & Pollán, M. (2022). SARS-CoV-2 surveillance strategy in essential workers of the Madrid City Council during the first epidemic wave in Spain, March-July 2020. Occupational and Environmental Medicine, 79(5). https://doi.org/10.1136/oemed-2021-107654

Martinez-Huedo, M. A., Lopez-De-Andrés, A., Mora-Zamorano, E., Hernández-Barrera, V., Jiménez-Trujillo, I., Zamorano-Leon, J. J., & Jiménez-García, R. (2020). Decreasing influenza vaccine coverage among adults with high-risk chronic diseases in Spain from 2014 to 2017. Human Vaccines & Immunotherapeutics, 16(1). https://doi.org/10.1080/21645515.2019.1646577

Martinot, M., Eyriey, M., Gravier, S., Kayser, D., Ion, C., Mohseni-Zadeh, M., Ongagna, J. C., Schieber, A., Kempf, C., & Centre Alsace Study Group. (2022). Evolution of baseline characteristics and severe outcomes in COVID-19 inpatients during the first and second waves in Northeastern France. Infectious Diseases Now, 52(1). https://doi.org/10.1016/j.idnow.2021.10.002

Martín-Sánchez, V., Ruiz-Garcia, A., Vitelli-Storelli, F., Serrano-Cumplido, A., Barquilla-Garcia, A., Micó-Pérez, R. M., Olmo-Quintana, V., Calderón-Montero, A., & Segura-Fragoso, A. (2023). Case-fatality rate of SARS-CoV-2 infection during the third and fifth epidemic waves in Spain : Impact of vaccination. Semergen, 49(7). https://doi.org/10.1016/j.semerg.2023.102026

Maruotti, A., Ciccozzi, M., & Jona-Lasinio, G. (2022). COVID-19-induced excess mortality in Italy during the Omicron wave. IJID Regions, 4. https://doi.org/10.1016/j.ijregi.2022.07.005

Marx, K., Gončarova, K., Fedders, D., Kalbitz, S., Kellner, N., Fedders, M., & Lübbert, C. (2023). Clinical outcomes of hospitalized COVID-19 patients treated with remdesivir : A retrospective analysis of a large tertiary care center in Germany. Infection, 51(1). https://doi.org/10.1007/s15010-022-01841-8

Marziano, V., Guzzetta, G., Mammone, A., Riccardo, F., Poletti, P., Trentini, F., Manica, M., Siddu, A., Bella, A., Stefanelli, P., Pezzotti, P., Ajelli, M., Brusaferro, S., Rezza, G., & Merler, S. (2021). The effect of COVID-19 vaccination in Italy and perspectives for living with the virus. Nature Communications, 12(1). https://doi.org/10.1038/s41467-021-27532-w

Mason, T. F. D., Whitston, M., Hodgson, J., Watkinson, R. E., Lau, Y.-S., Abdulrazeg, O., & Sutton, M. (2021). Effects of BNT162b2 mRNA vaccine on COVID-19 infection and hospitalisation amongst older people : Matched case control study for England. BMC Medicine, 19(1). https://doi.org/10.1186/s12916-021-02149-4

Massey, A., Boennec, C., Restrepo-Ortiz, C. X., Blanchet, C., Alizon, S., & Sofonea, M. T. (2023). Real-time forecasting of COVID-19-related hospital strain in France using a non-Markovian mechanistic model. medRxiv, (Massey A., stats.massey@gmail.com; Sofonea M.T., mircea.sofonea@umontpellier.fr) MIVEGEC, Université de Montpellier, CNRS, IRD, Montpellier, France. https://doi.org/10.1101/2023.02.21.23286228

Mastrolia, M. V., Rubino, C., Resti, M., Trapani, S., & Galli, L. (2019). Characteristics and outcome of influenza-associated encephalopathy/encephalitis among children in a tertiary pediatric hospital in Italy, 2017-2019. BMC Infectious Diseases, 19(1). https://doi.org/10.1186/s12879-019-4636-5

Matabuena, M., Rodríguez-Mier, P., García-Meixide, C., & Leborán, V. (2021). COVID-19 : Estimation of the transmission dynamics in Spain using a stochastic simulator and black-box optimization techniques. COMPUTER METHODS AND PROGRAMS IN BIOMEDICINE, 211. https://doi.org/10.1016/j.cmpb.2021.106399

Matarazzo, N., Coluzzi, R., Imbrenda, V., Lanfredi, M., Galella, M., & Krauss, D. (2025). The role of peripherality in the spread of pandemic: Evidence from Basilicata (Southern Italy) during the first wave of COVID-19. INTERNATIONAL JOURNAL OF DISASTER RISK REDUCTION, 122. https://doi.org/10.1016/j.ijdrr.2025.105457

Mateo-Urdiales, A., Del Manso, M., Andrianou, X., Spuri, M., D’Ancona, F., Filia, A., Rota, M. C., Petrone, D., Vescio, M. F., Riccardo, F., Bella, A., Pezzotti, P., & Fabiani, M. (2021). Initial impact of SARS-Cov-2 vaccination on healthcare workers in Italy- Update on the 28th of March 2021. Vaccine, 39(34). https://doi.org/10.1016/j.vaccine.2021.07.003

Mateo-Urdiales, A., Sacco, C., Fotakis, E. A., Del Manso, M., Bella, A., Riccardo, F., Bressi, M., Rota, M. C., Petrone, D., Siddu, A., Fedele, G., Stefanelli, P., Palamara, A. T., Brusaferro, S., Rezza, G., Pezzotti, P., & Fabiani, M. (2023). Relative effectiveness of monovalent and bivalent mRNA boosters in preventing severe COVID-19 due to omicron BA.5 infection up to 4 months post-administration in people aged 60 years or older in Italy : A retrospective matched cohort study. The Lancet. Infectious Diseases, 23(12). https://doi.org/10.1016/S1473-3099(23)00374-2

Mateo-Urdiales, A., Spila Alegiani, S., Fabiani, M., Pezzotti, P., Filia, A., Massari, M., Riccardo, F., Tallon, M., Proietti, V., Del Manso, M., Puopolo, M., Spuri, M., Morciano, C., D'Ancona, F. P., Da Cas, R., Battilomo, S., Bella, A., Menniti-Ippolito, F., Italian Integrated Surveillance of COVID-19 study group, & on behalf of the Italian COVID-19 vaccines registry. (2021). Risk of SARS-CoV-2 infection and subsequent hospital admission and death at different time intervals since first dose of COVID-19 vaccine administration, Italy, 27 December 2020 to mid-April 2021. Euro Surveillance: Bulletin Europeen Sur Les Maladies Transmissibles = European Communicable Disease Bulletin, 26(25). https://doi.org/10.2807/1560-7917.ES.2021.26.25.2100507

Mathur, R., Rentsch, C. T., Morton, C. E., Hulme, W. J., Schultze, A., MacKenna, B., Eggo, R. M., Bhaskaran, K., Wong, A. Y. S., Williamson, E. J., Forbes, H., Wing, K., McDonald, H. I., Bates, C., Bacon, S., Walker, A. J., Evans, D., Inglesby, P., Mehrkar, A., … OpenSAFELY Collaborative. (2021). Ethnic differences in SARS-CoV-2 infection and COVID-19-related hospitalisation, intensive care unit admission, and death in 17 million adults in England : An observational cohort study using the OpenSAFELY platform. Lancet (London, England), 397(10286). https://doi.org/10.1016/S0140-6736(21)00634-6

Mattera, R. (2022). A weighted approach for spatio-temporal clustering of COVID-19 spread in Italy. Spatial and Spatio-Temporal Epidemiology, 41. https://doi.org/10.1016/j.sste.2022.100500

Mattila, J.-M., Vuorinen, T., & Heikkinen, T. (2023). Trends and Changes in Influenza-associated Hospitalizations in Children During 25 Years in Finland, 1993-2018. The Pediatric Infectious Disease Journal, 42(4). https://doi.org/10.1097/INF.0000000000003815

Mattiuzzi, C., Henry, B. M., & Lippi, G. (2023). Correlation between relative age-standardized mortality rates and COVID-19 mortality over time in Italy. Acta Bio-Medica: Atenei Parmensis, 94(2). https://doi.org/10.23750/abm.v94i2.14139

Mattock, R., Gibbons, I., Moss, J., Mealing, S., Largeron, N., Carroll, S., & Alvarez, F. P. (2021). Cost-effectiveness of high dose versus adjuvanted trivalent influenza vaccines in England and Wales. Journal of Medical Economics, 24(1). https://doi.org/10.1080/13696998.2021.2000780

Matvejeff, A., Laitinen, A., Korhonen, M., Oksanen, L., Geneid, A., Sanmark, E., & Vuorinen, V. (2024). Superspreading of SARS-CoV-2 at a choir rehearsal in Finland-A computational fluid dynamics view on aerosol transmission and patient interviews. PLOS ONE, 19(9). https://doi.org/10.1371/journal.pone.0302250

Mauro, A., De Grazia, F., Lenti, M. V., Penagini, R., Frego, R., Ardizzone, S., Savarino, E., Radaelli, F., Bosani, M., Orlando, S., Amato, A., Dinelli, M., Ferretti, F., Filippi, E., Vecchi, M., Stradella, D., Bardone, M., Pozzi, L., Rovedatti, L., … Di Sabatino, A. (2021). Upper gastrointestinal bleeding in COVID-19 inpatients : Incidence and management in a multicenter experience from Northern Italy. Clinics and Research in Hepatology and Gastroenterology, 45(3). https://doi.org/10.1016/j.clinre.2020.07.025

Mayneris-Perxachs, J., Russo, M. F., Ramos, R., de Hollanda, A., Arxé, A. A., Rottoli, M., Arnoriaga-Rodríguez, M., Comas-Cufí, M., Bartoletti, M., Verrastro, O., Gudiol, C., Fages, E., Giménez, M., Gil, A. de G., Bernante, P., Tinahones, F., Carratalà, J., Pagotto, U., Hernández-Aguado, I., … Obesity-T2DM Covid19 Study Group. (2021). Blood Hemoglobin Substantially Modulates the Impact of Gender, Morbid Obesity, and Hyperglycemia on COVID-19 Death Risk : A Multicenter Study in Italy and Spain. Frontiers in Endocrinology, 12. https://doi.org/10.3389/fendo.2021.741248

Mazagatos, C., Delgado-Sanz, C., Milagro, A., Liébana-Rodríguez, M., & Larrauri, A. (2023). Impact of Influenza Vaccination on the Burden of Severe Influenza in the Elderly : Spain, 2017-2020. Vaccines, 11(6). https://doi.org/10.3390/vaccines11061110

Mazagatos, C., Delgado-Sanz, C., Monge, S., Pozo, F., Oliva, J., Sandonis, V., Gandarillas, A., Quiñones-Rubio, C., Ruiz-Sopeña, C., Gallardo-García, V., Basile, L., Barranco-Boada, M. I., Hidalgo-Pardo, O., Vazquez-

Cancela, O., García-Vázquez, M., Fernández-Sierra, A., Milagro-Beamonte, A., Ordobás, M., Martínez-Ochoa, E., … SARI surveillance VE group in Spain. (2022). COVID-19 vaccine effectiveness against hospitalization due to SARS-CoV-2 : A test-negative design study based on Severe Acute Respiratory Infection (SARI) sentinel surveillance in Spain. Influenza and Other Respiratory Viruses, 16(6). https://doi.org/10.1111/irv.13026

Mazagatos, C., Delgado-Sanz, C., Oliva, J., Gherasim, A., Larrauri, A., & Spanish Influenza Surveillance System. (2018). Exploring the risk of severe outcomes and the role of seasonal influenza vaccination in pregnant women hospitalized with confirmed influenza, Spain, 2010/11-2015/16. PloS One, 13(8). https://doi.org/10.1371/journal.pone.0200934

Mazagatos, C., Godoy, P., Muñoz Almagro, C., Pozo, F., Larrauri, A., & IVE in Pregnant Women Working Group. (2020). Effectiveness of influenza vaccination during pregnancy to prevent severe infection in children under 6 months of age, Spain, 2017-2019. Vaccine, 38(52). https://doi.org/10.1016/j.vaccine.2020.07.014

Mazagatos, C., Mendioroz, J., Rumayor, M. B., Gallardo García, V., Álvarez Río, V., Cebollada Gracia, A. D., Batalla Rebollo, N., Barranco Boada, M. I., Pérez-Martínez, O., Lameiras Azevedo, A. S., López González-Coviella, N., Castrillejo, D., Fernández Ibáñez, A., Giménez Duran, J., Ramírez Córcoles, C., Ramos Marín, V., Larrauri, A., Monge, S., & SARI Sentinel Surveillance RSV Study Group. (2024). Estimated Impact of Nirsevimab on the Incidence of Respiratory Syncytial Virus Infections Requiring Hospital Admission in Children < 1 Year, Weeks 40, 2023, to 8, 2024, Spain. Influenza and Other Respiratory Viruses, 18(5). https://doi.org/10.1111/irv.13294

Mazagatos, C., Monge, S., Olmedo, C., Vega, L., Gallego, P., Martín-Merino, E., Sierra, M. J., Limia, A., Larrauri, A., Working Group for the surveillance and control of COVID-19 in Spain, & Working group for the surveillance and control of COVID-19 in Spain. (2021). Effectiveness of mRNA COVID-19 vaccines in preventing SARS-CoV-2 infections and COVID-19 hospitalisations and deaths in elderly long-term care facility residents, Spain, weeks 53 2020 to 13 2021. Euro Surveillance: Bulletin Europeen Sur Les Maladies Transmissibles = European Communicable Disease Bulletin, 26(24). https://doi.org/10.2807/1560-7917.ES.2021.26.24.2100452

Mazela, J., Jackowska, T., Czech, M., Helwich, E., Martyn, O., Aleksiejuk, P., Smaga, A., Glazewska, J., & Wysocki, J. (2024). Epidemiology of Respiratory Syncytial Virus Hospitalizations in Poland : An Analysis from 2015 to 2023 Covering the Entire Polish Population of Children Aged under Five Years. Viruses, 16(5). https://doi.org/10.3390/v16050704

Mazick, A., Christiansen, A. H., Samuelsson, S., & Mølbak, K. (2006). Using sentinel surveillance to monitor effectiveness of influenza vaccine is feasible : A pilot study in Denmark. Euro Surveillance: Bulletin Europeen Sur Les Maladies Transmissibles = European Communicable Disease Bulletin, 11(10). http://www.ncbi.nlm.nih.gov/pubmed/17130655

Mazzucco, W., Stefanelli, P., Marotta, C., Cernigliaro, A., Maida, C. M., Angeloni, U., Silenzi, A., Fruscione, S., Barone, T., Rezza, G., Vitale, F., Tramuto, F., & SAMI-Surv Collaboration. (2024). SARS-CoV-2 molecular surveillance of migrant populations arriving to Italy via the Mediterranean Sea : Lessons learnt. Epidemiologia E Prevenzione, 48(4-5). https://doi.org/10.19191/EP24.4-5.S1.115

McCarthy, C. V., O’Mara, O., van Leeuwen, E., CMMID COVID-19 Working Group, Jit, M., & Sandmann, F. (2022). The impact of COVID-19 vaccination in prisons in England and Wales : A metapopulation model. BMC Public Health, 22(1). https://doi.org/10.1186/s12889-022-13219-4

McDonald, S. A., Lagerweij, G. R., de Boer, P., de Melker, H. E., Pijnacker, R., Mughini Gras, L., Kretzschmar, M. E., den Hartog, G., van Gageldonk-Lafeber, A. B., RIVM COVID-19 surveillance, epidemiology team, van den F, S., & Wallinga, J. (2022). The estimated disease burden of acute COVID-19 in the Netherlands in 2020, in disability-adjusted life-years. European Journal of Epidemiology, 37(10). https://doi.org/10.1007/s10654-022-00895-0

McDonald, S. A., Presanis, A. M., De Angelis, D., van der Hoek, W., Hooiveld, M., Donker, G., & Kretzschmar, M. E. (2014). An evidence synthesis approach to estimating the incidence of seasonal influenza in the Netherlands. Influenza and Other Respiratory Viruses, 8(1). https://doi.org/10.1111/irv.12201

McDonald, S. A., Soetens, L. C., Schipper, C. M. A., Friesema, I., van den Wijngaard, C. C., Teirlinck, A., Neppelenbroek, N., van den Hof, S., Wallinga, J., & van Hoek, A. J. (2021). Testing behaviour and positivity for SARS-CoV-2 infection : Insights from web-based participatory surveillance in the Netherlands. BMJ Open, 11(12). https://doi.org/10.1136/bmjopen-2021-056077

McDonald, S. A., van den Wijngaard, C. C., Wielders, C. C. H., Friesema, I. H. M., Soetens, L., Paolotti, D., van den Hof, S., & van Hoek, A. J. (2021). Risk factors associated with the incidence of self-reported COVID-19-like illness : Data from a web-based syndromic surveillance system in the Netherlands. Epidemiology and Infection, 149. https://doi.org/10.1017/S0950268821001187

McDonald, S. A., van Asten, L., van der Hoek, W., Donker, G. A., & Wallinga, J. (2016). The impact of national vaccination policy changes on influenza incidence in the Netherlands. Influenza and Other Respiratory Viruses, 10(2). https://doi.org/10.1111/irv.12366

McDonald, S. A., van Lier, A., Plass, D., & Kretzschmar, M. E. (2012). The impact of demographic change on the estimated future burden of infectious diseases : Examples from hepatitis B and seasonal influenza in the Netherlands. BMC Public Health, 12. https://doi.org/10.1186/1471-2458-12-1046

McDonald, S. A., van Wijhe, M., van Asten, L., van der Hoek, W., & Wallinga, J. (2018). Years of Life Lost Due to Influenza-Attributable Mortality in Older Adults in the Netherlands : A Competing-Risks Approach. American Journal of Epidemiology, 187(8). https://doi.org/10.1093/aje/kwy021

McManus, O., Christiansen, L. E., Nauta, M., Krogsgaard, L. W., Bahrenscheer, N. S., von Kappelgaard, L., Christiansen, T., Hansen, M., Hansen, N. C., Kähler, J., Rasmussen, A., Richter, S. R., Rasmussen, L. D., Franck, K. T., & Ethelberg, S. (2023). Predicting COVID-19 Incidence Using Wastewater Surveillance Data, Denmark, October 2021-June 2022. Emerging Infectious Diseases, 29(8). https://doi.org/10.3201/eid2908.221634

Meakin, S., Abbott, S., Bosse, N., Munday, J., Gruson, H., Hellewell, J., Sherratt, K., CMMID COVID-19 Working Group, & Funk, S. (2022). Comparative assessment of methods for short-term forecasts of COVID-19 hospital admissions in England at the local level. BMC Medicine, 20(1). https://doi.org/10.1186/s12916-022-02271-x

Medeiros Figueiredo, A., Daponte-Codina, A., Moreira Marculino Figueiredo, D. C., Toledo Vianna, R. P., Costa de Lima, K., & Gil-García, E. (2021). [Factors associated with the incidence and mortality from COVID-19 in the autonomous communities of Spain]. Gaceta Sanitaria, 35(5). https://doi.org/10.1016/j.gaceta.2020.05.004

Medema, G., Heijnen, L., Elsinga, G., Italiaander, R., & Brouwer, A. (2020). Presence of SARS-Coronavirus-2 RNA in Sewage and Correlation with Reported COVID-19 Prevalence in the Early Stage of the Epidemic in The Netherlands. Environmental Science & Technology Letters, 7(7). https://doi.org/10.1021/acs.estlett.0c00357

Medici, M. C., Arcangeletti, M. C., Rossi, G. A., Lanari, M., Merolla, R., Paparatti, U. D. L., Chezzi, C., & Osservatorio VRS Study Group. (2006). Four year incidence of respiratory syncytial virus infection in infants and young children referred to emergency departments for lower respiratory tract diseases in Italy : The « Osservatorio VRS » Study (2000-2004). The New Microbiologica, 29(1). http://www.ncbi.nlm.nih.gov/pubmed/16608123

Mégarbane, B., Bourasset, F., & Scherrmann, J.-M. (2021). Is Curfew Effective in Limiting SARS-CoV-2 Progression ? An Evaluation in France Based on Epidemiokinetic Analyses. Journal of General Internal Medicine, 36(9). https://doi.org/10.1007/s11606-021-06953-9

Mehl, A., Bergey, F., Cawley, C., & Gilsdorf, A. (2020). Syndromic Surveillance Insights from a Symptom Assessment App Before and During COVID-19 Measures in Germany and the United Kingdom : Results From Repeated Cross-Sectional Analyses. JMIR mHealth and uHealth, 8(10). https://doi.org/10.2196/21364

Meijboom, M. J., Riphagen-Dalhuisen, J., & Hak, E. (2018). The potential economic value of influenza vaccination for healthcare workers in The Netherlands. Influenza and Other Respiratory Viruses, 12(4). https://doi.org/10.1111/irv.12558

Meijboom, M. J., Rozenbaum, M. H., Benedictus, A., Luytjes, W., Kneyber, M. C. J., Wilschut, J. C., Hak, E., & Postma, M. J. (2012). Cost-effectiveness of potential infant vaccination against respiratory syncytial virus infection in The Netherlands. Vaccine, 30(31). https://doi.org/10.1016/j.vaccine.2012.04.072

Meijer, A., Jonges, M., Abbink, F., Ang, W., van Beek, J., Beersma, M., Bloembergen, P., Boucher, C., Claas, E., Donker, G., van Gageldonk-Lafeber, R., Isken, L., de Jong, A., Kroes, A., Leenders, S., van der Lubben, M., Mascini, E., Niesters, B., Oosterheert, J. J., … Koopmans, M. (2011). Oseltamivir-resistant pandemic A(H1N1) 2009 influenza viruses detected through enhanced surveillance in the Netherlands, 2009-2010. Antiviral Research, 92(1), 81–89. https://doi.org/10.1016/j.antiviral.2011.07.004

Meijer, R. I., Hoekstra, T., van den Oever, N. C. G., Simsek, S., van den Bergh, J. P., Douma, R. A., Reidinga, A. C., Moeniralam, H. S., Dormans, T., Amsterdam UMC COVID-19 biobank study group, & Smits, M. M. (2021). Treatment with a DPP-4 inhibitor at time of hospital admission for COVID-19 is not associated with improved clinical outcomes : Data from the COVID-PREDICT cohort study in The Netherlands. Journal of Diabetes and Metabolic Disorders, 20(2). https://doi.org/10.1007/s40200-021-00833-z

Meima, A., Whelan, J., Dijks, J., van der Hagen, N., van Duuren, M., & Tjon-A-Tsien, A. (2023). Introducing a novel « real-time » outbreak alert and notification system to monitor SARS-CoV-2 outbreaks and case fatality in elderly care facilities, the Netherlands, 2020-2022. Journal of Public Health Research, 12(1). https://doi.org/10.1177/22799036231160634

Meintrup, D., Nowak-Machen, M., & Borgmann, S. (2022). A Comparison of Germany and the United Kingdom Indicates That More SARS-CoV-2 Circulation and Less Restrictions in the Warm Season Might Reduce Overall COVID-19 Burden. Life (Basel, Switzerland), 12(7). https://doi.org/10.3390/life12070953

Mejdoubi, M., Djennaoui, M., & Kyndt, X. (2021). Link between COVID-19-related in-hospital mortality in continental France administrative areas and weather : An ecological study. BMJ Open, 11(3). https://doi.org/10.1136/bmjopen-2020-043269

Melazzini, F., Colaneri, M., Fumoso, F., Freddi, G., Lenti, M. V., Pieri, T. C., Piloni, D., Noris, P., Pieresca, C., Preti, P. S., Russo, M., Corsico, A., Tavazzi, G., Baldanti, F., Triarico, A., Mojoli, F., Bruno, R., Di Sabatino, A., & San Matteo Pavia COVID-19 Task Force. (2021). Venous thromboembolism and COVID-19 : A single center experience from an academic tertiary referral hospital of Northern Italy. Internal and Emergency Medicine, 16(5). https://doi.org/10.1007/s11739-020-02550-6

Mellor, J., Christie, R., Guilder, J., Paton, R. S., Elgohari, S., Watson, C., Deeny, S. R., & Ward, T. (2024). A comparative study of influenza surveillance systems and administrative data in England during the 2022-2023 season. PLOS Global Public Health, 4(9). https://doi.org/10.1371/journal.pgph.0003627

Mellor, J., Fyles, M., Paton, R. S., Phillips, A., Overton, C. E., & Ward, T. (2025). Assessing the impact of SARS-CoV-2 on influenza-like illness surveillance trends in the community during the 2023/2024 winter in England. International Journal of Infectious Diseases: IJID: Official Publication of the International Society for Infectious Diseases, 150. https://doi.org/10.1016/j.ijid.2024.107307

Mellor, J., Jones, O., & Ward, T. (2024). The Impact of Healthcare Pressures on the COVID-19 Hospitalisation Fatality Risk in England. Journal of Epidemiology and Global Health, 14(4). https://doi.org/10.1007/s44197-024-00310-9

Mellor, J., Tang, M. L., Jones, O., Ward, T., Riley, S., & Deeny, S. R. (2024). Forecasting COVID-19, Influenza and RSV hospitalisations over winter 2023/24 in England. medRxiv, (Mellor J., jonathon.mellor@ukhsa.gov.uk; Tang M.L.; Jones O.; Ward T.; Riley S.; Deeny S.R.) Data Analytics and Surveillance Group, UK Health Security Agency, London, United Kingdom. https://doi.org/10.1101/2024.09.07.24313175

Mena, G., Casas, I., Casañ, C., Auñón, M., Matas, L., Mòdol, J.-M., & Esteve, M. (2021). Influenza vaccination coverage and factors associated with severe laboratory-confirmed influenza-related illness in patients receiving care at a tertiary hospital in Catalonia (Spain) during the 2018-2019 epidemic season. PloS One, 16(12). https://doi.org/10.1371/journal.pone.0260397

Mendola, M., Tonelli, F., Garletti, F. S., Greco, D., Fiscella, M., Cucchi, I., Costa, M. C., & Carrer, P. (2021). COVID-19 impact and vaccine effectiveness among healthcare workers of a large University Hospital in Lombardy, Italy. La Medicina Del Lavoro, 112(6). https://doi.org/10.23749/mdl.v112i6.11983

Menegale, F., Vezzosi, L., Tirani, M., Scarioni, S., Odelli, S., Morani, F., Borriello, C., Pariani, E., Dorigatti, I., Cereda, D., Merler, S., & Poletti, P. (2025). Impact of routine prophylaxis with monoclonal antibodies and maternal immunisation to prevent respiratory syncytial virus hospitalisations, Lombardy region, Italy, 2024/25 season. Euro Surveillance: Bulletin Europeen Sur Les Maladies Transmissibles = European Communicable Disease Bulletin, 30(14), 2400637. https://doi.org/10.2807/1560-7917.ES.2025.30.14.2400637

Mengual-Chuliá, B., Alonso-Cordero, A., Cano, L., Mosquera, M. D. M., de Molina, P., Vendrell, R., Reyes-Prieto, M., Jané, M., Torner, N., Martínez, A. I., Vila, J., Díez-Domingo, J., Marcos, M. Á., & López-Labrador, F. X. (2021). Whole-Genome Analysis Surveillance of Influenza A Virus Resistance to Polymerase Complex Inhibitors in Eastern Spain from 2016 to 2019. Antimicrobial Agents and Chemotherapy, 65(6). https://doi.org/10.1128/AAC.02718-20

Mennini, F. S., Bini, C., Marcellusi, A., Rinaldi, A., & Franco, E. (2018). Cost-effectiveness of switching from trivalent to quadrivalent inactivated influenza vaccines for the at-risk population in Italy. Human Vaccines & Immunotherapeutics, 14(8). https://doi.org/10.1080/21645515.2018.1469368

Mennis, E., Hobus, M., van den Muijsenbergh, M., & van Loenen, T. (2024). COVID-19 related morbidity and mortality in people experiencing homelessness in the Netherlands. PloS One, 19(2). https://doi.org/10.1371/journal.pone.0296754

Menniti-Ippolito, F., Da Cas, R., Traversa, G., Santuccio, C., Felicetti, P., Tartaglia, L., Trotta, F., Di Pietro, P., Barabino, P., Renna, S., Riceputi, L., Tovo, P.-A., Gabiano, C., Urbino, A., Baroero, L., Le Serre, D., Virano, S., Perilongo, G., Daverio, M., … Italian Multicentre Study Group for Drug and Vaccine Safety in Children. (2014). Vaccine effectiveness against severe laboratory-confirmed influenza in children : Results of two consecutive seasons in Italy. Vaccine, 32(35). https://doi.org/10.1016/j.vaccine.2014.06.048

Menon, D. K., Taylor, B. L., & Ridley, S. A. (2005). Modelling the impact of an influenza pandemic on critical care services in England. Anaesthesia, 60(10), 952–954. https://doi.org/10.1111/j.1365-2044.2005.04372.x

Mensah, A. A., Campbell, H., Stowe, J., Seghezzo, G., Simmons, R., Lacy, J., Bukasa, A., O'Boyle, S., Ramsay, M. E., Brown, K., & Ladhani, S. (2021). Risk of SARS-CoV-2 reinfections in children : Prospective national surveillance, January 2020 to July 2021, England. medRxiv, (Mensah A.A.; Campbell H., Helen.Campbell@phe.gov.uk; Stowe J.; Simmons R.; Lacy J.; Bukasa A.; O'Boyle S.; Ramsay M.E.; Brown K.; Ladhani S.) Immunisation and Vaccine Preventable Diseases Division, UK Health Security Agency, London, United Kingdom. https://doi.org/10.1101/2021.12.10.21267372

Mensah, A. A., Sinnathamby, M., Zaidi, A., Coughlan, L., Simmons, R., Ismail, S. A., Ramsay, M. E., Saliba, V., & Ladhani, S. N. (2021). SARS-CoV-2 infections in children following the full re-opening of schools and the impact of national lockdown : Prospective, national observational cohort surveillance, July-December 2020, England. The Journal of Infection, 82(4). https://doi.org/10.1016/j.jinf.2021.02.022

Mercier, A., Wilkinson, D. A., Lebarbenchon, C., Mavingui, P., & Yemadje-Menudier, L. (2022). Spread of SARS-CoV-2 Variants on Réunion Island, France, 2021. Emerging Infectious Diseases, 28(4). https://doi.org/10.3201/eid2804.212243

Merino, M., Marinescu, M., Cascajo, A., Carretero, J., & Singh, D. (2023). Evaluating the spread of Omicron COVID-19 variant in Spain*. FUTURE GENERATION COMPUTER SYSTEMS-THE INTERNATIONAL JOURNAL OF ESCIENCE, 149. https://doi.org/10.1016/j.future.2023.07.025

Merk, H., Kühlmann-Berenzon, S., Linde, A., & Nyrén, O. (2014). Associations of hand-washing frequency with incidence of acute respiratory tract infection and influenza-like illness in adults : A population-based study in Sweden. BMC Infectious Diseases, 14. https://doi.org/10.1186/1471-2334-14-509

Merk, H., Nylén, G., Kühlmann-Berenzon, S., & Linde, A. (2014). Number needed to vaccinate to prevent hospitalizations of pregnant women due to inter-pandemic influenza in Sweden, 2003-2009. Vaccine, 32(52), 7135–7140. https://doi.org/10.1016/j.vaccine.2014.10.027

Méroc, E., Liang, C., Iantomasi, R., Onwuchekwa, C., Innocenti, G. P., d'Angela, D., Molalign, S., Tran, T. M. P., Basu, S., Gessner, B. D., Bruyndonckx, R., Polkowska-Kramek, A., & Begier, E. (2024). A Model-Based Estimation of RSV-Attributable Incidence of Hospitalizations and Deaths in Italy Between 2015 and 2019. Infectious Diseases and Therapy, 13(11). https://doi.org/10.1007/s40121-024-01041-x

Merrick, R., Chudasama, D., Flannagan, J., Campos-Matos, I., Howard, A., Bindra, R., Gill, O. N., Dabrera, G., & Lamagni, T. (2022). Differential impact of quarantine policies for recovered COVID-19 cases in England : A case cohort study of surveillance data, June to December 2020. BMC Public Health, 22(1). https://doi.org/10.1186/s12889-022-14254-x

Meschiari, M., Cozzi-Lepri, A., Tonelli, R., Bacca, E., Menozzi, M., Franceschini, E., Cuomo, G., Bedini, A., Volpi, S., Milic, J., Brugioni, L., Romagnoli, E., Pietrangelo, A., Corradini, E., Coloretti, I., Biagioni, E., Busani, S., Girardis, M., Cossarizza, A., … Modena COVID-19 Working Group (MoCo19). (2022). First and second waves among hospitalised patients with COVID-19 with severe pneumonia: A comparison of 28-day mortality over the 1-year pandemic in a tertiary university hospital in Italy. BMJ Open, 12(1), e054069. https://doi.org/10.1136/bmjopen-2021-054069

Meurisse, M., Catteau, L., van Loenhout, J. A. F., Braeye, T., De Mot, L., Serrien, B., Blot, K., Cauët, E., Van Oyen, H., Cuypers, L., Belgian Collaborative Group On Covid-Hospital Surveillance, null, Covid-Genomics Belgium Consortium, null, Robert, A., & Van Goethem, N. (2023). Homologous and Heterologous Prime-Boost Vaccination : Impact on Clinical Severity of SARS-CoV-2 Omicron Infection among Hospitalized COVID-19 Patients in Belgium. Vaccines, 11(2). https://doi.org/10.3390/vaccines11020378

Meurisse, M., Lajot, A., Devleesschauwer, B., Van Cauteren, D., Van Oyen, H., Van den Borre, L., & Brondeel, R. (2022). The association between area deprivation and COVID-19 incidence : A municipality-level spatio-temporal study in Belgium, 2020-2021. Archives of Public Health = Archives Belges De Sante Publique, 80(1). https://doi.org/10.1186/s13690-022-00856-9

Meurisse, M., Lajot, A., Dupont, Y., Lesenfants, M., Klamer, S., Rebolledo, J., Lernout, T., Leroy, M., Capron, A., Van Bussel, J., Quoilin, S., Andre, E., Kehoe, K., Waumans, L., Van Acker, J., Vandenberg, O., Van den Wijngaert, S., Verdonck, A., Cuypers, L., & Van Cauteren, D. (2021). One year of laboratory-based COVID-19 surveillance system in Belgium : Main indicators and performance of the laboratories (March 2020-21). Archives of Public Health = Archives Belges De Sante Publique, 79(1). https://doi.org/10.1186/s13690-021-00704-2

Meyer, M., Ruebsteck, E., Eifinger, F., Klein, F., Oberthuer, A., van Koningsbruggen-Rietschel, S., Huenseler, C., & Weber, L. T. (2022). Morbidity of Respiratory Syncytial Virus (RSV) Infections : RSV Compared With Severe Acute Respiratory Syndrome Coronavirus 2 Infections in Children Aged 0-4 Years in Cologne, Germany. The Journal of Infectious Diseases, 226(12). https://doi.org/10.1093/infdis/jiac052

Meyers, E., Deschepper, E., Duysburgh, E., De Rop, L., De Burghgraeve, T., Van Ngoc, P., Digregorio, M., Delogne, S., Coen, A., De Clercq, N., Buret, L., Coenen, S., Sutter, A. D., Scholtes, B., Verbakel, J. Y., Cools, P., & Heytens, S. (2022). Declining Prevalence of SARS-CoV-2 Antibodies among Vaccinated Nursing Home Residents and Staff Six Months after the Primary BNT162b2 Vaccination Campaign in Belgium : A Prospective Cohort Study. Viruses, 14(11). https://doi.org/10.3390/v14112361

Michelozzi, P., de' Donato, F., De Sario, M., Scortichini, M., Stafoggia, M., Noccioli, F., Andrianou, X., Boros, S., Del Manso, M., Fabiani, M., Urdiales, A. M., Pezzotti, P., Rossi, P., Rezza, G., Costa, G., & Davoli, M. (2020). [Temporal variations in excess mortality during phase 1 and phase 2 of the COVID-19 epidemic in Italy]. Epidemiologia E Prevenzione, 44(5-6 Suppl 2). https://doi.org/10.19191/EP20.5-6.S2.123

Michelozzi, P., de'Donato, F., Scortichini, M., De Sario, M., Noccioli, F., Rossi, P., & Davoli, M. (2020). Mortality impacts of the coronavirus disease (COVID-19) outbreak by sex and age : Rapid mortality surveillance system, Italy, 1 February to 18 April 2020. Euro Surveillance: Bulletin Europeen Sur Les Maladies Transmissibles = European Communicable Disease Bulletin, 25(19). https://doi.org/10.2807/1560-7917.ES.2020.25.19.2000620

Mihai, R. V., Badiu, D., Petcu, L. C., Gheorghe, E., & Postolache, P. (2021). RISK FACTORS OF INFLUENZA A AND B VIRUS INFECTION IN CHILDREN POPULATION FROM SOUTH-EASTERN AREA OF ROMANIA. MEDICAL-SURGICAL JOURNAL-REVISTA MEDICO-CHIRURGICALA, 125(2), 209–216. https://doi.org/10.22551/MSJ.2021.02.05

Mikolai, J., Dorey, P., Keenan, K., & Kulu, H. (2023). Spatial patterns of COVID-19 and non-COVID-19 mortality across waves of infection in England, Wales, and Scotland. Social Science & Medicine (1982), 338. https://doi.org/10.1016/j.socscimed.2023.116330

Milani, L., Cigliano, F., Catalano, A., Macciotta, A., Viola, M., Caramello, V., Costa, G., Ricceri, F., & Sacerdote, C. (2021). Characteristics of patients affecting the duration of positivity at SARS-CoV-2 : A cohort analysis of the first wave of epidemic in Italy. Epidemiologia E Prevenzione, 45(6). https://doi.org/10.19191/EP21.6.141

Milano, G., Capitani, E., Camarri, A., Bova, G., Capecchi, P. L., Lazzeri, G., Lipari, D., Montomoli, E., & Manini, I. (2023). Surveillance of Influenza and Other Airborne Transmission Viruses during the 2021/2022 Season in Hospitalized Subjects in Tuscany, Italy. Vaccines, 11(4). https://doi.org/10.3390/vaccines11040776

Milazzo, L., Lai, A., Pezzati, L., Oreni, L., Bergna, A., Conti, F., Meroni, C., Minisci, D., Galli, M., Corbellino, M., Antinori, S., & Ridolfo, A. L. (2021). Dynamics of the seroprevalence of SARS-CoV-2 antibodies among healthcare workers at a COVID-19 referral hospital in Milan, Italy. Occupational and Environmental Medicine. https://doi.org/10.1136/oemed-2020-107060

Miller, E., Hoschler, K., Hardelid, P., Stanford, E., Andrews, N., & Zambon, M. (2010). Incidence of 2009 pandemic influenza A H1N1 infection in England: A cross-sectional serological study. Lancet (London, England), 375(9720), 1100–1108. https://doi.org/10.1016/S0140-6736(09)62126-7

Minardi, V., Gallo, R., Possenti, V., Contoli, B., Di Fonzo, D., D'Andrea, E., & Masocco, M. (2023). Influenza Vaccination Uptake and Prognostic Factors among Health Professionals in Italy : Results from the Nationwide Surveillance PASSI 2015-2018. Vaccines, 11(7). https://doi.org/10.3390/vaccines11071223

Mingot-Castellano, M. E., Alcalde-Mellado, P., Pascual-Izquierdo, C., Perez Rus, G., Calo Pérez, A., Martinez, M. P., López-Jaime, F. J., Abalo Perez, L., Gonzalez-Porras, J. R., López Fernández, F., Caparrós Miranda, I. S., González-López, T. J., Moreno Beltrán, M. E., Rubio Escuin, R., Jimenez Bárcenas, R., & on behalf GEPTI (Grupo Español de Trombocitopenia Inmune). (2021). Incidence, characteristics and clinical profile of severe acute respiratory syndrome coronavirus-2 (SARS-CoV-2) infection in patients with pre-existing primary immune thrombocytopenia (ITP) in Spain. British Journal of Haematology, 194(3). https://doi.org/10.1111/bjh.17506

Minnai, F., De Bellis, G., Dragani, T. A., & Colombo, F. (2022). COVID-19 mortality in Italy varies by patient age, sex and pandemic wave. Scientific Reports, 12(1), 4604. https://doi.org/10.1038/s41598-022-08573-7

Minodier, L., Masse, S., Capai, L., Blanchon, T., Ceccaldi, P.-E., van der Werf, S., Hanslik, T., Charrel, R., & Falchi, A. (2019). Risk factors for seasonal influenza virus detection in stools of patients consulting in general practice for acute respiratory infections in France, 2014-2016. Influenza and Other Respiratory Viruses, 13(4). https://doi.org/10.1111/irv.12523

Miraglia Del Giudice, G., Della Polla, G., Postiglione, M., & Angelillo, I. F. (2023). Willingness and hesitancy of parents to vaccinate against COVID-19 their children ages 6 months to 4 years with frail conditions in Italy. Frontiers in Public Health, 11. https://doi.org/10.3389/fpubh.2023.1212652

Miraglia Del Giudice, G., Folcarelli, L., Napoli, A., Corea, F., Angelillo, I. F., & Collaborative Working Group. (2022). COVID-19 vaccination hesitancy and willingness among pregnant women in Italy. Frontiers in Public Health, 10. https://doi.org/10.3389/fpubh.2022.995382

Miraglia Del Giudice, G., Napoli, A., Corea, F., Folcarelli, L., & Angelillo, I. F. (2022). Evaluating COVID-19 Vaccine Willingness and Hesitancy among Parents of Children Aged 5-11 Years with Chronic Conditions in Italy. Vaccines, 10(3). https://doi.org/10.3390/vaccines10030396

Mira-Iglesias, A., Demont, C., López-Labrador, F. X., Mengual-Chuliá, B., García-Rubio, J., Carballido-Fernández, M., Tortajada-Girbés, M., Mollar-Maseres, J., Schwarz-Chavarri, G., Puig-Barberà, J., Díez-Domingo, J., & Valencia Hospital Network for the Study of Influenza and other Respiratory Viruses. (2022). Role of age and birth month in infants hospitalized with RSV-confirmed disease in the Valencia Region, Spain. Influenza and Other Respiratory Viruses, 16(2). https://doi.org/10.1111/irv.12937

Mira-Iglesias, A., López-Labrador, F. X., Baselga-Moreno, V., Tortajada-Girbés, M., Mollar-Maseres, J., Carballido-Fernández, M., Schwarz-Chavarri, G., Puig-Barberà, J., Díez-Domingo, J., & Valencia Hospital

Network for the Study of Influenza and Respiratory Viruses Disease. (2019). Influenza vaccine effectiveness against laboratory-confirmed influenza in hospitalised adults aged 60 years or older, Valencia Region, Spain, 2017/18 influenza season. Euro Surveillance: Bulletin Europeen Sur Les Maladies Transmissibles = European Communicable Disease Bulletin, 24(31). https://doi.org/10.2807/1560-7917.ES.2019.24.31.1800461

Mira-Iglesias, A., López-Labrador, F. X., García-Rubio, J., Mengual-Chuliá, B., Tortajada-Girbés, M., Mollar-Maseres, J., Carballido-Fernández, M., Schwarz-Chavarri, G., Puig-Barberà, J., & Díez-Domingo, J. (2021). Influenza Vaccine Effectiveness and Waning Effect in Hospitalized Older Adults. Valencia Region, Spain, 2018/2019 Season. International Journal of Environmental Research and Public Health, 18(3). https://doi.org/10.3390/ijerph18031129

Mira-Iglesias, A., López-Labrador, F. X., Guglieri-López, B., Tortajada-Girbés, M., Baselga-Moreno, V., Cano, L., Mollar-Maseres, J., Carballido-Fernández, M., Schwarz-Chavarri, G., Díez-Domingo, J., Puig-Barberà, J., & Valencia Hospital Network for the Study of Influenza and Respiratory Viruses Disease. (2018). Influenza vaccine effectiveness in preventing hospitalisation of individuals 60 years of age and over with laboratory-confirmed influenza, Valencia Region, Spain, influenza season 2016/17. Euro Surveillance: Bulletin Europeen Sur Les Maladies Transmissibles = European Communicable Disease Bulletin, 23(8). https://doi.org/10.2807/1560-7917.ES.2018.23.8.17-00318

Mira-Iglesias, A., Mengual-Chuliá, B., Cano, L., García-Rubio, J., Tortajada-Girbés, M., Carballido-Fernández, M., Mollar-Maseres, J., Schwarz-Chavarri, G., García-Esteban, S., Puig-Barberà, J., Díez-Domingo, J., & López-Labrador, F. X. (2022). Retrospective screening for SARS-CoV-2 among influenza-like illness hospitalizations : 2018-2019 and 2019-2020 seasons, Valencia region, Spain. Influenza and Other Respiratory Viruses, 16(1). https://doi.org/10.1111/irv.12899

Mirani, M., Favacchio, G., Carrone, F., Betella, N., Biamonte, E., Morenghi, E., Mazziotti, G., & Lania, A. (2020). Impact of Comorbidities and Glycemia at Admission and Dipeptidyl Peptidase 4 Inhibitors in Patients With Type 2 Diabetes With COVID-19: A Case Series From an Academic Hospital in Lombardy, Italy. DIABETES CARE, 43(12), 3042–3049. https://doi.org/10.2337/dc20-1340

Mitchell, A., Hassan, M., Kahn, F., Litins'ka, Y., Almgren, M., Malmqvist, U., Östergren, P.-O., Inghammar, M., Björk, J., & Bennet, L. (2025). Barriers and motivators associated with COVID-19 vaccination-a vaccine acceptance scoring system based on a population survey in southern Sweden. European Journal of Public Health, 35(3), 477–483. https://doi.org/10.1093/eurpub/ckaf030

Mitchell, A., Inghammar, M., Bennet, L., Östergren, P.-O., Moghaddassi, M., & Björk, J. (2024). COVID-19 vaccine uptake in Skåne county, Sweden, in relation to individual-level and area-level sociodemographic factors: A register-based cross-sectional analysis. BMJ Public Health, 2(1), e000437. https://doi.org/10.1136/bmjph-2023-000437

Mksoud, M., Ittermann, T., Holtfreter, B., Söhnel, A., Söhnel, C., Welk, A., Ulm, L., Becker, K., Hübner, N.-O., Rau, A., Kindler, S., & Kocher, T. (2022). Prevalence of SARS-CoV-2 IgG antibodies among dental teams in Germany. Clinical Oral Investigations, 26(5). https://doi.org/10.1007/s00784-021-04363-z

Modenese, A., Loney, T., & Gobba, F. (2022). COVID-19-Related Mortality amongst Physicians in Italy : Trend Pre- and Post-SARS-CoV-2 Vaccination Campaign. Healthcare (Basel, Switzerland), 10(7). https://doi.org/10.3390/healthcare10071187

Modig, K., Ahlbom, A., & Ebeling, M. (2021). Excess mortality from COVID-19 : Weekly excess death rates by age and sex for Sweden and its most affected region. European Journal of Public Health, 31(1). https://doi.org/10.1093/eurpub/ckaa218

Mohamed, M. O., Gale, C. P., Kontopantelis, E., Doran, T., de Belder, M., Asaria, M., Luscher, T., Wu, J., Rashid, M., Stephenson, C., Denwood, T., Roebuck, C., Deanfield, J., & Mamas, M. A. (2020). Sex Differences in Mortality Rates and Underlying Conditions for COVID-19 Deaths in England and Wales. Mayo Clinic Proceedings, 95(10). https://doi.org/10.1016/j.mayocp.2020.07.009

Moirano, G., Richiardi, L., Novara, C., & Maule, M. (2020). Approaches to Daily Monitoring of the SARS-CoV-2 Outbreak in Northern Italy. Frontiers in Public Health, 8. https://doi.org/10.3389/fpubh.2020.00222

Moirano, G., Schmid, M., & Barone-Adesi, F. (2020). Short-Term Effects of Mitigation Measures for the Containment of the COVID-19 Outbreak : An Experience From Northern Italy. Disaster Medicine and Public Health Preparedness, 14(4). https://doi.org/10.1017/dmp.2020.119

Mokrani, D., Le Hingrat, Q., Thy, M., Choquet, C., Joly, V., Lariven, S., Rioux, C., Deconinck, L., Loubet, P., Papo, T., Crestani, B., Bunel, V., Bouadma, L., Khalil, A., Armand-Lefèvre, L., Raynaud-Simon, A., Timsit, J.-F., Lescure, F.-X., Yazdanpanah, Y., … Peiffer-Smadja, N. (2024). Clinical characteristics and outcomes of respiratory syncytial virus-associated ARF in immunocompetent patients : A seven-year experience at a tertiary hospital in France. The Journal of Infection, 89(1). https://doi.org/10.1016/j.jinf.2024.106180

Mølbak, K., Widgren, K., Jensen, K. S., Ethelberg, S., Andersen, P. H., Christiansen, A. H., Emborg, H.-D., Gubbels, S., Harder, K. M., Krause, T. G., Mazick, A., Nielsen, L. P., Nielsen, J., Valentiner-Branth, P., & Glismann, S. (2011). Burden of illness of the 2009 pandemic of influenza A (H1N1) in Denmark. Vaccine, 29, B63-69. https://doi.org/10.1016/j.vaccine.2011.03.064

Molenberghs, G., Faes, C., Verbeeck, J., Deboosere, P., Abrams, S., Willem, L., Aerts, J., Theeten, H., Devleesschauwer, B., Bustos Sierra, N., Renard, F., Herzog, S., Lusyne, P., Van der Heyden, J., Van Oyen, H., Van Damme, P., & Hens, N. (2022). COVID-19 mortality, excess mortality, deaths per million and infection fatality ratio, Belgium, 9 March 2020 to 28 June 2020. Euro Surveillance: Bulletin Europeen Sur Les Maladies Transmissibles = European Communicable Disease Bulletin, 27(7). https://doi.org/10.2807/1560-7917.ES.2022.27.7.2002060

Molenkamp, R., Fanoy, E., Derickx, L., de Groot, T., Jonges, M., Leenstra, T., Nijhuis, R., Pas, S., Vahidnia, A., von Wintersdorff, C., Mulder, B., & Koopmans, M. (2021). Supplementing SARS-CoV-2 genomic surveillance with PCR-based variant detection for real-time actionable information, the Netherlands, June to July 2021. Euro Surveillance: Bulletin Europeen Sur Les Maladies Transmissibles = European Communicable Disease Bulletin, 26(40). https://doi.org/10.2807/1560-7917.ES.2021.26.40.2100921

Molnar, D., Anastassopoulou, A., Poulsen Nautrup, B., Schmidt-Ott, R., Eichner, M., Schwehm, M., Dos Santos, G., Ultsch, B., Bekkat-Berkani, R., von Krempelhuber, A., Van Vlaenderen, I., & Van Bellinghen, L.-A. (2022). Cost-utility analysis of increasing uptake of universal seasonal quadrivalent influenza vaccine (QIV) in children aged 6 months and older in Germany. Human Vaccines & Immunotherapeutics, 18(5). https://doi.org/10.1080/21645515.2022.2058304

Monaco, M., Floridia, M., Giuliano, M., Palmieri, L., Lo Noce, C., Pantosti, A., Palamara, A. T., Brusaferro, S., Onder, G., & Italian National Institute of Health COVID-19 Mortality Group. (2022). Hospital-acquired bloodstream infections in patients deceased with COVID-19 in Italy (2020-2021). Frontiers in Medicine, 9. https://doi.org/10.3389/fmed.2022.1041668

Mondejar-Lopez, P., Quintana-Gallego, E., Giron-Moreno, R. M., Cortell-Aznar, I., Ruiz de Valbuena-Maiz, M., Diab-Caceres, L., Prados-Sanchez, C., Alvarez-Fernandez, A., Garcia-Marcos, P. W., Peñalver-Mellado, C., Pastor-Vivero, M. D., Olveira, C., Lopez-Neyra, A., Castillo-Corullon, S., Palma-Milla, S., Perez-Ruiz, E., Sole-Jover, A., Barrio, M. I., Sanchez-Solis, M., … CF-COVID19-Spain Registry Group. (2020). Impact of SARS-CoV-2 infection in patients with cystic fibrosis in Spain : Incidence and results of the national CF-COVID19-Spain survey. Respiratory Medicine, 170. https://doi.org/10.1016/j.rmed.2020.106062

Mondera, F., Cammalleri, V., Forestiero, F. M., Turatto, F., Direnzo, G. F. M., Napoli, A., Pirelli, F., Razafimpanana, N., Rossi, E., Baccolini, V., Cinti, L., Marzuillo, C., Barra, M., Antonelli, G., Badiani, A., & Villari, P. (2023). Adherence to SARS-CoV-2 Vaccination Recommendations among Patients with Substance Use Disorders : A Cross-Sectional Study in Rome, Italy. Vaccines, 11(9). https://doi.org/10.3390/vaccines11091434

Monfardini, L., Morassi, M., Botti, P., Stellini, R., Bettari, L., Pezzotti, S., Alì, M., Monaco, C. G., Magni, V., Cozzi, A., Schiaffino, S., & Bnà, C. (2020). Pulmonary thromboembolism in hospitalised COVID-19 patients at moderate to high risk by Wells score : A report from Lombardy, Italy. The British Journal of Radiology, 93(1113). https://doi.org/10.1259/bjr.20200407

Monge, S., Mazagatos, C., Olmedo, C., Rojas-Benedicto, A., Simón, F., Vega-Piris, L., Sierra, M. J., Limia, A., Larrauri, A., Viloria, L., Pintos, A. M., Gómez, J. H. G., Mateo, A. M., Pérez, A. I. R., Lorusso, N., Herrera, A. A., Alonso, J. P., García-Cenoz, M., Durán, J. G., … García, M. M. (2022). Brand-specific vaccine effectiveness

against SARS-CoV-2 infection, hospitalization and mortality, in people aged 50-59 years in Spain. Revista Espanola de Salud Publica, 96((Monge S., smonge@isciii.es; Mazagatos C.; Rojas-Benedicto A.; Vega-Piris L.; Larrauri A.) National Centre for Epidemiology, Institute of Health Carlos III, Madrid, Spain). Embase. https://www.embase.com/search/results?subaction=viewrecord&id=L2026600866&from=export

Monge, S., Rojas-Benedicto, A., Olmedo, C., Mazagatos, C., José Sierra, M., Limia, A., Martín-Merino, E., Larrauri, A., Hernán, M. A., & IBERCovid. (2022). Effectiveness of mRNA vaccine boosters against infection with the SARS-CoV-2 omicron (B.1.1.529) variant in Spain : A nationwide cohort study. The Lancet. Infectious Diseases, 22(9). https://doi.org/10.1016/S1473-3099(22)00292-4

Monopoli, G., Marino, R., Caldi, F., Fallahi, P., Perretta, S., Cosentino, F., Buselli, R., Gattini, Mignani, A., Veltri, A., Corsi, M., Lucisano, V., Larocca, F., Cristaudo, A., Guglielmi, G., & Foddis, R. (2022). Different COVID-19 outcomes in male and female healthcare workers of University Hospital in Italy. ARCHIVES DES MALADIES PROFESSIONNELLES ET DE L ENVIRONNEMENT, 83(4). https://doi.org/10.1016/j.admp.2022.01.012

Montalti, M., Rallo, F., Guaraldi, F., Bartoli, L., Po, G., Stillo, M., Perrone, P., Squillace, L., Dallolio, L., Pandolfi, P., Resi, D., Fantini, M. P., Reno, C., & Gori, D. (2021). Would Parents Get Their Children Vaccinated Against SARS-CoV-2 ? Rate and Predictors of Vaccine Hesitancy According to a Survey over 5000 Families from Bologna, Italy. Vaccines, 9(4). https://doi.org/10.3390/vaccines9040366

Montero, M. M., Hidalgo López, C., López Montesinos, I., Sorli, L., Barrufet Gonzalez, C., Villar-García, J., Güerri-Fernández, R., Herranz, M., Crespo, M., Arenas Jiménez, M. D., Pascual, J., González Juanes, C., & Horcajada, J. P. (2021). Impact of a Nosocomial COVID-19 Outbreak on a Non-COVID-19 Nephrology Ward during the First Wave of the Pandemic in Spain. Antibiotics (Basel, Switzerland), 10(6), 619. https://doi.org/10.3390/antibiotics10060619

Montes, M., Vicente, D., Pérez-Yarza, E. G., Cilla, G., & Pérez-Trallero, E. (2005). Influenza-related hospitalisations among children aged less than 5 years old in the Basque Country, Spain : A 3-year study (July 2001-June 2004). Vaccine, 23(34). https://doi.org/10.1016/j.vaccine.2005.04.006

Mook, P., Joseph, C., Gates, P., & Phin, N. (2007). Pilot scheme for monitoring sickness absence in schools during the 2006/07 winter in England : Can these data be used as a proxy for influenza activity? Euro Surveillance: Bulletin Europeen Sur Les Maladies Transmissibles = European Communicable Disease Bulletin, 12(12). https://doi.org/10.2807/esm.12.12.00755-en

Moore, G., Rickard, H., Stevenson, D., Aranega-Bou, P., Pitman, J., Crook, A., Davies, K., Spencer, A., Burton, C., Easterbrook, L., Love, H. E., Summers, S., Welch, S. R., Wand, N., Thompson, K.-A., Pottage, T., Richards, K. S., Dunning, J., & Bennett, A. (2021). Detection of SARS-CoV-2 within the healthcare environment : A multi-centre study conducted during the first wave of the COVID-19 outbreak in England. The Journal of Hospital Infection, 108. https://doi.org/10.1016/j.jhin.2020.11.024

Moore, H. L., Turner, C., Rawlinson, C., Chen, C., Verlander, N. Q., Anderson, C., & Hughes, G. J. (2024). Risk factors for COVID-19 transmission in England : A multilevel modelling study using routine contact tracing data. Epidemiology and Infection, 152. https://doi.org/10.1017/S0950268824001043

Mora, J., Pérez, S., & Dvorzhak, A. (2020). Application of a Semi-Empirical Dynamic Model to Forecast the Propagation of the COVID-19 Epidemics in Spain. FORECASTING, 2(4). https://doi.org/10.3390/forecast2040024

Mora, T., Martínez-Marcos, M., & Cabezas-Peña, C. (2025). The influenza vaccination's impact elderly's health outcomes in Catalonia (Spain). Health Policy (Amsterdam, Netherlands), 151. https://doi.org/10.1016/j.healthpol.2024.105213

Moral, L., Marco, N., Toral, T., Fuentes, M. J., Fuentes, L., & Lillo, L. (2011). Burden of severe 2009 pandemic influenza A (H1N1) infection in children in Southeast Spain. Enfermedades Infecciosas Y Microbiologia Clinica, 29(7), 497–501. https://doi.org/10.1016/j.eimc.2011.01.015

Morales-Suárez-Varela, M., Llopis-González, A., González-Candela, F., Astray, J., Alonso, J., Garin, O., Castro, A., Galan, J. C., Soldevila, N., Castilla, J., Godoy, P., Delgado-Rodríguez, M., Martin, V., Mayoral, J. M.,

Pumarola, T., Quintana, J. M., Tamames, S., Rubio-López, N., & Dominguez, A. (2016). Economic Evaluation of Health Services Costs During Pandemic Influenza A (H1N1) Pdm09 Infection in Pregnant and Non-Pregnant Women in Spain. Iranian Journal of Public Health, 45(4), 423–434.

Morales-Suárez-Varela, M., Llopis-González, A., Vergara-Hernández, C., Fernandez-Fabrellas, E., Sanz, F., Perez-Lozano, M. J., Martin, V., Astray, J., Castilla, J., Egurrola, M., Force, L., Toledo, D., & Domínguez, À. (2017). Asthma in older people hospitalized with influenza in Spain : A case-control study. Allergy and Asthma Proceedings, 38(4). https://doi.org/10.2500/aap.2017.38.4060

Moreno Borraz, L. A., Giménez López, M., Carrera Lasfuentes, P., González Pérez, E., Ortíz Domingo, C., Bonafonte Marteles, J. L., Vicente Gaspar, C., Amorós de la Nieta, F., Sastre Heres, A., García Forcada, Á. L., Serrano Herrero, M. P., Fernández Doblado, S., Espinosa Val, M. C., Fernández Adarve, M. M., Narvión Carriquiri, A., Arto Maza, F., Barea Gil, M., Aznar Vázquez, I., Sisas Rubio, R., … Coarasa Lirón de Robles, A. (2021). [Prevalence of SARS-CoV-2 coronavirus infection in patients and professional staff at a medium or long-stay hospital in Spain]. Revista Espanola De Geriatria Y Gerontologia, 56(2). https://doi.org/10.1016/j.regg.2020.10.005

Moreno-Galarraga, L., Urretavizcaya-Martínez, M., Alegría Echauri, J., García Howard, M., Ruperez García, E., Aguilera-Albesa, S., Alzina de Aguilar, V., & Herranz Aguirre, M. (2020). SARS-CoV-2 infection in children requiring hospitalization : The experience of Navarra, Spain. World Journal of Pediatrics: WJP, 16(6). https://doi.org/10.1007/s12519-020-00393-x

Moreno-Iribas, C., Floristán, Y., Iniesta Martinez, I., Aicua, E. A., Guevara Eslava, M., & Delfrade, J. (2022). [Excess of mortality and mortality from COVID-19 and other causes of death in 2020 in Navarra, Spain]. Anales Del Sistema Sanitario De Navarra, 45(3). https://doi.org/10.23938/ASSN.1018

Moreno-Perez, D., Calvo, C., & Five Study Group. (2014). Epidemiological and clinical data of hospitalizations associated with respiratory syncytial virus infection in children under 5 years of age in Spain : FIVE multicenter study. Influenza and Other Respiratory Viruses, 8(2). https://doi.org/10.1111/irv.12224

Moreno-Pérez, D., Korobova, A., Croche-Santander, F. de B., Cordón-Martínez, A., Díaz-Morales, O., Martínez-Campos, L., Pérez-González, E., Martínez-Padilla, M. D. C., Santos-Pérez, J. L., Brioso-Galiana, J., Sánchez-Códez, M. I., Del Diego-Salas, J., Rivera-Izquierdo, M., & Lorusso, N. (2025). Nirsevimab Prophylaxis for Reduction of Respiratory Syncytial Virus Complications in Hospitalised Infants: The Multi-Centre Study During the 2023-2024 Season in Andalusia, Spain (NIRSEGRAND). Vaccines, 13(2), 175. https://doi.org/10.3390/vaccines13020175

Moreno-Torres, V., de la Fuente, S., Mills, P., Muñoz, A., Muñez, E., Ramos, A., Fernández-Cruz, A., Arias, A., Pintos, I., Vargas, J. A., Cuervas-Mons, V., & de Mendoza, C. (2021). Major determinants of death in patients hospitalized with COVID-19 during the first epidemic wave in Madrid, Spain. Medicine, 100(16). https://doi.org/10.1097/MD.0000000000025634

Moreno-Torres, V., Mendoza, C. de, Mellor-Pita, S., Martínez-Urbistondo, M., Durán-Del Campo, P., Tutor-Ureta, P., Vázquez-Comendador, J.-M., Calderón-Parra, J., Múñez-Rubio, E., Ramos-Martínez, A., Fernández-Cruz, A., Castejón, R., & Vargas-Nuñez, J.-A. (2022). Systemic Autoimmune Diseases in Patients Hospitalized with COVID-19 in Spain : A Nation-Wide Registry Study. Viruses, 14(8). https://doi.org/10.3390/v14081631

Moreno-Vásquez, M., Vidal-Alaball, J., Saez, M., Barceló, M. A., & PERIS Project collaborative team. (2024). Impacts of COVID-19 on clinical indicators and mortality in patients with chronic conditions in Catalonia, Spain : A retrospective population-based cohort study. Journal of Global Health, 14. https://doi.org/10.7189/jogh.14.05020

Morfeld, P., & Erren, T. C. (2020). [Deaths in nine regions of Italy in February/March 2020 : « Mortality Excess Loupe » for SARS-CoV-2/COVID-19-Epidemiology in Germany]. Gesundheitswesen (Bundesverband Der Arzte Des Offentlichen Gesundheitsdienstes (Germany)), 82(5). https://doi.org/10.1055/a-1160-5859

Morfeld, P., Timmermann, B., Groß, J. V., Lewis, P., & Erren, T. C. (2021). Before, During, and After the First Wave of COVID-19 : Mortality Analyses Reveal Relevant Trends in Germany and its States until June 2020. Gesundheitswesen (Bundesverband Der Arzte Des Offentlichen Gesundheitsdienstes (Germany)), 83(8-09). https://doi.org/10.1055/a-1531-5507

Morfeld, P., Timmermann, B., Groß, J. V., Lewis, P., Cocco, P., & Erren, T. C. (2021). COVID-19 : Heterogeneous Excess Mortality and « Burden of Disease » in Germany and Italy and Their States and Regions, January-June 2020. Frontiers in Public Health, 9. https://doi.org/10.3389/fpubh.2021.663259

Moriña, D., Fernández-Fontelo, A., Cabaña, A., Arratia, A., Ávalos, G., & Puig, P. (2021). Cumulated burden of COVID-19 in Spain from a Bayesian perspective. European Journal of Public Health, 31(4). https://doi.org/10.1093/eurpub/ckab118

Morrissey, K., Spooner, F., Salter, J., & Shaddick, G. (2021). Area level deprivation and monthly COVID-19 cases : The impact of government policy in England. Social Science & Medicine (1982), 289. https://doi.org/10.1016/j.socscimed.2021.114413

Morrone, A., Di Simone, E., Buonomini, A. R., Panattoni, N., Pimpinelli, F., Pontone, M., Saraceni, P., Ercoli, L., Ralli, M., Petrone, F., & Cristaudo, A. (2023). A report about the experience of COVID-19 active surveillance of homeless, undocumented people, and shelter staff in two cities of Lazio, Italy. European Review for Medical and Pharmacological Sciences, 27(21). https://doi.org/10.26355/eurrev_202311_34359

Mortensen, J. K., Blauenfeldt, R. A., Hedegaard, J. N., Morberg Wejse, C., Johnsen, S. P., Andersen, G., & Simonsen, C. Z. (2024). Prevalence and impact of SARS-CoV-2 infection among patients with acute ischaemic stroke : A nationwide register-based cohort study in Denmark. BMJ Open, 14(3). https://doi.org/10.1136/bmjopen-2023-081527

Morvan, M., Jacomo, A. L., Souque, C., Wade, M. J., Hoffmann, T., Pouwels, K., Lilley, C., Singer, A. C., Porter, J., Evens, N. P., Walker, D. I., Bunce, J. T., Engeli, A., Grimsley, J., O'Reilly, K. M., & Danon, L. (2022). An analysis of 45 large-scale wastewater sites in England to estimate SARS-CoV-2 community prevalence. Nature Communications, 13(1). https://doi.org/10.1038/s41467-022-31753-y

Morwinsky, S., Nitsche, N., & Acosta, E. (2021). COVID-19 fatality in Germany : Demographic determinants of variation in case-fatality rates across and within German federal states during the first and second waves. DEMOGRAPHIC RESEARCH, 45. https://doi.org/10.4054/DemRes.2021.45.45

Mosnier, A., Caini, S., Daviaud, I., Bensoussan, J.-L., Stoll-Keller, F., Bui, T. T., Lina, B., Van der Werf, S., Cohen, J. M., & GROG network. (2015). Ten influenza seasons in France : Distribution and timing of influenza A and B circulation, 2003-2013. BMC Infectious Diseases, 15. https://doi.org/10.1186/s12879-015-1056-z

Mosnier, A., Daviaud, I., Casalegno, J. S., Ruetsch, M., Burugorri, C., Nauleau, E., Bui, T. T., Fleury, H., Lina, B., van der Werf, S., Cohen, J. M., & Regional Groups for Influenza Surveillance. (2017). Influenza B burden during seasonal influenza epidemics in France. Medecine Et Maladies Infectieuses, 47(1). https://doi.org/10.1016/j.medmal.2016.11.006

Mosnino, E., Bernardes, L. S., Mattern, J., Hipólito Micheletti, B., Aparecida de Castro Maldonado, A., Vauloup-Fellous, C., Doucet-Populaire, F., De Luca, D., Benachi, A., & Vivanti, A. J. (2022). Impact of SARS-CoV-2 Alpha and Gamma Variants among Symptomatic Pregnant Women : A Two-Center Retrospective Cohort Study between France and Brazil. Journal of Clinical Medicine, 11(9). https://doi.org/10.3390/jcm11092663

Moss, J. W. E., Davidson, C., Mattock, R., Gibbons, I., Mealing, S., & Carroll, S. (2020). Quantifying the direct secondary health care cost of seasonal influenza in England. BMC Public Health, 20(1). https://doi.org/10.1186/s12889-020-09553-0

Mouter, N., Boxebeld, S., Kessels, R., van Wijhe, M., de Wit, A., Lambooij, M., & van Exel, J. (2022). Public Preferences for Policies to Promote COVID-19 Vaccination Uptake : A Discrete Choice Experiment in The Netherlands. Value in Health: The Journal of the International Society for Pharmacoeconomics and Outcomes Research, 25(8). https://doi.org/10.1016/j.jval.2022.03.013

Muccioli, L., Zenesini, C., Taruffi, L., Licchetta, L., Mostacci, B., Di Vito, L., Pasini, E., Volpi, L., Riguzzi, P., Ferri, L., Baccari, F., Nonino, F., Michelucci, R., Tinuper, P., Vignatelli, L., & Bisulli, F. (2022). Risk of hospitalization and death for COVID-19 in persons with epilepsy over a 20-month period : The EpiLink Bologna cohort, Italy. Epilepsia, 63(9). https://doi.org/10.1111/epi.17356

Muegge, R., Dean, N., Jack, E., & Lee, D. (2023). National lockdowns in England : The same restrictions for all, but do the impacts on COVID-19 mortality risks vary geographically? Spatial and Spatio-Temporal Epidemiology, 44. https://doi.org/10.1016/j.sste.2022.100559

Muenchhoff, M., Graf, A., Krebs, S., Quartucci, C., Hasmann, S., Hellmuth, J. C., Scherer, C., Osterman, A., Boehm, S., Mandel, C., Becker-Pennrich, A. S., Zoller, M., Stubbe, H. C., Munker, S., Munker, D., Milger, K., Gapp, M., Schneider, S., Ruhle, A., … Keppler, O. T. (2021). Genomic epidemiology reveals multiple introductions of SARS-CoV-2 followed by community and nosocomial spread, Germany, February to May 2020. Euro Surveillance: Bulletin Europeen Sur Les Maladies Transmissibles = European Communicable Disease Bulletin, 26(43). https://doi.org/10.2807/1560-7917.ES.2021.26.43.2002066

Mulla, D. P., Bochicchio, M. A., & Longo, A. (2025). Data-driven assessment of the effectiveness of non-pharmaceutical interventions on Covid spread mitigation in Italy. Smart Health, 35((Mulla D.P., divyapragna.mulla@unisalento.it; Longo A.) University of Salento, Lecce, Italy). Embase. https://doi.org/10.1016/j.smhl.2024.100524

Müller, D., & Szucs, T. D. (2005). [Coverage rates of influenza vaccine in Italy during the 2002/3 and 2003/04 seasons : A cross-sectional study]. Annali Di Igiene: Medicina Preventiva E Di Comunita, 17(4). http://www.ncbi.nlm.nih.gov/pubmed/16156395

Müller, D., Saliou, P., & Szucs, T. D. (2006). [Coverage rates of influenza vaccination in France : A population-based cross-sectional analysis of seasons 2001-2002 and 2002-2003]. Medecine Et Maladies Infectieuses, 36(1). https://doi.org/10.1016/j.medmal.2005.05.008

Müller, D., Wutzler, P., & Szucs, T. D. (2005). Influenza vaccination coverage rates in Germany a population-based cross-sectional analysis of the seasons 2002/2003 and 2003/2004. Medizinische Klinik (Munich, Germany: 1983), 100(1). https://doi.org/10.1007/s00063-005-1113-2

Muller, J., Tran Ba Loc, P., Binder Foucard, F., Borde, A., Bruandet, A., Le Bourhis-Zaimi, M., Lenne, X., Ouattara, É., Séguret, F., Gilleron, V., & Tezenas du Montcel, S. (2022). Major interregional differences in France of COVID-19 hospitalization and mortality from January to June 2020. Revue D’epidemiologie Et De Sante Publique, 70(6). https://doi.org/10.1016/j.respe.2022.08.008

Muller, S. A., Manintveld, O. C., Szymanski, M. K., Damman, K., van der Meer, M. G., Caliskan, K., van Laake, L. W., & Oerlemans, M. I. F. J. (2022). Characteristics and outcomes of COVID-19 in heart transplantation recipients in the Netherlands. Netherlands Heart Journal: Monthly Journal of the Netherlands Society of Cardiology and the Netherlands Heart Foundation, 30(11). https://doi.org/10.1007/s12471-022-01720-9

Munday, J. D., Abbott, S., Meakin, S., & Funk, S. (2023). Evaluating the use of social contact data to produce age-specific short-term forecasts of SARS-CoV-2 incidence in England. PLoS Computational Biology, 19(9). https://doi.org/10.1371/journal.pcbi.1011453

Munday, J. D., Jarvis, C. I., Gimma, A., Wong, K. L. M., van Zandvoort, K., CMMID COVID-19 Working Group, Funk, S., & Edmunds, W. J. (2021). Estimating the impact of reopening schools on the reproduction number of SARS-CoV-2 in England, using weekly contact survey data. BMC Medicine, 19(1). https://doi.org/10.1186/s12916-021-02107-0

Munford, L., Khavandi, S., & Bambra, C. (2022). COVID-19 and deprivation amplification : An ecological study of geographical inequalities in mortality in England. Health & Place, 78. https://doi.org/10.1016/j.healthplace.2022.102933

Munier-Marion, E., Bénet, T., Dananché, C., Soing-Altach, S., Maugat, S., Vaux, S., & Vanhems, P. (2017). Outbreaks of health care-associated influenza-like illness in France : Impact of electronic notification. American Journal of Infection Control, 45(11). https://doi.org/10.1016/j.ajic.2017.05.012

Muñoz, M. P., Soldevila, N., Martínez, A., Carmona, G., Batalla, J., Acosta, L. M., & Domínguez, A. (2011). Influenza vaccine coverage, influenza-associated morbidity and all-cause mortality in Catalonia (Spain). Vaccine, 29(31). https://doi.org/10.1016/j.vaccine.2011.04.067

Murakami, M., Fujita, T., Li, P., Imoto, S., & Yasutaka, T. (2022). Development of a COVID-19 risk assessment model for participants at outdoor music festivals : Evaluation of the validity and control measure effectiveness based on two actual events in Japan and Spain. PeerJ, 10. https://doi.org/10.7717/peerj.13846

Murari, A., Gelfusa, M., Craciunescu, T., Gelfusa, C., Gaudio, P., Bovesecchi, G., & Rossi, R. (2023). Effects of environmental conditions on COVID-19 morbidity as an example of multicausality : A multi-city case study in Italy. Frontiers in Public Health, 11. https://doi.org/10.3389/fpubh.2023.1222389

Murdaca, G., Noberasco, G., Battaglini, A., Vassallo, C., Giusti, F., Greco, M., Schiavi, C., Sticchi, L., Icardi, G., & Orsi, A. (2020). Systemic Sclerosis and Vaccinations : A Register-Based Cohort Study about Seasonal Influenza and Streptococcus pneumoniae Vaccination Rate and Uptake from Liguria Regional Center, Northwest Italy. Vaccines, 8(2). https://doi.org/10.3390/vaccines8020204

Murk, J.-L., van de Biggelaar, R., Stohr, J., Verweij, J., Buiting, A., Wittens, S., van Hooft, M., Diederen, B., Kluiters-de Hingh, Y., Ranschaer, E., Brouwer, A., Retera, J., Verheijen, M., Ramnarain, D., van Ek, I., & van Oers, J. (2020). [The first 100 COVID-19 patients admitted to the Elisabeth-Tweesteden Hospital, Tilburg, The Netherlands]. Nederlands Tijdschrift Voor Geneeskunde, 164. http://www.ncbi.nlm.nih.gov/pubmed/32391997

Murray, J., Bottle, A., Sharland, M., Modi, N., Aylin, P., Majeed, A., Saxena, S., & Medicines for Neonates Investigator Group. (2014). Risk factors for hospital admission with RSV bronchiolitis in England : A population-based birth cohort study. PloS One, 9(2). https://doi.org/10.1371/journal.pone.0089186

Murris-Espin, M., Aubert, M., Bosdure, E., Weil-Olivier, C., & Dubus, J.-C. (2008). [Coverage rate of influenza vaccine in healthcare workers in the 12 cystic fibrosis centres of the Greater South Region of France in 2005/2006]. Revue Des Maladies Respiratoires, 25(5). https://doi.org/10.1016/s0761-8425(08)71612-7

Mussard, S., & Alperin, M. (2024). Risk factors associated to the COVID-19 : Comparisons between France and Luxembourg. ECONOMICS BULLETIN, 44(1).

Mytton, O. T., Rutter, P. D., & Donaldson, L. J. (2012). Influenza A(H1N1)pdm09 in England, 2009 to 2011: A greater burden of severe illness in the year after the pandemic than in the pandemic year. Euro Surveillance: Bulletin Europeen Sur Les Maladies Transmissibles = European Communicable Disease Bulletin, 17(14), 20139.

Nab, L., Parker, E. P. K., Andrews, C. D., Hulme, W. J., Fisher, L., Morley, J., Mehrkar, A., MacKenna, B., Inglesby, P., Morton, C. E., Bacon, S. C. J., Hickman, G., Evans, D., Ward, T., Smith, R. M., Davy, S., Dillingham, I., Maude, S., Butler-Cole, B. F. C., … OpenSAFELY Collaborative. (2023). Changes in COVID-19-related mortality across key demographic and clinical subgroups in England from 2020 to 2022 : A retrospective cohort study using the OpenSAFELY platform. The Lancet. Public Health, 8(5). https://doi.org/10.1016/S2468-2667(23)00079-8

Naber, S. K., Bruijning-Verhagen, P. C. J. L., de Hoog, M. L. A., & van Giessen, A. (2020). Cost-effectiveness of inactivated influenza vaccination in children with medical risk conditions in the Netherlands. Vaccine, 38(17). https://doi.org/10.1016/j.vaccine.2020.01.057

Nachtigall, I., Lenga, P., Jóźwiak, K., Thürmann, P., Meier-Hellmann, A., Kuhlen, R., Brederlau, J., Bauer, T., Tebbenjohanns, J., Schwegmann, K., Hauptmann, M., & Dengler, J. (2020). Clinical course and factors associated with outcomes among 1904 patients hospitalized with COVID-19 in Germany : An observational study. Clinical Microbiology and Infection: The Official Publication of the European Society of Clinical Microbiology and Infectious Diseases, 26(12). https://doi.org/10.1016/j.cmi.2020.08.011

Nafilyan, V., Dolby, T., Finning, K., Pawelek, P., Edge, R., Morgan, J., Glickman, M., Pearce, N., & van Tongeren, M. (2022). Differences in COVID-19 vaccination coverage by occupation in England : A national linked data study. OCCUPATIONAL AND ENVIRONMENTAL MEDICINE, 79(11). https://doi.org/10.1136/oemed-2021-108140

Nafilyan, V., Dolby, T., Razieh, C., Gaughan, C. H., Morgan, J., Ayoubkhani, D., Walker, S., Khunti, K., Glickman, M., & Yates, T. (2021). Sociodemographic inequality in COVID-19 vaccination coverage among elderly adults in England : A national linked data study. BMJ Open, 11(7). https://doi.org/10.1136/bmjopen-2021-053402

Nafilyan, V., Islam, N., Mathur, R., Ayoubkhani, D., Banerjee, A., Glickman, M., Humberstone, B., Diamond, I., & Khunti, K. (2021). Ethnic differences in COVID-19 mortality during the first two waves of the Coronavirus Pandemic: A nationwide cohort study of 29 million adults in England. European Journal of Epidemiology, 36(6), 605–617. https://doi.org/10.1007/s10654-021-00765-1

Nafilyan, V., Pawelek, P., Ayoubkhani, D., Rhodes, S., Pembrey, L., Matz, M., Coleman, M., Allemani, C., Windsor-Shellard, B., van Tongeren, M., & Pearce, N. (2022). Occupation and COVID-19 mortality in England : A national linked data study of 14.3 million adults. Occupational and Environmental Medicine, 79(7). https://doi.org/10.1136/oemed-2021-107818

Nafilyan, V., Ward, I. L., Robertson, C., Sheikh, A., & National Core Studies—Immunology Breakthrough Consortium. (2022). Evaluation of Risk Factors for Postbooster Omicron COVID-19 Deaths in England. JAMA Network Open, 5(9). https://doi.org/10.1001/jamanetworkopen.2022.33446

Nagy, L., Heikkinen, T., Sackeyfio, A., & Pitman, R. (2016). The Clinical Impact and Cost Effectiveness of Quadrivalent Versus Trivalent Influenza Vaccination in Finland. PharmacoEconomics, 34(9). https://doi.org/10.1007/s40273-016-0430-z

Naïditch, N., Thébaut, J.-F., Goubel, B., & Sarkozy, F. (2022). Study of determinants of influenza vaccination among diabetic patients in France. Medecine Des Maladies Metaboliques, 16(2). Embase. https://doi.org/10.1016/j.mmm.2021.11.007

Najjar-Debbiny, R., Nobili, A., Mannucci, P., Barnett-Griness, O., Saliba, W., Adir, Y., Galbussera, A., Tettamanti, M., D'Avanzo, B., & Harari, S. (2024). Mortality during the SARS-CoV-2 Pandemic: A Comparative Analysis between Lombardy in Italy and Israel. JOURNAL OF CLINICAL MEDICINE, 13(16). https://doi.org/10.3390/jcm13164766

Naouri, D., Vuagnat, A., Beduneau, G., Dres, M., Pham, T., Mercat, A., Combes, A., Demoule, A., Kimmoun, A., Schmidt, M., & Jamme, M. (2023). Trends in clinical characteristics and outcomes of all critically ill COVID-19 adult patients hospitalized in France between March 2020 and June 2021 : A national database study. Annals of Intensive Care, 13(1). https://doi.org/10.1186/s13613-022-01097-3

Nappo, A., Petricciuolo, M., Berno, G., Carnevali, A., Gruber, C. E. M., Bicchieraro, G., Spaccapelo, R., Rueca, M., Carletti, F., Spezia, P. G., Veneri, C., La Rosa, G., Suffredini, E., Focosi, D., Chillemi, G., Federici, E., & Maggi, F. (2025). One-Year Monitoring of the Evolution of SARS-CoV-2 Omicron Subvariants Through Wastewater Analysis (Central Italy, August 2023-July 2024). Life (Basel, Switzerland), 15(6), 850. https://doi.org/10.3390/life15060850

Nasreddine, R., Florence, E., Moutschen, M., Yombi, J.-C., Goffard, J.-C., Derdelinckx, I., Lacor, P., Vandekerckhove, L., Messiaen, P., Vandecasteele, S., Delforge, M., De Wit, S., & Belgian Research on AIDS and HIV Consortium (BREACH). (2021). Clinical characteristics and outcomes of COVID-19 in people living with HIV in Belgium : A multicenter, retrospective cohort. Journal of Medical Virology, 93(5). https://doi.org/10.1002/jmv.26828

Natalia, Y. A., Faes, C., Neyens, T., Hammami, N., & Molenberghs, G. (2023). Key risk factors associated with fractal dimension based geographical clustering of COVID-19 data in the Flemish and Brussels region, Belgium. Frontiers in Public Health, 11. https://doi.org/10.3389/fpubh.2023.1249141

Navaratnam, A. V., Gray, W. K., Day, J., Wendon, J., & Briggs, T. W. R. (2021). Patient factors and temporal trends associated with COVID-19 in-hospital mortality in England : An observational study using administrative data. The Lancet. Respiratory Medicine, 9(4). https://doi.org/10.1016/S2213-2600(20)30579-8

Nazroo, J., & Becares, L. (2020). Evidence for ethnic inequalities in mortality related to COVID-19 infections : Findings from an ecological analysis of England. BMJ Open, 10(12). https://doi.org/10.1136/bmjopen-2020-041750

Nebreda-Mayoral, T., Miguel-Gómez, M. A., March-Rosselló, G. A., Puente-Fuertes, L., Cantón-Benito, E., Martínez-García, A. M., Muñoz-Martín, A. B., & Orduña-Domingo, A. (2022). Bacterial/fungal infection in hospitalized patients with COVID-19 in a tertiary hospital in the Community of Castilla y León, Spain.

Enfermedades Infecciosas Y Microbiologia Clinica (English Ed.), 40(4). https://doi.org/10.1016/j.eimce.2022.02.002

Neovius, K., Buesch, K., Sandström, K., & Neovius, M. (2011). Cost-effectiveness analysis of palivizumab as respiratory syncytial virus prophylaxis in preterm infants in Sweden. Acta Paediatrica (Oslo, Norway: 1992), 100(10). https://doi.org/10.1111/j.1651-2227.2011.02309.x

Nesbitt, I., Kilner, A., Waldram, A., Richardson, A., Straughan, C., Cresswell, T., & Durham, L. (2012). The impact of influenza A on critical care in the north of England, winter 2010-11. Journal of the Intensive Care Society, 13(2). Embase. https://doi.org/10.1177/175114371201300212

Nessler, K., Krztoń-Królewiecka, A., Chmielowiec, T., Jarczewska, D., & Windak, A. (2014). Determinants of influenza vaccination coverage rates among primary care patients in Krakow, Poland and the surrounding region. Vaccine, 32(52). https://doi.org/10.1016/j.vaccine.2014.10.026

Neuberger, F., Grgic, M., Diefenbacher, S., Spensberger, F., Lehfeld, A.-S., Buchholz, U., Haas, W., Kalicki, B., & Kuger, S. (2022). COVID-19 infections in day care centres in Germany: Social and organisational determinants of infections in children and staff in the second and third wave of the pandemic. BMC Public Health, 22(1), 98. https://doi.org/10.1186/s12889-021-12470-5

Neuhauser, H., Rosario, A. S., Butschalowsky, H., Haller, S., Hoebel, J., Michel, J., Nitsche, A., Poethko-Müller, C., Prütz, F., Schlaud, M., Steinhauer, H. W., Wilking, H., Wieler, L. H., Schaade, L., Liebig, S., Gößwald, A., Grabka, M. M., Zinn, S., & Ziese, T. (2021). Germany's low SARS-CoV-2 seroprevalence confirms effective containment in 2020 : Results of the nationwide RKI-SOEP study. medRxiv, (Neuhauser H., neuhauserh@rki.de; Butschalowsky H.; Haller S.; Hoebel J.; Michel J.; Nitsche A.; Poethko-Müller C.; Prütz F.; Schlaud M.; Wilking H.; Wieler L.H.; Schaade L.; Gößwald A.; Ziese T.) Robert Koch Institute, Berlin, Germany. https://doi.org/10.1101/2021.11.22.21266711

Neumann, M., Aigner, A., Rossow, E., Schwarz, D., Marschallek, M., Steinmann, J., Stücker, R., Koenigs, I., & Stock, P. (2021). Low SARS-CoV-2 seroprevalence but high perception of risk among healthcare workers at children's hospital before second pandemic wave in Germany. World Journal of Pediatrics: WJP, 17(5), 484–494. https://doi.org/10.1007/s12519-021-00447-8

Neyens, T., Faes, C., Vranckx, M., Pepermans, K., Hens, N., Van Damme, P., Molenberghs, G., Aerts, J., & Beutels, P. (2020). Can COVID-19 symptoms as reported in a large-scale online survey be used to optimise spatial predictions of COVID-19 incidence risk in Belgium? Spatial and Spatio-Temporal Epidemiology, 35. https://doi.org/10.1016/j.sste.2020.100379

Nguipdop-Djomo, P., Oswald, W. E., Halliday, K. E., Cook, S., Sturgess, J., Sundaram, N., Warren-Gash, C., Fine, P. E., Glynn, J., Allen, E., Clark, T. G., Ford, B., Judd, A., Ireland, G., Poh, J., Bonell, C., Dawe, F., Rourke, E., Diamond, I., … COVID-19 Schools Infection Survey Study Group. (2023). Risk factors for SARS-CoV-2 infection in primary and secondary school students and staff in England in the 2020/2021 school year : A longitudinal study. International Journal of Infectious Diseases: IJID: Official Publication of the International Society for Infectious Diseases, 128. https://doi.org/10.1016/j.ijid.2022.12.030

Nguyen, M. H., Nguyen, T. H. T., Molenberghs, G., Abrams, S., Hens, N., & Faes, C. (2023). The impact of national and international travel on spatio-temporal transmission of SARS-CoV-2 in Belgium in 2021. BMC Infectious Diseases, 23(1). https://doi.org/10.1186/s12879-023-08368-9

Nguyen, V., Liu, Y., Mumford, R., Flanagan, B., Patel, P., Braithwaite, I., Shrotri, M., Byrne, T., Beale, S., Aryee, A., Fong, W. L. E., Fragaszy, E., Geismar, C., Navaratnam, A. M. D., Hardelid, P., Kovar, J., Pope, A., Cheng, T., Hayward, A., … Virus Watch Collaborative. (2023). Tracking Changes in Mobility Before and After the First SARS-CoV-2 Vaccination Using Global Positioning System Data in England and Wales (Virus Watch) : Prospective Observational Community Cohort Study. JMIR Public Health and Surveillance, 9. https://doi.org/10.2196/38072

Nicotra, E. F., Pili, R., Gaviano, L., Carrogu, G. P., Berti, R., Grassi, P., & Petretto, D. R. (2021). COVID-19 and the excess of mortality in Italy from January to April 2020 : What are the risks for oldest old? Journal of Public Health Research, 11(1). https://doi.org/10.4081/jphr.2021.2399

Niecwietajewa, I., Frączek, M., Mroczkowska, M., & Frączek, M. (2023). Identifying Predominant Causes of Death Among Hospitalized COVID-19 Patients During Poland's Second and Third Waves. Medical Science Monitor: International Medical Journal of Experimental and Clinical Research, 29. https://doi.org/10.12659/MSM.941455

Niedzielewski, K., Bartczuk, R. P., Bielczyk, N., Bogucki, D., Dreger, F., Dudziuk, G., Górski, Ł., Gruziel-Słomka, M., Haman, J., Kaczorek, A., Kisielewski, J., Krupa, B., Moszyński, A., Nowosielski, J. M., Radwan, M., Semeniuk, M., Tymoszuk, U., Zieliński, J., & Rakowski, F. (2024). Forecasting SARS-CoV-2 epidemic dynamic in Poland with the pDyn agent-based model. Epidemics, 49. https://doi.org/10.1016/j.epidem.2024.100801

Niekler, P., Goettler, D., Liese, J. G., & Streng, A. (2024). Hospitalizations due to respiratory syncytial virus (RSV) infections in Germany : A nationwide clinical and direct cost data analysis (2010-2019). Infection, 52(5). https://doi.org/10.1007/s15010-023-02122-8

Nielsen, J., Krause, T. G., & Mølbak, K. (2018). Influenza-associated mortality determined from all-cause mortality, Denmark 2010/11-2016/17 : The FluMOMO model. Influenza and Other Respiratory Viruses, 12(5). https://doi.org/10.1111/irv.12564

Nielsen, J., Mazick, A., Glismann, S., & Mølbak, K. (2011). Excess mortality related to seasonal influenza and extreme temperatures in Denmark, 1994-2010. BMC Infectious Diseases, 11. https://doi.org/10.1186/1471-2334-11-350

Nielsen, J., Rod, N. H., Vestergaard, L. S., & Lange, T. (2021). Estimates of mortality attributable to COVID-19 : A statistical model for monitoring COVID-19 and seasonal influenza, Denmark, spring 2020. Euro Surveillance: Bulletin Europeen Sur Les Maladies Transmissibles = European Communicable Disease Bulletin, 26(8). https://doi.org/10.2807/1560-7917.ES.2021.26.8.2001646

Niessen, A., Teirlinck, A. C., McDonald, S. A., van der Hoek, W., van Gageldonk-Lafeber, R., RIVM COVID-19 epidemiology, surveillance group, & Knol, M. J. (2022). Sex differences in COVID-19 mortality in the Netherlands. Infection, 50(3). https://doi.org/10.1007/s15010-021-01744-0

Niessen, F. A., Bruijning-Verhagen, P. C. J. L., Bonten, M. J. M., VECTOR study group, & Knol, M. J. (2024). Vaccine effectiveness against COVID-19 related hospital admission in the Netherlands by medical risk condition : A test-negative case-control study. Vaccine, 42(15). https://doi.org/10.1016/j.vaccine.2024.04.017

Niessen, F. A., Knol, M. J., Hahné, S. J. M., VECTOR study group, Bonten, M. J. M., & Bruijning-Verhagen, P. C. J. L. (2022). Vaccine effectiveness against COVID-19 related hospital admission in the Netherlands : A test-negative case-control study. Vaccine, 40(34). https://doi.org/10.1016/j.vaccine.2022.06.011

Nightingale, E. S., Abbott, S., Russell, T. W., CMMID Covid-19 Working Group, Lowe, R., Medley, G. F., & Brady, O. J. (2022). The local burden of disease during the first wave of the COVID-19 epidemic in England : Estimation using different data sources from changing surveillance practices. BMC Public Health, 22(1). https://doi.org/10.1186/s12889-022-13069-0

Nijman, G., Wientjes, M., Ramjith, J., Janssen, N., Hoogerwerf, J., Abbink, E., Blaauw, M., Dofferhoff, T., van Apeldoorn, M., Veerman, K., de Mast, Q., Ten Oever, J., Hoefsloot, W., Reijers, M. H., van Crevel, R., & van de Maat, J. S. (2021). Risk factors for in-hospital mortality in laboratory-confirmed COVID-19 patients in the Netherlands : A competing risk survival analysis. PloS One, 16(3). https://doi.org/10.1371/journal.pone.0249231

Nikolaeva, A., & Versnel, J. (2022). Analytical observational study evaluating global pandemic preparedness and the effectiveness of early COVID-19 responses in Ethiopia, Nigeria, Singapore, South Korea, Sweden, Taiwan, UK and USA. BMJ Open, 12(2), e053374. https://doi.org/10.1136/bmjopen-2021-053374

Nikoloudis, D., Kountouras, D., & Hiona, A. (2020). The frequency of combined IFITM3 haplotype involving the reference alleles of both rs12252 and rs34481144 is in line with COVID-19 standardized mortality ratio of ethnic groups in England. PeerJ, 8. https://doi.org/10.7717/peerj.10402

Nitsch-Osuch, A., & Wardyn, K. (2009a). Influenza vaccine coverage in age-related risk groups in Poland, 2004-2007. Central European Journal of Public Health, 17(4). https://doi.org/10.21101/cejph.a3536

Nitsch-Osuch, A., & Wardyn, K. A. (2009b). Influenza and pneumococcal vaccine coverage among persons aged over 65 years in Poland, 2004-2007. Family Medicine and Primary Care Review, 11(3). Embase. https://www.embase.com/search/results?subaction=viewrecord&id=L355350540&from=export

Nittari, G., Marino, P., Gibelli, F., Sossai, P., Sirignano, A., & Ricci, G. (2021). Role of meteorological factors in the spread of the Severe Acute Respiratory Syndrome Coronavirus 2 (SARS-CoV-2) pandemic in Italy. European Review for Medical and Pharmacological Sciences, 25(22), 7135–7143. https://doi.org/10.26355/eurrev_202111_27267

Noel, L., Marion, E., Boufercha, R., Martin, F., Zandotti, C., Charrel, R., Bouhadfane, M., Lehucher-Michel, M. P., & Villa, A. (2022). Screening of health workers exposed to SARS-CoV-2 in a university hospital in the south of France. International Archives of Occupational and Environmental Health, 95(2). https://doi.org/10.1007/s00420-021-01789-6

Nohl, A., Afflerbach, C., Lurz, C., Brune, B., Ohmann, T., Weichert, V., Zeiger, S., & Dudda, M. (2021). Acceptance of COVID-19 Vaccination among Front-Line Health Care Workers : A Nationwide Survey of Emergency Medical Services Personnel from Germany. Vaccines, 9(5). https://doi.org/10.3390/vaccines9050424

Nohynek, H., Baum, U., Syrjänen, R., Ikonen, N., Sundman, J., & Jokinen, J. (2016). Effectiveness of the live attenuated and the inactivated influenza vaccine in two-year-olds—A nationwide cohort study Finland, influenza season 2015/16. Euro Surveillance: Bulletin Europeen Sur Les Maladies Transmissibles = European Communicable Disease Bulletin, 21(38). https://doi.org/10.2807/1560-7917.ES.2016.21.38.30346

Nomah, D. K., Reyes-Urueña, J., Alonso, L., Díaz, Y., Moreno-Fornés, S., Aceiton, J., Bruguera, A., Martín-Iguacel, R., Imaz, A., Gutierrez, M. D. M., Román, R. W., Suanzes, P., Ambrosioni, J., Casabona, J., Miro, J. M., Llibre, J. M., & The Piscis Study Group, null. (2023). Comparative Analysis of Primary and Monovalent Booster SARS-CoV-2 Vaccination Coverage in Adults with and without HIV in Catalonia, Spain. Vaccines, 12(1). https://doi.org/10.3390/vaccines12010044

Nordström, P., Ballin, M., & Nordström, A. (2021). Effectiveness of heterologous ChAdOx1 nCoV-19 and mRNA prime-boost vaccination against symptomatic Covid-19 infection in Sweden : A nationwide cohort study. The Lancet Regional Health. Europe, 11. https://doi.org/10.1016/j.lanepe.2021.100249

Nordström, P., Ballin, M., & Nordström, A. (2022a). Effectiveness of a fourth dose of mRNA COVID-19 vaccine against all-cause mortality in long-term care facility residents and in the oldest old : A nationwide, retrospective cohort study in Sweden. The Lancet Regional Health. Europe, 21. https://doi.org/10.1016/j.lanepe.2022.100466

Nordström, P., Ballin, M., & Nordström, A. (2022b). Risk of infection, hospitalisation, and death up to 9 months after a second dose of COVID-19 vaccine : A retrospective, total population cohort study in Sweden. Lancet (London, England), 399(10327). https://doi.org/10.1016/S0140-6736(22)00089-7

Nordström, P., Ballin, M., & Nordström, A. (2022c). Risk of SARS-CoV-2 reinfection and COVID-19 hospitalisation in individuals with natural and hybrid immunity : A retrospective, total population cohort study in Sweden. The Lancet. Infectious Diseases, 22(6). https://doi.org/10.1016/S1473-3099(22)00143-8

Nordström, P., Ballin, M., & Nordström, A. (2022d). Safety and effectiveness of COVID-19 mRNA vaccination and risk factors for hospitalisation caused by the omicron variant in 0.8 million adolescents : A nationwide cohort study in Sweden. medRxiv, (Nordström P., peter.nordstrom@umu.se; Ballin M.; Nordström A.) Department of Community Medicine and Rehabilitation, Unit of Geriatric Medicine, Umeå University, Umeå, Sweden. https://doi.org/10.1101/2022.10.19.22281286

Nordvall, D., Drobin, D., Timpka, T., & Hahn, R. G. (2023). Co-morbidity associated with development of severe COVID-19 before vaccine availability: A retrospective cohort study in the first pandemic year among the middle-aged and elderly in Jönköping county, Sweden. BMC Infectious Diseases, 23(1), 156. https://doi.org/10.1186/s12879-023-08115-0

Nottmeyer, L. N., & Sera, F. (2021). Influence of temperature, and of relative and absolute humidity on COVID-19 incidence in England—A multi-city time-series study. Environmental Research, 196. https://doi.org/10.1016/j.envres.2021.110977

Novacescu, A. N., Buzzi, B., Bedreag, O., Papurica, M., Rogobete, A. F., Sandesc, D., Sorescu, T., Baditoiu, L., Musuroi, C., Vlad, D., & Licker, M. (2022). Bacterial and Fungal Superinfections in COVID-19 Patients Hospitalized in an Intensive Care Unit from Timișoara, Romania. Infection and Drug Resistance, 15, 7001–7014. https://doi.org/10.2147/IDR.S390681

Novazzi, F., Giombini, E., Rueca, M., Baj, A., Fabeni, L., Genoni, A., Ferrante, F. D., Gramigna, G., Gruber, C. E. M., Boutahar, S., Minosse, C., Butera, O., Pasciuta, R., Focosi, D., Colombo, A., Antinori, A., Girardi, E., Vaia, F., & Maggi, F. (2023). Genomic surveillance of SARS-CoV-2 positive passengers on flights from China to Italy, December 2022. Euro Surveillance: Bulletin Europeen Sur Les Maladies Transmissibles = European Communicable Disease Bulletin, 28(2). https://doi.org/10.2807/1560-7917.ES.2023.28.2.2300008

Novelli, L., Raimondi, F., Ghirardi, A., Pellegrini, D., Capodanno, D., Sotgiu, G., Guagliumi, G., Senni, M., Russo, F. M., Lorini, F. L., Rizzi, M., Barbui, T., Rambaldi, A., Cosentini, R., Grazioli, L. S., Marchesi, G., Sferrazza Papa, G. F., Cesa, S., Colledan, M., … HPG23 COVID-19 Study Group. (2021). At the peak of COVID-19 age and disease severity but not comorbidities are predictors of mortality : COVID-19 burden in Bergamo, Italy. Panminerva Medica, 63(1). https://doi.org/10.23736/S0031-0808.20.04063-X

Novelli, V., Fassio, F., Resani, G., Bussa, M., Durbano, A., Meloni, A., Oliva, G., Cutti, S., Girardi, D., Odone, A., Villani, S., Marena, C., Muzzi, A., & Monti, M. C. (2022). Clinical Characteristics and Potential Risk Factors Associated with the SARS-CoV-2 Infection : Survey on a Health Care Workers (HCWs) Population in Northern Italy. International Journal of Environmental Research and Public Health, 19(13). https://doi.org/10.3390/ijerph19138194

Nuijten, M., Lebmeier, M., & Wittenberg, W. (2009). Cost effectiveness of palivizumab for RSV prevention in high-risk children in the Netherlands. Journal of Medical Economics, 12(4). https://doi.org/10.3111/13696990903316961

Núñez, O., Olmedo, C., Moreno-Perez, D., Lorusso, N., Fernández Martínez, S., Pastor Villalba, P. E., Gutierrez, Á., Alonso Garcia, M., Latasa, P., Sancho, R., Mendioroz, J., Martinez-Marcos, M., Muñoz Platón, E., García Rivera, M. V., Pérez-Martinez, O., Álvarez-Gil, R., Rivas Wagner, E., López Gonzalez-Coviella, N., Zornoza, M., … Nirsevimab Effectiveness Study Collaborators. (2025). Effectiveness of catch-up and at-birth nirsevimab immunisation against RSV hospital admission in the first year of life : A population-based case-control study, Spain, 2023/24 season. Euro Surveillance: Bulletin Europeen Sur Les Maladies Transmissibles = European Communicable Disease Bulletin, 30(5). https://doi.org/10.2807/1560-7917.ES.2025.30.5.2400596

Núñez-Gil, I. J., Fernández-Pérez, C., Estrada, V., Becerra-Muñoz, V. M., El-Battrawy, I., Uribarri, A., Fernández-Rozas, I., Feltes, G., Viana-Llamas, M. C., Trabattoni, D., López-País, J., Pepe, M., Romero, R., Castro-Mejía, A. F., Cerrato, E., Astrua, T. C., D'Ascenzo, F., Fabregat-Andres, O., Moreu, J., … HOPE COVID-19 Investigators. (2021). Mortality risk assessment in Spain and Italy, insights of the HOPE COVID-19 registry. Internal and Emergency Medicine, 16(4). https://doi.org/10.1007/s11739-020-02543-5

Nurchis, M. C., Pascucci, D., Sapienza, M., Villani, L., D'Ambrosio, F., Castrini, F., Specchia, M. L., Laurenti, P., & Damiani, G. (2020). Impact of the Burden of COVID-19 in Italy : Results of Disability-Adjusted Life Years (DALYs) and Productivity Loss. International Journal of Environmental Research and Public Health, 17(12). https://doi.org/10.3390/ijerph17124233

Nuttens, C., Barbet, V., Bignon-Favary, C., Lambourg, E., Fiévez, S., Blanc, E., Vacheret, M., Liliu, H., Vanhems, P., Casalegno, J.-S., Watier, L., Loubet, P., Liang, C., Begier, E., & Lemaitre, M. (2025). Estimation of GP visits, hospitalizations and deaths attributable to RSV and influenza and costs associated with hospitalizations in older adults in France, 2010-2020. medRxiv. https://doi.org/10.1101/2025.04.29.25326541

Nwaru, C., Bonander, C., Li, H., Santosa, A., Löve, J., & Nyberg, F. (2025). Neighbourhood immigrant density and COVID-19 infection and hospitalisation among healthcare workers in Sweden: A register-based observational study. BMJ Public Health, 3(1), e001501. https://doi.org/10.1136/bmjph-2024-001501

Nyberg, T., Ferguson, N. M., Nash, S. G., Webster, H. H., Flaxman, S., Andrews, N., Hinsley, W., Bernal, J. L., Kall, M., Bhatt, S., Blomquist, P., Zaidi, A., Volz, E., Aziz, N. A., Harman, K., Funk, S., Abbott, S., COVID-19 Genomics UK (COG-UK) consortium, Hope, R., … Thelwall, S. (2022). Comparative analysis of the risks of hospitalisation and death associated with SARS-CoV-2 omicron (B.1.1.529) and delta (B.1.617.2) variants in

England : A cohort study. Lancet (London, England), 399(10332). https://doi.org/10.1016/S0140-6736(22)00462-7

Ocak, G., Khairoun, M., van Stigt Thans, M., Meeder, D., Moeniralam, H., Dekker, F. W., Verhaar, M. C., Bos, W. J. W., & Kaasjager, K. A. H. (2023). Migration background and COVID-19 related intensive care unit admission and mortality in the Netherlands : A cohort study. PloS One, 18(4). https://doi.org/10.1371/journal.pone.0284036

Ocana de Sentuary, C., Testard, C., Lagrée, M., Leroy, M., Gasnier, L., Enes-Dias, A., Leruste, C., Diallo, D., Génin, M., Rakza, T., & Dubos, F. (2025). Acceptance and safety of the RSV-preventive treatment of newborns with nirsevimab in the maternity department : A prospective longitudinal cohort study in France. EClinicalMedicine, 79. https://doi.org/10.1016/j.eclinm.2024.102986

Ochel, P., Eitze, S., Siegers, R., Betsch, C., & Seufert, A. (2022). Determinants of Adoption and Rejection of Protective Measures During the SARS-CoV-2 Pandemic: A Longitudinal Study in Germany's Second Wave. SOCIAL PSYCHOLOGICAL BULLETIN, 17. https://doi.org/10.32872/spb.7515

Ochoa Sangrador, C., Garmendia Leiza, J. R., Pérez Boillos, M. J., Pastrana Ara, F., Lorenzo Lobato, M. D. P., & Andrés de Llano, J. M. (2021). [Impact of COVID-19 on mortality in the autonomous community of Castilla y León (Spain)]. Gaceta Sanitaria, 35(5). https://doi.org/10.1016/j.gaceta.2020.04.009

Offergeld, R., Preußel, K., Zeiler, T., Aurich, K., Baumann-Baretti, B. I., Ciesek, S., Corman, V. M., Dienst, V., Drosten, C., Görg, S., Greinacher, A., Grossegesse, M., Haller, S., Heuft, H.-G., Hofmann, N., Horn, P. A., Houareau, C., Gülec, I., Jiménez Klingberg, C. L., … der Heiden, M. A. (2023). Monitoring the SARS-CoV-2 Pandemic: Prevalence of Antibodies in a Large, Repetitive Cross-Sectional Study of Blood Donors in Germany-Results from the SeBluCo Study 2020-2022. Pathogens (Basel, Switzerland), 12(4), 551. https://doi.org/10.3390/pathogens12040551

Oh, D.-Y., Buda, S., Biere, B., Reiche, J., Schlosser, F., Duwe, S., Wedde, M., von Kleist, M., Mielke, M., Wolff, T., & Dürrwald, R. (2021). Trends in respiratory virus circulation following COVID-19-targeted nonpharmaceutical interventions in Germany, January—September 2020 : Analysis of national surveillance data. The Lancet Regional Health. Europe, 6. https://doi.org/10.1016/j.lanepe.2021.100112

Oliva, C., Di Maddaloni, F., Marcellusi, A., & Favato, G. (2021). Cross-regional variations of Covid-19 mortality in Italy : An ecological study. Journal of Public Health (Oxford, England), 43(2). Medline. https://doi.org/10.1093/pubmed/fdaa248

Oliva, J., Delgado-Sanz, C., Larrauri, A., & Spanish Influenza Surveillance System. (2018). Estimating the burden of seasonal influenza in Spain from surveillance of mild and severe influenza disease, 2010-2016. Influenza and Other Respiratory Viruses, 12(1). https://doi.org/10.1111/irv.12499

Olmedo Lucerón, C., Limia Sánchez, A., & Santamarina, C. (2021). [Vaccination confidence against influenza in Spain : Reasons of hesitancy discourses and attitudes in general population and healthcare workers.]. Revista Espanola De Salud Publica, 95. http://www.ncbi.nlm.nih.gov/pubmed/33764342

Olry de Labry-Lima, A., Saez-de la Fuente, J., Abdel-Kader Martin, L., Alegre-Del Rey, E. J., García-Cabrera, E., & Sierra-Sánchez, J. F. (2022). Factors associated with mortality in patients hospitalized for COVID-19 in Spain. Data from the RERFAR registry. Farmacia Hospitalaria: Organo Oficial De Expresion Cientifica De La Sociedad Espanola De Farmacia Hospitalaria, 46(2). http://www.ncbi.nlm.nih.gov/pubmed/35379097

Onorato, L., Calò, F., Maggi, P., Allegorico, E., Gentile, I., Sangiovanni, V., Esposito, V., Dell'Isola, C., Calabria, G., Pisapia, R., Salomone Megna, A., Masullo, A., Manzillo, E., Russo, G., Parrella, R., Dell'Aquila, G., Gambardella, M., Di Perna, F., Pisaturo, M., … CoviCam Group. (2023). Prevalence and Epidemiological and Clinical Features of Bacterial Infections in a Large Cohort of Patients Hospitalized for COVID-19 in Southern Italy : A Multicenter Study. Antibiotics (Basel, Switzerland), 12(7). https://doi.org/10.3390/antibiotics12071124

OpenSAFELY Collaborative, Tazare, J., Walker, A. J., Tomlinson, L. A., Hickman, G., Rentsch, C. T., Williamson, E. J., Bhaskaran, K., Evans, D., Wing, K., Mathur, R., Wong, A. Y., Schultze, A., Bacon, S., Bates, C., Morton, C. E., Curtis, H. J., Nightingale, E., McDonald, H. I., … Goldacre, B. (2022). Rates of serious

clinical outcomes in survivors of hospitalisation with COVID-19 in England : A descriptive cohort study within the OpenSAFELY platform. Wellcome Open Research, 7. https://doi.org/10.12688/wellcomeopenres.17735.1

Orioli, L., Servais, T., Belkhir, L., Laterre, P.-F., Thissen, J.-P., Vandeleene, B., Maiter, D., Yombi, J. C., & Hermans, M. P. (2021). Clinical characteristics and short-term prognosis of in-patients with diabetes and COVID-19 : A retrospective study from an academic center in Belgium. Diabetes & Metabolic Syndrome, 15(1). https://doi.org/10.1016/j.dsx.2020.12.020

Orlando, S., de Santo, C., Mosconi, C., Di Gaspare, F., Chatzichristou, P., Emberti Gialloreti, L., Ciccacci, F., Morciano, L., Varrenti, D., Liotta, G., & Palombi, L. (2023). COVID-19 infection rate and mortality in a local health authority in Italy : Differences between home-dwelling and residential older adults. Public Health in Practice (Oxford, England), 6. https://doi.org/10.1016/j.puhip.2023.100448

Orlewska, K., Kozieł, D., Klusek, J., & Orlewska, E. (2022). Burden of COVID-19 Mortality and Morbidity in Poland in 2020. International Journal of Environmental Research and Public Health, 19(9). https://doi.org/10.3390/ijerph19095432

Orlewska, K., Wierzba, W., & Śliwczynski, A. (2022). Cost-effectiveness analysis of COVID-19 vaccination in Poland. Archives of Medical Science: AMS, 18(4). https://doi.org/10.5114/aoms/144626

Orsi, A., Colomba, G. M. E., Pojero, F., Calamusa, G., Alicino, C., Trucchi, C., Canepa, P., Ansaldi, F., Vitale, F., & Tramuto, F. (2018). Trends of influenza B during the 2010-2016 seasons in 2 regions of north and south Italy : The impact of the vaccine mismatch on influenza immunisation strategy. Human Vaccines & Immunotherapeutics, 14(3). https://doi.org/10.1080/21645515.2017.1342907

Ørsted, I., Mølvadgaard, M., Nielsen, H. L., & Nielsen, H. (2013). The first, second and third wave of pandemic influenza A (H1N1)pdm09 in North Denmark Region 2009-2011: A population-based study of hospitalizations. Influenza and Other Respiratory Viruses, 7(5), 776–782. https://doi.org/10.1111/irv.12093

Ortiz-Lana, N., Garrote, E., Arístegui, J., Rementeria, J., García-Martínez, J.-A., McCoig, C., García-Corbeira, P., Devadiga, R., & Tafalla, M. (2017). A prospective study to assess the burden of influenza-related hospitalizations and emergency department visits among children in Bilbao, Spain (2010-2011). Anales De Pediatria, 87(6). https://doi.org/10.1016/j.anpede.2017.01.002

Örtqvist, A. K., Magnus, M. C., Söderling, J., Oakley, L., Nybo Andersen, A.-M., Håberg, S. E., & Stephansson, O. (2022). The association between maternal characteristics and SARS-CoV-2 in pregnancy : A population-based registry study in Sweden and Norway. Scientific Reports, 12(1). https://doi.org/10.1038/s41598-022-12395-y

Örtqvist, A., Berggren, I., Insulander, M., De Jong, B., & Svenungsson, B. (2011). Effectiveness of an adjuvanted monovalent vaccine against the 2009 pandemic strain of influenza A(H1N1)v, in Stockholm county, Sweden. Clinical Infectious Diseases, 52(10), 1203–1211. https://doi.org/10.1093/cid/cir182

Oshinubi, K., Ibrahim, F., Rachdi, M., & Demongeot, J. (2022). Functional data analysis : Application to daily observation of COVID-19 prevalence in France. AIMS MATHEMATICS, 7(4). https://doi.org/10.3934/math.2022298

Osmënaj, T., van Roon, A., Labuschagne, L., Pijpers, J., Smagge, B., de Melker, H., van den Hof, S., & Hahné, S. (2025). Determinants for not keeping up to date with COVID-19 vaccination in the 2023 vaccination round among medical risk groups, the Netherlands. Vaccine, 62, 127561. https://doi.org/10.1016/j.vaccine.2025.127561

Otte Im Kampe, E., Lehfeld, A.-S., Buda, S., Buchholz, U., & Haas, W. (2020). Surveillance of COVID-19 school outbreaks, Germany, March to August 2020. Euro Surveillance: Bulletin Europeen Sur Les Maladies Transmissibles = European Communicable Disease Bulletin, 25(38). https://doi.org/10.2807/1560-7917.ES.2020.25.38.2001645

Översti, S., Weber, A., Baran, V., Kieninger, B., Dilthey, A., Houwaart, T., Walker, A., Schneider-Brachert, W., & Kühnert, D. (2025). Evolutionary and epidemic dynamics of COVID-19 in Germany exemplified by three Bayesian phylodynamic case studies. Bioinformatics and Biology Insights, 19. https://doi.org/10.1177/11779322251321065

Overton, C. E., Pellis, L., Stage, H. B., Scarabel, F., Burton, J., Fraser, C., Hall, I., House, T. A., Jewell, C., Nurtay, A., Pagani, F., & Lythgoe, K. A. (2022). EpiBeds : Data informed modelling of the COVID-19 hospital burden in England. PLoS Computational Biology, 18(9). https://doi.org/10.1371/journal.pcbi.1010406

Paccalin, M., Gavazzi, G., Berkovitch, Q., Leleu, H., Moreau, R., Ciglia, E., Burlet, N., & Mould-Quevedo, J. F. (2024). Cost-Effectiveness of Adjuvanted Quadrivalent Influenza Vaccine for Adults over 65 in France. Vaccines, 12(6). https://doi.org/10.3390/vaccines12060574

Packer, S., Patrzylas, P., Smith, I., Chen, C., Wensley, A., Nsonwu, O., Dack, K., Turner, C., Anderson, C., Kwiatkowska, R., Oliver, I., Edeghere, O., Fraser, G., & Hughes, G. (2024). COVID-19 cluster surveillance using exposure data collected from routine contact tracing : The genomic validation of a novel informatics-based approach to outbreak detection in England. PLOS Digital Health, 3(4). https://doi.org/10.1371/journal.pdig.0000485

Paduano, S., Facchini, M. C., Borsari, L., D'Alterio, A., Iacuzio, L., Greco, A., Fioretti, E., Creola, G., Kahfian, Z., Zona, S., Bargellini, A., & Filippini, T. (2023). Health surveillance for SARS-CoV-2 : Infection spread and vaccination coverage in the schools of Modena province, Italy. Frontiers in Public Health, 11. https://doi.org/10.3389/fpubh.2023.1240315

Paez, A., Lopez, F. A., Menezes, T., Cavalcanti, R., & Pitta, M. G. da R. (2021). A Spatio-Temporal Analysis of the Environmental Correlates of COVID-19 Incidence in Spain. Geographical Analysis, 53(3). https://doi.org/10.1111/gean.12241

Pagani, G., Conti, F., Giacomelli, A., Oreni, L., Beltrami, M., Pezzati, L., Casalini, G., Rondanin, R., Prina, A., Zagari, A., Rusconi, S., & Galli, M. (2021). Differences in the Prevalence of SARS-CoV-2 Infection and Access to Care between Italians and Non-Italians in a Social-Housing Neighbourhood of Milan, Italy. International Journal of Environmental Research and Public Health, 18(20). https://doi.org/10.3390/ijerph182010621

Pagani, G., Giacomelli, A., Conti, F., Bernacchia, D., Rondanin, R., Prina, A., Scolari, V., Rizzo, A., Beltrami, M., Caimi, C., Gandolfi, C. E., Castaldi, S., Rivieccio, B. A., Buonanno, G., Marano, G., Ottomano, C., Boracchi, P., Biganzoli, E., & Galli, M. (2021). Prevalence of SARS-CoV-2 in an area of unrestricted viral circulation : Mass seroepidemiological screening in Castiglione d'Adda, Italy. PloS One, 16(2). https://doi.org/10.1371/journal.pone.0246513

Paganuzzi, M., Nattino, G., Ghilardi, G. I., Costantino, G., Rossi, C., Cortellaro, F., Cosentini, R., Paglia, S., Migliori, M., Mira, A., Bertolini, G., & Fenice network. (2024). Assessing the heterogeneity of the impact of COVID-19 incidence on all-cause excess mortality among healthcare districts in Lombardy, Italy, to evaluate the local response to the pandemic: An ecological study. BMJ Open, 14(2), e077476. https://doi.org/10.1136/bmjopen-2023-077476

Pageaud, S., Eyraud-Loisel, A., Bertoglio, J.-P., Bienvenüe, A., Leboisne, N., Pothier, C., Rigotti, C., Ponthus, N., Gauchon, R., Gueyffier, F., Vanhems, P., Iwaz, J., Loisel, S., Roy, P., & On Behalf Of The CovDyn Group Covid Dynamics, null. (2022). Predicted Impacts of Booster, Immunity Decline, Vaccination Strategies, and Non-Pharmaceutical Interventions on COVID-19 Outcomes in France. Vaccines, 10(12). https://doi.org/10.3390/vaccines10122033

Paggi, R., Barbiero, A., Manciulli, T., Miftode, A., Tilli, M., Lagi, F., Mencarini, J., Borchi, B., Pozzi, M., Bartalesi, F., Spinicci, M., Martini, L., Coppola, A., Nozzoli, C., Peris, A., Bonizzoli, M., Pieralli, F., Bartoloni, A., & Zammarchi, L. (2023). Characteristics of COVID-19 vaccinated and unvaccinated patients admitted to Careggi University Hospital, Florence, Italy. Internal and Emergency Medicine, 18(3). https://doi.org/10.1007/s11739-023-03231-w

Pagnesi, M., Inciardi, R. M., Lombardi, C. M., Agostoni, P., Ameri, P., Barbieri, L., Bellasi, A., Camporotondo, R., Canale, C., Carubelli, V., Carugo, S., Catagnano, F., Dalla Vecchia, L. A., Danzi, G. B., Di Pasquale, M., Gaudenzi, M., Giovinazzo, S., Gnecchi, M., Guazzi, M., … Metra, M. (2021). Determinants of the protective effect of glucocorticoids on mortality in hospitalized patients with COVID-19 : Insights from the Cardio-COVID-Italy multicenter study. International Journal of Infectious Diseases: IJID: Official Publication of the International Society for Infectious Diseases, 108. https://doi.org/10.1016/j.ijid.2021.05.056

Paireau, J., Charpignon, M.-L., Larrieu, S., Calba, C., Hozé, N., Boëlle, P.-Y., Thiebaut, R., Prague, M., & Cauchemez, S. (2023). Impact of non-pharmaceutical interventions, weather, vaccination, and variants on COVID-19 transmission across departments in France. BMC Infectious Diseases, 23(1). https://doi.org/10.1186/s12879-023-08106-1

Paireau, J., Durand, C., Raimbault, S., Cazaubon, J., Mortamet, G., Viriot, D., Milesi, C., Daudens-Vaysse, E., Ploin, D., Tessier, S., Vanel, N., Chappert, J.-L., Levieux, K., Ollivier, R., Daoudi, J., Coignard, B., Leteurtre, S., Parent-du-Châtelet, I., & Vaux, S. (2024). Nirsevimab Effectiveness Against Cases of Respiratory Syncytial Virus Bronchiolitis Hospitalised in Paediatric Intensive Care Units in France, September 2023-January 2024. Influenza and Other Respiratory Viruses, 18(6). https://doi.org/10.1111/irv.13311

Palamenghi, L., Barello, S., Boccia, S., & Graffigna, G. (2020). Mistrust in biomedical research and vaccine hesitancy : The forefront challenge in the battle against COVID-19 in Italy. European Journal of Epidemiology, 35(8). https://doi.org/10.1007/s10654-020-00675-8

Palandri, L., Rizzi, C., Vandelli, V., Filippini, T., Ghinoi, A., Carrozzi, G., Girolamo, G. D., Morlini, I., Coratza, P., Giovannetti, E., Russo, M., Soldati, M., Righi, E., & DISCOV-19 study group. (2025). Environmental, climatic, socio-economic factors and non-pharmacological interventions : A comprehensive four-domain risk assessment of COVID-19 hospitalization and death in Northern Italy. International Journal of Hygiene and Environmental Health, 263. https://doi.org/10.1016/j.ijheh.2024.114471

Palladino, R., Bollon, J., Ragazzoni, L., & Barone-Adesi, F. (2020). Excess Deaths and Hospital Admissions for COVID-19 Due to a Late Implementation of the Lockdown in Italy. International Journal of Environmental Research and Public Health, 17(16). https://doi.org/10.3390/ijerph17165644

Palladino, R., Mercogliano, M., Fiorilla, C., Frangiosa, A., Iodice, S., Sanduzzi Zamparelli, S., Montella, E., Triassi, M., & Sanduzzi Zamparelli, A. (2022). Association between COVID-19 and Sick Leave for Healthcare Workers in a Large Academic Hospital in Southern Italy : An Observational Study. International Journal of Environmental Research and Public Health, 19(15). https://doi.org/10.3390/ijerph19159670

Palma-García, A., Moreno-Pérez, D., Rivera-Izquierdo, M., Cardero-Rivas, M., Del Diego-Salas, J., & Lorusso, N. (2025). Effectiveness of Influenza Vaccination in Children Aged 6-59 Months during the Inaugural 2022-2023 Season in Andalusia, Spain. The Journal of Pediatrics, 282, 114558. https://doi.org/10.1016/j.jpeds.2025.114558

Palmieri, L., Palmer, K., Lo Noce, C., Meli, P., Giuliano, M., Floridia, M., Tamburo de Bella, M., Piccioli, A., Brusaferro, S., Onder, G., Andrianou, X., Barbariol, P., Bella, A., Bellino, S., Benelli, E., Bertinato, L., Boros, S., Brambilla, G., Calcagnini, G., … Zona, A. (2021). Differences in the clinical characteristics of COVID-19 patients who died in hospital during different phases of the pandemic: National data from Italy. Aging Clinical and Experimental Research, 33(1), 193–199. https://doi.org/10.1007/s40520-020-01764-0

Palmieri, L., Vanacore, N., Donfrancesco, C., Lo Noce, C., Canevelli, M., Punzo, O., Raparelli, V., Pezzotti, P., Riccardo, F., Bella, A., Fabiani, M., D'Ancona, F. P., Vaianella, L., Tiple, D., Colaizzo, E., Palmer, K., Rezza, G., Piccioli, A., Brusaferro, S., … Italian National Institute of Health COVID-19 Mortality Group. (2020). Clinical Characteristics of Hospitalized Individuals Dying With COVID-19 by Age Group in Italy. The Journals of Gerontology. Series A, Biological Sciences and Medical Sciences, 75(9). https://doi.org/10.1093/gerona/glaa146

Palus, D. K., Gołębiewska, M. E., Piątek, O., Majeranowski, A., Owczuk, R., Kuziemski, K., & Stefaniak, T. (2022). Analysing COVID treatment outcomes in dedicated wards at a large university hospital in northern Poland. A result-based observational study. medRxiv, (Palus D.K.; Gołębiewska M.E., margol@gumed.edu.pl; Piątek O.) Faculty of Medicine, Medical University of Gdansk, Smoluchowskiego 17, Gdańsk, Poland. https://doi.org/10.1101/2022.07.07.22277395

Pană, A., Pistol, A., Streinu-Cercel, A., & Ileanu, B.-V. (2020). Burden of influenza in Romania. A retrospective analysis of 2014/15—2018/19 seasons in Romania. Germs, 10(4), 201–209. https://doi.org/10.18683/germs.2020.1206

Panovska-Griffiths, J., Swallow, B., Hinch, R., Cohen, J., Rosenfeld, K., Stuart, R. M., Ferretti, L., Di Lauro, F., Wymant, C., Izzo, A., Waites, W., Viner, R., Bonell, C., Fraser, C., Klein, D., Kerr, C. C., & COVID-19

Genomics UK (COG-UK) Consortium. (2022). Statistical and agent-based modelling of the transmissibility of different SARS-CoV-2 variants in England and impact of different interventions. Philosophical Transactions. Series A, Mathematical, Physical, and Engineering Sciences, 380(2233). https://doi.org/10.1098/rsta.2021.0315

Pariani, E., Amendola, A., Piatti, A., Anselmi, G., Ranghiero, A., Bubba, L., Rosa, A. M., Pellegrinelli, L., Binda, S., Coppola, L., Gramegna, M., & Zanetti, A. (2015). Ten years (2004-2014) of influenza surveillance in Northern Italy. Human Vaccines & Immunotherapeutics, 11(1). https://doi.org/10.4161/hv.35863

Pariani, E., Amendola, A., Ranghiero, A., Anselmi, G., & Zanetti, A. (2013). Surveillance of influenza viruses in the post-pandemic era (2010-2012) in Northern Italy. Human Vaccines & Immunotherapeutics, 9(3), 657–666. https://doi.org/10.4161/hv.23262

Paridans, M., Monseur, J., Donneau, A.-F., Gillain, N., Husson, E., Leclercq, D., Meuris, C., Darcis, G., Moutschen, M., Saegerman, C., Gillet, L., Bureau, F., Guillaume, M., & Pétré, B. (2022). The Dynamic Relationship between the Intention and Final Decision for the COVID-19 Booster : A Study among Students and Staff at the University of Liège, Belgium. Vaccines, 10(9). https://doi.org/10.3390/vaccines10091485

Paris, S., Inciardi, R. M., Lombardi, C. M., Tomasoni, D., Ameri, P., Carubelli, V., Agostoni, P., Canale, C., Carugo, S., Danzi, G., Di Pasquale, M., Sarullo, F., La Rovere, M. T., Mortara, A., Piepoli, M., Porto, I., Sinagra, G., Volterrani, M., Gnecchi, M., … Metra, M. (2021). Implications of atrial fibrillation on the clinical course and outcomes of hospitalized COVID-19 patients : Results of the Cardio-COVID-Italy multicentre study. Europace: European Pacing, Arrhythmias, and Cardiac Electrophysiology: Journal of the Working Groups on Cardiac Pacing, Arrhythmias, and Cardiac Cellular Electrophysiology of the European Society of Cardiology, 23(10). https://doi.org/10.1093/europace/euab146

Parola, F., Brach Del Prever, A., Deut, V., Costagliola, G., Guidi, C., Ragusa, N., Tuscano, A., Timeus, F., & Berger, M. (2024). Impact of SARS-CoV-2 Pandemic and Lockdown on the HRSV Circulation: Experience of Three Spoke Hospitals in Northern Italy. Viruses, 16(2), 230. https://doi.org/10.3390/v16020230

Pascucci, D., Grossi, A., Lontano, A., Marziali, E., Nurchis, M. C., Grassi, V. M., Raponi, M., Vetrugno, G., Capelli, G., Calabrò, G. E., Staiti, D., Sanguinetti, M., Damiani, G., & Laurenti, P. (2022). Risk of Infection and Duration of Protection after the Booster Dose of the Anti-SARS-CoV-2 Vaccine BNT162b2 among Healthcare Workers in a Large Teaching Hospital in Italy : Results of an Observational Study. Vaccines, 11(1). https://doi.org/10.3390/vaccines11010025

Passamonti, F., Cattaneo, C., Arcaini, L., Bruna, R., Cavo, M., Merli, F., Angelucci, E., Krampera, M., Cairoli, R., Della Porta, M. G., Fracchiolla, N., Ladetto, M., Gambacorti Passerini, C., Salvini, M., Marchetti, M., Lemoli, R., Molteni, A., Busca, A., Cuneo, A., … ITA-HEMA-COV Investigators. (2020). Clinical characteristics and risk factors associated with COVID-19 severity in patients with haematological malignancies in Italy : A retrospective, multicentre, cohort study. The Lancet. Haematology, 7(10). https://doi.org/10.1016/S2352-3026(20)30251-9

Patel, V., Levick, B., Boult, S., Gibbons, D. C., Drysdale, M., Lloyd, E. J., Singh, M., & Birch, H. J. (2024). Characteristics and outcomes of COVID-19 patients presumed to be treated with sotrovimab in NHS hospitals in England. BMC Infectious Diseases, 24(1). https://doi.org/10.1186/s12879-024-09311-2

Patel, V., Yarwood, M. J., Levick, B., Gibbons, D. C., Drysdale, M., Kerr, W., Watkins, J. D., Young, S., Pierce, B. F., Lloyd, E. J., Birch, H. J., Kamalati, T., & Brett, S. J. (2022). Characteristics and outcomes of patients with COVID-19 at high-risk of disease progression receiving sotrovimab, oral antivirals or no treatment in England. medRxiv, (Patel V.; Gibbons D.C.; Drysdale M.; Kerr W.; Lloyd E.J.; Birch H.J.) GSK, Middlesex, United Kingdom. https://doi.org/10.1101/2022.11.28.22282808

Paternoster, M., Masse, S., van der Werf, S., Lina, B., Levy-Bruhl, D., Villechenaud, N., Valette, M., Behillil, S., Bernard-Stoecklin, S., Guerrisi, C., Blanchon, T., Falchi, A., Hanslik, T., Turbelin, C., & Souty, C. (2021). Estimation of influenza-attributable burden in primary care from season 2014/2015 to 2018/2019, France. European Journal of Clinical Microbiology & Infectious Diseases: Official Publication of the European Society of Clinical Microbiology, 40(6). https://doi.org/10.1007/s10096-021-04161-1

Paterson, P., Chantler, T., & Larson, H. J. (2018). Reasons for non-vaccination : Parental vaccine hesitancy and the childhood influenza vaccination school pilot programme in England. Vaccine, 36(36). https://doi.org/10.1016/j.vaccine.2017.08.016

Patone, M., Thomas, K., Hatch, R., Tan, P. S., Coupland, C., Liao, W., Mouncey, P., Harrison, D., Rowan, K., Horby, P., Watkinson, P., & Hippisley-Cox, J. (2021). Mortality and critical care unit admission associated with the SARS-CoV-2 lineage B.1.1.7 in England : An observational cohort study. The Lancet. Infectious Diseases, 21(11). https://doi.org/10.1016/S1473-3099(21)00318-2

Pebody, R. G., Green, H. K., Andrews, N., Boddington, N. L., Zhao, H., Yonova, I., Ellis, J., Steinberger, S., Donati, M., Elliot, A. J., Hughes, H. E., Pathirannehelage, S., Mullett, D., Smith, G. E., de Lusignan, S., & Zambon, M. (2015). Uptake and impact of vaccinating school age children against influenza during a season with circulation of drifted influenza A and B strains, England, 2014/15. Euro Surveillance: Bulletin Europeen Sur Les Maladies Transmissibles = European Communicable Disease Bulletin, 20(39). https://doi.org/10.2807/1560-7917.ES.2015.20.39.30029

Pebody, R. G., Green, H. K., Andrews, N., Zhao, H., Boddington, N., Bawa, Z., Durnall, H., Singh, N., Sunderland, A., Letley, L., Ellis, J., Elliot, A. J., Donati, M., Smith, G. E., de Lusignan, S., & Zambon, M. (2014). Uptake and impact of a new live attenuated influenza vaccine programme in England : Early results of a pilot in primary school-age children, 2013/14 influenza season. Euro Surveillance: Bulletin Europeen Sur Les Maladies Transmissibles = European Communicable Disease Bulletin, 19(22). https://doi.org/10.2807/1560-7917.es2014.19.22.20823

Pebody, R. G., Green, H. K., Warburton, F., Sinnathamby, M., Ellis, J., Mølbak, K., Nielsen, J., de Lusignan, S., & Andrews, N. (2018). Significant spike in excess mortality in England in winter 2014/15—Influenza the likely culprit. Epidemiology and Infection, 146(9). https://doi.org/10.1017/S0950268818001152

Pebody, R. G., Zhao, H., Whitaker, H. J., Ellis, J., Donati, M., Zambon, M., & Andrews, N. (2020). Effectiveness of influenza vaccine in children in preventing influenza associated hospitalisation, 2018/19, England. Vaccine, 38(2). https://doi.org/10.1016/j.vaccine.2019.10.035

Pebody, R., Andrews, N., Waight, P., Malkani, R., McCartney, C., Ellis, J., & Miller, E. (2011). No effect of 2008/09 seasonal influenza vaccination on the risk of pandemic H1N1 2009 influenza infection in England. Vaccine, 29(14), 2613–2618. https://doi.org/10.1016/j.vaccine.2011.01.046

Pebody, R., Sile, B., Warburton, F., Sinnathamby, M., Tsang, C., Zhao, H., Ellis, J., & Andrews, N. (2017). Live attenuated influenza vaccine effectiveness against hospitalisation due to laboratory-confirmed influenza in children two to six years of age in England in the 2015/16 season. Euro Surveillance: Bulletin Europeen Sur Les Maladies Transmissibles = European Communicable Disease Bulletin, 22(4). https://doi.org/10.2807/1560-7917.ES.2017.22.4.30450

Pebody, R., Whitaker, H., Zhao, H., Andrews, N., Ellis, J., Donati, M., & Zambon, M. (2020). Protection provided by influenza vaccine against influenza-related hospitalisation in ≥65 year olds : Early experience of introduction of a newly licensed adjuvanted vaccine in England in 2018/19. Vaccine, 38(2). https://doi.org/10.1016/j.vaccine.2019.10.032

Pecks, U., Bohlmann, M. K., Andresen, K., Büchel, J., Bartmann, C., Sitter, M., Tihon, A., Kranke, P., Wöckel, A., Hollweck, R., Dressler-Steinbach, I., Gruessner, S., Gruber, T. M., Eichinger, T., Manz, J., Ruehl, I. M., Lihs, A., Biermann, A.-L., Bauerfeind, L. M., … Rath, W. (2025). SARS-CoV-2 infection in pregnant women and incidence of thromboembolic disease: An analysis of the Covid-19-Related Obstetric and Neonatal Outcome Study (CRONOS) in Germany. Archives of Gynecology and Obstetrics, 311(6), 1667–1682. https://doi.org/10.1007/s00404-025-08007-5

Pelat, C., Bonmarin, I., Ruello, M., Fouillet, A., Caserio-Schönemann, C., Levy-Bruhl, D., Le Strat, Y., & Regional Influenza study group. (2017). Improving regional influenza surveillance through a combination of automated outbreak detection methods : The 2015/16 season in France. Euro Surveillance: Bulletin Europeen Sur Les Maladies Transmissibles = European Communicable Disease Bulletin, 22(32). https://doi.org/10.2807/1560-7917.ES.2017.22.32.30593

Pelat, C., Falchi, A., Carrat, F., Mosnier, A., Bonmarin, I., Turbelin, C., Vaux, S., van der Werf, S., Cohen, J. M., Lina, B., Blanchon, T., & Hanslik, T. (2011). Field effectiveness of pandemic and 2009-2010 seasonal vaccines against 2009-2010 A(H1N1) influenza: Estimations from surveillance data in France. PloS One, 6(5), e19621. https://doi.org/10.1371/journal.pone.0019621

Pelat, C., Lasserre, A., Xavier, A., Turbelin, C., Blanchon, T., & Hanslik, T. (2013). Hospitalization of influenza-like illness patients recommended by general practitioners in France between 1997 and 2010. Influenza and Other Respiratory Viruses, 7(1). https://doi.org/10.1111/j.1750-2659.2012.00356.x

Pelullo, C. P., Tortoriello, P., Torsiello, L., Lombardi, C., Napolitano, F., & Di Giuseppe, G. (2022). Preventive Measures for SARS-CoV-2 in the Workplace and Vaccine Acceptance : Assessment of Knowledge, Attitudes and Behaviors of Workers in Southern Italy. Vaccines, 10(11). https://doi.org/10.3390/vaccines10111872

Peñalvo, J. L., Genbrugge, E., Mertens, E., Sagastume, D., van der Sande, M. A. B., Widdowson, M.-A., Van Beckhoven, D., & Belgian Collaborative Group on COVID-19 Hospital Surveillance. (2021). Insights into the association of ACEIs/ARBs use and COVID-19 prognosis : A multistate modelling study of nationwide hospital surveillance data from Belgium. BMJ Open, 11(9). https://doi.org/10.1136/bmjopen-2021-053393

Penot, P., Chateauneuf, J., Auperin, I., Cordel, H., Letembet, V.-A., Bottero, J., & Cailhol, J. (2023). Socioeconomic impact of the COVID-19 crisis and early perceptions of COVID-19 vaccines among immigrant and nonimmigrant people living with HIV followed up in public hospitals in Seine-Saint-Denis, France. PloS One, 18(10). https://doi.org/10.1371/journal.pone.0276038

Pepe, A., Valitutti, F., Veneruso, D., Bove, M., De Anseris, A. G. E., Nazzaro, L., Pisano, P., Melis, D., & Mandato, C. (2022). Severe Acute Respiratory Syndrome Coronavirus 2 (SARS-CoV-2) antigen detection in the Emergency Department : Data from a pediatric cohort during the fourth COVID-19 wave in Italy. Italian Journal of Pediatrics, 48(1). https://doi.org/10.1186/s13052-022-01343-1

Percivalle, E., Cambiè, G., Cassaniti, I., Nepita, E. V., Maserati, R., Ferrari, A., Di Martino, R., Isernia, P., Mojoli, F., Bruno, R., Tirani, M., Cereda, D., Nicora, C., Lombardo, M., & Baldanti, F. (2020). Prevalence of SARS-CoV-2 specific neutralising antibodies in blood donors from the Lodi Red Zone in Lombardy, Italy, as at 06 April 2020. Euro Surveillance: Bulletin Europeen Sur Les Maladies Transmissibles = European Communicable Disease Bulletin, 25(24). https://doi.org/10.2807/1560-7917.ES.2020.25.24.2001031

Peregrina-Rivas, J. A., Fernández-Reyes, D., de Salazar-González, A., García-García, F., Montero-Alonso, M. Á., Hernández-Quero, J., & Guirao-Arrabal, E. (2025). Post-COVID-19 tuberculosis in southeastern Spain : Incidence, risk factors and the role of latent tuberculosis infection screening. Revista Espanola De Quimioterapia: Publicacion Oficial De La Sociedad Espanola De Quimioterapia, 38(2). https://doi.org/10.37201/req/111.2024

Pérez-Ciordia, I., Guillén-Grima, F., Aguinaga, I., & Brugos, A. (2016). Vaccination coverage and factors influencing the attitude of influenza vaccination in occupational groups (firefighters, teachers, police and nursing homes caregivers) in Navarra (Spain). Vacunas, 17(1). Embase. https://doi.org/10.1016/j.vacun.2016.03.003

Pérez-de-Llano, L., Romay-Lema, E. M., Baloira-Villar, A., Anchorena, C., Torres-Durán, M. L., Sousa, A., Corbacho-Abelaira, D., Paz-Ferrin, J., Diego-Roza, C., Vilariño-Maneiro, L., Marcos, P. J., Montero-Martínez, C., de la Iglesia-Martínez, F., Riveiro-Blanco, V., Rodríguez-Núñez, N., Abal-Arca, J., Bustillo-Casado, M., & Golpe, R. (2021). COVID-19 pneumonia in Galicia (Spain) : Impact of prognostic factors and therapies on mortality and need for mechanical ventilation. PloS One, 16(6). https://doi.org/10.1371/journal.pone.0253465

Pérez-García, F., Pérez-Zapata, A., Arcos, N., De la Mata, M., Ortiz, M., Simón, E., Hervás Fernández, I., González Ventosa, V., Muñoz Monte, M., González Arroyo, J., Pérez-Tanoira, R., & Cuadros-González, J. (2021). Severe acute respiratory coronavirus virus 2 (SARS-CoV-2) infection among hospital workers in a severely affected institution in Madrid, Spain : A surveillance cross-sectional study. Infection Control and Hospital Epidemiology, 42(7). https://doi.org/10.1017/ice.2020.1303

Pérez-Gilaberte, J. B., Martín-Iranzo, N., Aguilera, J., Almenara-Blasco, M., de Gálvez, M. V., & Gilaberte, Y. (2023). Correlation between UV Index, Temperature and Humidity with Respect to Incidence and Severity of COVID 19 in Spain. International Journal of Environmental Research and Public Health, 20(3). https://doi.org/10.3390/ijerph20031973

Pérez-Gimeno, G., Mazagatos, C., Lorusso, N., Basile, L., Martínez-Pino, I., Corpas Burgos, F., Batalla Rebolla, N., Rumayor Zarzuelo, M. B., Andreu Ivorra, B., Giménez Duran, J., Castrillejo, D., Guiu Cañete, I., Huerta Huerta, M., García Becerril, M., Ramos Marín, V., Casas, I., Pozo, F., Monge, S., SiVIRA group, & Members of the SiVIRA sentinel surveillance study group. (2024). Effectiveness of influenza vaccines in children aged 6 to 59 months : A test-negative case-control study at primary care and hospital level, Spain 2023/24. Euro Surveillance: Bulletin Europeen Sur Les Maladies Transmissibles = European Communicable Disease Bulletin, 29(40). https://doi.org/10.2807/1560-7917.ES.2024.29.40.2400618

Pérez-González, A., Araújo-Ameijeiras, A., Fernández-Villar, A., Crespo, M., Poveda, E., & Cohort COVID-19 Galicia Hlth Res I. (2022). Long COVID in hospitalized and non-hospitalized patients in a large cohort in Northwest Spain, a prospective cohort study. SCIENTIFIC REPORTS, 12(1). https://doi.org/10.1038/s41598-022-07414-x

Pérez-Rubio, A., & Eiros, J. M. (2018). [Economic and Health impact of influenza vaccination with adjuvant MF59 in population over 64 years in Spain]. Revista Espanola De Quimioterapia: Publicacion Oficial De La Sociedad Espanola De Quimioterapia, 31(1). http://www.ncbi.nlm.nih.gov/pubmed/29355006

Perez-Rubio, A., Flores, R., Aragon, J. R., Sanchez, J., Marquez-Peláez, S., Alvarez, P., Muriel, A. O., & Mould-Quevedo, J. (2025). Cost-Effectiveness of Adjuvanted Influenza Vaccine Compared with Standard and High-Dose Influenza Vaccines for Persons Aged ≥50 Years in Spain. Vaccines, 13(3), 323. https://doi.org/10.3390/vaccines13030323

Perico, L., Tomasoni, S., Peracchi, T., Perna, A., Pezzotta, A., Remuzzi, G., & Benigni, A. (2020). COVID-19 and lombardy: TESTing the impact of the first wave of the pandemic: The prevalence of SARS-CoV-2 infection in northern Italy. EBioMedicine, 61. https://doi.org/10.1016/j.ebiom.2020.103069

Perone, G. (2022). Comparison of ARIMA, ETS, NNAR, TBATS and hybrid models to forecast the second wave of COVID-19 hospitalizations in Italy. The European Journal of Health Economics: HEPAC: Health Economics in Prevention and Care, 23(6). https://doi.org/10.1007/s10198-021-01347-4

Perramon, A., Soriano-Arandes, A., Pino, D., Lazcano, U., Andrés, C., Català, M., Gatell, A., Carulla, M., Canadell, D., Ricós, G., Riera-Bosch, M. T., Burgaya, S., Salvadó, O., Cantero, J., Vilà, M., Poblet, M., Sánchez, A., Ristol, A. M., Serrano, P., … Soler-Palacin, P. (2021). Schools as a Framework for COVID-19 Epidemiological Surveillance of Children in Catalonia, Spain : A Population-Based Study. Frontiers in Pediatrics, 9. https://doi.org/10.3389/fped.2021.754744

Perramon-Malavez, A., de Rioja, V. L., Coma, E., Hermosilla, E., Fina, F., Martínez-Marcos, M., Mendioroz, J., Cabezas, C., Montañola-Sales, C., Prats, C., & Soriano-Arandes, A. (2024). Introduction of nirsevimab in Catalonia, Spain : Description of the incidence of bronchiolitis and respiratory syncytial virus in the 2023/2024 season. European Journal of Pediatrics, 183(12). https://doi.org/10.1007/s00431-024-05779-x

Perramon-Malavez, A., Hermosilla, E., Coma, E., Fina, F., Reñé, A., Martínez-Marcos, M., Mendioroz, J., Prats, C., Soriano-Arandes, A., & Cabezas, C. (2025). Effectiveness of Nirsevimab Immunoprophylaxis Against Respiratory Syncytial Virus-related Outcomes in Hospital Care Settings : A Seasonal Cohort Study of Infants in Catalonia (Spain). The Pediatric Infectious Disease Journal. https://doi.org/10.1097/INF.0000000000004672

Perrotta, D., Bella, A., Rizzo, C., & Paolotti, D. (2017). Participatory Online Surveillance as a Supplementary Tool to Sentinel Doctors for Influenza-Like Illness Surveillance in Italy. PloS One, 12(1). https://doi.org/10.1371/journal.pone.0169801

Perrotta, D., Tizzoni, M., Paolotti, D., & ACM. (2017). Using Participatory Web-based Surveillance Data to Improve Seasonal Influenza Forecasting in Italy. PROCEEDINGS OF THE 26TH INTERNATIONAL CONFERENCE ON WORLD WIDE WEB (WWW'17). https://doi.org/10.1145/3038912.3052670

Pertile, R., Battistella, C., De Nisi, M., Zuccali, M. G., Mantovani, W., & Moretti, F. (2023). The COVID-19 vaccination in 4,772 pregnant women in the province of Trento (North-East Italy). Characteristics of vaccinated women. Epidemiologia E Prevenzione, 47(6). https://doi.org/10.19191/EP23.6.A636.082

Perumal, N., Steffen, A., Ullrich, A., & Siedler, A. (2022). Impact of COVID-19 immunisation on COVID-19 incidence, hospitalisations, and deaths by age group in Germany from December 2020 to October 2021. Vaccine, 40(21). https://doi.org/10.1016/j.vaccine.2022.04.002

Peter, R. S., Nieters, A., Kräusslich, H.-G., Brockmann, S. O., Göpel, S., Kindle, G., Merle, U., Steinacker, J. M., Rothenbacher, D., Kern, W. V., August, D., Bauer, C., Blankenhorn, B., Bopp-Haas, U., Bunk, S., Deibert, P., Dietz, A., Friedmann-Bette, B., Giesen, R., … Wolfers, K. (2022). Prevalence, determinants, and impact on general health and working capacity of post-acute sequelae of COVID-19 six to 12 months after infection : A population-based retrospective cohort study from southern Germany. medRxiv, (Peter R.S.; Rothenbacher D.) Institute of Epidemiology and Medical Biometry, Ulm University, Ulm, Germany. https://doi.org/10.1101/2022.03.14.22272316

Peters, C., Dulon, M., Westermann, C., Kozak, A., & Nienhaus, A. (2022). Long-Term Effects of COVID-19 on Workers in Health and Social Services in Germany. International Journal of Environmental Research and Public Health, 19(12). https://doi.org/10.3390/ijerph19126983

Petersen, J., Mülder, L. M., Kegel, P., Röthke, N., Wiegand, H. F., Lieb, K., Walter, H., Bröcker, A.-L., Liebe, S., Tüscher, O., Pfennig, A., Maicher, B., Hellwig, S., Padberg, F., Adorjan, K., Unterecker, S., Wessels, P., Rose, D.-M., & Beutel, M. E. (2022). [Willingness to get vaccinated among hospital staff in Germany : What is the role of COVID-19 conspiracy assumptions?]. Bundesgesundheitsblatt, Gesundheitsforschung, Gesundheitsschutz, 65(11). https://doi.org/10.1007/s00103-022-03593-0

Petrelli, A., & Di Napoli, A. (2022). [The impact of COVID-19 on the immigrant population in Italy. Context, methodology and synthesis of the main evidence from the project of the National Institute for Health, Migration and Poverty (INMP) and Italian Regions]. Epidemiologia E Prevenzione, 46(4). https://doi.org/10.19191/EP22.4S1.051

Petrovici, N., Belbe, S. Ștefana, Mare, C. C., & Cotoi, C. C. (2023). Hybrid health regimes: Access to primary care physicians and COVID-19 vaccine uptake across municipalities in Romania. Social Science & Medicine (1982), 337, 116305. https://doi.org/10.1016/j.socscimed.2023.116305

Petrusevich, D. A. (2020). Clustering of Covid-19 morbidity cases in Germany. IOP Conference Series. Materials Science and Engineering, 862(4). https://doi.org/10.1088/1757-899X/862/4/042037

Phijffer, E. W. E. M., Wildenbeest, J. G., Brouwer, C. N. M., de Hoog, M., Kneyber, M. C. J., Maebe, S., Nusmeier, A., Riedijk, M. A., Wösten-van Asperen, R. M., van Woensel, J. B. M., Bont, L. J., & Frederix, G. J. W. (2024). Healthcare costs related to respiratory syncytial virus in paediatric intensive care units in the Netherlands : A nationwide prospective observational study (the BRICK study). The Lancet Regional Health. Europe, 43. https://doi.org/10.1016/j.lanepe.2024.100965

Piazza, M., Amicizia, D., Marchini, F., Astengo, M., Grammatico, F., Battaglini, A., Sticchi, C., Paganino, C., Lavieri, R., Andreoli, G., Orsi, A., Icardi, G., & Ansaldi, F. (2022). Who Is at Higher Risk of SARS-CoV-2 Reinfection ? Results from a Northern Region of Italy. VACCINES, 10(11). https://doi.org/10.3390/vaccines10111885

Picard, G., Fournier, L., Maisa, A., Grolhier, C., Chent, S., Huchet-Kervalla, C., Sudour, J., Pretet, M., Josset, L., Behillil, S., Schaeffer, J., Laboratory group, COVID-19 Investigation Group, Members of the Laboratory group, & Members of the COVID-19 Investigation group. (2023). Emergence, spread and characterisation of the SARS-CoV-2 variant B.1.640 circulating in France, October 2021 to February 2022. Euro Surveillance: Bulletin Europeen Sur Les Maladies Transmissibles = European Communicable Disease Bulletin, 28(22). https://doi.org/10.2807/1560-7917.ES.2023.28.22.2200671

Piepenburg, S. M., Maslarska, M., Kaier, K., Mühlen, C. von Z., Westermann, D., & Hehrlein, C. (2024). The Impact of COVID-19 on Mortality and Clinical Characteristics in Hospitalized Patients With Peripheral Artery Disease in the Year 2020 in Germany. Angiology. https://doi.org/10.1177/00033197241251905

Pierangeli, A., Trotta, D., Scagnolari, C., Ferreri, M. L., Nicolai, A., Midulla, F., Marinelli, K., Antonelli, G., & Bagnarelli, P. (2014). Rapid spread of the novel respiratory syncytial virus A ON1 genotype, central Italy, 2011 to 2013. Euro Surveillance: Bulletin Europeen Sur Les Maladies Transmissibles = European Communicable Disease Bulletin, 19(26). https://doi.org/10.2807/1560-7917.es2014.19.26.20843

Piernas, C., Patone, M., Astbury, N. M., Gao, M., Sheikh, A., Khunti, K., Shankar-Hari, M., Dixon, S., Coupland, C., Aveyard, P., Hippisley-Cox, J., & Jebb, S. A. (2022). Associations of BMI with COVID-19 vaccine uptake, vaccine effectiveness, and risk of severe COVID-19 outcomes after vaccination in England : A population-based cohort study. The Lancet. Diabetes & Endocrinology, 10(8). https://doi.org/10.1016/S2213-8587(22)00158-9

Pierobon, S., Braggion, M., Fedeli, U., Nordio, M., Basso, C., & Zorzi, M. (2022). Impact of vaccination on the spread of SARS-CoV-2 infection in north-east Italy nursing homes. A propensity score and risk analysis. Age and Ageing, 51(1). https://doi.org/10.1093/ageing/afab224

Pierson-Marchandise, M., Castelain, S., Chevalier, C., Brochot, E., Schmit, J.-L., Diouf, M., Ganry, O., & Gignon, M. (2022). Hospital-wide SARS-CoV-2 antibody screening of 4840 staff members in a University Medical Center in France : A cross-sectional study. BMJ Open, 12(5). https://doi.org/10.1136/bmjopen-2020-047010

Pietrzak, Ł., Polok, K., Halik, R., Szuster-Ciesielska, A., & Szczeklik, W. (2023). Effectiveness of the BNT162b2 vaccine in preventing COVID-19-associated deaths in Poland. Polish Archives of Internal Medicine, 133(9). https://doi.org/10.20452/pamw.16453

Pijpers, J., van Roon, A., van Roekel, C., Labuschagne, L., Smagge, B., Ferreira, J. A., de Melker, H., & Hahné, S. (2023). Determinants of COVID-19 Vaccine Uptake in The Netherlands : A Nationwide Registry-Based Study. Vaccines, 11(9). https://doi.org/10.3390/vaccines11091409

Piltch-Loeb, R., Harriman, N. W., Healey, J., Bonetti, M., Toffolutti, V., Testa, M. A., Su, M., & Savoia, E. (2021). COVID-19 Vaccine Concerns about Safety, Effectiveness, and Policies in the United States, Canada, Sweden, and Italy among Unvaccinated Individuals. Vaccines, 9(10). https://doi.org/10.3390/vaccines9101138

Pilz, M., Küfer, K.-H., Mohring, J., Münch, J., Wlazło, J., & Leithäuser, N. (2023). Statistical analysis of three data sources for Covid-19 monitoring in Rhineland-Palatinate, Germany. medRxiv, (Pilz M., maximilian.pilz@itwm.fraunhofer.de; Küfer K.-H.; Mohring J.; Münch J.; Wlazło J.; Leithäuser N.) Fraunhofer Institute for Industrial Mathematics, Germany. https://doi.org/10.1101/2023.09.21.23295894

Pimenoff, V. N., Björnstedt, M., & Dillner, J. (2021). Severe features during outbreak but low mortality observed immediately before and after a March-May 2020 COVID-19 outbreak in Stockholm, Sweden. International Journal of Infectious Diseases: IJID: Official Publication of the International Society for Infectious Diseases, 110. https://doi.org/10.1016/j.ijid.2021.08.005

Pineda-Moncusí, M., Allery, F., Abbasizanjani, H., Powell, D., Prats-Uribe, A., Thygesen, J. H., Wood, A., Tomlinson, C., Banerjee, A., Akbari, A., Delmestri, A., Coates, L. C., Denaxas, S., Khunti, K., Collins, G., Prieto-Alhambra, D., Khalid, S., & CVD-COVID-UK/COVID-IMPACT Consortium. (2025). Ethnic disparities in COVID-19 mortality and cardiovascular disease in England and Wales between 2020-2022. Nature Communications, 16(1), 6059. https://doi.org/10.1038/s41467-025-59951-4

Pini, A., Merk, H., Carnahan, A., Galanis, I., VAN Straten, E., Danis, K., Edelstein, M., & Wallensten, A. (2017). High added value of a population-based participatory surveillance system for community acute gastrointestinal, respiratory and influenza-like illnesses in Sweden, 2013-2014 using the web. Epidemiology and Infection, 145(6). https://doi.org/10.1017/S0950268816003290

Pinkas, J., Jankowski, M., Szumowski, Ł., Lusawa, A., Zgliczyński, W. S., Raciborski, F., Wierzba, W., & Gujski, M. (2020). Public Health Interventions to Mitigate Early Spread of SARS-CoV-2 in Poland. Medical Science Monitor: International Medical Journal of Experimental and Clinical Research, 26. https://doi.org/10.12659/MSM.924730

Pintea-Simon, I.-A., Bancu, L., Mare, A. D., Ciurea, C. N., Toma, F., Brukner, M. C., Văsieșiu, A.-M., & Man, A. (2024). Secondary Bacterial Infections in Critically Ill COVID-19 Patients Admitted in the Intensive Care Unit of a Tertiary Hospital in Romania. Journal of Clinical Medicine, 13(20), 6201. https://doi.org/10.3390/jcm13206201

Piralla, A., Lunghi, G., Ruggiero, L., Girello, A., Bianchini, S., Rovida, F., Caimmi, S., Marseglia, G. L., Principi, N., Baldanti, F., & Esposito, S. (2017). Molecular epidemiology of influenza B virus among

hospitalized pediatric patients in Northern Italy during the 2015-16 season. PloS One, 12(10). https://doi.org/10.1371/journal.pone.0185893

Piralla, A., Mojoli, F., Pellegrinelli, L., Ceriotti, F., Valzano, A., Grasselli, G., Gismondo, M. R., Micheli, V., Castelli, A., Farina, C., Arosio, M., Lorini, F. L., Fanti, D., Busni, A., Laratta, M., Maggi, F., Novazzi, F., Cabrini, L., Callegaro, A. P., … Pariani, E. (2023). Impact of SARS-CoV-2 Omicron and Delta variants in patients requiring intensive care unit (ICU) admission for COVID-19, Northern Italy, December 2021 to January 2022. Respiratory Medicine and Research, 83. https://doi.org/10.1016/j.resmer.2023.100990

Pistellato, I., Fonzo, M., Calzavara, A., Sorrentino, P., Selle, V., Sbrogiò, L. G., & Bertoncello, C. (2023). The spread of SARS-CoV-2 at school through the different pandemic waves: A population-based study in Italy. European Journal of Pediatrics, 182(1), 173–179. https://doi.org/10.1007/s00431-022-04654-x

Pitigoi, D., Ivanciuc, A. E., Necula, G., Lupulescu, E., Alexandrescu, V., & Savulescu, C. (2012). Influenza vaccine effectiveness to prevent medically attended laboratory confirmed influenza during season 2010-2011 in Romania: A case control study. REVISTA ROMANA DE MEDICINA DE LABORATOR, 20(2), 127–134.

Piţigoi, D., Lupulescu, E., Necula, G., Mihai, M. E., Cherciu, C. M., Tecu, C., & Alexandrescu, V. (2014). Influenza viruses circulation and the effectiveness of seasonal influenza vaccine in Romania during the season 2013-2014. BMC Infectious Diseases, 14(7).

Pitigoi, D., Necula, G., Alexandrescu, V., Mihai, M. E., Cherciu, C. M., Tecu, C., Popovici, O., Popescu, R., & Lupulescu, E. (2015). Circulating influenza viruses and the effectiveness of seasonal influenza vaccine in Romania, season 2012-2013. REVISTA ROMANA DE MEDICINA DE LABORATOR, 23(1), 9–20. https://doi.org/10.1515/rrlm-2015-0007

Pițigoi, D., Nițescu, M., Streinu-Cercel, A., Bacruban, R., Ivanciuc, A. E., Lazăr, M., Cherciu, C. M., Crăciun, M. D., Aramă, V., Streinu-Cercel, A., & Săndulescu, O. (2019). Characteristics of influenza in elderly patients with and without diabetes, hospitalized for severe acute respiratory infection in a tertiary care hospital from Bucharest Romania—A three-year prospective epidemiological surveillance study. Germs, 9(3), 142–147. https://doi.org/10.18683/germs.2019.1169

Pițigoi, D., Streinu-Cercel, A., Ivanciuc, A. E., Lazãr, M., Cherciu, C. M., Mihai, M. E., Nițescu, M., Aramă, V., Crăciun, M. D., Streinu-Cercel, A., & Săndulescu, O. (2020). Surveillance of medically-attended influenza in elderly patients from Romania-data from three consecutive influenza seasons (2015/16, 2016/17, and 2017/18). Influenza and Other Respiratory Viruses, 14(5), 530–540. https://doi.org/10.1111/irv.12752

Pitman, R. J., Melegaro, A., Gelb, D., Siddiqui, M. R., Gay, N. J., & Edmunds, W. J. (2007). Assessing the burden of influenza and other respiratory infections in England and Wales. The Journal of Infection, 54(6). https://doi.org/10.1016/j.jinf.2006.09.017

Pitman, R. J., Nagy, L. D., & Sculpher, M. J. (2013). Cost-effectiveness of childhood influenza vaccination in England and Wales : Results from a dynamic transmission model. Vaccine, 31(6). https://doi.org/10.1016/j.vaccine.2012.12.010

Pitman, R. J., White, L. J., & Sculpher, M. (2012). Estimating the clinical impact of introducing paediatric influenza vaccination in England and Wales. Vaccine, 30(6). https://doi.org/10.1016/j.vaccine.2011.11.106

Pitrelli, A. (2016). Introduction of a quadrivalent influenza vaccine in Italy : A budget impact analysis. Journal of Preventive Medicine and Hygiene, 57(1). http://www.ncbi.nlm.nih.gov/pubmed/27346938

Piubelli, C., Deiana, M., Pomari, E., Silva, R., Bisoffi, Z., Formenti, F., Perandin, F., Gobbi, F., & Buonfrate, D. (2021). Overall decrease in SARS-CoV-2 viral load and reduction in clinical burden : The experience of a hospital in northern Italy. Clinical Microbiology and Infection: The Official Publication of the European Society of Clinical Microbiology and Infectious Diseases, 27(1). https://doi.org/10.1016/j.cmi.2020.10.006

Pivette, M., Auvigne, V., Guérin, P., & Mueller, J. E. (2017). [Real-time monitoring of anti-influenza vaccination in the 65 and over population in France based on vaccine sales]. Revue D'epidemiologie Et De Sante Publique, 65(2). https://doi.org/10.1016/j.respe.2016.12.025

Pivette, M., Nicolay, N., de Lauzun, V., & Hubert, B. (2020). Characteristics of hospitalizations with an influenza diagnosis, France, 2012-2013 to 2016-2017 influenza seasons. Influenza and Other Respiratory Viruses, 14(3). https://doi.org/10.1111/irv.12719

Pivetti, M., Melotti, G., Bonomo, M., & Hakoköngäs, E. (2021). Conspiracy Beliefs and Acceptance of COVID-Vaccine : An Exploratory Study in Italy. SOCIAL SCIENCES-BASEL, 10(3). https://doi.org/10.3390/socsci10030108

Pivetti, M., Paleari, F., Barni, D., Russo, C., & Di Battista, S. (2023). Self-transcendence values, vaccine hesitancy, and COVID-19 vaccination : Some results from Italy. SOCIAL INFLUENCE, 18(1). https://doi.org/10.1080/15534510.2023.2261632

Pizzuti, C., Socievole, A., Prasse, B., & Van Mieghem, P. (2020). Network-based prediction of COVID-19 epidemic spreading in Italy. APPLIED NETWORK SCIENCE, 5(1). https://doi.org/10.1007/s41109-020-00333-8

Plans Rubió, P., Jambrina, A. M., Rius, P., Carmona, G., Rabanal, M., & Gironès, M. (2022). High Influenza Vaccine Effectiveness and Absence of Increased Influenza-like-Illness Epidemic Activity in the 2021-2022 Influenza Season in Catalonia (Spain) Based on Surveillance Data Collected by Sentinel Pharmacies. Vaccines, 10(12). https://doi.org/10.3390/vaccines10122140

Plantinga, N. L., van Lanschot, M. C. J., Raven, C. F. H., Schuurman, R., Rirash, A. F., van Deursen, B., Boland, G. J., Siksma, T. O., Fries, E., Mostert, M., Thijsen, S. F. T., & Hofstra, L. M. (2023). Integrated surveillance of human respiratory viruses in addition to SARS-CoV-2 in a public testing facility in the Netherlands. Journal of Clinical Virology: The Official Publication of the Pan American Society for Clinical Virology, 158. https://doi.org/10.1016/j.jcv.2022.105346

Plass, D., Mangen, M.-J. J., Kraemer, A., Pinheiro, P., Gilsdorf, A., Krause, G., Gibbons, C. L., VAN Lier, A., McDONALD, S. A., Brooke, R. J., Kramarz, P., Cassini, A., & Kretzschmar, M. E. E. (2014). The disease burden of hepatitis B, influenza, measles and salmonellosis in Germany : First results of the burden of communicable diseases in Europe study. Epidemiology and Infection, 142(10). https://doi.org/10.1017/S0950268813003312

Plat, R., Vasile, M., Roubille, F., & Mercier, G. (2023). Relationships between the COVID-19 lockdown, socioeconomic factors and acute coronary syndrome hospitalisations in France. PloS One, 18(6). https://doi.org/10.1371/journal.pone.0286700

Platten, M., Cranen, R., Peters, C., Wisplinghoff, H., Nienhaus, A., Bach, A. D., & Michels, G. (2021). [Prevalence of SARS-CoV-2 in employees of a general hospital in Northrhine-Westphalia, Germany]. Deutsche Medizinische Wochenschrift (1946), 146(5). https://doi.org/10.1055/a-1322-5355

Platten, M., Nienhaus, A., Peters, C., Cranen, R., Wisplinghoff, H., Kersten, J. F., Bach, A. D., & Michels, G. (2022). Cumulative Incidence of SARS-CoV-2 in Healthcare Workers at a General Hospital in Germany during the Pandemic-A Longitudinal Analysis. International Journal of Environmental Research and Public Health, 19(4), 2429. https://doi.org/10.3390/ijerph19042429

Poblador-Plou, B., Carmona-Pírez, J., Ioakeim-Skoufa, I., Poncel-Falcó, A., Bliek-Bueno, K., Cano-Del Pozo, M., Gimeno-Feliú, L. A., González-Rubio, F., Aza-Pascual-Salcedo, M., Bandrés-Liso, A. C., Díez-Manglano, J., Marta-Moreno, J., Mucherino, S., Gimeno-Miguel, A., Prados-Torres, A., & EpiChron Group, null. (2020). Baseline Chronic Comorbidity and Mortality in Laboratory-Confirmed COVID-19 Cases : Results from the PRECOVID Study in Spain. International Journal of Environmental Research and Public Health, 17(14). https://doi.org/10.3390/ijerph17145171

Pockett, R. D., Campbell, D., Carroll, S., Rajoriya, F., & Adlard, N. (2013). Rotavirus, respiratory syncytial virus and non-rotaviral gastroenteritis analysis of hospital readmissions in England and Wales. Acta Paediatrica (Oslo, Norway: 1992), 102(4). https://doi.org/10.1111/apa.12124

Poethko-Müller, C., & Bödeker, B. (2017). The uptake of influenza vaccination for the 2013/2014 season in Germany. Journal of Health Monitoring, 2(4). https://doi.org/10.17886/RKI-GBE-2017-124

Poethko-Müller, C., Ordonez-Cruickshank, A., Nübel, J., Sarganas, G., Gößwald, A., Schmid, L., Rosario, A. S., Hoebel, J., Schlaud, M., & Scheidt-Nave, C. (2023). Prevalence of Long COVID-associated symptoms in adults with and without SARS-CoV-2 infection in Germany : Results of the population-based study “Corona Monitoring Nationwide 2021/22” (RKI-SOEP-2). medRxiv, (Poethko-Müller C., poethko-muellerc@rki.de; Ordonez-Cruickshank A.; Nübel J.; Sarganas G.; Gößwald A.; Schmid L.; Rosario A.S.; Hoebel J.; Schlaud M.; Scheidt-Nave C.) Robert Koch Institute (RKI), Germany. https://doi.org/10.1101/2023.09.12.23295426

Pokutnaya, D., Loiacono, M. M., Booth, H., Williams, R., Ma, C., Parker, J., Bricout, H., Farrow, S., & Nealon, J. (2022). The impact of clinical risk conditions on influenza and pneumonia diagnoses in England : A nationally representative retrospective cohort study, 2010-2019. Epidemiology and Infection, 150. https://doi.org/10.1017/S0950268822000838

Politi, J., Martín-Sánchez, M., Mercuriali, L., Borras-Bermejo, B., Lopez-Contreras, J., Vilella, A., Villar, J., COVID-19 Surveillance Working Group of Barcelona, Orcau, A., de Olalla, P. G., & Rius, C. (2021). Epidemiological characteristics and outcomes of COVID-19 cases : Mortality inequalities by socio-economic status, Barcelona, Spain, 24 February to 4 May 2020. Euro Surveillance: Bulletin Europeen Sur Les Maladies Transmissibles = European Communicable Disease Bulletin, 26(20). https://doi.org/10.2807/1560-7917.ES.2021.26.20.2001138

Polkowska-Kramek, A., Begier, E., Bruyndonckx, R., Liang, C., Beese, C., Brestrich, G., Tran, T. M. P., Nuttens, C., Casas, M., Bayer, L. J., Huebbe, B., Ewnetu, W. B., Agudelo, J. L. R., Gessner, B. D., von Eiff, C., & Rohde, G. (2024). Estimated Incidence of Hospitalizations and Deaths Attributable to Respiratory Syncytial Virus Infections Among Adults in Germany Between 2015 and 2019. Infectious Diseases and Therapy, 13(4). https://doi.org/10.1007/s40121-024-00951-0

Pollán, M., Pérez-Gómez, B., Pastor-Barriuso, R., Oteo, J., Hernán, M. A., Pérez-Olmeda, M., Sanmartín, J. L., Fernández-García, A., Cruz, I., Fernández de Larrea, N., Molina, M., Rodríguez-Cabrera, F., Martín, M., Merino-Amador, P., León Paniagua, J., Muñoz-Montalvo, J. F., Blanco, F., Yotti, R., & ENE-COVID Study Group. (2020). Prevalence of SARS-CoV-2 in Spain (ENE-COVID) : A nationwide, population-based seroepidemiological study. Lancet (London, England), 396(10250). https://doi.org/10.1016/S0140-6736(20)31483-5

Polyakov, P., Souty, C., Böelle, P.-Y., & Breban, R. (2019). Classification of Spatiotemporal Data for Epidemic Alert Systems : Monitoring Influenza-Like Illness in France. American Journal of Epidemiology, 188(4). https://doi.org/10.1093/aje/kwy254

Popa, M. V., Gurzu, I. L., Handra, C. M., Mandanach, C., Gurzu, B., Mîndru, D. E., Duceac Covrig, M., Ciuhodaru, M. I., & Duceac, L. D. (2025). Long COVID in Healthcare Workers from a Pediatric Hospital in Romania: A Cross-Sectional Study of Prevalence, Symptom Burden, and the Role of Vaccination and Reinfection. Journal of Clinical Medicine, 14(16), 5782. https://doi.org/10.3390/jcm14165782

Pople, D., Monk, E. J. M., Evans, S., Foulkes, S., Islam, J., Wellington, E., Atti, A., Hope, R., Robotham, J., Hopkins, S., Brown, C. S., Hall, V. J., & SIREN Study Group. (2022). Burden of SARS-CoV-2 infection in healthcare workers during second wave in England and impact of vaccines : Prospective multicentre cohort study (SIREN) and mathematical model. BMJ (Clinical Research Ed.), 378. https://doi.org/10.1136/bmj-2022-070379

Popoviciu, M. S., Paduraru, L., Stoica, R. A., Stoian, A. P., Teodorescu, C., & Cavalu, S. (2023). Prevalence of comorbidities and survival analysis of COVID-19 patients—An observational study from a tertiary healthcare center in North West Romania. JOURNAL OF MIND AND MEDICAL SCIENCES, 10(2), 330–338. https://doi.org/10.22543/2392-7674.1401

Porreca, A., & Di Nicola, M. (2023). Flu vaccination coverage in Italy in the COVID-19 era : A fuzzy functional k-means (FFKM) approach. Journal of Infection and Public Health, 16(11). https://doi.org/10.1016/j.jiph.2023.08.025

Porretta, A. D., Baggiani, A., Arzilli, G., Casigliani, V., Mariotti, T., Mariottini, F., Scardina, G., Sironi, D., Totaro, M., Barnini, S., & Privitera, G. P. (2020). Increased Risk of Acquisition of New Delhi Metallo-Beta-Lactamase-Producing Carbapenem-Resistant Enterobacterales (NDM-CRE) among a Cohort of COVID-19 Patients in a Teaching Hospital in Tuscany, Italy. Pathogens (Basel, Switzerland), 9(8). https://doi.org/10.3390/pathogens9080635

Porru, S., Spiteri, G., Monaco, M. G. L., Valotti, A., Carta, A., Lotti, V., Diani, E., Lippi, G., Gibellini, D., & Verlato, G. (2022). Post-Vaccination SARS-CoV-2 Infections among Health Workers at the University Hospital of Verona, Italy : A Retrospective Cohort Survey. Vaccines, 10(2). https://doi.org/10.3390/vaccines10020272

Portero de la Cruz, S., & Cebrino, J. (2020). Trends, Coverage and Influencing Determinants of Influenza Vaccination in the Elderly : A Population-Based National Survey in Spain (2006-2017). Vaccines, 8(2). https://doi.org/10.3390/vaccines8020327

Posteraro, B., De Angelis, G., Menchinelli, G., D'Inzeo, T., Fiori, B., De Maio, F., Cortazzo, V., Sanguinetti, M., & Spanu, T. (2021). Risk Factors for Mortality in Adult COVID-19 Patients Who Develop Bloodstream Infections Mostly Caused by Antimicrobial-Resistant Organisms : Analysis at a Large Teaching Hospital in Italy. Journal of Clinical Medicine, 10(8). https://doi.org/10.3390/jcm10081752

Postiglione, M., Miraglia Del Giudice, G., Della Polla, G., & Angelillo, I. F. (2023). Analysis of the COVID-19 vaccine willingness and hesitancy among parents of healthy children aged 6 months-4 years : A cross-sectional survey in Italy. Frontiers in Public Health, 11. https://doi.org/10.3389/fpubh.2023.1241514

Postma, M. J., Cheng, C.-Y., Buyukkaramikli, N. C., Hernandez Pastor, L., Vandersmissen, I., Van Effelterre, T., Openshaw, P., & Simoens, S. (2023). Predicted Public Health and Economic Impact of Respiratory Syncytial Virus Vaccination with Variable Duration of Protection for Adults ≥60 Years in Belgium. Vaccines, 11(5). https://doi.org/10.3390/vaccines11050990

Postma, M. J., Novak, A., Scheijbeler, H. W. K. F. H., Gyldmark, M., van Genugten, M. L. L., & Wilschut, J. C. (2007). Cost effectiveness of oseltamivir treatment for patients with influenza-like illness who are at increased risk for serious complications of influenza : Illustration for the Netherlands. PharmacoEconomics, 25(6). https://doi.org/10.2165/00019053-200725060-00005

Poukka, E., Baum, U., Palmu, A. A., Lehtonen, T. O., Salo, H., Nohynek, H., & Leino, T. (2022). Cohort study of Covid-19 vaccine effectiveness among healthcare workers in Finland, December 2020—October 2021. Vaccine, 40(5). https://doi.org/10.1016/j.vaccine.2021.12.032

Poukka, E., Perälä, J., Nohynek, H., Goebeler, S., Auranen, K., Leino, T., & Baum, U. (2024). Relative effectiveness of bivalent boosters against severe COVID-19 outcomes among people aged ≥ 65 years in Finland, September 2022 to August 2023. Euro Surveillance: Bulletin Europeen Sur Les Maladies Transmissibles = European Communicable Disease Bulletin, 29(37). https://doi.org/10.2807/1560-7917.ES.2024.29.37.2300587

Pouquet, M., Decarreaux, D., Di Domenico, L., Sabbatini, C. E., Prévot-Monsacre, P., Fourié, T., Villarroel, P. M. S., Priet, S., Blanché, H., Sebaoun, J.-M., Deleuze, J.-F., Turbelin, C., Rossignol, L., Werner, A., Kochert, F., Grosgogeat, B., Rabiega, P., Laupie, J., Abraham, N., … Falchi, A. (2024). SARS-CoV-2 infection prevalence and associated factors among primary healthcare workers in France after the third COVID-19 wave. Scientific Reports, 14(1). https://doi.org/10.1038/s41598-024-55477-9

Pouw, N., van de Maat, J., Veerman, K., Ten Oever, J., Janssen, N., Abbink, E., Reijers, M., de Mast, Q., Hoefsloot, W., van Crevel, R., Slieker, K., van Apeldoorn, M., Blaauw, M., Dofferhoff, A., & Hoogerwerf, J. (2021). Clinical characteristics and outcomes of 952 hospitalized COVID-19 patients in The Netherlands : A retrospective cohort study. PloS One, 16(3). https://doi.org/10.1371/journal.pone.0248713

Pouwels, K. B., House, T., Pritchard, E., Robotham, J. V., Birrell, P. J., Gelman, A., Vihta, K.-D., Bowers, N., Boreham, I., Thomas, H., Lewis, J., Bell, I., Bell, J. I., Newton, J. N., Farrar, J., Diamond, I., Benton, P., Walker, A. S., & COVID-19 Infection Survey Team. (2021). Community prevalence of SARS-CoV-2 in England from April to November, 2020 : Results from the ONS Coronavirus Infection Survey. The Lancet. Public Health, 6(1). https://doi.org/10.1016/S2468-2667(20)30282-6

Powell, A. A., Ireland, G., Leeson, R., Lacey, A., Ford, B., Poh, J., Ijaz, S., Shute, J., Cherepanov, P., Tedder, R., Bottomley, C., Dawe, F., Mangtani, P., Jones, P., Nguipdop-Djomo, P., Ladhani, S. N., & COVID-19 Schools Infection Survey Group. (2023). National and regional prevalence of SARS-CoV-2 antibodies in primary and secondary school children in England : The School Infection Survey, a national open cohort study, November 2021SARS-CoV-2 antibody prevalence in school children. The Journal of Infection, 86(4). https://doi.org/10.1016/j.jinf.2023.02.016

Prada, J. P., Maag, L. E., Siegmund, L., Bencurova, E., Chunguang, L., Koutsilieri, E., Dandekar, T., & Scheller, C. (2021). Estimation of R0 for the spread of SARS-CoV-2 in Germany from excess mortality. medRxiv, (Prada J.P.; Bencurova E.; Chunguang L.; Dandekar T., dandekar@biozentrum.uni-wuerzburg.de) Department of Bioinformatics, Biocenter, University of Würzburg, Am Hubland, Würzburg, Germany. https://doi.org/10.1101/2021.11.14.21266295

Prévost, B., Retbi, A., Binder-Foucard, F., Borde, A., Bruandet, A., Corvol, H., Gilleron, V., Le Bourhis-Zaimi, M., Lenne, X., Muller, J., Ouattara, E., Séguret, F., Tran Ba Loc, P., & Tezenas du Montcel, S. (2022). Risk factors for admission to the pediatric critical care unit among children hospitalized with COVID-19 in France. Frontiers in Pediatrics, 10. https://doi.org/10.3389/fped.2022.975826

Prieto-Alhambra, D., Balló, E., Coma, E., Mora, N., Aragón, M., Prats-Uribe, A., Fina, F., Benítez, M., Guiriguet, C., Fàbregas, M., Medina-Peralta, M., & Duarte-Salles, T. (2021). Filling the gaps in the characterization of the clinical management of COVID-19 : 30-day hospital admission and fatality rates in a cohort of 118 150 cases diagnosed in outpatient settings in Spain. International Journal of Epidemiology, 49(6). https://doi.org/10.1093/ije/dyaa190

Primieri, C., Chiavarini, M., Giacchetta, I., de Waure, C., & Bietta, C. (2023). COVID-19 Vaccination Actual Uptake and Potential Inequalities Due to Socio-Demographic Characteristics : A Population-Based Study in the Umbria Region, Italy. Vaccines, 11(8). https://doi.org/10.3390/vaccines11081351

Prinz, A. L., & Richter, D. J. (2022). Long-term exposure to fine particulate matter air pollution : An ecological study of its effect on COVID-19 cases and fatality in Germany. Environmental Research, 204(Pt A). https://doi.org/10.1016/j.envres.2021.111948

Proniewska-Sadowska, M., Poznańska, A., & Brydak, L. B. (2024). Influenza Hemagglutinin Antibody Levels in the Elderly : Impact of Sex, Age, and Influenza/COVID-19 Vaccination Status During the 2021/2022 Epidemic Season in Warsaw, Poland. Medical Science Monitor: International Medical Journal of Experimental and Clinical Research, 30. https://doi.org/10.12659/MSM.945002

Puggina, A., Dovizio, M., Domnich, A., Marijam, A., Veronesi, C., Rizzo, C., Vicentini, M., Degli Esposti, L., Calabrò, G. E., & Fonseca, M. J. (2025). Demographics and clinical burden of disease among RSV-hospitalized older adults in Italy: A retrospective cohort study. Human Vaccines & Immunotherapeutics, 21(1), 2479334. https://doi.org/10.1080/21645515.2025.2479334

Puggina, A., Rumi, F., Zarkadoulas, E., Marijam, A., & Calabró, G. E. (2025). The Potential Public Health Impact of the Adjuvanted Respiratory Syncytial Virus Prefusion F Protein Vaccine Among Older Adults in Italy. Vaccines, 13(3), 212. https://doi.org/10.3390/vaccines13030212

Puig-Barberà, J., Arnedo-Pena, A., Pardo-Serrano, F., Tirado-Balaguer, M. D., Pérez-Vilar, S., Silvestre-Silvestre, E., Calvo-Mas, C., Safont-Adsuara, L., Ruiz-García, M., & Surveillance and Vaccine Evaluation Group during the autumn 2009 H1N1 pandemic wave in Castellón, ... (2010). Effectiveness of seasonal 2008-2009, 2009-2010 and pandemic vaccines, to prevent influenza hospitalizations during the autumn 2009 influenza pandemic wave in Castellón, Spain. A test-negative, hospital-based, case-control study. Vaccine, 28(47), 7460–7467. https://doi.org/10.1016/j.vaccine.2010.09.042

Puig-Barberà, J., García-de-Lomas, J., Díez-Domingo, J., Arnedo-Pena, A., Ruiz-García, M., Limón-Ramírez, R., Pérez-Vilar, S., Micó-Esparza, J. L., Tortajada-Girbés, M., Carratalá-Munuera, C., Larrea-González, R., Beltrán-Garrido, J. M., Otero-Reigada, M. D. C., Mollar-Maseres, J., Correcher-Medina, P., Schwarz-Chavarri, G., Gil-Guillén, V., & Valencia Hospital Network for the Study of Influenza and Respiratory Virus Disease. (2014). Influenza vaccine effectiveness in preventing influenza A(H3N2)-related hospitalizations in adults targeted for vaccination by type of vaccine : A hospital-based test-negative study, 2011-2012 A(H3N2) predominant influenza season, Valencia, Spain. PloS One, 9(11). https://doi.org/10.1371/journal.pone.0112294

Pulido, J., Donat, M., Moreno, A., Politi, J., Cea-Soriano, L., Sordo, L., Mateo-Urdiales, A., Ronda, E., Belza, M. J., Barrio, G., & Regidor, E. (2024). Assessing educational disparities in COVID-19 related excess mortality in Spain : A population register-linked mortality study. Frontiers in Public Health, 12. https://doi.org/10.3389/fpubh.2024.1381298

Pullano, G., Valdano, E., Scarpa, N., Rubrichi, S., & Colizza, V. (2020). Evaluating the effect of demographic factors, socioeconomic factors, and risk aversion on mobility during the COVID-19 epidemic in France under lockdown : A population-based study. The Lancet. Digital Health, 2(12). https://doi.org/10.1016/S2589-7500(20)30243-0

Pumarola, T., Díez-Domingo, J., Martinón-Torres, F., Redondo Margüello, E., de Lejarazu Leonardo, R. O., Carmo, M., Bizouard, G., Drago, G., López-Belmonte, J. L., Bricout, H., de Courville, C., & Gil-de-Miguel, A. (2023). Excess hospitalizations and mortality associated with seasonal influenza in Spain, 2008-2018. BMC Infectious Diseases, 23(1). https://doi.org/10.1186/s12879-023-08015-3

Purrmann, L., Speichert, L.-J., Bäuerle, A., Teufel, M., Krakowczyk, J. B., Beckord, J., Felderhoff-Müser, U., Skoda, E.-M., & Dinse, H. (2023). COVID-19 Vaccine for Children : Determinants and Beliefs Contributing to Vaccination Decision of Parents in Germany 2021/2022. Vaccines, 12(1). https://doi.org/10.3390/vaccines12010020

Puzelli, S., Di Martino, A., Facchini, M., Fabiani, C., Calzoletti, L., Di Mario, G., Palmieri, A., Affanni, P., Camilloni, B., Chironna, M., D'Agaro, P., Giannecchini, S., Pariani, E., Serra, C., Rizzo, C., Bella, A., Donatelli, I., Castrucci, M. R., & Italian Influenza Laboratory Network. (2019). Co-circulation of the two influenza B lineages during 13 consecutive influenza surveillance seasons in Italy, 2004-2017. BMC Infectious Diseases, 19(1). https://doi.org/10.1186/s12879-019-4621-z

Puzelli, S., Facchini, M., de Marco, M. A., Palmieri, A., Spagnolo, D., Boros, S., Corcioli, F., Trotta, D., Bagnarelli, P., Azzi, A., Cassone, A., Rezza, G., Pompa, M. G., Oleari, F., & Donatelli, I. (2010). Molecular surveillance of pandemic influenza A(H1N1) viruses circulating in Italy from May 2009 to February 2010: Association between haemagglutinin mutations and clinical outcome. Eurosurveillance, 15(43), 1–3. https://doi.org/10.2807/ese.15.43.19696-en

Qamar, A. I., Gronwald, L., Timmesfeld, N., & Diebner, H. H. (2022). Local socio-structural predictors of COVID-19 incidence in Germany. Frontiers in Public Health, 10. https://doi.org/10.3389/fpubh.2022.970092

Quaranta, G., Formica, G., Machado, J. T., Lacarbonara, W., & Masri, S. F. (2020). Understanding COVID-19 nonlinear multi-scale dynamic spreading in Italy. Nonlinear Dynamics, 101(3). https://doi.org/10.1007/s11071-020-05902-1

Quentin, E., Ahmed, I., Duong, C.-H., Tubert-Bitter, P., & Escolano, S. (2025). Covid-19 Vaccination During Pregnancy in France: A Descriptive Study of Uptake Using the National Healthcare data System. Vaccine, 58, 127223. https://doi.org/10.1016/j.vaccine.2025.127223

Quercioli, C., Bosco, R., Bova, G., Mandò, M., De Marco, M., Dei, S., Gusinu, R., & Messina, G. (2023). Evaluating the effect of COVID-19 incidence on Emergency Departments admissions. Results from a retrospective study in Central Italy during the first year of pandemic. ANNALI DI IGIENE MEDICINA PREVENTIVA E DI COMUNITA, 35(5), 572–585. https://doi.org/10.7416/ai.2023.2570

Quéromès, G., Destras, G., Bal, A., Regue, H., Burfin, G., Brun, S., Fanget, R., Morfin, F., Valette, M., Trouillet-Assant, S., Lina, B., Frobert, E., & Josset, L. (2021). Characterization of SARS-CoV-2 ORF6 deletion variants detected in a nosocomial cluster during routine genomic surveillance, Lyon, France. Emerging Microbes & Infections, 10(1). https://doi.org/10.1080/22221751.2021.1872351

Rabe, A. P. J., Loke, W. J., Kalyani, R. N., Tummala, R., Stirnadel-Farrant, H. A., Were, J., & Winthrop, K. L. (2023). Impact of SARS-CoV-2 infection on patients with systemic lupus erythematosus in England prior to vaccination : A retrospective observational cohort study. BMJ Open, 13(11). https://doi.org/10.1136/bmjopen-2022-071072

Rabilloud, M., Riche, B., Etard, J. F., Elsensohn, M.-H., Voirin, N., Bénet, T., Iwaz, J., Ecochard, R., & Vanhems, P. (2022). COVID-19 outbreaks in nursing homes : A strong link with the coronavirus spread in the surrounding population, France, March to July 2020. PloS One, 17(1). https://doi.org/10.1371/journal.pone.0261756

Rachas, A., Fontaine, R., Thomas, M., Robine, J.-M., Gavazzi, G., Laurent, M., Carcaillon-Bentata, L., & Canouï-Poitrine, F. (2023). Individual and contextual risk factors for mortality in nursing home residents during

the first wave of COVID-19 in France : A multilevel analysis of a nationwide cohort study. Age and Ageing, 52(8). https://doi.org/10.1093/ageing/afad165

Radermacher, J., Thiel, S., Kannt, A., & Fröhlich, H. (2025). Wastewater as an early indicator for short-term forecasting COVID-19 hospitalization in Germany. BMC Public Health, 25(1), 2910. https://doi.org/10.1186/s12889-025-24149-2

Radev, S. T., Graw, F., Chen, S., Mutters, N. T., Eichel, V. M., Bärnighausen, T., & Köthe, U. (2021). OutbreakFlow : Model-based Bayesian inference of disease outbreak dynamics with invertible neural networks and its application to the COVID-19 pandemics in Germany. PLoS Computational Biology, 17(10). Embase. https://doi.org/10.1371/journal.pcbi.1009472

Radziejewska, J., Arkowski, J., Susło, R., Kędzierski, K., & Wawrzyńska, M. (2023). Analysis of COVID-19 Incidence and Protective Potential of Persisting IgG Class Antibodies against SARS-CoV-2 Infection in Hospital Staff in Poland. Vaccines, 11(7). https://doi.org/10.3390/vaccines11071198

Raffetti, E., Mondino, E., & Di Baldassarre, G. (2022). COVID-19 vaccine hesitancy in Sweden and Italy : The role of trust in authorities. Scandinavian Journal of Public Health, 50(6). https://doi.org/10.1177/14034948221099410

Rafia, R., Martyn-St James, M., Harnan, S., Metry, A., Hamilton, J., & Wailoo, A. (2022). A Cost-Effectiveness Analysis of Remdesivir for the Treatment of Hospitalized Patients With COVID-19 inno England and Wales. Value in Health: The Journal of the International Society for Pharmacoeconomics and Outcomes Research, 25(5). https://doi.org/10.1016/j.jval.2021.12.015

Rahi, M., Le Pluart, D., Beaudet, A., Ismaël, S., Parisey, M., Poey, N., Tarhini, H., Lescure, F.-X., Yazdanpanah, Y., & Deconinck, L. (2021). Sociodemographic characteristics and transmission risk factors in patients hospitalized for COVID-19 before and during the lockdown in France. BMC Infectious Diseases, 21(1). https://doi.org/10.1186/s12879-021-06419-7

Rahmon, I., Bosmans, M., Baliatsas, C., Hooiveld, M., Marra, E., & Dückers, M. (2024). COVID-19 Health Impact : A Use Case for Syndromic Surveillance System Monitoring Based on Primary Care Patient Registries in the Netherlands. JMIR Public Health and Surveillance, 10. https://doi.org/10.2196/53368

Rai, K. K., Gowman, H., Seif, M., Massey, L., Volkman, H., Schmetz, A., Nguyen, J., & Yang, J. (2025). COVID-19-Related Healthcare Resource Utilisation and Costs in Paediatric Patients in Germany: A Population-Based Study. Advances in Therapy. https://doi.org/10.1007/s12325-025-03313-5

Raja, A. I., Nicholls, G., Coldwell, M., van Veldhoven, K., Sandys, V., Atkinson, B., Nicholls, I., Spencer, A., Graham, A., Higgins, H., Atchison, C., Keen, C., Fletcher, T., Pearce, N., Brickley, E. B., & Chen, Y. (2024). A SARS-CoV-2 outbreak investigation at a storage and distribution centre in England : An assessment of worker- and workplace-related risk factors. Scientific Reports, 14(1). https://doi.org/10.1038/s41598-024-60194-4

Rakowski, F., Gruziel, M., Bieniasz-Krzywiec, L., & Radomski, J. (2010). Influenza epidemic spread simulation for Poland—A large scale, individual based model study. PHYSICA A-STATISTICAL MECHANICS AND ITS APPLICATIONS, 389(16). https://doi.org/10.1016/j.physa.2010.04.029

Ramón-Dangla, R., Rico-Gómez, M. L., & Issa-Khozouz, R. (2021). [Mortality by COVID-19 in Spain. Approximation through public health expenditure by autonomous communities.]. Revista Espanola De Salud Publica, 95. http://www.ncbi.nlm.nih.gov/pubmed/33664220

Ramos-Rincón, J.-M., Bernabeu-Wittel, M., Fiteni-Mera, I., López-Sampalo, A., López-Ríos, C., García-Andreu, M.-D.-M., Mancebo-Sevilla, J.-J., Jiménez-Juan, C., Matía-Sanz, M., López-Quirantes, P., Rubio-Rivas, M., Paredes-Ruiz, D., González-San-Narciso, C., González-Vega, R., Sanz-Espinosa, P., Hernández-Milián, A., Gonzalez-Noya, A., Gil-Sánchez, R., Boixeda, R., … SEMI-COVID-19 Network. (2022). Clinical Features and Risk Factors for Mortality Among Long-term Care Facility Residents Hospitalized Due to COVID-19 in Spain. The Journals of Gerontology. Series A, Biological Sciences and Medical Sciences, 77(4). https://doi.org/10.1093/gerona/glab305

Ramos-Rincon, J.-M., Buonaiuto, V., Ricci, M., Martín-Carmona, J., Paredes-Ruíz, D., Calderón-Moreno, M., Rubio-Rivas, M., Beato-Pérez, J.-L., Arnalich-Fernández, F., Monge-Monge, D., Vargas-Núñez, J.-A., Acebes-

Repiso, G., Mendez-Bailon, M., Perales-Fraile, I., García-García, G.-M., Guisado-Vasco, P., Abdelhady-Kishta, A., Pascual-Pérez, M.-L.-R., Rodríguez-Fernández-Viagas, C., … SEMI-COVID-19 Network. (2021). Clinical Characteristics and Risk Factors for Mortality in Very Old Patients Hospitalized With COVID-19 in Spain. The Journals of Gerontology. Series A, Biological Sciences and Medical Sciences, 76(3). https://doi.org/10.1093/gerona/glaa243

Ramos-Rincon, J.-M., Cobos-Palacios, L., López-Sampalo, A., Ricci, M., Rubio-Rivas, M., Martos-Pérez, F., Lalueza-Blanco, A., Moragón-Ledesma, S., Fonseca-Aizpuru, E.-M., García-García, G.-M., Beato-Perez, J.-L., Josa-Laorden, C., Arnalich-Fernández, F., Molinos-Castro, S., Torres-Peña, J.-D., Artero, A., Vargas-Núñez, J.-A., Mendez-Bailon, M., Loureiro-Amigo, J., … On Behalf Of The Semi-Covid-Network, null. (2022). Ethnicity and Clinical Outcomes in Patients Hospitalized for COVID-19 in Spain : Results from the Multicenter SEMI-COVID-19 Registry. Journal of Clinical Medicine, 11(7). https://doi.org/10.3390/jcm11071949

Ramos-Rincon, J.-M., Cobos-Palacios, L., López-Sampalo, A., Ricci, M., Rubio-Rivas, M., Nuñez-Rodriguez, M.-V., Miranda-Godoy, R., García-Leoni, M.-E., Fernández-Madera-Martínez, R., García-García, G.-M., Beato-Perez, J.-L., Monge-Monge, D., Asín-Samper, U., Bustamante-Vega, M., Rábago-Lorite, I., Freire-Castro, S.-J., Miramontes-González, J.-P., Magallanes-Gamboa, J.-O., Alcalá-Pedrajas, J.-N., … SEMI-COVID-19 Network. (2022). Differences in clinical features and mortality in very old unvaccinated patients (≥ 80 years) hospitalized with COVID-19 during the first and successive waves from the multicenter SEMI-COVID-19 Registry (Spain). BMC Geriatrics, 22(1). https://doi.org/10.1186/s12877-022-03191-4

Ramos-Rincón, J.-M., Pinargote-Celorio, H., González-de-la-Aleja, P., Sánchez-Payá, J., Reus, S., Rodríguez-Díaz, J.-C., & Merino, E. (2024). Impact of influenza related hospitalization in Spain : Characteristics and risk factor of mortality during five influenza seasons (2016 to 2021). Frontiers in Public Health, 12. https://doi.org/10.3389/fpubh.2024.1360372

Ramos-Rincon, J.-M., Pinargote-Celorio, H., Sanchez-Martinez, R., Rodríguez-Diaz, J.-C., Otero, S., Valero-Sempere, F., Torrús, D., Valero-Novella, B., Riera, G., Boix, V., & Merino, E. (2025). In-Hospital Death and Risk Factors in Adults With Influenza in Spain (2016-2022): A Cross-Sectional Study With Focus on Older Patients. Health Science Reports, 8(3), e70458. https://doi.org/10.1002/hsr2.70458

Ramos-Rincón, J.-M., Sánchez-Paya, J., González-De-La-Aleja, P., Rodríguez-Díaz, J.-C., & Merino, E. (2025). A national population-based study of mortality and risk factors in COVID-19-hospitalized patients in Spain (2020-2021). Frontiers in Public Health, 13, 1488283. https://doi.org/10.3389/fpubh.2025.1488283

Rance, F., Chave, C., De Blic, J., Deschildre, A., Donato, L., Dubus, J., Fayon, M., Labbe, A., Le Bourgeois, M., Llerena, C., Le Manach, G., Pin, I., Santos, C., Thumerelle, C., Aubert, M., & Weil-Olivier, C. (2008). Low influenza vaccination coverage in asthmatic children in France in 2006-7. Euro Surveillance: Bulletin Europeen Sur Les Maladies Transmissibles = European Communicable Disease Bulletin, 13(43). https://doi.org/10.2807/ese.13.43.19016-en

Ranzenigo, M., Ceccarelli, D., Galli, L., Bertoni, C., Siribelli, A., Messina, E., Hasson, H., Lolatto, R., Castagna, A., Uberti-Foppa, C., & Morsica, G. (2023). Risk of mortality in people with chronic liver diseases hospitalized for Coronavirus disease of 2019 (COVID-19) in a tertiary hospital in Lombardy, Italy. The New Microbiologica, 46(3). http://www.ncbi.nlm.nih.gov/pubmed/37747475

Ranzi, A., Stafoggia, M., Giannini, S., Ancona, C., Bella, A., Cattani, G., Pezzotti, P., Iavarone, I., & EpiCovAir Study Group. (2023). [Long-term exposure to ambient air pollution and the incidence of SARS-CoV-2 infections in Italy : The EpiCovAir study]. Epidemiologia E Prevenzione, 47(3). https://doi.org/10.19191/EP23.3.A605.025

Raponi, S., Durazzi, F., Derus, N. R., Giampieri, E., Miglio, R., Castellani, G., & Sala, C. (2023). Risk Factors for Admission into COVID-19 General Wards, Sub-Intensive and Intensive Care Units among SARS-CoV-2 Positive Subjects in the Municipality of Bologna, Italy. medRxiv, (Raponi S.; Durazzi F.) Department of Physics and Astronomy “Augusto Righi”, University of Bologna, Bologna, Italy. https://doi.org/10.1101/2023.07.12.23292559

Rasmussen, S., Petersen, M., & Hoiby, N. (2021). SARS-CoV-2 infection dynamics in Denmark, February through October 2020 : Nature of the past epidemic and how it may develop in the future. PLOS ONE, 16(4). https://doi.org/10.1371/journal.pone.0249733

Rattay, P., Michalski, N., Domanska, O. M., Kaltwasser, A., De Bock, F., Wieler, L. H., & Jordan, S. (2021). Differences in risk perception, knowledge and protective behaviour regarding COVID-19 by education level among women and men in Germany. Results from the COVID-19 Snapshot Monitoring (COSMO) study. PloS One, 16(5). https://doi.org/10.1371/journal.pone.0251694

Ravanan, R., Callaghan, C. J., Mumford, L., Ushiro-Lumb, I., Thorburn, D., Casey, J., Friend, P., Parameshwar, J., Currie, I., Burnapp, L., Baker, R., Dudley, J., Oniscu, G. C., Berman, M., Asher, J., Harvey, D., Manara, A., Manas, D., Gardiner, D., & Forsythe, J. L. R. (2020). SARS-CoV-2 infection and early mortality of waitlisted and solid organ transplant recipients in England : A national cohort study. American Journal of Transplantation: Official Journal of the American Society of Transplantation and the American Society of Transplant Surgeons, 20(11). https://doi.org/10.1111/ajt.16247

Rave, M., & Kauermann, G. (2024). The Skellam distribution revisited : Estimating the unobserved incoming and outgoing ICU COVID-19 patients on a regional level in Germany. STATISTICAL MODELLING. https://doi.org/10.1177/1471082X241235024

Ravelli, E., & Gonzales Martinez, R. (2022). Environmental risk factors of airborne viral transmission : Humidity, Influenza and SARS-CoV-2 in the Netherlands. Spatial and Spatio-Temporal Epidemiology, 41. https://doi.org/10.1016/j.sste.2021.100432

Rawson, T., Hinsley, W., Sonabend, R., Semenova, E., Cori, A., & Ferguson, N. M. (2024). The impact of health inequity on regional variation of COVID-19 transmission in England. medRxiv, (Rawson T., t.rawson@imperial.ac.uk; Hinsley W.; Sonabend R.; Cori A.; Ferguson N.M.) MRC, Centre for Global Infectious Disease Analysis, Jameel Institute, School of Public Health, Imperial College London, London, United Kingdom. https://doi.org/10.1101/2024.04.20.24306121

Razzini, K., Castrica, M., Menchetti, L., Maggi, L., Negroni, L., Orfeo, N. V., Pizzoccheri, A., Stocco, M., Muttini, S., & Balzaretti, C. M. (2020). SARS-CoV-2 RNA detection in the air and on surfaces in the COVID-19 ward of a hospital in Milan, Italy. The Science of the Total Environment, 742. https://doi.org/10.1016/j.scitotenv.2020.140540

Recalde, M., Roel, E., Pistillo, A., Sena, A. G., Prats-Uribe, A., Ahmed, W.-U.-R., Alghoul, H., Alshammari, T. M., Alser, O., Areia, C., Burn, E., Casajust, P., Dawoud, D., DuVall, S. L., Falconer, T., Fernández-Bertolín, S., Golozar, A., Gong, M., Lai, L. Y. H., … Duarte-Salles, T. (2021). Characteristics and outcomes of 627 044 COVID-19 patients living with and without obesity in the United States, Spain, and the United Kingdom. International Journal of Obesity (2005), 45(11). https://doi.org/10.1038/s41366-021-00893-4

Rector, A., Bloemen, M., Thijssen, M., Delang, L., Raymenants, J., Thibaut, J., Pussig, B., Fondu, L., Aertgeerts, B., Van Ranst, M., Van Geet, C., Arnout, J., & Wollants, E. (2023). Monitoring of SARS-CoV-2 concentration and circulation of variants of concern in wastewater of Leuven, Belgium. Journal of Medical Virology, 95(2). https://doi.org/10.1002/jmv.28587

Redondo González, O., Olteanu Olteanu, F. C., Arechederra Calderón, J. J., Bravo Villaseñor, C. M., Miras Aguilar, I., & Rodríguez Arbaizar, J. (2020). Flu and VRS 2018-19 Season and vaccine effectiveness against the need for hospital flu treatment in Guadalajara province, Spain. Medicina Clinica, 155(3). https://doi.org/10.1016/j.medcli.2020.01.021

Redondo, E., Drago, G., López-Belmonte, J. L., Guillén, J. M., Bricout, H., Alvarez, F. P., Callejo, D., & Gil de Miguel, Á. (2021). Cost-utility analysis of influenza vaccination in a population aged 65 years or older in Spain with a high-dose vaccine versus an adjuvanted vaccine. Vaccine, 39(36). https://doi.org/10.1016/j.vaccine.2021.07.048

Reeves, R. M., Hardelid, P., Gilbert, R., Warburton, F., Ellis, J., & Pebody, R. G. (2017). Estimating the burden of respiratory syncytial virus (RSV) on respiratory hospital admissions in children less than five years of age in England, 2007-2012. Influenza and Other Respiratory Viruses, 11(2). https://doi.org/10.1111/irv.12443

Reeves, R. M., Hardelid, P., Panagiotopoulos, N., Minaji, M., Warburton, F., & Pebody, R. (2019). Burden of hospital admissions caused by respiratory syncytial virus (RSV) in infants in England : A data linkage modelling study. The Journal of Infection, 78(6). https://doi.org/10.1016/j.jinf.2019.02.012

Reggio Emilia Covid-19 Working Group. (2020). Prevalence of SARS-CoV-2 (Covid-19) in Italians and in immigrants in an area of Northern Italy (Reggio Emilia). Epidemiologia E Prevenzione, 44(4). https://doi.org/10.19191/EP20.4.P304.061

Rehn, M., Carnahan, A., Merk, H., Kühlmann-Berenzon, S., Galanis, I., Linde, A., & Nyrén, O. (2014). Evaluation of an Internet-based monitoring system for influenza-like illness in Sweden. PloS One, 9(5). https://doi.org/10.1371/journal.pone.0096740

Reich, T., & Budka, M. (2019). Proof of concept for a syndromic surveillance system based on routine ambulance records in the South West of England, for the influenza season 2016/2017. British Paramedic Journal, 4(2). https://doi.org/10.29045/14784726.2019.09.4.2.22

Reilev, M., Kristensen, K. B., Pottegård, A., Lund, L. C., Hallas, J., Ernst, M. T., Christiansen, C. F., Sørensen, H. T., Johansen, N. B., Brun, N. C., Voldstedlund, M., Støvring, H., Thomsen, M. K., Christensen, S., Gubbels, S., Krause, T. G., Mølbak, K., & Thomsen, R. W. (2020). Characteristics and predictors of hospitalization and death in the first 11 122 cases with a positive RT-PCR test for SARS-CoV-2 in Denmark : A nationwide cohort. International Journal of Epidemiology, 49(5). https://doi.org/10.1093/ije/dyaa140

Reilev, M., Olesen, M., Kildegaard, H., Støvring, H., Andersen, J. H., Hallas, J., Lund, L. C., Ladebo, L., Ernst, M. T., Damkier, P., Jensen, P. B., Pottegård, A., & Rasmussen, L. (2022). Changing characteristics over time of individuals receiving COVID-19 vaccines in Denmark : A population-based descriptive study of vaccine uptake. Scandinavian Journal of Public Health, 50(6). https://doi.org/10.1177/14034948221108246

Reinwald, M., Deckert, P. M., Ritter, O., Andresen, H., Schreyer, A. G., Weylandt, K. H., Dammermann, W., & Lüth, S. (2021). Prevalence and Course of IgA and IgG Antibodies against SARS-CoV-2 in Healthcare Workers during the First Wave of the COVID-19 Outbreak in Germany : Interim Results from an Ongoing Observational Cohort Study. Healthcare (Basel, Switzerland), 9(5). https://doi.org/10.3390/healthcare9050498

Reis, M., Michalski, N., Bartig, S., Wulkotte, E., Poethko-Müller, C., Graeber, D., Rosario, A. S., Hövener, C., & Hoebel, J. (2024). Reconsidering inequalities in COVID-19 vaccine uptake in Germany : A spatiotemporal analysis combining individual educational level and area-level socioeconomic deprivation. Scientific Reports, 14(1). https://doi.org/10.1038/s41598-024-75273-9

Remaeus, K., Savchenko, J., Brismar Wendel, S., Brusell Gidlöf, S., Graner, S., Jones, E., Molin, J., Saltvedt, S., Wallström, T., & Pettersson, K. (2020). Characteristics and short-term obstetric outcomes in a case series of 67 women test-positive for SARS-CoV-2 in Stockholm, Sweden. Acta Obstetricia Et Gynecologica Scandinavica, 99(12). https://doi.org/10.1111/aogs.14006

Remón-Berrade, M., Guillen-Aguinaga, S., Sarrate-Adot, I., Garcia-Garcia, M. P., Lerga-Berruezo, M. D. C., Guillen-Aguinaga, L., & Guillen-Grima, F. (2021). Risk of Secondary Household Transmission of COVID-19 from Health Care Workers in a Hospital in Spain. Epidemiologia (Basel, Switzerland), 3(1). https://doi.org/10.3390/epidemiologia3010001

Remppis, J., Ganzenmueller, T., Kohns Vasconcelos, M., Heinzel, O., Handgretinger, R., & Renk, H. (2021). A case series of children and young people admitted to a tertiary care hospital in Germany with COVID-19. BMC Infectious Diseases, 21(1). https://doi.org/10.1186/s12879-021-05791-8

Remschmidt, C., Rieck, T., Bödeker, B., & Wichmann, O. (2015). Application of the screening method to monitor influenza vaccine effectiveness among the elderly in Germany. BMC Infectious Diseases, 15. https://doi.org/10.1186/s12879-015-0882-3

Renard, F., Scohy, A., Van der Heyden, J., Peeters, I., Dequeker, S., Vandael, E., Van Goethem, N., Dubourg, D., De Viron, L., Kongs, A., Hammami, N., Devleesschauwer, B., Sasse, A., Rebolledo Gonzalez, J., & Bustos Sierra, N. (2021). Establishing an ad hoc COVID-19 mortality surveillance during the first epidemic wave in Belgium, 1 March to 21 June 2020. Euro Surveillance: Bulletin Europeen Sur Les Maladies Transmissibles = European Communicable Disease Bulletin, 26(48). https://doi.org/10.2807/1560-7917.ES.2021.26.48.2001402

Renault, V., Fontaine, S., Mpouam, S. E., & Saegerman, C. (2022). Main determinants of the acceptance of COVID-19 control measures by the population : A first pilot survey at the University of Liege, Belgium. Transboundary and Emerging Diseases, 69(4). https://doi.org/10.1111/tbed.14410

Reno, C., Lenzi, J., Navarra, A., Barelli, E., Gori, D., Lanza, A., Valentini, R., Tang, B., & Fantini, M. P. (2020). Forecasting COVID-19-Associated Hospitalizations under Different Levels of Social Distancing in Lombardy and Emilia-Romagna, Northern Italy : Results from an Extended SEIR Compartmental Model. Journal of Clinical Medicine, 9(5). https://doi.org/10.3390/jcm9051492

Reno, C., Maietti, E., Fantini, M. P., Savoia, E., Manzoli, L., Montalti, M., & Gori, D. (2021). Enhancing COVID-19 Vaccines Acceptance : Results from a Survey on Vaccine Hesitancy in Northern Italy. Vaccines, 9(4). https://doi.org/10.3390/vaccines9040378

Requena-Méndez, A., Mougkou, A., Hedberg, P., van der Werff, S. D., Tanushi, H., Hertting, O., Färnert, A., Nyberg, F., Naucler, P., & COVID-19-data-review collaborators. (2022). SARS-CoV-2 testing in patients with low COVID-19 suspicion at admission to a tertiary care hospital, Stockholm, Sweden, March to September 2020. Euro Surveillance: Bulletin Europeen Sur Les Maladies Transmissibles = European Communicable Disease Bulletin, 27(7). https://doi.org/10.2807/1560-7917.ES.2022.27.7.2100079

Resta, E., Cuscianna, E., Pierucci, P., Custodero, C., Solfrizzi, V., Sabbà, C., Palmisano, C. M., Barratta, F., De Candia, M. L., Tummolo, M. G., Capozza, E., Lomuscio, S., De Michele, L., Tafuri, S., Resta, O., & Lenato, G. M. (2023). Significant burden of post-COVID exertional dyspnoea in a South-Italy region : Knowledge of risk factors might prevent further critical overload on the healthcare system. Frontiers in Public Health, 11. https://doi.org/10.3389/fpubh.2023.1273853

Rhedin, S., Hamrin, J., Naucler, P., Bennet, R., Rotzén-Östlund, M., Färnert, A., & Eriksson, M. (2012). Respiratory viruses in hospitalized children with influenza-like illness during the h1n1 2009 pandemic in Sweden [corrected]. PloS One, 7(12), e51491. https://doi.org/10.1371/journal.pone.0051491

Riad, F., Alruwaili, B., Gemeay, A., & Hussam, E. (2022). Statistical modeling for COVID-19 virus spread in Kingdom of Saudi Arabia and Netherlands. ALEXANDRIA ENGINEERING JOURNAL, 61(12). https://doi.org/10.1016/j.aej.2022.03.015

Ribera, J.-M., Morgades, M., Coll, R., Barba, P., López-Lorenzo, J.-L., Montesinos, P., Foncillas, M.-A., Cabrero, M., Gómez-Centurión, I., Morales, M.-D., Varela, M.-R., Herrera, P., García-Cadenas, I., Calbacho, M., Torrent, A., Maluquer, C., Calabuig, M., Garcia-Guiñon, A., Bautista, G., … Piñana, J.-L. (2021). Frequency, Clinical Characteristics and Outcome of Adults With Acute Lymphoblastic Leukemia and COVID 19 Infection in the First vs. Second Pandemic Wave in Spain. Clinical Lymphoma, Myeloma & Leukemia, 21(10), e801–e809. https://doi.org/10.1016/j.clml.2021.06.024

Ribes, M., Montañà, J., Vidal, M., Aguilar, R., Nicolás, P., Alfonso, U., Rodrigo, N., Carolis, C., Dobaño, C., Moncunill, G., & Chaccour, C. (2023). Seroprevalence and socioeconomic impact of the first SARS-CoV-2 infection wave in a small town in Navarre, Spain. Scientific Reports, 13(1). https://doi.org/10.1038/s41598-023-30542-x

Riccardo, F., Ajelli, M., Andrianou, X. D., Bella, A., Del Manso, M., Fabiani, M., Bellino, S., Boros, S., Urdiales, A. M., Marziano, V., Rota, M. C., Filia, A., D'Ancona, F., Siddu, A., Punzo, O., Trentini, F., Guzzetta, G., Poletti, P., Stefanelli, P., … COVID-19 working group. (2020). Epidemiological characteristics of COVID-19 cases and estimates of the reproductive numbers 1 month into the epidemic, Italy, 28 January to 31 March 2020. Euro Surveillance: Bulletin Europeen Sur Les Maladies Transmissibles = European Communicable Disease Bulletin, 25(49). https://doi.org/10.2807/1560-7917.ES.2020.25.49.2000790

Riccò, M., Baldassarre, A., Provenzano, S., Corrado, S., Cerviere, M. P., Parisi, S., Marchesi, F., & Bottazzoli, M. (2022). Infodemiology of RSV in Italy (2017-2022) : An Alternative Option for the Surveillance of Incident Cases in Pediatric Age? Children (Basel, Switzerland), 9(12). https://doi.org/10.3390/children9121984

Rieg, S., von Cube, M., Kalbhenn, J., Utzolino, S., Pernice, K., Bechet, L., Baur, J., Lang, C. N., Wagner, D., Wolkewitz, M., Kern, W. V., Biever, P., & COVID UKF Study Group. (2020). COVID-19 in-hospital mortality and mode of death in a dynamic and non-restricted tertiary care model in Germany. PloS One, 15(11). https://doi.org/10.1371/journal.pone.0242127

Rifino, N., Censori, B., Agazzi, E., Alimonti, D., Bonito, V., Camera, G., Conti, M. Z., Foresti, C., Frigeni, B., Gerevini, S., Grimoldi, M., La Gioia, S., Partziguian, T., Quadri, S., Riva, R., Servalli, M. C., Sgarzi, M., Storti, B., Vedovello, M., … Sessa, M. (2021). Neurologic manifestations in 1760 COVID-19 patients admitted to Papa

Giovanni XXIII Hospital, Bergamo, Italy. Journal of Neurology, 268(7). https://doi.org/10.1007/s00415-020-10251-5

Rigoine de Fougerolles, T., Puig-Barbera, J., Kassianos, G., Vanhems, P., Schelling, J., Crepey, P., de Lejarazu, R. O., Ansaldi, F., Fruhwein, M., Galli, C., Mosnier, A., Pariani, E., Rasuli, A., Vitoux, O., Watkins, J., Weinke, T., & Bricout, H. (2022). A comparison of coronavirus disease 2019 and seasonal influenza surveillance in five European countries : France, Germany, Italy, Spain and the United Kingdom. Influenza and Other Respiratory Viruses, 16(3). https://doi.org/10.1111/irv.12941

Riphagen-Dalhuisen, J., Kuiphuis, J. C. F., Procé, A. R., Luytjes, W., Postma, M. J., & Hak, E. (2012). Contributing factors to influenza vaccine uptake in general hospitals : An explorative management questionnaire study from the Netherlands. BMC Public Health, 12. https://doi.org/10.1186/1471-2458-12-1101

Rittweger, J., Gilardi, L., Baltruweit, M., Dally, S., Erbertseder, T., Mittag, U., Naeem, M., Schmid, M., Schmitz, M.-T., Wüst, S., Dech, S., Jordan, J., Antoni, T., & Bittner, M. (2022). Temperature and particulate matter as environmental factors associated with seasonality of influenza incidence—An approach using Earth observation-based modeling in a health insurance cohort study from Baden-Württemberg (Germany). Environmental Health: A Global Access Science Source, 21(1). https://doi.org/10.1186/s12940-022-00927-y

Rizzo, C., & Bella, A. (2016). The impact of influenza virus B in Italy : Myth or reality? Journal of Preventive Medicine and Hygiene, 57(1). Embase. https://www.embase.com/search/results?subaction=viewrecord&id=L611306276&from=export

Rizzo, C., Bella, A., Alfonsi, V., Puzelli, S., Palmieri, A. P., Chironna, M., Pariani, E., Piatti, A., Tiberti, D., Ghisetti, V., Rangoni, R., Colucci, M. E., Affanni, P., Germinario, C., & Castrucci, M. R. (2016). Influenza vaccine effectiveness in Italy : Age, subtype-specific and vaccine type estimates 2014/15 season. Vaccine, 34(27). https://doi.org/10.1016/j.vaccine.2016.04.072

Rizzo, C., Declich, S., Bella, A., Caporali, M. G., Lana, S., Pompa, M. G., Vellucci, L., & Salmaso, S. (2009). Enhanced epidemiological surveillance of influenza A(H1N1)v in Italy. Euro Surveillance: Bulletin Europeen Sur Les Maladies Transmissibles = European Communicable Disease Bulletin, 14(27). https://doi.org/10.2807/ese.14.27.19266-en

Rizzo, C., Gesualdo, F., Loconsole, D., Pandolfi, E., Bella, A., Orsi, A., Guarona, G., Panatto, D., Icardi, G., Napoli, C., Orsi, G. B., Manini, I., Montomoli, E., Campagna, I., Russo, L., Alfonsi, V., Puzelli, S., Reale, A., Raucci, U., … Tozzi, A. E. (2020). Moderate Vaccine Effectiveness against Severe Acute Respiratory Infection Caused by A(H1N1)pdm09 Influenza Virus and No Effectiveness against A(H3N2) Influenza Virus in the 2018/2019 Season in Italy. Vaccines, 8(3). https://doi.org/10.3390/vaccines8030427

Rizzo, M., Foresti, L., & Montano, N. (2020). Comparison of Reported Deaths From COVID-19 and Increase in Total Mortality in Italy. JAMA Internal Medicine, 180(9). https://doi.org/10.1001/jamainternmed.2020.2543

Robert, J., Detournay, B., Levant, M. C., Uhart, M., Gourmelen, J., & Cohen, J. M. (2020). Flu vaccine coverage for recommended populations in France. Medecine Et Maladies Infectieuses, 50(8). https://doi.org/10.1016/j.medmal.2019.12.004

Roberts, L. N., Navaratnam, A. V., Arya, R., Briggs, T. W. R., & Gray, W. K. (2022). Venous thromboembolism in patients hospitalised with COVID-19 in England. Thrombosis Research, 213. https://doi.org/10.1016/j.thromres.2022.03.017

Robotto, A., Lembo, D., Quaglino, P., Brizio, E., Polato, D., Civra, A., Cusato, J., & Di Perri, G. (2022). Wastewater-based SARS-CoV-2 environmental monitoring for Piedmont, Italy. Environmental Research, 203. https://doi.org/10.1016/j.envres.2021.111901

Roccetti, M. (2023). Excess mortality and COVID-19 deaths in Italy : A peak comparison study. Mathematical Biosciences and Engineering: MBE, 20(4). https://doi.org/10.3934/mbe.2023304

Roche, B., Garchitorena, A., & Roiz, D. (2020). The impact of lockdown strategies targeting age groups on the burden of COVID-19 in France. Epidemics, 33. https://doi.org/10.1016/j.epidem.2020.100424

Rockenfeller, R., Günther, M., & Mörl, F. (2023). Reports of deaths are an exaggeration : All-cause and NAA-test-conditional mortality in Germany during the SARS-CoV-2 era. Royal Society Open Science, 10(8). https://doi.org/10.1098/rsos.221551

Rodilla, E., López-Carmona, M. D., Cortes, X., Cobos-Palacios, L., Canales, S., Sáez, M. C., Campos Escudero, S., Rubio-Rivas, M., Díez Manglano, J., Freire Castro, S. J., Vázquez Piqueras, N., Mateo Sanchis, E., Pesqueira Fontan, P. M., Magallanes Gamboa, J. O., González García, A., Madrid Romero, V., Tamargo Chamorro, L., González Moraleja, J., Villanueva Martínez, J., … SEMI-COVID-19 Network. (2021). Impact of Arterial Stiffness on All-Cause Mortality in Patients Hospitalized With COVID-19 in Spain. Hypertension (Dallas, Tex.: 1979), 77(3). https://doi.org/10.1161/HYPERTENSIONAHA.120.16563

Rodríguez de Limia Ramírez, K., Ruiz-Robledillo, N., Duro-Torrijos, J. L., García-Román, V., Albaladejo-Blázquez, N., & Ferrer-Cascales, R. (2021). Prevalence of SARS-CoV-2 Infection in a Sample of Health Workers in Two Health Departments of the Valencian Community in Spain. International Journal of Environmental Research and Public Health, 19(1). https://doi.org/10.3390/ijerph19010066

Rodríguez, A., Ruiz-Botella, M., Martín-Loeches, I., Jimenez Herrera, M., Solé-Violan, J., Gómez, J., Bodí, M., Trefler, S., Papiol, E., Díaz, E., Suberviola, B., Vallverdu, M., Mayor-Vázquez, E., Albaya Moreno, A., Canabal Berlanga, A., Sánchez, M., Del Valle Ortíz, M., Ballesteros, J. C., Martín Iglesias, L., … COVID-19 SEMICYUC Working Group. (2021). Deploying unsupervised clustering analysis to derive clinical phenotypes and risk factors associated with mortality risk in 2022 critically ill patients with COVID-19 in Spain. Critical Care (London, England), 25(1). https://doi.org/10.1186/s13054-021-03487-8

Rodríguez, J. P., Aleta, A., & Moreno, Y. (2023). Digital cities and the spread of COVID-19 : Characterizing the impact of non-pharmaceutical interventions in five cities in Spain. Frontiers in Public Health, 11. https://doi.org/10.3389/fpubh.2023.1122230

Rodríguez-Barranco, M., Rivas-García, L., Quiles, J. L., Redondo-Sánchez, D., Aranda-Ramírez, P., Llopis-González, J., Sánchez Pérez, M. J., & Sánchez-González, C. (2021). The spread of SARS-CoV-2 in Spain : Hygiene habits, sociodemographic profile, mobility patterns and comorbidities. Environmental Research, 192. https://doi.org/10.1016/j.envres.2020.110223

Rodríguez-Blanco, N., Tuells, J., Vila-Candel, R., & Nolasco, A. (2019). Adherence and Concordance of Influenza and Pertussis Vaccination Coverage in Pregnant Women in Spain. International Journal of Environmental Research and Public Health, 16(4). https://doi.org/10.3390/ijerph16040543

Rodriguez-Gonzalez, C. G., Chamorro-de-Vega, E., Valerio, M., Amor-Garcia, M. A., Tejerina, F., Sancho-Gonzalez, M., Narrillos-Moraza, A., Gimenez-Manzorro, A., Manrique-Rodriguez, S., Machado, M., Olmedo, M., Escudero-Vilaplana, V., Villanueva-Bueno, C., Torroba-Sanz, B., Melgarejo-Ortuño, A., Vicente-Valor, J., Herranz, A., Bouza, E., Muñoz, P., & Sanjurjo, M. (2021). COVID-19 in hospitalised patients in Spain : A cohort study in Madrid. International Journal of Antimicrobial Agents, 57(2). https://doi.org/10.1016/j.ijantimicag.2020.106249

Rodríguez-Rieiro, C., Carrasco-Garrido, P., Hernández-Barrera, V., de Andres, A., Jimenez-Trujillo, I., de Miguel, A., & Jiménez-García, R. (2012). Pandemic influenza hospitalization in Spain (2009) Incidence, in-hospital mortality, comorbidities and costs. HUMAN VACCINES & IMMUNOTHERAPEUTICS, 8(4), 443–447. https://doi.org/10.4161/hv.18911

Rodríguez-Rieiro, C., Domínguez-Berjón, M. F., Esteban-Vasallo, M. D., Sánchez-Perruca, L., Astray-Mochales, J., Fornies, D. I., Ordoñez, D. B., & Jiménez-García, R. (2010). Vaccination coverage against 2009 seasonal influenza in chronically ill children and adults : Analysis of population registries in primary care in Madrid (Spain). Vaccine, 28(38). https://doi.org/10.1016/j.vaccine.2010.07.013

Rodríguez-Rieiro, C., Domínguez-Berjón, M. F., Esteban-Vasallodomínguez-Berjón, M. D., Cuadrado, A. R., Carrasco-Garrido, P., & Jiménez-García, R. (2011). Coverage and predictors of influenza vaccine uptake among adults aged 16 to 59 years suffering from a chronic condition in Madrid, Spain. Human Vaccines, 7(5). https://doi.org/10.4161/hv.7.5.14984

Rodríguez-Rieiro, C., Esteban-Vasallo, M. D., Domínguez-Berjón, M. F., Astray-Mochales, J., Iniesta-Fornies, D., Barranco-Ordoñez, D., Cameno-Heras, M., & Jiménez-García, R. (2011). Coverage and predictors of

vaccination against 2009 pandemic H1N1 influenza in Madrid, Spain. Vaccine, 29(6), 1332–1338. https://doi.org/10.1016/j.vaccine.2010.10.061

Rodríguez-Rieiro, C., Hernández-Barrera, V., Carrasco-Garrido, P., de Andres, A. L., & Jiménez-García, R. (2011). Vaccination against 2008/2009 and 2009/2010 seasonal influenza in Spain : Coverage among high risk subjects, HCWs, immigrants and time trends from the 2005/2006 campaign. Vaccine, 29(35). https://doi.org/10.1016/j.vaccine.2011.06.030

Roederer, T., Mollo, B., Vincent, C., Leduc, G., Sayyad, J., Mosnier, M., & Vandentorren, S. (2022). Drivers and prevalence of COVID-19 vaccine uptake among homeless and precariously housed people in France : A cross-sectional population-based study. medRxiv, (Roederer T., thomas.roederer@epicentre.msf.org; Mollo B.; Vincent C.; Leduc G.; Sayyad J.) Epicentre, Paris, France. https://doi.org/10.1101/2022.07.18.22276918

Roederer, T., Mollo, B., Vincent, C., Leduc, G., Sayyad-Hilario, J., Mosnier, M., & Vandentorren, S. (2023). Estimating COVID-19 vaccine uptake and its drivers among migrants, homeless and precariously housed people in France. Communications Medicine, 3(1). https://doi.org/10.1038/s43856-023-00257-1

Roederer, T., Mollo, B., Vincent, C., Nikolay, B., Llosa, A. E., Nesbitt, R., Vanhomwegen, J., Rose, T., Goyard, S., Anna, F., Torre, C., Fourrey, E., Simons, E., Hennequin, W., Mills, C., & Luquero, F. J. (2021). Seroprevalence and risk factors of exposure to COVID-19 in homeless people in Paris, France : A cross-sectional study. The Lancet. Public Health, 6(4). https://doi.org/10.1016/S2468-2667(21)00001-3

Roel, E., Pistillo, A., Recalde, M., Sena, A. G., Fernández-Bertolín, S., Aragón, M., Puente, D., Ahmed, W.-U.-R., Alghoul, H., Alser, O., Alshammari, T. M., Areia, C., Blacketer, C., Carter, W., Casajust, P., Culhane, A. C., Dawoud, D., DeFalco, F., DuVall, S. L., … Duarte-Salles, T. (2021). Characteristics and Outcomes of Over 300,000 Patients with COVID-19 and History of Cancer in the United States and Spain. Cancer Epidemiology, Biomarkers & Prevention: A Publication of the American Association for Cancer Research, Cosponsored by the American Society of Preventive Oncology, 30(10). https://doi.org/10.1158/1055-9965.EPI-21-0266

Roelofs, B., Ballas, D., Haisma, H., & Edzes, A. (2022). Spatial mobility patterns and COVID-19 incidence : A regional analysis of the second wave in the Netherlands. REGIONAL SCIENCE POLICY AND PRACTICE, 14. https://doi.org/10.1111/rsp3.12575

Roessler, M., Tesch, F., Batram, M., Jacob, J., Loser, F., Weidinger, O., Wende, D., Vivirito, A., Toepfner, N., Ehm, F., Seifert, M., Nagel, O., König, C., Jucknewitz, R., Armann, J. P., Berner, R., Treskova-Schwarzbach, M., Hertle, D., Scholz, S., … Schmitt, J. (2022). Post-COVID-19-associated morbidity in children, adolescents, and adults : A matched cohort study including more than 157,000 individuals with COVID-19 in Germany. PLoS Medicine, 19(11). https://doi.org/10.1371/journal.pmed.1004122

Rohleder, S., & Bozorgmehr, K. (2021). Monitoring the spatiotemporal epidemiology of Covid-19 incidence and mortality : A small-area analysis in Germany. Spatial and Spatio-Temporal Epidemiology, 38. https://doi.org/10.1016/j.sste.2021.100433

Rohleder, S., Costa, D. D., & Bozorgmehr, P. K. (2022). Area-level socioeconomic deprivation, non-national residency, and Covid-19 incidence : A longitudinal spatiotemporal analysis in Germany. EClinicalMedicine, 49. https://doi.org/10.1016/j.eclinm.2022.101485

Roig-Sánchez, N., Talaya Peñalver, A., Poveda Ruiz, N., Del Pozo, A., Hernández Campillo, A. M., Pérez Bernabéu, A., Martínez-López, B., González-Cuello, I., García-López, M., Borrajo Brunete, E., Wikman-Jorgensen, P., & Llenas-García, J. (2024). [COVID-19 readmissions during the first three epidemic periods in Orihuela, Spain : Incidence, risk factors and letality]. Revista Espanola De Salud Publica, 98. http://www.ncbi.nlm.nih.gov/pubmed/38516897

Rojas-Quiroz, J., & Marmolejo-Duarte, C. (2022). Determining Equality of Infection Rates : A Spatial Analysis of Factors Associated with the Spread of COVID-19 in Barcelona, Spain. JOURNAL OF URBAN PLANNING AND DEVELOPMENT, 148(3). https://doi.org/10.1061/(ASCE)UP.1943-5444.0000848

Rolland, S., Nguyen, L. L., Descamps, A., Galtier, F., Duval, X., Vanhems, P., Lainé, F., Tattevin, P., Bauer, R., Launay, O., & FLUVAC study group. (2025). Influenza and pneumococcal vaccine coverage among adults hospitalised with acute respiratory infection in France : A prospective cohort study. International Journal of

Infectious Diseases: IJID: Official Publication of the International Society for Infectious Diseases. https://doi.org/10.1016/j.ijid.2025.107811

Rollier, M., Miranda, G. H. B., Vergeynst, J., Meys, J., Alleman, T. W., Belgian Collaborative Group on covid-19 Hospital Surveillance, & Baetens, J. M. (2023). Mobility and the spatial spread of sars-cov-2 in Belgium. Mathematical Biosciences, 360. https://doi.org/10.1016/j.mbs.2022.108957

Romain-Scelle, N., Riche, B., Benet, T., & Rabilloud, M. (2025). Predictive quality of census-based socio-economic indicators on Covid-19 infection risk at a fine spatial scale in France. Scientific Reports, 15(1), 22076. https://doi.org/10.1038/s41598-025-03768-0

Romanowska, M., Nowak, I., Rybicka, K., & Brydak, L. B. (2008). The introduction of the SENTINEL influenza surveillance system in Poland—Experiences and lessons learned from the first three epidemic seasons. Euro Surveillance: Bulletin Europeen Sur Les Maladies Transmissibles = European Communicable Disease Bulletin, 13(8). http://www.ncbi.nlm.nih.gov/pubmed/18445411

Romanowska, M., Stefańska, I., & Brydak, L. B. (2013). Influenza and influenza-like illnesses in Poland in the epidemic season 2010/2011 according to the SENTINEL influenza surveillance system. Polish Journal of Microbiology, 62(1). http://www.ncbi.nlm.nih.gov/pubmed/23829077

Romanowska, M., Zycińska, K., Rybicka, K., & Brydak, L. B. (2007). The assessment of influenza virus activity in Poland in the epidemic season 2006/2007 on the basis of data from the SENTINEL influenza surveillance system. Family Medicine and Primary Care Review, 9(3). Embase. https://www.embase.com/search/results?subaction=viewrecord&id=L47610404&from=export

Romero García, C., Iftimi, A., Briz-Redón, Á., Zanin, M., Otero, M., Ballester, M., de Andrés, J., Landoni, G., de Las Marinas, D., Catalá Bauset, J. C., Mandingorra, J., Conca, J., Correcher, J., Ferrer, C., & Lozano, M. (2021). Trends in Incidence and Transmission Patterns of COVID-19 in Valencia, Spain. JAMA Network Open, 4(6). https://doi.org/10.1001/jamanetworkopen.2021.13818

Rommel, A., Lippe, E. von der, Plass, D., Ziese, T., Diercke, M., Heiden, M. A. der, Haller, S., Wengler, A., & BURDEN 2020 Study Group. (2021). The COVID-19 Disease Burden in Germany in 2020—Years of Life Lost to Death and Disease Over the Course of the Pandemic. Deutsches Arzteblatt International, 118(9), 145–151. https://doi.org/10.3238/arztebl.m2021.0147

Rommel, A., von der Lippe, E., Plass, D., Ziese, T., Diercke, M., an der Heiden, M., Haller, S., Wengler, A., & BURDEN 2020 Study Grp. (2021). The COVID-19 Disease Burden in Germany in 2020. DEUTSCHES ARZTEBLATT INTERNATIONAL, 118(9). https://doi.org/10.3238/arztebl.m2021.0147

Roncati, L., Galeazzi, C., Bartolacelli, G., & Caramaschi, S. (2024). A Real-World Nationwide Study on COVID-19 Trend in Italy during the Autumn-Winter Season of 2020 (before Mass Vaccination) and 2021 (after Mass Vaccination) Integrated with a Retrospective Analysis of the Mortality Burden per Year. Microorganisms, 12(3). https://doi.org/10.3390/microorganisms12030435

Roques, L., Klein, E. K., Papaïx, J., Sar, A., & Soubeyrand, S. (2020). Impact of Lockdown on the Epidemic Dynamics of COVID-19 in France. Frontiers in Medicine, 7. https://doi.org/10.3389/fmed.2020.00274

Rosano, A., Bella, A., Gesualdo, F., Acampora, A., Pezzotti, P., Marchetti, S., Ricciardi, W., & Rizzo, C. (2019). Investigating the impact of influenza on excess mortality in all ages in Italy during recent seasons (2013/14-2016/17 seasons). International Journal of Infectious Diseases: IJID: Official Publication of the International Society for Infectious Diseases, 88. https://doi.org/10.1016/j.ijid.2019.08.003

Rose, M. A., Damm, O., Greiner, W., Knuf, M., Wutzler, P., Liese, J. G., Krüger, H., Wahn, U., Schaberg, T., Schwehm, M., Kochmann, T. F., & Eichner, M. (2014). The epidemiological impact of childhood influenza vaccination using live-attenuated influenza vaccine (LAIV) in Germany : Predictions of a simulation study. BMC Infectious Diseases, 14. https://doi.org/10.1186/1471-2334-14-40

Rose, M. A., Stoermann, J., Bittner-Brewe, J., Rosewich, M., Eickmeier, O., & Schulze, J. (2013). Effectiveness, tolerability and patient satisfaction of paediatric live-attenuated influenza immunization (LAIV) in routine-care in Germany : A case-control-study. Trials in Vaccinology, 2(1). Embase. https://doi.org/10.1016/j.trivac.2013.09.003

Rosenkilde, S., Ekholm, O., Møller, S. P., Nielsen, M. B. D., & Thygesen, L. C. (2023). Factors related to COVID-19 vaccine hesitancy in Denmark : A national study in the autumn of 2021. Scandinavian Journal of Public Health, 51(5). https://doi.org/10.1177/14034948221144661

Rosillo, N., Del-Águila-Mejía, J., Rojas-Benedicto, A., Guerrero-Vadillo, M., Peñuelas, M., Mazagatos, C., Segú-Tell, J., Ramis, R., & Gómez-Barroso, D. (2021). Real time surveillance of COVID-19 space and time clusters during the summer 2020 in Spain. BMC Public Health, 21(1). https://doi.org/10.1186/s12889-021-10961-z

Rosner, B. M., Falkenhorst, G., Kumpf, I., Enßle, M., Hicketier, A., Dörre, A., Stark, K., & Wilking, H. (2024). Case-control study of behavioural and societal risk factors for sporadic SARS-CoV-2 infections, Germany, 2020-2021 (CoViRiS study). Epidemiology and Infection, 152. https://doi.org/10.1017/S0950268824000050

Rosolen, V., Turoldo, F., Zamaro, G., Del Bianco, F., Pezzotti, P., Castriotta, L., & Barbone, F. (2023). COVID-19 vaccination effectiveness in the population of Friuli Venezia Giulia, North-East Italy. Control of bias associated with divergent compliance to policies in a test-negative case-control study. BMC PUBLIC HEALTH, 23(1). https://doi.org/10.1186/s12889-023-17244-9

Roso-Llorach, A., Serra-Picamal, X., Cos, F. X., Pallejà-Millán, M., Mateu, L., Rosell, A., Almirante, B., Ferrer, J., Gasa, M., Gudiol, C., Moreno, A. M., Morales-Rull, J. L., Rexach, M., Sabater, G., Auguet, T., Vidal, F., Lerida, A., Rebull, J., Khunti, K., … Paredes, R. (2022). Evolving mortality and clinical outcomes of hospitalized subjects during successive COVID-19 waves in Catalonia, Spain. Global Epidemiology, 4. https://doi.org/10.1016/j.gloepi.2022.100071

Rossi, M., D'Avenio, G., La Rosa, G., Ferraro, G., Mancini, P., Veneri, C., Iaconelli, M., Lucentini, L., Bonadonna, L., Cerroni, M., Simonetti, F., Brandtner, D., Suffredini, E., Grigioni, M., Ahamed, SI, Ardagna, CA, Bian, … F. (2022). Surveillance of SARS-CoV-2 in Urban Wastewater in Italy. 2022 IEEE INTERNATIONAL CONFERENCE ON DIGITAL HEALTH (IEEE ICDH 2022). https://doi.org/10.1109/ICDH55609.2022.00026

Rößler, S., Ankert, J., Baier, M., Pletz, M. W., & Hagel, S. (2021). Influenza-associated in-hospital mortality during the 2017/2018 influenza season : A retrospective multicentre cohort study in central Germany. Infection, 49(1). https://doi.org/10.1007/s15010-020-01529-x

Rossmann, K., Grossmann, G., Frangoulidis, D., Clasen, R., Munch, M., Hasenknopf, M., Wurzbacher, C., Tiehm, A., Stange, C., Ho, J., Woermann, M., & Drewes, J. (2022). Innovative SARS-CoV-2 crisis management in the public health sector : Corona dashboard and wastewater surveillance using the example of Berchtesgadener Land, Germany. BUNDESGESUNDHEITSBLATT-GESUNDHEITSFORSCHUNG-GESUNDHEITSSCHUTZ, 65(3). https://doi.org/10.1007/s00103-021-03425-7

Rostila, M., Cederström, A., Wallace, M., Aradhya, S., Ahrne, M., & Juárez, S. P. (2023). Inequalities in COVID-19 severe morbidity and mortality by country of birth in Sweden. Nature Communications, 14(1). https://doi.org/10.1038/s41467-023-40568-4

Rothan-Tondeur, M., de Wazieres, B., Lejeune, B., Gavazzi, G., & Observatoire pour le Risque Infectieux en Gériatrie Association. (2006). Influenza vaccine coverage for healthcare workers in geriatric settings in France. Aging Clinical and Experimental Research, 18(6). https://doi.org/10.1007/BF03324852

Rousseau, M.-C., Hully, M., Milh, M., Juzeau, D., Pollez, B., Peudenier, S., Bahi Buisson, N., Gautheron, V., French Polyhandicap (PLH), C. O. G., Chabrol, B., & Billette de Villemeur, T. (2021). Clinical characteristics of COVID-19 infection in polyhandicapped persons in France. Archives De Pediatrie: Organe Officiel De La Societe Francaise De Pediatrie, 28(5). https://doi.org/10.1016/j.arcped.2021.04.004

Rouveix, E., Greffe, S., Dupont, C., Gherissi Cherni, D., Beauchet, A., Sordet Guepet, H., Gavazzi, G., Gaillat, J., & Intergroupe Société de pathologie infectieuse de langue française/Société de gériatrie et gérontologie clinique (Spilf/SGGC). (2013). [Low vaccination coverage against influenza in elderly hospitalized in France]. La Revue De Medecine Interne, 34(12). https://doi.org/10.1016/j.revmed.2013.02.006

Roux, J., Massonnaud, C. R., Colizza, V., Cauchemez, S., & Crépey, P. (2023). Modeling the impact of national and regional lockdowns on the 2020 spring wave of COVID-19 in France. Scientific Reports, 13(1). https://doi.org/10.1038/s41598-023-28687-w

Rovetta, A., & Bhagavathula, A. S. (2022). The Impact of COVID-19 on Mortality in Italy : Retrospective Analysis of Epidemiological Trends. JMIR Public Health and Surveillance, 8(4). https://doi.org/10.2196/36022

Rovetta, A., & Castaldo, L. (2020). Relationships between Demographic, Geographic, and Environmental Statistics and the Spread of Novel Coronavirus Disease (COVID-19) in Italy. Cureus, 12(11). https://doi.org/10.7759/cureus.11397

Rovida, F., Cassaniti, I., Percivalle, E., Sarasini, A., Paolucci, S., Klersy, C., Cutti, S., Novelli, V., Marena, C., Luzzaro, F., De Vito, G., Schiavo, R., Lo Cascio, G., Lilleri, D., & Baldanti, F. (2021). Incidence of SARS-CoV-2 infection in health care workers from Northern Italy based on antibody status : Immune protection from secondary infection- A retrospective observational case-controlled study. International Journal of Infectious Diseases: IJID: Official Publication of the International Society for Infectious Diseases, 109. https://doi.org/10.1016/j.ijid.2021.07.003

Rovida, F., Esposito, G. L., Rissone, M., Novelli, V., Cutti, S., Muzzi, A., Rona, C., Bertoli, E., Daglio, M., Piralla, A., Paolucci, S., Campanini, G., Ferrari, G., Giardina, F., Zavaglio, F., Lilleri, D., Grugnetti, A. M., Grugnetti, G., Odone, A., … Baldanti, F. (2022). Characteristics and outcomes of vaccinated and nonvaccinated patients hospitalized in a single Italian hub for COVID-19 during the Delta and Omicron waves in Northern Italy. International Journal of Infectious Diseases: IJID: Official Publication of the International Society for Infectious Diseases, 122. https://doi.org/10.1016/j.ijid.2022.06.028

Rueca, M., Berno, G., Agresta, A., Spaziante, M., Gruber, C. E. M., Fabeni, L., Giombini, E., Butera, O., Barca, A., Scognamiglio, P., Girardi, E., Maggi, F., Valli, M. B., Vairo, F., & Sars-CoV-Lazio Genomic Surveillance Study Group, null. (2023). Genomic and Epidemiologic Surveillance of SARS-CoV-2 in the Pandemic Period: Sequencing Network of the Lazio Region, Italy. Viruses, 15(11), 2192. https://doi.org/10.3390/v15112192

Ruiz Azcona, L., Roman-Rodriguez, M., Llort Bove, M., van Boven, J. F., & Santibáñez Margüello, M. (2020). Prevalence of Seasonal Influenza Vaccination in Chronic Obstructive Pulmonary Disease (COPD) Patients in the Balearic Islands (Spain) and Its Effect on COPD Exacerbations : A Population-Based Retrospective Cohort Study. International Journal of Environmental Research and Public Health, 17(11). https://doi.org/10.3390/ijerph17114027

Ruiz-Aragón, J., & Márquez-Peláez, S. (2023). An Economic Comparison in the Elderly of Adjuvanted Quadrivalent Influenza Vaccine with Recombinant Quadrivalent Influenza Vaccine in Spain. Vaccines, 11(2). https://doi.org/10.3390/vaccines11020427

Ruiz-Aragón, J., Gani, R., Márquez, S., & Alvarez, P. (2020). Estimated cost-effectiveness and burden of disease associated with quadrivalent cell-based and egg-based influenza vaccines in Spain. Human Vaccines & Immunotherapeutics, 16(9). https://doi.org/10.1080/21645515.2020.1712935

Ruiz-Aragón, J., Márquez-Peláez, S., Gani, R., Alvarez, P., & Guerrero-Luduena, R. (2022). Cost-Effectiveness and Burden of Disease for Adjuvanted Quadrivalent Influenza Vaccines Compared to High-Dose Quadrivalent Influenza Vaccines in Elderly Patients in Spain. Vaccines, 10(2). https://doi.org/10.3390/vaccines10020176

Ruiz-Montero, R., Ortiz-González Serna, R., Guzmán-Herrador, B. R., Barranco Quintana, J. L., Gavilán León, F. J., Salcedo Leal, I., Torcello Gaspar, R., Romero Muñoz, M. J., & Díaz Molina, C. (2020). Suboptimal influenza vaccine uptake among healthcare workers in a regional reference hospital in Spain, 2017–2018. Need for new approaches. Vacunas, 21(1). Embase. https://doi.org/10.1016/j.vacun.2019.12.001

Rumpler, R., Venkataraman, S., & Göransson, P. (2020). An observation of the impact of CoViD-19 recommendation measures monitored through urban noise levels in central Stockholm, Sweden. Sustainable Cities and Society, 63. https://doi.org/10.1016/j.scs.2020.102469

Russo, E., Esposito, P., Taramasso, L., Magnasco, L., Saio, M., Briano, F., Russo, C., Dettori, S., Vena, A., Di Biagio, A., Garibotto, G., Bassetti, M., Viazzi, F., & GECOVID working group. (2021). Kidney disease and all-

cause mortality in patients with COVID-19 hospitalized in Genoa, Northern Italy. Journal of Nephrology, 34(1). https://doi.org/10.1007/s40620-020-00875-1

Russo, P., Tacconelli, E., Olimpieri, P. P., Celant, S., Colatrella, A., Tomassini, L., & Palù, G. (2022). Mortality in SARS-CoV-2 Hospitalized Patients Treated with Remdesivir : A Nationwide, Registry-Based Study in Italy. Viruses, 14(6). https://doi.org/10.3390/v14061197

Rutter, M., Lanyon, P. C., Grainge, M. J., Hubbard, R., Bythell, M., Stilwell, P., Aston, J., McPhail, S., Stevens, S., & Pearce, F. A. (2023). COVID-19 infection, admission and death and the impact of corticosteroids among people with rare autoimmune rheumatic disease during the second wave of COVID-19 in England : Results from the RECORDER Project. Rheumatology (Oxford, England), 62(12). https://doi.org/10.1093/rheumatology/kead150

Rutter, P. D., Mytton, O. T., Mak, M., & Donaldson, L. J. (2012). Socio-economic disparities in mortality due to pandemic influenza in England. International Journal of Public Health, 57(4), 745–750. https://doi.org/10.1007/s00038-012-0337-1

Rybak, A., Cohen, R., Bangert, M., Kramer, R., Delobbe, J.-F., Deberdt, P., Cahn-Sellem, F., Béchet, S., & Levy, C. (2024). Assessing the Burden of Respiratory Syncytial Virus-related Bronchiolitis in Primary Care and at 15-Day and 6-Month Follow-up Before Prophylaxis in France : A Test-negative Study. The Pediatric Infectious Disease Journal, 43(7). https://doi.org/10.1097/INF.0000000000004360

Rybak, A., Ouldali, N., Werner, A., Casha, P., Robert, B., De Pontual, L., Béchet, S., Cahn-Sellem, F., Angoulvant, F., Cohen, R., Levy, C., Anxionnat, R., Auburtin, B., Azemar, B., Barrey, C., Belgaid, A., Benkaddouss, S., Benoist, G., Bensaid, P., … Gajdos, V. (2025). Characteristics of Children Hospitalized for Acute COVID-19 in France From February 2020 to December 2023. Pediatric Infectious Disease Journal, 44(4), 346–350. https://doi.org/10.1097/INF.0000000000004681

Rypdal, M., Rypdal, K., Løvsletten, O., Sørbye, S. H., Ytterstad, E., & Bianchi, F. M. (2021). Estimation of Excess Mortality and Years of Life Lost to COVID-19 in Norway and Sweden between March and November 2020. International Journal of Environmental Research and Public Health, 18(8). https://doi.org/10.3390/ijerph18083913

Rząd, M., Kanecki, K., Lewtak, K., Tyszko, P., Szwejkowska, M., Goryński, P., & Nitsch-Osuch, A. (2022). Human Respiratory Syncytial Virus Infections among Hospitalized Children in Poland during 2010-2020 : Study Based on the National Hospital Registry. Journal of Clinical Medicine, 11(21). https://doi.org/10.3390/jcm11216451

Rzymski, P., Pleśniak, R., Piekarska, A., Sznajder, D., Moniuszko-Malinowska, A., Tomasiewicz, K., Skwara, P., Zarębska-Michaluk, D., Turzańska, K., Piasecki, M., Hlebowicz, J., Sikorska, K., Mazur, M., Pazgan-Simon, M., & Flisiak, R. (2025). Tracking clinical severity of influenza in adult hospitalized patients in 2024: Data from the FluTer registry in Poland. Vaccine, 61, 127443. https://doi.org/10.1016/j.vaccine.2025.127443

Rzymski, P., Poniedziałek, B., Rosińska, J., Rogalska, M., Zarębska-Michaluk, D., Rorat, M., Moniuszko-Malinowska, A., Lorenc, B., Kozielewicz, D., Piekarska, A., Sikorska, K., Dworzańska, A., Bolewska, B., Angielski, G., Kowalska, J., Podlasin, R., Oczko-Grzesik, B., Mazur, W., Szymczak, A., & Flisiak, R. (2022). The association of airborne particulate matter and benzo[a]pyrene with the clinical course of COVID-19 in patients hospitalized in Poland. Environmental Pollution (Barking, Essex: 1987), 306. https://doi.org/10.1016/j.envpol.2022.119469

Sá, F. (2022). Do vaccinations reduce inequality in Covid-19 mortality ? Evidence from England. Social Science & Medicine (1982), 305. https://doi.org/10.1016/j.socscimed.2022.115072

Saad, N. J., Moek, F., Steitz, F., Murajda, L., Bärnighausen, T., Zoller, T., Pörtner, K., & Muller, N. (2021). A longitudinal study on symptom duration and 60-day clinical course in non-hospitalised COVID-19 cases in Berlin, Germany, March to May, 2020. Euro Surveillance: Bulletin Europeen Sur Les Maladies Transmissibles = European Communicable Disease Bulletin, 26(43). https://doi.org/10.2807/1560-7917.ES.2021.26.43.2001757

Saadatian-Elahi, M., Amour, S., Elias, C., Henaff, L., Dananché, C., & Vanhems, P. (2021). Tobacco smoking and severity of COVID-19 : Experience from a hospital-based prospective cohort study in Lyon, France. JOURNAL OF MEDICAL VIROLOGY, 93(12). https://doi.org/10.1002/jmv.27233

Saadatian-Elahi, M., Henaff, L., Elias, C., Nunes, M. C., Hot, A., Martin-Gaujard, G., Escuret, V., Amour, S., & Vanhems, P. (2023). Patient influenza vaccination reduces the risk of hospital-acquired influenza : An incident test negative-case control study in Lyon university hospital, France (2004-2020). Vaccine, 41(30). https://doi.org/10.1016/j.vaccine.2023.05.060

Saarinen, S., Moustgaard, H., Remes, H., Sallinen, R., & Martikainen, P. (2022). Income differences in COVID-19 incidence and severity in Finland among people with foreign and native background : A population-based cohort study of individuals nested within households. PLoS Medicine, 19(8). https://doi.org/10.1371/journal.pmed.1004038

Saavedra, P., Santana, A., Bello, L., Pacheco, J.-M., & Sanjuán, E. (2021). A Bayesian spatio-temporal analysis of mortality rates in Spain : Application to the COVID-19 2020 outbreak. Population Health Metrics, 19(1). https://doi.org/10.1186/s12963-021-00259-y

Sacco, C., Del Manso, M., Mateo-Urdiales, A., Rota, M. C., Petrone, D., Riccardo, F., Bella, A., Siddu, A., Battilomo, S., Proietti, V., Popoli, P., Menniti Ippolito, F., Palamara, A. T., Brusaferro, S., Rezza, G., Pezzotti, P., Fabiani, M., & Italian National COVID-19 Integrated Surveillance System and the Italian COVID-19 vaccines registry. (2022). Effectiveness of BNT162b2 vaccine against SARS-CoV-2 infection and severe COVID-19 in children aged 5-11 years in Italy : A retrospective analysis of January-April, 2022. Lancet (London, England), 400(10346). https://doi.org/10.1016/S0140-6736(22)01185-0

Sacco, C., Mateo-Urdiales, A., Petrone, D., Spuri, M., Fabiani, M., Vescio, M. F., Bressi, M., Riccardo, F., Del Manso, M., Bella, A., Pezzotti, P., & Italian Integrated Surveillance of COVID-19 study group. (2021). Estimating averted COVID-19 cases, hospitalisations, intensive care unit admissions and deaths by COVID-19 vaccination, Italy, January-September 2021. Euro Surveillance: Bulletin Europeen Sur Les Maladies Transmissibles = European Communicable Disease Bulletin, 26(47). https://doi.org/10.2807/1560-7917.ES.2021.26.47.2101001

Sacco, C., Petrone, D., Del Manso, M., Mateo-Urdiales, A., Fabiani, M., Bressi, M., Bella, A., Pezzotti, P., Rota, M. C., Riccardo, F., & Italian Integrated Surveillance of COVID-19 study group. (2022). Risk and protective factors for SARS-CoV-2 reinfections, surveillance data, Italy, August 2021 to March 2022. Euro Surveillance: Bulletin Europeen Sur Les Maladies Transmissibles = European Communicable Disease Bulletin, 27(20). https://doi.org/10.2807/1560-7917.ES.2022.27.20.2200372

Sacerdote, C., Castiglione, A., Pagano, E., Migliore, E., Pivetta, E., Auzzas, G. M., Brenstisci, C., Brunetti, F., Dafilé, C., Gangemi, M., Giacometti, L., Gilardetti, M., Martinis, V. H., Saccona, F., Stura, A., Turco, D., Balestro, C., Zozzoli, S., Filandra, U., … Ciccone, G. (2020). Clinical and epidemiological characteristics associated with pneumonia at disease onset in patients admitted for COVID-19 to the Emergency Department of a large Hospital in Piedmont (North-Western Italy). Epidemiologia E Prevenzione, 44(5-6 Suppl 2). https://doi.org/10.19191/EP20.5-6.S2.121

Sachedina, N., & Donaldson, L. J. (2010). Paediatric mortality related to pandemic influenza A H1N1 infection in England: An observational population-based study. Lancet (London, England), 376(9755), 1846–1852. https://doi.org/10.1016/S0140-6736(10)61195-6

Saez, M., Tobias, A., & Barceló, M. A. (2020). Effects of long-term exposure to air pollutants on the spatial spread of COVID-19 in Catalonia, Spain. Environmental Research, 191. https://doi.org/10.1016/j.envres.2020.110177

Saez, M., Tobias, A., Varga, D., & Barceló, M. A. (2020). Effectiveness of the measures to flatten the epidemic curve of COVID-19. The case of Spain. The Science of the Total Environment, 727. https://doi.org/10.1016/j.scitotenv.2020.138761

Sagoschen, I., Keller, K., Wild, J., Münzel, T., & Hobohm, L. (2022). Case Fatality of Hospitalized Patients with COVID-19 Infection Suffering from Acute Respiratory Distress Syndrome in Germany. Viruses, 14(11). https://doi.org/10.3390/v14112515

Salerno, L., Craxì, L., Amodio, E., & Lo Coco, G. (2021). Factors Affecting Hesitancy to mRNA and Viral Vector COVID-19 Vaccines among College Students in Italy. Vaccines, 9(8). https://doi.org/10.3390/vaccines9080927

Salgueira, M., Almenara, M., Gutierrez-Pizarraya, A., Belmar, L., Labrador, P. J., Melero, R., Serrano, M., Portolés, J. M., Molina, A., Poch, E., Ramos, N., Lloret, M. J., Echarri, R., Díaz-Mancebo, R., González-Lara, D. M., Sánchez, J. E., & Soler, M. J. (2024). Characterization of hospitalized patients with acute kidney injury associated with COVID-19 in Spain : Renal replacement therapy and mortality. FRA-COVID SEN Registry Data. Nefrologia, 44(4). https://doi.org/10.1016/j.nefroe.2023.03.017

Salinas-Botrán, A., Sanz-Cánovas, J., Pérez-Somarriba, J., Pérez-Belmonte, L. M., Cobos-Palacios, L., Rubio-Rivas, M., de-Cossío-Tejido, S., Ramos-Rincón, J. M., Méndez-Bailón, M., Gómez-Huelgas, R., & SEMI-COVID-19 group. (2022). Clinical characteristics and risk factors for mortality upon admission in patients with heart failure hospitalized due to COVID-19 in Spain. Revista Clinica Espanola, 222(5). https://doi.org/10.1016/j.rceng.2021.06.004

Salje, H., Tran Kiem, C., Lefrancq, N., Courtejoie, N., Bosetti, P., Paireau, J., Andronico, A., Hozé, N., Richet, J., Dubost, C.-L., Le Strat, Y., Lessler, J., Levy-Bruhl, D., Fontanet, A., Opatowski, L., Boelle, P.-Y., & Cauchemez, S. (2020). Estimating the burden of SARS-CoV-2 in France. Science (New York, N.Y.), 369(6500). https://doi.org/10.1126/science.abc3517

Salo, H., Lehtonen, T., Auranen, K., Baum, U., & Leino, T. (2022). Predictors of hospitalisation and death due to SARS-CoV-2 infection in Finland : A population-based register study with implications to vaccinations. Vaccine, 40(24). https://doi.org/10.1016/j.vaccine.2022.04.055

Saltyte Benth, J., & Hofoss, D. (2008). Modelling and prediction of weekly incidence of influenza A specimens in England and Wales. Epidemiology and Infection, 136(12). https://doi.org/10.1017/S0950268808000307

Salvadore, F., Fiscon, G., & Paci, P. (2021). Integro-differential approach for modeling the COVID-19 dynamics—Impact of confinement measures in Italy. Computers in Biology and Medicine, 139. https://doi.org/10.1016/j.compbiomed.2021.105013

Samaras, L., García-Barriocanal, E., & Sicilia, M.-A. (2017). Syndromic Surveillance Models Using Web Data : The Case of Influenza in Greece and Italy Using Google Trends. JMIR Public Health and Surveillance, 3(4). https://doi.org/10.2196/publichealth.8015

San Román Montero, J., Gil-Prieto, R., Martín, R. J., de Lejarazu, R. O., Gallardo-Pino, C., & Gil de Miguel, A. (2021). Influenza hospitalizations in children under 1 year old in Spain : The importance of maternal immunization. Human Vaccines & Immunotherapeutics, 17(6). https://doi.org/10.1080/21645515.2020.1845523

Sanchez-Luna, M., Burgos-Pol, R., Oyagüez, I., Figueras-Aloy, J., Sánchez-Solís, M., Martinón-Torres, F., & Carbonell-Estrany, X. (2017). Cost-utility analysis of Palivizumab for Respiratory Syncytial Virus infection prophylaxis in preterm infants : Update based on the clinical evidence in Spain. BMC Infectious Diseases, 17(1). https://doi.org/10.1186/s12879-017-2803-0

Sanchez-Piedra, C., Cruz-Cruz, C., Gamiño-Arroyo, A.-E., & Prado-Galbarro, F.-J. (2021). Effects of air pollution and climatology on COVID-19 mortality in Spain. Air Quality, Atmosphere, & Health, 14(11). https://doi.org/10.1007/s11869-021-01062-2

Sandrini, M., Andreano, A., Murtas, R., Tunesi, S., Riussi, A., Guido, D., Greco, M. T., Gattoni, M. E., Gervasi, F., Consolazio, D., Adreoni, L., Decarli, A., & Russo, A. G. (2020). Assessment of the Overall Mortality during the COVID-19 Outbreak in the Provinces of Milan and Lodi (Lombardy Region, Northern Italy). Epidemiologia E Prevenzione, 44(5-6 Suppl 2). https://doi.org/10.19191/EP20.5-6.S2.124

Sangalli, D., Martinelli-Boneschi, F., Versino, M., Colombo, I., Ciccone, A., Beretta, S., Marcheselli, S., Altavilla, R., Roncoroni, M., Beretta, S., Lorusso, L., Cavallini, A., Prelle, A., Guidetti, D., La Gioia, S., Santalucia, P., Zanferrari, C., Grampa, G., D'Adda, E., … SNO-COVID-19 group. (2021). Impact of SARS-CoV-2 infection on acute intracerebral haemorrhage in northern Italy. Journal of the Neurological Sciences, 426. https://doi.org/10.1016/j.jns.2021.117479

Sankatsing, V. D. V., van Summeren, J., Abreha, F. M., Pandolfi, E., Chironna, M., Loconsole, D., Kramer, R., Paget, J., & Rizzo, C. (2025). Economic Impact of Respiratory Syncytial Virus Infections in Children Under 5 Years of Age Attending Primary Care in Italy : A Prospective Cohort Study in Two Regions. Influenza and Other Respiratory Viruses, 19(2). https://doi.org/10.1111/irv.70074

Sanmarchi, F., Capodici, A., Golinelli, D., Lenzi, J., Zamparini, M., Toth, F., De Girolamo, G., & Stoto, M. A. (2025). Regional variations in Italy's COVID-19 death toll: A descriptive analysis of excess mortality and associated factors from 2020 to 2021. Population Health Metrics, 23(1), 9. https://doi.org/10.1186/s12963-025-00370-4

San-Román-Montero, J. M., Gil Prieto, R., Gallardo Pino, C., Hinojosa Mena, J., Zapatero Gaviria, A., & Gil de Miguel, A. (2019). Inpatient hospital fatality related to coding (ICD-9-CM) of the influenza diagnosis in Spain (2009-2015). BMC Infectious Diseases, 19(1). https://doi.org/10.1186/s12879-019-4308-5

Sant Fruchtman, C., Fischer, F. B., Monzón Llamas, L., Tavakkoli, M., Cobos Muñoz, D., & Antillon, M. (2022). Did COVID-19 Policies Have the Same Effect on COVID-19 Incidence Among Women and Men ? Evidence From Spain and Switzerland. International Journal of Public Health, 67. https://doi.org/10.3389/ijph.2022.1604994

Santamaría, L., & Hortal, J. (2021). COVID-19 effective reproduction number dropped during Spain's nationwide dropdown, then spiked at lower-incidence regions. The Science of the Total Environment, 751. https://doi.org/10.1016/j.scitotenv.2020.142257

Santa-Olalla Peralta, P., Cortes García, M., Martínez Sánchez, E. V., Nogareda Moreno, F., Limia Sánchez, A., Pachón Del Amo, I., Sierra Moros, M. J., & Subcomité de Vigilancia Epidemiológica del Plan Nacional de Preparación y Respuesta ante una Pand... (2010). [Enhanced surveillance of initial cases of pandemic influenza (H1N1) 2009 infection in Spain, April-June 2009]. Revista Espanola De Salud Publica, 84(5), 529–546. https://doi.org/10.1590/s1135-57272010000500007

Santa-Olalla, P., Cortes-García, M., Vicente-Herrero, M., Castrillo-Villamandos, C., Arias-Bohigas, P., Pachon-del Amo, I., Sierra-Moros, M., & New Influenza A H1N1 Virus Invest. (2010). Risk factors for disease severity among hospitalised patients with 2009 pandemic influenza A (H1N1) in Spain, April—December 2009. EUROSURVEILLANCE, 15(38), 9–17.

Santella, B., Aliberti, S. M., Fortino, L., Donato, A., Andretta, V., Santoro, E., Franci, G., Capunzo, M., & Boccia, G. (2024). Age Differences and Prevalence of Comorbidities for Death and Survival in Patients with COVID-19 : A Single-Center Observational Study in a Region of Southern Italy. Life (Basel, Switzerland), 14(11). https://doi.org/10.3390/life14111376

Santos-Hövener, C., Busch, M. A., Koschollek, C., Schlaud, M., Hoebel, J., Hoffmann, R., Wilking, H., Haller, S., Allen, J., Wernitz, J., Butschalowsky, H., Kuttig, T., Stahlberg, S., Strandmark, J., Rosario, A. S., Gößwald, A., Nitsche, A., Hamouda, O., Drosten, C., … Lampert, T. (2020). Seroepidemiological study on the spread of SARS-CoV-2 in populations in especially affected areas in Germany—Study protocol of the CORONA-MONITORING lokal study. Journal of Health Monitoring, 5(Suppl 5). https://doi.org/10.25646/7053

Santos-Hövener, C., Neuhauser, H. K., Rosario, A. S., Busch, M., Schlaud, M., Hoffmann, R., Gößwald, A., Koschollek, C., Hoebel, J., Allen, J., Haack-Erdmann, A., Brockmann, S., Ziese, T., Nitsche, A., Michel, J., Haller, S., Wilking, H., Hamouda, O., Corman, V. M., … Lampert, T. (2020). Serology- and PCR-based cumulative incidence of SARS-CoV-2 infection in adults in a successfully contained early hotspot (CoMoLo study), Germany, May to June 2020. Euro Surveillance: Bulletin Europeen Sur Les Maladies Transmissibles = European Communicable Disease Bulletin, 25(47). https://doi.org/10.2807/1560-7917.ES.2020.25.47.2001752

Santos-Sancho, J. M., López-de Andrés, A., Jimenez-Trujillo, I., Hernández-Barrera, V., Carrasco-Garrido, P., Astasio-Arbiza, P., & Jimenez-Garcia, R. (2013). Adherence and factors associated with influenza vaccination among subjects with asthma in Spain. Infection, 41(2). https://doi.org/10.1007/s15010-013-0414-2

Sanz-Muñoz, I., Arroyo-Hernantes, I., Martín-Toribio, A., Toquero-Asensio, M., Sánchez-Martínez, J., Rodríguez-Crespo, C., Rojo-Rello, S., Domínguez-Gil, M., Hernández-Pérez, M., Tamayo, E., Gil-Prieto, R., Gil-de-Miguel, Á., & Eiros, J. M. (2025). Disease burden of influenza in Spain : A five-season study (2015-2020). Human Vaccines & Immunotherapeutics, 21(1). https://doi.org/10.1080/21645515.2024.2440206

Sanz-Rojo, S., Jiménez-García, R., López-de-Andrés, A., de Miguel-Diez, J., Perez-Farinos, N., & Zamorano-León, J. J. (2021). Influenza vaccination uptake among high-risk target groups and health care workers in Spain and change from 2017 to 2020. Vaccine, 39(48). https://doi.org/10.1016/j.vaccine.2021.10.059

Sartini, M., Del Puente, F., Oliva, M., Carbone, A., Blasi Vacca, E., Parisini, A., Boni, S., Bobbio, N., Feasi, M., Battistella, A., Pontali, E., & Cristina, M. L. (2021). Riding the COVID Waves: Clinical Trends, Outcomes, and Remaining Pitfalls of the SARS-CoV-2 Pandemic: An Analysis of Two High-Incidence Periods at a Hospital in Northern Italy. Journal of Clinical Medicine, 10(22), 5239. https://doi.org/10.3390/jcm10225239

Sartorius, B., Lawson, A. B., & Pullan, R. L. (2021). Modelling and predicting the spatio-temporal spread of COVID-19, associated deaths and impact of key risk factors in England. Scientific Reports, 11(1). https://doi.org/10.1038/s41598-021-83780-2

Sauvage, E., Gehanno, J., Thomas, N., & Rollin, L. (2023). Prevalence of SARS-CoV-2, serological study in France in non-confined and confined employees. ARCHIVES DES MALADIES PROFESSIONNELLES ET DE L ENVIRONNEMENT, 84(4). https://doi.org/10.1016/j.admp.2023.101798

Savarese, G., Carpinelli, L., De Chiara, A., Giordano, C., Perillo, M., Fornino, D., De Caro, F., Capunzo, M., & Moccia, G. (2022). Anti-SARS-CoV-2 Vaccination Campaign : Risk Perception, Emotional States, and Vaccine Hesitancy in a Sample of Adolescents' Vaccinated Parents in Southern Italy. Vaccines, 10(6). https://doi.org/10.3390/vaccines10060958

Saviano, M., Fierro, A., & Liccardo, A. (2023). A deterministic compartmental model for the transition between variants in the spread of Covid-19 in Italy. PloS One, 18(11). https://doi.org/10.1371/journal.pone.0293416

Savoia, E., Harriman, N. W., Piltch-Loeb, R., Bonetti, M., Toffolutti, V., & Testa, M. A. (2022). Exploring the Association between Misinformation Endorsement, Opinions on the Government Response, Risk Perception, and COVID-19 Vaccine Hesitancy in the US, Canada, and Italy. Vaccines, 10(5). https://doi.org/10.3390/vaccines10050671

Savulescu, C., Jiménez-Jorge, S., de Mateo, S., Pozo, F., Casas, I., Breña, P. P., Galmés, A., Vanrell, J. M., Rodriguez, C., Vega, T., Martinez, A., Torner, N., Ramos, J. M., Serrano, M. C., Castilla, J., Cenoz, M. G., Altzibar, J. M., Arteagoitia, J. M., Quiñones, C., … Larrauri, A. (2011). Using surveillance data to estimate pandemic vaccine effectiveness against laboratory confirmed influenza A(H1N1)2009 infection: Two case-control studies, Spain, season 2009-2010. BMC Public Health, 11, 899. https://doi.org/10.1186/1471-2458-11-899

Savulescu, C., Jiménez-Jorge, S., de Mateo, S., Ledesma, J., Pozo, F., Casas, I., Larrauri, A., & cycEVA Study Team. (2011). Effectiveness of the 2010/11 seasonal trivalent influenza vaccine in Spain : Preliminary results of a case-control study. Euro Surveillance: Bulletin Europeen Sur Les Maladies Transmissibles = European Communicable Disease Bulletin, 16(11). https://doi.org/10.2807/ese.16.11.19820-en

Savulescu, C., Jiménez-Jorge, S., Delgado-Sanz, C., de Mateo, S., Pozo, F., Casas, I., Larrauri, A., & Spanish Influenza Surveillance System. (2014). Higher vaccine effectiveness in seasons with predominant circulation of seasonal influenza A(H1N1) than in A(H3N2) seasons : Test-negative case-control studies using surveillance data, Spain, 2003-2011. Vaccine, 32(35). https://doi.org/10.1016/j.vaccine.2014.06.063

Savulescu, C., Valenciano, M., de Mateo, S., Larrauri, A., & cycEVA Study Team. (2010). Estimating the influenza vaccine effectiveness in elderly on a yearly basis using the Spanish influenza surveillance network—Pilot case-control studies using different control groups, 2008-2009 season, Spain. Vaccine, 28(16). https://doi.org/10.1016/j.vaccine.2010.01.054

Sberna, G., Guarini, R., Vaia, F., Maggi, F., Bordi, L., & Covid-Saliva Laboratory team. (2022). Monitoring of SARS-CoV-2 circulation using saliva testing in school children in Rome, Italy. International Journal of Infectious Diseases: IJID: Official Publication of the International Society for Infectious Diseases, 124. https://doi.org/10.1016/j.ijid.2022.09.007

Sberna, G., Lalle, E., Valli, M. B., Bordi, L., Garbuglia, A. R., & Amendola, A. (2022). Changes in the Circulation of Common Respiratory Pathogens among Hospitalized Patients with Influenza-like Illnesses in the

Lazio Region (Italy) during Fall Season of the Past Three Years. International Journal of Environmental Research and Public Health, 19(10). https://doi.org/10.3390/ijerph19105962

Scarpaci, M., Bracaloni, S., Esposito, E., De Angelis, L., Baglivo, F., Casini, B., Panatto, D., Ogliastro, M., Loconsole, D., Chironna, M., Pariani, E., Pellegrinelli, L., Pandolfi, E., Croci, I., Rizzo, C., & RSVComNet Italia. (2024). RSV Disease Burden in Primary Care in Italy : A Multi-Region Pediatric Study, Winter Season 2022-2023. Influenza and Other Respiratory Viruses, 18(4). https://doi.org/10.1111/irv.13282

Scarpone, C., Brinkmann, S. T., Große, T., Sonnenwald, D., Fuchs, M., & Walker, B. B. (2020). A multimethod approach for county-scale geospatial analysis of emerging infectious diseases : A cross-sectional case study of COVID-19 incidence in Germany. International Journal of Health Geographics, 19(1). https://doi.org/10.1186/s12942-020-00225-1

Schäfer, M., Wijaya, K. P., Rockenfeller, R., & Götz, T. (2022). The impact of travelling on the COVID-19 infection cases in Germany. BMC Infectious Diseases, 22(1). https://doi.org/10.1186/s12879-022-07396-1

Schaffer, A. L., Hulme, W. J., Horne, E., Parker, E. P. K., Walker, V., Stables, C., Mehrkar, A., Bacon, S. C. J., Bates, C., Goldacre, B., Walker, A. J., Hernán, M. A., & Sterne, J. A. C. (2023). Effect of the 2022 COVID-19 booster vaccination campaign in 50 year olds in England : Regression discontinuity analysis in OpenSAFELY. medRxiv, (Schaffer A.L., andrea.schaffer@phc.ox.ac.uk; Hulme W.J.; Stables C.; Mehrkar A.; Bacon S.C.J.; Goldacre B.; Walker A.J.) Bennett Institute for Applied Data Science, Nuffield Department of Primary Care Health Sciences, University of Oxford, Oxford, United Kingdom. https://doi.org/10.1101/2023.09.07.23295194

Schaffer, A. L., Hulme, W. J., Horne, E., Parker, E. P. K., Walker, V., Stables, C., Mehrkar, A., Bacon, S. C. J., Bates, C., Goldacre, B., Walker, A. J., OpenSAFELY Collaborative, Hernán, M. A., & Sterne, J. A. C. (2025). Effect of the 2022 COVID-19 booster vaccination campaign in people aged 50 years in England: Regression discontinuity analysis in OpenSAFELY-TPP. Vaccine, 59, 127257. https://doi.org/10.1016/j.vaccine.2025.127257

Schaubroeck, H., Vandenberghe, W., Boer, W., Boonen, E., Dewulf, B., Bourgeois, C., Dubois, J., Dumoulin, A., Fivez, T., Gunst, J., Hermans, G., Lormans, P., Meersseman, P., Mesotten, D., Stessel, B., Vanhoof, M., De Vlieger, G., & Hoste, E. (2022). Acute kidney injury in critical COVID-19 : A multicenter cohort analysis in seven large hospitals in Belgium. Critical Care (London, England), 26(1). https://doi.org/10.1186/s13054-022-04086-x

Schepp, R. M., Kaczorowska, J., van Gageldonk, P. G. M., Rouers, E. D. M., Sanders, E. A. M., Bruijning-Verhagen, P. C. J., & Berbers, G. A. M. (2023). Effect of Palivizumab Prophylaxis on Respiratory Syncytial Virus Infection in Very Preterm Infants in the First Year of Life in The Netherlands. Vaccines, 11(12). https://doi.org/10.3390/vaccines11121807

Scherbaum, R., Bartig, D., Richter, D., Kwon, E. H., Muhlack, S., Gold, R., Krogias, C., & Tönges, L. (2022). COVID-19 outcomes in hospitalized Parkinson’s disease patients in two pandemic waves in 2020: A nationwide cross-sectional study from Germany. Neurological Research and Practice, 4(1), 27. https://doi.org/10.1186/s42466-022-00192-x

Scherbaum, R., Kwon, E. H., Richter, D., Bartig, D., Gold, R., Krogias, C., & Tönges, L. (2021). Clinical Profiles and Mortality of COVID-19 Inpatients with Parkinson’s Disease in Germany. Movement Disorders: Official Journal of the Movement Disorder Society, 36(5). https://doi.org/10.1002/mds.28586

Schettino, M., Pellegrini, L., Picascia, D., Saibeni, S., Bezzio, C., Bini, F., Omazzi, B. F., Devani, M., Arena, I., Bongiovanni, M., Manes, G., & Della Corte, C. M. R. (2021). Clinical Characteristics of COVID-19 Patients With Gastrointestinal Symptoms in Northern Italy : A Single-Center Cohort Study. The American Journal of Gastroenterology, 116(2). https://doi.org/10.14309/ajg.0000000000000965

Schiaroli, E., De Socio, G. V., Martinelli, L., Malincarne, L., Savoia, M., Spinelli, A. L., & Francisci, D. (2021). Early Treatment with Bamlanivimab Alone does not Prevent COVID-19 Hospitalization and Its Post-Acute Sequelae. A Real Experience in Umbria, Italy. Mediterranean Journal of Hematology and Infectious Diseases, 13(1). https://doi.org/10.4084/MJHID.2021.061

Schindler, C. J. A., Wittenberg, I., Damm, O., Kramer, R., Mikolajczyk, R., & Schönfelder, T. (2024). Influenza-Associated Excess Mortality and Hospitalization in Germany from 1996 to 2018. Infectious Diseases and Therapy, 13(11). https://doi.org/10.1007/s40121-024-01043-9

Schmalhofer, C., Otte Im Kampe, E., Eheberg, D., Sandhu, H., Maier, M., Perschke, A., Mugwagwa, T., Fröling, E., & Kisser, A. (2025). Economic evaluation of oral Nirmatrelvir/ritonavir versus best supportive care in patients at high risk for progression to severe COVID-19 in Germany: A cost-effectiveness analysis. Journal of Medical Economics, 28(1), 1226–1240. https://doi.org/10.1080/13696998.2025.2536974

Schmidt, R., Majer, I., García Román, N., Rivas Basterra, A., Grubb, E., & Medrano López, C. (2017). Palivizumab in the prevention of severe respiratory syncytial virus infection in children with congenital heart disease; a novel cost-utility modeling study reflecting evidence-based clinical pathways in Spain. Health Economics Review, 7(1). https://doi.org/10.1186/s13561-017-0181-3

Schmidt, S. S. S., Iuliano, A. D., Vestergaard, L. S., Mazagatos-Ateca, C., Larrauri, A., Brauner, J. M., Olsen, S. J., Nielsen, J., Salomon, J. A., & Krause, T. G. (2022). All-cause versus cause-specific excess deaths for estimating influenza-associated mortality in Denmark, Spain, and the United States. Influenza and Other Respiratory Viruses, 16(4). https://doi.org/10.1111/irv.12966

Schmidt-Ott, R., Molnar, D., Anastassopoulou, A., Yanni, E., Krumm, C., Bekkat-Berkani, R., Dos Santos, G., Henneke, P., Knuf, M., Schwehm, M., & Eichner, M. (2020). Assessing direct and indirect effects of pediatric influenza vaccination in Germany by individual-based simulations. Human Vaccines & Immunotherapeutics, 16(4). https://doi.org/10.1080/21645515.2019.1682843

Schneider, P. P., van Gool, C. J., Spreeuwenberg, P., Hooiveld, M., Donker, G. A., Barnett, D. J., & Paget, J. (2020). Using web search queries to monitor influenza-like illness : An exploratory retrospective analysis, Netherlands, 2017/18 influenza season. Euro Surveillance: Bulletin Europeen Sur Les Maladies Transmissibles = European Communicable Disease Bulletin, 25(21). https://doi.org/10.2807/1560-7917.ES.2020.25.21.1900221

Schneider-Kamp, A. (2022). COVID-19 Vaccine Hesitancy in Denmark and Russia : A qualitative typology at the nexus of agency and health capital. SSM. Qualitative Research in Health, 2. https://doi.org/10.1016/j.ssmqr.2022.100116

Schoeps, A., Hoffmann, D., Tamm, C., Vollmer, B., Haag, S., Kaffenberger, T., Ferguson-Beiser, K., Kohlhase-Griebel, B., Basenach, S., Missal, A., Höfling, K., Michels, H., Schall, A., Kappes, H., Vogt, M., Jahn, K., Bärnighausen, T., & Zanger, P. (2021). Surveillance of SARS-CoV-2 transmission in educational institutions, August to December 2020, Germany. Epidemiology and Infection, 149. https://doi.org/10.1017/S0950268821002077

Schoeps, A., Walter, J., Vogt, M., Bent, S., Zanger, P., & Palatina Public Health Study Group. (2023). Direct and indirect vaccination effects on SARS-CoV-2 infection in day-care centres : Evaluating the policy for early vaccination of day-care staff in Germany, 2021. Epidemiology and Infection, 151. https://doi.org/10.1017/S0950268823000638

Scholz, S. M., Weidemann, F., Damm, O., Ultsch, B., Greiner, W., & Wichmann, O. (2021). Cost-Effectiveness of Routine Childhood Vaccination Against Seasonal Influenza in Germany. Value in Health: The Journal of the International Society for Pharmacoeconomics and Outcomes Research, 24(1). https://doi.org/10.1016/j.jval.2020.05.022

Scholz, S., Damm, O., Schneider, U., Ultsch, B., Wichmann, O., & Greiner, W. (2019). Epidemiology and cost of seasonal influenza in Germany—A claims data analysis. BMC Public Health, 19(1). https://doi.org/10.1186/s12889-019-7458-x

Scholz, S., Dobrindt, K., Tufts, J., Adams, S., Ghaswalla, P., Ultsch, B., & Gottlieb, J. (2024). The Burden of Respiratory Syncytial Virus (RSV) in Germany : A Comprehensive Data Analysis Suggests Underdetection of Hospitalisations and Deaths in Adults 60 Years and Older. Infectious Diseases and Therapy, 13(8). https://doi.org/10.1007/s40121-024-01006-0

Schrell, S., Ziemann, A., Garcia-Castrillo Riesgo, L., Rosenkötter, N., Llorca, J., Popa, D., Krafft, T., & SIDARTHa Project Consortium. (2013). Local implementation of a syndromic influenza surveillance system

using emergency department data in Santander, Spain. Journal of Public Health (Oxford, England), 35(3). https://doi.org/10.1093/pubmed/fdt043

Schulte, M., Leithäuser, N., & Mohring, J. (2025). Estimating the Effect of Self-Protection on Transmission Dynamics of SARS-CoV-2 in Germany in 2021. SSRN. https://doi.org/10.2139/ssrn.5389499

Schuppert, A., Polotzek, K., Karschau, J., & Karagiannidis, C. (2021). Effectiveness of extended shutdown measures during the ´Bundesnotbremse´ introduced in the third SARS-CoV-2 wave in Germany. Infection, 49(6). https://doi.org/10.1007/s15010-021-01713-7

Schwarzinger, M., Luchini, S., Teschl, M., Alla, F., Mallet, V., & Rehm, J. (2023). Mental disorders, COVID-19-related life-saving measures and mortality in France : A nationwide cohort study. PLoS Medicine, 20(2). https://doi.org/10.1371/journal.pmed.1004134

Schwarzinger, M., Watson, V., Arwidson, P., Alla, F., & Luchini, S. (2021). COVID-19 vaccine hesitancy in a representative working-age population in France : A survey experiment based on vaccine characteristics. The Lancet. Public Health, 6(4). https://doi.org/10.1016/S2468-2667(21)00012-8

Sciannameo, V., Goffi, A., Maffeis, G., Gianfreda, R., Jahier Pagliari, D., Filippini, T., Mancuso, P., Giorgi-Rossi, P., Alberto Dal Zovo, L., Corbari, A., Vinceti, M., & Berchialla, P. (2022). A deep learning approach for Spatio-Temporal forecasting of new cases and new hospital admissions of COVID-19 spread in Reggio Emilia, Northern Italy. Journal of Biomedical Informatics, 132. https://doi.org/10.1016/j.jbi.2022.104132

Scohy, A., Gruson, D., Simon, A., Kabamba-Mukadi, B., De Greef, J., Belkhir, L., Rodriguez-Villalobos, H., Robert, A., & Yombi, J. C. (2021). Seroprevalence of SARS-CoV-2 infection in health care workers of a teaching hospital in Belgium : Self-reported occupational and household risk factors for seropositivity. Diagnostic Microbiology and Infectious Disease, 100(4). https://doi.org/10.1016/j.diagmicrobio.2021.115414

Scortichini, M., Schneider Dos Santos, R., De' Donato, F., De Sario, M., Michelozzi, P., Davoli, M., Masselot, P., Sera, F., & Gasparrini, A. (2021). Excess mortality during the COVID-19 outbreak in Italy : A two-stage interrupted time-series analysis. International Journal of Epidemiology, 49(6). https://doi.org/10.1093/ije/dyaa169

Scozzari, G., Costa, C., Migliore, E., Coggiola, M., Ciccone, G., Savio, L., Scarmozzino, A., Pira, E., Cassoni, P., Galassi, C., Cavallo, R., & The Collaborative Group, null. (2021). Prevalence, Persistence, and Factors Associated with SARS-CoV-2 IgG Seropositivity in a Large Cohort of Healthcare Workers in a Tertiary Care University Hospital in Northern Italy. Viruses, 13(6). https://doi.org/10.3390/v13061064

Sebastiani, G., Massa, M., & Riboli, E. (2020). Covid-19 epidemic in Italy : Evolution, projections and impact of government measures. European Journal of Epidemiology, 35(4). https://doi.org/10.1007/s10654-020-00631-6

Seghezzo, G., Allen, H., Griffiths, C., Pooley, J., Beardsmore, L., Caul, S., Glickman, M., Clare, T., Dabrera, G., & Kall, M. (2024). Comparison of two COVID-19 mortality measures used during the pandemic response in England. International Journal of Epidemiology, 53(1), dyad116. https://doi.org/10.1093/ije/dyad116

Seidl, C., Coyer, L., Ackermann, N., Katz, K., Walter, J., Ippisch, S., Hoch, M., & Böhmer, M. M. (2023). SARS-CoV-2 Prevalence on and Incidence after Arrival in Travelers on Direct Flights from Cape Town, South Africa to Munich, Germany Shortly after Occurrence of the Omicron Variant in November/December 2021 : Results from the OMTRAIR Study. Pathogens (Basel, Switzerland), 12(2). https://doi.org/10.3390/pathogens12020354

Semenzato, L., Botton, J., Drouin, J., Baricault, B., Bertrand, M., Jabagi, M.-J., Cuenot, F., Vu, S. L., Dray-Spira, R., Weill, A., & Zureik, M. (2022). Characteristics associated with the residual risk of severe COVID-19 after a complete vaccination schedule : A cohort study of 28 million people in France. The Lancet Regional Health. Europe, 19. https://doi.org/10.1016/j.lanepe.2022.100441

Semenzato, L., Botton, J., Drouin, J., Cuenot, F., Dray-Spira, R., Weill, A., & Zureik, M. (2021). Chronic diseases, health conditions and risk of COVID-19-related hospitalization and in-hospital mortality during the first wave of the epidemic in France : A cohort study of 66 million people. The Lancet Regional Health. Europe, 8. https://doi.org/10.1016/j.lanepe.2021.100158

Serisier, A., Beale, S., Boukari, Y., Hoskins, S., Nguyen, V., Byrne, T., Fong, W. L. E., Fragaszy, E., Geismar, C., Kovar, J., Yavlinsky, A., Hayward, A., & Aldridge, R. W. (2023). A case-crossover study of the effect of vaccination on SARS-CoV-2 transmission relevant behaviours during a period of national lockdown in England and Wales. Vaccine, 41(2). https://doi.org/10.1016/j.vaccine.2022.11.073

Serraino, D., Zucchetto, A., Dal Maso, L., Del Zotto, S., Taboga, F., Clagnan, E., Fratino, L., Tosolini, F., & Burba, I. (2021). Prevalence, determinants, and outcomes of SARS-COV-2 infection among cancer patients. A population-based study in northern Italy. Cancer Medicine, 10(21). https://doi.org/10.1002/cam4.4271

Serrano-Cumplido, A., Ruíz-García, A., del Rio-Herrero, A., Antón-Eguía, P., Micó-Pérez, R., Calderón-Montero, A., Romero-Rodríguez, E., Segura-Fragoso, A., & Martín-Sánchez, V. (2024). Effect of the Omicron variant on cumulative incidence of infection and lethality during the sixth wave of the COVID-19 epidemic in Spain. MEDICINA DE FAMILIA-SEMERGEN, 50(2). https://doi.org/10.1016/j.semerg.2023.102073

Serrano-Ortiz, Á., Romero-Cabrera, J. L., Monserrat Villatoro, J., Cordero-Ramos, J., Ruiz-Montero, R., Ritoré, Á., Dopazo, J., Del Diego Salas, J., García Sánchez, V., Salcedo-Leal, I., Armengol de la Hoz, M. Á., Túnez, I., & Guzmán, M. Á. (2024). Assessing COVID-19 Vaccine Effectiveness and Risk Factors for Severe Outcomes through Machine Learning Techniques : A Real-World Data Study in Andalusia, Spain. Journal of Epidemiology and Global Health, 14(4). https://doi.org/10.1007/s44197-024-00298-2

Servais, T., Laurent, F., Roland, T., Rossi, C., De Groote, E., Godart, V., Repetto, E., Ponchon, M., Chasseur, P., Crenier, L., Van Eeckhoudt, S., Yango, J., Oriot, P., Morisca Gavriliu, M., Rouhard, S., Deketelaere, B., Maiter, D., Hermans, M. P., Yombi, J. C., & Orioli, L. (2024). Mortality-related risk factors of inpatients with diabetes and COVID-19 : A multicenter retrospective study in Belgium. Annales D'endocrinologie, 85(1). https://doi.org/10.1016/j.ando.2023.08.002

Servia-Dopazo, M., Purriños-Hermida, M. J., Pérez, S., García, J., Malvar-Pintos, A., en nombre del Grupo del Sistema de Vigilancia Microbiológica de la Gripe en Galicia, & Los miembros del Grupo del Sistema de Vigilancia Microbiológica de la Gripe en Galicia que han contribuido a redactar este artículo son. (2020). [Usefulness of the microbiological surveillance of respiratory syncytial virus in Galicia (Spain) : 2008-2017]. Gaceta Sanitaria, 34(5). https://doi.org/10.1016/j.gaceta.2018.11.009

Serwin, K., Ossowski, A., Szargut, M., Cytacka, S., Urbańska, A., Majchrzak, A., Niedźwiedź, A., Czerska, E., Pawińska-Matecka, A., Gołąb, J., & Parczewski, M. (2021). Molecular Evolution and Epidemiological Characteristics of SARS COV-2 in (Northwestern) Poland. Viruses, 13(7). https://doi.org/10.3390/v13071295

Setbon, M., Le Pape, M.-C., Létroublon, C., Caille-Brillet, A.-L., & Raude, J. (2011). The public's preventive strategies in response to the pandemic influenza A/H1N1 in France: Distribution and determinants. Preventive Medicine, 52(2), 178–181. https://doi.org/10.1016/j.ypmed.2010.11.010

Setti, L., Passarini, F., De Gennaro, G., Barbieri, P., Licen, S., Perrone, M., Piazzalunga, A., Borelli, M., Palmisani, J., Di Gilio, A., Rizzo, E., Colao, A., Piscitelli, P., & Miani, A. (2020). Potential role of particulate matter in the spreading of COVID-19 in Northern Italy : First observational study based on initial epidemic diffusion. BMJ OPEN, 10(9). https://doi.org/10.1136/bmjopen-2020-039338

Shaaban, A. N., Andersson, F., Peña, S., Caspersen, I. H., Magnusson, C., Orsini, N., Karvonen, S., Magnus, P., Hergens, M. P., & Galanti, M. R. (2023). The Association Between Tobacco Use and Risk of COVID-19 Infection and Clinical Outcomes in Sweden : A Population-Based Study. International Journal of Public Health, 68. https://doi.org/10.3389/ijph.2023.1606175

Shahzad, K., Shahzad, U., Iqbal, N., Shahzad, F., & Fareed, Z. (2020). Effects of climatological parameters on the outbreak spread of COVID-19 in highly affected regions of Spain. Environmental Science and Pollution Research International, 27(31). https://doi.org/10.1007/s11356-020-10551-3

Shang, H. L., & Xu, R. (2022). Change point detection for COVID-19 excess deaths in Belgium. Journal of Population Research (Canberra, A.C.T.), 39(4). https://doi.org/10.1007/s12546-021-09256-2

Sharp, A., Minaji, M., Panagiotopoulos, N., Reeves, R., Charlett, A., & Pebody, R. (2022). Estimating the burden of adult hospital admissions due to RSV and other respiratory pathogens in England. Influenza and Other Respiratory Viruses, 16(1). https://doi.org/10.1111/irv.12910

Shedrawy, J., Ernst, P., Lönnroth, K., & Nyberg, F. (2023). The burden of disease due to COVID-19 in Sweden 2020-2021 : A disability-adjusted life years (DALYs) study. Scandinavian Journal of Public Health, 51(5). https://doi.org/10.1177/14034948231160616

Shen, J. (2020). A recursive bifurcation model for early forecasting of COVID-19 virus spread in South Korea and Germany. Scientific Reports, 10(1). https://doi.org/10.1038/s41598-020-77457-5

Shen, Q., Joyce, E. E., Ebrahimi, O. V., Didriksen, M., Lovik, A., Sævarsdóttir, K. S., Magnúsdóttir, I., Mikkelsen, D. H., Unnarsdóttir, A. B., Hauksdóttir, A., Hoffart, A., Kähler, A. K., Thórdardóttir, E. B., Eythórsson, E., Frans, E. M., Tómasson, G., Ask, H., Hardardóttir, H., Jakobsdóttir, J., … Valdimarsdóttir, U. A. (2023). COVID-19 illness severity and 2-year prevalence of physical symptoms : An observational study in Iceland, Sweden, Norway and Denmark. The Lancet Regional Health. Europe, 35. https://doi.org/10.1016/j.lanepe.2023.100756

Sherratt, K., Abbott, S., Meakin, S. R., Hellewell, J., Munday, J. D., Bosse, N., CMMID COVID-19 Working Group, Jit, M., & Funk, S. (2021). Exploring surveillance data biases when estimating the reproduction number : With insights into subpopulation transmission of COVID-19 in England. Philosophical Transactions of the Royal Society of London. Series B, Biological Sciences, 376(1829). https://doi.org/10.1098/rstb.2020.0283

Shojaee, S., Pourhoseingholi, M. A., Ashtari, S., Vahedian-Azimi, A., Asadzadeh-Aghdaei, H., & Zali, M. R. (2020). Predicting the mortality due to Covid-19 by the next month for Italy, Iran and South Korea; a simulation study. Gastroenterology and Hepatology from Bed to Bench, 13(2). http://www.ncbi.nlm.nih.gov/pubmed/32308940

Shorten, R. J., Haslam, S., Hurley, M. A., Rowbottom, A., Myers, M., Wilkinson, P., & Orr, D. (2021). Seroprevalence of SARS-CoV-2 infection in healthcare workers in a large teaching hospital in the North West of England : A period prevalence survey. BMJ Open, 11(3). https://doi.org/10.1136/bmjopen-2020-045384

Shrotri, M., Krutikov, M., Nacer-Laidi, H., Azmi, B., Palmer, T., Giddings, R., Fuller, C., Irwin-Singer, A., Baynton, V., Tut, G., Moss, P., Hayward, A., Copas, A., & Shallcross, L. (2022). Duration of vaccine effectiveness against SARS-CoV-2 infection, hospitalisation, and death in residents and staff of long-term care facilities in England (VIVALDI) : A prospective cohort study. The Lancet. Healthy Longevity, 3(7). https://doi.org/10.1016/S2666-7568(22)00147-7

Shrotri, M., Krutikov, M., Palmer, T., Giddings, R., Azmi, B., Subbarao, S., Fuller, C., Irwin-Singer, A., Davies, D., Tut, G., Lopez Bernal, J., Moss, P., Hayward, A., Copas, A., & Shallcross, L. (2021). Vaccine effectiveness of the first dose of ChAdOx1 nCoV-19 and BNT162b2 against SARS-CoV-2 infection in residents of long-term care facilities in England (VIVALDI) : A prospective cohort study. The Lancet. Infectious Diseases, 21(11). https://doi.org/10.1016/S1473-3099(21)00289-9

Shroufi, A., Copping, J., Vivancos, R., & Slack, R. C. (2008). Influenza and pneumococcal vaccine uptake among nursing home residents in Nottingham, England : A postal questionnaire survey. BMC Geriatrics, 8. https://doi.org/10.1186/1471-2318-8-11

Shtele, E., Beria, P., & Lunkar, V. (2022). Using location-based social media data to explain COVID-19 spread in Italy. EUROPEAN JOURNAL OF TRANSPORT AND INFRASTRUCTURE RESEARCH, 22(2). https://doi.org/10.18757/ejtir.2022.22.2.5702

Shubin, M., Lebedev, A., Lyytikäinen, O., & Auranen, K. (2016). Revealing the True Incidence of Pandemic A(H1N1)pdm09 Influenza in Finland during the First Two Seasons—An Analysis Based on a Dynamic Transmission Model. PLoS Computational Biology, 12(3), e1004803. https://doi.org/10.1371/journal.pcbi.1004803

Shubin, M., Virtanen, M., Toikkanen, S., Lyytikäinen, O., & Auranen, K. (2014). Estimating the burden of A(H1N1)pdm09 influenza in Finland during two seasons. Epidemiology and Infection, 142(5). https://doi.org/10.1017/S0950268813002537

Sikkema, R. S., Pas, S. D., Nieuwenhuijse, D. F., O'Toole, Á., Verweij, J., van der Linden, A., Chestakova, I., Schapendonk, C., Pronk, M., Lexmond, P., Bestebroer, T., Overmars, R. J., van Nieuwkoop, S., van den Bijllaardt, W., Bentvelsen, R. G., van Rijen, M. M. L., Buiting, A. G. M., van Oudheusden, A. J. G.,

Diederen, B. M., … Koopmans, M. P. G. (2020). COVID-19 in health-care workers in three hospitals in the south of the Netherlands : A cross-sectional study. The Lancet. Infectious Diseases, 20(11). https://doi.org/10.1016/S1473-3099(20)30527-2

Siljander, M., Uusitalo, R., Pellikka, P., Isosomppi, S., & Vapalahti, O. (2022). Spatiotemporal clustering patterns and sociodemographic determinants of COVID-19 (SARS-CoV-2) infections in Helsinki, Finland. Spatial and Spatio-Temporal Epidemiology, 41. https://doi.org/10.1016/j.sste.2022.100493

Silva, M. L., Perrier, L., Späth, H.-M., Grog, I., Mosnier, A., Havet, N., Cohen, J. M., & IBGP team. (2014). Economic burden of seasonal influenza B in France during winter 2010-2011. BMC Public Health, 14. https://doi.org/10.1186/1471-2458-14-56

Silvennoinen, H., Peltola, V., Vainionpää, R., Ruuskanen, O., & Heikkinen, T. (2011). Incidence of influenza-related hospitalizations in different age groups of children in Finland : A 16-year study. The Pediatric Infectious Disease Journal, 30(2). https://doi.org/10.1097/inf.0b013e3181fe37c8

Silvestri, M., Marando, F., Costanzo, A. M., di Luzio Paparatti, U., & Rossi, G. A. (2016). Respiratory Syncytial Virus-associated hospitalization in premature infants who did not receive palivizumab prophylaxis in Italy : A retrospective analysis from the Osservatorio Study. Italian Journal of Pediatrics, 42. https://doi.org/10.1186/s13052-016-0252-9

Simón Méndez, L., López-Cuadrado, T., López Perea, N., Larrauri Cámara, A., & de Mateo Ontañón, S. (2012). [Premature mortality excess related to influenza in Spain during an interpandemic period]. Revista Espanola De Salud Publica, 86(2). https://doi.org/10.1590/S1135-57272012000200004

Simón Sacristán, M., Ybarra de Villavicencio, C., Collazos Blanco, A., Mayo Montero, M. E., Ariñez Fernández, M. D. C., Suárez Prieto, A., Zamora Cintas, M. I., & Mateo Maestre, M. (2021). SARS-COV-2 Infection and Specific Antibody Detection on Health Care Workers from a Military Hospital in Madrid, Spain. Current Microbiology, 78(8). https://doi.org/10.1007/s00284-021-02541-6

Sinnathamby, M. A., Warburton, F., Andrews, N., Boddington, N. L., Zhao, H., Ellis, J., Tessier, E., Donati, M., Elliot, A. J., Hughes, H. E., Byford, R., Smith, G. E., Tripathy, M., de Lusignan, S., Zambon, M., & Pebody, R. G. (2022). Uptake and impact of vaccinating primary school children against influenza : Experiences in the fourth season of the live attenuated influenza vaccination programme, England, 2016/2017. Influenza and Other Respiratory Viruses, 16(1). https://doi.org/10.1111/irv.12898

Sinnathamby, M., Twohig, K., Abdul Aziz, N., Halford, F., Zaidi, A., Harman, K., Thelwall, S., Allen, A., & Dabrera, G. (2025). Surveillance of International Travel of COVID-19 Cases (SuITCases) in England. Influenza and Other Respiratory Viruses, 19(8). https://doi.org/10.1111/irv.70141

Siqueira, C. A. D. S., Freitas, Y. N. L. de, Cancela, M. de C., Carvalho, M., Oliveras-Fabregas, A., & de Souza, D. L. B. (2020). The effect of lockdown on the outcomes of COVID-19 in Spain : An ecological study. PloS One, 15(7). https://doi.org/10.1371/journal.pone.0236779

Sisti, L. G., Di Napoli, A., Petrelli, A., Diodati, A., Cavani, A., Mirisola, C., & Costanzo, G. (2023). Newly arrived migrants did not represent an additional COVID-19 burden for Italy : Data from the italian information flow. Globalization and Health, 19(1). https://doi.org/10.1186/s12992-023-00926-9

Sjödin, H., Johansson, A. F., Brännström, Å., Farooq, Z., Kriit, H. K., Wilder-Smith, A., Åström, C., Thunberg, J., Söderquist, M., & Rocklöv, J. (2020). COVID-19 healthcare demand and mortality in Sweden in response to non-pharmaceutical mitigation and suppression scenarios. International Journal of Epidemiology, 49(5). https://doi.org/10.1093/ije/dyaa121

Sjögren, L., Stenberg, E., Thuccani, M., Martikainen, J., Rylander, C., Wallenius, V., Olbers, T., & Kindblom, J. M. (2021). Impact of obesity on intensive care outcomes in patients with COVID-19 in Sweden-A cohort study. PloS One, 16(10). https://doi.org/10.1371/journal.pone.0257891

Sjöström, B., Månsson, E., Viklund Kamienny, J., & Östberg, E. (2021). Characteristics and definitive outcomes of COVID-19 patients admitted to a secondary hospital intensive care unit in Sweden. Health Science Reports, 4(4). https://doi.org/10.1002/hsr2.446

Skogberg, N., Prinkey, T., Lilja, E., Koponen, P., & Castaneda, A. E. (2023). Association of sociodemographic characteristics with self-perceived access to COVID-19 information and adherence to preventive measures among migrant origin and general populations in Finland : A cross-sectional study. BMJ Open, 13(3). https://doi.org/10.1136/bmjopen-2022-069192

Slavec, A., Iwanowska, M., Bałandynowicz-Panfil, K., Olah, Șerban, Zvonar, M. Š., Štebe, J., & Łosiewicz, M. (2024). Determinants of COVID-19 vaccination intention in Central and Eastern Europe : A cross-sectional study in Poland, Romania, and Slovenia. Archives of Public Health = Archives Belges De Sante Publique, 82(1). https://doi.org/10.1186/s13690-024-01261-0

Slavec, A., Iwanowska, M., Bałandynowicz-Panfil, K., Olah, Șerban, Zvonar, M. Š., Štebe, J., & Łosiewicz, M. (2024). Determinants of COVID-19 vaccination intention in Central and Eastern Europe: A cross-sectional study in Poland, Romania, and Slovenia. Archives of Public Health = Archives Belges De Sante Publique, 82(1), 60. https://doi.org/10.1186/s13690-024-01261-0

Slim, M. A., Appelman, B., Peters-Sengers, H., Dongelmans, D. A., de Keizer, N. F., Schade, R. P., de Boer, M. G. J., Müller, M. C. A., Vlaar, A. P. J., Wiersinga, W. J., van Vught, L. A., & NICE COVID-19 Research Consortium and the COVIDPredict study group. (2022). Real-world Evidence of the Effects of Novel Treatments for COVID-19 on Mortality : A Nationwide Comparative Cohort Study of Hospitalized Patients in the First, Second, Third, and Fourth Waves in the Netherlands. Open Forum Infectious Diseases, 9(12). https://doi.org/10.1093/ofid/ofac632

Smagge, B., Labuschagne, L., Pijpers, J., van Roon, A., van den Hof, S., Hahné, S., & de Melker, H. (2024). Factors associated with lower COVID-19 vaccine uptake among populations with a migration background in the Netherlands. medRxiv, (Smagge B., bente.smagge@rivm.nl; Labuschagne L.; Pijpers J.; van Roon A.; den Hof S.; Hahné S.; de Melker H.) Centre for Infectious Disease Control, National Institute for Public Health and the Environment (RIVM), Bilthoven, Netherlands. https://doi.org/10.1101/2024.11.07.24316886

Smith, J. A. E., Hopkins, S., Turner, C., Dack, K., Trelfa, A., Peh, J., & Monks, P. S. (2022). Public health impact of mass sporting and cultural events in a rising COVID-19 prevalence in England. Epidemiology and Infection, 150. https://doi.org/10.1017/S0950268822000188

Smits, R. A. L., Trompet, S., van der Linden, C. M. J., van der Bol, J. M., Jansen, S. W. M., Polinder-Bos, H. A., Willems, H. C., Barten, D. G., Blomaard, L. C., de Boer, M. G. J., van Deudekom, F. J. A., Ellerbroek, J. L. J., Festen, J., van de Glind, E. M. M., Kampschreur, L. M., Karimi, O., Kroon, B., van Lanen, M. G. J. A., Lucke, J. A., … Mooijaart, S. P. (2022). Characteristics and outcomes of older patients hospitalised for COVID-19 in the first and second wave of the pandemic in The Netherlands: The COVID-OLD study. Age and Ageing, 51(3), afac048. https://doi.org/10.1093/ageing/afac048

Sofonea, M. T., Roquebert, B., Foulongne, V., Morquin, D., Verdurme, L., Trombert-Paolantoni, S., Roussel, M., Bonetti, J.-C., Zerah, J., Haim-Boukobza, S., & Alizon, S. (2022). Analyzing and Modeling the Spread of SARS-CoV-2 Omicron Lineages BA.1 and BA.2, France, September 2021-February 2022. Emerging Infectious Diseases, 28(7). https://doi.org/10.3201/eid2807.220033

Soldevila, N., Acosta, L., Martínez, A., Godoy, P., Torner, N., Rius, C., Jané, M., Domínguez, A., & Surveillance of Hospitalized Cases of Severe Influenza in Catalonia Working Group. (2021). Behavior of hospitalized severe influenza cases according to the outcome variable in Catalonia, Spain, during the 2017-2018 season. Scientific Reports, 11(1). https://doi.org/10.1038/s41598-021-92895-5

Song, F., & Bachmann, M. O. (2021). Vaccination against COVID-19 and society’s return to normality in England : A modelling study of impacts of different types of naturally acquired and vaccine-induced immunity. BMJ Open, 11(11). https://doi.org/10.1136/bmjopen-2021-053507

Sorg, A. L., Hufnagel, M., Doenhardt, M., Diffloth, N., Schroten, H., Kries, R. V., Berner, R., & Armann, J. (2021). Risk of hospitalization, severe disease, and mortality due to COVID-19 and PIMS-TS in children with SARS-CoV-2 infection in Germany. medRxiv, (Sorg A.L.; Kries R.V.) Institute of Social Paediatrics and Adolescent Medicine, Division of Paediatric Epidemiology, Ludwig-Maximilians-University Munich, Germany. https://doi.org/10.1101/2021.11.30.21267048

Soriano, A., Montejano, R., Sanz-Moreno, J., Figueira, J. C., Grau, S., Güerri-Fernández, R., Castro-Gómez, A., Pérez-Román, I., Hidalgo-Vega, Á., & González-Domínguez, A. (2021). Impact of Remdesivir on the Treatment of COVID-19 During the First Wave in Spain. Advances in Therapy, 38(7). https://doi.org/10.1007/s12325-021-01804-9

Soriano, J. B., Peláez, A., Fernández, E., Moreno, L., & Ancochea, J. (2022). The Emergence of COVID-19 as a Cause of Death in 2020 and its Effect on Mortality by Diseases of the Respiratory System in Spain : Trends and Their Determinants Compared to 2019. Archivos De Bronconeumologia, 58. https://doi.org/10.1016/j.arbres.2022.03.001

Sotoodeh, A., Hedberg, P., Granath, F., Alfvén, T., & Nauclér, P. (2024). Sociodemographic determinants of COVID-19 vaccination in adolescents in Stockholm, Sweden. Vaccine, 42(26). https://doi.org/10.1016/j.vaccine.2024.126388

Souris, M., & Gonzalez, J.-P. (2020). COVID-19 : Spatial analysis of hospital case-fatality rate in France. PloS One, 15(12). https://doi.org/10.1371/journal.pone.0243606

Souty, C., Blanchon, T., Bonmarin, I., Lévy-Bruhl, D., Behillil, S., Enouf, V., Valette, M., Bouscambert, M., Turbelin, C., Capai, L., Roussel, V., Hanslik, T., & Falchi, A. (2015). Early estimates of 2014/15 seasonal influenza vaccine effectiveness in preventing influenza-like illness in general practice using the screening method in France. Human Vaccines & Immunotherapeutics, 11(7). https://doi.org/10.1080/21645515.2015.1046661

Souty, C., Guerrisi, C., Masse, S., Lina, B., van der Werf, S., Bernard-Stoecklin, S., Turbelin, C., Falchi, A., Hanslik, T., & Blanchon, T. (2021). Impact of the lockdown on the burden of COVID-19 in outpatient care in France, spring 2020. Infectious Diseases (London, England), 53(5). https://doi.org/10.1080/23744235.2021.1880024

Souty, C., Vilcu, A.-M., Capai, L., van der Werf, S., Valette, M., Blanchon, T., Lina, B., Behillil, S., Hanslik, T., & Falchi, A. (2017). Early estimates of 2016/17 seasonal influenza vaccine effectiveness in primary care in France. Journal of Clinical Virology: The Official Publication of the Pan American Society for Clinical Virology, 95. https://doi.org/10.1016/j.jcv.2017.08.002

Sowa, P., Kiszkiel, Ł., Laskowski, P. P., Alimowski, M., Szczerbiński, Ł., Paniczko, M., Moniuszko-Malinowska, A., & Kamiński, K. (2021). COVID-19 Vaccine Hesitancy in Poland-Multifactorial Impact Trajectories. Vaccines, 9(8). https://doi.org/10.3390/vaccines9080876

Spaccaferri, G., Calba, C., Vilain, P., Garras, L., Durand, C., Pilorget, C., Atiki, N., Bernillon, P., Bosc, L., Fougère, E., Hanon, J.-B., Henry, V., Huchet-Kervella, C., Martel, M., Pontiès, V., Mouly, D., Rolland du Roscoat, E., Le Vu, S., Desenclos, J.-C., … Rolland, P. (2021). COVID-19 hotspots through clusters analysis in France (may-October 2020) : Where should we track the virus to mitigate the spread? BMC Public Health, 21(1). https://doi.org/10.1186/s12889-021-11857-8

Spetz, M., Lundberg, L., Nwaru, C., Li, H., Santosa, A., Leach, S., Gisslén, M., Hammar, N., Rosvall, M., & Nyberg, F. (2022). The social patterning of Covid-19 vaccine uptake in older adults : A register-based cross-sectional study in Sweden. The Lancet Regional Health. Europe, 15. https://doi.org/10.1016/j.lanepe.2022.100331

Spinella, C., & Mio, A. M. (2021). Simulation of the impact of people mobility, vaccination rate, and virus variants on the evolution of Covid-19 outbreak in Italy. Scientific Reports, 11(1). https://doi.org/10.1038/s41598-021-02546-y

Spreco, A., Andersson, C., Sjödahl, R., & Niward, K. (2025). 60-day mortality and the role of SARS-CoV-2 in hospital admissions of immunocompromised patients during later Omicron period: A population-based study in Sweden. Infectious Diseases (London, England), 57(6), 561–573. https://doi.org/10.1080/23744235.2025.2465828

Spreco, A., Andersson, C., Sjödahl, R., & Timpka, T. (2023). Concordance between COVID-19 mortality statistics derived from clinical audit and death certificates in Östergötland county, Sweden. Public Health, 221. https://doi.org/10.1016/j.puhe.2023.06.007

Spreco, A., Dahlström, Ö., Jöud, A., Nordvall, D., Fagerström, C., Blomqvist, E., Gustafsson, F., Hinkula, J., Schön, T., & Timpka, T. (2022). Effectiveness of the BNT162b2 mRNA Vaccine Compared with Hybrid Immunity in Populations Prioritized and Non-Prioritized for COVID-19 Vaccination in 2021-2022 : A Naturalistic Case-Control Study in Sweden. Vaccines, 10(8). https://doi.org/10.3390/vaccines10081273

Spreco, A., Jöud, A., Eriksson, O., Soltesz, K., Källström, R., Dahlström, Ö., Eriksson, H., Ekberg, J., Jonson, C.-O., Fraenkel, C.-J., Lundh, T., Gerlee, P., Gustafsson, F., & Timpka, T. (2022). Nowcasting (Short-Term Forecasting) of COVID-19 Hospitalizations Using Syndromic Healthcare Data, Sweden, 2020. Emerging Infectious Diseases, 28(3). https://doi.org/10.3201/eid2803.210267

Sprengholz, P., Korn, L., Eitze, S., Felgendreff, L., Siegers, R., Goldhahn, L., De Bock, F., Huebl, L., Böhm, R., & Betsch, C. (2022). Attitude toward a mandatory COVID-19 vaccination policy and its determinants: Evidence from serial cross-sectional surveys conducted throughout the pandemic in Germany. Vaccine, 40(51), 7370–7377. https://doi.org/10.1016/j.vaccine.2022.01.065

Spruit, J. R., Jansen, R. W. M. M., de Groot, J. R., de Vries, T. A. C., Hemels, M. E. W., Douma, R. A., de Haan, L. R., Brinkman, K., Moeniralam, H. S., de Kruif, M., Dormans, T., Appelman, B., Reidinga, A. C., Rusch, D., Gritters van den Oever, N. C., Schuurman, R. J., Beudel, M., & Simsek, S. (2023). Does atrial fibrillation affect prognosis in hospitalised COVID-19 patients ? A multicentre historical cohort study in the Netherlands. BMJ Open, 13(12). https://doi.org/10.1136/bmjopen-2022-071137

Squeri, R., Riso, R., Facciolà, A., Genovese, C., Palamara, M. a. R., Ceccio, C., & La Fauci, V. (2017). Management of two influenza vaccination campaign in health care workers of a university hospital in the south Italy. Annali Di Igiene: Medicina Preventiva E Di Comunita, 29(3). https://doi.org/10.7416/ai.2017.2150

Srivastav, A., Stollenwerk, N., & Aguiar, M. (2022). Deterministic and Stochastic Dynamics of COVID-19 : The Case Study of Italy and Spain. COMPUTATIONAL AND MATHEMATICAL METHODS, 2022. https://doi.org/10.1155/2022/5780719

Stabler, M., & Kuebart, A. (2023). Tempo-spatial dynamics of COVID-19 in Germany – A phase model based on a pandemic severity indicator. medRxiv. https://doi.org/10.1101/2023.02.17.23286084

Stafoggia, M., Ranzi, A., Ancona, C., Bauleo, L., Bella, A., Cattani, G., Nobile, F., Pezzotti, P., Iavarone, I., & EpiCovAir Study Group. (2023). Long-Term Exposure to Ambient Air Pollution and Mortality among Four Million COVID-19 Cases in Italy : The EpiCovAir Study. Environmental Health Perspectives, 131(5). https://doi.org/10.1289/EHP11882

Stańczak-Mrozek, K. I., Sobczak, A., Lipiński, L., Sienkiewicz, E., Makarewicz, D., Topór-Mądry, R., Pinkas, J., & Sierpiński, R. A. (2021). The Potential Benefits of the Influenza Vaccination on COVID-19 Mortality Rate-A Retrospective Analysis of Patients in Poland. Vaccines, 10(1). https://doi.org/10.3390/vaccines10010005

Stang, A., Standl, F., Kowall, B., Brune, B., Böttcher, J., Brinkmann, M., Dittmer, U., & Jöckel, K.-H. (2020). Excess mortality due to COVID-19 in Germany. The Journal of Infection, 81(5). https://doi.org/10.1016/j.jinf.2020.09.012

Stapic, M., Schulz, R. S., Tamayo-Cuartero, E., Kurth, T., & Brinks, R. (2025). Measuring the disease burden of seasonal influenza in Germany 2015—2020 using the incidence-based disability-adjusted life years (DALYs). BMC Infectious Diseases, 25(1), 413. https://doi.org/10.1186/s12879-025-10613-2

Stapper, M., & Funk, S. (2025). Fine-Grid Spatial Interaction Matrices for Surveillance Models, with Application to Influenza in Germany. medRxiv. https://doi.org/10.1101/2025.04.03.25325159

Steens, A., Waaijenborg, S., Teunis, P. F. M., Reimerink, J. H. J., Meijer, A., van der Lubben, M., Koopmans, M., van der Sande, M. A. B., Wallinga, J., & van Boven, M. (2011). Age-dependent patterns of infection and severity explaining the low impact of 2009 influenza A (H1N1) : Evidence from serial serologic surveys in the Netherlands. American Journal of Epidemiology, 174(11). https://doi.org/10.1093/aje/kwr245

Steensels, D., Oris, E., Coninx, L., Nuyens, D., Delforge, M.-L., Vermeersch, P., & Heylen, L. (2020). Hospital-Wide SARS-CoV-2 Antibody Screening in 3056 Staff in a Tertiary Center in Belgium. JAMA, 324(2). https://doi.org/10.1001/jama.2020.11160

Stefanati, A., Lupi, S., Campo, G., Cocchio, S., Furlan, P., Baldo, V., & Gabutti, G. (2020). Influenza coverage rates in subjects with chronic heart diseases : Results obtained in four consecutive immunisation seasons in the Local Health Unit of Ferrara (North Italy)". Archives of Public Health = Archives Belges De Sante Publique, 78. https://doi.org/10.1186/s13690-020-00487-y

Stefanelli, P., Bella, A., Fedele, G., Pancheri, S., Leone, P., Vacca, P., Neri, A., Carannante, A., Fazio, C., Benedetti, E., Fiore, S., Fabiani, C., Simmaco, M., Santino, I., Zuccali, M. G., Bizzarri, G., Magnoni, R., Benetollo, P. P., Merler, S., … Ferro, A. (2021). Prevalence of SARS-CoV-2 IgG antibodies in an area of northeastern Italy with a high incidence of COVID-19 cases : A population-based study. Clinical Microbiology and Infection: The Official Publication of the European Society of Clinical Microbiology and Infectious Diseases, 27(4). https://doi.org/10.1016/j.cmi.2020.11.013

Stefanelli, P., Trentini, F., Petrone, D., Mammone, A., Ambrosio, L., Manica, M., Guzzetta, G., d’Andrea, V., Marziano, V., Zardini, A., Molina Grane’, C., Ajelli, M., Di Martino, A., Riccardo, F., Bella, A., Sane Schepisi, M., Maraglino, F., Poletti, P., Palamara, A. T., … Genomic SARS-CoV-2 National Surveillance Working Group. (2022). Tracking the progressive spread of the SARS-CoV-2 Omicron variant in Italy, December 2021 to January 2022. Euro Surveillance: Bulletin Europeen Sur Les Maladies Transmissibles = European Communicable Disease Bulletin, 27(45). https://doi.org/10.2807/1560-7917.ES.2022.27.45.2200125

Stefanizzi, P., Bianchi, F. P., Ascatigno, L., Pantaleo, N., Martinelli, A., Di Lorenzo, A., Notarnicola, A., Fischetti, F., & Tafuri, S. (2023). Incidence of SARS-COV-2 infection among swimming athletes : Data from real life in Apulia (Italy), July 2020/August 2021. Annali Di Igiene: Medicina Preventiva E Di Comunita, 35(1). https://doi.org/10.7416/ai.2022.2520

Steffen, A., Rieck, T., & Siedler, A. (2021). Monitoring of Influenza Vaccination Coverage among Pregnant Women in Germany Based on Nationwide Outpatient Claims Data : Findings for Seasons 2014/15 to 2019/20. Vaccines, 9(5). https://doi.org/10.3390/vaccines9050485

Stella, C., Berardi, C., Chiarito, A., Gennenzi, V., Postorino, S., Settanni, D., Cesarano, M., Xhemalaj, R., Tanzarella, E. S., Cutuli, S. L., Grieco, D. L., Conti, G., Antonelli, M., & De Pascale, G. (2023). Clinical features and 28-day mortality predictors of vaccinated patients admitted to a COVID-19 ICU hub in Italy. Journal of Anesthesia, Analgesia and Critical Care, 3(1). https://doi.org/10.1186/s44158-023-00130-6

Stemler, J., Salmanton-García, J., Weise, B., Többen, C., Joisten, C., Fleig, J., & Cornely, O. A. (2022). A pilot surveillance report of SARS-CoV-2 rapid antigen test results among volunteers in Germany, 1st week of July 2022. medRxiv, (Stemler J., Jannik.Stemler@uk-koeln.de; Salmanton-García J.; Weise B.; Többen C.; Joisten C.; Fleig J.; Cornely O.A.) University of Cologne, Faculty of Medicine, University Hospital Cologne, Translational Research, Cologne Excellence Cluster on Cellular Stress Responses in Aging-Associated Diseases (CECAD), Herderstrasse 52, Cologne, Germany. https://doi.org/10.1101/2022.07.18.22277744

Stephens, N., Béen, F., & Savic, D. (2022). An Analysis of SARS-CoV-2 in Wastewater to Evaluate the Effectiveness of Nonpharmaceutical Interventions against COVID-19 in The Netherlands. ACS ES&T Water, 2(11). https://doi.org/10.1021/acsestwater.2c00071

Stępień, E., Koleśnik, M., Mitura, K., Malm, M., Drop, B., Jędrych, M., & Polz-Dacewicz, M. (2021). SARS-CoV-2 Infection Prevalence in the Population of South-Eastern Poland. Diagnostics (Basel, Switzerland), 11(11). https://doi.org/10.3390/diagnostics11112115

Steul, K., Heudorf, U., Uphoff, H., & Kowall, B. (2023). Excess mortality during the SARS-CoV-2 pandemic in the City of Frankfurt/Main, Germany, in 2020 and 2021, adjusted for age trends and pandemic phases. GMS Hygiene and Infection Control, 18, Doc08. https://doi.org/10.3205/dgkh000434

Stoliaroff-Pepin, A., Peine, C., Herath, T., Lachmann, J., Hellenbrand, W., Perriat, D., Dörre, A., Nitsche, A., Michel, J., Grossegesse, M., Hofmann, N., Rinner, T., Kohl, C., Brinkmann, A., Meyer, T., Stern, D., Treindl, F., Dorner, B. G., Hein, S., … Harder, T. (2023). Vaccine effectiveness against severe COVID-19 during the Omicron wave in Germany : Results from the COViK study. Infection, 51(4). https://doi.org/10.1007/s15010-023-02012-z

Stoliaroff-Pepin, A., Peine, C., Herath, T., Lachmann, J., Perriat, D., Dörre, A., Nitsche, A., Michel, J., Grossegesse, M., Hofmann, N., Rinner, T., Kohl, C., Brinkmann, A., Meyer, T., Dorner, B. G., Stern, D.,

Treindl, F., Hein, S., Werel, L., … Harder, T. (2023). Effectiveness of vaccines in preventing hospitalization due to COVID-19 : A multicenter hospital-based case-control study, Germany, June 2021 to January 2022. Vaccine, 41(2). https://doi.org/10.1016/j.vaccine.2022.11.065

Stouten, V., Hubin, P., Haarhuis, F., van Loenhout, J. A. F., Billuart, M., Brondeel, R., Braeye, T., Van Oyen, H., Wyndham-Thomas, C., & Catteau, L. (2022). Incidence and Risk Factors of COVID-19 Vaccine Breakthrough Infections : A Prospective Cohort Study in Belgium. Viruses, 14(4). https://doi.org/10.3390/v14040802

Stouten, V., Van Evercooren, I., Vernemmen, C., Braeye, T., Catteau, L., Roelants, M., Billuart, M., Lamot, T., Sierra, N. B., Hammami, N., Vermeiren, E., Rosas, A., Blot, K., Schmelz, A. I., Nasiadka, L., Nganda, S., & van Loenhout, J. A. F. (2025). Averted mortality by COVID-19 vaccination in Belgium between 2021 and 2023. Vaccine, 60, 127290. https://doi.org/10.1016/j.vaccine.2025.127290

Strålin, K., Wahlström, E., Walther, S., Bennet-Bark, A. M., Heurgren, M., Lindén, T., Holm, J., & Hanberger, H. (2021). Mortality trends among hospitalised COVID-19 patients in Sweden : A nationwide observational cohort study. The Lancet Regional Health. Europe, 4. https://doi.org/10.1016/j.lanepe.2021.100054

Strålin, K., Wahlström, E., Walther, S., Bennet-Bark, A. M., Heurgren, M., Lindén, T., Holm, J., & Hanberger, H. (2022). Mortality in hospitalized COVID-19 patients was associated with the COVID-19 admission rate during the first year of the pandemic in Sweden. Infectious Diseases (London, England), 54(2), 145–151. https://doi.org/10.1080/23744235.2021.1983643

Stremel, T., Schnaidt, S., Bihrer, N., Fröling, E., Jacob, C., & Kisser, A. (2025). A Retrospective Claims Data Analysis on the Burden of COVID-19-Related Hospitalization in Adults at High Risk for Severe Disease Progression in Germany. Infectious Diseases and Therapy, 14(1). https://doi.org/10.1007/s40121-024-01088-w

Streng, A., Goettler, D., Haerlein, M., Lehmann, L., Ulrich, K., Prifert, C., Krempl, C., Weißbrich, B., & Liese, J. G. (2019). Spread and clinical severity of respiratory syncytial virus A genotype ON1 in Germany, 2011-2017. BMC Infectious Diseases, 19(1). https://doi.org/10.1186/s12879-019-4266-y

Streng, A., Prifert, C., Weissbrich, B., Liese, J. G., Bösel, N., Gašpirová, P., Henrich, K., Hösl, A., Klein, M., Schnelke, A., Wieg, C., Steinherr, H., Schreiner, H.-G., Horsinka, S., Wolf, A., Engelke, Pohl, W., Bohlein, B., Egler, K., … Grombach, J. (2015). Continued high incidence of children with severe influenza A(H1N1)pdm09 admitted to paediatric intensive care units in Germany during the first three post-pandemic influenza seasons, 2010/11-2012/13. BMC Infectious Diseases, 15(1). https://doi.org/10.1186/s12879-015-1293-1

Streng, A., Prifert, C., Weissbrich, B., Sauerbrei, A., Schmidt-Ott, R., & Liese, J. G. (2018). Subtype-specific Clinical Presentation, Medical Treatment and Family Impact of Influenza in Children 1-5 Years of Age Treated in Outpatient Practices in Germany During Three Postpandemic Years, 2013-2015. The Pediatric Infectious Disease Journal, 37(9). https://doi.org/10.1097/INF.0000000000001935

Stroffolini, T., Ciancio, A., Federico, A., Benigno, R. G., Colloredo, G., Lombardi, A., Niro, G. A., Verucchi, G., Ferrigno, L., Gioli, F., & Marignani, M. (2023). COVID-19 vaccination among cirrhotics in Italy : High coverage and effectiveness of 3 doses versus 2 in preventing breakthrough infection and hospitalization. Digestive and Liver Disease: Official Journal of the Italian Society of Gastroenterology and the Italian Association for the Study of the Liver, 55(3). https://doi.org/10.1016/j.dld.2022.11.016

Strollo, R., Maddaloni, E., Dauriz, M., Pedone, C., Buzzetti, R., & Pozzilli, P. (2021). Use of DPP4 inhibitors in Italy does not correlate with diabetes prevalence among COVID-19 deaths. Diabetes Research and Clinical Practice, 171. https://doi.org/10.1016/j.diabres.2020.108444

Strozza, C., Vigezzi, S., Callaway, J., & Aburto, J. M. (2024). The impact of COVID-19 on life expectancy across socioeconomic groups in Denmark. Population Health Metrics, 22(1). https://doi.org/10.1186/s12963-024-00323-3

Strzelecki, A., Azevedo, A., & Albuquerque, A. (2020). Correlation between the Spread of COVID-19 and the Interest in Personal Protective Measures in Poland and Portugal. Healthcare (Basel, Switzerland), 8(3). https://doi.org/10.3390/healthcare8030203

Strzelecki, A., Azevedo, A., Rizun, M., Rutecka, P., Zagała, K., Cicha, K., & Albuquerque, A. (2022). Human Mobility Restrictions and COVID-19 Infection Rates : Analysis of Mobility Data and Coronavirus Spread in Poland and Portugal. International Journal of Environmental Research and Public Health, 19(21). https://doi.org/10.3390/ijerph192114455

Stufano, A., Buonvino, N., Trombetta, C. M., Pontrelli, D., Marchi, S., Lobefaro, G., De Benedictis, L., Lorusso, E., Carofiglio, M. T., Vasinioti, V. I., Montomoli, E., Decaro, N., & Lovreglio, P. (2022). COVID-19 Outbreak and BNT162b2 mRNA Vaccination Coverage in a Correctional Facility during Circulation of the SARS-CoV-2 Omicron BA.1 Variant in Italy. Vaccines, 10(7). https://doi.org/10.3390/vaccines10071137

Stüven, P., Mühlenbruch, G., Evenschor-Ascheid, A., Conzen, E., Peters, C., Schablon, A., & Nienhaus, A. (2022). COVID-19 infections in staff of an emergency care hospital after the first wave of the pandemic in Germany. GMS Hygiene and Infection Control, 17, Doc04. https://doi.org/10.3205/dgkh000407

Suarez Castillo, M., Khaoua, H., & Courtejoie, N. (2022). Vaccine effectiveness and duration of protection against symptomatic infections and severe Covid-19 outcomes in adults aged 50 years and over, France, January to mid-December 2021. Global Epidemiology, 4. https://doi.org/10.1016/j.gloepi.2022.100076

Suárez-García, I., Martínez de Aramayona López, M. J., Sáez Vicente, A., & Lobo Abascal, P. (2020). SARS-CoV-2 infection among healthcare workers in a hospital in Madrid, Spain. The Journal of Hospital Infection, 106(2). https://doi.org/10.1016/j.jhin.2020.07.020

Suárez-García, I., Perales-Fraile, I., González-García, A., Muñoz-Blanco, A., Manzano, L., Fabregate, M., Díez-Manglano, J., Aizpuru, E. F., Fernández, F. A., García, A. G., Gómez-Huelgas, R., Ramos-Rincón, J.-M., & SEMI-COVID-19 Network. (2021). In-hospital mortality among immunosuppressed patients with COVID-19 : Analysis from a national cohort in Spain. PloS One, 16(8). https://doi.org/10.1371/journal.pone.0255524

Suárez-Varela, M. M., Llopis, A., Fernandez-Fabrellas, E., Sanz, F., Perez-Lozano, M. J., Martin, V., Astray, J., Castilla, J., Egurrola, M., Force, L., Toledo, D., Domínguez, À., & Working group of project pi12/02079 'effectiveness of influenza and pneumococcal polysaccharide 23-valent vaccines in people aged 65 years and more'. (2018). Asthma and influenza vaccination in elderly hospitalized patients : Matched case-control study in Spain. The Journal of Asthma: Official Journal of the Association for the Care of Asthma, 55(4). https://doi.org/10.1080/02770903.2017.1332204

Subissi, L., Bossuyt, N., Reynders, M., Gérard, M., Dauby, N., Bourgeois, M., Delaere, B., Quoilin, S., Van Gucht, S., Thomas, I., & Barbezange, C. (2020). Capturing respiratory syncytial virus season in Belgium using the influenza severe acute respiratory infection surveillance network, season 2018/19. Euro Surveillance: Bulletin Europeen Sur Les Maladies Transmissibles = European Communicable Disease Bulletin, 25(39). https://doi.org/10.2807/1560-7917.ES.2020.25.39.1900627

Subissi, L., Bossuyt, N., Reynders, M., Gérard, M., Dauby, N., Lacor, P., Daelemans, S., Lissoir, B., Holemans, X., Magerman, K., Jouck, D., Bourgeois, M., Delaere, B., Quoilin, S., Van Gucht, S., Thomas, I., & Barbezange, C. (2021). Spotlight influenza : Extending influenza surveillance to detect non-influenza respiratory viruses of public health relevance : Analysis of surveillance data, Belgium, 2015 to 2019. Euro Surveillance: Bulletin Europeen Sur Les Maladies Transmissibles = European Communicable Disease Bulletin, 26(38). https://doi.org/10.2807/1560-7917.ES.2021.26.38.2001104

Summanen, M., Kosunen, M., Kainu, V., Cansel, A., Niskanen, S., Nurmi, L., Leskelä, R.-L., & Isomeri, O. (2023). COVID-19 hospitalisations and all-cause mortality by risk group in Finland. PloS One, 18(5). https://doi.org/10.1371/journal.pone.0286142

Sun, Y., Hu, X., & Xie, J. (2021). Spatial inequalities of COVID-19 mortality rate in relation to socioeconomic and environmental factors across England. The Science of the Total Environment, 758. https://doi.org/10.1016/j.scitotenv.2020.143595

Suñer, C., Coma, E., Ouchi, D., Hermosilla, E., Baro, B., Rodríguez-Arias, M. À., Puig, J., Clotet, B., Medina, M., & Mitjà, O. (2022). Association between two mass-gathering outdoor events and incidence of SARS-CoV-2 infections during the fifth wave of COVID-19 in north-east Spain : A population-based control-matched analysis. The Lancet Regional Health. Europe, 15. https://doi.org/10.1016/j.lanepe.2022.100337

Suñer, C., Ouchi, D., Mas, M., Alarcon, R., Mesquida, M., Prat, N., Bonet-Simó, J., Izquierdo, M., Sánchez, I., Noguerola, S., Colet, M., Puigvendrelló, J., Henríquez, N., Miralles, R., Negredo, E., Noguera-Julian, M., Marks, M., Estrada, O., Ara, J., & Mitjà, O. (2021). A retrospective cohort study of risk factors for mortality among nursing homes exposed to COVID-19 in Spain. NATURE AGING, 1(7). https://doi.org/10.1038/s43587-021-00079-7

Suppressa, P., Pagella, F., Lenato, G. M., Gaetani, E., Serio, I., Masala, M. S., Spinozzi, G., Lizzio, R., Matti, E., De Silvestri, A., Passali, G. C., Aguglia, M., Crocione, C., & Sabbà, C. (2021). Characterization of epidemiological distribution and outcome of COVID-19 in patients with hereditary hemorrhagic telangiectasia : A nationwide retrospective multi-centre study during first wave in Italy. Orphanet Journal of Rare Diseases, 16(1). https://doi.org/10.1186/s13023-021-02000-2

Surveillance Group for New Influenza A(H1N1) Virus Investigation in Italy. (2009). Virological surveillance of human cases of influenza A(H1N1)v virus in Italy : Preliminary results. Euro Surveillance: Bulletin Europeen Sur Les Maladies Transmissibles = European Communicable Disease Bulletin, 14(24). https://doi.org/10.2807/ese.14.24.19247-en

Suski, P., Jörres, R. A., Engelhardt, S., Kahnert, K., Lenherr, K., Bauer, A., & Budweiser, S. (2024). Period of hospitalization and mortality in transferred versus non-transferred COVID-19 patients : Results from Germany. Scientific Reports, 14(1). https://doi.org/10.1038/s41598-024-57272-y

Susło, R., Pobrotyn, P., Brydak, L., Rypicz, Ł., Grata-Borkowska, U., & Drobnik, J. (2021). Seasonal Influenza and Low Flu Vaccination Coverage as Important Factors Modifying the Costs and Availability of Hospital Services in Poland : A Retrospective Comparative Study. International Journal of Environmental Research and Public Health, 18(10). https://doi.org/10.3390/ijerph18105173

Susło, R., Pobrotyn, P., Mierzecki, A., & Drobnik, J. (2022). Fear of Illness and Convenient Access to Vaccines Appear to Be the Missing Keys to Successful Vaccination Campaigns : Analysis of the Factors Influencing the Decisions of Hospital Staff in Poland concerning Vaccination against Influenza and COVID-19. Vaccines, 10(7). https://doi.org/10.3390/vaccines10071026

Sutton, J., Shahtahmassebi, G., Ribeiro, H., & Hanley, Q. (2022). Population density and spreading of COVID-19 in England and Wales. PLOS ONE, 17(3). https://doi.org/10.1371/journal.pone.0261725

Suwono, B., Steffen, A., Schweickert, B., Schönfeld, V., Brandl, M., Sandfort, M., Willrich, N., Eckmanns, T., & Haller, S. (2022). SARS-CoV-2 outbreaks in hospitals and long-term care facilities in Germany : A national observational study. The Lancet Regional Health. Europe, 14. https://doi.org/10.1016/j.lanepe.2021.100303

Svallfors, S., Larsson, E. C., Puranen, B., & Ekström, A. M. (2023). COVID-19 vaccine hesitancy among first-generation immigrants living in Sweden. European Journal of Public Health, 33(4). https://doi.org/10.1093/eurpub/ckad073

Symes, R., Keddie, S. H., Walker, J., McKeever, T., Ahmad, S., Arnold, D., Evans, C. M., Pelosi, E., Rahman, N. M., Sapey, E., Zambon, M., Watson, C., Bernal, J. L., Lim, W. S., & HARISS network collaborators. (2025). Burden of respiratory syncytial virus infection in older adults hospitalised in England during 2023/24. The Journal of Infection, 91(3), 106570. https://doi.org/10.1016/j.jinf.2025.106570

Symes, R., Whitaker, H. J., Ahmad, S., Arnold, D., Banerjee, S., Evans, C. M., Gore, R., Hart, J., Heaney, K., Kon, O. M., Melhuish, A., Zogaib, M. O., Pelosi, E., Rahman, N., Woltmann, G., McKeever, T., Zambon, M., Watson, C. H., Lim, W. S., … Cobbold, A. (2025). Vaccine effectiveness of a bivalent respiratory syncytial virus (RSV) pre-F vaccine against RSV-associated hospitalisation among adults aged 75-79 years in England. medRxiv. https://doi.org/10.1101/2025.06.13.25329583

Syrjänen, R. K., Jokinen, J., Ziegler, T., Sundman, J., Lahdenkari, M., Julkunen, I., & Kilpi, T. M. (2014). Effectiveness of pandemic and seasonal influenza vaccines in preventing laboratory-confirmed influenza in Adults: A clinical cohort study during epidemic seasons 2009-2010 and 2010-2011 in Finland. PLoS ONE, 9(9). https://doi.org/10.1371/journal.pone.0108538

Szymański, K., Hallmann, E., Łuniewska, K., Kondratiuk, K., Poznańska, A., & Brydak, L. B. (2021). Spread of Influenza Viruses in Poland and Neighboring Countries in Seasonal Terms. Pathogens (Basel, Switzerland), 10(3). https://doi.org/10.3390/pathogens10030316

Tabatabai, J., Ihling, C. M., Rehbein, R. M., Schnee, S. V., Hoos, J., Pfeil, J., Grulich-Henn, J., & Schnitzler, P. (2022). Molecular epidemiology of respiratory syncytial virus in hospitalised children in Heidelberg, Southern Germany, 2014-2017. Infection, Genetics and Evolution: Journal of Molecular Epidemiology and Evolutionary Genetics in Infectious Diseases, 98. https://doi.org/10.1016/j.meegid.2022.105209

Taccone, F. S., Van Goethem, N., De Pauw, R., Wittebole, X., Blot, K., Van Oyen, H., Lernout, T., Montourcy, M., Meyfroidt, G., Van Beckhoven, D., & Belgian Society of Intensive Care Medicine and the Belgian Collaborative Group on COVID-19 Hospital Surveillance. (2021). The role of organizational characteristics on the outcome of COVID-19 patients admitted to the ICU in Belgium. The Lancet Regional Health. Europe, 2. https://doi.org/10.1016/j.lanepe.2020.100019

Taillé, C., Roche, N., Tesson, F., Tardivon, C., Tran, V.-T., & Couffignal, C. (2022). Belief and adherence to COVID 19-lockdown restrictions in patients with asthma versus other chronic diseases : Results from a cross-sectional survey nested in the ComPaRe e-cohort, in France. The Journal of Asthma: Official Journal of the Association for the Care of Asthma, 59(8). https://doi.org/10.1080/02770903.2021.1941091

Tamandjou Tchuem, C. R., Auvigne, V., Vaux, S., Montagnat, C., Paireau, J., Monnier Besnard, S., Gabet, A., Benhajkassen, N., Le Strat, Y., Parent Du Chatelet, I., & Levy-Bruhl, D. (2023). Vaccine effectiveness and duration of protection of COVID-19 mRNA vaccines against Delta and Omicron BA.1 symptomatic and severe COVID-19 outcomes in adults aged 50 years and over in France. Vaccine, 41(13). https://doi.org/10.1016/j.vaccine.2023.02.062

Tamandjou, C., Auvigne, V., Schaeffer, J., Vaux, S., & Parent du Châtelet, I. (2023). Effectiveness of second booster compared to first booster and protection conferred by previous SARS-CoV-2 infection against symptomatic Omicron BA.2 and BA.4/5 in France. Vaccine, 41(17). https://doi.org/10.1016/j.vaccine.2023.03.031

Tammes, P. (2020). Social distancing, population density, and spread of COVID-19 in England : A longitudinal study. BJGP Open, 4(3). https://doi.org/10.3399/bjgpopen20X101116

Tan, P. S., Patone, M., Clift, A. K., Dambha-Miller, H., Saatci, D., Ranger, T. A., Garriga, C., Zaccardi, F., Shah, B. R., Coupland, C., Griffin, S. J., Khunti, K., & Hippisley-Cox, J. (2023). Factors influencing influenza, pneumococcal and shingles vaccine uptake and refusal in older adults : A population-based cross-sectional study in England. BMJ Open, 13(3). https://doi.org/10.1136/bmjopen-2021-058705

Tapia, J. C., Gavira, J., López, A., Llobera, L., Pallise, O., Marsal, I., Cochs, A., Ponce, O. J., Riudavets, M., Gich, I., Barnadas, A., & Majem, M. (2022). Ninety-day mortality and clinical outcomes of patients with solid tumours and COVID-19 infection during the first pandemic outbreak in Catalonia, Spain: A multicentre retrospective study. International Journal of Cancer, 150(8), 1310–1317. https://doi.org/10.1002/ijc.33909

Tarantola, A., & Hamidouche, M. (2025). Use of cell phone data to correct Île-de-France population estimates and SARS-CoV-2 incidence, July to September, 2021: A proof-of-concept exercise. Euro Surveillance: Bulletin Europeen Sur Les Maladies Transmissibles = European Communicable Disease Bulletin, 30(22), 2400530. https://doi.org/10.2807/1560-7917.ES.2025.30.22.2400530

Tateo, F., Fiorino, S., Peruzzo, L., Zippi, M., De Biase, D., Lari, F., & Melucci, D. (2022). Effects of environmental parameters and their interactions on the spreading of SARS-CoV-2 in North Italy under different social restrictions. A new approach based on multivariate analysis. Environmental Research, 210. https://doi.org/10.1016/j.envres.2022.112921

Tavolacci, M. P., Dechelotte, P., & Ladner, J. (2021). COVID-19 Vaccine Acceptance, Hesitancy, and Resistancy among University Students in France. Vaccines, 9(6). https://doi.org/10.3390/vaccines9060654

Taxbro, K., Granath, A., Sunnergren, O., Seifert, S., Jakubczyk, M. N., Persson, M., Hammarskjöld, A., Alkemark, C., Hammarskjöld, F., & RJL COVID-19 Research Group. (2021). Low mortality rates among

critically ill adults with COVID-19 at three non-academic intensive care units in south Sweden. Acta Anaesthesiologica Scandinavica, 65(10). https://doi.org/10.1111/aas.13972

Tchicaya, A., Lorentz, N., Leduc, K., & de Lanchy, G. (2021). COVID-19 mortality with regard to healthcare services availability, health risks, and socio-spatial factors at department level in France : A spatial cross-sectional analysis. PloS One, 16(9). https://doi.org/10.1371/journal.pone.0256857

Te Beest, D. E., Birrell, P. J., Wallinga, J., De Angelis, D., & Van Boven, M. (2015). Joint modelling of serological and hospitalization data reveals that high levels of pre-existing immunity and school holidays shaped the influenza A pandemic of 2009 in The Netherlands. Journal of the Royal Society Interface, 12(103). https://doi.org/10.1098/rsif.2014.1244

Tedeschi, S., Badia, L., Berveglieri, F., Ferrari, R., Coladonato, S., Gabrielli, S., Maestri, A., Peroni, G., Giannella, M., Rossi, A., Viale, P., & COVID-19 Fighting Team. (2021). Effective Containment of a COVID-19 Subregional Outbreak in Italy Through Strict Quarantine and Rearrangement of Local Health Care Services. Open Forum Infectious Diseases, 8(2). https://doi.org/10.1093/ofid/ofab024

Teirlinck, A. C., de Gier, B., Meijer, A., Donker, G., de Lange, M., Koppeschaar, C., van der Hoek, W., Kretzschmar, M. E., & McDonald, S. A. (2018). The incidence of symptomatic infection with influenza virus in the Netherlands 2011/2012 through 2016/2017, estimated using Bayesian evidence synthesis. Epidemiology and Infection, 147. https://doi.org/10.1017/S095026881800273X

Tenenbaum, T., Doenhardt, M., Diffloth, N., Berner, R., & Armann, J. P. (2022). High burden of RSV hospitalizations in Germany 2021-2022. Infection, 50(6). https://doi.org/10.1007/s15010-022-01889-6

Terlecki, M., Wojciechowska, W., Klocek, M., Drożdż, T., Kocowska-Trytko, M., Lis, P., Pavlinec, C., Pęksa, J. W., Kania, M., Siudak, Z., Januszewicz, A., Kreutz, R., Małecki, M., Grodzicki, T., Rajzer, M., & CraCov HHS Investigators. (2023). Prevalence and clinical implications of atrial fibrillation in patients hospitalized due to COVID-19 : Data from a registry in Poland. Frontiers in Cardiovascular Medicine, 10. https://doi.org/10.3389/fcvm.2023.1133373

Terlecki, M., Wojciechowska, W., Klocek, M., Olszanecka, A., Stolarz-Skrzypek, K., Grodzicki, T., Małecki, M., Katra, B., Garlicki, A., Bociąga-Jasik, M., Sładek, K., Matyja, A., Wordliczek, J., Słowik, A., Mach, T., Krzanowska, K., Krzanowski, M., Stręk, P., Chłosta, P., … Rajzer, M. (2021). Association between cardiovascular disease, cardiovascular drug therapy, and in-hospital outcomes in patients with COVID-19 : Data from a large single-center registry in Poland. Kardiologia Polska, 79(7-8). https://doi.org/10.33963/KP.15990

Tessier, E., Edelstein, M., Tsang, C., Kirsebom, F., Gower, C., Campbell, C. N. J., Ramsay, M., White, J., Andrews, N., Lopez-Bernal, J., & Stowe, J. (2023). Monitoring the COVID-19 immunisation programme through a national immunisation Management system—England's experience. International Journal of Medical Informatics, 170. https://doi.org/10.1016/j.ijmedinf.2022.104974

Tessier, E., Rai, Y., Clarke, E., Lakhani, A., Tsang, C., Makwana, A., Heard, H., Rickeard, T., Lakhani, S., Roy, P., Edelstein, M., Ramsay, M., Lopez-Bernal, J., White, J., Andrews, N., Campbell, C. N. J., & Stowe, J. (2022). Characteristics associated with COVID-19 vaccine uptake among adults aged 50 years and above in England (8 December 2020-17 May 2021) : A population-level observational study. BMJ Open, 12(3). https://doi.org/10.1136/bmjopen-2021-055278

Tessier, E., Warburton, F., Tsang, C., Rafeeq, S., Boddington, N., Sinnathamby, M., & Pebody, R. (2018). Population-level factors predicting variation in influenza vaccine uptake among adults and young children in England, 2015/16 and 2016/17. Vaccine, 36(23). https://doi.org/10.1016/j.vaccine.2018.04.074

Thi Khanh, H. N., De Troeyer, K., Smith, P., Demoury, C., & Casas, L. (2024). The impact of ambient temperature and air pollution on SARS-CoV2 infection and Post COVID-19 condition in Belgium (2021-2022). Environmental Research, 246. https://doi.org/10.1016/j.envres.2023.118066

Thiam, M.-M., Pontais, I., Forgeot, C., Pedrono, G., SurSaUD® Regional Focal Point, SOS Médecins, Group of Emergency Medicine Doctors, Paget, L.-M., Fouillet, A., & Caserio-Schönemann, C. (2022). Syndromic surveillance : A key component of population health monitoring during the first wave of the COVID-19 outbreak in France, February-June 2020. PloS One, 17(2). https://doi.org/10.1371/journal.pone.0260150

Thiberville, S.-D., Gaudart, J., Raoult, D., & Charrel, R. N. (2015). Influenza-attributable deaths in south-eastern France (1999 to 2010) : Mortality predictions were undependable. BMC Public Health, 15. https://doi.org/10.1186/s12889-015-1887-y

Thibon, P., Grenier, C., Erouart, S., Borgey, F., Le Hello, S., & Martel, M. (2023). Evolution of the incidence of COVID-19 during the first five waves in residents and professionals of nursing homes in Normandy, France. Aging Clinical and Experimental Research, 35(4). https://doi.org/10.1007/s40520-023-02375-1

Thomas, H. L., Andrews, N., Green, H. K., Boddington, N. L., Zhao, H., Reynolds, A., McMenamin, J., & Pebody, R. G. (2014). Estimating vaccine effectiveness against severe influenza in England and Scotland 2011/2012 : Applying the screening method to data from intensive care surveillance systems. Epidemiology and Infection, 142(1). https://doi.org/10.1017/S0950268813000824

Thompson, J. V., Meghani, N. J., Powell, B. M., Newell, I., Craven, R., Skilton, G., Bagg, L. J., Yaqoob, I., Dixon, M. J., Evans, E. J., Kambele, B., Rehman, A., & Ng Man Kwong, G. (2020). Patient characteristics and predictors of mortality in 470 adults admitted to a district general hospital in England with Covid-19. Epidemiology and Infection, 148. https://doi.org/10.1017/S0950268820002873

Thomsen, R. W., Christiansen, C. F., Heide-Jørgensen, U., Vogelstein, J. T., Vogelstein, B., Bettegowda, C., Tamang, S., Athey, S., & Sørensen, H. T. (2021). Association of α1-Blocker Receipt With 30-Day Mortality and Risk of Intensive Care Unit Admission Among Adults Hospitalized With Influenza or Pneumonia in Denmark. JAMA Network Open, 4(2). https://doi.org/10.1001/jamanetworkopen.2020.37053

Thorrington, D., Balasegaram, S., Cleary, P., Hay, C., & Eames, K. (2017). Social and Economic Impacts of School Influenza Outbreaks in England : Survey of Caregivers. The Journal of School Health, 87(3). https://doi.org/10.1111/josh.12484

Thorrington, D., van Leeuwen, E., Ramsay, M., Pebody, R., & Baguelin, M. (2017). Cost-effectiveness analysis of quadrivalent seasonal influenza vaccines in England. BMC Medicine, 15(1). https://doi.org/10.1186/s12916-017-0932-3

Ticinesi, A., Parise, A., Cerundolo, N., Nouvenne, A., Prati, B., Chiussi, G., Guerra, A., & Meschi, T. (2022). Multimorbidity and Frailty Are the Key Characteristics of Patients Hospitalized with COVID-19 Breakthrough Infection during Delta Variant Predominance in Italy : A Retrospective Study. Journal of Clinical Medicine, 11(18). https://doi.org/10.3390/jcm11185442

Tillard, C., Chazard, E., Faure, K., Bartolo, S., Martinot, A., & Dubos, F. (2022). Burden of influenza disease in children under 2 years of age hospitalized between 2011 and 2020 in France. The Journal of Infection, 84(2). https://doi.org/10.1016/j.jinf.2021.11.006

Tilmanne, A., Pirson, M., Leclercq, P., Van Den Bulcke, J., & Bruyneel, A. (2024). Evaluation of the costs of care for pediatric patients hospitalized for RSV: a retrospective cohort study in Belgium. medRxiv, (Tilmanne A.) Infectious diseases and Infection Prevention and Control Department, CHU Tivoli, La Louviere, Belgium. https://doi.org/10.1101/2024.11.21.24317618

Tilmanne, A., Pirson, M., Leclercq, P., Van Den Bulcke, J., Dauvergne, J. E., & Bruyneel, A. (2025). Evaluation of the costs of care for pediatric patients hospitalized for RSV: A retrospective cohort study in Belgium. Vaccine, 55, 127065. https://doi.org/10.1016/j.vaccine.2025.127065

Timelli, L., & Girardi, E. (2021). Effect of timing of implementation of containment measures on Covid-19 epidemic. The case of the first wave in Italy. PloS One, 16(1). https://doi.org/10.1371/journal.pone.0245656

Timmers, T., Janssen, L., Stohr, J., Murk, J. L., & Berrevoets, M. a. H. (2020). Using eHealth to Support COVID-19 Education, Self-Assessment, and Symptom Monitoring in the Netherlands : Observational Study. JMIR mHealth and uHealth, 8(6). https://doi.org/10.2196/19822

Timpka, T., Eriksson, O., Spreco, A., Gursky, E., Strömgren, M., Holm, E., Ekberg, J., Dahlström, Ö., Valter, L., & Eriksson, H. (2012). Age as a Determinant for Dissemination of Seasonal and Pandemic Influenza: An Open Cohort Study of Influenza Outbreaks in Ostergotland County, Sweden. PLOS ONE, 7(2). https://doi.org/10.1371/journal.pone.0031746

Timpka, T., Spreco, A., Eriksson, O., Dahlström, Ö., Gursky, E. A., Strömgren, M., Holm, E., Ekberg, J., Hinkula, J., Nyce, J. M., & Eriksson, H. (2014). Predictive performance of telenursing complaints in influenza surveillance : A prospective cohort study in Sweden. Euro Surveillance: Bulletin Europeen Sur Les Maladies Transmissibles = European Communicable Disease Bulletin, 19(46). https://doi.org/10.2807/1560-7917.es2014.19.46.20966

Tinuoya, A., Allen, A., Rawlinson, C., Aziz, N. I. B. A., Cook, B., Woods, A., Dabrera, G., Evans, I., Willner, S., & Tessier, E. (2025). Evaluating COVID-19 cases reported across prisons in England from 2020 to 2023: Is enhanced address matching a game-changer for surveillance? Public Health, 244, 105748. https://doi.org/10.1016/j.puhe.2025.105748

Tiwari, A., Lipponen, A., Hokajärvi, A.-M., Luomala, O., Sarekoski, A., Rytkönen, A., Österlund, P., Al-Hello, H., Juutinen, A., Miettinen, I. T., Savolainen-Kopra, C., & Pitkänen, T. (2022). Detection and quantification of SARS-CoV-2 RNA in wastewater influent in relation to reported COVID-19 incidence in Finland. Water Research, 215. https://doi.org/10.1016/j.watres.2022.118220

Tizzani, M., De Gaetano, A., Jarvis, C. I., Gimma, A., Wong, K., Edmunds, W. J., Beutels, P., Hens, N., Coletti, P., & Paolotti, D. (2023). Impact of tiered measures on social contact and mixing patterns of in Italy during the second wave of COVID-19. BMC Public Health, 23(1). https://doi.org/10.1186/s12889-023-15846-x

Tobías, A., Molina, T., Rodrigo, M., & Saez, M. (2021). Meteorological factors and incidence of COVID-19 during the first wave of the pandemic in Catalonia (Spain): A multi-county study. One Health (Amsterdam, Netherlands), 12, 100239. https://doi.org/10.1016/j.onehlt.2021.100239

Toledo, D. M., Robbins, A. A., Gallagher, T. L., Hershberger, K. C., Barney, R. E., Salmela, S. M., Pilcher, D., Cervinski, M. A., Nerenz, R. D., Szczepiorkowski, Z. M., Tsongalis, G. J., Lefferts, J. A., Martin, I. W., & Hubbard, J. A. (2022). Wastewater-Based SARS-CoV-2 Surveillance in Northern New England. Microbiology Spectrum, 10(2). https://doi.org/10.1128/spectrum.02207-21

Tomaino, L., Pinilla, J., Rodríguez-Mireles, S., González López-Valcárcel, B., Barber-Pérez, P., Sierra, A., La Vecchia, C., & Serra-Majem, L. (2021). Impact of sandstorm and carnival celebrations on SARS-CoV-2 spreading in Tenerife and Gran Canaria (Canary Islands, Spain). Gaceta Sanitaria, 35(6). https://doi.org/10.1016/j.gaceta.2020.09.006

Tomas, J., Lelièvre, F., Bercelli, P., Glanddier, P.-Y., Fanello, S., Tuffreau, F., & Tallec, A. (2011). Hospital admissions related to influenza in France during the 2006/2007 epidemic. Revue D'epidemiologie Et De Sante Publique, 59(3). https://doi.org/10.1016/j.respe.2011.01.005

Tomasoni, D., Inciardi, R. M., Lombardi, C. M., Tedino, C., Agostoni, P., Ameri, P., Barbieri, L., Bellasi, A., Camporotondo, R., Canale, C., Carubelli, V., Carugo, S., Catagnano, F., Dalla Vecchia, L. A., Danzi, G. B., Di Pasquale, M., Gaudenzi, M., Giovinazzo, S., Gnecchi, M., … Metra, M. (2020). Impact of heart failure on the clinical course and outcomes of patients hospitalized for COVID-19. Results of the Cardio-COVID-Italy multicentre study. European Journal of Heart Failure, 22(12). https://doi.org/10.1002/ejhf.2052

Tomczyk, S., Hönning, A., Hermes, J., Grossegesse, M., Hofmann, N., Michel, J., Neumann, M., Nitsche, A., Hoppe, B., Eckmanns, T., Schmidt-Traub, H., & Zappel, K. (2022). Longitudinal SARS-CoV-2 seroepidemiological investigation among healthcare workers at a tertiary care hospital in Germany. BMC Infectious Diseases, 22(1). https://doi.org/10.1186/s12879-022-07057-3

Tomori, D., Rübsamen, N., Berger, T., Scholz, S., Walde, J., Wittenberg, I., Lange, B., Kuhlmann, A., Horn, J., Mikolajczyk, R., Jaeger, V., & Karch, A. (2021). Individual social contact data and population mobility data as early markers of SARS-CoV-2 transmission dynamics during the first wave in Germany-an analysis based on the COVIMOD study. BMC MEDICINE, 19(1). https://doi.org/10.1186/s12916-021-02139-6

Tondel, M., Nordquist, T., Helgesson, M., & Svartengren, M. (2024). COVID-19 : Incidence and mortality in Sweden comparing all foreign-born to all Swedish-born individuals in different occupations in an unvaccinated cohort of year 2020. Occupational and Environmental Medicine, 81(3). https://doi.org/10.1136/oemed-2023-108952

Tonne, C., Ranzani, O., Alari, A., Ballester, J., Basagaña, X., Chaccour, C., Dadvand, P., Duarte, T., Foraster, M., Milà, C., Nieuwenhuijsen, M. J., Olmos, S., Rico, A., Sunyer, J., Valentín, A., & Vivanco, R. (2024). Air Pollution in Relation to COVID-19 Morbidity and Mortality : A Large Population-Based Cohort Study in Catalonia, Spain (COVAIR-CAT). Research Report (Health Effects Institute), 2024(220). http://www.ncbi.nlm.nih.gov/pubmed/39468856

Tönshoff, B., Müller, B., Elling, R., Renk, H., Meissner, P., Hengel, H., Garbade, S. F., Kieser, M., Jeltsch, K., Grulich-Henn, J., Euler, J., Stich, M., Chobanyan-Jürgens, K., Zernickel, M., Janda, A., Wölfle, L., Stamminger, T., Iftner, T., Ganzenmueller, T., … Kräusslich, H.-G. (2021). Prevalence of SARS-CoV-2 Infection in Children and Their Parents in Southwest Germany. JAMA Pediatrics, 175(6). https://doi.org/10.1001/jamapediatrics.2021.0001

Tormos, R., Fonseca I Casas, P., & Garcia-Alamino, J. M. (2022). In-person school reopening and the spread of SARS-CoV-2 during the second wave in Spain. Frontiers in Public Health, 10. https://doi.org/10.3389/fpubh.2022.990277

Tornhammar, P., Jernberg, T., Bergström, G., Blomberg, A., Engström, G., Engvall, J., Fall, T., Gisslén, M., Janson, C., Lind, L., Sköld, C. M., Sundström, J., Söderberg, S., Zaigham, S., Östgren, C. J., Andersson, D. P., & Ueda, P. (2021). Association of cardiometabolic risk factors with hospitalisation or death due to COVID-19 : Population-based cohort study in Sweden (SCAPIS). BMJ Open, 11(9). https://doi.org/10.1136/bmjopen-2021-051359

Torti, C., Olimpieri, P. P., Bonfanti, P., Tascini, C., Celant, S., Tacconi, D., Nicastri, E., Tacconelli, E., Cacopardo, B., Perrella, A., Buccoliero, G. B., Parruti, G., Bassetti, M., Biagetti, C., Giacometti, A., Erne, E. M., Frontuto, M., Lanzafame, M., Summa, V., … Palù, G. (2023). Real-life comparison of mortality in patients with SARS-CoV-2 infection at risk for clinical progression treated with molnupiravir or nirmatrelvir plus ritonavir during the Omicron era in Italy : A nationwide, cohort study. The Lancet Regional Health. Europe, 31. https://doi.org/10.1016/j.lanepe.2023.100684

Toubiana, L., Mucchielli, L., Bouaud, J., & Chaillot, P. (2023). What the analysis of causes of death in France in 2020 reveals about the impact of the Covid-19 epidemic. medRxiv, (Toubiana L., laurent.toubiana@inserm.fr; Bouaud J.) Inserm, Sorbonne Université, Université Sorbonne Paris Nord, UMR S_1142, LIMICS, Paris, France. https://doi.org/10.1101/2023.03.07.23286673

Tozzi, A. E., Gesualdo, F., Urbani, E., Sbenaglia, A., Ascione, R., Procopio, N., Croci, I., & Rizzo, C. (2021). Digital Surveillance Through an Online Decision Support Tool for COVID-19 Over One Year of the Pandemic in Italy: Observational Study. Journal of Medical Internet Research, 23(8), e29556. https://doi.org/10.2196/29556

Traini, M. C., Caponi, C., Ferrari, R., & De Socio, G. V. (2020). A study of SARS-CoV-2 epidemiology in Italy : From early days to secondary effects after social distancing. Infectious Diseases (London, England), 52(12). https://doi.org/10.1080/23744235.2020.1797157

Tramuto, F., Restivo, V., Costantino, C., Colomba, G. M. E., Maida, C. M., Casuccio, A., & Vitale, F. (2019). Surveillance Data for Eight Consecutive Influenza Seasons in Sicily, Italy. The American Journal of Tropical Medicine and Hygiene, 101(6). https://doi.org/10.4269/ajtmh.19-0059

Traore, A., Charniga, K., Grellet, S., Terpant, G., Da Cruz, H., Lamy, A., Thomas, N., Gbaguidi, G., Mercier, A., Prudhomme, J., Visseaux, B., Vieillefond, V., Haim-Boukobza, S., Giannoli, J.-M., RELAB Study Group, Laboratory group, Castro-Alvarez, J., Kouamen, A.-C., Rameix-Welti, M.-A., … Members of the RELAB Study Group. (2025). Monitoring SARS-CoV-2 variants with complementary surveillance systems : Risk evaluation of the Omicron JN.1 variant in France, August 2023 to January 2024. Euro Surveillance: Bulletin Europeen Sur Les Maladies Transmissibles = European Communicable Disease Bulletin, 30(1). https://doi.org/10.2807/1560-7917.ES.2025.30.1.2400293

Travi, G., Rossotti, R., Merli, M., D'Amico, F., Chiappetta, S., Giussani, G., Panariello, A., Corradin, M., Vecchi, M., Raimondi, A., Baiguera, C., Nocita, B., Epis, O. M., Tarsia, P., Galbiati, F., Colombo, F., Fumagalli, R., Scaglione, F., Moreno, M., … Niguarda COVID-19 Group. (2021). Neurological manifestations in patients hospitalized with COVID-19 : A retrospective analysis from a large cohort in Northern Italy. The European Journal of Neuroscience, 53(8). https://doi.org/10.1111/ejn.15159

Trebbien, R., Fischer, T. K., Krause, T. G., Nielsen, L., Nielsen, X. C., Weinreich, L. S., Lis-Tønder, J., Skov, M. N., Christiansen, C. B., & Emborg, H.-D. (2017). Changes in genetically drifted H3N2 influenza A viruses and vaccine effectiveness in adults 65 years and older during the 2016/17 season in Denmark. Journal of Clinical Virology: The Official Publication of the Pan American Society for Clinical Virology, 94. https://doi.org/10.1016/j.jcv.2017.06.007

Treggiari, D., Piubelli, C., Formenti, F., Silva, R., & Perandin, F. (2022). Resurgence of Respiratory Virus after Relaxation of COVID-19 Containment Measures : A Real-World Data Study from a Regional Hospital of Italy. International Journal of Microbiology, 2022. https://doi.org/10.1155/2022/4915678

Treggiari, D., Pomari, C., Zavarise, G., Piubelli, C., Formenti, F., & Perandin, F. (2024). Characteristics of Respiratory Syncytial Virus Infections in Children in the Post-COVID Seasons : A Northern Italy Hospital Experience. Viruses, 16(1). https://doi.org/10.3390/v16010126

Triggiano, F., De Giglio, O., Apollonio, F., Brigida, S., Fasano, F., Mancini, P., Ferraro, G., Veneri, C., La Rosa, G., Suffredini, E., Lucentini, L., Ungaro, N., Di Vittorio, G., Mongelli, O., Albano, N., & Montagna, M. (2023). Wastewater-based Epidemiology and SARS-CoV-2 : Variant Trends in the Apulia Region (Southern Italy) and Effect of Some Environmental Parameters. FOOD AND ENVIRONMENTAL VIROLOGY, 15(4). https://doi.org/10.1007/s12560-023-09565-0

Trigo-Tasende, N., Vallejo, J., Rumbo-Feal, S., Conde-Perez, K., Nasser-Ali, M., Tarrio-Saavedra, J., Barbeito, I., Lamelo, F., Cao, R., Ladra, S., Bou, G., & Poza, M. (2023). Building-Scale Wastewater-Based Epidemiology for SARS-CoV-2 Surveillance at Nursing Homes in A Coruña, Spain. ENVIRONMENTS, 10(11). https://doi.org/10.3390/environments10110189

Trottier, J., Darques, R., Ait Mouheb, N., Partiot, E., Bakhache, W., Deffieu, M. S., & Gaudin, R. (2020). Post-lockdown detection of SARS-CoV-2 RNA in the wastewater of Montpellier, France. One Health (Amsterdam, Netherlands), 10. https://doi.org/10.1016/j.onehlt.2020.100157

Trucchi, C., Alicino, C., Orsi, A., Paganino, C., Barberis, I., Grammatico, F., Canepa, P., Rappazzo, E., Bruzzone, B., Sticchi, L., & Ansaldi, F. (2017). Fifteen years of epidemiologic, virologic and syndromic influenza surveillance : A focus on type B virus and the effects of vaccine mismatch in Liguria region, Italy. Human Vaccines & Immunotherapeutics, 13(2). https://doi.org/10.1080/21645515.2017.1264779

Trucchi, C., D'Amelio, M., Amicizia, D., Orsi, A., Loiacono, I., Tosatto, R., Piazza, M. F., Paganino, C., Pitrelli, A., Icardi, G., & Ansaldi, F. (2021). Lowering the recommended age for the free and active offer of influenza vaccination in Italy : Clinical and economic impact analysis in the Liguria region. Human Vaccines & Immunotherapeutics, 17(5). https://doi.org/10.1080/21645515.2020.1810494

Tsionas, M., Martins, M., & Heshmati, A. (2023). Effects of the vaccination and public support on covid-19 cases and number of deaths in Sweden. OPERATIONAL RESEARCH, 23(3). https://doi.org/10.1007/s12351-023-00794-6

Tunnicliffe, L., & Warren-Gash, C. (2022). Investigating the effects of population density of residence and rural/urban classification on rate of influenza-like illness symptoms in England and Wales. Influenza and Other Respiratory Viruses, 16(6). https://doi.org/10.1111/irv.13032

Tuppin, P., Choukroun, S., Samson, S., Weill, A., Ricordeau, P., & Allemand, H. (2012). [Vaccination against seasonal influenza in France in 2010 and 2011 : Decrease of coverage rates and associated factors]. Presse Medicale (Paris, France: 1983), 41(11). https://doi.org/10.1016/j.lpm.2012.05.017

Tuppin, P., Samson, S., Weill, A., Ricordeau, P., & Allemand, H. (2009). [Influenza vaccination coverage in France in 2007-2008 : Contribution of vaccination refund data from the general health insurance scheme]. Medecine Et Maladies Infectieuses, 39(10). https://doi.org/10.1016/j.medmal.2009.08.013

Tuppin, P., Samson, S., Weill, A., Ricordeau, P., & Allemand, H. (2011). Seasonal influenza vaccination coverage in France during two influenza seasons (2007 and 2008) and during a context of pandemic influenza A(H1N1) in 2009. Vaccine, 29(28), 4632–4637. https://doi.org/10.1016/j.vaccine.2011.04.064

Turbelin, C., Souty, C., Pelat, C., Hanslik, T., Sarazin, M., Blanchon, T., & Falchi, A. (2013). Age distribution of influenza like illness cases during post-pandemic A(H3N2): Comparison with the twelve previous seasons, in France. PloS One, 8(6), e65919. https://doi.org/10.1371/journal.pone.0065919

Turrini, M., Gardellini, A., Beretta, L., Buzzi, L., Ferrario, S., Vasile, S., Clerici, R., Colzani, A., Liparulo, L., Scognamiglio, G., Imperiali, G., Corrado, G., Strada, A., Galletti, M., Castiglione, N., & Zanon, C. (2021). Clinical Course and Risk Factors for In-Hospital Mortality of 205 Patients with SARS-CoV-2 Pneumonia in Como, Lombardy Region, Italy. Vaccines, 9(6). https://doi.org/10.3390/vaccines9060640

Turska-Kawa, A., & Pilch, I. (2022). Political beliefs and the acceptance of the SARS-CoV-2 pandemic restrictions. The case of Poland. PloS One, 17(3), e0264502. https://doi.org/10.1371/journal.pone.0264502

Twomey, E. P., Herman, D., Marín-Rodríguez, J. A., & Jimenez-Moleon, J. J. (2024). Influenza Vaccination Uptake and Associated Factors among Individuals with Diabetes Mellitus in Spain : A Cross-Sectional Study Using Data from the European Health Interview Survey 2020. Vaccines, 12(8). https://doi.org/10.3390/vaccines12080915

Tzialla, C., Marcellusi, A., Decembrino, L., Ghirardello, S., Licari, A., Marseglia, G., Tavella, E., & Manzoni, P. (2025). Estimated impact of nirsevimab prophylaxis on the economic burden of respiratory syncytial virus disease in Northern Italy. Italian Journal of Pediatrics, 51(1), 151. https://doi.org/10.1186/s13052-025-01991-z

Ughi, N., Bernasconi, D. P., Del Gaudio, F., Dicuonzo, A., Maloberti, A., Giannattasio, C., Tarsia, P., Puoti, M., Scaglione, F., Beltrami, L., Colombo, F., Bertuzzi, M., Bellone, A., Adinolfi, A., Valsecchi, M. G., Epis, O. M., Rossetti, C., & Niguarda COVID Working Group. (2022). Trends in all-cause mortality of hospitalized patients due to SARS-CoV-2 infection from a monocentric cohort in Milan (Lombardy, Italy). Zeitschrift Fur Gesundheitswissenschaften = Journal of Public Health, 30(8). https://doi.org/10.1007/s10389-021-01675-y

Ughi, N., Bernasconi, D. P., Gagliardi, C., Del Gaudio, F., Dicuonzo, A., Maloberti, A., Giannattasio, C., Rossetti, C., Valsecchi, M. G., & Epis, O. M. (2023). Trends in severe outcomes in SARS-CoV-2-positive hospitalized patients with rheumatic diseases : A monocentric observational and case-control study in northern Italy. Reumatismo, 75(2). https://doi.org/10.4081/reumatismo.2023.1542

Unlu, A., Truong, S., Sawhney, N., Sivelae, J., & Tammi, T. (2024). Tracing the dynamics of misinformation and vaccine stance in Finland amid COVID-19. INFORMATION COMMUNICATION & SOCIETY. https://doi.org/10.1080/1369118X.2024.2331756

Uphoff, H., An der Heiden, M., Schweiger, B., Campe, H., Beier, D., Helmeke, C., Littmann, M., Haas, W., Buda, S., Faensen, D., Feig, M., Altmann, D., Wichmann, O., Eckmanns, T., & Buchholz, U. (2011). Effectiveness of the AS03-adjuvanted vaccine against pandemic influenza virus A/(H1N1) 2009—A comparison of two methods; Germany, 2009/10. PloS One, 6(7), e19932. https://doi.org/10.1371/journal.pone.0019932

Uppalaiah, B., Reddy, D., Rajalakshmi, K., Vignesh, P., Govindan, V., & Donganont, S. (2025). Hybrid modeling approaches for predicting COVID-19 mortality: A comparative study across USA, France, and India. RESULTS IN ENGINEERING, 26. https://doi.org/10.1016/j.rineng.2025.105092

Urdiales, T., Dernie, F., Català, M., Prats-Uribe, A., Prats, C., & Prieto-Alhambra, D. (2023). Association between ethnic background and COVID-19 morbidity, mortality and vaccination in England : A multistate cohort analysis using the UK Biobank. BMJ Open, 13(9). https://doi.org/10.1136/bmjopen-2023-074367

Urso, P., Cattaneo, A., Pulvirenti, S., Vercelli, F., Cavallo, D. M., & Carrer, P. (2023). Early-phase pandemic in Italy: Covid-19 spread determinant factors. Heliyon, 9(4), e15358. https://doi.org/10.1016/j.heliyon.2023.e15358

Vagliano, I., Brinkman, S., Abu-Hanna, A., Arbous, M. S., Dongelmans, D. A., Elbers, P. W. G., de Lange, D. W., van der Schaar, M., de Keizer, N. F., Schut, M. C., & Dutch COVID-19 Research Consortium. (2022). Can we reliably automate clinical prognostic modelling ? A retrospective cohort study for ICU triage prediction of in-hospital mortality of COVID-19 patients in the Netherlands. International Journal of Medical Informatics, 160. https://doi.org/10.1016/j.ijmedinf.2022.104688

Vagliano, I., Schut, M. C., Abu-Hanna, A., Dongelmans, D. A., de Lange, D. W., Gommers, D., Cremer, O. L., Bosman, R. J., Rigter, S., Wils, E.-J., Frenzel, T., de Jong, R., Peters, M. A. A., Kamps, M. J. A., Ramnarain, D., Nowitzky, R., Nooteboom, F. G. C. A., de Ruijter, W., Urlings-Strop, L. C., … Dutch COVID-19 Research

Consortium, the D. I. D. S. A. C.-19 C. (2022). Assess and validate predictive performance of models for in-hospital mortality in COVID-19 patients : A retrospective cohort study in the Netherlands comparing the value of registry data with high-granular electronic health records. International Journal of Medical Informatics, 167. https://doi.org/10.1016/j.ijmedinf.2022.104863

Valent, F., & Tullio, A. (2019). Glycaemic control, antidiabetic medications and influenza vaccination coverage among patients with diabetes in Udine, Italy. Family Medicine and Community Health, 7(3). https://doi.org/10.1136/fmch-2019-000198

Valent, F., Gentilini, M. A., & Fateh-Moghadam, P. (2021). COVID-19 hospitalizations in children in the Autonomous Province of Trento (Northern Italy), year 2020. Epidemiologia E Prevenzione, 45(6). https://doi.org/10.19191/EP21.6.112

Valentowitsch, J. (2020). Flattening the COVID-19 Curve : The Impact of Contact Restrictions on the Infection Curve in Germany. Gesundheitswesen (Bundesverband Der Arzte Des Offentlichen Gesundheitsdienstes (Germany)), 82(7). https://doi.org/10.1055/a-1194-4967

Valera Felices, J. L., Gimeno Cardells, A., Gimeno Peribañez, M. A., Díaz-Pérez, D., Miranda Valladares, S., & Peña-Otero, D. (2021). [Risk factors associated with SARS-CoV-2 infection among health professionals in Spain]. Anales Del Sistema Sanitario De Navarra, 44(3). https://doi.org/10.23938/ASSN.0971

Valero, C., Barba, R., Marcos, D. P., Puente, N., Riancho, J. A., & Santurtún, A. (2022). Influence of weather factors on the incidence of COVID-19 in Spain. Medicina Clinica (English Ed.), 159(6). https://doi.org/10.1016/j.medcle.2021.10.018

Vallecillo, G., Durán, X., Canosa, I., Roquer, A., Martinez, M. C., & Perelló, R. (2022). COVID-19 vaccination coverage and vaccine hesitancy among people with opioid use disorder in Barcelona, Spain. Drug and Alcohol Review, 41(6). https://doi.org/10.1111/dar.13502

Valletta, M., Canevelli, M., D'Antonio, F., Trebbastoni, A., Talarico, G., Campanelli, A., Monti, M., Di Vita, A., Salati, E., Imbriano, L., Margiotta, R., Barbetti, S., Diana, S., Buscarnera, S., Blasi, M., Salzillo, M., Pugliese, G., Vanacore, N., & Bruno, G. (2022). Prevalence and Safety of COVID-19 Vaccination in Community-Dwelling People with Dementia : Findings from a Tertiary Memory Clinic in Italy. JOURNAL OF ALZHEIMERS DISEASE, 87(4). https://doi.org/10.3233/JAD-220077

Valls, J., Tobías, A., Satorra, P., & Tebé, C. (2021). [COVID19-Tracker : A shiny app to analise data on SARS-CoV-2 epidemic in Spain]. Gaceta Sanitaria, 35(1). https://doi.org/10.1016/j.gaceta.2020.04.002

Valour, F., Bénet, T., & Chidiac, C. (2013). Pandemic A(H1N1)2009 influenza vaccination in Lyon University Hospitals, France: Perception and attitudes of hospital workers. Vaccine, 31(4), 592–595. https://doi.org/10.1016/j.vaccine.2012.11.070

van 't Klooster, T., Wielders, C., Donker, T., Isken, L., Meijer, A., van den Wijngaard, C., van der Sande, M., & van der Hoek, W. (2010). Surveillance of Hospitalisations for 2009 Pandemic Influenza A(H1N1) in the Netherlands, 5 June-31 December 2009. EUROSURVEILLANCE, 15(2), 9–16.

Van Cauteren, D., Vaux, S., de Valk, H., Le Strat, Y., Vaillant, V., & Lévy-Bruhl, D. (2012). Burden of influenza, healthcare seeking behaviour and hygiene measures during the A(H1N1)2009 pandemic in France: A population based study. BMC Public Health, 12, 947. https://doi.org/10.1186/1471-2458-12-947

van de Berg, S., Coyer, L., von Both, U., Scheuerer, T., Kolberg, L., Hoch, M., & Böhmer, M. M. (2024). Coverage and determinants of COVID-19 child vaccination in Munich, Germany in October 2022-January 2023 : Results of the COVIP-Virenwächter study. European Journal of Pediatrics, 183(9). https://doi.org/10.1007/s00431-024-05617-0

van den Berg, J. M., Blom, M. T., Overbeek, J. A., Remmelzwaal, S., Herings, R. M. C., & Elders, P. J. M. (2025). Rates of SARS-CoV-2 Breakthrough Infection or Severe COVID-19 and Associated Risk Factors After Primary and Booster Vaccination Against COVID-19 in the Netherlands. Vaccines, 13(6), 564. https://doi.org/10.3390/vaccines13060564

Van den Borre, L., Devleesschauwer, B., Gadeyne, S., Vanthomme, K., & Willaert, D. (2025). Understanding excess mortality during COVID in Belgium : The influence of pre-existing health status and social factors. Archives of Public Health = Archives Belges De Sante Publique, 83(1). https://doi.org/10.1186/s13690-025-01499-2

Van den Borre, L., Gadeyne, S., Devleesschauwer, B., & Vanthomme, K. (2024). Uncovering the toll of the first three COVID-19 waves : Excess mortality and social patterns in Belgium. Archives of Public Health = Archives Belges De Sante Publique, 82(1). https://doi.org/10.1186/s13690-024-01444-9

van den Broek-Altenburg, E., & Atherly, A. (2021). Adherence to COVID-19 policy measures : Behavioral insights from The Netherlands and Belgium. PloS One, 16(5). https://doi.org/10.1371/journal.pone.0250302

Van der Heyden, J., Leclercq, V., Duysburgh, E., Cornelissen, L., Desombere, I., Roukaerts, I., & Gisle, L. (2024). Prevalence of SARS-CoV-2 antibodies and associated factors in the adult population of Belgium : A general population cohort study between March 2021 and April 2022. Archives of Public Health = Archives Belges De Sante Publique, 82(1). https://doi.org/10.1186/s13690-024-01298-1

van der Weerd, W., Timmermans, D. R., Beaujean, D. J., Oudhoff, J., & van Steenbergen, J. E. (2011). Monitoring the level of government trust, risk perception and intention of the general public to adopt protective measures during the influenza A (H1N1) pandemic in The Netherlands. BMC Public Health, 11, 575. https://doi.org/10.1186/1471-2458-11-575

van der Maaden, T., Mutubuki, E. N., de Bruijn, S., Leung, K. Y., Knoop, H., Slootweg, J., Tulen, A. D., Wong, A., van Hoek, A. J., Franz, E., & Van den Wijngaard, C. C. (2022). Prevalence and severity of symptoms 3 months after infection with SARS-CoV-2 compared to test-negative and population controls in the Netherlands. medRxiv, (van der Maaden T., tessa.van.der.maaden@rivm.nl; Mutubuki E.N.; de Bruijn S.; Leung K.Y.; Tulen A.D.; Wong A.; van Hoek A.J.; Franz E.; Van den Wijngaard C.C.) Center for Infectious Disease Control, National Institute for Public Health and the Environment (RIVM), Bilthoven, Netherlands. https://doi.org/10.1101/2022.06.15.22276439

van der Pol, S., Postma, M. J., & Boersma, C. (2024). Antivirals to prepare for surges in influenza cases : An economic evaluation of baloxavir marboxil for the Netherlands. The European Journal of Health Economics: HEPAC: Health Economics in Prevention and Care, 25(9). https://doi.org/10.1007/s10198-024-01683-1

van der Pol, S., Zeevat, F., Postma, M. J., & Boersma, C. (2024). Cost-effectiveness of high-dose influenza vaccination in the Netherlands : Incorporating the impact on both respiratory and cardiovascular hospitalizations. Vaccine, 42(15). https://doi.org/10.1016/j.vaccine.2024.04.040

van Gageldonk-Lafeber, A. B., Hooiveld, M., Meijer, A., Donker, G. A., Veldman-Ariesen, M.-J., van der Hoek, W., & van der Sande, M. A. B. (2011). The relative clinical impact of 2009 pandemic influenza A (H1N1) in the community compared to seasonal influenza in the Netherlands was most marked among 5-14 year olds. Influenza and Other Respiratory Viruses, 5(6), e513-520. https://doi.org/10.1111/j.1750-2659.2011.00260.x

van Gageldonk-Lafeber, R. A. B., Riesmeijer, R. M., Friesema, I. H. M., Meijer, A., Isken, L. D., Timen, A., & van der Sande, M. A. B. (2011). Case-based reported mortality associated with laboratory-confirmed influenza A(H1N1) 2009 virus infection in the Netherlands: The 2009-2010 pandemic season versus the 2010-2011 influenza season. BMC Public Health, 11, 758. https://doi.org/10.1186/1471-2458-11-758

Van Goethem, N., Chung, P. Y. J., Meurisse, M., Vandromme, M., De Mot, L., Brondeel, R., Stouten, V., Klamer, S., Cuypers, L., Braeye, T., Catteau, L., Nevejan, L., van Loenhout, J. A. F., & Blot, K. (2022). Clinical Severity of SARS-CoV-2 Omicron Variant Compared with Delta among Hospitalized COVID-19 Patients in Belgium during Autumn and Winter Season 2021-2022. Viruses, 14(6). https://doi.org/10.3390/v14061297

Van Goethem, N., Vandromme, M., Van Oyen, H., Haarhuis, F., Brondeel, R., Catteau, L., André, E., Cuypers, L., Belgian Collaborative Group on COVID-19 Hospital surveillance, COVID-19 Genomics Belgium consortium, Blot, K., & Serrien, B. (2022). Severity of infection with the SARS-CoV-2 B.1.1.7 lineage among hospitalized COVID-19 patients in Belgium. PloS One, 17(6). https://doi.org/10.1371/journal.pone.0269138

Van Goethem, N., Vilain, A., Wyndham-Thomas, C., Deblonde, J., Bossuyt, N., Lernout, T., Rebolledo Gonzalez, J., Quoilin, S., Melis, V., & Van Beckhoven, D. (2020). Rapid establishment of a national surveillance

of COVID-19 hospitalizations in Belgium. Archives of Public Health = Archives Belges De Sante Publique, 78(1). https://doi.org/10.1186/s13690-020-00505-z

van Halem, K., Bruyndonckx, R., van der Hilst, J., Cox, J., Driesen, P., Opsomer, M., Van Steenkiste, E., Stessel, B., Dubois, J., & Messiaen, P. (2020). Risk factors for mortality in hospitalized patients with COVID-19 at the start of the pandemic in Belgium: A retrospective cohort study. BMC Infectious Diseases, 20(1), 897. https://doi.org/10.1186/s12879-020-05605-3

Van Heirstraeten, L., Ekinci, E., Smet, M., Berkell, M., Willen, L., Coppens, J., Spiessens, A., Xavier, B. B., Lammens, C., Verhaegen, J., Van Damme, P., Goossens, H., Beutels, P., Matheeussen, V., Desmet, S., Theeten, H., & Malhotra-Kumar, S. (2022). Detection of SARS-CoV-2 in young children attending day-care centres in Belgium, May 2020 to February 2022. Euro Surveillance: Bulletin Europeen Sur Les Maladies Transmissibles = European Communicable Disease Bulletin, 27(21). https://doi.org/10.2807/1560-7917.ES.2022.27.21.2200380

van Leeuwen, E., Wilkins, N., & Watson, C. H. (2025). Influenza hospital admissions prevented by vaccination: A transmission dynamic analysis of the 2022/2023 and 2023/2024 programmes in England. medRxiv. https://doi.org/10.1101/2025.05.11.25327378

van Boven, M., Hetebrij, W. A., Swart, A., Nagelkerke, E., van der Beek, R. F., Stouten, S., Hoogeveen, R. T., Miura, F., Kloosterman, A., van der Drift, A.-M. R., Welling, A., Lodder, W. J., & de Roda Husman, A. M. (2023). Patterns of SARS-CoV-2 circulation revealed by a nationwide sewage surveillance programme, the Netherlands, August 2020 to February 2022. Euro Surveillance: Bulletin Europeen Sur Les Maladies Transmissibles = European Communicable Disease Bulletin, 28(25). https://doi.org/10.2807/1560-7917.ES.2023.28.25.2200700

van Doorn, E., Darvishian, M., Dijkstra, F., Bijlsma, M. J., Donker, G. A., de Lange, M. M. A., Cadenau, L. M., Hak, E., & Meijer, A. (2017). [Effectiveness of influenza vaccine in the Netherlands : Predominant circulating virus type and vaccine match are important conditions]. Nederlands Tijdschrift Voor Geneeskunde, 161. http://www.ncbi.nlm.nih.gov/pubmed/28558853

van Ewijk, C. E., Kooijman, M. N., Fanoy, E., Raven, S. F., Middeldorp, M., Shah, A., de Gier, B., de Melker, H. E., Hahné, S. J., & Knol, M. J. (2022). COVID-19 vaccine effectiveness against SARS-CoV-2 infection during the Delta period, a nationwide study adjusting for chance of exposure, the Netherlands, July to December 2021. Euro Surveillance: Bulletin Europeen Sur Les Maladies Transmissibles = European Communicable Disease Bulletin, 27(45). https://doi.org/10.2807/1560-7917.ES.2022.27.45.2200217

van Iersel, S. C. J. L., McDonald, S. A., de Gier, B., Knol, M. J., de Melker, H. E., van Werkhoven, C. H., Hahné, S. J. M., van Hoek, A. J., Hofhuis, A., Maxwell, A., Niessen, A., Teirlinck, A., Valk, A.-W., Verstraten, C., Laarman, C., Berry, D., van Wees, D., van Meijeren, D., Klinkenberg, D., … Boere, T. (2022). Number of COVID-19 hospitalisations averted by vaccination : Estimates for the Netherlands, January 6, 2021 through August 30, 2022. medRxiv, (van Iersel S.C.J.L., ddesalve@som.geisinger.edu; McDonald S.A.; de Gier B.; Knol M.J.; Melker H.E.; van Werkhoven C.H.; Hahné S.J.M.) Center for Infectious Disease Control, National Institute for Public Health and the Environment (RIVM), Bilthoven, Netherlands. https://doi.org/10.1101/2022.12.20.22283713

van Leeuwen, E., Panovska-Griffiths, J., Elgohari, S., Charlett, A., & Watson, C. (2023). The interplay between susceptibility and vaccine effectiveness control the timing and size of an emerging seasonal influenza wave in England. Epidemics, 44. https://doi.org/10.1016/j.epidem.2023.100709

van Lier, A., Steens, A., Ferreira, J. A., van der Maas, N. A. T., & de Melker, H. E. (2012). Acceptance of vaccination during pregnancy : Experience with 2009 influenza A (H1N1) in the Netherlands. Vaccine, 30(18). https://doi.org/10.1016/j.vaccine.2012.02.030

van Loenhout, J. A. F., Vanderplanken, K., Van den Broucke, S., & Aujoulat, I. (2022). COVID-19 measures in Belgium : How perception and adherence of the general population differ between time periods. BMC Public Health, 22(1). https://doi.org/10.1186/s12889-022-12654-7

van Loon, W., Theuring, S., Hommes, F., Mall, M. A., Seybold, J., Kurth, T., & Mockenhaupt, F. P. (2021). Prevalence of SARS-CoV-2 Infections Among Students, Teachers, and Household Members During Lockdown

and Split Classes in Berlin, Germany. JAMA Network Open, 4(9). https://doi.org/10.1001/jamanetworkopen.2021.27168

van Roekel, C., Labuschagne, L., Pijpers, J., van Roon, A., Smagge, B., Ferreira, J. A., Hahné, S., & de Melker, H. (2023). Determinants of COVID-19 booster uptake in the Netherlands, autumn 2022 : How well were those at risk for severe disease reached? medRxiv, (van Roekel C., caren.van.roekel@rivm.nl; Labuschagne L.; Pijpers J.; van Roon A.; Smagge B.; Hahné S.; de Melker H.) Epidemiology and Surveillance, National Institute for Public Health and the Environment, Bilthoven, Netherlands. https://doi.org/10.1101/2023.08.04.23293632

van Roekel, C., Labuschagne, L., Pijpers, J., van Roon, A., Smagge, B., Ferreira, J. A., Hahné, S., & de Melker, H. (2024). Factors associated with COVID-19 autumn 2022 booster uptake in the Netherlands among older adults aged ≥ 60 years and younger adults with chronic conditions. Vaccine, 42(2). https://doi.org/10.1016/j.vaccine.2023.12.027

van Werkhoven, C. H., Valk, A.-W., Smagge, B., de Melker, H. E., Knol, M. J., Hahné, S. J., van den Hof, S., & de Gier, B. (2024). Early COVID-19 vaccine effectiveness of XBB.1.5 vaccine against hospitalisation and admission to intensive care, the Netherlands, 9 October to 5 December 2023. Euro Surveillance: Bulletin Europeen Sur Les Maladies Transmissibles = European Communicable Disease Bulletin, 29(1). https://doi.org/10.2807/1560-7917.ES.2024.29.1.2300703

Vanbesien, M., Molenberghs, G., Geenen, C., Thibaut, J., Gorissen, S., André, E., & Raymenants, J. (2022). Risk factors for SARS-CoV-2 transmission in student residences : A case-ascertained study in Leuven, Belgium from October 2020 until May 2021. medRxiv, (Vanbesien M.) Faculty of medicine, KU Leuven, Belgium. https://doi.org/10.1101/2022.03.23.22272836

Vandentorren, S., Smaïli, S., Chatignoux, E., Maurel, M., Alleaume, C., Neufcourt, L., Kelly-Irving, M., & Delpierre, C. (2022). The effect of social deprivation on the dynamic of SARS-CoV-2 infection in France : A population-based analysis. The Lancet. Public Health, 7(3). https://doi.org/10.1016/S2468-2667(22)00007-X

Vandercam, G., Simon, A., Scohy, A., Belkhir, L., Kabamba, B., Rodriguez-Villalobos, H., & Yombi, J. C. (2020). Clinical characteristics and humoral immune response in healthcare workers with COVID-19 in a teaching hospital in Belgium. The Journal of Hospital Infection, 106(4). https://doi.org/10.1016/j.jhin.2020.09.018

Vanderplanken, K., Van den Broucke, S., Aujoulat, I., & van Loenhout, J. A. F. (2021). The Relation between Perceived and Actual Understanding and Adherence : Results from a National Survey on COVID-19 Measures in Belgium. International Journal of Environmental Research and Public Health, 18(19). https://doi.org/10.3390/ijerph181910200

Vanthomme, K., Gadeyne, S., Lusyne, P., & Vandenheede, H. (2021). A population-based study on mortality among Belgian immigrants during the first COVID-19 wave in Belgium. Can demographic and socioeconomic indicators explain differential mortality? SSM - Population Health, 14. https://doi.org/10.1016/j.ssmph.2021.100797

Vargas Molina, S. A., Barrionuevo, J. F. S., & Perles Roselló, M. J. (2025). Effect of urban structure, population density and proximity to contagion on COVID-19 infections during the SARS-CoV-2 Alpha and Omicron waves in Málaga, Spain, March 2020 to December 2021. Euro Surveillance: Bulletin Europeen Sur Les Maladies Transmissibles = European Communicable Disease Bulletin, 30(3). https://doi.org/10.2807/1560-7917.ES.2025.30.3.2400174

Variskallio, S., Moustgaard, H., Remes, H., & Martikainen, P. (2025). Association of parental education with adolescents' COVID-19 vaccine uptake: A nationwide register-based study in Finland. Vaccine, 63, 127615. https://doi.org/10.1016/j.vaccine.2025.127615

Vauhkonen, H., Nguyen, P. T., Kant, R., Plyusnin, I., Erdin, M., Kurkela, S., Liimatainen, H., Ikonen, N., Blomqvist, S., Liitsola, K., Lindh, E., Helve, O., Jarva, H., Loginov, R., Palva, A., Hannunen, T., Hannula, S., Parry, M., Kauppi, P., … Vapalahti, O. (2022). Introduction and Rapid Spread of SARS-CoV-2 Omicron Variant and Dynamics of BA.1 and BA.1.1 Sublineages, Finland, December 2021. Emerging Infectious Diseases, 28(6). https://doi.org/10.3201/eid2806.220515

Vaux, S., Fonteneau, L., Venier, A.-G., Gautier, A., Soing Altrach, S., Parneix, P., & Lévy-Bruhl, D. (2022). Influenza vaccination coverage of professionals working in nursing homes in France and related determinants, 2018-2019 season : A cross-sectional survey. BMC Public Health, 22(1). https://doi.org/10.1186/s12889-022-13412-5

Vaux, S., Noël, D., Fonteneau, L., Guthmann, J.-P., & Lévy-Bruhl, D. (2010). Influenza vaccination coverage of healthcare workers and residents and their determinants in nursing homes for elderly people in France : A cross-sectional survey. BMC Public Health, 10. https://doi.org/10.1186/1471-2458-10-159

Vaux, S., Van Cauteren, D., Guthmann, J.-P., Le Strat, Y., Vaillant, V., de Valk, H., & Lévy-Bruhl, D. (2011). Influenza vaccination coverage against seasonal and pandemic influenza and their determinants in France: A cross-sectional survey. BMC Public Health, 11, 30. https://doi.org/10.1186/1471-2458-11-30

Vázquez Rivas, F., Nieto Schwarz, S., Villarreal Carreño, J., Deschamps Perdomo, Á., Villanueva, G. P., Garrafa, M., & Del Campo, M. T. (2021). Serological study of healthcare workers in four different hospitals in Madrid (Spain) with no previous history of COVID-19. Occupational and Environmental Medicine. https://doi.org/10.1136/oemed-2020-107001

Vázquez-Fernández del Pozo, S., Hernández-Barrera, V., Carrasco-Garrido, P., Gil de Miguel, A., & Jiménez-García, R. (2007). [Changes in influenza vaccination coverage in Spain from 2001 to 2003. Analysis by autonomous communities]. Gaceta Sanitaria, 21(1). https://doi.org/10.1157/13099114

Vázquez-Morón, S., Iglesias-Caballero, M., Lepe, J. A., Garcia, F., Melón, S., Marimon, J. M., García de Viedma, D., Folgueira, M. D., Galán, J. C., López-Causapé, C., Benito-Ruesca, R., Alcoba-Florez, J., Gonzalez Candelas, F., Toro, M. de, Fajardo, M., Ezpeleta, C., Lázaro, F., Pérez Castro, S., Cuesta, I., … On Behalf Of Relecov Network Members, null. (2023). Enhancing SARS-CoV-2 Surveillance through Regular Genomic Sequencing in Spain : The RELECOV Network. International Journal of Molecular Sciences, 24(10). https://doi.org/10.3390/ijms24108573

Veenstra, T., van Schelven, P. D., Ten Have, Y. M., Swaan, C. M., & van den Akker, W. M. R. (2023). Extensive Spread of SARS-CoV-2 Delta Variant among Vaccinated Persons during 7-Day River Cruise, the Netherlands. Emerging Infectious Diseases, 29(4). https://doi.org/10.3201/eid2904.221433

Velay, A., Gallais, F., Wendling, M.-J., Bayer, S., Reix, N., Schneider, A., Glady, L., Collongues, N., Lessinger, J.-M., Hansmann, Y., Kling-Pillitteri, L., De Sèze, J., Gonzalez, M., Schmidt-Mutter, C., Meyer, N., & Fafi-Kremer, S. (2022). COVID-19 exposure in SARS-CoV-2-seropositive hospital staff members during the first pandemic wave at Strasbourg University Hospital, France. Infectious Diseases Now, 52(1), 23–30. https://doi.org/10.1016/j.idnow.2021.11.002

Ventura, M., Di Napoli, A., Caranci, N., Adorno, V., Bartolini, L., Corsaro, A., Spadea, T., Rusciani, R., Di Girolamo, C., Cacciani, L., Agabiti, N., Profili, F., Milli, C., Silvestri, C., Cernigliaro, A., Giorgi Rossi, P., D’Amato, S., & Petrelli, A. (2024). [Impact of COVID-19 on foreign population resident in urban areas of Italy : Selection of indicators, data sources, and definition of geographical stratification levels]. Epidemiologia E Prevenzione, 48(4-5). https://doi.org/10.19191/EP24.4-5.S1.112

Vera-Punzano, N., Navascués, A., Armendáriz, L., Viguria, N., Herranz-Aguirre, M., García Cenoz, M., Trobajo-Sanmartín, C., Echeverria, A., Martínez-Baz, I., Ezpeleta, C., Ezpeleta, G., & Castilla, J. (2025). [Nirsevimab immunization effectiveness against respiratory syncytial virus hospitalization in newborns: Two season of use in Navarre, Spain]. Anales Del Sistema Sanitario De Navarra, 48(2), e1133. https://doi.org/10.23938/ASSN.1133

Vera-Punzano, N., Trobajo-Sanmartín, C., Navascués, A., Echeverria, A., Casado, I., Ezpeleta, C., Castilla, J., & Martínez-Baz, I. (2025). Hospitalisation due to respiratory syncytial virus in a population-based cohort of older adults in Spain, 2016/17 to 2019/20. Euro Surveillance: Bulletin Europeen Sur Les Maladies Transmissibles = European Communicable Disease Bulletin, 30(10), 2400364. https://doi.org/10.2807/1560-7917.ES.2025.30.10.2400364

Verbeeck, J., Vandersmissen, G., Peeters, J., Klamer, S., Hancart, S., Lernout, T., Dewatripont, M., Godderis, L., & Molenberghs, G. (2021). Confirmed COVID-19 Cases per Economic Activity during Autumn Wave in

Belgium. International Journal of Environmental Research and Public Health, 18(23). https://doi.org/10.3390/ijerph182312489

Vercelli, M., Lillini, R., Arata, L., Zangrillo, F., Bagnasco, A., Sasso, L., Magliani, A., Gasparini, R., Amicizia, D., & Panatto, D. (2018). Analysis of influenza vaccination coverage among the elderly in Genoa (Italy) based on a deprivation index, 2009-2013. Journal of Preventive Medicine and Hygiene, 59(4 Suppl 2). https://doi.org/10.15167/2421-4248/jpmh2018.59.4s2.1171

Verger, P., Cortaredona, S., Pulcini, C., Casanova, L., Peretti-Watel, P., & Launay, O. (2015). Characteristics of patients and physicians correlated with regular influenza vaccination in patients treated for type 2 diabetes : A follow-up study from 2008 to 2011 in southeastern France. Clinical Microbiology and Infection: The Official Publication of the European Society of Clinical Microbiology and Infectious Diseases, 21(10). https://doi.org/10.1016/j.cmi.2015.06.017

Verger, P., Fressard, L., Cortaredona, S., Lévy-Bruhl, D., Loulergue, P., Galtier, F., & Bocquier, A. (2018). Trends in seasonal influenza vaccine coverage of target groups in France, 2006/07 to 2015/16: Impact of recommendations and 2009 influenza A(H1N1) pandemic. Euro Surveillance: Bulletin Europeen Sur Les Maladies Transmissibles = European Communicable Disease Bulletin, 23(48), 1700801. https://doi.org/10.2807/1560-7917.ES.2018.23.48.1700801

Verger, P., Peretti-Watel, P., Gagneux-Brunon, A., Botelho-Nevers, E., Sanchez, A., Gauna, F., Fressard, L., Bonneton, M., Launay, O., & Ward, J. K. (2021). Acceptance of childhood and adolescent vaccination against COVID-19 in France : A national cross-sectional study in May 2021. Human Vaccines & Immunotherapeutics, 17(12). https://doi.org/10.1080/21645515.2021.2004838

Vermeiren, E., van Loenhout, J. A. F., Nasiadka, L., Stouten, V., Billuart, M., Van Evercooren, I., Catteau, L., & Hubin, P. (2023). Factors underlying COVID-19 booster vaccine uptake among adults in Belgium. BMC Research Notes, 16(1). https://doi.org/10.1186/s13104-023-06608-4

Vicente, D., Cilla, G., Montes, M., Mendiola, J., & Pérez-Trallero, E. (2009). Rapid spread of drug-resistant influenza A viruses in the Basque Country, northern Spain, 2000-1 to 2008-9. Euro Surveillance: Bulletin Europeen Sur Les Maladies Transmissibles = European Communicable Disease Bulletin, 14(20). https://doi.org/10.2807/ese.14.20.19215-en

Vicentini, C., Bordino, V., Gardois, P., & Zotti, C. M. (2020). Early assessment of the impact of mitigation measures on the COVID-19 outbreak in Italy. Public Health, 185. https://doi.org/10.1016/j.puhe.2020.06.028

Viedma, E., Dahdouh, E., González-Alba, J. M., González-Bodi, S., Martínez-García, L., Lázaro-Perona, F., Recio, R., Rodríguez-Tejedor, M., Folgueira, M. D., Cantón, R., Delgado, R., García-Rodríguez, J., Galán, J. C., Mingorance, J., & On Behalf Of The Sars-CoV-Working Groups, null. (2021). Genomic Epidemiology of SARS-CoV-2 in Madrid, Spain, during the First Wave of the Pandemic: Fast Spread and Early Dominance by D614G Variants. Microorganisms, 9(2), 454. https://doi.org/10.3390/microorganisms9020454

Viguria, N., Martínez-Baz, I., Moreno-Galarraga, L., Sierrasesúmaga, L., Salcedo, B., & Castilla, J. (2018). Respiratory syncytial virus hospitalization in children in northern Spain. PloS One, 13(11). https://doi.org/10.1371/journal.pone.0206474

Vila-Candel, R., Navarro-Illana, P., Navarro-Illana, E., Castro-Sánchez, E., Duke, K., Soriano-Vidal, F. J., Tuells, J., & Díez-Domingo, J. (2016). Determinants of seasonal influenza vaccination in pregnant women in Valencia, Spain. BMC Public Health, 16(1). https://doi.org/10.1186/s12889-016-3823-1

Vila-Corcoles, A., Satue-Gracia, E., Vila-Rovira, A., de Diego-Cabanes, C., Forcadell-Peris, M. J., Hospital-Guardiola, I., Ochoa-Gondar, O., & Basora-Gallisa, J. (2021). COVID19-related and all-cause mortality risk among middle-aged and older adults across the first epidemic wave of SARS-COV-2 infection : A population-based cohort stuJune 2020.dy in Southern Catalonia, Spain, March-. BMC Public Health, 21(1). https://doi.org/10.1186/s12889-021-11879-2

Vilca, L. M., Cesari, E., Tura, A. M., Di Stefano, A., Vidiri, A., Cavaliere, A. F., & Cetin, I. (2020). Barriers and facilitators regarding influenza and pertussis maternal vaccination uptake : A multi-center survey of pregnant

women in Italy. European Journal of Obstetrics, Gynecology, and Reproductive Biology, 247. https://doi.org/10.1016/j.ejogrb.2020.02.007

Vilca, L. M., Sarno, L., Cesari, E., Vidiri, A., Antonazzo, P., Ravennati, F., Cavaliere, A. F., Guida, M., & Cetin, I. (2021). Differences between influenza and pertussis vaccination uptake in pregnancy : A multi-center survey study in Italy. European Journal of Public Health, 31(6). https://doi.org/10.1093/eurpub/ckab095

Vilca, L. M., Verma, A., Bonati, M., & Campins, M. (2018). Impact of influenza on outpatient visits and hospitalizations among pregnant women in Catalonia, Spain. The Journal of Infection, 77(6). https://doi.org/10.1016/j.jinf.2018.06.015

Vilcu, A. M., Souty, C., Enouf, V., Capai, L., Turbelin, C., Masse, S., Behillil, S., Valette, M., Guerrisi, C., Rossignol, L., Blanchon, T., Lina, B., Hanslik, T., & Falchi, A. (2018). Estimation of seasonal influenza vaccine effectiveness using data collected in primary care in France : Comparison of the test-negative design and the screening method. Clinical Microbiology and Infection: The Official Publication of the European Society of Clinical Microbiology and Infectious Diseases, 24(4). https://doi.org/10.1016/j.cmi.2017.09.003

Villani, E. R., Carfì, A., Di Paola, A., Palmieri, L., Donfrancesco, C., Lo Noce, C., Taruscio, D., Meli, P., Salerno, P., Kodra, Y., Pricci, F., Tamburo de Bella, M., Floridia, M., Onder, G., & Italian National Institute of Health CoVID-19 Mortality Group. (2020). Clinical characteristics of individuals with Down syndrome deceased with CoVID-19 in Italy-A case series. American Journal of Medical Genetics. Part A, 182(12). https://doi.org/10.1002/ajmg.a.61867

Vimercati, L., Bianchi, F. P., Mansi, F., Ranieri, B., Stefanizzi, P., De Nitto, S., & Tafuri, S. (2019). Influenza vaccination in health-care workers : An evaluation of an on-site vaccination strategy to increase vaccination uptake in HCWs of a South Italy Hospital. Human Vaccines & Immunotherapeutics, 15(12). https://doi.org/10.1080/21645515.2019.1625645

Vinceti, M., Balboni, E., Rothman, K. J., Teggi, S., Bellino, S., Pezzotti, P., Ferrari, F., Orsini, N., & Filippini, T. (2022). Substantial impact of mobility restrictions on reducing COVID-19 incidence in Italy in 2020. Journal of Travel Medicine, 29(6). https://doi.org/10.1093/jtm/taac081

Vinceti, M., Filippini, T., Rothman, K. J., Di Federico, S., & Orsini, N. (2021a). SARS-CoV-2 infection incidence during the first and second COVID-19 waves in Italy. Environmental Research, 197. https://doi.org/10.1016/j.envres.2021.111097

Vinceti, M., Filippini, T., Rothman, K. J., Di Federico, S., & Orsini, N. (2021b). The association between first and second wave COVID-19 mortality in Italy. BMC Public Health, 21(1). https://doi.org/10.1186/s12889-021-12126-4

Visco-Comandini, U., Castilletti, C., Lionetti, R., Meschi, S., Montalbano, M., Rianda, A., Taibi, C., Sorace, C., Guglielmo, N., Piccolo, P., Paci, P., Ettorre, G. M., & Gianpiero, D. (2022). High prevalence of asymptomatic SARS-CoV-2 infection in a cohort of liver transplant recipients in central Italy. Journal of Liver Transplantation, 5. https://doi.org/10.1016/j.liver.2021.100064

Vito, T., Gianfranco, C., Gianfranco, F., Mariarosaria, M., Marica, R., & Alessandro, C. (2025). Meteorological Factors and the Spread of COVID-19: A Territorial Analysis in Italy. METEOROLOGICAL APPLICATIONS, 32(2). https://doi.org/10.1002/met.70048

Vittecoq, M., Roche, B., Cohen, J.-M., Renaud, F., Thomas, F., & Gauthier-Clerc, M. (2015). Does the weather play a role in the spread of pandemic influenza? A study of H1N1pdm09 infections in France during 2009-2010. Epidemiology and Infection, 143(16), 3384–3393. https://doi.org/10.1017/S0950268815000941

Vivanco-Hidalgo, R. M., Molina, I., Martinez, E., Roman-Viñas, R., Sánchez-Montalvá, A., Fibla, J., Pontes, C., Velasco Muñoz, C., & Real World Data Working Group. (2021). Incidence of COVID-19 in patients exposed to chloroquine and hydroxychloroquine : Results from a population-based prospective cohort in Catalonia, Spain, 2020. Euro Surveillance: Bulletin Europeen Sur Les Maladies Transmissibles = European Communicable Disease Bulletin, 26(9). https://doi.org/10.2807/1560-7917.ES.2021.26.9.2001202

Vogel, S., von Both, U., Nowak, E., Ludwig, J., Köhler, A., Lee, N., Dick, E., Rack-Hoch, A., Wicklein, B., Neusser, J., Wagner, T., Schubö, A., Ustinov, M., Schimana, W., Busche, S., Kolberg, L., & Hoch, M. (2022).

SARS-CoV-2 Saliva Mass Screening in Primary Schools : A 10-Week Sentinel Surveillance Study in Munich, Germany. Diagnostics (Basel, Switzerland), 12(1). https://doi.org/10.3390/diagnostics12010162

Volff, M., Tonon, D., Bommel, Y., Peres, N., Lagier, D., Agard, G., Jacquier, A., Bartoli, A., Carvelli, J., Max, H., Simeone, P., Blasco, V., Pastene, B., Loundou, A., Boyer, L., Leone, M., Velly, L., Bourenne, J., Boussen, S., … Bruder, N. (2021). Factors Associated with 90-Day Mortality in Invasively Ventilated Patients with COVID-19 in Marseille, France. Journal of Clinical Medicine, 10(23). https://doi.org/10.3390/jcm10235650

von der Beck, D., Seeger, W., Herold, S., Günther, A., & Löh, B. (2017). Characteristics and outcomes of a cohort hospitalized for pandemic and seasonal influenza in Germany based on nationwide inpatient data. PloS One, 12(7), e0180920. https://doi.org/10.1371/journal.pone.0180920

von Linstow, M.-L., Rudolfsen, J. H., Olsen, J., Skovdal, M., & Staerke, N. B. (2024). Burden of disease and cost of illness of infants less than 6 months of age hospitalised with respiratory syncytial virus in Denmark—A 10-year national register-based study. BMC Infectious Diseases, 24(1). https://doi.org/10.1186/s12879-024-09975-w

Vos, E. R. A., den Hartog, G., Schepp, R. M., Kaaijk, P., van Vliet, J., Helm, K., Smits, G., Wijmenga-Monsuur, A., Verberk, J. D. M., van Boven, M., van Binnendijk, R. S., de Melker, H. E., Mollema, L., & van der Klis, F. R. M. (2020). Nationwide seroprevalence of SARS-CoV-2 and identification of risk factors in the general population of the Netherlands during the first epidemic wave. Journal of Epidemiology and Community Health, 75(6). https://doi.org/10.1136/jech-2020-215678

Vos, L. M., Oosterheert, J. J., Kuil, S. D., Viveen, M., Bont, L. J., Hoepelman, A. I. M., & Coenjaerts, F. E. J. (2019). High epidemic burden of RSV disease coinciding with genetic alterations causing amino acid substitutions in the RSV G-protein during the 2016/2017 season in The Netherlands. Journal of Clinical Virology: The Official Publication of the Pan American Society for Clinical Virology, 112. https://doi.org/10.1016/j.jcv.2019.01.007

Vos, L. M., Teirlinck, A. C., Lozano, J. E., Vega, T., Donker, G. A., Hoepelman, A. I., Bont, L. J., Oosterheert, J. J., & Meijer, A. (2019). Use of the moving epidemic method (MEM) to assess national surveillance data for respiratory syncytial virus (RSV) in the Netherlands, 2005 to 2017. Euro Surveillance: Bulletin Europeen Sur Les Maladies Transmissibles = European Communicable Disease Bulletin, 24(20). https://doi.org/10.2807/1560-7917.ES.2019.24.20.1800469

Vremera, T., Furtunescu, F. L., Leustean, M., Rafila, A., David, A., Radu, I., Cornienco, A. M., Gatea, A., Ilie, C., Iancu, L. S., & Pistol, A. (2022). Detection of anti-SARS-CoV-2-Spike/RBD antibodies in vaccinated elderly from residential care facilities in Romania, April 2021. Frontiers in Epidemiology, 2, 944820. https://doi.org/10.3389/fepid.2022.944820

Vusirikala, A., Flannagan, J., Czachorowski, M., Zaidi, A., Twohig, K. A., Plugge, E., Ellaby, N., Rice, W., Dabrera, G., Chudasama, D. Y., & Lamagni, T. (2022). Impact of SARS-CoV-2 Alpha variant (B.1.1.7) on prisons, England. Public Health, 204. https://doi.org/10.1016/j.puhe.2021.12.018

Vynnycky, E., Pitman, R., Siddiqui, R., Gay, N., & Edmunds, W. J. (2008). Estimating the impact of childhood influenza vaccination programmes in England and Wales. Vaccine, 26(41). https://doi.org/10.1016/j.vaccine.2008.06.101

Wachtler, B., Michalski, N., Nowossadeck, E., Diercke, M., Wahrendorf, M., Santos-Hövener, C., Lampert, T., & Hoebel, J. (2020). Socioeconomic inequalities in the risk of SARS-CoV-2 infection—First results from an analysis of surveillance data from Germany. Journal of Health Monitoring, 5(Suppl 7). https://doi.org/10.25646/7057

Wagner, M., Lampos, V., Yom-Tov, E., Pebody, R., & Cox, I. J. (2017). Estimating the Population Impact of a New Pediatric Influenza Vaccination Program in England Using Social Media Content. Journal of Medical Internet Research, 19(12). https://doi.org/10.2196/jmir.8184

Wahrendorf, M., Rupprecht, C. J., Dortmann, O., Scheider, M., & Dragano, N. (2021). [Higher risk of COVID-19 hospitalization for unemployed : An analysis of health insurance data from 1.28 million insured individuals in

Germany]. Bundesgesundheitsblatt, Gesundheitsforschung, Gesundheitsschutz, 64(3). https://doi.org/10.1007/s00103-021-03280-6

Wahrendorf, M., Schaps, V., Reuter, M., Hoebel, J., Wachtler, B., Jacob, J., Alibone, M., & Dragano, N. (2023). [Occupational differences of COVID-19 morbidity and mortality in Germany. An analysis of health insurance data from 3.17 million insured persons]. Bundesgesundheitsblatt, Gesundheitsforschung, Gesundheitsschutz, 66(8). https://doi.org/10.1007/s00103-023-03738-9

Waize, M., Marijic, P., Marijam, A., Gkalapi, F., Turriani, E., Jakobs, F., Jaidhauser, I., Münch, D., Pedron, S., & Zarkadoulas, E. (2025). Public health impact and cost-effectiveness of the adjuvanted RSVPreF3 vaccine for respiratory syncytial virus prevention among adults aged 50 years and older in Germany. Expert Review of Vaccines, 24(1), 782–796. https://doi.org/10.1080/14760584.2025.2539887

Walker, J. L., Rentsch, C. T., McDonald, H. I., Bak, J., Minassian, C., Amirthalingam, G., Edelstein, M., & Thomas, S. (2021). Social determinants of pertussis and influenza vaccine uptake in pregnancy : A national cohort study in England using electronic health records. BMJ Open, 11(6). https://doi.org/10.1136/bmjopen-2020-046545

Walker, J. L., Zhao, H., Dabrera, G., Andrews, N., Thomas, S. L., Tsang, C., Ellis, J., Donati, M., & Pebody, R. G. (2020). Assessment of Effectiveness of Seasonal Influenza Vaccination During Pregnancy in Preventing Influenza Infection in Infants in England, 2013-2014 and 2014-2015. The Journal of Infectious Diseases, 221(1). https://doi.org/10.1093/infdis/jiz310

Walkowiak, M. P., Domaradzki, J., & Walkowiak, D. (2022). Better Late Than Never : Predictors of Delayed COVID-19 Vaccine Uptake in Poland. Vaccines, 10(4). https://doi.org/10.3390/vaccines10040528

Walkowiak, M. P., Domaradzki, J., & Walkowiak, D. (2023). Are We Facing a Tsunami of Vaccine Hesitancy or Outdated Pandemic Policy in Times of Omicron? Analyzing Changes of COVID-19 Vaccination Trends in Poland. Vaccines, 11(6), 1065. https://doi.org/10.3390/vaccines11061065

Walkowiak, M. P., Walkowiak, D., & Walkowiak, J. (2023). To vaccinate or to isolate ? Establishing which intervention leads to measurable mortality reduction during the COVID-19 Delta wave in Poland. Frontiers in Public Health, 11. https://doi.org/10.3389/fpubh.2023.1221964

Walkowiak, M. P., Walkowiak, J. B., & Walkowiak, D. (2022). More Time, Carrot-and-Stick, or Piling Coffins ? Estimating the Role of Factors Overcoming COVID-19 Vaccine Hesitancy in Poland and Lithuania in the Years 2021-2022. Vaccines, 10(9). https://doi.org/10.3390/vaccines10091523

Wallemacq, S., Danwang, C., Scohy, A., Belkhir, L., De Greef, J., Kabamba, B., & Yombi, J. C. (2022). A comparative analysis of the outcomes of patients with influenza or COVID-19 in a tertiary hospital in Belgium. Journal of Infection and Chemotherapy: Official Journal of the Japan Society of Chemotherapy, 28(11). https://doi.org/10.1016/j.jiac.2022.07.012

Walter, D., Böhmer, M. M., Heiden, M. an der, Reiter, S., Krause, G., & Wichmann, O. (2011). Monitoring pandemic influenza A(H1N1) vaccination coverage in Germany 2009/10—Results from thirteen consecutive cross-sectional surveys. Vaccine, 29(23), 4008–4012. https://doi.org/10.1016/j.vaccine.2011.03.069

Wang, C.-W., de Jong, E. P., Faure, J. A., Ellington, J. L., Chen, C.-H. S., & Chan, C.-C. (2022). A matter of trust : A qualitative comparison of the determinants of COVID-19 vaccine hesitancy in Taiwan, the United States, the Netherlands, and Haiti. Human Vaccines & Immunotherapeutics, 18(5). https://doi.org/10.1080/21645515.2022.2050121

Wang, H., Kwok, K. O., Li, R., & Riley, S. (2023). Forecasting regional-level COVID-19 hospitalisation in England as an ordinal variable using the machine learning method. medRxiv, (Wang H.; Riley S., s.riley@imperial.ac.uk) School of Public Health, Imperial College London, United Kingdom. https://doi.org/10.1101/2023.10.17.23297138

Wang, H., Varol, T., Gültzow, T., Zimmermann, H. M. L., Ruiter, R. A. C., & Jonas, K. J. (2024). Spatio-temporal distributions of COVID-19 vaccine doses uptake in the Netherlands : A Bayesian ecological modelling analysis. Epidemiology and Infection, 152. https://doi.org/10.1017/S0950268824001249

Wang, H., Xu, K., Li, Z., Pang, K., & He, H. (2020). Improved Epidemic Dynamics Model and Its Prediction for COVID-19 in Italy. APPLIED SCIENCES-BASEL, 10(14). https://doi.org/10.3390/app10144930

Wang, H.-I., Doran, T., Crooks, M. G., Khunti, K., Heightman, M., Gonzalez-Izquierdo, A., Qummer Ul Arfeen, M., Loveless, A., Banerjee, A., & Van Der Feltz-Cornelis, C. (2024). Prevalence, risk factors and characterisation of individuals with long COVID using Electronic Health Records in over 1.5 million COVID cases in England. The Journal of Infection, 89(4). https://doi.org/10.1016/j.jinf.2024.106235

Ward, H., Atchison, C., Whitaker, M., Ainslie, K. E. C., Elliott, J., Okell, L., Redd, R., Ashby, D., Donnelly, C. A., Barclay, W., Darzi, A., Cooke, G., Riley, S., & Elliott, P. (2021). SARS-CoV-2 antibody prevalence in England following the first peak of the pandemic. Nature Communications, 12(1), 905. https://doi.org/10.1038/s41467-021-21237-w

Ward, H., Atchison, C., Whitaker, M., Davies, B., Ashby, D., Darzi, A., Chadeau-Hyam, M., Riley, S., Donnelly, C. A., Barclay, W., Cooke, G. S., & Elliott, P. (2023). Design and Implementation of a National Program to Monitor the Prevalence of SARS-CoV-2 IgG Antibodies in England Using Self-Testing : The REACT-2 Study. American Journal of Public Health, 113(11). https://doi.org/10.2105/AJPH.2023.307381

Ward, H., Cooke, G. S., Atchison, C., Whitaker, M., Elliott, J., Moshe, M., Brown, J. C., Flower, B., Daunt, A., Ainslie, K., Ashby, D., Donnelly, C. A., Riley, S., Darzi, A., Barclay, W., & Elliott, P. (2021). Prevalence of antibody positivity to SARS-CoV-2 following the first peak of infection in England : Serial cross-sectional studies of 365,000 adults. The Lancet Regional Health. Europe, 4. https://doi.org/10.1016/j.lanepe.2021.100098

Ward, J. L., Harwood, R., Kenny, S., Cruz, J., Clark, M., Davis, P. J., Draper, E. S., Hargreaves, D., Ladhani, S. N., Gent, N., Williams, H. E., Luyt, K., Turner, S., Whittaker, E., Bottle, A., Fraser, L. K., & Viner, R. M. (2023). Pediatric Hospitalizations and ICU Admissions Due to COVID-19 and Pediatric Inflammatory Multisystem Syndrome Temporally Associated With SARS-CoV-2 in England. JAMA Pediatrics, 177(9). https://doi.org/10.1001/jamapediatrics.2023.2357

Ward, J. L., Harwood, R., Smith, C., Kenny, S., Clark, M., Davis, P. J., Draper, E. S., Hargreaves, D., Ladhani, S., Linney, M., Luyt, K., Turner, S., Whittaker, E., Fraser, L. K., & Viner, R. M. (2022). Risk factors for PICU admission and death among children and young people hospitalized with COVID-19 and PIMS-TS in England during the first pandemic year. Nature Medicine, 28(1), 193–200. https://doi.org/10.1038/s41591-021-01627-9

Warner, J. G., Portlock, J., Smith, J., & Rutter, P. (2013). Increasing seasonal influenza vaccination uptake using community pharmacies : Experience from the Isle of Wight, England. The International Journal of Pharmacy Practice, 21(6). https://doi.org/10.1111/ijpp.12037

Waterlow, N. R., Procter, S. R., van Leeuwen, E., Radhakrishnan, S., Jit, M., & Eggo, R. M. (2023). The potential cost-effectiveness of next generation influenza vaccines in England and Wales : A modelling analysis. Vaccine, 41(41). https://doi.org/10.1016/j.vaccine.2023.08.031

Webster, H. H., Nyberg, T., Sinnathamby, M. A., Aziz, N. A., Ferguson, N., Seghezzo, G., Blomquist, P. B., Bridgen, J., Chand, M., Groves, N., Myers, R., Hope, R., Ashano, E., Lopez-Bernal, J., De Angelis, D., Dabrera, G., Presanis, A. M., & Thelwall, S. (2022). Hospitalisation and mortality risk of SARS-COV-2 variant omicron sub-lineage BA.2 compared to BA.1 in England. Nature Communications, 13(1). https://doi.org/10.1038/s41467-022-33740-9

Wei, Y., Harriman, N. W., Piltch-Loeb, R., Testa, M. A., & Savoia, E. (2022). Exploring the Association between Negative Emotions and COVID-19 Vaccine Acceptance : A Cross-Sectional Analysis of Unvaccinated Adults in Sweden. Vaccines, 10(10). https://doi.org/10.3390/vaccines10101695

Weil-Olivier, C., Lina, B., & GEIG Grp Expertise Information. (2011). Vaccination coverage with seasonal and pandemic influenza vaccines in children in France, 2009-2010 season. VACCINE, 29(40), 7075–7079. https://doi.org/10.1016/j.vaccine.2011.07.018

Weinberger, T., Steffen, J., Osterman, A., Mueller, T. T., Muenchhoff, M., Wratil, P. R., Graf, A., Krebs, S., Quartucci, C., Spaeth, P. M., Grabein, B., Adorjan, K., Blum, H., Keppler, O. T., & Klein, M. (2021). Prospective Longitudinal Serosurvey of Healthcare Workers in the First Wave of the Severe Acute Respiratory Syndrome Coronavirus 2 (SARS-CoV-2) Pandemic in a Quaternary Care Hospital in Munich, Germany. Clinical

Infectious Diseases: An Official Publication of the Infectious Diseases Society of America, 73(9), e3055–e3065. https://doi.org/10.1093/cid/ciaa1935

Weinert, M., Diekmannshemke, J., Braegelmann, K., Batram, M., Witte, J., Scholz, S., Mehta, D., Ultsch, B., Dobrindt, K., & Gottlieb, J. (2025). Respiratory Syncytial Virus (RSV) as a Secondary Diagnosis among Hospitalized Patients in Germany: Outcomes and Economic Burden. Infectious Diseases and Therapy, 14(6), 1299–1312. https://doi.org/10.1007/s40121-025-01152-z

Weinmann-Menke, J., Wendtner, C.-M., Häckl, D., Lohe, V., Dang, P. L., Jah, F., & Kneidinger, N. (2025). Burden of COVID-19 in immunocompromised patients in Germany: A retrospective, observational Study on Health Insurance Data from 2021 to 2022. Infection. https://doi.org/10.1007/s15010-025-02516-w

Welsh, C. E., Albani, V., Matthews, F. E., & Bambra, C. (2021). The effects of the first national lockdown in England on geographical inequalities in the evolution of COVID-19 case rates : An ecological study. medRxiv, (Welsh C.E., Claire.Welsh@newcastle.ac.uk; Albani V.; Matthews F.E.; Bambra C.) Population Health Sciences Institute, Newcastle University, United Kingdom. https://doi.org/10.1101/2021.11.09.21266122

Werner, P., Kesik-Brodacka, M., Nowak, K., Olszewski, R., Kaleta, M., & Liebers, D. (2022). Modeling the Spatial and Temporal Spread of COVID-19 in Poland Based on a Spatial Interaction Model. ISPRS INTERNATIONAL JOURNAL OF GEO-INFORMATION, 11(3). https://doi.org/10.3390/ijgi11030195

Werner, P., Skrynyk, O., Porczek, M., Szczepankowska-Bednarek, U., Olszewski, R., & Kesik-Brodacka, M. (2021). The Effects of Climate and Bioclimate on COVID-19 Cases in Poland. REMOTE SENSING, 13(23). https://doi.org/10.3390/rs13234946

Wessendorf, L., Richter, E., Schulte, B., Schmithausen, R., Exner, M., Lehmann, N., Coenen, M., Fuhrmann, C., Kellings, A., Hüsing, A., Jöckel, K., & Streeck, H. (2022). Dynamics, outcomes and prerequisites of the first SARS-CoV-2 superspreading event in Germany in February 2020 : A cross-sectional epidemiological study. BMJ OPEN, 12(4). https://doi.org/10.1136/bmjopen-2021-059809

Westhaus, S., Weber, F.-A., Schiwy, S., Linnemann, V., Brinkmann, M., Widera, M., Greve, C., Janke, A., Hollert, H., Wintgens, T., & Ciesek, S. (2021). Detection of SARS-CoV-2 in raw and treated wastewater in Germany—Suitability for COVID-19 surveillance and potential transmission risks. The Science of the Total Environment, 751. https://doi.org/10.1016/j.scitotenv.2020.141750

Whitaker, H. J., Hassell, K., Hoschler, K., Power, L., Stowe, J., Boddington, N. L., Tsang, C., Zhao, H., Linley, E., Button, E., Okusi, C., Aspden, C., Byford, R., deLusignan, S., Amirthalingam, G., Zambon, M., Andrews, N. J., & Watson, C. (2024). Influenza vaccination during the 2021/22 season : A data-linkage test-negative case-control study of effectiveness against influenza requiring emergency care in England and serological analysis of primary care patients. Vaccine, 42(7). https://doi.org/10.1016/j.vaccine.2024.02.006

White, L., Waris, M., Cane, P., Nokes, D., & Medley, G. (2005). The transmission dynamics of groups A and B human respiratory syncytial virus (hRSV) in England & Wales and Finland : Seasonality and cross-protection. EPIDEMIOLOGY AND INFECTION, 133(2). https://doi.org/10.1017/S0950268804003450

Wichmann, O., Stöcker, P., Poggensee, G., Altmann, D., Walter, D., Hellenbrand, W., Krause, G., & Eckmanns, T. (2010). Pandemic influenza A(H1N1) 2009 breakthrough infections and estimates of vaccine effectiveness in Germany 2009-2010. EUROSURVEILLANCE, 15(18), 2–5.

Wick, M., Poshtiban, A., Kramer, R., Bangert, M., Lange, M., Wetzke, M., & Damm, O. (2023). Inpatient burden of respiratory syncytial virus in children ≤2 years of age in Germany : A retrospective analysis of nationwide hospitalization data, 2019-2022. Influenza and Other Respiratory Viruses, 17(11). https://doi.org/10.1111/irv.13211

Widgren, K., Magnusson, M., Hagstam, P., Widerström, M., Örtqvist, Å., Einemo, I. M., Follin, P., Lindblom, A., Mäkitalo, S., Wik, O., Österlund, A., Grünewald, M., Uhnoo, I., & Linde, A. (2013). Prevailing effectiveness of the 2009 influenza A(H1N1)pdm09 vaccine during the 2010/11 season in Sweden. Euro Surveillance: Bulletin Europeen Sur Les Maladies Transmissibles = European Communicable Disease Bulletin, 18(15). http://www.ncbi.nlm.nih.gov/pubmed/23594578

Widgren, K., Nielsen, J., & Mølbak, K. (2010). Registry-based surveillance of influenza-associated hospitalisations during the 2009 influenza pandemic in Denmark: The hidden burden on the young. PloS One, 5(11), e13939. https://doi.org/10.1371/journal.pone.0013939

Wieching, A., Benser, J., Kohlhauser-Vollmuth, C., Weissbrich, B., Streng, A., & Liese, J. G. (2012). Clinical characteristics of pediatric hospitalizations associated with 2009 pandemic influenza A (H1N1) in Northern Bavaria, Germany. BMC Research Notes, 5, 304. https://doi.org/10.1186/1756-0500-5-304

Wiegand, H. F., Maicher, B., Rueb, M., Wessels, P., Besteher, B., Hellwig, S., Pfennig, A., Rohner, H., Unterecker, S., Hölzel, L. P., Philipsen, A., Domschke, K., Falkai, P., Lieb, K., & Adorjan, K. (2022). COVID-19 vaccination rates in hospitalized mentally ill patients compared to the general population in Germany : Results from the COVID Ψ Vac study. European Psychiatry: The Journal of the Association of European Psychiatrists, 65(1). https://doi.org/10.1192/j.eurpsy.2022.33

Wieland, T. (2020). A phenomenological approach to assessing the effectiveness of COVID-19 related nonpharmaceutical interventions in Germany. Safety Science, 131. https://doi.org/10.1016/j.ssci.2020.104924

Wielders, C., van Lier, E., van't Klooster, T., van Gageldonk-Lafeber, A., van den Wijngaard, C., Haagsma, J., Donker, G., Meijer, A., van der Hoek, W., Lugnér, A., Kretzschmar, M., & van der Sande, M. (2012). The burden of 2009 pandemic influenza A(H1N1) in the Netherlands. EUROPEAN JOURNAL OF PUBLIC HEALTH, 22(1), 150–157. https://doi.org/10.1093/eurpub/ckq187

Wiese-Posselt, M., Leitmeyer, K., Hamouda, O., Bocter, N., Zöllner, I., Haas, W., & Ammon, A. (2006). Influenza vaccination coverage in adults belonging to defined target groups, Germany, 2003/2004. Vaccine, 24(14). https://doi.org/10.1016/j.vaccine.2005.12.020

Wijnans, L., Dieleman, J., Voordouw, B., & Sturkenboom, M. (2013). Effectiveness of MF59TM adjuvanted influenza A(H1N1)pdm09 vaccine in risk groups in the Netherlands. PloS One, 8(4). https://doi.org/10.1371/journal.pone.0063156

Wilde, H., Tomlinson, C., Mateen, B. A., Selby, D., Kanthimathinathan, H. K., Denaxas, S., Flaxman, S., Vollmer, S., Pagel, C., Brown, K., & CVD-COVID-UK/COVID-IMPACT Consortium. (2025). Trends in Pediatric Hospital Admissions Caused or Contributed by SARS-CoV-2 Infection in England. The Journal of Pediatrics, 276. https://doi.org/10.1016/j.jpeds.2024.114370

Wilde, H., Tomlinson, C., Mateen, B. A., Selby, D., Kanthimathinathan, H. K., Ramnarayan, P., Du Pre, P., Johnson, M., Pathan, N., Gonzalez-Izquierdo, A., Lai, A. G., Gurdasani, D., Pagel, C., Denaxas, S., Vollmer, S., Brown, K., & CVD-COVID-UK/COVID-IMPACT consortium. (2023). Hospital admissions linked to SARS-CoV-2 infection in children and adolescents : Cohort study of 3.2 million first ascertained infections in England. BMJ (Clinical Research Ed.), 382. https://doi.org/10.1136/bmj-2022-073639

Wilhelm, A., Schoth, J., Meinert-Berning, C., Agrawal, S., Bastian, D., Orschler, L., Ciesek, S., Teichgräber, B., Wintgens, T., Lackner, S., Weber, F.-A., & Widera, M. (2022). Wastewater surveillance allows early detection of SARS-CoV-2 omicron in North Rhine-Westphalia, Germany. The Science of the Total Environment, 846. https://doi.org/10.1016/j.scitotenv.2022.157375

Wilking, H., Buda, S., Von Der Lippe, E., Altmann, D., Krause, G., Eckmanns, T., & Haas, W. (2010). Mortality of 2009 pandemic influenza a (H1N1) in Germany. Eurosurveillance, 15(49), 5. https://doi.org/10.2807/ese.15.49.19741-en

Willem, L., Abrams, S., Franco, N., Coletti, P., Libin, P. J. K., Wambua, J., Couvreur, S., André, E., Wenseleers, T., Mao, Z., Torneri, A., Faes, C., Beutels, P., & Hens, N. (2024). The impact of quality-adjusted life years on evaluating COVID-19 mitigation strategies : Lessons from age-specific vaccination roll-out and variants of concern in Belgium (2020-2022). BMC Public Health, 24(1). https://doi.org/10.1186/s12889-024-18576-w

Williams, C. J., Schweiger, B., Diner, G., Gerlach, F., Haaman, F., Krause, G., Nienhaus, A., & Buchholz, U. (2010). Seasonal influenza risk in hospital healthcare workers is more strongly associated with household than occupational exposures : Results from a prospective cohort study in Berlin, Germany, 2006/07. BMC Infectious Diseases, 10. https://doi.org/10.1186/1471-2334-10-8

Williams, S., Crookes, A., Glass, K., & Glass, A. (2021). COVID-19 mortalities in England and Wales and the Peltzman offsetting effect. APPLIED ECONOMICS, 53(60). https://doi.org/10.1080/00036846.2021.1955089

Wojczyk, M., Niewiadomska, E., & Kowalska, M. (2023). The Incidence Proportion of SARS-CoV-2 Infections and the Percentage of Deaths among Infected Healthcare Workers in Poland. Journal of Clinical Medicine, 12(11). https://doi.org/10.3390/jcm12113714

Wolffram, D., Abbott, S., An der Heiden, M., Funk, S., Günther, F., Hailer, D., Heyder, S., Hotz, T., van de Kassteele, J., Küchenhoff, H., Müller-Hansen, S., Syliqi, D., Ullrich, A., Weigert, M., Schienle, M., & Bracher, J. (2023). Collaborative nowcasting of COVID-19 hospitalization incidences in Germany. PLoS Computational Biology, 19(8). https://doi.org/10.1371/journal.pcbi.1011394

Wollschläger, D., Gianicolo, E., Blettner, M., Hamann, R., Herm-Stapelberg, N., & Schoeps, M. (2021). Association of COVID-19 mortality with COVID-19 vaccination rates in Rhineland-Palatinate (Germany) from calendar week 1 to 20 in the year 2021 : A registry-based analysis. European Journal of Epidemiology, 36(12). https://doi.org/10.1007/s10654-021-00825-6

Woodland, L., Hodson, A., Webster, R. K., Amlôt, R., Smith, L. E., & Rubin, J. (2022). A Qualitative Study Evaluating the Factors Affecting Families' Adherence to the First COVID-19 Lockdown in England Using the COM-B Model and TDF. International Journal of Environmental Research and Public Health, 19(12). https://doi.org/10.3390/ijerph19127305

Worcel, A., Ali, B. M., Ramos-Pascual, S., Stirling, P., & Chary, F. G. (2021). Low mortality from COVID-19 at a nursing facility in France following a combined preventive and active treatment protocol. Annals of Palliative Medicine, 10(11). https://doi.org/10.21037/apm-21-1707

Wouterse, B., Geisler, J., Bär, M., & van Doorslaer, E. (2023). Has COVID-19 increased inequality in mortality by income in the Netherlands? Journal of Epidemiology and Community Health, 77(4). https://doi.org/10.1136/jech-2022-219845

Wouterse, B., Ram, F., & van Baal, P. (2022). Quality-Adjusted Life-Years Lost Due to COVID-19 Mortality : Methods and Application for The Netherlands. Value in Health: The Journal of the International Society for Pharmacoeconomics and Outcomes Research, 25(5). https://doi.org/10.1016/j.jval.2021.12.008

Wurtz, N., Revol, O., Jardot, P., Giraud-Gatineau, A., Houhamdi, L., Soumagnac, C., Annessi, A., Lacoste, A., Colson, P., Aherfi, S., & Scola, B. L. (2021). Monitoring the Circulation of SARS-CoV-2 Variants by Genomic Analysis of Wastewater in Marseille, South-East France. Pathogens (Basel, Switzerland), 10(8). https://doi.org/10.3390/pathogens10081042

Wurtzer, S., Marechal, V., Mouchel, J. M., Maday, Y., Teyssou, R., Richard, E., Almayrac, J. L., & Moulin, L. (2020). Evaluation of lockdown effect on SARS-CoV-2 dynamics through viral genome quantification in waste water, Greater Paris, France, 5 March to 23 April 2020. Euro Surveillance: Bulletin Europeen Sur Les Maladies Transmissibles = European Communicable Disease Bulletin, 25(50). https://doi.org/10.2807/1560-7917.ES.2020.25.50.2000776

Xu, C., Pang, Y., Liu, Z., Shen, J., Liao, M., & Li, P. (2024). Insights into COVID-19 stochastic modelling with effects of various transmission rates : Simulations with real statistical data from UK, Australia, Spain, and India. PHYSICA SCRIPTA, 99(2). https://doi.org/10.1088/1402-4896/ad186c

Yakubenko, S. (2021). Home alone ? Effect of weather-induced behaviour on spread of SARS-CoV-2 in Germany. Economics and Human Biology, 42. https://doi.org/10.1016/j.ehb.2021.100998

Yang, H. M., Lombardi Junior, L. P., Castro, F. F. M., & Yang, A. C. (2021). Mathematical modeling of the transmission of SARS-CoV-2-Evaluating the impact of isolation in São Paulo State (Brazil) and lockdown in Spain associated with protective measures on the epidemic of CoViD-19. PloS One, 16(6). https://doi.org/10.1371/journal.pone.0252271

Yang, J., Andersen, K. M., Rai, K. K., Tritton, T., Mugwagwa, T., Reimbaeva, M., Tsang, C., McGrath, L. J., Payne, P., Backhouse, B. E., Mendes, D., Butfield, R., Naicker, K., Araghi, M., Wood, R., & Nguyen, J. L. (2023). Healthcare resource utilisation and costs of hospitalisation and primary care among adults with COVID-

19 in England : A population-based cohort study. BMJ Open, 13(12). https://doi.org/10.1136/bmjopen-2023-075495

Yang, J., Andersen, K. M., Rai, K. K., Tritton, T., Mugwagwa, T., Tsang, C., Reimbaeva, M., McGrath, L. J., Payne, P., Backhouse, B., Mendes, D., Butfield, R., Wood, R., & Nguyen, J. L. (2024). Health Care Resource Utilization and Costs Associated With COVID-19 Among Pediatrics Managed in the Community or Hospital Setting in England : A Population-based Cohort Study. The Pediatric Infectious Disease Journal, 43(3). https://doi.org/10.1097/INF.0000000000004213

Yang, J., Rai, K. K., Alfred, T., Massey, L., Massey, O., McGrath, L., Andersen, K. M., Tritton, T., Tsang, C., Butfield, R., Reynard, C., Mendes, D., & Nguyen, J. L. (2025). The impact of COVID vaccination on incidence of long COVID and healthcare resource utilisation in a primary care cohort in England, 2021-2022. BMC Infectious Diseases, 25(1). https://doi.org/10.1186/s12879-024-10097-6

Yang, J., Rai, K. K., Seif, M., Volkman, H. R., Ren, J., Schmetz, A., Gowman, H., Massey, L., Pather, S., & Nguyen, J. L. (2024). COVID-19-Related Work Absenteeism and Associated Lost Productivity Cost in Germany : A Population-Based Study. Journal of Occupational and Environmental Medicine, 66(6). https://doi.org/10.1097/JOM.0000000000003093

Yang, J., Tamberou, C., Arnee, E., Squara, P.-A., Boukhlal, A., Nguyen, J. L., Volkman, H. R., Fievez, S., Lepoutre-Bourguet, M., Ben Romdhane, H., Renaudat, C., Crépey, P., & Robineau, O. (2025). Quantifying all-cause healthcare resource utilization and costs of children with mild-to-moderate long COVID in France. Journal of Medical Economics, 28(1), 1002–1013. https://doi.org/10.1080/13696998.2025.2525002

Yang, J., Tamberou, C., Arnee, E., Squara, P.-A., Boukhlal, A., Nguyen, J. L., Volkman, H. R., Fiévez, S., Lepoutre-Bourguet, M., Ren, J., Ben Romdhane, H., Crépey, P., & Robineau, O. (2025). All-cause healthcare resource utilization and costs among community-managed adults with long-COVID in France, 2020-2023. Journal of Medical Economics, 28(1), 535–543. https://doi.org/10.1080/13696998.2025.2485626

Yates, T., Summerfield, A., Razieh, C., Banerjee, A., Chudasama, Y., Davies, M. J., Gillies, C., Islam, N., Lawson, C., Mirkes, E., Zaccardi, F., Khunti, K., & Nafilyan, V. (2022). A population-based cohort study of obesity, ethnicity and COVID-19 mortality in 12.6 million adults in England. Nature Communications, 13(1). https://doi.org/10.1038/s41467-022-28248-1

Yin, X., Aiken, J. M., Harris, R., & Bamber, J. L. (2024). A Bayesian spatio-temporal model of COVID-19 spread in England. Scientific Reports, 14(1). https://doi.org/10.1038/s41598-024-60964-0

Zaczyński, A., Hampel, M., Piątkiewicz, P., Nasiłowski, J., Butkiewicz, S., Religioni, U., Barańska, A., Herda, J., Neumann-Podczaska, A., Vaillancourt, R., & Merks, P. (2023). The Course of COVID-19 among Unvaccinated Patients-Data from the National Hospital in Warsaw, Poland. Vaccines, 11(3). https://doi.org/10.3390/vaccines11030675

Zaczyński, A., Hampel, M., Piątkiewicz, P., Nasiłowski, J., Butkiewicz, S., Religioni, U., Barańska, A., Malm, M., Neumann-Podczaska, A., Vaillancourt, R., & Merks, P. (2023). Clinical Course of 53 Previously Vaccinated Patients Admitted to the National Hospital in Warsaw, Poland with COVID-19 Between November 2021 and March 2022. Medical Science Monitor: International Medical Journal of Experimental and Clinical Research, 29. https://doi.org/10.12659/MSM.939841

Zaidi, A., Harris, R., Hall, J., Woodhall, S., Andrews, N., Dunbar, K., Lopez-Bernal, J., & Dabrera, G. (2023). Effects of Second Dose of SARS-CoV-2 Vaccination on Household Transmission, England. Emerging Infectious Diseases, 29(1). https://doi.org/10.3201/eid2901.220996

Zaino, A., Bonacquisti, M., Russo, L., Sabetta, T., Solipaca, A., Ricciardi, W., Boccia, S., & Villani, L. (2025). Influenza vaccination coverages in Italy from 1999/00 to 2023/24: A joinpoint regression analysis. JOURNAL OF INFECTION AND PUBLIC HEALTH, 18(10). https://doi.org/10.1016/j.jiph.2025.102875

Zaitri, M., Bibi, M., & Torres, D. (2021). Optimal control to limit the spread of COVID-19 in Italy. KUWAIT JOURNAL OF SCIENCE. https://doi.org/10.48129/kjs.splcov.13961

Zaldo-Aubanell, Q., Campillo I López, F., Bach, A., Serra, I., Olivet-Vila, J., Saez, M., Pino, D., & Maneja, R. (2021). Community Risk Factors in the COVID-19 Incidence and Mortality in Catalonia (Spain). A Population-

Based Study. International Journal of Environmental Research and Public Health, 18(7). https://doi.org/10.3390/ijerph18073768

Zamorano-Leon, J. J., Jimenez-Garcia, R., Lopez-de-Andres, A., de-Miguel-Diez, J., Carabantes-Alarcon, D., Albaladejo-Vicente, R., Villanueva-Orbaiz, R., Zekri-Nechar, K., & Sanz-Rojo, S. (2021). Low Levels of Influenza Vaccine Uptake among the Diabetic Population in Spain : A Time Trend Study from 2011 to 2020. Journal of Clinical Medicine, 11(1). https://doi.org/10.3390/jcm11010068

Zanchi, S., La Greca, C., Forgione, C., Bettari, L., Cortinovis, S., Pero, G., Pecora, D., Botti, P., Bnà, C., & Cuccia, C. (2021). Pulmonary embolism and SARS-CoV-2 : Analysis of the characteristics of patients admitted to a center in northern Italy for pulmonary embolism associated with COVID-19. GIORNALE ITALIANO DI CARDIOLOGIA, 22(9).

Zangrillo, A., Beretta, L., Scandroglio, A. M., Monti, G., Fominskiy, E., Colombo, S., Morselli, F., Belletti, A., Silvani, P., Crivellari, M., Monaco, F., Azzolini, M. L., Reineke, R., Nardelli, P., Sartorelli, M., Votta, C. D., Ruggeri, A., Ciceri, F., De Cobelli, F., … COVID-BioB Study Group. (2020). Characteristics, treatment, outcomes and cause of death of invasively ventilated patients with COVID-19 ARDS in Milan, Italy. Critical Care and Resuscitation: Journal of the Australasian Academy of Critical Care Medicine, 22(3). https://doi.org/10.1016/S1441-2772(23)00387-3

Zarbo, C., Candini, V., Ferrari, C., d'Addazio, M., Calamandrei, G., Starace, F., Caserotti, M., Gavaruzzi, T., Lotto, L., Tasso, A., Zamparini, M., & de Girolamo, G. (2022). COVID-19 Vaccine Hesitancy in Italy : Predictors of Acceptance, Fence Sitting and Refusal of the COVID-19 Vaccination. Frontiers in Public Health, 10. https://doi.org/10.3389/fpubh.2022.873098

Zavala, M., Ireland, G., Amin-Chowdhury, Z., Ramsay, M. E., & Ladhani, S. N. (2022). Acute and Persistent Symptoms in Children With Polymerase Chain Reaction (PCR)-Confirmed Severe Acute Respiratory Syndrome Coronavirus 2 (SARS-CoV-2) Infection Compared With Test-Negative Children in England : Active, Prospective, National Surveillance. Clinical Infectious Diseases: An Official Publication of the Infectious Diseases Society of America, 75(1). https://doi.org/10.1093/cid/ciab991

Zayet, S., Zahra, H., Royer, P.-Y., Tipirdamaz, C., Mercier, J., Gendrin, V., Lepiller, Q., Marty-Quinternet, S., Osman, M., Belfeki, N., Toko, L., Garnier, P., Pierron, A., Plantin, J., Messin, L., Villemain, M., Bouiller, K., & Klopfenstein, T. (2021). Post-COVID-19 Syndrome : Nine Months after SARS-CoV-2 Infection in a Cohort of 354 Patients : Data from the First Wave of COVID-19 in Nord Franche-Comté Hospital, France. Microorganisms, 9(8). https://doi.org/10.3390/microorganisms9081719

Zeevat, F., van der Pol, S., Westra, T., Beck, E., Postma, M. J., & Boersma, C. (2025). Cost-effectiveness Analysis of COVID-19 mRNA XBB.1.5 Fall 2023 Vaccination in the Netherlands. Advances in Therapy. https://doi.org/10.1007/s12325-025-03112-y

Zehender, G., Pariani, E., Piralla, A., Lai, A., Gabanelli, E., Ranghiero, A., Ebranati, E., Amendola, A., Campanini, G., Rovida, F., Ciccozzi, M., Galli, M., Baldanti, F., & Zanetti, A. R. (2012). Reconstruction of the Evolutionary Dynamics of the A(H1N1)pdm09 Influenza Virus in Italy during the Pandemic and Post-Pandemic Phases. PLoS ONE, 7(11). https://doi.org/10.1371/journal.pone.0047517

Zeitoun, J.-D., Faron, M., & Lefèvre, J. H. (2020). Impact of the local care environment and social characteristics on aggregated hospital fatality rate from COVID-19 in France : A nationwide observational study. Public Health, 189. https://doi.org/10.1016/j.puhe.2020.09.015

Zhan, C., Tse, C., Lai, Z., Hao, T., & Su, J. (2020). Prediction of COVID-19 spreading profiles in South Korea, Italy and Iran by data-driven coding. PLOS ONE, 15(7). https://doi.org/10.1371/journal.pone.0234763.r004

Zhang, H., Thygesen, J. H., Shi, T., Gkoutos, G. V., Hemingway, H., Guthrie, B., Wu, H., & Genomics England Research Consortium. (2022). Increased COVID-19 mortality rate in rare disease patients : A retrospective cohort study in participants of the Genomics England 100,000 Genomes project. Orphanet Journal of Rare Diseases, 17(1). https://doi.org/10.1186/s13023-022-02312-x

Zhang, J., Lim, Y.-H., Chen, J., Hyman, S., Cole-Hunter, T., Tuffier, S., Napolitano, G., Bergmann, M., Mortensen, L. H., Brandt, J., Mueller, N., Barboza, E. P., Hoogh, K. de, Hoek, G., Loft, S., & Andersen, Z. J.

(2024). Residential greenspace and COVID-19 morbidity and mortality : A nationwide cohort study in Denmark. Environment International, 194. https://doi.org/10.1016/j.envint.2024.109173

Zhang, X., Hungerford, D., Green, M. A., García-Fiñana, M., Buchan, I. E., & Barr, B. (2024). Evaluating the impacts of tiered restrictions introduced in England in December 2020 on covid-19 hospitalisations : A synthetic control study. medRxiv, (Zhang X., xingna.zhang@liverpool.ac.uk; Buchan I.E.; Barr B.) Public Health, Policy and Systems, Institute of Population Health, University of Liverpool, Liverpool, United Kingdom. https://doi.org/10.1101/2024.02.28.24303487

Zhang, Z., Fu, D., & Wang, J. (2023). How containment policy and medical service impact COVID-19 transmission : A cross-national comparison among China, the USA, and Sweden. International Journal of Disaster Risk Reduction: IJDRR, 91. https://doi.org/10.1016/j.ijdrr.2023.103685

Zhao, H., Green, H., Lackenby, A., Donati, M., Ellis, J., Thompson, C., Bermingham, A., Field, J., Sebastianpillai, P., Zambon, M., Watson, J., & Pebody, R. (2014). A new laboratory-based surveillance system (Respiratory DataMart System) for influenza and other respiratory viruses in England : Results and experience from 2009 to 2012. Euro Surveillance: Bulletin Europeen Sur Les Maladies Transmissibles = European Communicable Disease Bulletin, 19(3). https://doi.org/10.2807/1560-7917.es2014.19.3.20680

Zhao, H., Harris, R., Ellis, J., & Pebody, R. (2015). Ethnicity, deprivation and mortality due to 2009 pandemic influenza A(H1N1) in England during the 2009/2010 pandemic and the first post-pandemic season. EPIDEMIOLOGY AND INFECTION, 143(16), 3375–3383. https://doi.org/10.1017/S0950268815000576

Zhelyazkova, A., Kim, S., Klein, M., Prueckner, S., Horster, S., Kressirer, P., Choukér, A., Coenen, M., & Adorjan, K. (2022). COVID-19 Vaccination Intent, Barriers and Facilitators in Healthcare Workers : Insights from a Cross-Sectional Study on 2500 Employees at LMU University Hospital in Munich, Germany. Vaccines, 10(8). https://doi.org/10.3390/vaccines10081231

Ziauddeen, H., Subramaniam, N., & Gurdasani, D. (2021). Modelling the impact of lockdown-easing measures on cumulative COVID-19 cases and deaths in England. BMJ Open, 11(9). https://doi.org/10.1136/bmjopen-2020-042483

Ziegler, L., Lundström, A., Havervall, S., Thålin, C., & Gigante, B. (2022). IL-6 signalling biomarkers in hospitalised patients with moderate to severe SARS-CoV-2 infection in a single centre study in Sweden. Cytokine, 159. https://doi.org/10.1016/j.cyto.2022.156020

Ziuzia-Januszewska, L., Januszewski, M., Sosnowska-Nowak, J., Janiszewski, M., Dobrzyński, P., Jakimiuk, A. A., & Jakimiuk, A. J. (2022). COVID-19 Severity and Mortality in Two Pandemic Waves in Poland and Predictors of Poor Outcomes of SARS-CoV-2 Infection in Hospitalized Young Adults. Viruses, 14(8), 1700. https://doi.org/10.3390/v14081700

Zlateva, K. T., Vijgen, L., Dekeersmaeker, N., Naranjo, C., & Van Ranst, M. (2007). Subgroup prevalence and genotype circulation patterns of human respiratory syncytial virus in Belgium during ten successive epidemic seasons. Journal of Clinical Microbiology, 45(9). https://doi.org/10.1128/JCM.00339-07

Zolotusca, L., Jorgensen, P., Popovici, O., Pistol, A., Popovici, F., Widdowson, M.-A., Alexandrescu, V., Ivanciuc, A., Cheng, P.-Y., Gross, D., Brown, C. S., & Mott, J. A. (2014). Risk factors associated with fatal influenza, Romania, October 2009-May 2011. Influenza and Other Respiratory Viruses, 8(1), 8–12. https://doi.org/10.1111/irv.12209

Zongo, P., Zorom, M., Mophou, G., Dorville, R., & Beaumont, C. (2020). A model of COVID-19 transmission to understand the effectiveness of the containment measures : Application to data from France. Epidemiology and Infection, 148. https://doi.org/10.1017/S0950268820002162

Zoran, M. A., Savastru, R. S., Savastru, D. M., & Tautan, M. N. (2020). Assessing the relationship between surface levels of PM2.5 and PM10 particulate matter impact on COVID-19 in Milan, Italy. The Science of the Total Environment, 738. https://doi.org/10.1016/j.scitotenv.2020.139825

Zoran, M. A., Savastru, R. S., Savastru, D. M., & Tautan, M. N. (2022). Cumulative effects of air pollution and climate drivers on COVID-19 multiwaves in Bucharest, Romania. Process Safety and Environmental Protection:

Transactions of the Institution of Chemical Engineers, Part B, 166, 368–383. https://doi.org/10.1016/j.psep.2022.08.042

Zoran, M. A., Savastru, R. S., Savastru, D. M., Tautan, M. N., Baschir, L. A., & Tenciu, D. V. (2022). Assessing the impact of air pollution and climate seasonality on COVID-19 multiwaves in Madrid, Spain. Environmental Research, 203. https://doi.org/10.1016/j.envres.2021.111849

Zorn, J., Simões, M., Velders, G. J. M., Gerlofs-Nijland, M., Strak, M., Jacobs, J., Dijkema, M. B. A., Hagenaars, T. J., Smit, L. A. M., Vermeulen, R., Mughini-Gras, L., Hogerwerf, L., & Klinkenberg, D. (2024). Effects of long-term exposure to outdoor air pollution on COVID-19 incidence : A population-based cohort study accounting for SARS-CoV-2 exposure levels in the Netherlands. Environmental Research, 252(Pt 1). https://doi.org/10.1016/j.envres.2024.118812

Zubcoff, J.-J., Olcina, J., Morales, J., Mazón, J.-N., & Mayoral, A. M. (2023). Usefulness of open data to determine the incidence of COVID-19 and its relationship with atmospheric variables in Spain during the 2020 lockdown. Technological Forecasting and Social Change, 186. https://doi.org/10.1016/j.techfore.2022.122108

Zuccon, W., Comassi, P., Adriani, L., Bergamaschini, G., Bertin, E., Borromeo, R., Corti, S., De Petri, F., Dolci, F., Galmozzi, A., Gigliotti, A., Gualdoni, L., Guerra, C., Khosthiova, A., Leati, G., Lupi, G., Moscato, P., Perotti, V., Piantelli, M., … Viganò, G. (2021). Intensive care for seriously ill patients affected by novel coronavirus sars—CoV - 2 : Experience of the Crema Hospital, Italy. The American Journal of Emergency Medicine, 45. https://doi.org/10.1016/j.ajem.2020.08.005

Zucs, P., Buchholz, U., Haas, W., & Uphoff, H. (2005). Influenza associated excess mortality in Germany, 1985-2001. Emerging Themes in Epidemiology, 2. https://doi.org/10.1186/1742-7622-2-6
